# Understanding the Conformational Behavior of Fluorinated Piperidines: The Origin of the Axial‐F Preference

**DOI:** 10.1002/chem.202001355

**Published:** 2020-05-12

**Authors:** Zackaria Nairoukh, Felix Strieth‐Kalthoff, Klaus Bergander, Frank Glorius

**Affiliations:** ^1^ Organisch-Chemisches Institut Westfälische Wilhelms-Universität Münster Corrensstraße 40 48149 Münster Germany

**Keywords:** conformational behavior, fluorine, NMR analysis, piperidines, solvation effect

## Abstract

Gaining an understanding of the conformational behavior of fluorinated compounds would allow for expansion of the current molecular design toolbox. In order to facilitate drug discovery efforts, a systematic survey of a series of diversely substituted and protected fluorinated piperidine derivatives has been carried out using NMR spectroscopy. Computational investigations reveal that, in addition to established delocalization forces such as charge–dipole interactions and hyperconjugation, solvation and solvent polarity play a major role. This work codifies a new design principle for conformationally rigid molecular scaffolds.

## Graphical abstract

**Fluorine ax‐plained**! The axial‐F preference of fluorinated piperidines can be attributed to delocalization forces such as charge–dipole interactions and hyperconjugation. In addition to these established forces, solvation and solvent polarity were found to play a major role in the stabilization of these entities, which was supported by experimental and computational investigations of a variety of fluorinated piperidine derivatives (see scheme).

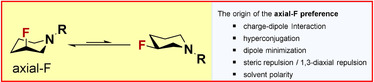

The introduction of fluorine atoms into molecules and materials across many fields of academic and industrial research is now commonplace, owing to their unique properties and effects.^1^ Therefore, the incorporation of fluorine into drug lead candidates has been recognized as a powerful strategy to improve their pharmacokinetic and physicochemical properties.^2^ For example, the high C−F bond energy increases metabolic stability^2^ and the electronic effects of fluorine allow for modification of critical properties such as the p*K*_a_.^2^ Significantly, a fine‐tuning of polarity and lipophilicity can increase solubility and membrane permeability, which may in turn increase the likelihood of success in clinical trials (Scheme 1).

**Scheme 1:**
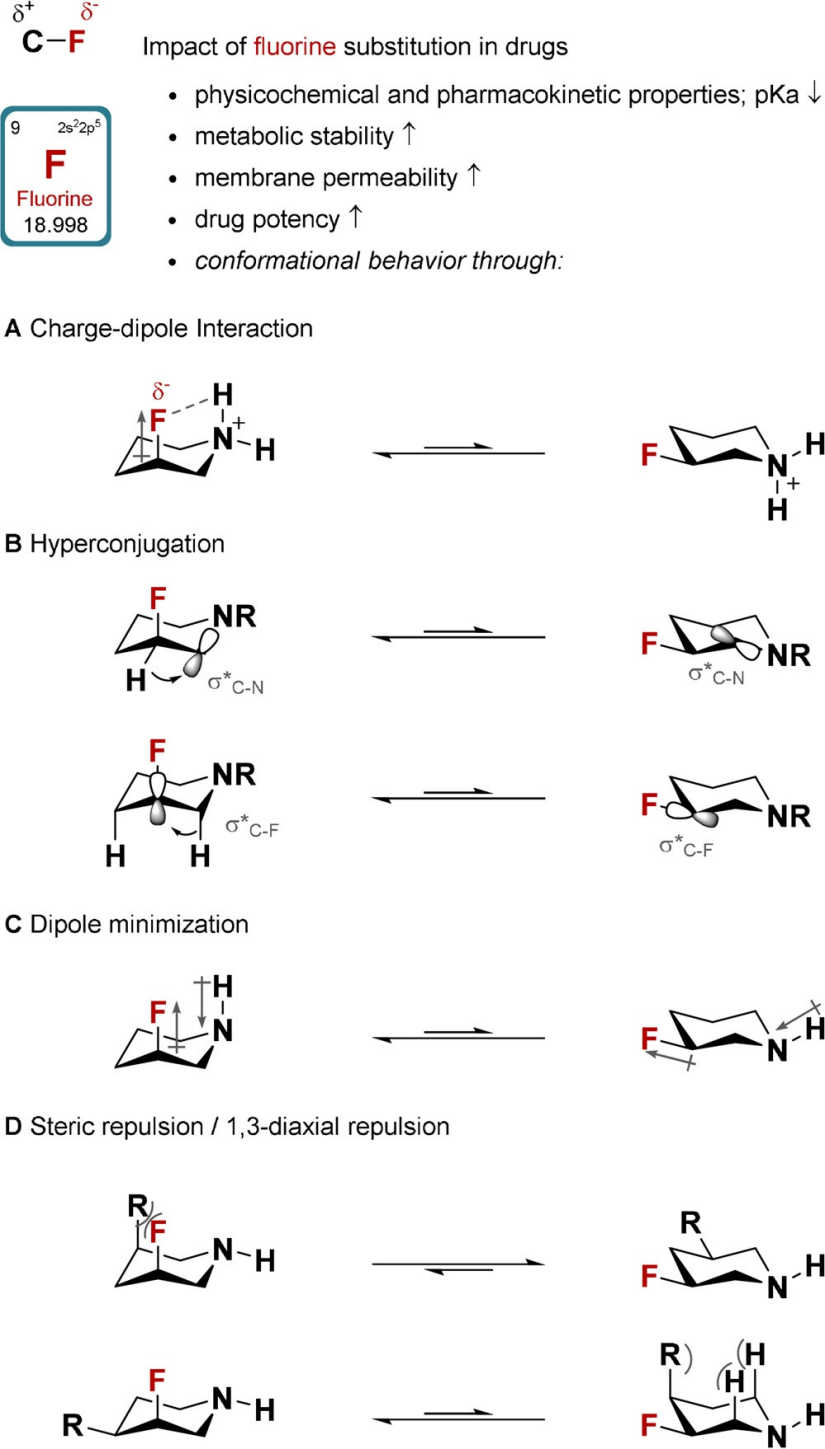
The conformational preferences of fluorinated piperidine derivatives can be attributed to A) charge‐dipole interactions, B) hyperconjugation, C) dipole minimization, and D) steric repulsion.

A particularly striking feature of fluorine substitution is its impact on the relative orientation of a C−F bond when incorporated into aliphatic carbocyclic and acyclic systems, which allows for the design of highly polar compounds.^3^ For aliphatic, heterocyclic systems, these effects can lead to more rigid structures, which enable the stabilization of well‐defined conformers. Fluorinated piperidines represent an exceptionally interesting case for these phenomena, since the piperidine moiety and related saturated *N*‐containing heterocycles are frequently present in bioactive compounds.^4^ Owing to limited synthetic access, typically via tedious, multi‐step synthesis, the study of their conformational behavior has been the subject of few reports, mainly focusing on 3‐fluoropiperidine (**1**) derivatives. For instance, the axial orientation of fluorine in the protonated 3‐fluoropiperidinium cation was mainly attributed to the occurrence of strong charge–dipole interactions (C−F⋅⋅⋅ HN^+^) (Scheme 1 A).^5^, ^6^ In addition, hyperconjugative interactions, often referred to as the fluorine *gauche* effect, can contribute to the stabilization of the axial orientation of the fluorine atom, mainly through electron donation from anti‐periplanar C−H bonds into the low‐lying σ*_C−F_ and σ*_C−N_ orbitals (Scheme 1 B).^3^, ^7^ Additional factors such as dipole minimization, steric repulsion and solvation effects have been described to partially contribute to the conformational behavior, but were considered to be the least competitive (Schemes 1 C–D).^6^, ^8^

While most studies are limited to examples of 3‐fluoropiperidine (**1**) derivatives, an extensive and systematic evaluation of the conformational effects of a wide range of substitution patterns has not been carried out until now. We believe that such a study would be highly valuable to the scientific community, in particular since even slight changes in the three‐dimensional structure might dramatically change the likelihood of success of lead compounds in therapeutic applications.^2^

We recently described a straightforward process for the preparation of fluorinated piperidines.^9^ In this reaction, fluoropyridine precursors underwent a catalytic dearomatization‐hydrogenation sequence to furnish a plethora of substituted, all‐*cis*‐(multi)fluorinated piperidines in a highly diastereoselective fashion. Within the course of this study we became interested in the conformational behavior of the newly accessed fluorinated piperidines (**1**–**12**), obtained as the trifluoroacetamide (**1A–12A**) or HCl salts (**1B–12B**). Analysis of the ^3^ *J*(^19^F,^1^H) coupling in NMR experiments allowed us to determine the relative orientation of the fluorine atom(s), which were often found to adopt either axial or equatorial orientations exclusively.^10^ In addition to the TFA and HCl analogues, we prepared an additional library of unprotected fluorinated piperidines (NH‐analogues, **1C–12C**) and studied their conformational behavior. To rationalize the conformational behavior of the fluorinated piperidine derivatives (**1**–**12**), we performed a systematic computational analysis (M06‐2X/def2‐QZVPP). Individual DFT calculations were performed in the gas phase and in solution using a polarizable continuum model (PCM, TFA analogues in CHCl_3_, HCl‐ and NH‐analogues in water). Pleasingly, the experimentally observed conformer could be predicted computationally in almost all cases. For instance, the free enthalpy differences Δ*G* between the two conformers in 3‐ fluoropiperidine (**1**) and 3,5‐difluoropiperidine (**2**) derivatives indicate a strong preference towards the F_axial_ conformation in solution (Scheme 2). Interestingly, while the axial preference in solution for HCl‐analogues (**1B**, **2B**) is mainly attributed to electrostatic interactions (Δ*E*_elect,a‐e_ for **1B** and **2B** is +12.6, +14.7 kcal mol^−1^, respectively), hyperconjugative interactions are found to play a significant role in TFA‐ (**1A**, **2A**) and NH‐analogues (**1C**, **2C**) (Δ*E*_hyperc,a‐e_ for **1A**, **1C**, **2A**, and **2C** is +3.3, +5.1, +11.7, and +10.8 kcal mol^−1^, respectively—for more details see the Supporting Information). It should be noted that the axial preference of fluorinated piperidine analogues of **1** and **2** was confirmed experimentally by NMR studies (Scheme 2). Along these lines, we also performed the same analysis on 3‐fluoro‐4‐methylpiperidine (**3**) (Scheme 3). Both computational and experimental studies showed high axial preference for all variants (TFA‐, HCl‐, and NH‐ analogues). In this particular case, we believe that in addition to the abovementioned forces (Δ*E*_elect,a‐e_ of **3A**, **3B** and **3C** is −0.8, +8.5, and +13.5 kcal mol^−1^, respectively; Δ*E*_hyperc,a‐e_ of **3A**, **3B** and **3C** is +5.0, +3.0, and +5.8 kcal mol^−1^, respectively), the steric influence of the methyl substituent (*A*_Me_=1.7 kcal mol^−1^) plays a major role in promoting the axial preference of the fluorine atom (ΔΔ*E*_steric,a‐e_ of **3A**, **3B** and **3C** is +7.5, +6.4, and +0.3 kcal mol^−1^, respectively, relative to **1A‐C**).

**Scheme 2:**
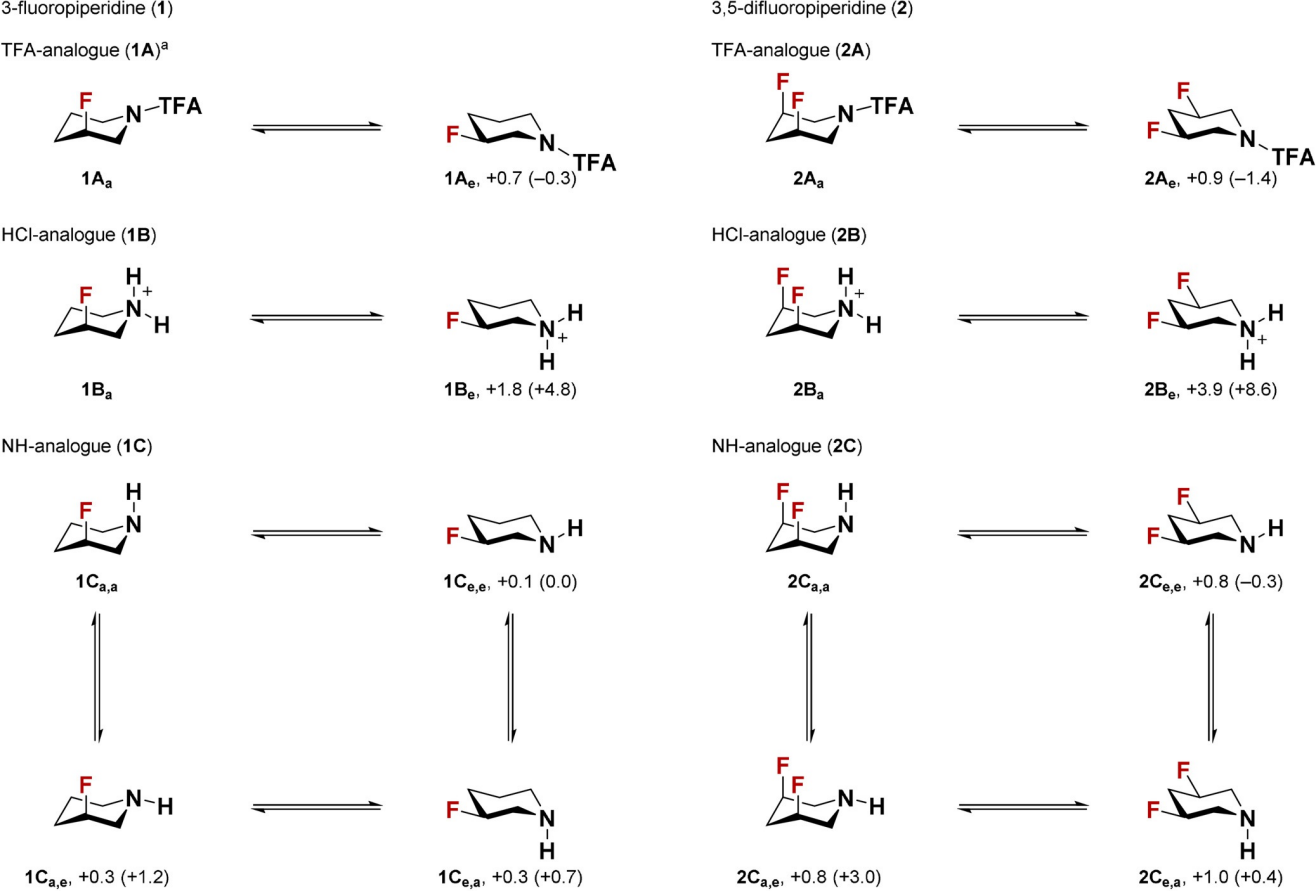
The conformational preferences of 3‐fluoropiperidine (**1**) and 3,5‐difluoropiperidine (**2**) and their TFA‐(**A**), HCl‐(**B**), and NH‐(**C**)‐analogues. The free enthalpy differences between the equatorial conformer to the axial conformer (Δ*G*) are presented as follows: Δ*G* Solvent (Δ*G* Gas Phase). The Δ*G* values for TFA‐, and for both HCl‐, and NH‐analogues are given in chloroform and water, respectively. All values are given in kcal mol^−1^. Experimentally, all analogues of **1** and **2** showed high axial preference. In NH‐analogues **1C** and **2C**, we were unable to determine the orientation of the N−H bond because of a fast H/D exchange in solution. [a] Both computational analysis and experimental observation were carried out in toluene.

**Scheme 3:**
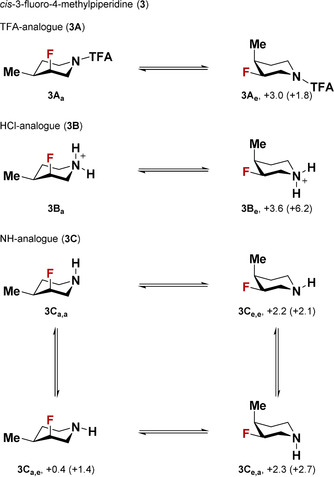
The conformational preferences of *cis*‐3‐fluoro‐4‐methylpiperidine (**3**) and its TFA‐(**A**), HCl‐(**B**), and NH‐(**C**)‐ analogues. All values are given in kcal mol^−1^. The experimental observation is based on ^3^ *J*(^19^F,^1^H) values. See the Supporting Information for more details.

Inspired by these preliminary results, we conducted the same systematic analysis for all of the newly accessed fluorinated piperidine derivatives, including all different analogues (**1**–**12**) (Table 1). The free enthalpy differences (Δ*G*), electrostatic, hyperconjugation and steric contributions including dipole moments and geometries for all conformers are presented in detail in the Supplementary Information.

**Table 1:** Conformational behavior of all‐*cis*‐(multi)fluorinated piperidines.^[a]^

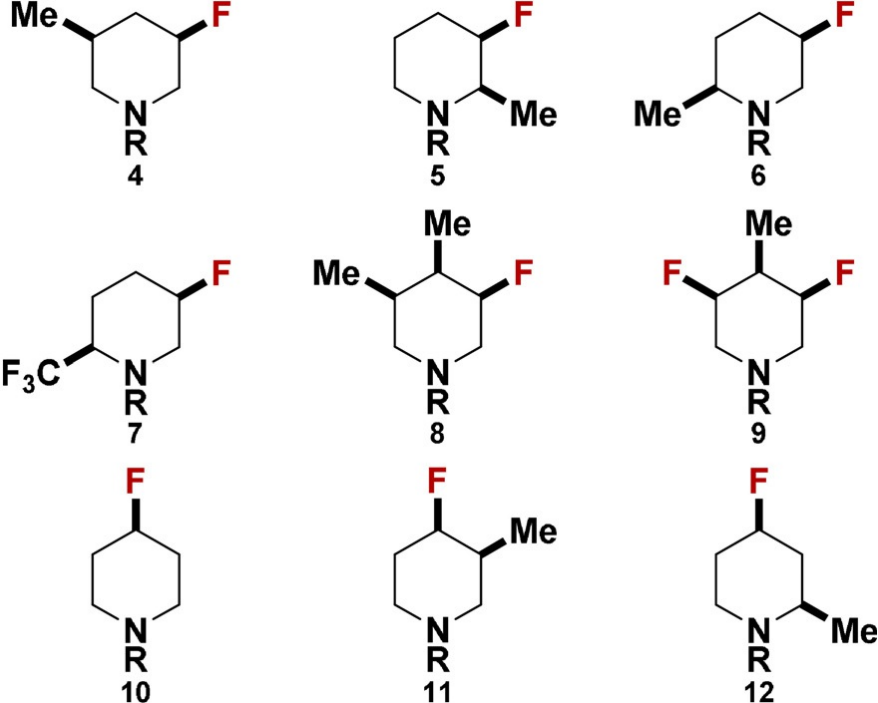				
	$\Delta G^{298}\left(a\to e\right)$ [kcal mol^−1^]			
Compd.	Gas phase	H_2_O	CHCl_3_	Exptl.
**1, A**	+0.1	–	−0.4^[a]^	axial
**B**	+4.8	+1.8	–	axial
**C**	0.0	+0.1	–	axial
**2, A**	−1.4	–	+0.9	axial
**B**	+8.6	+3.9	–	axial
**C**	−0.3	+0.8	–	axial
**3, A**	+1.8	–	+3.0	axial
**B**	+6.2	+3.6	–	axial
**C**	+2.1	+2.2	–	axial
**4, A**	−1.9	–	−1.2	equatorial
**B**	+2.9	−0.4	–	equatorial
**C**	−1.9	−2.0	–	equatorial
**5, A**	−4.3	–	−3.7	equatorial
**B**	+6.2	+3.3	–	axial
**C**	+2.5	+2.1	–	axial
**6, A**	−3.7	–	−3.3	equatorial
**B**	+6.8	+3.5	–	axial
**C**	+2.5	+2.7	–	axial
**7, A**	−6.0	–	−4.4	equatorial
**B**	+7.7	+5.2	–	axial
**C**	+1.3	+2.1	–	axial
**8, A**	+0.2	–	+0.6	axial
**B**	+4.2	+1.1	–	axial
**C**	+0.4	+0.3	–	axial
**9, A**	+0.1	–	+2.3	axial
**B**	+9.5	+5.1	–	axial
**C**	+1.7	+2.8	–	axial
**10, A**	+0.7	–	+0.4	axial
**B**	+3.0	+1.0	–	equatorial
**C**	−0.9	−0.4	–	equatorial
**11, A**	+1.5	–	+1.4	axial
**B**	+3.9	+2.3	–	axial
**C**	+0.5	+1.1	–	axial
**12, A**	+3.7	–	+5.4	axial
**B**	+0.4	−1.7	–	equatorial
**C**	−3.7	−3.7	–	equatorial

[a] The conformational preferences of fluorinated piperidine (**1**–**12**) and its R=TFA‐(**A**), HCl‐(**B**), and NH‐(**C**)‐analogues. The Δ*G* values for TFA‐ and for both HCl‐, and NH‐analogues are given in chloroform and water, respectively. All values are given in kcal mol^−1^. [b] This compound was measured in toluene.

As mentioned above, in the vast majority of cases the computed conformer free enthalpy differences in both TFA‐, HCl‐, and NH‐analogues (in solution) are in qualitative agreement with the experimentally observed conformational preferences (Table 1). In a singular event however, the supposedly less stable conformer, as derived from computational analysis, was observed experimentally. The free enthalpy difference of 4‐fluoropiperidinium salt (**10B**) in the gas phase and in aqueous solution (+3.0, +1.0 kcal mol^−1^, respectively) suggests that the axial orientation of the fluorine atom should be more favored. In aqueous solution, the equatorial conformer was observed to be dominant. This puzzling observation suggests that additional factors might play a major role in predicting the conformational preference. While examining all computational results, we realized that that the molecular dipole moment μ has a significant impact on the stabilization energy of conformers in polar solution. In the case of the 4‐fluoropiperidinium salt (**10B**), the equatorial conformer has a significantly larger dipole moment (*μ*_e,gas_=8.0 D) than the axial conformer (*μ*_a,gas_=6.4 D) and can therefore be significantly stabilized in aqueous solution. Such an effect can be observed particularly for charged species in highly polar solvents and is presumably underestimated computationally by the simple PCM.

Consequently, we became interested in examining whether solvent polarity can affect conformational behavior, as suggested by Abraham for the rotamers of ethane derivatives in the 1960s.^11^ We initially investigated whether the axial preference is preserved in 3,5‐difluoropiperidine (**2C**) in different solvents (See the Supplementary Information for more details). Both computational and experimental analyses showed that the fluorine atoms adopt an exclusively axial orientation in all cases (see the Supporting Information for more details). The computational analysis however suggests an increasing stability of the more polar F_axial_ conformer with increasing solvent polarity (Δ*G*_a–e_=+0.2, +0.5, +0.6, +0.8, and +0.8 kcal mol^−1^ in C_6_H_6_, CHCl_3_, CH_2_Cl_2_, DMSO and H_2_O respectively).

To further explore these phenomena, we conducted the same analysis on 3,5‐difluoropiperidine (**2**), employing different N‐protecting groups (**13**–**15**) (Table 2). Initially, we examined the conformational behavior of the TFA‐analogue (**2A**) in different solvents and identified the same clear correlation between solvent polarity and the preference for the F_axial_ conformation; the higher the solvent polarity, the higher the preference for axial orientation (^3^ *J*(3‐F_a_,4‐H_a_) values in C_6_H_6_, CHCl_3_, CH_2_Cl_2_, and DMSO are 34.1, 36.1, 38.8, 44.4 Hz, respectively). The same observation was made while studying acetyl‐protected 3,5‐difluoropiperidine (**13**): in both chloroform and DMSO an axial preference was obtained with significantly higher values of Δ*G* and ^3^ *J*(3‐F_a_,4‐H_a_) in the more polar solvent (DMSO). Encouraged by these results, we considered applying this technique to promote the formation of the F_axial_ conformer in further 3,5‐difluoropiperidine analogues (Table 2). Computational investigations in the gas phase, as well as the experimental observation in chloroform, suggest that in both Pivaloyl‐ (Piv) and *tert*‐butoxycarbonyl (Boc)‐protected 3,5‐difluoropiperidine (**14**, **15**), the fluorine atoms adopt an equatorial orientation (^3^ *J*(3‐F_a_,4‐H_a_)=7.3, 12.5 Hz for **14** and **15** respectively). By increasing the solvent polarity from chloroform (*ϵ*=4.81) to DMSO (*ϵ*=46.7), the conformational behavior of both species can be inverted, favoring the F_axial_ conformation orientation (^3^ *J*(3‐F_a_,4‐H_a_)=38.5, 40.4 Hz for **14** and **15** respectively).

**Table 2:** The conformational preferences of 3,5‐difluoropiperidine derivatives.^[a]^

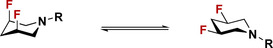					
Compd.	Solvent	$\Delta G^{298}\left(a\to e\right)$ [Kcal mol^−1^]	*μ* (a)	*μ* (e)	Exptl.
R=TFA (**2A**)	none	−1.4	6.58	2.20	–
	C_6_H_6_	+0.1	8.06	2.73	axial
	CHCl_3_	+0.9	8.94	3.04	axial
	CH_2_Cl_2_	+1.0	9.35	3.18	axial
	DMSO	+2.0	9.79	3.33	axial
R=AC (**13**)	none	−1.5	–	–	–
	CHCl_3_	+0.3	–	–	axial
	DMSO	+2.0	–	–	axial
R=Piv (**14**)	none	−2.4	–	–	–
	CHCl_3_	−0.9	–	–	equatorial
	DMSO	+2.0	–	–	axial
R=Boc (**15**)	none	−2.4	–	–	–
	CHCl_3_	−0.9	–	–	equatorial
	DMSO	+2.0	–	–	axial

[a] All values are given in kcal mol^−1^. The experimental observation is based on ^3^ *J*(^19^F,^1^H) values. See the Supporting Information for more details.

These results suggest that C−F bonds, although often considered to be a bioisostere of C−H bonds,^2^ can significantly alter the conformational behavior of fluorinated heterocycles such as piperidines. To illustrate how this concept could potentially be applied in the context of molecular design, we investigated the behavior of 4‐methylpiperidine (**16**) and its fluorinated analogue *cis*‐3,5‐difluoro‐*trans*‐4‐methylpiperidine (**17**) computationally (Scheme 4).

**Scheme 4:**
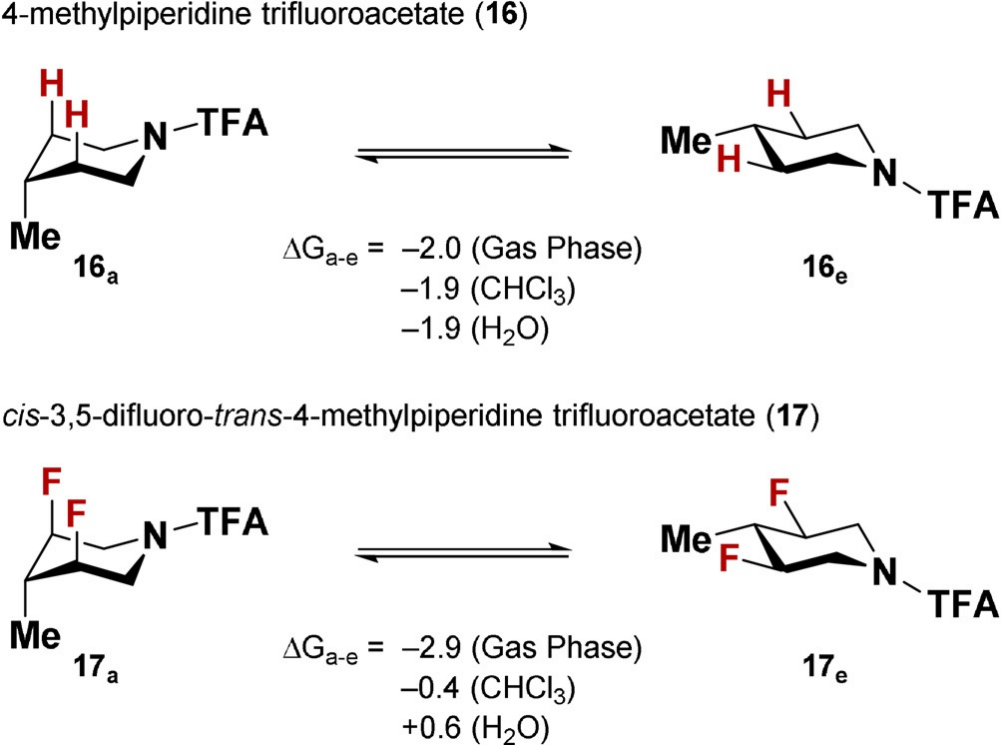
Tuning the conformational behavior of 4‐methylpiperidine analogues by fluorine substitutions. All free enthalpy values are given in kcal mol^−1^.

As expected for the sterically demanding methyl group, the Me_equatorial_ conformer is preferred in both compounds, both in the gas phase and in chloroform (Scheme 4). By switching to more polar solvents such as water, the conformational equilibrium can be significantly shifted. Whilst compound **16** retains its Me_equatorial_ conformer in polar solution, the fluorine atoms in **17** induce a conformational inversion, directing the methyl group into the sterically hindered axial position—showcasing how fluorine substitution can be utilized to manipulate the conformational behavior of polar molecules.^12^

In conclusion, the conformational behavior of fluorinated piperidines is influenced by the interplay of different forces such as electrostatic interactions, hyperconjugation and steric factors. In this Communication we provide, for the first time, a detailed and systematic overview of the major parameters that can control the conformational behavior of fluorinated piperidine derivatives while covering a wide range of substitution patterns on the piperidine ring. The fluorinated piperidines were analyzed experimentally (through NMR studies) and computationally (through DFT computations). Interestingly, in addition to the common forces that contribute to the stabilization of a specific conformer, we realized that the dipole moment can be used to further manipulate the orientation of the fluorine atoms, particularly in polar solutions. These forces may eventually be used to fine‐tune the conformational structure of lead compounds which can dramatically affect their likelihood of success in therapeutic applications.

## Conflict of interest

The authors declare no conflict of interest.

## Supporting information

As a service to our authors and readers, this journal provides supporting information supplied by the authors. Such materials are peer reviewed and may be re‐organized for online delivery, but are not copy‐edited or typeset. Technical support issues arising from supporting information (other than missing files) should be addressed to the authors.

Supplementary

## References

[1a] K. Müller , C. Faeh , F. Diederich , Science 2007, 317, 1881–1886; 17901324 10.1126/science.1131943

[1b] M. Hird , Chem. Soc. Rev. 2007, 36, 2070–2095; 17982522 10.1039/b610738a

[1c] S. Purser , P. R. Moore , S. Swallow , V. Gouverneur , Chem. Soc. Rev. 2008, 37, 320–330; 18197348 10.1039/b610213c

[1d] P. T. Lowe , D. O'Hagan , J. Fluor. Chem. 2020, 230, 109420.

[2a] P. Shah , A. D. Westwell , J. Enzym. Inhib. Med. Chem. 2007, 22, 527–540; 10.1080/14756360701425014 18035820

[2b] D. O′Hagan , Chem. Soc. Rev. 2008, 37, 308–319; 18197347 10.1039/b711844a

[2c] J. Wang , M. Sánchez-Roselló , J. L. Aceña , C. del Pozo , A. E. Sorochinsky , S. Fustero , V. A. Soloshonok , H. Liu , Chem. Rev. 2014, 114, 2432–2506; 24299176 10.1021/cr4002879

[2d] E. P. Gillis , K. J. Eastman , M. D. Hill , D. J. Donnelly , N. A. Meanwell , J. Med. Chem. 2015, 58, 8315–8359. 26200936 10.1021/acs.jmedchem.5b00258

[3a] A. J. Durie , A. M. Z. Slawin , T. Lebl , P. Kirsch , D. O′Hagan , Chem. Commun. 2011, 47, 8265–8267; 10.1039/c1cc13016a 21709894

[3b] A. J. Durie , A. M. Z. Slawin , T. Lebl , P. Kirsch , D. O′Hagan , Chem. Commun. 2012, 48, 9643–9645; 10.1039/c2cc34679f 22911247

[3c] N. S. Keddie , A. M. Z. Slawin , T. Lebl , D. Philp , D. O′Hagan , Nat. Chem. 2015, 7, 483–488; 25991526 10.1038/nchem.2232

[3d] Z. Fang , N. Al-Maharik , A. M. Z. Slawin , M. Bühl , D. O′Hagan , Chem. Commun. 2016, 52, 5116–5119; 10.1039/c6cc01348a 26996764

[3e] N. Aiguabella , M. C. Holland , R. Gilmour , Org. Biomol. Chem. 2016, 14, 5534–5538; 26880180 10.1039/c6ob00025h

[3f] I. G. Molnár , R. Gilmour , J. Am. Chem. Soc. 2016, 138, 5004–5007; 26978593 10.1021/jacs.6b01183

[3g] M. P. Wiesenfeldt , Z. Nairoukh , W. Li , F. Glorius , Science 2017, 357, 908–912; 28798044 10.1126/science.aao0270

[3h] C. J. Thomson , Q. Zhang , N. Al-Maharik , M. Bühl , D. B. Cordes , A. M. Z. Slawin , D. O'Hagan , Chem. Commun. 2018, 54, 8415–8418; 10.1039/c8cc04964e 29999054

[3i] F. Scheidt , M. Schäfer , J. C. Sarie , C. G. Daniliuc , J. J. Molloy , R. Gilmour , Angew. Chem. Int. Ed. 2018, 57, 16431–16435; 10.1002/anie.201810328 30255972

[3j] A. Sadurní , R. Gilmour , Eur. J. Org. Chem. 2018, 3684–3687; 10.1002/ejoc.201800618 PMC6099233 30147438

[3k] Z. Fang , D. B. Cordes , A. M. Z. Slawin , D. O′Hagan , Chem. Commun. 2019, 55, 10539–10542. 10.1039/c9cc05749h 31414105

[4a] D. O′Hagan , Nat. Prod. Rep. 2000, 17, 435–446; 11072891 10.1039/a707613d

[4b] E. Vitaku , D. T. Smith , J. T. Njardarson , J. Med. Chem. 2014, 57, 10257–10274. 25255204 10.1021/jm501100b

[5] The strong axial preference in 3-fluoropiperidinium cation and analogues in the gas phase was dictated by electrostatic interactions, which can be both charge dipole (C−F⋅⋅⋅HN^+^) and hydrogen bond (F⋅⋅⋅H(N^+^)) interactions. In aqueous solution, charge dipole interactions were strongly attenuated and hyperconjugation was calculated to be at least competitive with Lewis-type interactions. For more information, see ref. [6].

[6a] D. C. Lankin , N. S. Chandrakumar , S. N. Rao , D. P. Spangler , J. P. Snyder , J. Am. Chem. Soc. 1993, 115, 3356–3357;

[6b] J. P. Snyder , N. S. Chandrakumar , H. Sato , D. C. Lankin , J. Am. Chem. Soc. 2000, 122, 544–545;

[6c] A. Sun , D. C. Lankin , K. Hardcastle , J. P. Snyder , Chem. Eur. J. 2005, 11, 1579–1591; 15662680 10.1002/chem.200400835

[6d] J. M. Silla , W. G. D. P. Silva , R. A. Cormanich , R. Rittner , C. F. Tormena , M. P. Freitas , J. Phys. Chem. A 2014, 118, 503–507. 24377652 10.1021/jp410458w

[7a] N. E. J. Gooseman , D. O'Hagan , M. J. G. Peach , A. M. Z. Slawin , D. J. Tozer , R. J. Young , Angew. Chem. Int. Ed. 2007, 46, 5904–5908; 10.1002/anie.200700714 17610229

[7b] C. Thiehoff , L. Schifferer , C. G. Daniliuc , N. Santschi , R. Gilmour , J. Fluor. Chem. 2016, 182, 121–126;

[7c] I. G. Molnár , M. C. Holland , C. G. Daniliuc , K. N. Houk , R. Gilmour , Synlett 2016, 27, 1051–1055;

[7d] N. Santschi , C. Thiehoff , M. C. Holland , C. G. Daniliuc , K. N. Houk , R. Gilmour , Organometallics 2016, 35, 3040–3044;

[7e] F. Scheidt , P. Selter , N. Santschi , M. C. Holland , D. V. Dudenko , C. G. Daniliuc , C. Mück-Lichtenfeld , M. R. Hansen , R. Gilmour , Chem. Eur. J. 2017, 23, 6142–6149; 27788283 10.1002/chem.201604632

[7f] C. Thiehoff , Y. P. Rey , R. Gilmour , Isr. J. Chem. 2017, 57, 92–100;

[7g] R. A. Cormanich , D. O'Hagan , M. Bühl , Angew. Chem. Int. Ed. 2017, 56, 7867–7870; 10.1002/anie.201704112 28561937

[7h] M. Aufiero , R. Gilmour , Acc. Chem. Res. 2018, 51, 1701–1710; 29894155 10.1021/acs.accounts.8b00192

[7i] C. S. Teschers , C. G. Daniliuc , G. Kehr , R. Gilmour , J. Fluorine Chem. 2018, 210, 1–5.

[8] M. P. Freitas , Org. Biomol. Chem. 2013, 11, 2885–2890. 23515623 10.1039/c3ob40187a

[9] Z. Nairoukh , M. Wollenburg , C. Schlepphorst , K. Bergander , F. Glorius , Nat. Chem. 2019, 11, 264–270. 30664720 10.1038/s41557-018-0197-2 PMC6522351

[10] It should be noted that the vicinal ^3^ *J*(^19^F,^1^H_a_) coupling constants provide useful insight into the conformational structure, since large values of ^3^ *J*(^19^F,^1^H_a_) indicate axial preference and small values of ^3^ *J*(F,H_a_) indicate equatorial preference (for more details, see Supporting Information, Section 4, ref. [9]).

[11a] R. J. Abraham , L. Cavalli , K. G. R. Pachler , Mol. Phys. 1966, 11, 471–494;

[11b] R. J. Abraham , M. A. Cooper , J. Chem. Soc., Chem. Commun. 1966, 588–589;

[11c] R. J. Abraham , J. Phys. Chem. 1969, 73, 1192–1199;

[11d] R. J. Abraham , G. Gatti , J. Chem. Soc. B 1969, 961–968;

[11e] R. J. Abraham , K. Parry , J. Chem. Soc. B 1970, 539–545;

[11f] L. Cavalli , R. J. Abraham , Mol. Phys. 1970, 19, 265–274;

[11g] R. J. Abraham , R. H. Kemp , J. Chem. Soc. B 1971, 1240–1245.

[12] Examples on chameleon molecules:

[12a] P. A. Carrupt , B. Testa , A. Bechalany , N. El Tayar , P. Descas , D. Perrissoud , J. Med. Chem. 1991, 34, 1272–1275; 2016703 10.1021/jm00108a005

[12b] N. El Tayar , A. E. Mark , P. Vallat , R. M. Brunne , B. Testa , W. F. van Gunsteren , J. Med. Chem. 1993, 36, 3757–3764; 8254605 10.1021/jm00076a002

[12c] L. Fielding , J. K. Clark , R. McGuire , J. Org. Chem. 1996, 61, 5978–5981;

[12d] S. J. Fox , S. Gourdain , A. Coulthurst , C. Fox , I. Kuprov , J. W. Essex , C.-K. Skylaris , B. Linclau , Chem. Eur. J. 2015, 21, 1682–1691. 25418601 10.1002/chem.201405317

