# Supplementary material for: Understanding the Conformational Behavior of Fluorinated Piperidines: The Origin of the Axial‐F Preference
Source: Chemistry. 2020 May 12;26(28):6141–6. doi: 10.1002/chem.202001355 (PMC7317880; doi:10.1002/chem.202001355)

Chemistry–A European Journal

Supporting Information

Understanding the Conformational Behavior of Fluorinated Piperidines: The Origin of the Axial-F Preference

Zackaria Nairoukh+,* Felix Strieth-Kalthoff+, Klaus Bergander, and Frank Glorius*[a]

Understanding the conformational behavior of fluorinated piperidines: The origin of the axial-F orientation

Zackaria Nairoukh*, Felix Strieth-Kalthoff, Klaus Bergander, Frank Glorius*
Correspondence to: glorius@uni-muenster.de; z.nairoukh@mail.huji.ac.il

Supplementary Information

Table of Contents

Materials and Methods	3
Preparation of Fluoropiperidine Derivatives	5
NMR and Computational Analysis	8
Synthesis of All-*cis*-(multi)fluorinated Piperidines	27
References	66
Computational Data	67
Geometries	76
NMR Spectra	118

Materials and Methods

All trifluoroacetyl-(TFA) and *tert*-butyloxycarbonyl-(Boc)-fluoropiperidine derivatives were prepared according to previously published procedures.[1] The preparation of acetyl-(Ac)- and pivaloyl-(Piv)-fluoropiperidine derivatives as well as the preparation of fluoropiperidinium hydrochloride salts and non-protonated fluoropiperidine derivatives is described in the following section.

^{1}H and ^{13}C, and ^{19}F NMR spectra were recorded on a Bruker Avance II 300 or Avance II 400, Agilent DD2 500 or Agilent DD2 600 in the indicated solvents. Chemical shifts (δ) are given in ppm relative to TMS. The residual solvent signals were used as references and the chemical shifts converted to the TMS scale ($CDCl_3$: δ_H = 7.26 ppm, δ_C = 77.16 ppm; CD_2Cl_2: δ_H = 5.32 ppm, δ_C = 54.0 ppm; C_6D_6: δ_H = 7.16 ppm, δ_C = 128.06 ppm; D_2O: δ_H = 4.79 ppm; DMSO-d_6: δ_H = 2.50 ppm, δ_C = 39.5 ppm). ^{19}F NMR spectra are referenced according to the proton resonance of TMS as the primary reference for the unified chemical shift scale (IUPAC recommendation 2001). ESI mass spectra were recorded on a Bruker Daltonics MicroTof spectrometer.

Typical NMR sample contains 20 mg of the measured compounds. For literature known compounds, only ^{1}H NMR spectra in different deuterated solvents are presented. For newly synthesized compounds, fully detailed NMR spectra and ESI-MS are given.

The experimental determination of the axial or equatorial orientation of the fluorine atoms on the piperidine ring was done through NMR studies. It has been shown that the vicinal $^{3}J(^{19}F,^{1}H)$ coupling constants provide useful insight into conformational structure, as large values of $^{3}J(^{19}F,^{1}H_a)$ indicate axial preference and small values of $^{3}J(^{19}F,^{1}H_a)$ indicate equatorial preference.[1] For determining the $^{3}J(^{19}F,^{1}H_a)$ we applied selective, multi-selective and band-selective decoupling schemes. This was done using the Pbox provided by Vnmrj of Agilent.

Computations were carried out using the ORCA 4.1.1 software package, as developed by Neese and co-workers.[2] All structures are given in the .xyz format and were visualized using *Jmol*.[3] Gas phase geometries of the respective conformers were optimized using the M06-2X hybrid functional[4] in Ahlrich's def2-TZVPP basis set.[5] All conformers were confirmed to be local minima on the respective potential energy surface by the absence of negative eigenvalues of the Hessian after numerical harmonic frequency analysis at the same level of computation. No symmetry of internal coordinate constraints were applied. Higher energy conformers (in particular, rotamers of the respective acetyl-, pivaloyl- or carbamoyl-protected amines) were taken into account. The

electronic energy of the previously optimized geometries was then determined by an additional single-point calculation on the M06-2X / def2-QZVPP level.[4, 5] The reported free energy values (in E_h and kcal mol^{-1}) were then obtained as the sum of the single point electronic energies and the respective free energy corrections (ZPVE, thermal corrections, enthalpy correction, entropy correction), as obtained from the harmonic frequency analysis. Geometries and free energies for the conformers in solution were obtained accordingly. For both geometry optimization and single point calculation, solvation effects were included using the CPCM continuum solvation model (presets for benzene, dichloromethane, chloroform, dimethyl sulfoxide and water, respectively).[6] NBO analysis was performed using the NBO 6.0 software.[7] The difference in electronic energies between two conformers was attributed to a sum of hyperconjugative, steric and electrostatic contributions.

$$\Delta E_{el} = \Delta E_{\text{hyperconj}} + \underbrace{\Delta E_{\text{steric}} + \Delta E_{\text{electrostat}}}_{\Delta E_{\text{loc}}}$$

Hyperconjugative stabilization was quantified by subtracting the actual electronic energy from the energy of a hypothetical fully localized structure E_{loc}, deleting all antibonding orbitals from the electronic energy computation ($DEL LEWIS keyword). Steric contributions were obtained using the NBO/NLMO steric analysis ($NBO STERIC keyword). The residual difference in electronic energies was attributed to electrostatic interactions, neglecting potential coupling between steric and electrostatic interactions, and potential non-Lewis electrostatic interactions. Individual hyperconjugative interactions were extracted from the result of a second order perturbation theory analysis of the Fock matrix within the basis of NBOs.

Preparation of Fluoropiperidine Derivatives

Preparation of fluoropiperidinium hydrochloride salts (**1B–12B**).

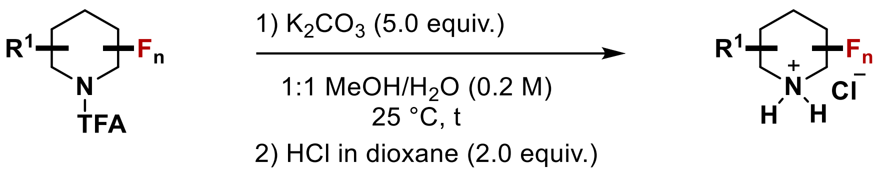

Following a literature procedure,[1] K_2CO_3 (5.0 equiv) was added in one portion to a solution of TFA-fluoropiperidine derivative (**1A–12A**) (1.0 equiv.) in 1:1 methanol/water (0.2 M) at room temperature. After stirring the solution for the indicated time, the reaction mixture was acidified with 2 M aqueous solution of HCl. After removal of all volatiles, 2 M aqueous solution of NaOH and CH_2Cl_2 (5 mL) were added. The organic layer was separated and the aqueous layer was further extracted with CH_2Cl_2 (3×5 mL). The organic layers were combined, dried over $MgSO_4$ and filtrated. HCl (4 M in 1,4-dioxane, 2.0 equiv.) was added to the filtrate and all volatiles were removed to yield the corresponding fluoropiperidinium hydrochloride (**1B–12B**) as a white solid. NMR samples were prepared by dissolving the fluoropiperidinium adducts in D_2O.

Preparation of non-protonated fluoropiperidine derivatives (**1C–12C**).

General procedure A

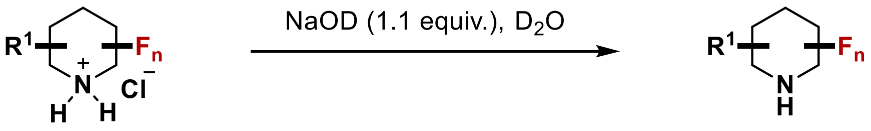

To a solution of fluoropiperidinium hydrochloride salt (**1B–12B**, 1.0 equiv.) in D_2O (1 mL) was added NaOD (1.1 equiv.) at room temperature. The mixture was then transferred to NMR tube and submitted for measurements.

General procedure B

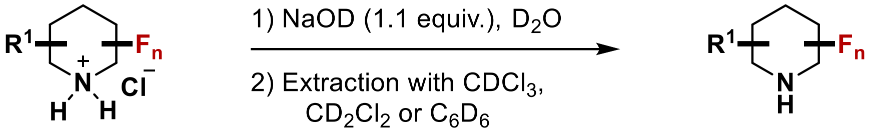

To a solution of fluoropiperidinium hydrochloride salt (**1B**–**12B**, 1.0 equiv.) in D_2O (1 mL) was added NaOD (1.1 equiv.) at room temperature. The aqueous solution was then extracted with a deuterated solvent ($CDCl_3$, CD_2Cl_2 or C_6D_6), dried over $MgSO_4$ and transferred to NMR tube for the required measurements.

Preparation of 1-(cis-3,5-difluoropiperidin-1-yl)ethan-1-one (**13**).

To a solution of *cis*-3,5-difluoropiperidine hydrochloride **2B** (0.157 g, 1.0 mmol, 1.0 equiv.) in H_2O (1 mL) was added 1M NaOH (1.1 mL, 1.1 mmol, 1.1 equiv.) at room temperature. The aqueous solution was then extracted with CH_2Cl_2 (3×5 mL), dried over $MgSO_4$ and filtrated to deliver the non-protonated fluoropiperidine derivative in CH_2Cl_2 solution.

To the prepared solution, acetyl chloride (0.117 g, 1.5 mmol, 1.5 equiv.) and triethylamine (0.42 mL, 3.0 mmol, 3.0 equiv.) were added at room temperature. Upon the completion of the reaction (followed by TLC), water (10 mL) was added. The organic layer was separated and the aqueous layer was further extracted with CH_2Cl_2 (3×5 mL). The organic layers were combined, dried over $MgSO_4$, filtrated and concentrated under reduced pressure. The crude was then submitted to column chromatography (0-100% ethyl acetate in pentane) to obtain the final product as a white solid (0.15 g, 0.92 mmol, 92%).

Preparation of 1-(cis-3,5-difluoropiperidin-1-yl)-2,2-dimethylpropan-1-one (**14**).

To a solution of *cis*-3,5-difluoropiperidine hydrochloride **2B** (0.504 g, 3.2 mmol, 1.0 equiv.) in H_2O (2 mL) was added 1M NaOH (3.5 mL, 3.5 mmol, 1.1 equiv.) at room temperature. The aqueous solution was then extracted with CH_2Cl_2 (3×5 mL), dried over $MgSO_4$ and filtrated to deliver the non-protonated fluoropiperidine derivative in CH_2Cl_2 solution.

To the prepared solution, pivaloyl chloride (1.1 g, 9.6 mmol, 3.0 equiv.) and triethylamine (1.3 mL, 9.6 mmol, 3.0 equiv.) were added at room temperature. Upon the completion of the reaction (followed by TLC), water (10 mL) was added. The organic layer was separated and the

aqueous layer was further extracted with CH_2Cl_2 (3×5 mL). The organic layers were combined, dried over $MgSO_4$, filtrated and concentrated under reduced pressure. The crude was then submitted to column chromatography (0-10% ethyl acetate in pentane) to obtain the final product as a white solid (0.612 g, 2.9 mmol, 90%).

2,2,2-Trifluoro-1-(3-fluoropiperidin-1-yl)ethan-1-one (1A)

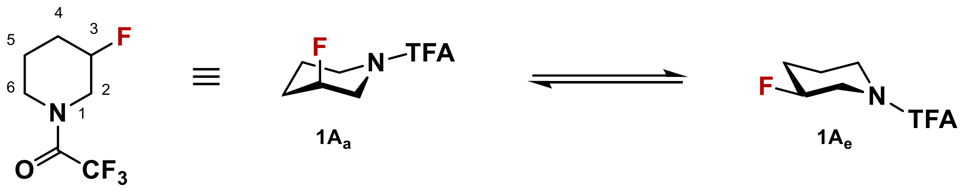

This compound was prepared following a recent literature procedure.[1]

Experimental observation

The product was present as a ~1:1 mixture of amide bond rotamers.

In **Toluene-*d*$_8$**: The orientation of the fluorine atom was assigned as axial due to the large value of ^3J(F,H$_a$).[1] [**Rot A: ^3J(2-H$_a$,3-F) = 26.0 Hz, Rot B: ^3J(2-H$_a$,3-F) = 26.0 Hz**]

Computational observation

The calculated ΔG is given for the energetically lowest conformers.

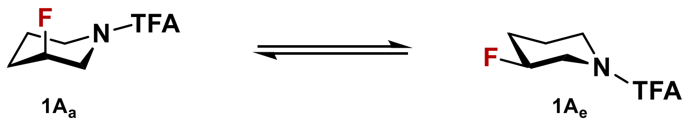

Solvent	ΔG(a→e)	ΔE_{elect}	ΔE_{hyperc}	ΔE_{steric}	μ_a	μ_e	Experimental
**Toluene**	+0.1	+6.5	+3.3	–9.6	6.8	4.3	axial
**gas phase**	–0.4	+9.4	+2.4	–12.1	5.6	3.6	-

All values are given in kcal/mol at 298 K.

3-Fluoropiperidine hydrochloride (1B)

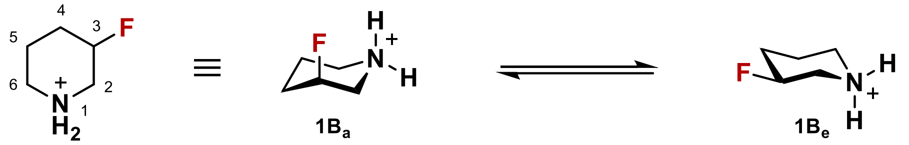

This compound was prepared following a recent literature procedure.[1]

<u>Experimental observation</u>

In **D_2O**: The orientation of the fluorine atom was assigned as axial due to the large value of ^{3}J(F,H_a).[1] [^{3}***J*****(2-H_a,3-F) = 38.7 Hz**]

<u>Computational observation</u>

The calculated ΔG is given for the energetically lowest conformers.

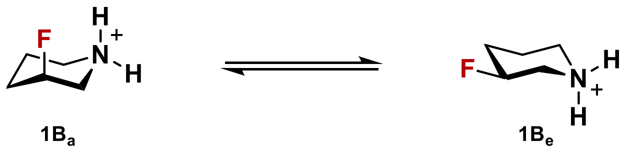

Solvent	ΔG(a→e)	ΔE_{elect}	ΔE_{hyperc}	ΔE_{steric}	μ_a	μ_e	Experimental
**H_2O**	+1.8	+12.6	+3.8	–14.4	6.9	9.0	axial
**gas phase**	+4.8	+22.1	–0.1	–16.8	5.0	7.0	-

All values are given in kcal/mol at 298 K.

3-Fluoropiperidine (1C)

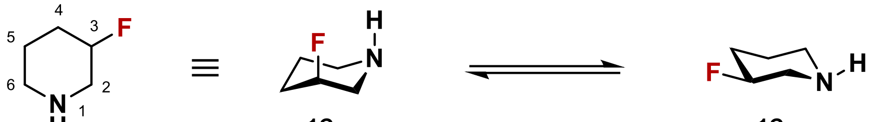

The preparation of this compound is described in the previous section.

Experimental observation

In **D_2O**: Due to peaks overlapping in NMR spectra, we could not verify the orientation of the fluorine atom through $^3J(F,H_a)$. Therefore we conducted additional NMR studies that includes $^1H\{^{19}F\}$ NMR with homo-decoupling of proton at two different positions 4-H and 2-H (separately). From these experiments, we found that the vicinal coupling constants $^3J(3\text{-}H_e,2\text{-}H_a)$, $^3J(3\text{-}H_e,2\text{-}H_{eq})$, $^3J(3\text{-}H_e,4\text{-}H_a)$ and $^3J(3\text{-}H_e,4\text{-}H_e)$ are of small values (~4.4 Hz). In addition, we could not observe any axial-axial interaction between 3-H and 4-H/2-H. Accordingly, we believe that the axial orientation of the fluorine atom in this case is dominant.

^{1}H NMR (500 MHz, D_2O, 299 K) δ 4.79 (d, J = 47.3 Hz, 1H overlaps with solvent), 3.04 – 2.91 (m, 2H), 2.89 – 2.81 (m, 1H), 2.78 – 2.69 (m, 1H), 1.97 – 1.75 (m, 3H), 1.61 – 1.52 (m, 1H); **^{1}H{^{19}F} NMR** (500 MHz, D_2O, 299 K) δ 4.79 (s, 1H overlaps with solvent), 3.07 – 2.92 (m, 2H), 2.90 – 2.81 (m, 1H), 2.78 – 2.69 (m, 1H), 2.00 – 1.76 (m, 3H), 1.60 – 1.51 (m, 1H); **^{13}C NMR** (126 MHz, D_2O, 299 K) δ 88.49 (d, J = 166.3 Hz), 48.24 (d, J = 21.9 Hz), 44.14, 28.58 (d, J = 19.8 Hz), 20.89 (d, J = 3.9 Hz); **^{13}C{sel-^{19}F at -183 ppm} NMR** (126 MHz, D_2O, 299 K) δ 88.49, 48.24, 44.14, 28.58, 20.89; **^{19}F NMR** (470 MHz, D_2O, 299 K) δ -183.33; **^{19}F{^{1}H} NMR** (470 MHz, D_2O, 299 K) δ -183.33.

Computational observation

The calculated ΔG is presented as follows: ΔG in water (ΔG gas phase) and are given for the energetically lowest conformers.

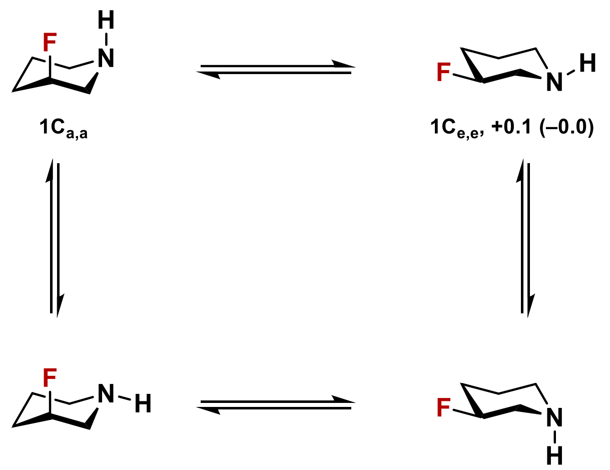

Solvent	ΔG(a,a→e,e)	ΔE_{elect}	ΔE_{hyperc}	ΔE_{steric}	$\mu_{a,a}$	$\mu_{e,e}$	Experimental
**H_2O**	+0.1	+12.7	+5.1	–17.7	2.3	2.9	axial dominant
**gas phase**	–0.0	+6.9	+4.8	–11.7	1.6	2.2	-

All values are given in kcal/mol at 298 K.

1-(*cis*-3,5-Difluoropiperidin-1-yl)-2,2,2-trifluoroethan-1-one (2A)

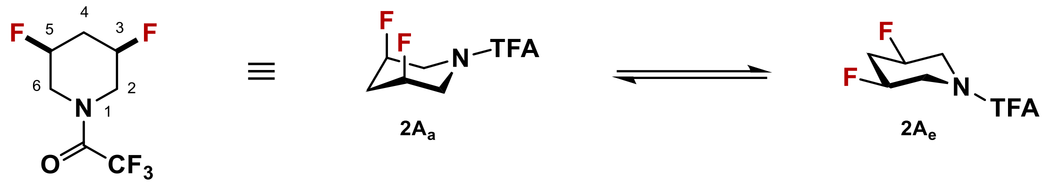

For this particular sample we conducted several computations and NMR experiments in different solvents in order to investigate the effect of solvent's dipole moment on the orientation of the fluorine atoms.

This compound was prepared following a recent literature procedure.[1]

Experimental observation

In **$CDCl_3$**: The orientation of the fluorine atoms was assigned as axial due to the large values of ^3J(F,H_a).[1] [**^3J(2-H_a,3-F) = 32.1 Hz, ^3J(4-H_a,3-F) = 36.1 Hz, ^3J(4-H_a,5-F) = 36.1 Hz, ^3J(6-H_a,5-F) = 30.6 Hz**]

In **DMSO**: The orientation of the fluorine atoms was assigned as axial due to the large values of ^3J(F,H_a). [**^3J(2-H_a,3-F) = 38.6 Hz, ^3J(4-H_a,3-F) = 44.4 Hz, ^3J(4-H_a,5-F) = 44.4 Hz, ^3J(6-H_a,5-F) = 40.1 Hz**]

^{1}H NMR (500 MHz, DMSO-d_6, 299 K) δ 4.95 (dm, *J* = 46.0 Hz, 1H), 4.90 (dm, *J* = 46.0 Hz, 1H), 4.56 (ddq, *J* = 14.8, 12.4, 2.5 Hz, 1H), 4.09 (dddd, *J* = 13.0, 9.3, 4.9, 2.5 Hz, 1H), 3.62 (ddd, *J* = 38.6, 15.1, 1.5 Hz, 1H), 3.23 (dd, *J* = 40.1, 14.5 Hz, 1H), 2.30 (dtt, *J* = 17.3, 12.4, 2.5 Hz, 1H), 2.10 (tdt, *J* = 44.4, 16.2, 3.3 Hz, 1H); **^{1}H{^{19}F} NMR** (500 MHz, DMSO-d_6, 299 K) δ 4.96 (bs, 1H), 4.91 (bs, 1H), 4.56 (dq, *J* = 14.6, 2.5 Hz, 1H), 4.09 (dm, *J* = 15.1 Hz, 1H), 3.62 (d, *J* = 15.0 Hz, 1H), 3.23 (d, *J* = 14.5 Hz, 1H), 2.30 (dp, *J* = 16.2, 2.5 Hz, 1H), 2.10 (dt, *J* = 16.2, 3.3 Hz, 1H).

In **CD_2Cl_2**: The orientation of the fluorine atoms was assigned as axial due to the large values of ^3J(F,H_a). [**^3J(2-H_a,3-F) = 32.9 Hz, ^3J(4-H_a,3-F) = 38.8 Hz, ^3J(4-H_a,5-F) = 38.8 Hz, ^3J(6-H_a,5-F) = 34.6 Hz**]

^{1}H NMR (500 MHz, CD_2Cl_2, 299 K) δ 4.86 (dm, *J* = 45.1 Hz, 1H), 4.79 (dm, *J* = 45.7 Hz, 1H), 4.66 – 4.58 (m, 1H), 4.21 – 4.12 (m, 1H), 3.46 (dd, *J* = 32.9, 14.9 Hz, 1H), 3.18 (ddm, *J* = 34.6,

14.5 Hz, 1H), 2.52 – 2.38 (m, 1H), 2.03 (tdm, *J* = 38.8, 15.8 Hz, 1H); **^{1}H{^{19}F} NMR** (500 MHz, CD_2Cl_2, 299 K) δ 4.89 – 4.85 (m, 1H), 4.83 – 4.76 (m, 1H), 4.62 (dm, *J* = 14.5 Hz, 1H), 4.16 (dm, *J* = 14.9 Hz, 1H), 3.46 (d, *J* = 15.0 Hz, 1H), 3.17 (dd, *J* = 14.5, 3.6 Hz, 1H), 2.46 (dm, *J* = 15.8 Hz, 1H), 2.02 (dm, *J* = 15.8 Hz, 1H).

In **C_6D_6**: The orientation of the fluorine atoms was assigned as axial due to the large values of ^3J(F,H_a). [**^3J(2-H_a,3-F) = 29.6 Hz, ^3J(4-H_a,3-F) = 34.1 Hz, ^3J(4-H_a,5-F) = 34.1 Hz, ^3J(6-H_a,5-F) = 30.6 Hz**]

^{1}H NMR (500 MHz, C_6D_6, 299 K) δ 4.03 – 3.95 (m, 1H), 3.82 (dm, *J* = 45.0 Hz, 1H overlaps with 5-H_e), 3.74 (dm, *J* = 45.7 Hz, 1H overlaps with 3-H_e), 3.45 – 3.35 (m, 1H), 2.46 (dm, *J* = 29.6 Hz, 1H overlaps with 6-H_a), 2.40 (dm, *J* = 30.6 Hz, 1H overlaps with 2-H_a), 1.75 – 1.63 (m, 1H), 0.90 (tdt, *J* = 34.1, 15.1, 3.7 Hz, 1H); **^{1}H{^{19}F} NMR** (500 MHz, C_6D_6, 299 K) δ 3.99 (dm, *J* = 14.2 Hz, 1H), 3.85 – 3.81 (m, 1H), 3.78 – 3.73 (m, 1H), 3.40 (dm *J* = 14.7 Hz, 1H), 2.47 (dd, *J* = 14.7, 2.4 Hz, 1H), 2.40 (dd, *J* = 14.2, 2.4 Hz, 1H), 1.69 (dtt, *J* = 15.1, 4.5, 1.7 Hz, 1H), 0.90 (dt, *J* = 15.1, 3.7 Hz, 1H).

Computational observation

The calculated ΔG is given for the energetically lowest conformers.

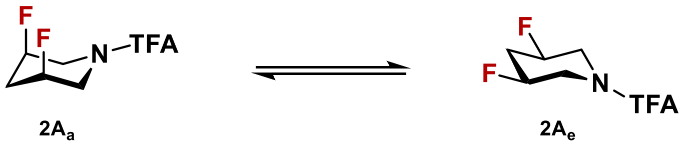

Solvent	ΔG(a→e)	ΔE_{elect}	ΔE_{hyperc}	ΔE_{steric}	μ_a	μ_e	Experimental
**DMSO**	+2.0	+10.6	+12.1	–20.8	9.8	3.3	axial
**CH_2Cl_2**	+1.4	+8.5	+11.9	–19.0	9.3	3.2	axial
**$CHCl_3$**	+0.9	+8.1	+11.7	–18.8	8.9	3.0	axial
**C_6H_6**	+0.1	+12.4	+11.4	–23.7	8.1	2.7	axial
**gas phase**	–1.4	+12.5	+11.0	–24.8	6.6	2.2	-

All values are given in kcal/mol at 298 K.

cis-3,5-Difluoropiperidine hydrochloride (2B)

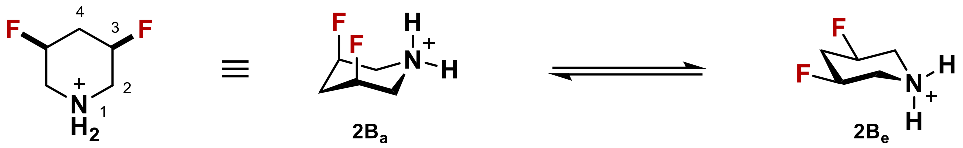

This compound was prepared following a recent literature procedure.[1]

Experimental observation

In **D_2O**: The orientation of the fluorine atoms was assigned as axial due to the large values of ^{3}J(F,H$_a$).[1] [**^{3}J(2-H$_a$,3-F) = 39.5 Hz, ^{3}J(4-H$_a$,3-F) = 46.7 Hz**]

Computational observation

The calculated ΔG is given for the energetically lowest conformers.

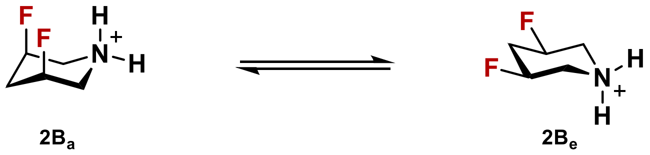

Solvent	ΔG(a→e)	ΔE_{elect}	ΔE_{hyperc}	ΔE_{steric}	μ_a	μ_e	Experimental
**H_2O**	+3.9	+14.7	+11.0	–21.1	9.1	10.1	axial
**gas phase**	+8.6	+33.9	+5.4	–30.3	6.8	7.8	-

All values are given in kcal/mol at 298 K.

***cis*-3,5-Difluoropiperidine (2C)**

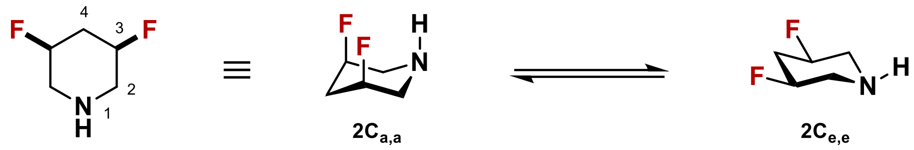

For this particular sample we conducted NMR experiments in different solvents in order to investigate the effect of solvent's dipole moment on the orientation of the fluorine atoms.

The preparation of this compound is described in the previous section.

Experimental observation

In **D_2O**: The orientation of the fluorine atoms was assigned as axial due to the large values of ^3J(F,H_a). [**^3J(2-H_a,3-F) = 40.2 Hz, ^3J(4-H_a,3-F) = 44.2 Hz**]

^{1}H NMR (500 MHz, D_2O, 299 K) δ 4.81 (dm, J = 46.6 Hz, 2H overlap with solvent), 3.23 – 3.13 (m, 2H), 2.85 (dd, J = 40.2, 14.6 Hz, 2H), 2.47 – 2.35 (m, 1H), 2.10 (tdt, J = 44.2, 16.1, 3.3 Hz, 1H); **^{1}H{^{19}F} NMR** (500 MHz, D_2O, 299 K) δ 4.85 – 4.81 (m, 2H), 3.18 (dm, J = 14.9 Hz, 2H), 2.86 (d, J = 14.8 Hz, 2H), 2.40 (dm, J = 16.1 Hz, 1H), 2.10 (dt, J = 16.8, 3.3 Hz, 1H); **^{13}C NMR** (126 MHz, D_2O, 299 K) δ 86.68 (d, J = 168.3 Hz), 47.35 – 47.03 (m), 32.20 (t, J = 20.1 Hz); **^{13}C{sel-^{19}F at -181 ppm} NMR** (126 MHz, D_2O, 299 K) δ 86.68, 47.19, 32.20; **^{19}F NMR** (470 MHz, D_2O, 299 K) δ -181.73 – -182.13 (m); **^{19}F{^{1}H} NMR** (470 MHz, D_2O, 299 K) δ -181.94.

In **DMSO**: The orientation of the fluorine atoms was assigned as axial due to the large value of ^3J(F,H_a). [**^3J(2-H_a,3-F) = 31.9 Hz**]

It should be noted that the NMR sample contained small amount of water that were needed for the preparation of the non-protonated piperidine.

^{1}H NMR (600 MHz, DMSO-d_6, 299 K) δ 4.57 (dm, J = 47.1 Hz, 2H), 2.81 (ddt, J = 13.6, 11.8, 2.6 Hz, 2H), 2.72 (dd, J = 31.9, 14.2 Hz, 2H), 2.14 – 1.93 (m, 2H); **^{1}H{^{19}F} NMR** (600 MHz, DMSO-d_6, 299 K) δ 4.60 – 4.52 (m, 2H), 2.82 (dd, J = 14.3, 4.2 Hz, 2H), 2.72 (d, J = 14.4 Hz, 2H), 2.07 – 2.00 (m, 2H).

In **$CDCl_3$**: The orientation of the fluorine atoms was assigned as axial due to the large values of ^3J(F,H$_a$). [$\boldsymbol{^3J}$**(2-H$_a$,3-F) = 30.1 Hz,** $\boldsymbol{^3J}$**(4-H$_a$,3-F) = 34.5 Hz**]

^{1}H NMR (600 MHz, CD_2Cl_2, 299 K) δ 4.57 (dm, *J* = 46.0 Hz, 2H), 3.14 – 3.05 (m, 2H), 2.91 (ddd, *J* = 30.1, 14.3, 2.2 Hz, 2H), 2.35 – 2.23 (m, 1H), 2.06 (tdt, *J* = 34.5, 14.9, 3.6 Hz, 1H); **^{1}H{^{19}F} NMR** (600 MHz, CD_2Cl_2, 299 K) δ 4.62 – 4.55 (m, 2H), 3.09 (ddd, *J* = 14.3, 4.4, 1.9 Hz, 2H), 2.91 (d, *J* = 14.3 Hz, 2H), 2.29 (dm, *J* = 14.8 Hz, 1H), 2.06 (dt, *J* = 14.9, 3.6 Hz, 1H).

In **CD_2Cl_2**: The orientation of the fluorine atoms was assigned as axial due to the large values of ^3J(F,H$_a$). [$\boldsymbol{^3J}$**(2-H$_a$,3-F) = 28.5 Hz,** $\boldsymbol{^3J}$**(4-H$_a$,3-F) = 33.5 Hz**]

^{1}H NMR (600 MHz, CD_2Cl_2, 299 K) δ 4.53 (dm, *J* = 47.2 Hz, 2H), 3.03 – 2.96 (m, 2H), 2.89 (dm, *J* = 28.5 Hz, 2H), 2.25 – 2.15 (m, 1H), 2.07 (tdt, *J* = 33.5, 14.6, 3.6 Hz, 1H); **^{1}H{^{19}F} NMR** (600 MHz, CD_2Cl_2, 299 K) δ 4.56 – 4.50 (m, 2H), 3.00 (ddd, *J* = 14.1, 4.8, 1.8 Hz, 2H), 2.90 (dd, *J* = 14.2, 2.5 Hz, 2H), 2.19 (dtt, *J* = 14.7, 5.1, 1.9 Hz, 1H), 2.08 (dt, *J* = 14.6, 3.6 Hz, 1H).

In **C_6D_6**: The dominant orientation of the fluorine atoms is assigned to be axial based on the values of ^3J(F,H$_a$). [$\boldsymbol{^3J}$**(2-H$_a$,3-F) = 21.2 Hz,** $\boldsymbol{^3J}$**(4-H$_a$,3-F) = 25.5 Hz**]

^{1}H NMR (600 MHz, C_6D_6, 299 K) δ 3.92 (dm, *J* = 47.6 Hz, 2H), 2.54 – 2.48 (m, 2H), 2.43 (dd, *J* = 21.2, 13.3 Hz, 2H), 1.83 – 1.72 (m, 1H), 1.59 (tdt, *J* = 25.5, 14.0, 3.9 Hz, 1H); **^{1}H{^{19}F} NMR** (600 MHz, C_6D_6, 299 K) δ 3.94 – 3.90 (m, 2H), 2.51 (ddd, *J* = 13.4, 6.1, 1.3 Hz, 2H), 2.42 (dd, *J* = 13.3, 3.3 Hz, 2H), 1.81 – 1.74 (m, 1H), 1.59 (dt, *J* = 13.6, 4.0 Hz, 1H).

Computational observation

The calculated ΔG values in different solvents for the energetically lowest conformers are presented. In the scheme, the values of ΔG in water (ΔG gas phase) are given.

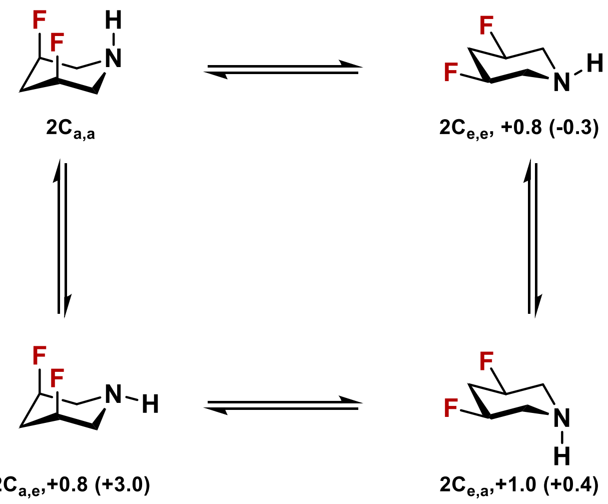

Solvent	ΔG(a,a→e,e)	ΔE_{elect}	ΔE_{hyperc}	ΔE_{steric}	$\mu_{a,a}$	$\mu_{e,e}$	Experimental
**DMSO**	+0.8	+20.8	+10.8	–30.7	4.4	2.8	axial
**H_2O**	+0.8	+20.8	+10.8	–30.7	4.4	2.8	axial
**CH_2Cl_2**	+0.6	+20.8	+10.9	–30.9	4.3	2.8	axial
**$CHCl_3$**	+0.5	+20.5	+11.0	–30.9	4.1	2.7	axial
**C_6H_6**	+0.2	+20.2	+11.1	–31.0	3.7	2.6	axial
**gas phase**	–0.3	+20.6	+11.3	–32.1	3.1	2.3	-

All values are given in kcal/mol at 298 K.

2,2,2-Trifluoro-1-(*cis*-3-fluoro-4-methylpiperidin-1-yl)ethan-1-one (3A)

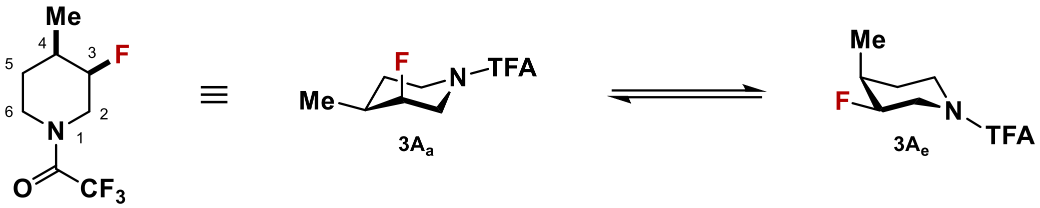

This compound was prepared following a recent literature procedure.[1]

Experimental observation

The product was present as a ~1:1 mixture of amide bond rotamers.

In **$CDCl_3$**: The orientation of the fluorine atom was assigned as axial due to the large value of ^{3}J(F,H_a).[1] [**Rot A: ^{3}J(2-H_a,3-F) = 38.3 Hz, Rot B: ^{3}J(2-H_a,3-F) = 36.6 Hz**]

Computational observation

The calculated ΔG is given for the energetically lowest conformers.

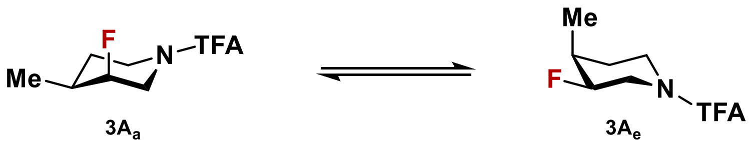

Solvent	ΔG(a→e)	ΔE_{elect}	ΔE_{hyperc}	ΔE_{steric}	μ_a	μ_e	Experimental
**$CHCl_3$**	+2.3	–0.8	+5.0	–2.1	7.5	5.8	axial
**gas phase**	+1.8	+0.5	+4.4	–3.3	5.6	4.4	-

All values are given in kcal/mol at 298 K.

***cis*-3-Fluoro-4-methylpiperidine hydrochloride (3B)**

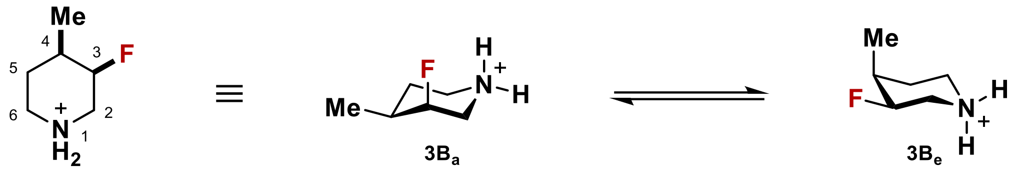

This compound was prepared following a recent literature procedure.[1]

Experimental observation

In **D_2O**: The orientation of the fluorine atoms was assigned as axial due to the large values of ^3J(F,H_a).[1] [**^3J(2-H_a,3-F) = 36.6 Hz, ^3J(4-H_a,3-F) = 36.5 Hz**]

Computational observation

The calculated ΔG is given for the energetically lowest conformers.

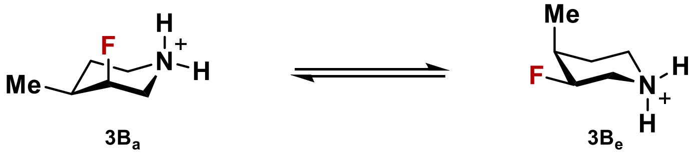

Solvent	ΔG(a→e)	ΔE_{elect}	ΔE_{hyperc}	ΔE_{steric}	μ_a	μ_e	Experimental
**H_2O**	+3.6	+8.5	+3.0	–8.0	8.3	9.7	axial
**gas phase**	+6.2	+16.3	–0.8	–9.3	6.0	7.4	-

All values are given in kcal/mol at 298 K.

***cis*-3-Fluoro-4-methylpiperidine (3C)**

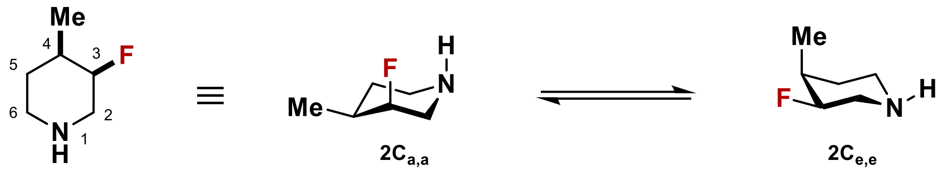

The preparation of this compound is described in the previous section.

<u>Experimental observation</u>

In **D_2O**: The orientation of the fluorine atom was assigned as axial due to the large values of ^3J(F,H_a). [**^3J(2-H_a,3-F) = 42.3 Hz, ^3J(4-H_a,3-F) = 37.3 Hz**]

^{1}H NMR (600 MHz, D_2O, 299 K) δ 4.56 (d, J = 48.3 Hz, 1H), 3.12 (t, J = 13.0 Hz, 1H), 2.91 (dm, J = 12.9 Hz, 1H), 2.65 (dd, J = 42.3, 14.3 Hz, 1H), 2.50 (tdd, J = 12.5, 3.3, 1.4 Hz, 1H), 1.70 (dm, J = 37.3 Hz, 1H), 1.50 – 1.34 (m, 2H), 0.96 (d, J = 6.9 Hz, 3H); **^{1}H{^{19}F} NMR** (600 MHz, D_2O, 299 K) δ 4.56 (bs, 1H), 3.12 (d, J = 13.8 Hz, 1H), 2.91 (d, J = 12.9 Hz, 1H), 2.65 (bs, 1H), 2.50 (td, J = 12.6, 3.2 Hz, 1H), 1.70 (bs, 1H), 1.49 – 1.33 (m, 2H), 0.96 (d, J = 6.9 Hz, 3H); **^{13}C NMR** (151 MHz, D_2O, 299 K) δ 92.05 (d, J = 169.6 Hz), 48.81 – 47.93 (m), 44.21, 33.08 (d, J = 20.2 Hz), 28.13, 16.93; **^{13}C{sel-^{19}F at -202 ppm} NMR** (151 MHz, D_2O, 299 K) δ 92.02, 48.37, 44.20, 33.08, 28.12, 16.98; **^{19}F NMR** (564 MHz, D_2O, 299 K) δ -202.43; **^{19}F{^{1}H} NMR** (564 MHz, D_2O, 299 K) δ -202.43.

Computational observation

The calculated ΔG values in different solvents for the energetically lowest conformers are presented. In the scheme, the values of ΔG in water (ΔG gas phase) are given.

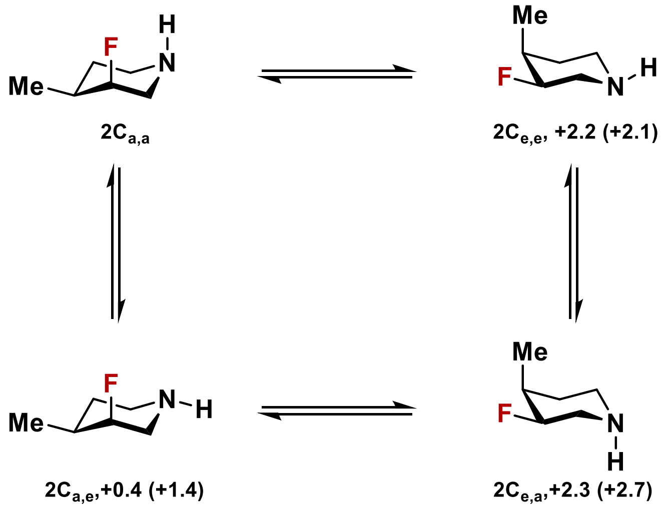

Solvent	ΔG(a,a→e,e)	ΔE_{elect}	ΔE_{hyperc}	ΔE_{steric}	$\mu_{a,a}$	$\mu_{e,e}$	Experimental
**H_2O**	+2.2	+13.5	+5.8	–17.4	2.3	2.8	axial
**gas phase**	+2.1	+12.4	+5.6	–16.1	1.6	2.1	-

All values are given in kcal/mol at 298 K.

2,2,2-Trifluoro-1-(*cis*-3-fluoro-5-methylpiperidin-1-yl)ethan-1-one (4A)

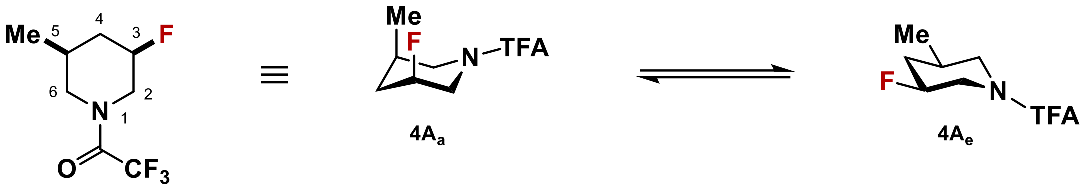

This compound was prepared following a recent literature procedure.[1]

<u>Experimental observation</u>

The product was present as a ~1:1 mixture of amide bond rotamers.

In **$CDCl_3$**: The orientation of the fluorine atom was assigned as equatorial due to the small values of ^3J(F,H_a).[1] [**Rot A: ^3J(2-H_a,3-F) = 4.4 Hz, ^3J(4-H_a,3-F) = 12.0 Hz, Rot B: ^3J(2-H_a,3-F) = 5.1 Hz, ^3J(4-H_a,3-F) = 12.0 Hz**]

<u>Computational observation</u>

The calculated ΔG is given for the energetically lowest conformers.

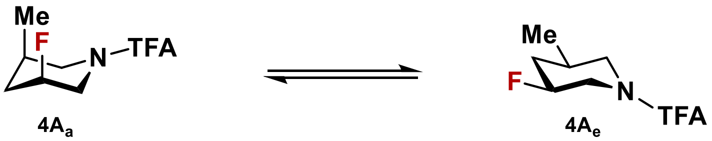

Solvent	ΔG(a→e)	ΔE_{elect}	ΔE_{hyperc}	ΔE_{steric}	μ_a	μ_e	Experimental
**$CHCl_3$**	–1.2	–3.6	+6.7	–3.8	7.5	5.6	equatorial
**gas phase**	–1.7	–3.2	+5.9	–3.9	5.5	4.3	-

All values are given in kcal/mol at 298 K.

***cis*-3-Fluoro-5-methylpiperidine hydrochloride (4B)**

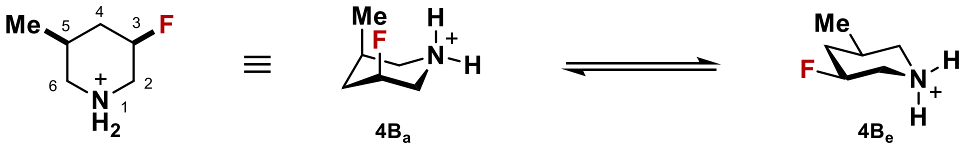

This compound was prepared following a recent literature procedure.[1]

Experimental observation

In **D_2O**: Due to peaks overlapping and broadening in NMR spectra, we could not verify the orientation of the fluorine atom through $^3J(F,H_a)$ values. Therefore additional NMR studies that includes NOE and HF/FH-HetNOE experiments were conducted.[1] All those experiments showed unequivocally that the fluorine atom adopts equatorial orientation.[1]

Computational observation

The calculated ΔG is given for the energetically lowest conformers.

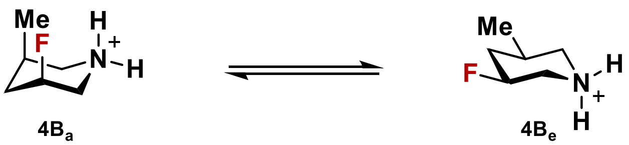

Solvent	ΔG(a→e)	ΔE_{elect}	ΔE_{hyperc}	ΔE_{steric}	μ_a	μ_e	Experimental
**H_2O**	–0.4	+10.3	+6.6	–16.7	7.4	8.9	equatorial
**gas phase**	+2.9	+23.4	+2.4	–22.5	5.3	6.8	-

All values are given in kcal/mol at 298 K.

cis-3-Fluoro-5-methylpiperidine (4C)

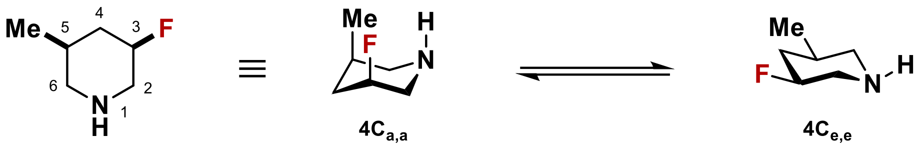

The preparation of this compound is described in the previous section.

<u>Experimental observation</u>

In **D_2O**: The orientation of the fluorine atom was assigned as equatorial due to the small values of ^3J(F,H_a). [**^3J(2-H_a,3-F) = 5.7 Hz, ^3J(4-H_a,3-F) = 10.6 Hz**]

^{1}H NMR (600 MHz, D_2O, 299 K) δ 4.64 (dtt, *J* = 48.6, 10.0, 4.7 Hz, 1H), 3.24 – 3.19 (m, 1H), 2.85 (dt, *J* = 12.5, 4.1 Hz, 1H), 2.46 (ddd, *J* = 11.8, 10.0, 5.7 Hz, 1H), 2.25 – 2.17 (m, 1H), 2.08 (dd, *J* = 12.6, 10.8 Hz, 1H), 1.73 – 1.63 (m, 1H), 1.31 – 1.20 (m, 1H), 0.95 (dd, *J* = 6.7, 1.1 Hz, 3H); **^{1}H{^{19}F} NMR** (600 MHz, D_2O, 299 K) δ 4.72 – 4.55 (m, 1H), 3.21 (dd, *J* = 11.6, 3.9 Hz, 1H), 2.85 (dd, *J* = 12.7, 4.1 Hz, 1H), 2.46 (dd, *J* = 12.0, 9.8 Hz, 1H), 2.24 – 2.19 (m, 1H), 2.08 (dd, *J* = 12.6, 10.8 Hz, 1H), 1.72 – 1.63 (m, 1H), 1.29 – 1.21 (m, 1H), 0.95 (d, *J* = 6.7 Hz, 3H); **^{13}C NMR** (151 MHz, D_2O, 299 K) δ 89.89 (d, *J* = 168.7 Hz), 51.18 (d, *J* = 1.8 Hz), 48.50 (d, *J* = 24.2 Hz), 38.73 (d, *J* = 16.1 Hz), 30.29 (d, *J* = 9.3 Hz), 18.09 (d, *J* = 1.1 Hz); **^{13}C{sel-^{19}F at -175 ppm} NMR** (151 MHz, D_2O, 299 K) δ 89.85, 51.16, 48.49, 38.72, 30.28, 18.07; **^{19}F NMR** (564 MHz, D_2O, 299 K) δ -175.97 (dd, *J* = 48.4, 4.7 Hz); **^{19}F{^{1}H} NMR** (564 MHz, D_2O, 299 K) δ -175.97.

Computational observation

The calculated ΔG values in different solvents for the energetically lowest conformers are presented. In the scheme, the values of ΔG in water (ΔG gas phase) are given.

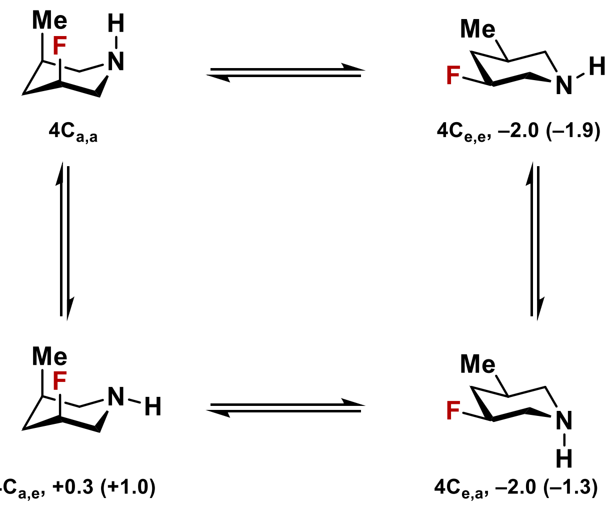

Solvent	ΔG(a,a→e,e)	ΔE_{elect}	ΔE_{hyperc}	ΔE_{steric}	$\mu_{a,a}$	$\mu_{e,e}$	Experimental
**H_2O**	–2.0	+13.0	+7.2	–21.7	2.4	2.8	equatorial
**gas phase**	–1.9	+6.6	+7.1	–15.1	1.6	2.2	-

All values are given in kcal/mol at 298 K.

2,2,2-Trifluoro-1-(*cis*-3-fluoro-2-methylpiperidin-1-yl)ethan-1-one (5A)

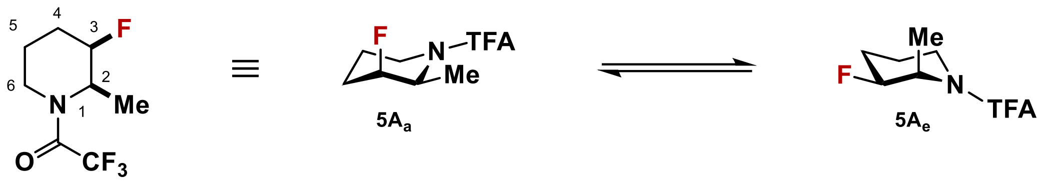

This compound was prepared following a recent literature procedure.[1]

Experimental observation

The product was present as a ~1.5:1 mixture of amide bond rotamers.

In **$CDCl_3$**: Due to peaks overlapping and broadening in NMR spectra, we could not verify the orientation of the fluorine atom through $^{3}J(F,H_a)$ values. Therefore additional NMR studies that includes NOE and HF/FH-HetNOE experiments were conducted.[1] All those experiments showed unequivocally that the fluorine atom adopts equatorial orientation.[1]

Computational observation

The calculated ΔG is given for the energetically lowest conformers.

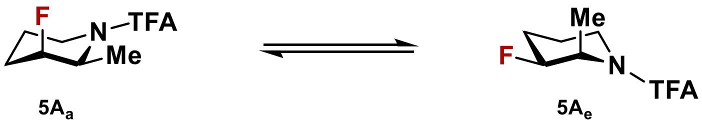

Solvent	$\Delta G(a\rightarrow e)$	ΔE_{elect}	ΔE_{hyperc}	ΔE_{steric}	μ_a	μ_e	Experimental
**$CHCl_3$**	–3.7	–5.1	+3.8	–2.1	7.7	5.6	equatorial
**gas phase**	–4.3	–6.4	+5.1	–2.6	5.6	4.1	-

All values are given in kcal/mol at 298 K.

cis-3-Fluoro-2-methylpiperidine hydrochloride (5B)

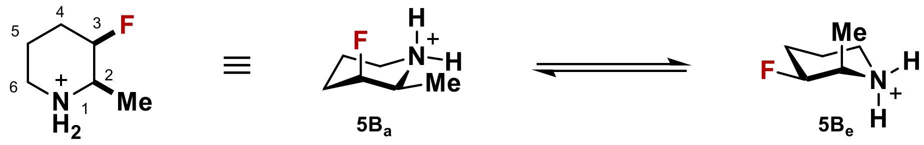

This compound was prepared following a recent literature procedure.[1]

<u>Experimental observation</u>

In **D_2O**: The orientation of the fluorine atoms was assigned as axial due to the large values of ^3J(F,H$_a$).[1] [**^3J(2-H$_a$,3-F) = 31.5 Hz, ^3J(4-H$_a$,3-F) = 46.0 Hz**]

<u>Computational observation</u>

The calculated ΔG is given for the energetically lowest conformers.

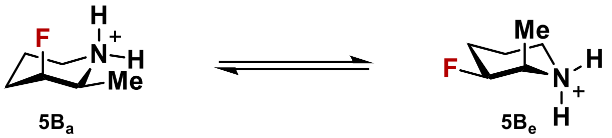

Solvent	ΔG(a→e)	ΔE_{elect}	ΔE_{hyperc}	ΔE_{steric}	μ_a	μ_e	Experimental
**H_2O**	+3.3	+10.2	+3.6	–10.5	6.3	8.7	axial
**gas phase**	+6.2	+15.1	–0.0	–8.8	4.5	6.6	-

All values are given in kcal/mol at 298 K.

cis-3-Fluoro-2-methylpiperidine (5C)

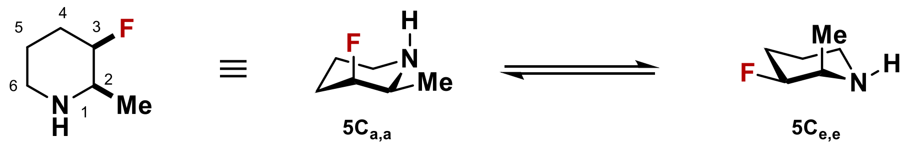

The preparation of this compound is described in the previous section.

Experimental observation

In **D_2O**: The orientation of the fluorine atom was assigned as axial due to the large value of ^3J(F,H_a). [**^3J(2-H_a,3-F) = 32.3 Hz**]

NMR spectra contain traces of the corresponding piperidine carboxylic acid due to a fast reaction of the free amine with carbon dioxide.

^{1}H NMR (600 MHz, D_2O, 299 K) δ 4.61 (d, *J* = 49.8 Hz, 1H), 2.98 – 2.91 (m, 1H), 2.76 (dqd, *J* = 32.3, 6.8, 1.4 Hz, 1H), 2.63 – 2.56 (m, 1H), 2.12 – 2.04 (m, 1H), 1.76 – 1.60 (m, 2H), 1.50 – 1.44 (m, 1H), 1.10 (d, *J* = 6.8 Hz, 3H); **^{1}H{^{19}F} NMR** (600 MHz, D_2O, 299 K) δ 4.61 (bs, 1H), 2.97 – 2.91 (m, 1H), 2.76 (q, *J* = 6.8 Hz, 1H), 2.59 (td, *J* = 12.5, 3.1 Hz, 1H), 2.12 – 2.05 (m, 1H), 1.76 – 1.60 (m, 2H), 1.49 – 1.44 (m, 1H), 1.10 (d, *J* = 6.8 Hz, 3H); **^{13}C NMR** (151 MHz, D_2O, 299 K) δ 91.28 (d, *J* = 166.0 Hz), 52.63 (d, *J* = 19.2 Hz), 44.49, 28.68 (d, *J* = 21.1 Hz), 19.29, 16.77 (d, *J* = 4.9 Hz); **^{13}C{sel-^{19}F at -201 ppm} NMR** (151 MHz, D_2O, 299 K) δ 91.27, 52.62, 44.48, 28.67, 19.27, 16.74; **^{19}F NMR** (564 MHz, D_2O, 299 K) δ -201.54; **^{19}F{^{1}H} NMR** (564 MHz, D_2O, 299 K) δ -201.54.

Computational observation

The calculated ΔG values in different solvents for the energetically lowest conformers are presented. In the scheme, the values of ΔG in water (ΔG gas phase) are given.

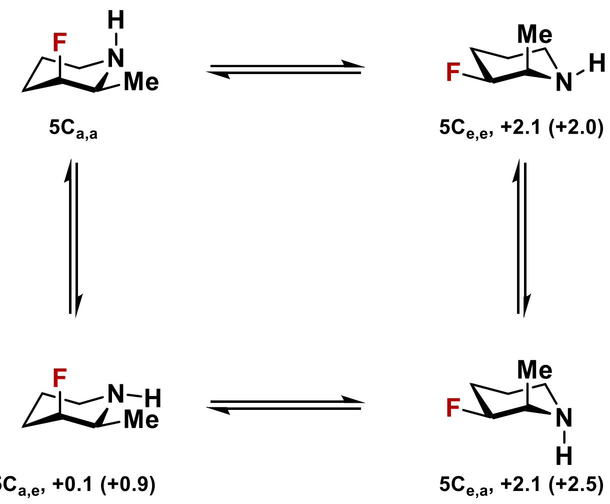

Solvent	ΔG(a,a→e,e)	ΔE_{elect}	ΔE_{hyperc}	ΔE_{steric}	$\mu_{a,a}$	$\mu_{e,e}$	Experimental
**H_2O**	+2.1	+18.4	+3.1	–19.6	2.4	2.8	axial
**gas phase**	+2.0	+11.8	+2.8	–12.8	1.6	2.0	-

All values are given in kcal/mol at 298 K.

2,2,2-Trifluoro-1-(*cis*-5-fluoro-2-methylpiperidin-1-yl)ethan-1-one (6A)

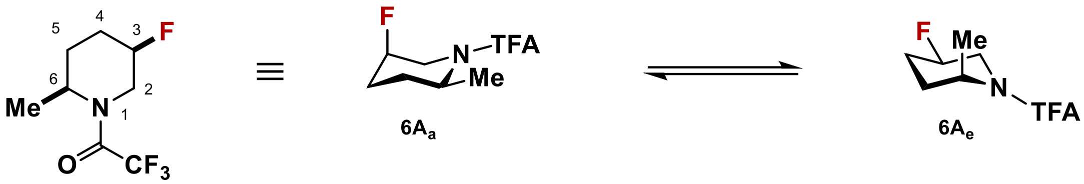

This compound was prepared following a recent literature procedure.[1]

<u>Experimental observation</u>

The product was present as a ~1:1 mixture of amide bond rotamers.

In **$CDCl_3$**: The orientation of the fluorine atom was assigned as equatorial due to the small values of $^3J(F,H_a)$.[1] [**Rot A: $^3J(2\text{-}H_a,3\text{-}F)$ = 5.8 Hz, Rot B: $^3J(2\text{-}H_a,3\text{-}F)$ = 5.8 Hz**]

<u>Computational observation</u>

The calculated ΔG is given for the energetically lowest conformers.

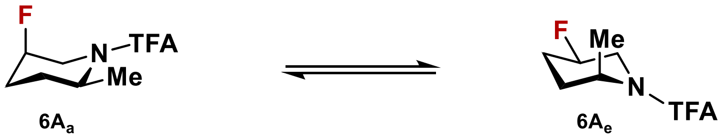

Solvent	ΔG(a→e)	ΔE_{elect}	ΔE_{hyperc}	ΔE_{steric}	μ_a	μ_e	Experimental
**$CHCl_3$**	–3.3	+13.4	+1.1	–17.5	7.6	4.8	equatorial
**gas phase**	–3.7	+22.5	–0.6	–25.3	5.6	3.7	-

All values are given in kcal/mol at 298 K.

***cis*-5-Fluoro-2-methylpiperidine hydrochloride (6B)**

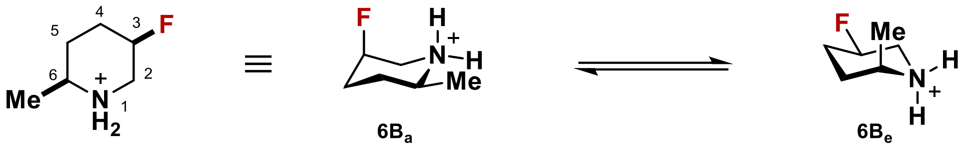

This compound was prepared following a recent literature procedure.[1]

<u>Experimental observation</u>

In **D_2O**: The orientation of the fluorine atom was assigned as axial due to the large value of ^3J(F,H_a).[1] [^{3}***J*****(2-H$_a$,3-F) = 40.7 Hz**]

<u>Computational observation</u>

The calculated ΔG is given for the energetically lowest conformers.

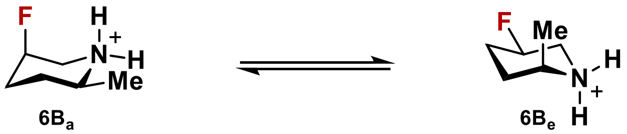

Solvent	ΔG(a→e)	ΔE_{elect}	ΔE_{hyperc}	ΔE_{steric}	μ_a	μ_e	Experimental
**H_2O**	+3.5	+11.2	+4.3	–11.9	5.9	8.3	axial
**gas phase**	+6.8	+22.2	+1.4	–16.8	4.3	6.3	-

All values are given in kcal/mol at 298 K.

***cis*-5-Fluoro-2-methylpiperidine (6C)**

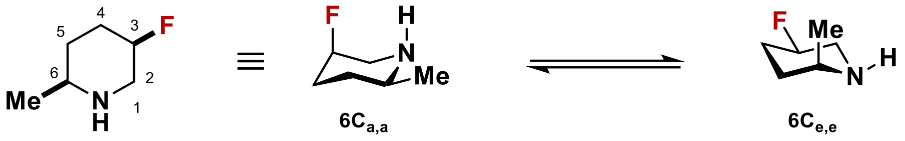

The preparation of this compound is described in the previous section.

Experimental observation

In **D_2O**: The orientation of the fluorine atom was assigned as axial due to the large values of ^3J(F,H_a). [**^3J(2-H_a,3-F) = 43.3 Hz, ^3J(4-H_a,3-F) = 48.1 Hz**]

NMR spectra contain traces of the corresponding piperidine carboxylic acid due to a fast reaction of the free amine with carbon dioxide.

^{1}H NMR (600 MHz, D_2O, 299 K) δ 4.76 (d, *J* = 47.7 Hz, 1H overlaps with solvent), 3.14 (ddt, *J* = 14.8, 12.3, 2.7 Hz, 1H), 2.75 (ddd, *J* = 43.3, 14.3, 1.5 Hz, 1H), 2.67 – 2.59 (m, 1H), 2.03 (ddt, *J* = 14.8, 11.5, 3.2 Hz, 1H), 1.68 (dtdd, *J* = 48.1, 14.8, 4.9, 2.3 Hz, 1H), 1.54 (ddt, *J* = 13.5, 5.1, 2.7 Hz, 1H), 1.36 (tdd, *J* = 13.5, 11.3, 4.1 Hz, 1H), 1.04 (d, *J* = 6.4 Hz, 3H); **^{1}H{^{19}F} NMR** (600 MHz, D_2O, 299 K) δ 4.76 (s, 1H), 3.14 (d, *J* = 14.2 Hz, 1H), 2.81 – 2.70 (m, 1H), 2.63 (dtd, *J* = 12.7, 6.4, 2.7 Hz, 1H), 2.03 (dm, *J* = 14.6 Hz, 1H), 1.77 – 1.61 (m, 1H), 1.57 – 1.51 (m, 1H), 1.36 (tdd, *J* = 13.5, 11.3, 4.1 Hz, 1H), 1.04 (d, *J* = 6.4 Hz, 3H); **^{13}C NMR** (151 MHz, D_2O, 299 K) δ 87.77 (d, *J* = 163.3 Hz), 50.21, 48.32 (dm, *J* = 20.2 Hz), 28.22 (d, *J* = 20.9 Hz), 27.71, 21.19; **^{13}C{sel-^{19}F at -186 ppm} NMR** (151 MHz, D_2O, 299 K) δ 87.74, 50.20, 48.31, 28.21, 27.70, 21.14; **^{19}F NMR** (564 MHz, D_2O, 299 K) δ -186.43 (qt, *J* = 49.2, 12.2 Hz); **^{19}F{^{1}H} NMR** (564 MHz, D_2O, 299 K) δ -186.43.

Computational observation

The calculated ΔG values in different solvents for the energetically lowest conformers are presented. In the scheme, the values of ΔG in water (ΔG gas phase) are given.

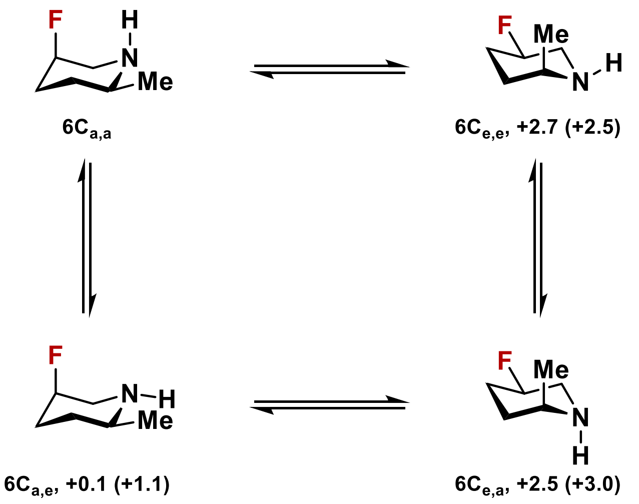

Solvent	ΔG(a,a→e,e)	ΔE_{elect}	ΔE_{hyperc}	ΔE_{steric}	$\mu_{a,a}$	$\mu_{e,e}$	Experimental
**H_2O**	+2.7	+11.7	+4.0	–13.4	2.4	2.9	axial
**gas phase**	+2.5	+11.6	+4.3	–13.5	1.6	2.3	-

All values are given in kcal/mol at 298 K.

2,2,2-Trifluoro-1-(*cis*-5-fluoro-2-(trifluoromethyl)piperidin-1-yl)ethan-1-one (7A)

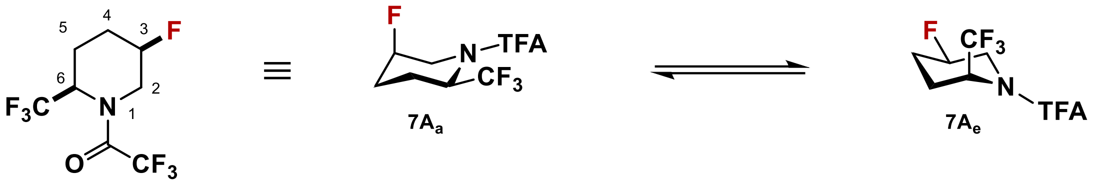

This compound was prepared following a recent literature procedure.[1]

<u>Experimental observation</u>

In **$CDCl_3$**: The orientation of the fluorine atom was assigned as equatorial due to the small values of ${}^3J$(F,H_a).[1] [$\mathbf{{}^3J(2\text{-}H_a,3\text{-}F) = 5.8\ Hz}$]

<u>Computational observation</u>

The calculated ΔG is given for the energetically lowest conformers.

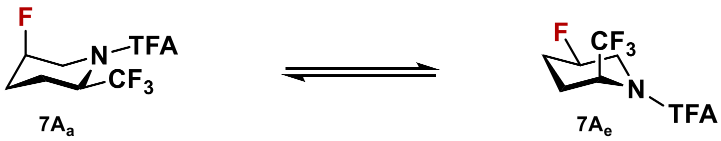

Solvent	ΔG(a→e)	ΔE_{elect}	ΔE_{hyperc}	ΔE_{steric}	μ_a	μ_e	Experimental
**$CHCl_3$**	–4.4	+19.6	+1.6	–25.6	8.4	4.0	equatorial
**gas phase**	–6.0	+20.8	–0.4	–26.4	6.2	2.9	-

All values are given in kcal/mol at 298 K.

cis-5-Fluoro-2-(trifluoromethyl)piperidine hydrochloride (7B)

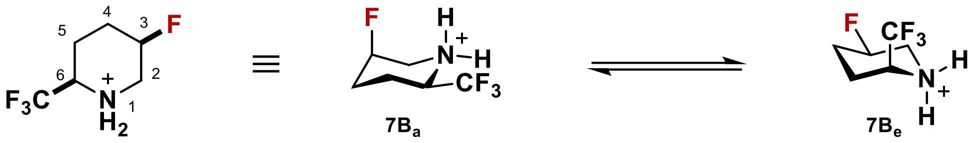

This compound was prepared following a recent literature procedure.[1]

<u>Experimental observation</u>

In **D_2O**: The orientation of the fluorine atom was assigned as axial due to the large values of ^3J(F,H_a).[1] [**^3J(2-H_a,3-F) = 39.7 Hz, ^3J(4-H_a,3-F) = 45.0 Hz**]

<u>Computational observation</u>

The calculated ΔG is given for the energetically lowest conformers.

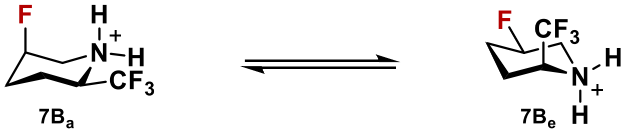

Solvent	ΔG(a→e)	ΔE_{elect}	ΔE_{hyperc}	ΔE_{steric}	μ_a	μ_e	Experimental
**H_2O**	+5.2	+19.5	+2.3	–17.3	7.7	9.4	axial
**gas phase**	+7.7	+27.6	–0.8	–19.2	6.3	7.0	-

All values are given in kcal/mol at 298 K.

cis-5-Fluoro-2-(trifluoromethyl)piperidine (7C)

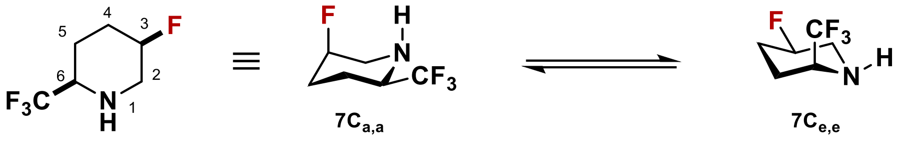

The preparation of this compound is described in the previous section.

Experimental observation

In **D_2O**: The orientation of the fluorine atom was assigned as axial due to the large value of ${}^3J$(F,H_a). [**${}^3J$(2-H_a,3-F) = 41.9 Hz**]

^{1}H NMR (600 MHz, D_2O, 299 K) δ 4.82 (d, *J* = 45.0 Hz, 1H overlaps with solvent), 3.34 (ddt, *J* = 14.9, 10.3, 5.0 Hz, 1H), 3.27 (ddt, *J* = 14.6, 11.9, 2.7 Hz, 1H), 2.85 (dd, *J* = 41.9, 14.4 Hz, 1H), 2.23 – 2.15 (m, 1H), 1.86 – 1.69 (m, 3H); **^{1}H{^{19}F} NMR** (600 MHz, D_2O, 299 K) δ 4.83 (bs, 1H), 3.39 – 3.31 (m, 1H), 3.27 (d, *J* = 14.4 Hz, 1H), 2.85 (dd, *J* = 14.4, 1.4 Hz, 1H), 2.22 – 2.17 (m, 1H), 1.84 – 1.73 (m, 3H); **^{13}C NMR** (151 MHz, D_2O, 299 K) δ 125.44 (q, *J* = 278.6 Hz), 86.51 (d, *J* = 166.2 Hz), 55.97 (q, *J* = 29.2 Hz), 47.63 (d, *J* = 20.2 Hz), 26.68 (d, *J* = 21.1 Hz), 18.65 (d, *J* = 1.7 Hz); **^{13}C{sel-^{19}F at -187 ppm} NMR** (151 MHz, D_2O, 299 K) δ 125.45 (q, *J* = 278.3 Hz), 86.50, 55.97 (q, *J* = 29.0 Hz), 47.64, 26.68, 18.65; **^{19}F NMR** (564 MHz, D_2O, 299 K) -77.19 (d, *J* = 7.2 Hz), -186.68 – -188.85 (m); **^{19}F{^{1}H} NMR** (564 MHz, D_2O, 299 K) δ -77.19, -187.79.

Computational observation

The calculated ΔG values in different solvents for the energetically lowest conformers are presented. In the scheme, the values of ΔG in water (ΔG gas phase) are given.

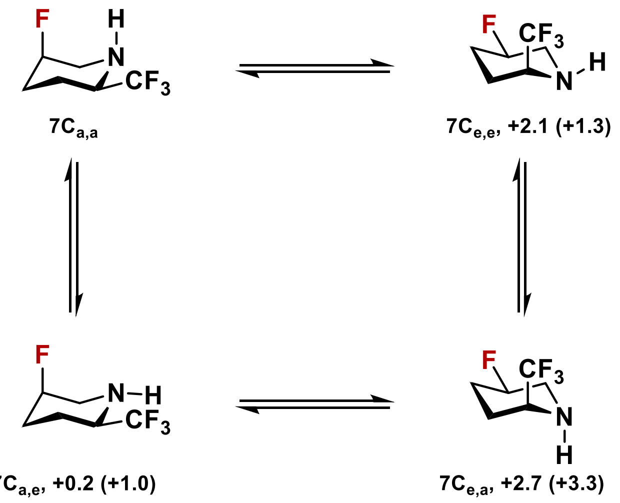

Solvent	ΔG(a,a→e,e)	ΔE_{elect}	ΔE_{hyperc}	ΔE_{steric}	$\mu_{a,a}$	$\mu_{e,e}$	Experimental
**H_2O**	+2.1	+32.5	–2.2	–28.4	4.7	2.8	axial
**gas phase**	+1.3	+30.7	–0.6	–29.0	3.4	2.1	-

All values are given in kcal/mol at 298 K.

2,2,2-Trifluoro-1-(*cis*-3-fluoro-4,5-dimethylpiperidin-1-yl)ethan-1-one (8A)

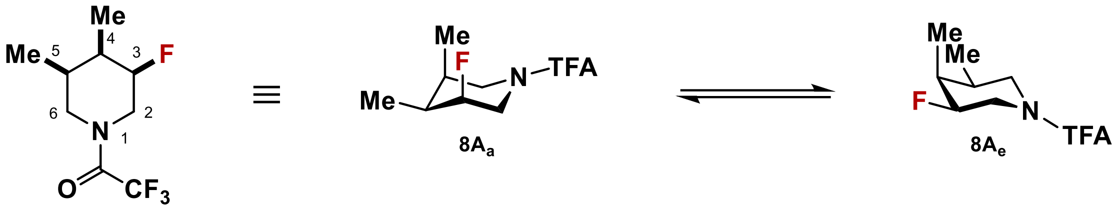

This compound was prepared following a recent literature procedure.[1]

Experimental observation

The product was present as a ~1:1 mixture of amide bond rotamers.

In **$CDCl_3$**: The orientation of the fluorine atom was assigned as axial due to the large values of $^{3}J(F,H_a)$.[1] [**Rot A: $^{3}J(2\text{-}H_a,3\text{-}F)$ = 30.4 Hz, $^{3}J(4\text{-}H_a,3\text{-}F)$ = 27.0 Hz, Rot B: $^{3}J(2\text{-}H_a,3\text{-}F)$ = 30.4 Hz, $^{3}J(4\text{-}H_a,3\text{-}F)$ = 27.0 Hz**]

Computational observation

The calculated ΔG is given for the energetically lowest conformers.

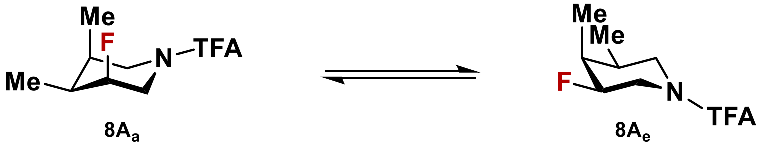

Solvent	ΔG(a→e)	ΔE_{elect}	ΔE_{hyperc}	ΔE_{steric}	μ_a	μ_e	Experimental
**$CHCl_3$**	+0.6	–0.3	+7.3	–6.2	7.4	5.8	axial
**gas phase**	+0.2	–3.6	+7.0	–3.1	5.4	4.8	-

All values are given in kcal/mol at 298 K.

cis-3-Fluoro-4,5-dimethylpiperidine hydrochloride (8B)

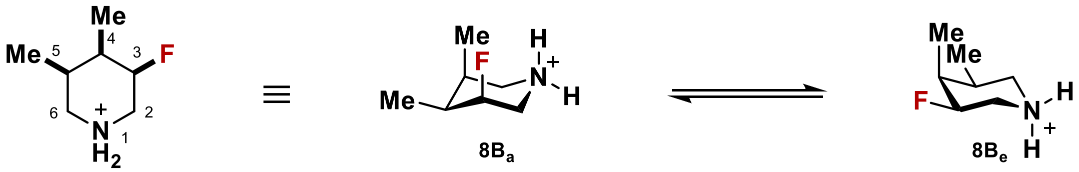

This compound was prepared following a recent literature procedure.[1]

<u>Experimental observation</u>

In **D_2O**: The orientation of the fluorine atom was assigned as axial due to the large value of ^3J(F,H_a).[1] [**^3J(2-H_a,3-F) = 34.3 Hz**]

<u>Computational observation</u>

The calculated ΔG is given for the energetically lowest conformers.

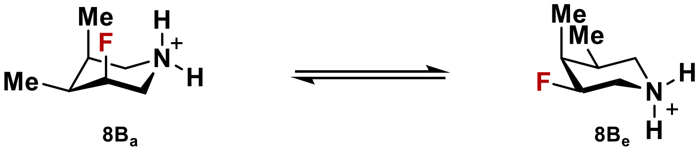

Solvent	ΔG(a→e)	ΔE_{elect}	ΔE_{hyperc}	ΔE_{steric}	μ_a	μ_e	Experimental
**H_2O**	+1.1	+17.0	+6.3	–21.8	8.5	9.6	axial
**gas phase**	+4.2	+24.5	+2.5	–22.7	6.0	7.1	-

All values are given in kcal/mol at 298 K.

***cis*-3-Fluoro-4,5-dimethylpiperidine (8C)**

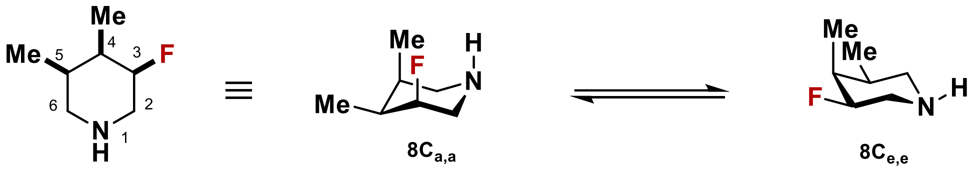

The preparation of this compound is described in the previous section.

Experimental observation

In **D_2O**: The dominant orientation of the fluorine atoms is assigned to be axial based on the values of $^{3}J(F,H_a)$. [**^{3}J(2-H_a,3-F) = 26.0 Hz, ^{3}J(4-H_a,3-F) = 26.3 Hz**]

NMR spectra contain mixture of diastereomers originated from the recently reported procedure (*d.r.* 95:5:0).[1] Only the signals of the major diastereomer are listed. The NMR spectra also contain traces of the corresponding piperidine carboxylic acid due to a fast reaction of the free amine with carbon dioxide.

^{1}H NMR (600 MHz, D_2O, 299 K) δ 4.64 (ddt, *J* = 48.0, 6.6, 3.4 Hz, 1H), 2.95 (ddd, *J* = 13.7, 9.4, 6.4 Hz, 1H), 2.79 (ddd, *J* = 26.0, 13.6, 3.3 Hz, 1H), 2.67 – 2.58 (m, 2H), 2.06 (dm, *J* = 26.3 Hz, 1H), 1.75 (ddp, *J* = 11.5, 7.1, 4.4, 3.6 Hz, 1H), 0.97 (d, *J* = 7.2 Hz, 3H), 0.95 (dd, *J* = 7.3, 1.8 Hz, 3H); **^{1}H{^{19}F} NMR** (600 MHz, D_2O, 299 K) δ 4.64 (bs, 1H), 2.95 (dd, *J* = 13.5, 6.4 Hz, 1H), 2.78 (dd, *J* = 13.7, 3.2 Hz, 1H), 2.67 – 2.58 (m, 2H), 2.12 – 2.01 (m, 1H), 1.75 (ddt, *J* = 9.8, 7.0, 3.5 Hz, 1H), 0.97 (d, *J* = 7.2 Hz, 3H), 0.95 (d, *J* = 7.3 Hz, 3H); **^{13}C NMR** (151 MHz, D_2O, 299 K) δ 93.10 (d, *J* = 171.0 Hz), 48.08, 45.64 (dm, *J* = 20.5 Hz), 35.71 (d, *J* = 17.2 Hz), 32.27 (d, *J* = 1.9 Hz), 13.84 (d, *J* = 3.5 Hz), 9.86 (bs); **^{13}C{sel-^{19}F at -188 ppm} NMR** (151 MHz, D_2O, 299 K) δ 93.08, 48.07, 45.64, 35.70, 32.26, 13.83, 9.88; **^{19}F NMR** (564 MHz, D_2O, 299 K) δ -188.53 (bs); **^{19}F{^{1}H} NMR** (564 MHz, D_2O, 299 K) δ -188.56 (bs).

Computational observation

The calculated ΔG values in different solvents for the energetically lowest conformers are presented. In the scheme, the values of ΔG in water (ΔG gas phase) are given.

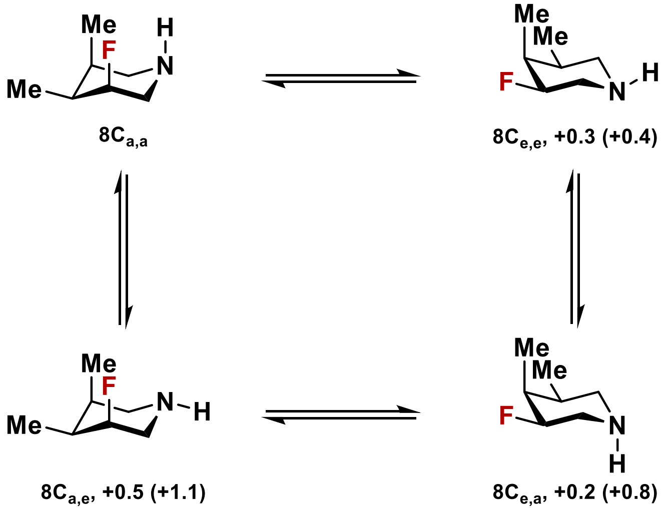

Solvent	ΔG(a,a→e,e)	ΔE_{elect}	ΔE_{hyperc}	ΔE_{steric}	$\mu_{a,a}$	$\mu_{e,e}$	Experimental
**H_2O**	+0.3	+20.1	+8.6	–28.4	2.4	2.8	axial
**gas phase**	+0.4	+17.6	+8.5	–25.8	1.6	2.1	-

All values are given in kcal/mol at 298 K.

1-(*cis*-3,5-Difluoro-4-methylpiperidin-1-yl)-2,2,2-trifluoroethan-1-one (9A)

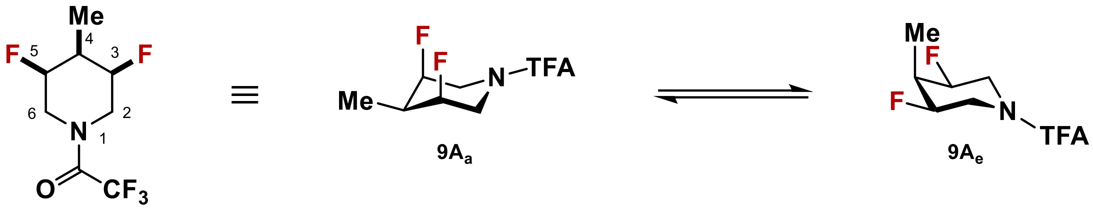

This compound was prepared following a recent literature procedure.[1]

<u>Experimental observation</u>

In **$CDCl_3$**: The orientation of the fluorine atoms was assigned as axial due to the large values of ^3J(F,H_a).[1] [**^3J(2-H_a,3-F) = 37.8 Hz, ^3J(4-H_a,3-F) = 34.9 Hz, ^3J(4-H_a,5-F) = 34.9 Hz, ^3J(6-H_a,5-F) = 35.6 Hz**]

<u>Computational observation</u>

The calculated ΔG is given for the energetically lowest conformers.

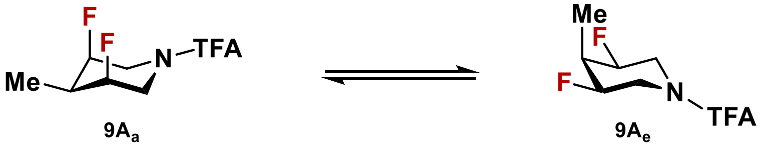

Solvent	ΔG(a→e)	ΔE_{elect}	ΔE_{hyperc}	ΔE_{steric}	μ_a	μ_e	Experimental
**$CHCl_3$**	+2.2	+8.1	+12.2	–18.2	8.7	3.4	axial
**gas phase**	+0.1	+5.0	+12.2	–17.4	6.3	2.6	-

All values are given in kcal/mol at 298 K.

cis-3,5-Difluoro-4-methylpiperidine hydrochloride (9B)

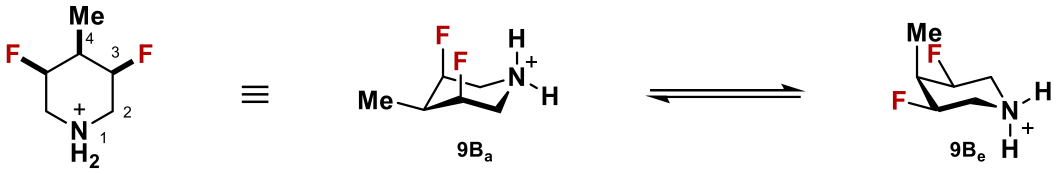

This compound was prepared following a recent literature procedure.[1]

<u>Experimental observation</u>

In **D_2O**: The orientation of the fluorine atoms was assigned as axial due to the large values of ^{3}J(F,H_a).[1] [**^{3}J(2-H_a,3-F) = 40.9 Hz, ^{3}J(4-H_a,3-F) = 38.1 Hz**]

<u>Computational observation</u>

The calculated ΔG is given for the energetically lowest conformers.

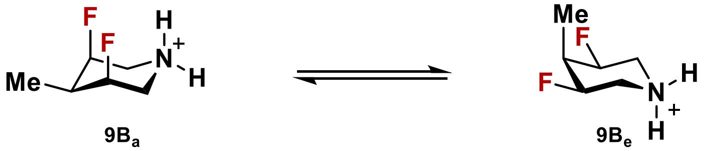

Solvent	ΔG(a→e)	ΔE_{elect}	ΔE_{hyperc}	ΔE_{steric}	μ_a	μ_e	Experimental
**H_2O**	+5.1	+11.5	+10.4	–16.3	10.0	10.6	axial
**gas phase**	+9.5	+21.8	+4.9	–17.1	7.2	8.0	-

All values are given in kcal/mol at 298 K.

***cis*-3,5-Difluoro-4-methylpiperidine (9C)**

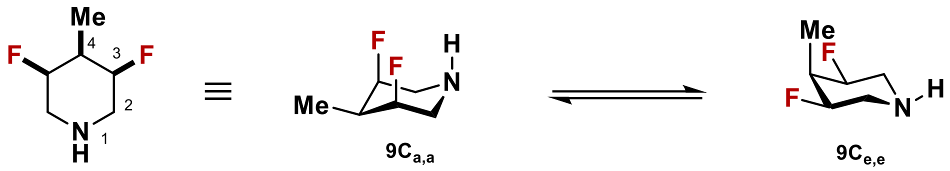

The preparation of this compound is described in the previous section.

<u>Experimental observation</u>

In **D_2O**: The orientation of the fluorine atoms was assigned as axial due to the large values of ^3J(F,H$_a$). [**^3J(2-H$_a$,3-F) = 43.3 Hz, ^3J(4-H$_a$,3-F) = 39.4 Hz**]

^{1}H NMR (600 MHz, D_2O, 299 K) δ 4.63 (dd, J = 47.9, 2.2 Hz, 2H), 3.24 (t, J = 13.5 Hz, 2H), 2.76 (dd, J = 43.3, 14.8 Hz, 2H), 1.95 (tm, J = 39.4 Hz, 1H), 1.16 (d, J = 7.2 Hz, 3H); **^{1}H{^{19}F} NMR** (600 MHz, D_2O, 299 K) δ 4.64 – 4.61 (m, 2H), 3.24 (d, J = 14.9 Hz, 2H), 2.76 (d, J = 15.1 Hz, 2H), 1.98 – 1.91 (m, 1H), 1.16 (d, J = 7.2 Hz, 3H); **^{13}C NMR** (151 MHz, D_2O, 299 K) δ 90.65 (d, J = 173.3 Hz), 47.84 – 47.51 (m), 35.29 (t, J = 19.2 Hz), 12.48; **^{13}C{sel-^{19}F at -197 ppm} NMR** (151 MHz, D_2O, 299 K) δ 90.64, 47.69, 35.28, 12.46; **^{19}F NMR** (564 MHz, D_2O, 299 K) δ -196.89 – -197.33 (m); **^{19}F{^{1}H} NMR** (564 MHz, D_2O, 299 K) δ -197.11.

Computational observation

The calculated ΔG values in different solvents for the energetically lowest conformers are presented. In the scheme, the values of ΔG in water (ΔG gas phase) are given.

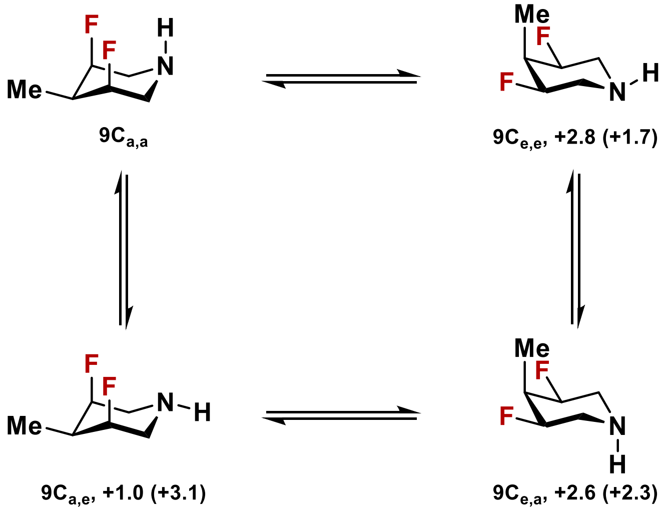

Solvent	ΔG(a,a→e,e)	ΔE_{elect}	ΔE_{hyperc}	ΔE_{steric}	$\mu_{a,a}$	$\mu_{e,e}$	Experimental
**H_2O**	+2.8	+19.0	+12.0	–28.4	4.4	2.6	axial
**gas phase**	+1.7	+18.6	+12.8	–29.8	2.9	2.0	-

All values are given in kcal/mol at 298 K.

2,2,2-Trifluoro-1-(4-fluoropiperidin-1-yl)ethan-1-one (10A)

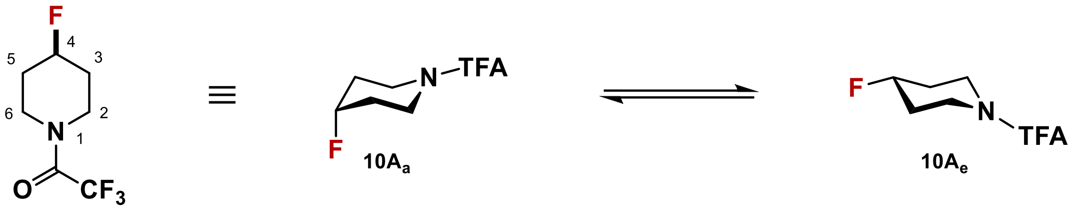

This compound was prepared following a recent literature procedure.[1]

Experimental observation

In **CDCl3**: The orientation of the fluorine atom was assigned as axial due to the large values of ^{3}J(F,H$_a$).[1] [^{3}J**(3-H$_a$,4-F) = 34.5 Hz,** ^{3}J**(5-H$_a$,4-F) = 34.5 Hz**]

Computational observation

The calculated ΔG is given for the energetically lowest conformers.

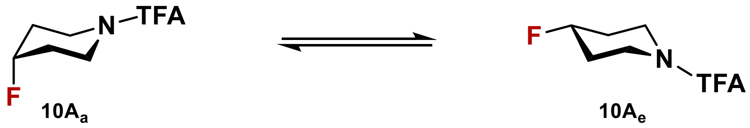

Solvent	ΔG(a→e)	ΔE_{elect}	ΔE_{hyperc}	ΔE_{steric}	μ_a	μ_e	Experimental
**$CHCl_3$**	+0.4	+6.7	+1.6	–8.1	5.0	4.9	axial
**gas phase**	+0.7	+9.6	+0.6	–9.5	3.9	3.7	-

All values are given in kcal/mol at 298 K.

4-Fluoropiperidine hydrochloride (10B)

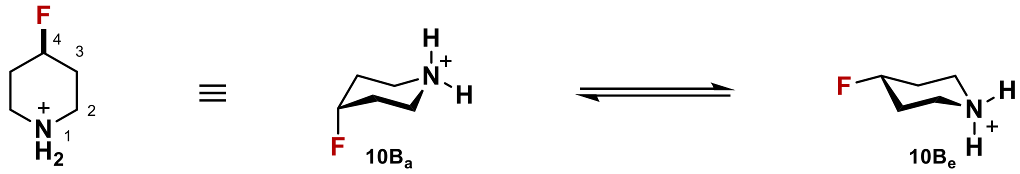

This compound was prepared following a recent literature procedure.[1]

<u>Experimental observation</u>

In **D2O**: Due to peaks overlapping and broadening in NMR spectra, we could not verify the orientation of the fluorine atom through $^3J(F,H_a)$ values. Therefore additional NMR studies that includes NOE and HF/FH-HetNOE experiments were conducted.[1] All those experiments showed a dynamic behavior where the equilibrium favors equatorial fluorine orientation.[1]

<u>Computational observation</u>

The calculated ΔG is given for the energetically lowest conformers.

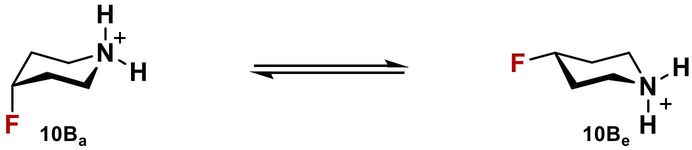

Solvent	ΔG(a→e)	ΔE_{elect}	ΔE_{hyperc}	ΔE_{steric}	μ_a	μ_e	Experimental
**H_2O**	+1.0	+12.4	2.5	–14.2	8.5	10.2	equatorial dominant
**gas phase**	+3.0	+21.5	–0.1	–18.2	6.4	8.0	-

All values are given in kcal/mol at 298 K.

4-Fluoropiperidine (10C)

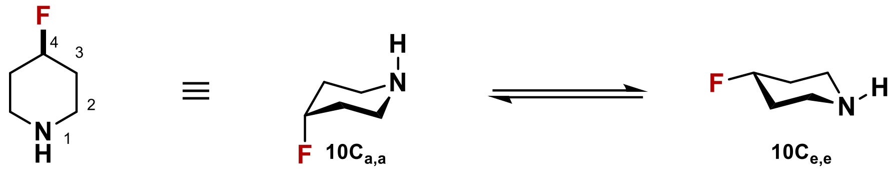

The preparation of this compound is described in the previous section.

Experimental observation

In $\mathbf{D_2O}$: The orientation of the fluorine atom was assigned as equatorial due to the small value of ^{3}J(F,H_a). [$\mathbf{^{3}\mathit{J}(3\text{-}H_a,4\text{-}F) = 10.6\ Hz}$]

The NMR spectra contain traces of defluorinated side-product, originated from the recently reported procedure,[1] which were inseparable from the product.

^{1}**H NMR** (600 MHz, D_2O, 299 K) δ 4.91 (dtt, *J* = 48.3, 7.3, 3.5 Hz, 1H), 3.08 (ddt, *J* = 11.7, 7.3, 3.4 Hz, 2H), 2.81 (ddd, *J* = 12.4, 7.5, 4.0 Hz, 2H), 1.99 (dddd, *J* = 25.5, 12.1, 8.2, 3.8 Hz, 2H), 1.81 (dddd, *J* = 21.1, 10.6, 7.4, 4.4 Hz, 2H); ^{1}**H{^{19}F} NMR** (600 MHz, D_2O, 299 K) δ 4.90 (bs, 1H), 3.08 (ddd, *J* = 12.3, 8.0, 3.9 Hz, 2H), 2.81 (ddd, *J* = 12.4, 7.5, 4.0 Hz, 2H), 2.04 – 1.95 (m, 2H), 1.85 – 1.76 (m, 2H); ^{13}**C NMR** (151 MHz, D_2O, 299 K) δ 89.49 (d, *J* = 166.3 Hz), 41.13 (d, *J* = 6.6 Hz), 30.44 (d, *J* = 19.0 Hz); ^{13}**C{sel-^{19}F at -176 ppm} NMR** (151 MHz, D_2O, 299 K) δ 89.50, 41.13, 30.44; ^{19}**F NMR** (564 MHz, D_2O, 299 K) δ -176.16 (bs); ^{19}**F{^{1}H} NMR** (564 MHz, D_2O, 299 K) δ -176.16 (bs).

Computational observation

The calculated ΔG values in different solvents for the energetically lowest conformers are presented. In the scheme, the values of ΔG in water (ΔG gas phase) are given.

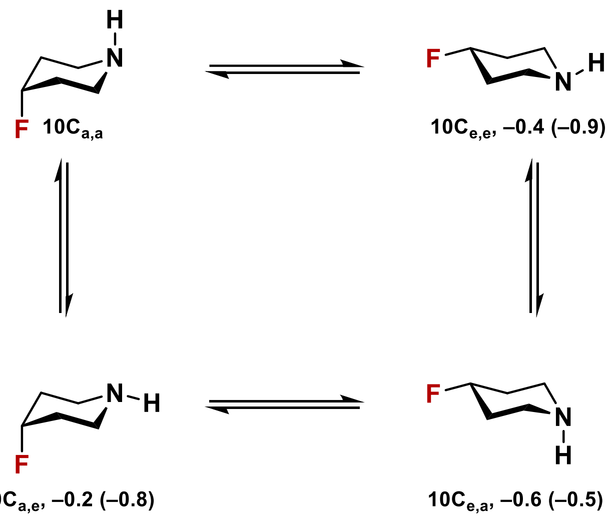

Solvent	ΔG(a,a→e,e)	ΔE_{elect}	ΔE_{hyperc}	ΔE_{steric}	$\mu_{a,a}$	$\mu_{e,e}$	Experimental
**H_2O**	–0.4	+17.1	+2.8	–20.6	3.4	3.6	equatorial
**gas phase**	–0.9	+8.1	+1.9	–10.8	2.4	2.7	-

All values are given in kcal/mol at 298 K.

2,2,2-Trifluoro-1-(*cis*-4-fluoro-3-methylpiperidin-1-yl)ethan-1-one (11A)

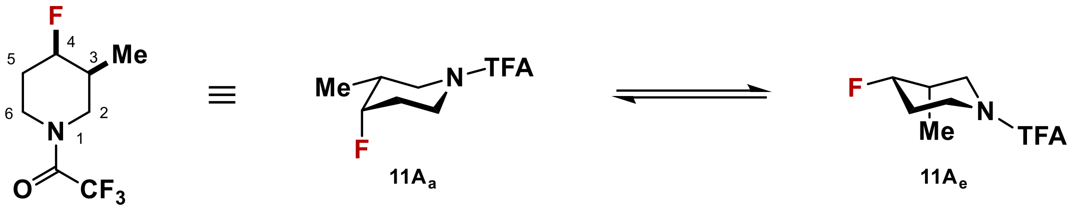

This compound was prepared following a recent literature procedure.[1]

Experimental observation

The product was present as a ~1:1 mixture of amide bond rotamers.

In **$CDCl_3$**: The orientation of the fluorine atom was assigned as axial due to the large values of $^{3}J(F,H_a)$.[1] [**Rot A: ^{3}J(3-H_a,4-F) = 33.5 Hz, ^{3}J(5-H_a,4-F) = 41.2 Hz, Rot B: ^{3}J(3-H_a,4-F) = 33.5 Hz, ^{3}J(5-H_a,4-F) = 41.2 Hz**]

Computational observation

The calculated ΔG is given for the energetically lowest conformers.

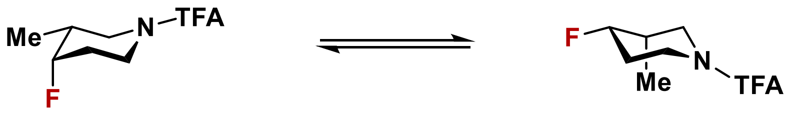

Solvent	$\Delta G(a \rightarrow e)$	ΔE_{elect}	ΔE_{hyperc}	ΔE_{steric}	μ_a	μ_e	Experimental
**$CHCl_3$**	+1.7	+4.2	+1.2	–4.4	5.0	4.7	axial
**gas phase**	+1.8	+0.7	+0.4	+0.2	3.9	3.4	-

All values are given in kcal/mol at 298 K.

***cis*-4-Fluoro-3-methylpiperidine hydrochloride (11B)**

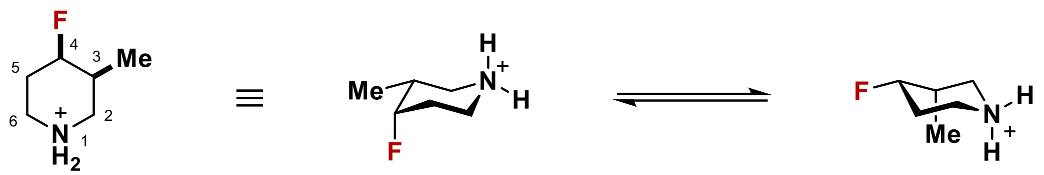

This compound was prepared following a recent literature procedure.[1]

Experimental observation

In **D_2O**: The orientation of the fluorine atom was assigned as axial due to the large values of $^{3}J(F,H_a)$.[1] [**$^{3}J(3\text{-}H_a,4\text{-}F)$ = 34.5 Hz, $^{3}J(5\text{-}H_a,4\text{-}F)$ = 43.3 Hz**]

Computational observation

The calculated ΔG is given for the energetically lowest conformers.

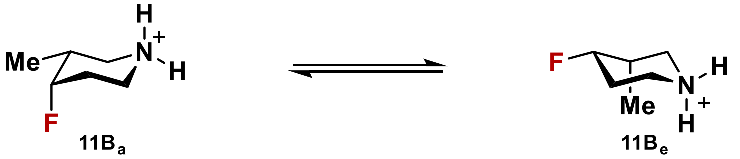

Solvent	ΔG(a→e)	ΔE_{elect}	ΔE_{hyperc}	ΔE_{steric}	μ_a	μ_e	Experimental
**H_2O**	+2.3	+7.1	+0.8	–5.5	9.1	10.3	axial
**gas phase**	+3.9	+8.1	–1.6	–2.7	6.7	7.8	-

All values are given in kcal/mol at 298 K.

***cis*-4-Fluoro-3-methylpiperidine (11C)**

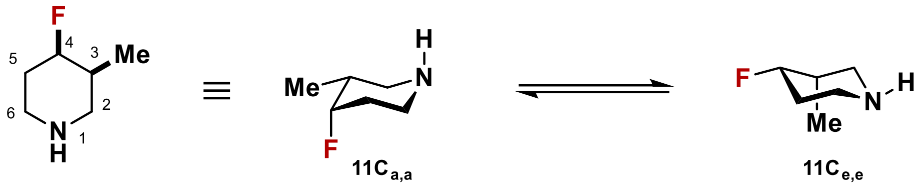

The preparation of this compound is described in the previous section.

<u>Experimental observation</u>

In **D_2O**: The orientation of the fluorine atom was assigned as axial due to the large values of ^3J(F,H_a). [**^3J(3-H_a,4-F) = 35.6 Hz, ^3J(5-H_a,4-F) = 43.0 Hz**]

^{1}H NMR (600 MHz, D_2O, 299 K) δ 4.85 (dm, J = 49.2 Hz, 1H), 2.79 – 2.72 (m, 3H), 2.55 (ddd, J = 12.6, 10.9, 1.6 Hz, 1H), 2.01 – 1.93 (m, 1H), 1.83 (dm, J = 35.6 Hz, 1H overlaps with 5-H_a), 1.72 (dm, J = 43.0 Hz, 1H overlaps with 3-H_a), 0.95 (d, J = 7.0 Hz, 3H); **^{1}H{^{19}F} NMR** (600 MHz, D_2O, 299 K) δ 4.85 (bs, 1H), 2.79 – 2.72 (m, 3H), 2.55 (dd, J = 12.7, 11.0 Hz, 1H), 1.97 (dq, J = 14.6, 3.4 Hz, 1H), 1.87 – 1.79 (m, 1H), 1.77 – 1.68 (m, 1H), 0.95 (d, J = 7.0 Hz, 3H); **^{13}C NMR** (151 MHz, D_2O, 299 K) δ 92.99 (d, J = 166.5 Hz), 46.47 (d, J = 2.2 Hz), 39.25, 34.18 (d, J = 19.2 Hz), 30.12 (d, J = 20.2 Hz), 13.66 (d, J = 4.3 Hz); **^{13}C{sel-^{19}F at -198 ppm} NMR** (151 MHz, D_2O, 299 K) δ 92.99, 46.47, 39.25, 34.18, 30.12, 13.66; **^{19}F NMR** (564 MHz, D_2O, 299 K) δ -198.52 (bs); **^{19}F{^{1}H} NMR** (564 MHz, D_2O, 299 K) δ -198.62 (bs).

<u>Computational observation</u>

The calculated ΔG values in different solvents for the energetically lowest conformers are presented. In the scheme, the values of ΔG in water (ΔG gas phase) are given.

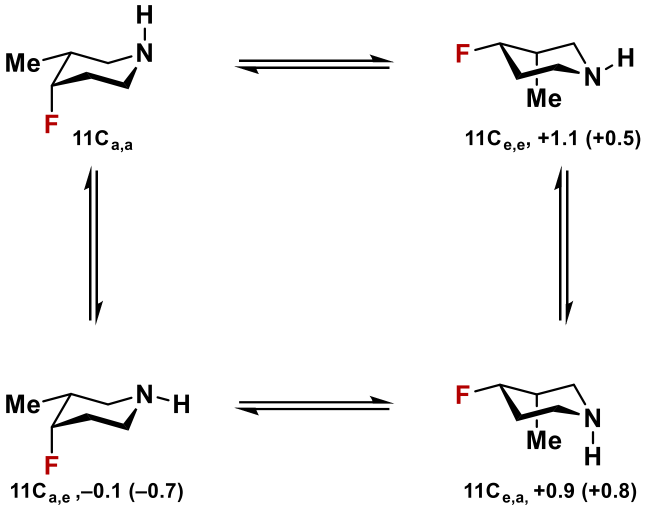

Solvent	ΔG(a,a→e,e)	ΔE_{elect}	ΔE_{hyperc}	ΔE_{steric}	$\mu_{a,a}$	$\mu_{e,e}$	Experimental
**H_2O**	+1.1	–4.9	+2.8	+2.8	3.5	3.6	axial
**gas phase**	+0.5	+4.8	+2.1	–6.6	2.3	2.6	-

All values are given in kcal/mol at 298 K.

2,2,2-Trifluoro-1-(*cis*-4-fluoro-2-methylpiperidin-1-yl)ethan-1-one (12A)

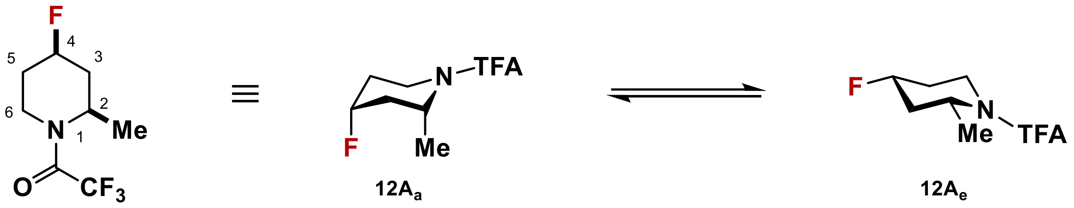

This compound was prepared following a recent literature procedure.[1]

Experimental observation

The product was present as a ~1:1 mixture of amide bond rotamers.

In **$CDCl_3$**: The orientation of the fluorine atom was assigned as axial due to the large values of ^3J(F,H_a).[1] [**Rot A: ^3J(3-H_a,4-F) = 47.0 Hz, ^3J(5-H_a,4-F) = 44.0 Hz, Rot B: ^3J(3-H_a,4-F) = 47.0 Hz, ^3J(5-H_a,4-F) = 44.0 Hz**]

Computational observation

The calculated ΔG is given for the energetically lowest conformers.

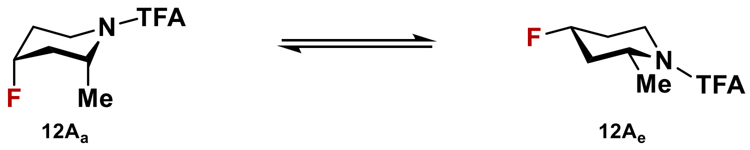

Solvent	ΔG(a→e)	ΔE_{elect}	ΔE_{hyperc}	ΔE_{steric}	μ_a	μ_e	Experimental
**$CHCl_3$**	+3.6	+11.8	+4.1	–12.4	5.1	5.4	axial
**gas phase**	+3.7	+15.2	+3.5	–15.1	4.0	3.9	-

All values are given in kcal/mol at 298 K.

cis-4-Fluoro-2-methylpiperidine hydrochloride (12B)

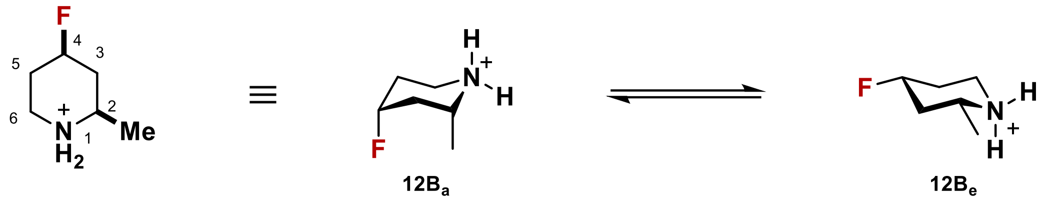

This compound was prepared following a recent literature procedure.[1]

Experimental observation

In **D_2O**: The orientation of the fluorine atom was assigned as equatorial due to the small values of ^{3}J(F,H_a).[1] [**^{3}J(3-H_a,4-F) = 10.5 Hz, ^{3}J(5-H_a,4-F) = 10.0 Hz**]

Computational observation

The calculated ΔG is given for the energetically lowest conformers.

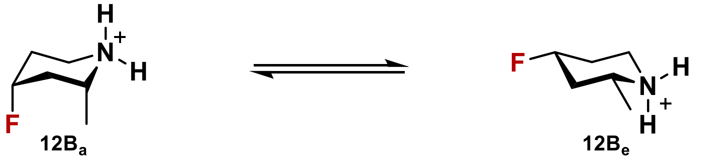

Solvent	ΔG(a→e)	ΔE_{elect}	ΔE_{hyperc}	ΔE_{steric}	μ_a	μ_e	Experimental
**H_2O**	–1.7	+4.7	+3.7	–9.6	8.4	9.2	equatorial
**gas phase**	+0.4	+15.5	+1.1	–15.8	6.1	7.1	-

All values are given in kcal/mol at 298 K.

***cis*-4-Fluoro-2-methylpiperidine (12C)**

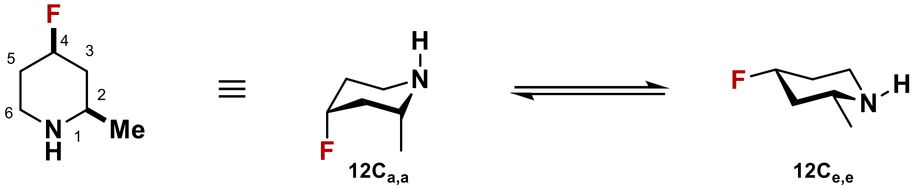

The preparation of this compound is described in the previous section.

Experimental observation

In **D_2O**: The orientation of the fluorine atom was assigned as equatorial due to the small values of ^3J(F,H$_a$). [$\mathbf{^3J}$**(3-H$_a$,4-F) = 11.5 Hz,** $\mathbf{^3J}$**(5-H$_a$,4-F) = 10.1 Hz**]

NMR spectra contain mixture of diastereomers originated from the recently reported procedure (*d.r.* 92:8).[1] Only the signals of the major diastereomer are listed.

^{1}H NMR (600 MHz, D_2O, 299 K) δ 4.70 (dm, *J* = 49.1 Hz, 1H), 3.09 – 3.00 (m, 1H), 2.70 – 2.61 (m, 1H), 2.54 (tm, *J* = 12.8 Hz, 1H), 2.17 – 2.11 (m, 1H), 2.10 – 2.04 (m, 1H), 1.47 (dm, *J* = 10.1 Hz, 1H), 1.21 (dm, *J* = 11.5 Hz, 1H), 1.10 (d, *J* = 6.4 Hz, 3H); **^{1}H{^{19}F} NMR** (600 MHz, D_2O, 299 K) δ 4.70 (tt, *J* = 10.8, 5.0 Hz, 1H), 3.05 (ddd, *J* = 13.0, 4.8, 2.7 Hz, 1H), 2.65 (tdd, *J* = 12.7, 6.3, 2.4 Hz, 1H), 2.54 (td, *J* = 13.0, 2.7 Hz, 1H), 2.13 (ddt, *J* = 11.6, 4.7, 2.1 Hz, 1H), 2.07 (ddt, *J* = 11.7, 4.9, 2.4 Hz, 1H), 1.47 (qd, *J* = 12.0, 4.7 Hz, 1H), 1.21 (q, *J* = 11.4 Hz, 1H), 1.10 (d, *J* = 6.4 Hz, 3H); **^{13}C NMR** (151 MHz, D_2O, 299 K) δ 92.02 (d, *J* = 167.0 Hz), 49.40 (d, *J* = 12.0 Hz), 42.32 (d, *J* = 12.7 Hz), 39.88 (d, *J* = 16.0 Hz), 31.64 (d, *J* = 17.1 Hz), 20.89 (d, *J* = 1.4 Hz); **^{13}C{sel-^{19}F at -166 ppm} NMR** (151 MHz, D_2O, 299 K) δ 92.02, 49.40, 42.32, 39.89, 31.64, 20.92; **^{19}F NMR** (564 MHz, D_2O, 299 K) δ -166.35 (dm, *J* = 50.0 Hz); **^{19}F{^{1}H} NMR** (564 MHz, D_2O, 299 K) δ -166.35.

Computational observation

The calculated ΔG values in different solvents for the energetically lowest conformers are presented. In the scheme, the values of ΔG in water (ΔG gas phase) are given.

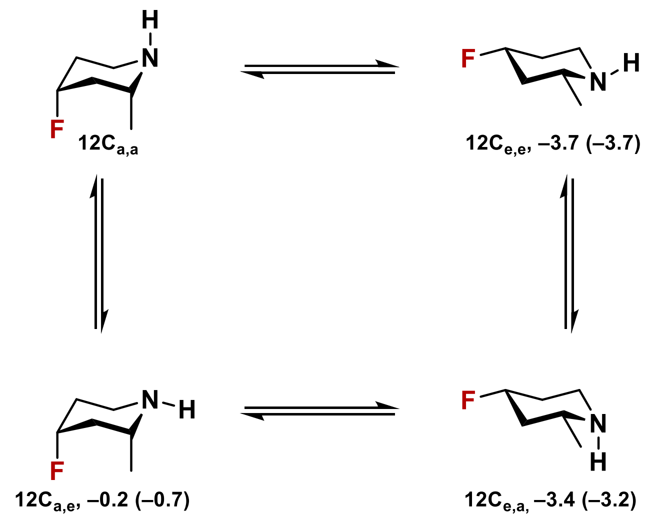

Solvent	ΔG(a,a→e,e)	ΔE_{elect}	ΔE_{hyperc}	ΔE_{steric}	$\mu_{a,a}$	$\mu_{e,e}$	Experimental
**H_2O**	–3.7	+4.0	+3.8	–11.1	3.4	3.8	equatorial
**gas phase**	–3.7	+5.3	+2.4	–11.1	2.3	2.7	-

All values are given in kcal/mol at 298 K.

1-(*cis*-3,5-Difluoropiperidin-1-yl)ethan-1-one (13)

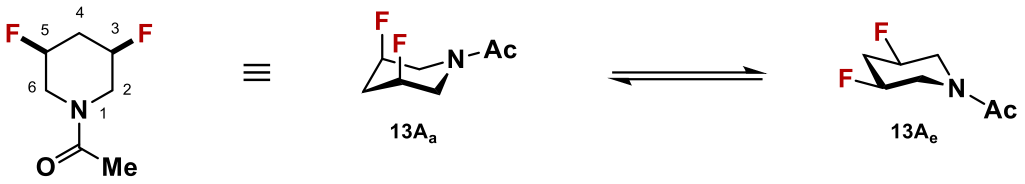

For this particular sample we conducted NMR experiments in different solvents in order to investigate the effect of solvent's dipole moment on the orientation of the fluorine atoms.

The preparation of this compound is described in the previous section.

Experimental observation

In **$CDCl_3$**: The orientation of the fluorine atoms was assigned as axial due to the large values of ^3J(F,H$_a$). [**^3J(2-H$_a$,3-F) = 29.0 Hz, ^3J(6-H$_a$,5-F) = 29.9 Hz, ^3J(4-H$_a$,3-F) = 33.5 Hz, ^3J(4-H$_a$,5-F) = 33.5 Hz**]

^{1}H NMR (600 MHz, $CDCl_3$, 299 K) δ 4.71 (d, J = 46.5 Hz, 2H), 4.43 – 4.35 (m, 1H), 3.90 – 3.82 (m, 1H), 3.46 (dd, J = 29.0, 14.5 Hz, 1H), 3.27 (dd, J = 29.9, 14.3 Hz, 1H), 2.38 – 2.28 (m, 1H), 2.14 (s, 3H), 2.06 (tdt, J = 33.5, 15.0, 3.2 Hz, 1H overlaps with Me); **^{1}H{^{19}F} NMR** (600 MHz, $CDCl_3$, 299 K) δ 4.74 – 4.67 (m, 2H), 4.40 (dm, J = 14.2 Hz, 1H), 3.86 (dd, J = 14.4, 4.6 Hz, 1H), 3.46 (d, J = 14.5 Hz, 1H), 3.27 (d, J = 14.2 Hz, 1H), 2.33 (dm, J = 15.0 Hz, 1H), 2.14 (s, 3H), 2.07 (dt, J = 14.9, 3.2 Hz, 1H); **^{13}C NMR** (151 MHz, $CDCl_3$, 299 K) δ 170.44, 84.52 (d, J = 178.2 Hz), 84.36 (d, J = 179.5 Hz), 49.83 (d, J = 23.4 Hz), 44.88 (d, J = 23.8 Hz), 34.44 (t, J = 20.9 Hz), 21.45; **^{13}C{sel-^{19}F at -183 ppm} NMR** (151 MHz, $CDCl_3$, 299 K) δ 170.44, 84.53, 84.37, 49.83, 44.88, 34.44, 21.45; **^{19}F NMR** (564 MHz, $CDCl_3$, 299 K) δ -182.77 – -183.32 (m); **^{19}F{^{1}H} NMR** (564 MHz, $CDCl_3$, 299 K) δ -182.99 (d, J = 11.1 Hz), -183.10 (d, J = 11.1 Hz).

ESI-MS: calculated [$C_7H_{11}NOF_2$ +Na]$^+$: 186.0701, found 186.0717.

In **DMSO**: The orientation of the fluorine atoms was assigned as axial due to the large values of ^3J(F,H$_a$). [**^3J(2-H$_a$,3-F) = 36.2 Hz, ^3J(6-H$_a$,5-F) = 37.5 Hz, ^3J(4-H$_a$,3-F) = 40.6 Hz, ^3J(4-H$_a$,5-F) = 40.6 Hz**]

^{1}H NMR (600 MHz, DMSO-d_6, 299 K) δ 4.81 (dm, J = 46.7 Hz, 1H), 4.76 (dm, J = 46.5 Hz, 1H), 4.44 (dddt, J = 14.4, 12.4, 3.8, 2.0 Hz, 1H), 3.96 (dddt, J = 15.0, 11.2, 3.8, 2.0 Hz, 1H), 3.42 (ddd,

J = 36.2, 14.9, 1.9 Hz, 1H), 3.01 (ddd, *J* = 37.5, 14.2, 2.1 Hz, 1H), 2.24 – 2.15 (m, 1H overlaps with 4-H_a), 2.07 (tdt, *J* = 40.6, 15.6, 3.3 Hz, 1H overlaps with 4-H_e and Me), 2.01 (s, 3H overlaps with 4-H_a); **^{1}H{^{19}F} NMR** (600 MHz, DMSO-d_6, 299 K) δ 4.83 – 4.79 (m, 1H), 4.78 – 4.75 (m, 1H), 4.44 (ddt, *J* = 14.3, 3.8, 2.0 Hz, 1H), 3.96 (ddt, *J* = 14.9, 3.9, 2.0 Hz, 1H), 3.42 (dd, *J* = 14.8, 1.9 Hz, 1H), 3.01 (dd, *J* = 14.2, 2.0 Hz, 1H), 2.19 (dtt, *J* = 15.6, 3.5, 2.1 Hz, 1H), 2.07 (dt, *J* = 15.5, 3.4 Hz, 1H), 2.01 (s, 3H).

<u>Computational observation</u>

The calculated ΔG is given for the energetically lowest conformers.

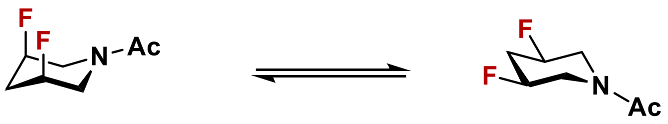

$13A_a$ **$13A_e$**

Solvent	ΔG(a→e)	ΔE_{elect}	ΔE_{hyperc}	ΔE_{steric}	μ_a	μ_e	Experimental
**DMSO**	+1.1	+14.7	+10.4	–23.6	+8.9	+4.2	axial
**$CHCl_3$**	+0.4	+18.5	+9.7	–27.6	+8.1	+3.8	axial
**gas phase**	–1.6	+21.1	+8.6	–30.8	5.8	2.8	-

All values are given in kcal/mol at 298 K.

1-(*cis*-3,5-Difluoropiperidin-1-yl)-2,2-dimethylpropan-1-one (14)

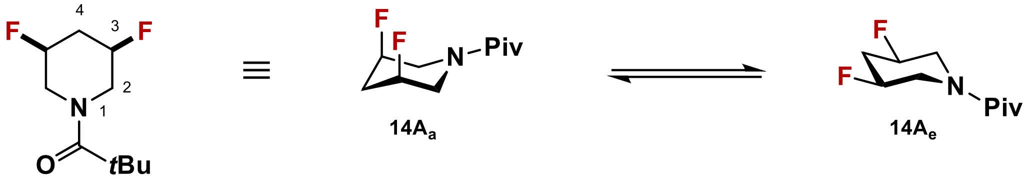

For this particular sample we conducted NMR experiments in deuterated solvents in order to investigate the effect of solvent's dipole moment on the orientation of the fluorine atoms.

The preparation of this compound is described in the previous section.

<u>Experimental observation</u>

In **$CDCl_3$**: The orientation of the fluorine atoms was assigned as equatorial due to the small values of ^3J(F,H_a). [**^3J(2-H_a,3-F) = 7.3 Hz, ^3J(4-H_a,3-F) = 8.0 Hz**]

^{1}H NMR (500 MHz, $CDCl_3$, 299 K) δ 4.63 (ddq, *J* = 46.3, 7.0, 3.5 Hz, 2H), 3.86 (ddd, *J* = 20.7, 13.5, 3.4 Hz, 2H), 3.75 (dt, *J* = 14.1, 7.3 Hz, 2H), 2.42 – 2.24 (m, 1H), 2.15 (dm, *J* = 8.0 Hz, 1H), 1.31 (s, 9H); **^{1}H{^{19}F} NMR** (500 MHz, $CDCl_3$, 299 K) δ 4.63 (tt, *J* = 7.0, 3.8 Hz, 2H), 3.86 (dm, *J* = 13.6 Hz, 2H), 3.75 (dd, *J* = 13.7, 6.8 Hz, 2H), 2.32 (dt, *J* = 13.6, 4.1 Hz, 1H), 2.15 (dtt, *J* = 13.7, 6.9, 0.9 Hz, 1H), 1.31 (s, 9H); **^{13}C NMR** (126 MHz, $CDCl_3$, 299 K) δ 177.74, 84.41 (d, *J* = 180.2 Hz), 48.34 (d, *J* = 25.3 Hz), 39.15, 35.94 (t, *J* = 20.6 Hz), 28.62; **^{13}C{sel-^{19}F at -183 ppm} NMR** (126 MHz, $CDCl_3$, 299 K) δ 177.74, 84.43, 48.34, 39.15, 35.94, 28.62; **^{19}F NMR** (470 MHz, $CDCl_3$, 299 K) δ -182.32 – -183.44 (m); **^{19}F{^{1}H} NMR** (470 MHz, $CDCl_3$, 299 K) δ -183.11.
ESI-MS: calculated $[C_{10}H_{17}NOF_2 + Na]^+$: 228.1170, found 228.1184.

In **DMSO**: The orientation of the fluorine atoms was assigned as axial due to the large values of ^3J(F,H_a). [**^3J(2-H_a,3-F) = 34.1 Hz, ^3J(4-H_a,3-F) = 38.5 Hz]**

^{1}H NMR (600 MHz, DMSO-d_6, 299 K) δ 4.79 (dqd, *J* = 46.6, 3.8, 1.9 Hz, 2H), 4.38 – 4.30 (m, 2H), 3.27 (dd, *J* = 34.1, 14.0 Hz, 2H), 2.19 – 2.15 (m, 1H overlaps with 4-H_a), 2.07 (tdt, *J* = 38.5, 15.3, 3.5 Hz, 1H overlaps with 4-H_e), 1.21 (s, 9H); **^{1}H{^{19}F} NMR** (600 MHz, DMSO-d_6, 299 K) δ 4.82 – 4.76 (m, 2H), 4.34 (dm, *J* = 14.9 Hz, 2H), 3.27 (d, *J* = 14.4 Hz, 2H), 2.17 (dtt, *J* = 15.2, 3.7, 1.8 Hz, 1H), 2.10 (dt, *J* = 15.3, 3.7 Hz, 1H), 1.21 (s, 9H).

Computational observation

The calculated ΔG is given for the energetically lowest conformers.

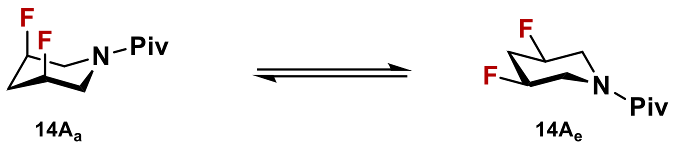

Solvent	ΔG(a→e)	ΔE_{elect}	ΔE_{hyperc}	ΔE_{steric}	μ_a	μ_e	Experimental
**DMSO**	+0.5	+23.6	+11.6	–34.5	8.4	4.2	axial
**$CHCl_3$**	–1.0	+17.2	+11.8	–29.2	7.6	3.8	equatorial
**gas phase**	–2.3	+7.6	+13.4	–23.2	5.2	2.8	-

All values are given in kcal/mol at 298 K.

tert-Butyl cis-3,5-difluoropiperidine-1-carboxylate (15)

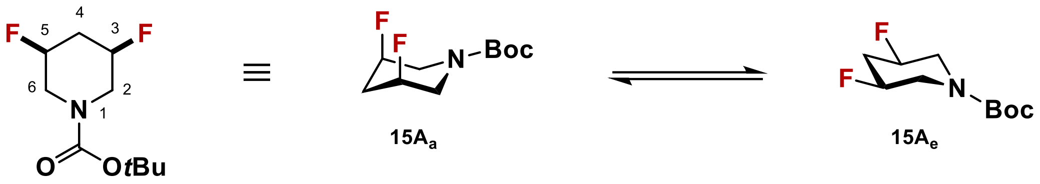

For this particular sample we conducted several computations and NMR experiments in different solvents in order to investigate the effect of solvent's dipole moment on the orientation of the fluorine atoms.

This compound was prepared following a recent literature procedure.[1]

Experimental observation

Broadening in NMR spectra was observed due to the dynamic behavior of the Boc group.

In **$CDCl_3$**: The orientation of the fluorine atoms was assigned as equatorial due to the small values of ^3J(F,H$_a$). [**^3J(4-H$_a$,3-F) = 12.5 Hz, ^3J(4-H$_a$,5-F) = 12.5 Hz**]

^{1}H NMR (600 MHz, $CDCl_3$, 299 K) δ 4.61 (d, *J* = 46.8 Hz, 2H), 3.71 – 3.54 (m, 4H), 2.21 (tdt, *J* = 25.2, 13.8, 4.1 Hz, 1H overlaps with 4-H$_a$), 2.12 (dm, *J* = 12.5 Hz, 1H overlaps with 4-H$_e$), 1.46 (s, 9H); **^{1}H{^{19}F} NMR** (600 MHz, $CDCl_3$, 299 K) δ 4.61 (s, 2H), 3.65 – 3.56 (m, 4H), 2.21 (dt, *J* = 13.9, 4.0 Hz, 1H), 2.12 (dt, *J* = 13.4, 6.6 Hz, 1H), 1.46 (s, 9H); **^{13}C NMR** (151 MHz, $CDCl_3$, 299 K) δ 154.99, 84.37 (d, *J* = 173.0 Hz), 80.75, 47.78 (bs), 46.70 (bs), 35.51 (t, *J* = 20.6 Hz), 28.42; **^{13}C{sel-^{19}F at -183 ppm} NMR** (151 MHz, $CDCl_3$, 299 K) δ 154.98, 84.37, 80.74, 47.69 (bs), 46.60 (bs), 35.51, 28.42; **^{19}F NMR** (564 MHz, $CDCl_3$, 299 K) δ -183.37 (bs); **^{19}F{^{1}H} NMR** (564 MHz, $CDCl_3$, 299 K) δ -183.36 (bs).

ESI-MS: calculated [$C_{10}H_{17}NO_2F_2$ +Na]$^+$: 244.1125, found 244.1125.

In **DMSO**: The orientation of the fluorine atoms was assigned as axial due to the large values of ^3J(F,H$_a$). [**^3J(2-H$_a$,3-F) = 33.8 Hz, ^3J(4-H$_a$,3-F) = 40.4 Hz, ^3J(4-H$_a$,5-F) = 40.4 Hz, ^3J(6-H$_a$,5-F) = 33.8 Hz**]

^{1}H NMR (600 MHz, DMSO-*d*$_6$, 299 K) δ 4.74 (d, *J* = 46.7 Hz, 2H), 4.04 (bs, 2H), 3.15 (tm, *J* = 33.8 Hz, 2H), 2.16 (ddddt, *J* = 15.9, 12.3, 10.2, 3.7, 1.7 Hz, 1H), 2.02 (tdt, *J* = 40.4, 15.5, 3.4 Hz,

1H), 1.40 (s, 9H); **^{1}H{^{19}F} NMR** (600 MHz, DMSO-d_6, 299 K) δ 4.74 (s, 2H), 4.04 (bs, 2H), 3.26 – 3.03 (m, 2H), 2.16 (dtt, J = 15.5, 3.8, 2.0 Hz, 1H), 2.02 (dt, J = 15.6, 3.5 Hz, 1H), 1.40 (s, 9H).

Computational observation

The calculated ΔG is given for the energetically lowest conformers.

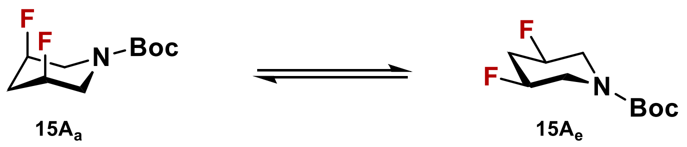

Solvent	ΔG(a→e)	ΔE_{elect}	ΔE_{hyperc}	ΔE_{steric}	μ_a	μ_e	Experimental
**DMSO**	+0.7	+30.4	+12.9	–42.5	6.5	3.3	axial
**$CHCl_3$**	–1.0	+30.0	+12.8	–42.7	5.9	3.1	equatorial
**gas phase**	–2.4	+36.9	+12.4	–51.3	4.2	2.4	-

All values are given in kcal/mol at 298 K.

1-(4-Methylpiperidin-1-yl)-2,2,2-trifluoromethylethan-1-one (16)

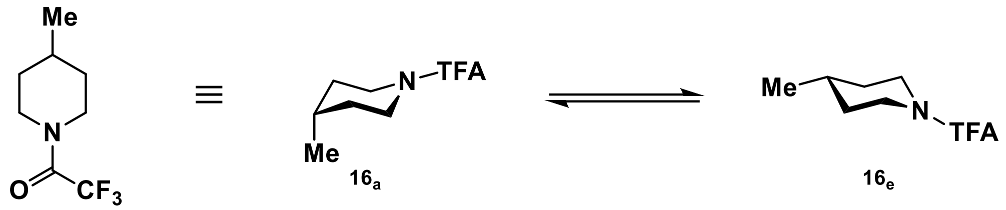

<u>Computational observation</u>

The calculated ΔG is given for the energetically lowest conformers.

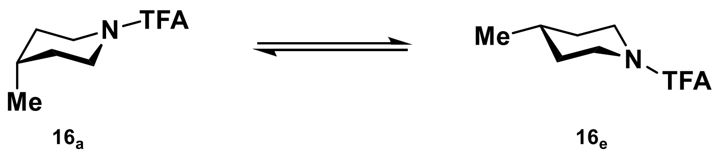

Solvent	$\Delta G(a \rightarrow e)$	ΔE_{elect}	ΔE_{hyperc}	ΔE_{steric}	$\mu_{a,a}$	$\mu_{e,e}$	Experimental
**DMSO**	–2.0	+4.7	–0.9	–5.4	6.8	6.8	-
**H_2O**	–1.9	+4.7	–0.9	–5.4	6.8	6.8	-
**CH_2Cl_2**	–1.8	+4.3	–1.0	–5.0	6.5	6.5	-
**$CHCl_3$**	–1.9	+1.8	–0.9	–2.6	6.3	6.3	-
**C_6H_6**	–2.0	+3.8	–1.1	–4.5	5.8	5.8	-
**gas phase**	–2.0	+0.6	–1.1	–1.2	4.9	4.8	-

All values are given in kcal/mol at 298 K.

1-(*cis*-3,5-Difluoro-*trans*-4-methylpiperidin-1-yl)-2,2,2-trifluoromethylethan-1-one (17)

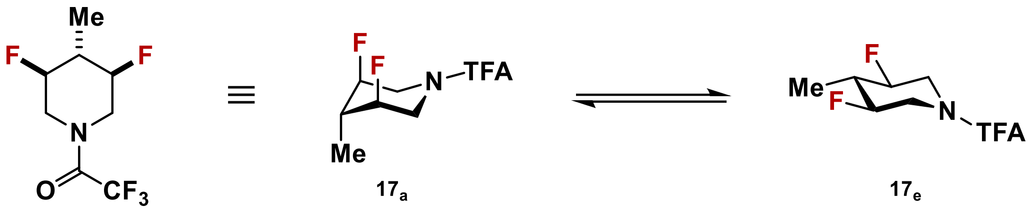

17a   17e

Computational observation

The calculated ΔG is given for the energetically lowest conformers.

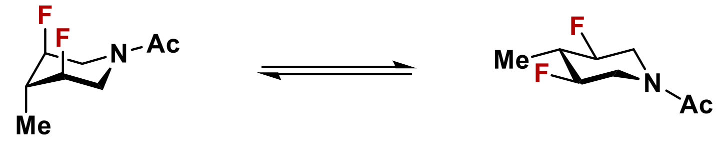

17a   17e

Solvent	ΔG(a→e)	ΔE_{elect}	ΔE_{hyperc}	ΔE_{steric}	$\mu_{a,a}$	$\mu_{e,e}$	Experimental
**DMSO**	+0.5	+2.7	+9.1	–11.2	9.8	3.5	-
**H_2O**	+0.6	+2.7	+9.1	–11.3	9.9	3.5	-
**CH_2Cl_2**	–0.0	+0.6	+8.9	–9.4	9.5	3.4	-
**$CHCl_3$**	–0.4	+0.5	+8.8	–9.6	9.1	3.2	-
**C_6H_6**	–2.0	–0.7	+8.6	–9.1	8.2	2.9	-
**gas phase**	–2.9	+3.8	+8.6	–15.1	6.7	2.4	-

All values are given in kcal/mol at 298 K.

References

1. Z. Nairoukh, M. Wollenburg, C. Schlepphorst, K. Bergander, F. Glorius, *Nat. Chem.* **2019**, *11*, 264.
2. a) F. Neese, *WIREs Comput. Mol. Sci.* **2012**, *2*, 73. b) F. Neese, *WIREs Comput. Mol. Sci.* **2018**, *8*, e1327.
3. Jmol: an open-source Java viewer for chemical structures in 3D. http://www.jmol.org/.
4. Y. Zhao, D. G. Truhlar, *Theor. Chem. Acc.* **2008**, *120*, 215.
5. F. Weigend, R. Ahlrichs, *Phys. Chem. Chem. Phys.* **2005**, *7*, 3297.
6. a) V. Barone, M. Cossi, *J. Phys. Chem. A* **1998**, *102*, 1995. b) M. Cossi, N. Rega, G. Scalmani, V. Barone, *J. Comput. Chem.* **2003**, *24*, 669.
7. E. D. Glendening, A. E. Reed, J. E. Carpenter, F. Weinhold, *NBO Version 3.1*.

Computational Data

Comp. No.	Solvent	Electronic Energy [Eh]	ZPVE [Eh]	Thermal Correction [Eh]	Enthalpy Correction [Eh]	Entropy Correction [Eh]
**1A_a**	Gas Phase	-801.6259459	0.1689493	0.01133137	0.00094421	-0.04919698
**1A_a**	Toluene	-801.634133	0.16864346	0.01132307	0.00094421	-0.04913137
**1A_e**	Gas Phase	-801.6263801	0.16880073	0.01140211	0.00094421	-0.04938804
**1A_e**	Toluene	-801.6338477	0.16851314	0.01140735	0.00094421	-0.04938029
**1B_a**	Gas Phase	-351.530378	0.16835041	0.00624385	0.00094421	-0.03711602
**1B_a**	Water	-351.6326598	0.1682198	0.00631765	0.00094421	-0.03732519
**1B_e**	Gas Phase	-351.5221225	0.16801694	0.00635132	0.00094421	-0.03742287
**1B_e**	Water	-351.6295947	0.16811267	0.00635205	0.00094421	-0.03743271
**1C_ae**	Gas Phase	-351.1597787	0.15296809	0.00622141	0.00094421	-0.03708196
**1C_ae**	Water	-351.1704709	0.15269654	0.00619851	0.00094421	-0.03702969
**1C_aa**	Gas Phase	-351.1619248	0.15313089	0.00621352	0.00094421	-0.0370766
**1C_aa**	Water	-351.1708822	0.15271469	0.00624126	0.00094421	-0.03713985
**1C_ee**	Gas Phase	-351.1618747	0.15305333	0.00622065	0.00094421	-0.03710172
**1C_ee**	Water	-351.1706597	0.1526886	0.00621756	0.00094421	-0.03709072
**1C_ea**	Gas Phase	-351.1605979	0.1529366	0.00626151	0.00094421	-0.03719315
**1C_ea**	Water	-351.1703391	0.15271284	0.00626188	0.00094421	-0.03719523
**2A_a**	Gas Phase	-900.8816604	0.16101833	0.01211917	0.00094421	-0.05100981
**2A_a**	Benzene	-900.892217	0.16080437	0.01211744	0.00094421	-0.0509751
**2A_a**	Dichloromethane	-900.9003424	0.16047975	0.01129392	0.00094421	-0.04922862
**2A_a**	Chloroform	-900.8978157	0.16066426	0.01212913	0.00094421	-0.05099004
**2A_a**	DMSO	-900.9029415	0.16055378	0.01212662	0.00094421	-0.05096904
**2A_a**	Water	-900.9032053	0.16053968	0.01212936	0.00094421	-0.0509735
**2A_e**	Gas Phase	-900.8836352	0.16096724	0.01222411	0.00094421	-0.05132188
**2A_e**	Benzene	-900.8919635	0.16070132	0.0122501	0.00094421	-0.05139424
**2A_e**	Dichloromethane	-900.8977977	0.16060495	0.01222449	0.00094421	-0.05128684
**2A_e**	Chloroform	-900.8960406	0.1606235	0.0122435	0.00094421	-0.05134876
**2A_e**	DMSO	-900.8995407	0.16053115	0.01223756	0.00094421	-0.05131627
**2A_e**	Water	-900.8997162	0.16052755	0.01223589	0.00094421	-0.05131139

Comp. No.	Solvent	Electronic Energy [Eh]	ZPVE [Eh]	Thermal Correction [Eh]	Enthalpy Correction [Eh]	Entropy Correction [Eh]
**2C_ee**	Gas Phase	-450.4222648	0.14542198	0.00696804	0.00094421	-0.03917046
**2C_ee**	Benzene	-450.4283847	0.14527285	0.00695724	0.00094421	-0.03913491
**2C_ee**	Dichloromethane	-450.432526	0.1451169	0.00698024	0.00094421	-0.03919794
**2C_ee**	Chloroform	-450.4313016	0.14517539	0.00697341	0.00094421	-0.03918554
**2C_ee**	DMSO	-450.4337225	0.14508166	0.0069824	0.00094421	-0.03919916
**2C_ee**	Water	-450.433842	0.14508354	0.00698135	0.00094421	-0.03919725
**2C_ea**	Gas Phase	-450.4209177	0.14523881	0.00703444	0.00094421	-0.03931768
**2C_ea**	Benzene	-450.4275901	0.14519336	0.0070261	0.00094421	-0.03930381
**2C_ea**	Dichloromethane	-450.4321603	0.14515023	0.00702779	0.00094421	-0.03930885
**2C_ea**	Chloroform	-450.4307934	0.1451697	0.00702407	0.00094421	-0.03929919
**2C_ea**	DMSO	-450.4335054	0.14521798	0.00701602	0.00094421	-0.0392816
**2C_ea**	Water	-450.4336407	0.14522606	0.00701737	0.00094421	-0.03928451
**2C_ae**	Gas Phase	-450.4167679	0.14513902	0.00697708	0.00094421	-0.03910142
**2C_ae**	Benzene	-450.4252986	0.14509296	0.00694825	0.00094421	-0.03903571
**2C_ae**	Dichloromethane	-450.4317693	0.14505474	0.00693099	0.00094421	-0.03899219
**2C_ae**	Chloroform	-450.42977	0.14506082	0.00693872	0.00094421	-0.03901428
**2C_ae**	DMSO	-450.4337855	0.14506134	0.0069274	0.00094421	-0.03898338
**2C_ae**	Water	-450.4339923	0.14507976	0.00692276	0.00094421	-0.03897331
**2C_aa**	Gas Phase	-450.4218862	0.14546089	0.00694339	0.00094421	-0.03906333
**2C_aa**	Benzene	-450.4287983	0.14529905	0.00695349	0.00094421	-0.03907755
**2C_aa**	Dichloromethane	-450.4336555	0.14519819	0.00695935	0.00094421	-0.03908468
**2C_aa**	Chloroform	-450.432188	0.14522116	0.00695957	0.00094421	-0.03908697
**2C_aa**	DMSO	-450.4351093	0.14516262	0.00696186	0.00094421	-0.03908754
**2C_aa**	Water	-450.435256	0.14515752	0.00696235	0.00094421	-0.03908844
**3A_a**	Gas Phase	-840.9398689	0.19700024	0.0128136	0.00094421	-0.05224187
**3A_a**	Chloroform	-840.9516605	0.19653986	0.01282299	0.00094421	-0.05222229
**3A_e**	Gas Phase	-840.9373161	0.19726744	0.01279345	0.00094421	-0.05222211
**3A_e**	Chloroform	-840.9481592	0.19676352	0.01281713	0.00094421	-0.05223844
**3B_a**	Gas Phase	-390.8453896	0.19638613	0.00769226	0.00094421	-0.04024415
**3B_a**	Water	-390.9460341	0.19626065	0.00774314	0.00094421	-0.04039481

Comp. No.	Solvent	Electronic Energy [E_h]	ZPVE [E_h]	Thermal Correction [E_h]	Enthalpy Correction [E_h]	Entropy Correction [E_h]
**3B_e**	Gas Phase	-390.8354585	0.19641687	0.00770118	0.00094421	-0.04028583
**3B_e**	Water	-390.9406814	0.19661467	0.00767359	0.00094421	-0.04023764
**3C_ae**	Gas Phase	-390.473243	0.18102694	0.00766429	0.00094421	-0.04019317
**3C_ae**	Water	-390.4838329	0.18069321	0.00764032	0.00094421	-0.04013868
**3C_aa**	Gas Phase	-390.4756426	0.18122548	0.00764256	0.00094421	-0.04016537
**3C_aa**	Water	-390.4844915	0.18081529	0.00765648	0.00094421	-0.04019514
**3C_ee**	Gas Phase	-390.4726544	0.1814969	0.00759222	0.00094421	-0.04003724
**3C_ee**	Water	-390.4813313	0.1810497	0.00757865	0.00094421	-0.03999378
**3C_ea**	Gas Phase	-390.4715489	0.18140172	0.00761779	0.00094421	-0.04008927
**3C_ea**	Water	-390.4810823	0.18105404	0.00761586	0.00094421	-0.04008262
**4A_a**	Gas Phase	-840.9380827	0.19724179	0.01264227	0.00094421	-0.05166772
**4A_a**	Chloroform	-840.9499255	0.19678333	0.01263207	0.00094421	-0.05162098
**4A_e**	Gas Phase	-840.9399186	0.19683157	0.01289392	0.00094421	-0.05241024
**4A_e**	Chloroform	-840.9508543	0.19638561	0.0129133	0.00094421	-0.05243683
**4B_a**	Gas Phase	-390.8426546	0.19648425	0.00763904	0.00094421	-0.04013884
**4B_a**	Water	-390.9429668	0.196368	0.00766209	0.00094421	-0.04021186
**4B_e**	Gas Phase	-390.8373315	0.19610864	0.00778239	0.00094421	-0.04058233
**4B_e**	Water	-390.9430543	0.19614016	0.00779374	0.00094421	-0.04062399
**4C_ae**	Gas Phase	-390.4714529	0.18128362	0.00754708	0.00094421	-0.03992141
**4C_ae**	Water	-390.480976	0.18071351	0.00758811	0.00094421	-0.04002575
**4C_aa**	Gas Phase	-390.4731709	0.18141371	0.0075465	0.00094421	-0.03993877
**4C_aa**	Water	-390.4819939	0.18107617	0.00750854	0.00094421	-0.03983668
**4C_ee**	Gas Phase	-390.4755312	0.18104879	0.00765437	0.00094421	-0.04026993
**4C_ee**	Water	-390.4844743	0.18059586	0.00765259	0.00094421	-0.04026001
**4C_ea**	Gas Phase	-390.474424	0.18094129	0.00770719	0.00094421	-0.04039216
**4C_ea**	Water	-390.4843432	0.18061678	0.00770578	0.00094421	-0.04038857
**5A_a**	Gas Phase	-840.9331314	0.19744122	0.01254499	0.00094421	-0.05139452
**5A_a**	Chloroform	-840.9447066	0.19690447	0.01258967	0.00094421	-0.05148371
**5A_e**	Gas Phase	-840.9391887	0.19713343	0.01281675	0.00094421	-0.05212767
**5A_e**	Chloroform	-840.9500709	0.19671024	0.01279687	0.00094421	-0.05209453

Comp. No.	Solvent	Electronic Energy [Eh]	ZPVE [Eh]	Thermal Correction [Eh]	Enthalpy Correction [Eh]	Entropy Correction [Eh]
**5B_a**	Gas Phase	-390.8499078	0.19629763	0.00767043	0.00094421	-0.04016662
**5B_a**	Water	-390.9473783	0.19621357	0.00775075	0.00094421	-0.04038723
**5B_e**	Gas Phase	-390.8400293	0.19632459	0.00768476	0.00094421	-0.04023
**5B_e**	Water	-390.9427392	0.19660802	0.00756538	0.00094421	-0.03994581
**5C_ae**	Gas Phase	-390.4756708	0.18101751	0.00763667	0.00094421	-0.04012766
**5C_ae**	Water	-390.485658	0.18059096	0.00763065	0.00094421	-0.04011088
**5C_aa**	Gas Phase	-390.4771249	0.18114373	0.00765618	0.00094421	-0.04018013
**5C_aa**	Water	-390.4858376	0.18066476	0.00766511	0.00094421	-0.04019251
**5C_ee**	Gas Phase	-390.4742597	0.18133165	0.00760285	0.00094421	-0.04003301
**5C_ee**	Water	-390.482848	0.18086963	0.007604	0.00094421	-0.04003428
**5C_ea**	Gas Phase	-390.4733895	0.18135162	0.00760698	0.00094421	-0.04005578
**5C_ea**	Water	-390.4828226	0.18094322	0.00761845	0.00094421	-0.04008603
**6A_a**	Gas Phase	-840.9338674	0.19712694	0.01257976	0.00094421	-0.05145692
**6A_a**	Chloroform	-840.9451703	0.19665764	0.01259902	0.00094421	-0.05148391
**6A_e**	Gas Phase	-840.93929	0.19707249	0.0128277	0.00094421	-0.05219499
**6A_e**	Chloroform	-840.9500191	0.19663466	0.01281918	0.00094421	-0.05214099
**6B_a**	Gas Phase	-390.8498803	0.19617696	0.00765589	0.00094421	-0.04018899
**6B_a**	Water	-390.9478147	0.19586991	0.0077484	0.00094421	-0.04043313
**6B_e**	Gas Phase	-390.8391004	0.19624634	0.00769377	0.00094421	-0.04029724
**6B_e**	Water	-390.9426872	0.19626881	0.00769224	0.00094421	-0.04030439
**6C_ae**	Gas Phase	-390.4758832	0.18091855	0.00762295	0.00094421	-0.04014556
**6C_ae**	Water	-390.4866601	0.18062266	0.00755101	0.00094421	-0.03996809
**6C_aa**	Gas Phase	-390.4777825	0.18104477	0.00762234	0.00094421	-0.04015211
**6C_aa**	Water	-390.4867059	0.18063117	0.00763115	0.00094421	-0.04017193
**6C_ee**	Gas Phase	-390.474037	0.18124917	0.00760484	0.00094421	-0.04009064
**6C_ee**	Water	-390.4827291	0.18082888	0.00759912	0.00094421	-0.04007788
**6C_ea**	Gas Phase	-390.4732172	0.18126088	0.00760814	0.00094421	-0.04011021
**6C_ea**	Water	-390.4830227	0.1808648	0.00762354	0.00094421	-0.04015494
**7A_a**	Gas Phase	-1138.728248	0.1741844	0.01455138	0.00094421	-0.05608935
**7A_a**	Chloroform	-1138.742016	0.17371648	0.01456243	0.00094421	-0.05609464

Comp. No.	Solvent	Electronic Energy [Eh]	ZPVE [Eh]	Thermal Correction [Eh]	Enthalpy Correction [Eh]	Entropy Correction [Eh]
**7A_e**	Gas Phase	-1138.737738	0.17442237	0.01474117	0.00094421	-0.05662169
**7A_e**	Chloroform	-1138.748694	0.17381775	0.01480929	0.00094421	-0.05676251
**7B_a**	Gas Phase	-688.6338359	0.17345359	0.00957155	0.00094421	-0.0452195
**7B_a**	Water	-688.7390937	0.17305183	0.00977784	0.00094421	-0.04577268
**7B_e**	Gas Phase	-688.6215475	0.17339225	0.00959307	0.00094421	-0.04514972
**7B_e**	Water	-688.7320217	0.17380057	0.0095458	0.00094421	-0.04507791
**7C_ae**	Gas Phase	-688.2796547	0.1585795	0.00951651	0.00094421	-0.04513425
**7C_ae**	Water	-688.2929145	0.1581745	0.00951731	0.00094421	-0.04512443
**7C_aa**	Gas Phase	-688.2813934	0.15868262	0.00949823	0.00094421	-0.04510205
**7C_aa**	Water	-688.2931305	0.15815529	0.009526	0.00094421	-0.04515787
**7C_ee**	Gas Phase	-688.279636	0.15889365	0.00950529	0.00094421	-0.04498004
**7C_ee**	Water	-688.2899473	0.15823823	0.00954003	0.00094421	-0.0450408
**7C_ea**	Gas Phase	-688.2762702	0.15869289	0.00955005	0.00094421	-0.04505896
**7C_ea**	Water	-688.2891518	0.15828512	0.00954313	0.00094421	-0.04504776
**8A_a**	Gas Phase	-880.2507371	0.22539288	0.01411982	0.00094421	-0.05460079
**8A_a**	Chloroform	-880.2623052	0.22488707	0.01412995	0.00094421	-0.05462755
**8A_e**	Gas Phase	-880.2502769	0.22545207	0.01420171	0.00094421	-0.0549507
**8A_e**	Chloroform	-880.2610386	0.22490327	0.01422352	0.00094421	-0.05497025
**8B_a**	Gas Phase	-430.1567416	0.22480191	0.0089966	0.00094421	-0.04294869
**8B_a**	Water	-430.2552905	0.22463158	0.00906713	0.00094421	-0.04313992
**8B_e**	Gas Phase	-430.149895	0.22473285	0.00904227	0.00094421	-0.04309384
**8B_e**	Water	-430.2535374	0.22474703	0.00907088	0.00094421	-0.04318461
**8C_ae**	Gas Phase	-429.7840955	0.20952604	0.00894273	0.00094421	-0.04281757
**8C_ae**	Water	-429.7935556	0.20901332	0.00897776	0.00094421	-0.04290965
**8C_aa**	Gas Phase	-429.7859444	0.20969246	0.00892838	0.00094421	-0.04281282
**8C_aa**	Water	-429.7945978	0.20920188	0.00894512	0.00094421	-0.04284581
**8C_ee**	Gas Phase	-429.7853935	0.20972313	0.00893673	0.00094421	-0.04284051
**8C_ee**	Water	-429.7941218	0.20920212	0.00894845	0.00094421	-0.04287023
**8C_ea**	Gas Phase	-429.7845618	0.20966686	0.00895802	0.00094421	-0.04288716
**8C_ea**	Water	-429.7942459	0.20922986	0.00898115	0.00094421	-0.04294813

Comp. No.	Solvent	Electronic Energy [Eh]	ZPVE [Eh]	Thermal Correction [Eh]	Enthalpy Correction [Eh]	Entropy Correction [Eh]
**9A_a**	Gas Phase	-940.1948588	0.18916859	0.01366254	0.00094421	-0.0541192
**9A_a**	Chloroform	-940.2104416	0.18883027	0.01363615	0.00094421	-0.05401677
**9A_e**	Gas Phase	-940.1950964	0.18950613	0.01361881	0.00094421	-0.05405058
**9A_e**	Chloroform	-940.2069659	0.18897225	0.01366124	0.00094421	-0.0541473
**9B_a**	Gas Phase	-490.0979344	0.18856093	0.00844122	0.00094421	-0.04214427
**9B_a**	Water	-490.2039846	0.18870557	0.00849228	0.00094421	-0.04227794
**9B_e**	Gas Phase	-490.0826093	0.18850663	0.0085208	0.00094421	-0.04238395
**9B_e**	Water	-490.1957649	0.18866672	0.00854106	0.00094421	-0.04245688
**9C_ae**	Gas Phase	-489.7300861	0.17331171	0.00844661	0.00094421	-0.04215687
**9C_ae**	Water	-489.7466928	0.1731928	0.00836933	0.00094421	-0.04196661
**9C_aa**	Gas Phase	-489.735469	0.17365755	0.00839375	0.00094421	-0.04208115
**9C_aa**	Water	-489.7482402	0.17330843	0.00841727	0.00094421	-0.04212243
**9C_ee**	Gas Phase	-489.7330015	0.17391362	0.00836761	0.00094421	-0.04202201
**9C_ee**	Water	-489.7439577	0.17342684	0.00839712	0.00094421	-0.04208546
**9C_ea**	Gas Phase	-489.7319796	0.1737738	0.0084125	0.00094421	-0.04211834
**9C_ea**	Water	-489.7441242	0.17341379	0.00845053	0.00094421	-0.04222066
**10A_a**	Gas Phase	-801.6284032	0.16896879	0.01137663	0.00094421	-0.04946828
**10A_a**	Chloroform	-801.6395066	0.16850184	0.01137936	0.00094421	-0.0494352
**10A_e**	Gas Phase	-801.6273874	0.16887529	0.01133477	0.00094421	-0.04927865
**10A_e**	Chloroform	-801.6390035	0.16851712	0.01135683	0.00094421	-0.04930666
**10B_a**	Gas Phase	-351.5301748	0.16823669	0.00627341	0.00094421	-0.03719035
**10B_a**	Water	-351.6347746	0.16811316	0.00637208	0.00094421	-0.03749039
**10B_e**	Gas Phase	-351.5250141	0.16804432	0.00632918	0.00094421	-0.03737591
**10B_e**	Water	-351.6334149	0.16823859	0.00629825	0.00094421	-0.0373068
**10C_ae**	Gas Phase	-351.1625006	0.15325032	0.0061813	0.00094421	-0.03700131
**10C_ae**	Water	-351.1713795	0.15276061	0.00618994	0.00094421	-0.03701612
**10C_aa**	Gas Phase	-351.1610358	0.15307855	0.00620771	0.00094421	-0.03705125
**10C_aa**	Water	-351.1708619	0.15269659	0.006236	0.00094421	-0.03712181
**10C_ee**	Gas Phase	-351.1623525	0.15304856	0.00621035	0.00094421	-0.03709128
**10C_ee**	Water	-351.1714528	0.15271887	0.00620835	0.00094421	-0.03708517

Comp. No.	Solvent	Electronic Energy [Eh]	ZPVE [Eh]	Thermal Correction [Eh]	Enthalpy Correction [Eh]	Entropy Correction [Eh]
**10C_ea**	Gas Phase	-351.1617011	0.15298624	0.00623675	0.00094421	-0.0371581
**10C_ea**	Water	-351.1718592	0.1527355	0.00624111	0.00094421	-0.03716634
**11A_a**	Gas Phase	-840.9422407	0.19702256	0.01289005	0.00094421	-0.05248776
**11A_a**	Chloroform	-840.9530003	0.19638325	0.01295953	0.00094421	-0.05259982
**11A_e**	Gas Phase	-840.9401224	0.19729802	0.01269629	0.00094421	-0.05183244
**11A_e**	Chloroform	-840.9514582	0.19705445	0.01268718	0.00094421	-0.05179894
**11B_a**	Gas Phase	-390.8455476	0.19638472	0.00772018	0.00094421	-0.04030837
**11B_a**	Water	-390.947998	0.19640458	0.00773805	0.00094421	-0.04037977
**11B_e**	Gas Phase	-390.8394488	0.19644097	0.00768542	0.00094421	-0.04026069
**11B_e**	Water	-390.9445031	0.19652299	0.00772373	0.00094421	-0.04037976
**11C_ae**	Gas Phase	-390.4758974	0.18130604	0.00763625	0.00094421	-0.04013822
**11C_ae**	Water	-390.4846589	0.18076428	0.00764669	0.00094421	-0.04015891
**11C_aa**	Gas Phase	-390.4746895	0.18117078	0.00766404	0.00094421	-0.04019458
**11C_aa**	Water	-390.484392	0.18072055	0.00769225	0.00094421	-0.04026321
**11C_ee**	Gas Phase	-390.4742425	0.18137817	0.00759151	0.00094421	-0.04003891
**11C_ee**	Water	-390.4829239	0.18084404	0.00761397	0.00094421	-0.04008927
**11C_ea**	Gas Phase	-390.4736172	0.18140477	0.00759064	0.00094421	-0.04003769
**11C_ea**	Water	-390.4833872	0.1810943	0.00759572	0.00094421	-0.04005261
**12A_a**	Gas Phase	-840.9408223	0.19724261	0.01275249	0.00094421	-0.05207009
**12A_a**	Chloroform	-840.9516784	0.19671955	0.0127605	0.00094421	-0.05210527
**12A_e**	Gas Phase	-840.9351937	0.19721311	0.01260137	0.00094421	-0.05161401
**12A_e**	Chloroform	-840.9461902	0.19679629	0.01264337	0.00094421	-0.05177724
**12B_a**	Gas Phase	-390.8460162	0.1964402	0.00761188	0.00094421	-0.0400317
**12B_a**	Water	-390.9464266	0.19640999	0.00763112	0.00094421	-0.04010392
**12B_e**	Gas Phase	-390.8445704	0.19589943	0.00776609	0.00094421	-0.04053595
**12B_e**	Water	-390.9484908	0.19602502	0.00771969	0.00094421	-0.04040068
**12C_ae**	Gas Phase	-390.4741778	0.18142212	0.00754636	0.00094421	-0.03990905
**12C_ae**	Water	-390.4828002	0.18093447	0.00755173	0.00094421	-0.03992208
**12C_aa**	Gas Phase	-390.4729092	0.18134933	0.00755644	0.00094421	-0.03993927
**12C_aa**	Water	-390.4825491	0.18095488	0.00755639	0.00094421	-0.03994456

Comp. No.	Solvent	Electronic Energy [E_h]	ZPVE [E_h]	Thermal Correction [E_h]	Enthalpy Correction [E_h]	Entropy Correction [E_h]
**12C_ee**	Gas Phase	-390.478286	0.18098152	0.00762949	0.00094421	-0.04022217
**12C_ee**	Water	-390.487877	0.18054816	0.0076358	0.00094421	-0.04023338
**12C_ea**	Gas Phase	-390.4773269	0.180909	0.00765765	0.00094421	-0.04029172
**12C_ea**	Water	-390.4873876	0.18060441	0.00765247	0.00094421	-0.04027854
**13_e**	Gas Phase	-603.0972636	0.1835471	0.01033272	0.00094421	-0.04693444
**13_e**	Chloroform	-603.1117176	0.18336318	0.01030775	0.00094421	-0.04681252
**13_e**	DMSO	-603.1159209	0.18333834	0.01028599	0.00094421	-0.04672462
**13_a**	Gas Phase	-603.0955554	0.18373162	0.01012498	0.00094421	-0.04614494
**13_a**	Chloroform	-603.1128621	0.18346349	0.01018112	0.00094421	-0.04629832
**13_a**	DMSO	-603.1182766	0.18349889	0.01009694	0.00094421	-0.04607357
**14_a**	Gas Phase	-721.0227066	0.26909697	0.0141355	0.00094421	-0.05425624
**14_a**	Chloroform	-721.0386691	0.26890454	0.01401152	0.00094421	-0.05391373
**14_a**	DMSO	-721.043748	0.26864042	0.01410023	0.00094421	-0.05408482
**14_e**	Gas Phase	-721.0261475	0.26900742	0.01424383	0.00094421	-0.05457529
**14_e**	Chloroform	-721.0389444	0.26838451	0.01440748	0.00094421	-0.05514869
**14_e**	DMSO	-721.0427078	0.26816454	0.01356182	0.00094421	-0.05320403
**15_e**	Gas Phase	-796.2833106	0.2733248	0.01528841	0.00094421	-0.05702432
**15_e**	Chloroform	-796.2947864	0.27247927	0.0154523	0.00094421	-0.05765734
**15_e**	DMSO	-796.2980458	0.27220275	0.01458901	0.00094421	-0.05553863
**15_a**	Gas Phase	-796.2803916	0.27360263	0.01500503	0.00094421	-0.05612973
**15_a**	Chloroform	-796.294654	0.2729218	0.01508514	0.00094421	-0.05634949
**15_a**	DMSO	-796.2990611	0.27267934	0.01516926	0.00094421	-0.05663547

Comp. No.	Solvent	Electronic Energy [E_h]	ZPVE [E_h]	Thermal Correction [E_h]	Enthalpy Correction [E_h]	Entropy Correction [E_h]
**16_a**	Gas Phase	-741.6777787	0.20495266	0.01195644	0.00094421	-0.05024991
**16_a**	Benzene	-741.6837267	0.20467546	0.01192003	0.00094421	-0.05013012
**16_a**	Dichloromethane	-741.6881253	0.20427648	0.01200668	0.00094421	-0.05032388
**16_a**	Chloroform	-741.6867806	0.20442826	0.01196373	0.00094421	-0.05021864
**16_a**	DMSO	-741.68948	0.20430079	0.01194774	0.00094421	-0.0501768
**16_a**	Water	-741.6896129	0.20424224	0.01194608	0.00094421	-0.05017117
**16_e**	Gas Phase	-741.6804458	0.20459883	0.01202547	0.00094421	-0.05043681
**16_e**	Benzene	-741.686371	0.20426839	0.01201741	0.00094421	-0.05038409
**16_e**	Dichloromethane	-741.6907413	0.20400414	0.01202506	0.00094421	-0.05036565
**16_e**	Chloroform	-741.6894009	0.20408999	0.01201979	0.00094421	-0.05036716
**16_e**	DMSO	-741.6920886	0.20392426	0.01203885	0.00094421	-0.05039664
**16_e**	Water	-741.6922254	0.20392097	0.01203781	0.00094421	-0.05039309
**17_a**	Gas Phase	-940.1937221	0.18934864	0.01356321	0.00094421	-0.05388173
**17_a**	Benzene	-940.2042222	0.18902016	0.01269026	0.00094421	-0.051956
**17_a**	Dichloromethane	-940.212114	0.18884457	0.01358664	0.00094421	-0.0538402
**17_a**	Chloroform	-940.2096648	0.1889022	0.01357705	0.00094421	-0.05385483
**17_a**	DMSO	-940.2144477	0.18882597	0.01353705	0.00094421	-0.05368567
**17_a**	Water	-940.2147229	0.18880469	0.01354923	0.00094421	-0.05376113
**17_e**	Gas Phase	-940.1980607	0.18916465	0.01367332	0.00094421	-0.05412402
**17_e**	Benzene	-940.2059744	0.18885374	0.01370735	0.00094421	-0.0541995
**17_e**	Dichloromethane	-940.2115064	0.18862292	0.01372987	0.00094421	-0.05424675
**17_e**	Chloroform	-940.2098409	0.18869868	0.01372265	0.00094421	-0.05423674
**17_e**	DMSO	-940.2131561	0.18865707	0.01369387	0.00094421	-0.05414439
**17_e**	Water	-940.2133225	0.18861600	0.01370394	0.00094421	-0.05417927

1A_a, Gas Phase

C	-5.32148868182699	2.09878327766517	0.22079697168556
C	-5.23346030419343	0.87802802348472	-0.68402663354220
C	-4.10695217206062	2.99348629428958	0.06145065498225
F	-5.39195216243847	1.66011122938655	1.53279694706958
H	-6.24366217325913	2.64162723360519	0.00670115144496
C	-2.82285639685548	2.19881356943477	0.27639690831311
H	-4.17688895881526	3.82839340301073	0.75838257719594
H	-4.12581889957939	3.40996537718733	-0.95036864039914
C	-2.79628009052503	0.96300075510301	-0.61556007377435
H	-2.75290511020031	1.88840540830251	1.31971514979651
H	-1.95242606041206	2.81935611244344	0.06006615862235
N	-4.00235804653050	0.16414611228502	-0.43394670873968
H	-1.93843379910639	0.33460593553143	-0.39816117676228
H	-2.74849333062755	1.26404525442352	-1.66757047459166
H	-5.24625009022098	1.21289802781311	-1.72631470094918
H	-6.09475344395353	0.24057737759436	-0.52619848954207
C	-3.89375569705770	-1.07479993372055	0.08794215198142
O	-2.85834431881585	-1.59107730834543	0.42036182066037
C	-5.19301631992636	-1.91760203178182	0.21720124928083
F	-5.81406740535775	-2.02788720170980	-0.96624958185170
F	-6.05749756436130	-1.38097761712174	1.08307346118588
F	-4.89787897387592	-3.13181929888108	0.63490127793350

1A_a, Toluene

C	-5.32300254625423	2.10270935505912	0.21368983007350
C	-5.23146809528845	0.87644659542880	-0.68038463706348
C	-4.10753510091177	2.99483601679528	0.06276139530000
F	-5.40725397097791	1.66791689639119	1.53347735232627
H	-6.24306688721223	2.64315465586453	-0.00922597887165
C	-2.82244125487348	2.20250043898443	0.27936510195647
H	-4.18035714770940	3.82916013079274	0.76015666499297
H	-4.12451183035151	3.41150876021881	-0.94850110906458
C	-2.79348818725388	0.96451463572913	-0.60856645631452
H	-2.74604896185516	1.89723658363215	1.32397007655331
H	-1.95424425284061	2.82306092068899	0.05549901265756
N	-3.99940771525307	0.16270557597211	-0.41673827318014
H	-1.93038254980908	0.34289604300652	-0.39525463898342
H	-2.75681866976669	1.26120269731702	-1.66126303369408
H	-5.23339350203494	1.20456151677494	-1.72364857260718
H	-6.09572277671212	0.24276091191446	-0.52545781822248
C	-3.89358087783506	-1.07848088838737	0.07705177239550
O	-2.85635732486096	-1.61543684086940	0.39129484745722
C	-5.19511497701802	-1.91611644417140	0.21624186065084
F	-5.82848726666876	-2.02861869653257	-0.95842763888079
F	-6.04889652053747	-1.38220446741847	1.09380693893491
F	-4.89795958397519	-3.13423439719099	0.63154330358377

1A_e, Gas Phase

C	-5.99242294614089	1.71215752892824	-1.28228139793338
C	-5.61857173331416	0.69399871090271	-0.21169802739316
C	-4.90975613298309	2.75888411102150	-1.44897143584107
F	-7.17265666057111	2.31821409798580	-0.89691245498612
H	-6.17647424186008	1.19043421941482	-2.22602238072229
C	-3.55768178694023	2.09807873627972	-1.70835832763652
H	-4.86562113727813	3.34406273162187	-0.52570739311305
H	-5.17639379075101	3.43961994260653	-2.25726041927700
C	-3.24613017926745	1.06311466664842	-0.63460650344428
H	-2.76916135003678	2.85126605729504	-1.72715310733123
H	-3.55527557486970	1.60557633107947	-2.68364462446297
N	-4.33761224140014	0.10179752500004	-0.53466330762011
H	-3.13405861488630	1.55470993464325	0.33692198844767
H	-2.33233179401556	0.51976602543548	-0.85310955636612
H	-6.39527225537491	-0.05591848396208	-0.12287030868174
H	-5.53648629121300	1.21219871408308	0.74878283844918
C	-4.08960145537598	-1.19524943659748	-0.80984111437144
O	-3.01016240520535	-1.64137530586430	-1.10223136116009
C	-5.28445870608269	-2.18309810031553	-0.74791521103368
F	-6.24373111777089	-1.83686186746366	-1.61743891262834
F	-5.83057973636134	-2.21968896209488	0.47281871113855
F	-4.87958984830122	-3.39879717664801	-1.05075769403280

1A_e, Toluene

C	-5.98754777140911	1.71211501096241	-1.28769700983232
C	-5.61768681484966	0.69227457195634	-0.21798723119531
C	-4.91006967561238	2.76322291099130	-1.44506939781205
F	-7.17608496168129	2.31397806646279	-0.89954314356681
H	-6.17233335916623	1.19750594596330	-2.23417491328523
C	-3.55833647324425	2.10292614344811	-1.70781157301941
H	-4.86502380816450	3.34176215003276	-0.51784417984305
H	-5.17461150919793	3.44590445382993	-2.25234145632831
C	-3.24483686859915	1.06381943203636	-0.63938259137672
H	-2.76994730303646	2.85595427820755	-1.71888246782919
H	-3.55675965435489	1.61727327122547	-2.68634572184645
N	-4.33655586736740	0.09793632522928	-0.54539315781804
H	-3.13922297063202	1.54809718379359	0.33556350829396
H	-2.32807723283688	0.52741887838732	-0.86004412729041
H	-6.39657269880970	-0.05522310177206	-0.12937221768286
H	-5.52781947356054	1.20728083956170	0.74265580839053
C	-4.09346091314616	-1.19425824991533	-0.80939107758956
O	-3.01235161494693	-1.65269889394573	-1.09769105628213
C	-5.28807164408118	-2.18261411802868	-0.74156624681621
F	-6.25054285170520	-1.84775172015798	-1.60942158233709
F	-5.82945636094667	-2.22094076788946	0.48032260408286
F	-4.87866017265147	-3.40109261037895	-1.04150276901617

1B_a, Gas Phase

C	-5.38118290670021	1.36934314817799	-0.43140692022412
C	-4.52223322225276	0.63943450131116	-1.44819197314312
C	-4.59926740703858	1.70997331714868	0.82157555120911
C	-3.98612578397298	0.44996960644944	1.42708812937993
C	-3.18405804462960	-0.33695184402030	0.40716031388013
N	-4.03932425923705	-0.61954115607277	-0.79879838398568
H	-4.77478860982288	-0.18185503972961	1.83966558654737
H	-3.32536266905015	0.70566563883217	2.25410330177953
H	-5.82675647914079	2.25207713059679	-0.88811169974140
F	-6.40038502114223	0.48529949343294	-0.10345967653538
H	-5.09552307012229	0.36977050397547	-2.33124499795217
H	-3.65140385885745	1.22707762990600	-1.73366328392586
H	-5.26144991556595	2.19267146239722	1.53858939906354
H	-3.82359749686467	2.43474274146696	0.56123370772022
H	-2.32432217291490	0.22584785458922	0.04403699434971
H	-2.84436599615786	-1.29232436897557	0.79887556907313
H	-3.52862480743946	-1.19233872604531	-1.47025211081058
H	-4.86632827909019	-1.15094189344048	-0.50821950668435

1B_a, Water

C	-5.37555643922067	1.38712407181652	-0.43140491814149
C	-4.54852078286180	0.61161954416939	-1.43167596066371
C	-4.59837170490645	1.70368995207591	0.82802343811486
C	-3.99800134778135	0.43666892283599	1.42746631795700
C	-3.17915024522280	-0.32474509878992	0.40455024243081
N	-4.02154608431824	-0.62284761985514	-0.79157098153346
H	-4.79089147952478	-0.20939359685206	1.80914395386754
H	-3.35150603825812	0.68377291602991	2.26798148427240
H	-5.75861921572923	2.28849357593033	-0.90617441392195
F	-6.47470851215403	0.58626158677941	-0.09745975824224
H	-5.14018534677284	0.31866618537971	-2.29350635242407
H	-3.69555735107182	1.20538291907956	-1.75433185102645
H	-5.25485196245337	2.19992487174503	1.54171127104505
H	-3.80693357393961	2.40866296055215	0.56252905922610
H	-2.33618438493716	0.26403070100636	0.04605383467883
H	-2.81865117710752	-1.27354752901347	0.78961550299619
H	-3.48163118316942	-1.15471287678285	-1.47329318651450
H	-4.80423317057079	-1.22113148610682	-0.51867768212089

1B_e, Gas Phase

C	-5.42960188539503	1.34929036811959	-0.43678253109944
C	-4.58251961231123	0.60733942243422	-1.45066176548275
C	-4.63400478845827	1.68644019806731	0.82263802710561
C	-4.02816970160602	0.43220911194469	1.41945004618730
C	-3.19888958988407	-0.33641588577229	0.39367951842883
N	-4.02958089048824	-0.64058199574366	-0.81521577931373

H	-4.81382483286770	-0.21563562271224	1.82455738665129
F	-3.17297826694542	0.74416608008981	2.43854746615075
H	-5.79956523844623	2.26063379093920	-0.90485688193505
H	-6.31255703283955	0.75235430110222	-0.18961693557757
H	-5.14678144010045	0.30589830012660	-2.32985346807124
H	-3.72055130205703	1.19619290903586	-1.76314442473022
H	-5.27542248017743	2.16585929352477	1.56067526593112
H	-3.82699134988010	2.38496158803321	0.58844992754927
H	-2.36162556130896	0.27744577090140	0.06341281657152
H	-2.82608175222444	-1.27131139979755	0.80422063638614
H	-3.46875210101378	-1.15892482280092	-1.49279518042522
H	-4.80718217399604	-1.25200140749221	-0.55373412432661

1B_e, Water

C	-5.42759802503277	1.35038959541414	-0.43246588337256
C	-4.58290833228645	0.60153090985723	-1.44201390751592
C	-4.62694802709966	1.69206380295992	0.82222131384889
C	-4.03886008367953	0.42889994221555	1.40747757321915
C	-3.20438309715454	-0.32997037051055	0.38814058903438
N	-4.02706794428749	-0.63259727038354	-0.81129525357689
H	-4.82657176273055	-0.21625786131229	1.80314724119494
F	-3.19006162190629	0.73684386937044	2.46182155236797
H	-5.79610241723680	2.26040991370579	-0.90280927873262
H	-6.30083439691646	0.74713373102735	-0.17266903672690
H	-5.15586877976044	0.29009296216539	-2.31012745139329
H	-3.72750055711136	1.19162427336848	-1.76669505562699
H	-5.26407159058250	2.17749044449181	1.56050360258523
H	-3.81111373374405	2.37465940642960	0.57210643424254
H	-2.36889336198246	0.28540883588985	0.05855898361725
H	-2.83688737345560	-1.26712705785038	0.79458529295217
H	-3.45769386716619	-1.13934106103095	-1.48915315380737
H	-4.79171502786685	-1.26333406580782	-0.56236356230998

1C_ae, Gas Phase

C	-5.37619641053129	1.38114859883258	-0.43490158055643
C	-4.53620190300241	0.57695543317770	-1.41300765161446
C	-4.59609159035918	1.69337305561255	0.82930783732564
C	-3.99539622868178	0.42205724691733	1.41975326607591
C	-3.16916028718600	-0.30839885248439	0.36878722220017
N	-4.00607986437508	-0.61044843314175	-0.77797131155445
H	-4.79891165878840	-0.23515662157435	1.75558178570150
H	-3.37487954130627	0.66220116459185	2.28404656581569
H	-5.71167595774963	2.30150732816973	-0.91857648908795
F	-6.51584096879013	0.66515573824053	-0.09910665608016
H	-5.15594657028107	0.28765957546387	-2.26149252131774
H	-3.74713777933170	1.25480677363129	-1.78275802197504
H	-5.24833443139391	2.19746988041759	1.54313162735802
H	-3.79669609351475	2.39303926185433	0.56602154210120
H	-2.30159111672064	0.31850589478672	0.09928869597895
H	-2.78619831488737	-1.24414083090552	0.77604765134065
H	-3.52236128310041	-1.19176521359008	-1.44642196171152

1C_ae, Water

C	-5.36800682641342	1.38681335093588	-0.43867474484200
C	-4.53657404572189	0.57566810488824	-1.41207323224505
C	-4.60073925926293	1.69185910081705	0.83141810623267
C	-3.99367632532601	0.42338619985554	1.42283840228150
C	-3.17875047670179	-0.31436600709610	0.36901797257845
N	-4.02520467713703	-0.62710846711774	-0.77599911642520
H	-4.78900829188756	-0.23224526120606	1.78398015438385
H	-3.36059148306034	0.67269255683541	2.27510073401116
H	-5.71121407445636	2.30297106198034	-0.91969917898803
F	-6.52261915919166	0.65912321957315	-0.10574387748699
H	-5.14780905346371	0.30104579021804	-2.27163587540144
H	-3.73236607823497	1.23887824628569	-1.76666570149127
H	-5.25507151555514	2.19616384010344	1.54354381013929
H	-3.80336670778413	2.39301123262722	0.56877869500667
H	-2.32119663071054	0.31328103818904	0.07944719862483
H	-2.78578714008119	-1.24554409711020	0.77682345048791
H	-3.50671825501135	-1.17165990977895	-1.45272679686637

1C_aa, Gas Phase

C	-5.38332762699692	1.38967080844080	-0.42589557297850
C	-4.55125895609074	0.57280405778189	-1.40190836219068
C	-4.59686388323770	1.71090180576678	0.83188090345474
C	-3.99878771598035	0.44319794622351	1.43735365354468
C	-3.21831935716276	-0.33623564238659	0.38024296781582
N	-3.99860648456596	-0.63093176481786	-0.81248011163631
H	-4.80449810724636	-0.18692615350194	1.82117020765280
H	-3.35303657281529	0.69353105191865	2.28091151434021
H	-5.75815519430211	2.29902690104577	-0.90062413930059
F	-6.49851469330322	0.62822757822667	-0.07408161029492
H	-5.15552938765619	0.30762386537211	-2.26949512142385
H	-3.72495050054220	1.19850257718894	-1.75595730726650
H	-5.24169274043857	2.22551242086517	1.54487199441029
H	-3.79642602470845	2.40467502267139	0.55643680206848
H	-2.34639573387808	0.24832555211592	0.06586415755451
H	-2.83941887083874	-1.27266527014903	0.79011904813197
H	-4.75281815023635	-1.26731075676217	-0.58350902388215

1C_aa, Water

C	-5.37432647923573	1.39655489396221	-0.43093613514302
C	-4.55380551803506	0.56859805032783	-1.40349032834148
C	-4.59994068947921	1.70491051700440	0.83424289041512
C	-3.99897775525811	0.43692928208187	1.43587941424125
C	-3.21111886911819	-0.33302838992706	0.37968205337668
N	-3.99964445459848	-0.63906709864769	-0.81100373173978
H	-4.79767477286975	-0.20421060925914	1.81749600205658
H	-3.35415115901680	0.68897469871542	2.27929113140709
H	-5.73637663243034	2.30742345552966	-0.90865293130543
F	-6.51567723385557	0.64657345883784	-0.08656627542450
H	-5.16432829686426	0.30407262142405	-2.26704349021782
H	-3.72948841669737	1.19382132220505	-1.76040418276588
H	-5.24939901785735	2.21621020111127	1.54578735365295
H	-3.80027926722877	2.40124337590694	0.56524310078383
H	-2.35041722400606	0.26453911142380	0.06071599031484
H	-2.82308526156607	-1.26484251579139	0.79137436665208
H	-4.75990895188288	-1.26077237490503	-0.55671522796251

1C_ee, Gas Phase

C	-5.41969279523247	1.35514028713711	-0.44788299028893
C	-4.55052001594963	0.58398067912551	-1.43230081469529
C	-4.63363340499944	1.67925572579942	0.82154638837976
C	-4.02595101903969	0.41677782279080	1.39665978464326
C	-3.19952907095489	-0.31300630866887	0.35061919506189
N	-4.04415512614800	-0.61668817612041	-0.78898425741357
H	-4.80684019325343	-0.24768906889361	1.77471318725841
F	-3.20209195617019	0.74184951750300	2.46434980269972
H	-5.78193779438680	2.27486011169410	-0.90884587482818
H	-6.29196411221683	0.74570372378279	-0.20035913083670
H	-5.13601128362544	0.29353055557407	-2.30478904675403
H	-3.73662875635171	1.24184292280353	-1.77863913621291
H	-5.26614618774376	2.16291548480293	1.56672273358505
H	-3.81562502081951	2.36658513317982	0.58608136629127
H	-2.34440004684876	0.33264841364746	0.09458308042373
H	-2.81257779014637	-1.23882165420690	0.77528673822322
H	-3.55899542611308	-1.20774516995075	-1.44829102553669

1C_ee, Water

C	-5.42016405755767	1.35492012335193	-0.44595345287008
C	-4.55525598915239	0.57938874102458	-1.42957284364705
C	-4.63198588106071	1.68103447650457	0.82241349640925
C	-4.03316488704750	0.41473777497633	1.39131146410648
C	-3.20326823668504	-0.31540963236551	0.35119322279884
N	-4.04888147911118	-0.62884452324981	-0.78929853697847
H	-4.81264769563751	-0.24387909105887	1.77980953733785
F	-3.20033721705279	0.73752471620768	2.47020806111585
H	-5.77606928995301	2.27573725026333	-0.90884103645938
H	-6.29756176526196	0.75421997416730	-0.19304395080176
H	-5.13969543822965	0.29257499013787	-2.30368427585382
H	-3.73530176353416	1.22895500500352	-1.77197722225025
H	-5.26575244683024	2.16633066903357	1.56556532891314

H	-3.81214381739395	2.36354473239053	0.57898886135220
H	-2.35586407009132	0.33378511416012	0.08246034600702
H	-2.80907245422863	-1.23806603733925	0.77634199891957
H	-3.52953351117231	-1.18541428320790	-1.45545099809938

1C_ea, Gas Phase

C	-5.43634173331009	1.36838339098993	-0.43246689575380
C	-4.58555827280096	0.56302538902835	-1.41216928326851
C	-4.63021338223688	1.69390635152229	0.82443004714573
C	-4.03519715818233	0.43045465466590	1.41315797934160
C	-3.23664443466339	-0.33735937289317	0.36879184272578
N	-4.01092518541287	-0.63534677891809	-0.82219464555994
H	-4.82774548736244	-0.21080142663592	1.81364915486522
F	-3.20139169042114	0.75835364716127	2.47259529096088
H	-5.79309550896914	2.28771419509476	-0.90063364711887
H	-6.32089731621361	0.78096672682060	-0.16723047642629
H	-5.16420142827467	0.27875176062709	-2.29139834705838
H	-3.75596338764794	1.18474180605483	-1.76529618284600
H	-5.24480415901749	2.19760067435757	1.57120151424890
H	-3.80461019425483	2.36525639382785	0.56999491429238
H	-2.37891040359934	0.27803557124415	0.07864228444124
H	-2.84583346246042	-1.26081517086046	0.79549209517864
H	-4.73563679517247	-1.31282781208695	-0.61938564516855

1C_ea, Water

C	-5.43313611492518	1.36469755973587	-0.43355693419930
C	-4.58166669688759	0.56510341828104	-1.41511148718766
C	-4.62847410134264	1.69332772519720	0.82396792073797
C	-4.04120040411698	0.42595973105332	1.40378616234178
C	-3.23156257387481	-0.33532820439980	0.36641427054119
N	-4.01044727191218	-0.64134599353018	-0.82417701500718
H	-4.82911520254764	-0.21694237318645	1.80457325655709
F	-3.20096129727842	0.75023056217855	2.47820502482509
H	-5.79045176391247	2.28346467611659	-0.90134640399825
H	-6.31306492064244	0.77175338709369	-0.16744298110217
H	-5.16564333092795	0.28178322175048	-2.29091305543600
H	-3.75154983965863	1.18725140414878	-1.76533545767191
H	-5.24948658079710	2.19264282116866	1.56858742765446
H	-3.80352871736703	2.36479794404098	0.56676666787329
H	-2.37974035530205	0.28696242276425	0.07310785953628
H	-2.84147322599829	-1.25738842263342	0.79716970109466
H	-4.75646760250859	-1.28692987977955	-0.58751495655931

2A_a, Gas Phase

C	-5.49547788491601	1.20136414606274	0.36670195753307
C	-5.05203416406787	0.68438709051080	-0.99211531142127
C	-4.31316230226270	1.65615610309422	1.20753378507599
C	-3.18778059226802	0.63581833375096	1.23806781704885
C	-2.81836980897996	0.17259687015943	-0.16278226215226
N	-3.99266868225212	-0.29902628505498	-0.85974773078820
C	-4.20849192282403	-1.55128497394068	-1.32124725947614
O	-5.21703034989721	-1.91302994523473	-1.86537580609417
C	-3.05262614028086	-2.57906804104932	-1.17940875073376
F	-1.95757359377885	-2.15529916508462	-1.82897715937758
F	-3.41496702643112	-3.73171427123294	-1.70280411526310
F	-2.71602412099266	-2.78740861242379	0.09505383864382
H	-5.89013741201274	0.22752696302279	-1.50981206971380
H	-4.68672427933247	1.52886319947613	-1.58714892605308
H	-2.39189451335894	1.01790843186051	-0.71430373501257
H	-2.06226984522235	-0.60056889260848	-0.10142708681464
H	-6.19103991378920	2.03130057880733	0.21966719889395
F	-6.17967761457146	0.20251040472207	1.02663351491863
H	-4.64177835270379	1.88129317975319	2.22141300990980
H	-3.92084763911737	2.58038370909854	0.77309149592530
F	-3.56944579854076	-0.46365802037320	1.97690984170049
H	-2.30381804239948	1.06311919668404	1.71597775325067

2A_a, Benzene

C	-5.49331044478235	1.20338364227745	0.36343521543200
C	-5.05119738479974	0.68957285676452	-0.99515626299417
C	-4.31444452537740	1.65226070999950	1.20864320865379
C	-3.18649819275991	0.63731388104599	1.23257649754046
C	-2.81703432563172	0.17578187867252	-0.16711988840773
N	-3.99536639874543	-0.30137376957429	-0.86007937553813
C	-4.20820377982387	-1.54799794250357	-1.31369498165932
O	-5.22158412192675	-1.92317434237425	-1.85144378337024
C	-3.05137364496482	-2.57555831339262	-1.17442436522882
F	-1.95635809517151	-2.16028999385443	-1.82508086371757
F	-3.41858584110332	-3.73026396886846	-1.69785413969366
F	-2.71599243843819	-2.78897511424423	0.09947043374536
H	-5.89117456121757	0.24488873608333	-1.51900592071624
H	-4.67426967424841	1.53252899804687	-1.58239120648982
H	-2.40094782485481	1.02298627656609	-0.72053765169355
H	-2.05551792990363	-0.59192508364160	-0.10808234850359
H	-6.19206553812722	2.02942964289131	0.22099658423427
F	-6.18046919864250	0.19324737481675	1.02000590899364
H	-4.64314703883196	1.87376749855584	2.22333125202034
H	-3.92379857369213	2.57873918142123	0.77893265487394
F	-3.56859812259583	-0.47252442640897	1.97004537946125
H	-2.30390234436089	1.06035227772103	1.71333365305780

2A_a, Dichloromethane

C	-5.49142537618273	1.20143269200387	0.35680679852920
C	-5.04548354128812	0.70024097404201	-1.00380340354126
C	-4.31779527441729	1.64199450317750	1.21091734720176
C	-3.18496199394940	0.63435241646571	1.22469420559098
C	-2.81223357894701	0.18470658403579	-0.17690063151650
N	-3.99229890346521	-0.29662230364325	-0.86919791258420
C	-4.21129380849534	-1.54433286774124	-1.29696477345741
O	-5.23602469921678	-1.93471340859059	-1.81225920529330
C	-3.05312219752292	-2.57150160762659	-1.16321654437160
F	-1.96296645840449	-2.16743360721525	-1.82435811721563
F	-3.42933525504199	-3.73055423957509	-1.67781711408719
F	-2.71022184521497	-2.78301961601433	0.10898123634899
H	-5.88408082408374	0.26839412939136	-1.53952396528719
H	-4.65675699413035	1.54606999170635	-1.57694490855195
H	-2.40795904702411	1.03847224488035	-0.72647960319367
H	-2.04343716974306	-0.57612309278911	-0.12555373477936
H	-6.19382087517988	2.02434861763230	0.22233914634265
F	-6.18026810377785	0.17574417702807	1.00137512004984
H	-4.64886049414013	1.85298957974560	2.22709779339317
H	-3.93006469685995	2.57429897924258	0.79229593610566
F	-3.56602549378412	-0.48999354624481	1.95233864177885
H	-2.30540336913051	1.05341940008877	1.71207368853815

2A_a, Chloroform

C	-5.49205319180212	1.20130006413972	0.35835594340562
C	-5.04657252819309	0.69785851032854	-1.00206529661224
C	-4.31708389613248	1.64433734103503	1.21026335785888
C	-3.18535248225441	0.63479088186227	1.22669218779463
C	-2.81317661371867	0.18266033164413	-0.17462369668364
N	-3.99231680289206	-0.29704358403111	-0.86799200300539
C	-4.21087445962244	-1.54526991785251	-1.30080418091291
O	-5.23312867646487	-1.93149423752897	-1.82067128613024
C	-3.05313657826535	-2.57263823026593	-1.16567955938250
F	-1.96192144055215	-2.16553488584820	-1.82476560780874
F	-3.42711806893538	-3.73055492859162	-1.68213968606452
F	-2.71142881242461	-2.78412976683622	0.10680445066521
H	-5.88524410755102	0.26249979020230	-1.53509152362099
H	-4.66136667122730	1.54360942571421	-1.57827454609916
H	-2.40528350692088	1.03495865230322	-0.72450356758627
H	-2.04636821975985	-0.58010102306996	-0.12133145469304
H	-6.19363463946426	2.02503052649041	0.22214716776132
F	-6.17976839880838	0.17939832534876	1.00553324613178
H	-4.64768098281087	1.85760357175089	2.22612840402299
H	-3.92873929521667	2.57530626399068	0.78899864067397
F	-3.56644455971925	-0.48567728705346	1.95652893621840
H	-2.30514606726385	1.05526017626781	1.71239007406683

2A_a, DMSO

C	-5.49078800142334	1.19766001790688	0.35164830643153
C	-5.03995969098377	0.70865435134331	-1.01134935848757
C	-4.32062713543774	1.63337531064269	1.21229856970188

C	-3.18393794235160	0.63050578798050	1.21934002939565
C	-2.80783899367855	0.19197536231145	-0.18470443718721
N	-3.98732380255106	-0.29011814410523	-0.87913438123771
C	-4.21460160364306	-1.54179847033841	-1.28392106100904
O	-5.24846948818835	-1.93992771756491	-1.77842888778426
C	-3.05607998694507	-2.56918075633451	-1.15403163400302
F	-1.97061625358584	-2.17220663735273	-1.82547915669094
F	-3.43888681453971	-3.73126334127422	-1.65939645490695
F	-2.70456514815278	-2.77565593383174	0.11659519098774
H	-5.87617399638429	0.28401427955190	-1.55625060543876
H	-4.64565464287018	1.55863764936949	-1.57356446496094
H	-2.40936635080547	1.05117749506804	-0.72901022936896
H	-2.03480589029625	-0.56497285962632	-0.13942396190085
H	-6.19581914801966	2.01887528437097	0.22342512666072
F	-6.17865734047800	0.16153020960882	0.98538702170287
H	-4.65402652767727	1.83507499938713	2.22963479291538
H	-3.93541638420924	2.57046798279074	0.80253658806326
F	-3.56338222428093	-0.50268491752252	1.93799723297351
H	-2.30684263349780	1.04803004761866	1.71173177414367

2A_a, Water

C	-5.49070707117576	1.19736799904005	0.35109661007657
C	-5.03935266153654	0.70941752657847	-1.01204021693812
C	-4.32095406100442	1.63263058333463	1.21244461188709
C	-3.18382339706869	0.63031166674251	1.21887140257162
C	-2.80738903265819	0.19262595015247	-0.18533205318376
N	-3.98688789657876	-0.28960572656631	-0.87980264069864
C	-4.21495830722294	-1.54160299975969	-1.28250454530009
O	-5.24974289206749	-1.94034249542814	-1.77498993283194
C	-3.05637301913228	-2.56899965643722	-1.15335471639950
F	-1.97146704244371	-2.17255799484905	-1.82582880118969
F	-3.43985188115585	-3.73129142236054	-1.65796336006676
F	-2.70388966300996	-2.77517113184730	0.11705575977840
H	-5.87530132233790	0.28541734955535	-1.55783495421885
H	-4.64436676049308	1.55965478691776	-1.57332031523669
H	-2.40944569388143	1.05219853195662	-0.72936049279210
H	-2.03404981025760	-0.56403702940161	-0.14044850755461
H	-6.19610333069205	2.01831794894466	0.22346770157971
F	-6.17851114425183	0.16018253657070	0.98374644918019
H	-4.65461314929197	1.83342336844228	2.22987297467195
H	-3.93602722533388	2.57022323981849	0.80357025013716
F	-3.56304072884438	-0.50368701453796	1.93678228324063
H	-2.30698390956128	1.04769398313385	1.71177023828742

2A_e, Gas Phase

C	-5.48730074357699	1.18065010181729	0.25018098511048
C	-4.98977050454420	0.62399073505034	-1.07720761992831
C	-4.34116719684203	1.73518525554965	1.07666646277810
C	-3.23826035079541	0.70480565063605	1.24447011887264
C	-2.80685456690662	0.13341542015928	-0.09993668033862
N	-3.96807056021272	-0.36897358150121	-0.81744076486605
C	-3.97587966437698	-1.66102386312631	-1.22104866411845
O	-3.09693307466571	-2.45026391003948	-1.00055850542645
C	-5.20721672280481	-2.15619404281650	-2.02765964435058
F	-6.33760485007272	-2.02375144000390	-1.32139685180329
F	-5.06129261429879	-3.42814545657443	-2.33112046244420
F	-5.35056376592440	-1.47035886962049	-3.16714647255704
H	-5.81776028448859	0.20556029596093	-1.63635170627428
H	-4.55930241475682	1.44107761736653	-1.66443494711938
H	-2.33591826017131	0.93196613762794	-0.68182061048545
H	-2.09393890885420	-0.67428397069479	0.03224336007438
F	-6.38724314208887	2.19084224352994	-0.01656424737597
H	-6.01980721408293	0.39351950680112	0.79145781995588
H	-4.70114288577197	2.06948488968234	2.04906345278722
H	-3.92567842528040	2.59940550271330	0.55143201950860
H	-3.55476143083837	-0.10770131262585	1.90353690946735
F	-2.14270241864515	1.30813308610821	1.82799604853343

2A_e, Benzene

C	-5.48440170332886	1.17750256892232	0.25363999951856
C	-4.99034301475303	0.62192513577160	-1.07507232004268
C	-4.33957731938791	1.73882368149361	1.07558344299164
C	-3.24194375310732	0.70473454889282	1.24449624891695
C	-2.80657089650239	0.13315592819999	-0.09805926466085
N	-3.96830197172695	-0.37342295681905	-0.81500785136882
C	-3.97795354790196	-1.65734601275218	-1.22063613275597
O	-3.09842391267077	-2.45449783030147	-1.00646321792438
C	-5.20825540878908	-2.15225845095559	-2.02972735047600
F	-6.34077753907631	-2.02731030128067	-1.32862685345208
F	-5.05712715799381	-3.42675745557667	-2.33415456781106
F	-5.35041694009460	-1.47057282408937	-3.17063194636476
H	-5.82000884685118	0.20532413864728	-1.63288435190949
H	-4.55518613761730	1.43555541212208	-1.66218203704020
H	-2.34198729582890	0.93100335655587	-0.68471186524079
H	-2.09051123103005	-0.67041846427618	0.03860212106933
F	-6.38870710033895	2.18956200705708	-0.01695173067895
H	-6.01753793942141	0.39527266366986	0.79928859421300
H	-4.69950205411419	2.07294761445126	2.04800590651906
H	-3.92341633284738	2.59910696822966	0.54447686811697
H	-3.55798971795096	-0.10357871008516	1.90709567826288
F	-2.14023017866668	1.30858898212288	1.82828063011766

2A_e, Dichloromethane

C	-5.48236399961090	1.17640530419264	0.25707338758549
C	-4.99187269732562	0.61932482331600	-1.07200565963137
C	-4.33765296531952	1.74321519590598	1.07417438438208
C	-3.24511247881649	0.70544569025600	1.24531301425817
C	-2.80738956607184	0.13175693857135	-0.09524607391508
N	-3.96974880957233	-0.37801584008843	-0.81128058864377
C	-3.97866650271224	-1.65473917739540	-1.22218373576076
O	-3.09635446757276	-2.45662731183887	-1.01651663459926
C	-5.20849335839142	-2.14943995796257	-2.03246273640677
F	-6.34189303425663	-2.03211602140694	-1.33423111851639
F	-5.05245161226728	-3.42556479829736	-2.33932278926890
F	-5.35086966192932	-1.46971327852972	-3.17347051601008
H	-5.82322581786192	0.20334486076807	-1.62758268813408
H	-4.55312841250633	1.42923021814441	-1.66059496104431
H	-2.34676353987292	0.92794434614515	-0.68640971628455
H	-2.08991098139626	-0.66939358082562	0.04561642818500
F	-6.38936907695811	2.18966794247967	-0.01750636337979
H	-6.01573493687459	0.39880381569198	0.80766079381043
H	-4.69700869625266	2.07884036193368	2.04623691851858
H	-3.92038524574296	2.59918695771587	0.53709459092915
H	-3.56258999892757	-0.09865605139679	1.91114915206799
F	-2.13818413976032	1.30843956262086	1.82885491185824

2A_e, Chloroform

C	-5.48298594375173	1.17627238173013	0.25561761375052
C	-4.99090796489319	0.62072076903535	-1.07366096054751
C	-4.33853609126418	1.74107803699770	1.07486838748552
C	-3.24393310166350	0.70482659042188	1.24472070356538
C	-2.80669983752016	0.13286599005541	-0.09686183582743
N	-3.96864143037587	-0.37588564206058	-0.81350086175048
C	-3.97886739234940	-1.65541963814030	-1.22079408264700
O	-3.09852015877281	-2.45651607511163	-1.01050647052338
C	-5.20869602847847	-2.15020604396907	-2.03097753769520
F	-6.34204540013070	-2.02972199020806	-1.33231083957083
F	-5.05452351786314	-3.42609172625699	-2.33623912678789
F	-5.35039838225829	-1.47058890413281	-3.17242338311598
H	-5.82145663076094	0.20485825598787	-1.63059019229984
H	-4.55331237625315	1.43229177434976	-1.66108571313925
H	-2.34532300974825	0.93021456450860	-0.68610809695919
H	-2.08918596624794	-0.66873672009174	0.04205833760916
F	-6.38936282854867	2.18912690840130	-0.01710553093338
H	-6.01620803987598	0.39662380359948	0.80388931018838
H	-4.69828765956686	2.07550755883226	2.04721954209412
H	-3.92184432317154	2.59900668171806	0.54040953818851
H	-3.56050110967000	-0.10118581032027	1.90903980067485
F	-2.13893280683520	1.30829923465362	1.82870139824093

2A_e, DMSO

C	-5.48175967671952	1.17602724705925	0.25802221679237
C	-4.99224249277183	0.61872097768259	-1.07124268705880
C	-4.33710603257616	1.74438753865249	1.07379860911676
C	-3.24608249939098	0.70551659850607	1.24549411032636
C	-2.80755620186928	0.13155234995402	-0.09459902176874

N	-3.96999754759197	-0.37920229175506	-0.81048268523852
C	-3.97894054154612	-1.65385812495419	-1.22250673609968
O	-3.09590165901880	-2.45731359452962	-1.01907712357951
C	-5.20865050603948	-2.14854700392342	-2.03306036000901
F	-6.34226717288723	-2.03323031899958	-1.33576884693970
F	-5.05121136076493	-3.42534271711109	-2.34026641475998
F	-5.35090445345356	-1.46981609224981	-3.17430958171553
H	-5.82400780191579	0.20297741948586	-1.62632806589258
H	-4.55238838185646	1.42758545898309	-1.66011949110357
H	-2.34831726743371	0.92744166998769	-0.68698408436495
H	-2.08951604950565	-0.66879106946038	0.04731127594197
F	-6.38960399070306	2.18959692960870	-0.01759293486731
H	-6.01513260417165	0.39964686058521	0.80992353183993
H	-4.69634311513694	2.08028395562101	2.04579431879449
H	-3.91951098056738	2.59916641375359	0.53509632833780
H	-3.56409177788749	-0.09750338891547	1.91204755347686
F	-2.13763788619197	1.30804118201900	1.82921008877134

2A_e, Water

C	-5.48170027830551	1.17597071089433	0.25809733806368
C	-4.99225365255543	0.61869550064658	-1.07120280796377
C	-4.33706571257470	1.74446584016547	1.07377377422463
C	-3.24617493598679	0.70550113176398	1.24549936606203
C	-2.80755246718672	0.13156635359466	-0.09456916076630
N	-3.96998908127178	-0.37928204357258	-0.81045887497197
C	-3.97898353253492	-1.65375609887288	-1.22250619266644
O	-3.09590719221354	-2.45739608590783	-1.01922956122420
C	-5.20868214235572	-2.14845452088098	-2.03308302942917
F	-6.34231977591200	-2.03329406387209	-1.33589509011239
F	-5.05112252102664	-3.42533801359477	-2.34026452193243
F	-5.35090840899495	-1.46988376554823	-3.17439027865985
H	-5.82404577542305	0.20299478922684	-1.62627322643930
H	-4.55228751272492	1.42748111263188	-1.66007322980652
H	-2.34847030412557	0.92745767071624	-0.68704267066669
H	-2.08943454100609	-0.66868303986952	0.04741383699482
F	-6.38963303506775	2.18957044551868	-0.01759214576890
H	-6.01506667236533	0.39967921118592	0.81008999461621
H	-4.69630242072037	2.08035608129864	2.04577055389848
H	-3.91944718332112	2.59915455848352	0.53494938063199
H	-3.56422914078497	-0.09744287858633	1.91208968220038
F	-2.13759371354212	1.30797710457841	1.82925686371573

2C_ee, Gas Phase

C	-5.50478699130178	1.27851878588881	-0.38425569971181
C	-4.62354315194148	0.55052243472327	-1.38549771151688
C	-4.72266937119995	1.63288590792131	0.86849804456875
C	-4.01896237532667	0.41250317449696	1.43612532196774
C	-3.19399409836644	-0.28383208181569	0.36669579007488
N	-4.07393177386846	-0.62344263785578	-0.73501067430544
H	-3.61905470480309	-1.23390454551928	-1.39682176885427
H	-5.22077636765058	0.24662026588099	-2.24433551307182
H	-4.73838530675017	-0.29409107537028	1.85615860906646
F	-3.18194719790860	0.81324676122076	2.46334286512325
H	-3.85190919746668	1.25504685865422	-1.73216538318969
F	-5.97988921616470	2.44322103259675	-0.96214963022465
H	-6.36923755512673	0.65577140458120	-0.14299858349171
H	-2.37667875138990	0.39374680108682	0.07541243966375
H	-2.75570769165301	-1.19238895038662	0.77787459401885
H	-5.37856108988486	2.07904063850790	1.61596826514360
H	-3.96200515919688	2.37033522538864	0.59837903473898

2C_ee, Benzene

C	-5.50426510764347	1.27508708901019	-0.38184732900011
C	-4.62510279904014	0.54883458809620	-1.38446177621847
C	-4.72202178143486	1.63399631986389	0.86852483136247
C	-4.02230284141668	0.41128390768486	1.43383294651783
C	-3.19598257169613	-0.28519502381475	0.36710272532258
N	-4.07663365697140	-0.62835422900168	-0.73512873944918
H	-3.60548215231643	-1.22200976919857	-1.40231794528330
H	-5.22141668963801	0.24574282846536	-2.24414319060730
H	-4.74096814480512	-0.29130074206760	1.86002603806427
F	-3.18079501803079	0.81423739591193	2.46475808506000
H	-3.84954765119857	1.25002146996477	-1.72761696370677
F	-5.98018775371588	2.44517332449163	-0.96283143049833
H	-6.37220751524927	0.65888061207598	-0.13953273352093
H	-2.38201893600951	0.39335708635109	0.07053250177794
H	-2.75632186925698	-1.19312549991883	0.77800110845512
H	-5.37814141827131	2.07985504653133	1.61598543467052
H	-3.95864409330544	2.36731559555417	0.59433643705365

2C_ee, Dichloromethane

C	-5.50395982292461	1.27271899873416	-0.38015050141963
C	-4.62618596638370	0.54763206664160	-1.38369172507972
C	-4.72149238145420	1.63477250139928	0.86849276849058
C	-4.02467022110422	0.41040904030587	1.43229259965093
C	-3.19737922025302	-0.28610256598300	0.36736717204426
N	-4.07844317868661	-0.63166520915562	-0.73518966117354
H	-3.59593178834279	-1.21320747118145	-1.40597897564793
H	-5.22176468118795	0.24496879914572	-2.24399798584040
H	-4.74316945505047	-0.28930474732473	1.86234633631658
F	-3.18027187653539	0.81494466616018	2.46605405434947
H	-3.84784354300571	1.24623172100524	-1.72466781471046
F	-5.98063284890728	2.44663928736085	-0.96311362343150
H	-6.37410103815229	0.66085441295894	-0.13671594656693
H	-2.38566162358797	0.39287553158597	0.06694040806962
H	-2.75667733213537	-1.19364744850154	0.77790719935337
H	-5.37769986419651	2.08057557458192	1.61596411592836
H	-3.95615515809187	2.36510484226660	0.59136157966693

2C_ee, Chloroform

C	-5.50404657363386	1.27342656228419	-0.38064764609195
C	-4.62587225309804	0.54798195973393	-1.38392270050420
C	-4.72163940732444	1.63455497814690	0.86849879243122
C	-4.02396849575401	0.41067954108340	1.43274905090269
C	-3.19697152976043	-0.28585111887059	0.36729359293328
N	-4.07788695352833	-0.63068192933625	-0.73518940695699
H	-3.59869650130228	-1.21579349331435	-1.40493958365174
H	-5.22171598246702	0.24519054317632	-2.24401198428021
H	-4.74253141496460	-0.28988954290799	1.86162507096086
F	-3.18042023875951	0.81471576609904	2.46564777827680
H	-3.84839844319582	1.24739119962726	-1.72557491051685
F	-5.98050350701435	2.44617547023451	-0.96304014237798
H	-6.37350721423017	0.66023809727487	-0.13753503491383
H	-2.38455070227166	0.39299313548389	0.06806958051115
H	-2.75662754339563	-1.19351749807956	0.77797887835313
H	-5.37780882026613	2.08039347486481	1.61596589431637
H	-3.95689441903370	2.36579285449960	0.59225277060823

2C_ee, DMSO

C	-5.50388344664023	1.27199396863767	-0.37963300665206
C	-4.62650006675729	0.54730069208188	-1.38345714115290
C	-4.72129511007881	1.63502258807821	0.86844827196614
C	-4.02538385097533	0.41015582825832	1.43183267657581
C	-3.19781244694700	-0.28638625661391	0.36745916175716
N	-4.07898193678030	-0.63263486411470	-0.73521697486008
H	-3.59308237928625	-1.21050418278005	-1.40705217457500
H	-5.22181030352012	0.24477358437099	-2.24398707822654
H	-4.74389015993336	-0.28866620066303	1.86301138508363
F	-3.18016521055085	0.81515143110692	2.46648507975220
H	-3.84730328368987	1.24509645217882	-1.72376726586557
F	-5.98090474520646	2.44703911968752	-0.96313651464314
H	-6.37462131283165	0.66135873158146	-0.13580293320830
H	-2.38673975793437	0.39266934417699	0.06586111097892
H	-2.75679192753585	-1.19382775620811	0.77786006848074
H	-5.37751109393442	2.08083416830111	1.61592969250902
H	-3.95536296739780	2.36442335191990	0.59038564207996

2C_ee, Water

C	-5.50387561576117	1.27192340902154	-0.37958328198857
C	-4.62653127105998	0.54726575232815	-1.38343303424276
C	-4.72127902367362	1.63504402073965	0.86844661391270
C	-4.02545404592758	0.41012826060848	1.43178815055973
C	-3.19785279079768	-0.28641053029777	0.36746636817241

N	-4.07903789500465	-0.63273250556162	-0.73521611180664
H	-3.59280922662588	-1.21024302101129	-1.40715353765311
H	-5.22181211176066	0.24475156328417	-2.24398669115066
H	-4.74395766611021	-0.28860777232962	1.86308035990586
F	-3.18015524897468	0.81517367692069	2.46653006650655
H	-3.84724625419469	1.24497778806258	-1.72367629361952
F	-5.98092080837398	2.44708576531663	-0.96314119340494
H	-6.37467980769057	0.66141851085657	-0.13571836330998
H	-2.38685169095322	0.39265803092551	0.06574580338391
H	-2.75679238950686	-1.19383932685036	0.77784967836576
H	-5.37749759122637	2.08085462041596	1.61592838025889
H	-3.95528656235818	2.36435175757072	0.59029308611035

2C_ea, Gas Phase

C	-5.52029632184291	1.29199637861722	-0.36454675994743
C	-4.66823488379466	0.52340550938029	-1.36486155081076
C	-4.71085112280706	1.65378568834084	0.86892735784208
C	-4.03514970987221	0.42599247502435	1.45502873369035
C	-3.23565200608012	-0.31188565410857	0.39038252417021
N	-4.03938163636014	-0.64082809561556	-0.77115438475923
H	-4.71442621170317	-1.36548141008678	-0.56511098363865
H	-5.26823104538235	0.22027839443901	-2.22224760877123
H	-4.77603936257498	-0.25261429922372	1.89033435331240
F	-3.19140440221460	0.81800695043962	2.48055920418213
H	-3.88449342760046	1.19327457381031	-1.73223151094168
F	-5.99418036231976	2.45235681530223	-0.95298491568900
H	-6.39358794222202	0.69058721075435	-0.09138649664009
H	-2.41179227379021	0.33456692336122	0.07212648714068
H	-2.79831263452701	-1.21987670050936	0.80421894439797
H	-5.34542868499694	2.13263395682602	1.61459042260171
H	-3.93487797191141	2.36539128324852	0.57421618386052

2C_ea, Benzene

C	-5.51820323467216	1.28675194324158	-0.36352028835746
C	-4.66572212337288	0.52461337390226	-1.36663736488061
C	-4.71031981934844	1.65337897019244	0.86833117996118
C	-4.03761843390052	0.42331189997136	1.45050354748358
C	-3.23281952918606	-0.31074213013244	0.38887047282967
N	-4.03880836650404	-0.64318035918951	-0.77271882570476
H	-4.72637561318410	-1.35207011088438	-0.54877592755418
H	-5.26755103017087	0.22141117245895	-2.22264362878075
H	-4.77607841262679	-0.25510647766357	1.88680559877968
F	-3.19121230279031	0.81679117896475	2.48206087837477
H	-3.88083530063210	1.19493449643155	-1.73045670876703
F	-5.99565499411787	2.45238168893107	-0.95396466504809
H	-6.39166868926351	0.68718751942113	-0.09233637850076
H	-2.41163085237118	0.33844605841460	0.06961332375503
H	-2.79714191793081	-1.21877267845863	0.80420853678438
H	-5.34733026519466	2.12867095153096	1.61427769179240
H	-3.93336911473373	2.36358250286788	0.57224255783292

2C_ea, Dichloromethane

C	-5.51689414162327	1.28316062895566	-0.36277211582572
C	-4.66413456031432	0.52540959869702	-1.36776479614883
C	-4.71008970876996	1.65302548114031	0.86797321398721
C	-4.03934859127441	0.42149222338483	1.44749763029345
C	-3.23101200117738	-0.31002874234431	0.38793707191417
N	-4.03826281794196	-0.64457483468921	-0.77382009183812
H	-4.73357911838721	-1.34320612433080	-0.53856607406558
H	-5.26753700477383	0.22191526200360	-2.22250799006878
H	-4.77602220434234	-0.25673857369712	1.88467415152357
F	-3.19040688343034	0.81658928549614	2.48266487965691
H	-3.87867679620545	1.19622721392310	-1.72948002933405
F	-5.99596162332389	2.45285858825536	-0.95488308050765
H	-6.39053691908115	0.68499434754153	-0.09298895499301
H	-2.41127291580094	0.34082384801516	0.06830126652129
H	-2.79710239157532	-1.21833081208692	0.80443032547480
H	-5.34897857077301	2.12553185547724	1.61413259293276
H	-3.93252375120522	2.36244075425841	0.57103200047753

2C_ea, Chloroform

C	-5.51726964964180	1.28423407139090	-0.36299399151284
C	-4.66459623672763	0.52517852859815	-1.36743886026424
C	-4.71012765487653	1.65315199991880	0.86807009997470
C	-4.03883769775610	0.42203119245497	1.44838191845316
C	-3.23153879797630	-0.31023061145056	0.38820736116795
N	-4.03842839614622	-0.64416968681864	-0.77349492021050
H	-4.73149330216819	-1.34581725041292	-0.54153524473915
H	-5.26751733851569	0.22177031650847	-2.22257468519093
H	-4.77608334061234	-0.25624567119293	1.88525838816207
F	-3.19073455741891	0.81655022264396	2.48254567486149
H	-3.87929995228255	1.19583694509790	-1.72976662549666
F	-5.99596936165980	2.45265498283032	-0.95458041145415
H	-6.39083603344538	0.68562243844757	-0.09276727739870
H	-2.41139925994289	0.34014948806751	0.06867389689083
H	-2.79704941309297	-1.21843187774599	0.80434666434804
H	-5.34842704673711	2.12653447537667	1.61416498330987
H	-3.93273196099961	2.36277043628580	0.57136302909904

2C_ea, DMSO

C	-5.51653519234336	1.28211061837346	-0.36254170161122
C	-4.66369461430029	0.52564835274570	-1.36807927637151
C	-4.71005394275268	1.65291380629381	0.86787857391695
C	-4.03985064905856	0.42097969482715	1.44662966910918
C	-3.23050569276988	-0.30985163892798	0.38769300768316
N	-4.03805502677374	-0.64493581199490	-0.77416268587594
H	-4.73550663731945	-1.34068664218846	-0.53579261879499
H	-5.26761684393014	0.22199057999060	-2.22238330007935
H	-4.77598507399139	-0.25718319276748	1.88409953603559
F	-3.19004211515994	0.81665811403947	2.48276579091877
H	-3.87812073067721	1.19665757640699	-1.72924622116298
F	-5.99592954429772	2.45308479920368	-0.95518816550504
H	-6.39024219505022	0.68437582712106	-0.09317720611046
H	-2.41110641439659	0.34143532114690	0.06800765115250
H	-2.79724156853430	-1.21828286620104	0.80454704821323
H	-5.34952992394301	2.12453888826173	1.61410625486990
H	-3.93232383470151	2.36213657366929	0.57070364361219

2C_ea, Water

C	-5.51650149305976	1.28199962183909	-0.36251524032071
C	-4.66364508644852	0.52567917312365	-1.36811017457310
C	-4.71004613932052	1.65290985850481	0.86786468475685
C	-4.03990011807953	0.42093798799745	1.44653896932133
C	-3.23046285485699	-0.30984536551206	0.38767232417725
N	-4.03803500866133	-0.64496904242851	-0.77420168747223
H	-4.73570123896101	-1.34043518358196	-0.53553553596144
H	-5.26761178995309	0.22201418077156	-2.22237895523420
H	-4.77598333385805	-0.25721105384938	1.88404643947666
F	-3.18998954391616	0.81668152888171	2.48276061692987
H	-3.87805783804581	1.19671317616589	-1.72920859516912
F	-5.99595424506458	2.45309332197090	-0.95521305579106
H	-6.39019754800279	0.68428666186756	-0.09318389131180
H	-2.41108132163761	0.34147096410935	0.06798889577570
H	-2.79728147892481	-1.21829467648160	0.80456717248584
H	-5.34958258401159	2.12443719698248	1.61410396191963
H	-3.93230837719787	2.36212164963906	0.57066407099053

2C_ae, Gas Phase

C	-5.46864901899963	1.34280956478042	-0.40679129644649
C	-4.60832156410684	0.56553908537068	-1.38954082453302
C	-4.69682872413433	1.66519164675588	0.86295016261593
C	-3.96269497657066	0.46439840594771	1.43842648027152
C	-3.17910586617475	-0.26742426736744	0.36124388644276
N	-4.04351328445573	-0.59892618650287	-0.74772766904431
H	-3.59081733226848	-1.21120380750785	-1.40862816301238
H	-5.22798734227598	0.25300301495478	-2.22921663228597
F	-4.86214574630839	-0.39912708702437	2.03136360124046
H	-3.27468963791088	0.79413380994362	2.22113950811800
H	-3.84166508560496	1.26280886351051	-1.77173128273010
H	-5.80419853070785	2.26975283246371	-0.87895081772173
F	-6.60110514495660	0.61564286464258	-0.09847305230139
H	-2.33657747891822	0.38575500967657	0.07227492626094
H	-2.76624429910259	-1.18159310244139	0.78616287313287

H	-5.36444470217230	2.09234217483408	1.61095821020379
H	-3.94946126533179	2.42484717796337	0.61466008978910

2C_ae, Benzene

C	-5.46439255775694	1.34535832473735	-0.40671275322651
C	-4.61023184393360	0.56334553600526	-1.38769882036572
C	-4.69554225973040	1.66712316447256	0.86278249278815
C	-3.96038283438289	0.46818011975203	1.43599009533764
C	-3.18218857803336	-0.26905210926166	0.36166104823553
N	-4.05277876605705	-0.60869071312082	-0.74485761683743
H	-3.57713937925811	-1.19719060270970	-1.41328614192904
H	-5.22710252859813	0.25665861801061	-2.23157904938558
F	-4.86924471004176	-0.40008052942405	2.02923106197003
H	-3.27697160946813	0.79201517188640	2.22308829831781
H	-3.83674951225228	1.25373743341914	-1.76376018847557
H	-5.80757595603087	2.26806438064282	-0.87815785822369
F	-6.60170009067343	0.61069224960866	-0.09251794492482
H	-2.34561606053857	0.38458457148100	0.06300246488328
H	-2.76279577520660	-1.17977152358182	0.78756216282761
H	-5.36074332397643	2.09840773151339	1.61073147333632
H	-3.94729421406144	2.42456817656880	0.61264127567198

2C_ae, Dichloromethane

C	-5.46111874798367	1.34717959911930	-0.40648056653742
C	-4.61194084053932	0.56161894156810	-1.38618946431549
C	-4.69436067754171	1.66859640504294	0.86253084551292
C	-3.95884792202140	0.47099988603385	1.43405278529819
C	-3.18462835946160	-0.27030045279495	0.36216494233936
N	-4.05947393747324	-0.61630709937325	-0.74298036501391
H	-3.56568536458626	-1.18500071573587	-1.41691671032900
H	-5.22680507848738	0.25954743654359	-2.23324185919909
F	-4.87564829746739	-0.40104953488782	2.02727413999243
H	-3.27892389311733	0.79014927408695	2.22463301113709
H	-3.83341581115214	1.24641687226924	-1.75785686767738
H	-5.81013647102105	2.26662108685726	-0.87757945188468
F	-6.60240712815739	0.60646122691206	-0.08731282970524
H	-2.35271523774442	0.38339947604091	0.05607553610680
H	-2.76001568966378	-1.17823572963299	0.78891211294049
H	-5.35717863588864	2.10389312914397	1.61040809704872
H	-3.94514790769326	2.42396019880671	0.61062667028620

2C_ae, Chloroform

C	-5.46220559298154	1.34657111867558	-0.40654185727484
C	-4.61136029314284	0.56224659388274	-1.38667709496017
C	-4.69474108213754	1.66815573288163	0.86258412182417
C	-3.95929334896749	0.47016517228390	1.43465220983419
C	-3.18388172206903	-0.26996009904369	0.36202196379200
N	-4.05739613911102	-0.61386581273181	-0.74357661736488
H	-3.56927973535162	-1.18882556498597	-1.41584845528196
H	-5.22678276430987	0.25873101815745	-2.23278863028397
F	-4.87357721400502	-0.40064089964621	2.02797353718214
H	-3.27826169902029	0.79079501997043	2.22411145819034
H	-3.83440649632615	1.24885854641081	-1.75961025007106
H	-5.80956444025038	2.26699237812385	-0.87768913372372
F	-6.60214133862827	0.60758978888032	-0.08896384754281
H	-2.35038772808573	0.38360251552426	0.05830904454741
H	-2.76109272467997	-1.17884780029905	0.78852393452616
H	-5.35823556567873	2.10224058039943	1.61051998846090
H	-3.94584211525448	2.42414171151632	0.61111962814605

2C_ae, DMSO

C	-5.46004027019351	1.34777867355927	-0.40641571489547
C	-4.61251902954012	0.56103628719522	-1.38571129330893
C	-4.69400634613466	1.66900110432919	0.86246067890706
C	-3.95840569896533	0.47184055860035	1.43345235729899
C	-3.18542556330307	-0.27066624967589	0.36233736930076
N	-4.06153138823021	-0.61873332392544	-0.74243295959256
H	-3.56198884779411	-1.18100172580365	-1.41799171472435
H	-5.22679225566405	0.26042402071247	-2.23373440248253
F	-4.87770299064433	-0.40152357496240	2.02673385879590
H	-3.27956040382336	0.78955914765111	2.22511274729180
H	-3.83241571605442	1.24399978588185	-1.75607823276059
H	-5.81081807041723	2.26622022414151	-0.87742817415050
F	-6.60271891396875	0.60523869311871	-0.08567783266853
H	-2.35505196408278	0.38309940746269	0.05388119195263
H	-2.75903052027168	-1.17766493777992	0.78933534372721
H	-5.35601997899488	2.10569005902390	1.61028598710446
H	-3.94442204191750	2.42365185047103	0.60999079020464

2C_ae, Water

C	-5.45993249280236	1.34783686642345	-0.40640514825838
C	-4.61257980711865	0.56098058381510	-1.38566234263010
C	-4.69396733572226	1.66904319114459	0.86245259561969
C	-3.95836388654853	0.47192673766521	1.43339117915640
C	-3.18550583932160	-0.27070293067385	0.36235795537508
N	-4.06173158231671	-0.61897462872648	-0.74238226174388
H	-3.56160380748831	-1.18058455073661	-1.41810373642803
H	-5.22679640702132	0.26051154642143	-2.23378055714447
F	-4.87792931015846	-0.40157404886008	2.02666386186224
H	-3.27963254749988	0.78949642050920	2.22516609789910
H	-3.83232824344453	1.24376602694029	-1.75590320943079
H	-5.81089339820544	2.26617464320464	-0.87740869459709
F	-6.60274962919578	0.60509127371764	-0.08550095290298
H	-2.35528263851782	0.38306604852518	0.05367500606067
H	-2.75893563683110	-1.17760999946924	0.78938045546564
H	-5.35588515546222	2.10589783689436	1.61027027372888
H	-3.94433228234499	2.42360498320515	0.60990947796799

2C_aa, Gas Phase

C	-5.47097876137534	1.35771671578347	-0.39663164729345
C	-4.63460383930349	0.54303454124519	-1.37071885329626
C	-4.69058217197634	1.68479067543584	0.86718312345597
C	-3.96596976609201	0.47994958112550	1.44734913392088
C	-3.21237294815964	-0.28593665344622	0.37154688212730
N	-4.04077668764428	-0.63324317080895	-0.76627576894878
H	-4.77106491100667	-1.27196299251279	-0.47406919915161
H	-5.25310156175406	0.24503545557483	-2.21705299073057
F	-4.88895919559416	-0.38279835869285	2.02327504239555
H	-3.28585197591205	0.79500246653540	2.24241018943031
H	-3.83090213298100	1.17825599172031	-1.75861464355216
H	-5.82559776009204	2.27650927485691	-0.86995914324974
F	-6.59544290824683	0.61283575472061	-0.06694387285313
H	-2.37940218688341	0.33231849748434	0.01968882817908
H	-2.78755609550398	-1.19195025862910	0.80313779726300
H	-5.35303574666132	2.12034202805009	1.61504972809058
H	-3.93906135081340	2.43809045155742	0.61237539421301

2C_aa, Benzene

C	-5.46777304438783	1.35886508416814	-0.39708363107202
C	-4.63561572516229	0.54333632105251	-1.37177151685213
C	-4.69016478862913	1.68436225410914	0.86664205708816
C	-3.96409810867657	0.48190457110385	1.44509850182922
C	-3.21148846825846	-0.28658526449046	0.37257294590398
N	-4.03891400629750	-0.63502795881056	-0.76859349012683
H	-4.76917185548015	-1.27451384488099	-0.47664504209218
H	-5.25533039085124	0.24622055432909	-2.21749832172931
F	-4.89287822797768	-0.38408634986696	2.02481826103629
H	-3.28610886631391	0.79412620171948	2.24140358021733
H	-3.83412859386410	1.18161311500981	-1.75771276218481
H	-5.82490066329782	2.27530679399864	-0.87020397731231
F	-6.59881790977794	0.61141689769341	-0.06420801828766
H	-2.37960442175684	0.33398955193962	0.02395965657417
H	-2.78724836685398	-1.19206242208324	0.80578078866336
H	-5.35080958826064	2.12296116460196	1.61447894732336
H	-3.93820697415394	2.43616333040658	0.61071202102137

2C_aa, Dichloromethane

C	-5.46520553534589	1.35962664112304	-0.39763325799476
C	-4.63592327646742	0.54354653338833	-1.37258148073794
C	-4.68995847733703	1.68377920727759	0.86618211581836

C	-3.96246759703109	0.48330502765411	1.44323102707051
C	-3.21073499206070	-0.28697772621433	0.37295537946078
N	-4.03856035797748	-0.63683115330604	-0.76973427825253
H	-4.77018869913780	-1.27421089480239	-0.47562956491596
H	-5.25607272242558	0.24788916303702	-2.21851586371766
F	-4.89550422637730	-0.38588554160999	2.02531983810379
H	-3.28623033986445	0.79331857424520	2.24071551914669
H	-3.83528789686134	1.18348505627119	-1.75630138942207
H	-5.82464253974548	2.27426487763406	-0.87048847473604
F	-6.60095645088947	0.60929627716916	-0.06279948061421
H	-2.38024755556263	0.33545264119371	0.02573083002949
H	-2.78600541373449	-1.19162754547630	0.80743026456367
H	-5.34926937002355	2.12458308279368	1.61399802496525
H	-3.93800454915829	2.43497577962195	0.60987079123262

2C_aa, Chloroform

C	-5.46601214110836	1.35938947187791	-0.39745296883172
C	-4.63583850322406	0.54348424744816	-1.37233035580435
C	-4.69001474239001	1.68398296520803	0.86631926238760
C	-3.96296352104884	0.48289411607800	1.44381016804623
C	-3.21096915863582	-0.28687208983384	0.37284941954897
N	-4.03861821647611	-0.63624637106262	-0.76941588028284
H	-4.76976081367094	-1.27441057952074	-0.47609602992213
H	-5.25586602890670	0.24739942050209	-2.21819077004609
F	-4.89467162722507	-0.38524839949929	2.02522558666411
H	-3.28618877845459	0.79356062995807	2.24093725805081
H	-3.83496781964096	1.18293608358104	-1.75675444963487
H	-5.82478891210774	2.27454953504838	-0.87040374000833
F	-6.60030080117749	0.60999437319739	-0.06319570677455
H	-2.37999168095207	0.33493197717432	0.02523745041512
H	-2.78648891072748	-1.19181039594128	0.80695605886132
H	-5.34974693245203	2.12408790038518	1.61414599565263
H	-3.93807141180176	2.43536711539920	0.61010870167804

2C_aa, DMSO

C	-5.46439247906829	1.35984366625463	-0.39782925126125
C	-4.63596882557855	0.54360517257920	-1.37282478146162
C	-4.68992592098310	1.68354943964797	0.86604032083576
C	-3.96194036361992	0.48372443625954	1.44263057868435
C	-3.21050816077213	-0.28708741216648	0.37302932788367
N	-4.03861616019593	-0.63747933583294	-0.77000138340918
H	-4.77084646297320	-1.27385246122004	-0.47492488139842
H	-5.25615000341853	0.24853532746011	-2.21894935280358
F	-4.89628580999275	-0.38658190428950	2.02541283352613
H	-3.28622267509678	0.79310886420153	2.24045302409097
H	-3.83546905054819	1.18397787063571	-1.75570910757643
H	-5.82451954260211	2.27395448045653	-0.87058389706051
F	-6.60159592812922	0.60847869678724	-0.06245545556035
H	-2.38056502742843	0.33597934642505	0.02609012227734
H	-2.78544700741062	-1.19140807557725	0.80788066908326
H	-5.34881555940755	2.12504428604724	1.61384841464013
H	-3.93799102277472	2.43459760233146	0.60964281950971

2C_aa, Water

C	-5.46430692299968	1.35986713244674	-0.39784980690490
C	-4.63597388539530	0.54360915434876	-1.37284966212548
C	-4.68992190006056	1.68352444358749	0.86602646239047
C	-3.96188761778667	0.48376542467832	1.44256990587170
C	-3.21048316884901	-0.28709579934326	0.37303626315685
N	-4.03862263056502	-0.63754711374317	-0.77002633821113
H	-4.77091706271226	-1.27381134446317	-0.47484452359344
H	-5.25616277405936	0.24859491589981	-2.21898866859041
F	-4.89636890192033	-0.38666009484451	2.02541343708077
H	-3.28622791319158	0.79308231508512	2.24043263293871
H	-3.83548832404133	1.18402361037222	-1.75565356025700
H	-5.82449983105839	2.27392640223133	-0.87059396484236
F	-6.60166241395646	0.60839983216940	-0.06242293333182
H	-2.38060084133402	0.33604117255232	0.02612290922549
H	-2.78537915825812	-1.19137997446135	0.80792358438542
H	-5.34876871825141	2.12509218538056	1.61383199376853
H	-3.93798793556052	2.43455773810340	0.60962226903856

3A_a, Gas Phase

C	-5.44550366907381	1.19405138930101	0.31043872632505
C	-5.04685627196144	0.62204667287035	-1.04003493234303
C	-4.24381475719489	1.73442572257075	1.07359974559617
C	-3.17608116294713	0.64684566895216	1.17025990220926
C	-2.80996938068274	0.11121088568718	-0.20689680939594
N	-3.99784740011896	-0.36954484238437	-0.88804153733569
C	-4.22561245533360	-1.62129288333338	-1.33397984798902
O	-5.21707289229398	-1.97559148041885	-1.91714388261915
C	-3.12530113730883	-2.68496977442765	-1.07398402400711
F	-2.86514550901390	-2.81265970828823	0.23353401696096
F	-1.97869959906623	-2.36815585459636	-1.69036327515819
F	-3.51868368051075	-3.85710403711291	-1.52685605893651
H	-5.89986311520877	0.15593235422599	-1.52382172899033
H	-4.68715295270579	1.44103705409058	-1.67216805790719
H	-2.37549898684093	0.91280084684762	-0.81458403945809
H	-2.06993018231161	-0.67588380253113	-0.13281302376129
H	-6.19322702298796	1.97862286499567	0.16627504422356
F	-6.04385406056683	0.18887523748600	1.05421027781911
C	-4.63782074415546	2.27967464905873	2.43699558267764
H	-3.84309615488841	2.55475573782889	0.46589452048767
H	-3.55167028597961	-0.17525425345962	1.78423815772100
H	-2.28317816711490	1.04301915620376	1.65564776290330
H	-5.09925025397934	1.49893510211888	3.04114830953922
H	-5.34858597527976	3.10175902183297	2.34325553198612
H	-3.76131418247433	2.64950427248198	2.96956963945247

3A_a, Chloroform

C	-5.44369926350448	1.20473772468360	0.29982514496613
C	-5.04227857728837	0.62967471046749	-1.04640457190754
C	-4.24608460734149	1.73069695128229	1.07490769293952
C	-3.17646668648884	0.64488650494288	1.16857402569450
C	-2.80850958406874	0.11204771956569	-0.20855549639792
N	-4.00173408971383	-0.37525681825940	-0.88570670158305
C	-4.23011205294670	-1.62102856829969	-1.31060384622281
O	-5.23647286475803	-1.99720046343221	-1.87210811714619
C	-3.12051355073061	-2.68047438312251	-1.07057651347274
F	-2.84579356509507	-2.82228900670422	0.23052306345731
F	-1.98440275851261	-2.36502530486500	-1.70126839072878
F	-3.52019522620571	-3.85483300145713	-1.52767613633381
H	-5.89874890757976	0.18167881438033	-1.53907749329057
H	-4.66127783982986	1.44267488406451	-1.67052827421374
H	-2.38727530269959	0.91458232498729	-0.82081018178616
H	-2.06293736535796	-0.66963933187021	-0.13508418803810
H	-6.18522705950115	1.99198044228410	0.15119680137691
F	-6.06366747685681	0.19376825199481	1.03652645734538
C	-4.64188620575039	2.27051071266156	2.44023536600564
H	-3.84438509942719	2.55438001258944	0.47391683611339
H	-3.54399572016363	-0.17864612428929	1.78621582034447
H	-2.28264542695161	1.04522897485950	1.64778470273258
H	-5.08662429555244	1.48486229170224	3.05140932500246
H	-5.36429679929947	3.08258254031724	2.34702590740840
H	-3.76579967437562	2.65314014151671	2.96463876773472

3A_e, Gas Phase

C	-5.46994851404299	0.99172527944880	0.12346310123420
C	-4.84204739253304	0.67393775608984	-1.22779824263646
C	-4.43604536680214	1.51622872570905	1.12724398864466
C	-3.28323391945578	0.51964996514373	1.20785510429205
C	-2.71006306441073	0.16506614939735	-0.16067110652153
N	-3.77113216399183	-0.28952678390241	-1.04538969195185
C	-3.73532965781814	-1.55146138128899	-1.51935478576539
O	-2.87301966293352	-2.35559494819704	-1.27631101282645
C	-4.88817675729607	-1.99492696312596	-2.46252639870861
F	-4.96682136647462	-1.21379158691013	-3.54646699913479
F	-6.07477424709121	-1.95197439660728	-1.83944681542055
F	-4.68797026880929	-3.23188735998188	-2.86635153689283
H	-5.58730815750376	0.29115756614045	-1.91374787069945
H	-4.41600887406090	1.57233206138346	-1.68147297443753
H	-2.23282725050876	1.04847799368545	-0.59258716433533
H	-1.96639848877443	-0.61985687408384	-0.06443115341360
H	-6.26194590666363	1.73062723421934	-0.00581529410191
H	-5.93391230973492	0.07747763518487	0.50255457405351
H	-4.90490018835421	1.55227846690390	2.11337272844201

C	-3.96205267592196	2.92479909336236	0.78355682668994
H	-3.62131981697559	-0.39670948008015	1.69911898704476
F	-2.25708594883053	1.04171009911502	1.97482522323784
H	-3.44271679292122	2.96965933604965	-0.17390334750346
H	-3.28131990615917	3.29873266537245	1.54539883515068
H	-4.81699130193155	3.59928974697194	0.72752502556011

3A_e, Chloroform

C	-5.47161901511679	0.99504143246315	0.13941084967410
C	-4.85328388888580	0.66208441065391	-1.21146084602758
C	-4.43289150098280	1.52841889974662	1.13329597215757
C	-3.29325354077765	0.52122690448020	1.21782904475985
C	-2.71957423267388	0.15359726404186	-0.14665844795617
N	-3.78559416935728	-0.31053309341892	-1.02389900578815
C	-3.73088383773239	-1.54500186964658	-1.53440080941166
O	-2.84830388059121	-2.34982239316038	-1.32945469185661
C	-4.88366223746635	-1.98820835034600	-2.47841054491649
F	-4.97279796910409	-1.20168173466550	-3.55558970597640
F	-6.06869594930127	-1.97522160072461	-1.85773955166766
F	-4.66491457990948	-3.22220918782207	-2.89908426787899
H	-5.60580313837089	0.28017353004706	-1.88954311765195
H	-4.41716181015677	1.54991631157020	-1.67406995034836
H	-2.24918682877707	1.03191552107346	-0.59474462448687
H	-1.97350393994786	-0.62689244690553	-0.03989779723189
H	-6.25924606053761	1.73624416261477	0.00154242741446
H	-5.93669852333714	0.08844152973031	0.53452035951182
H	-4.90091533727588	1.57596350203996	2.11898213833468
C	-3.95283450128237	2.93033613649995	0.77033516263194
H	-3.63577580826556	-0.38547341166631	1.72088936994895
F	-2.25120129299318	1.03936188104398	1.98053043730467
H	-3.42325526765649	2.95814094349204	-0.18230390472274
H	-3.28327018389972	3.32031639554312	1.53488686725085
H	-4.80902250560045	3.60128526331530	0.69367463693266

3B_a, Gas Phase

C	-5.42993332401969	1.27050777479790	-0.42109681956800
C	-4.57933056537978	0.56353824852171	-1.45975585950983
C	-4.62229783471579	1.66783935907157	0.80558460347840
C	-3.96016575166890	0.42054964612499	1.39417925295590
C	-3.15949221423096	-0.35319113025992	0.36403645571518
N	-4.03188843702179	-0.67300157313922	-0.81945813267207
H	-3.51574860241020	-1.22744426467661	-1.50196901726643
H	-4.82982810121469	-1.23493792208458	-0.50669733305933
H	-5.16897357885616	0.26731148837323	-2.32345457008424
H	-4.72614377888516	-0.22439417251289	1.83200405728748
H	-3.28836701997459	0.70183911258333	2.20489754823579
H	-3.73971900607031	1.18034439606406	-1.77543579638831
H	-5.92600442739999	2.13140462100280	-0.86999015318988
F	-6.40551201571051	0.35201371652500	-0.05243346058851
H	-2.32614746368784	0.23279040925678	-0.02306425871710
H	-2.78112282991039	-1.29416657830626	0.75531464306293
C	-5.48048956876374	2.39339660612288	1.83171006973522
H	-3.84064480685120	2.35104762979825	0.45477102910969
H	-6.31018934269652	1.76786448377467	2.15959860529631
H	-5.89033112473104	3.31429270120813	1.41741387568128
H	-4.88821020580075	2.65790544775419	2.70697526048551

3B_a, Water

C	-5.42961002696303	1.28560794127629	-0.42289934209994
C	-4.60255926837565	0.53641299971704	-1.44279906055647
C	-4.62600571603250	1.65868183484938	0.81135668790776
C	-3.97747680354756	0.40488625028830	1.39542226982493
C	-3.15773069171595	-0.34192985035938	0.36299631829201
N	-4.01107511486179	-0.67471447162848	-0.81545804706410
H	-3.46216694388976	-1.18161573532329	-1.50908435583764
H	-4.76122146855643	-1.30674090510520	-0.52834526899418
H	-5.20679014971246	0.21761053325437	-2.28666377401970
H	-4.74947609947422	-0.25466526232430	1.79960246247252
H	-3.32146906394580	0.67663876691606	2.22207161629635
H	-3.78255498701746	1.16000104241456	-1.79332165323963
H	-5.86499630887534	2.16743588481677	-0.89222151628684
F	-6.48449392262340	0.44503484216286	-0.04969370231058
H	-2.33879030895413	0.26824576794087	-0.01465409242968

H	-2.76190905732408	-1.27634850053656	0.74877398651782
C	-5.47347154565038	2.39772683924557	1.83557170194924
H	-3.83380091750239	2.32794295344123	0.46056399671348
H	-6.29399369973183	1.77112607873121	2.18621480841330
H	-5.89409690654699	3.30864682400015	1.40795805791729
H	-4.86685099869885	2.67552616622257	2.69772893003406

3B_e, Gas Phase

C	-5.48217838190564	1.17833932163102	-0.45670607257811
C	-4.64392435994611	0.44290898196898	-1.48336585515496
C	-4.68425574364962	1.56888309494579	0.79496358552282
C	-4.02799265783078	0.31614120162283	1.36670457170543
C	-3.20073098124849	-0.44522904717976	0.33343503189042
N	-4.05830619027624	-0.78776451272256	-0.84503556370683
H	-3.51190893451024	-1.31386274836910	-1.52805133506126
H	-4.82146546982430	-1.40093677064449	-0.54702129235142
H	-5.22861474988706	0.12233497811855	-2.34215959572921
H	-4.79833467595441	-0.34872606633500	1.77410541846443
F	-3.16846810567232	0.62670353025617	2.38390326918406
H	-3.80002572390437	1.03666157874816	-1.83078285341724
H	-2.38051175654914	0.17084727631649	-0.03080273151909
H	-2.80357524581453	-1.36500045131837	0.75519647353925
H	-5.39703898328103	1.92192249337294	1.54232779701612
C	-3.68502005704523	2.69052166052091	0.53614262148349
H	-6.34401858897639	0.55999514402409	-0.18804235288322
H	-5.88491207285102	2.07509098888080	-0.92808385068472
H	-2.93230989293583	2.43610367564103	-0.21174714719746
H	-3.16207398312057	2.95314028826371	1.45311775819513
H	-4.21010344481666	3.57849538225780	0.18557212328237

3B_e, Water

C	-5.48032590328183	1.18146509215347	-0.45036768207273
C	-4.64361230532196	0.44029789211908	-1.47369301028507
C	-4.68301548584132	1.57365471473723	0.80018324020721
C	-4.03891402873951	0.31262970872776	1.35679468683656
C	-3.20570588427502	-0.43757615473718	0.32883333412891
N	-4.05319650336552	-0.77683622283657	-0.84257682377578
H	-3.49618313543653	-1.28763911211117	-1.52748604537598
H	-4.80181804273951	-1.41298919694937	-0.56021190971693
H	-5.23806448331125	0.11042463746066	-2.32026645448759
H	-4.81182864558465	-0.35124258036257	1.75107809143053
F	-3.18787430468499	0.61818818730368	2.40918738623420
H	-3.80744317098898	1.03365481414859	-1.83794482230889
H	-2.38367253183788	0.17637862897771	-0.03297418621403
H	-2.81565612509748	-1.36062945505981	0.74662665799525
H	-5.39385898752156	1.92898358176796	1.54845998130086
C	-3.67831114369619	2.68839096004206	0.53224145087579
H	-6.33130593175118	0.55563670534725	-0.17105474339753
H	-5.88434643829103	2.07661731768570	-0.92268873297183
H	-2.93281294672146	2.41489957670473	-0.21507698334609
H	-3.15304662965084	2.96131139274250	1.44610492911419
H	-4.20477737186129	3.57094951213827	0.16850163582895

3C_ae, Gas Phase

C	-5.43273054528343	1.27508614414173	-0.42583579207112
C	-4.58737989178118	0.50369992433671	-1.42511929822128
C	-4.62570549239145	1.64643764571937	0.81145730417821
C	-3.97643674972745	0.38958359124345	1.38723726046660
C	-3.14728402263557	-0.32344273123004	0.32711231727972
N	-3.99324588189967	-0.65962600722665	-0.80290843199519
H	-3.49825800105744	-1.21862887510547	-1.48220651257219
H	-5.21632446012142	0.18670984922063	-2.25689397806749
H	-4.75852990191162	-0.28464036535231	1.74392069741675
H	-3.34874176839948	0.65458461046276	2.24032095739376
H	-3.83778307352100	1.21315215319649	-1.81678134884713
H	-5.82980255526458	2.17590674259206	-0.90243887933864
F	-6.52614288761513	0.50821696437768	-0.04945599052877
H	-2.30461914169936	0.32867966431813	0.03962463640245
H	-2.72759031521113	-1.24384053002391	0.73331431501944
C	-5.46537738075097	2.39661402492682	1.83359299437116
H	-3.82610129568476	2.30900968434612	0.45860413823267
H	-6.29790053156176	1.78010441296871	2.17207125214579
H	-5.87448970237891	3.31533022757170	1.40978890602288

H	-4.86395640110369	2.66454286951601	2.70308545271236

3C_ae, Water

C	-5.42480750960847	1.28212819555003	-0.43094781088892
C	-4.58562956280895	0.50419108858681	-1.42465473934977
C	-4.62985371832592	1.64452392787015	0.81307805399887
C	-3.97534228598678	0.39054714954397	1.38984588478239
C	-3.15793500227226	-0.33161006697156	0.32759125339593
N	-4.01275546869977	-0.67694855388592	-0.80115373778127
H	-3.48545853626173	-1.19875695063607	-1.48904539322007
H	-5.20542638738694	0.20444201667982	-2.26964859031762
H	-4.75066176397754	-0.27978740614924	1.77128958210396
H	-3.33534370723925	0.66439726540043	2.23065266724861
H	-3.81764021791224	1.19888042353649	-1.79855621391012
H	-5.82700493289081	2.17925519331000	-0.90508948957409
F	-6.53303565615856	0.50374879878935	-0.06027034599257
H	-2.32397550719620	0.31898095237658	0.02082014332047
H	-2.73070864532139	-1.24793021532323	0.73477508459600
C	-5.46939315772850	2.39517886280264	1.83533011915044
H	-3.83220481707878	2.30881899172346	0.46182679102827
H	-6.28942610527844	1.77340289603948	2.19652548936699
H	-5.89328304854553	3.30345080105538	1.40332827585521
H	-4.85851396932194	2.68056662970142	2.69279297618727

3C_aa, Gas Phase

C	-5.43512653895797	1.28716503891701	-0.41865075640972
C	-4.60059379971269	0.49761084283159	-1.41435286608239
C	-4.62605237816426	1.66360445568851	0.81506669827867
C	-3.97174938701846	0.41320318231285	1.40292368615557
C	-3.19406746821431	-0.35496124317561	0.33591637592508
N	-3.99120775664469	-0.68316956110784	-0.83593219269018
H	-4.71606408850765	-1.34552697058413	-0.58705343635762
H	-5.21745305479158	0.20805593490653	-2.26512988043259
H	-4.75202754862820	-0.23255083069261	1.81627954701119
H	-3.31367018854803	0.69385407277780	2.22870885286405
H	-3.80685407996603	1.15128417958178	-1.79150238351593
H	-5.86414079201468	2.17807794199165	-0.88550496065276
F	-6.50720603020997	0.48197058895662	-0.02951801244121
H	-2.34630085172088	0.25109774945562	-0.00251555042556
H	-2.77958757503326	-1.27592260920923	0.74624333111640
C	-5.47225899989343	2.40571804355601	1.83740681878105
H	-3.83085181039791	2.33022095584827	0.45954518377774
H	-6.30176458677099	1.77952441384137	2.16614611238155
H	-5.88596091491489	3.32396516267443	1.41773392198506
H	-4.87717214989013	2.66901865142939	2.71243951073159

3C_aa, Water

C	-5.42593561576171	1.29646073798278	-0.42565985903771
C	-4.60083097374252	0.49518107824316	-1.41640593860953
C	-4.62751868295063	1.65941257476311	0.81589976923416
C	-3.97208200310026	0.40886895693149	1.40130243482023
C	-3.18917174395717	-0.35326843798394	0.33613519462122
N	-3.99429565689742	-0.69128647308755	-0.83375658298782
H	-4.72797325002941	-1.33584205299921	-0.55923478491788
H	-5.22258678383465	0.20784969795237	-2.26453869148822
H	-4.74728910413506	-0.24464565326865	1.81320309640683
H	-3.31417796813334	0.69096508555807	2.22637299451561
H	-3.80645532180466	1.14622362415130	-1.79425857212470
H	-5.84066930295810	2.18985406449762	-0.89667806684768
F	-6.52461222635328	0.50201248805689	-0.04618944214177
H	-2.35038424437981	0.26179579107095	-0.00665726262777
H	-2.76954062920548	-1.27050650031459	0.74944127679323
C	-5.47530709829161	2.39909463877786	1.83921852158083
H	-3.83286010309474	2.32836596025615	0.46581723625584
H	-6.29128163458974	1.76520049938335	2.18870912446290
H	-5.90539481017395	3.30665914734945	1.41234345489977
H	-4.87174284660644	2.67984477267940	2.70318609719245

3C_ee, Gas Phase

C	-5.47320797736719	1.18593996695423	-0.46722318685084
C	-4.61478815024316	0.41997047428984	-1.46649687256561
C	-4.68671338317045	1.56684163253868	0.79471339727986
C	-4.02727124453835	0.30728996176333	1.34544980774821
C	-3.20185469055754	-0.42160755296042	0.29776584928666
N	-4.06527740977922	-0.76030116113580	-0.81901044998232
H	-3.58008505598180	-1.34715799340794	-1.48216181837856
H	-5.22965869636407	0.10171482207060	-2.30862639299749
H	-4.79684155535028	-0.37153020981139	1.72111961920910
F	-3.20390200125609	0.62935445130645	2.41534656525428
H	-3.82971767452534	1.07943574592454	-1.86609627406642
H	-2.35093662670425	0.21465343346338	0.01365549894912
H	-2.80355366854089	-1.33520358002211	0.73837649556328
H	-5.39421917585005	1.92176101900869	1.54869472275746
C	-3.68167327515284	2.68492887779352	0.53701154841212
H	-6.32124059320158	0.55382175149572	-0.19400016270342
H	-5.87275343401015	2.08967038645098	-0.93183987471684
H	-2.95658983117381	2.42002932758156	-0.23197297321117
H	-3.13201314879242	2.92619275515770	1.44508450997548
H	-4.20376240744052	3.58248589153846	0.20349999103713

3C_ee, Water

C	-5.47456942016063	1.18541221931488	-0.46238383681231
C	-4.61883260278201	0.41839706379059	-1.46229535805964
C	-4.68583698271775	1.56796397354596	0.79836114654518
C	-4.03533094782791	0.30361129525483	1.34144158197198
C	-3.20578965228007	-0.42381686295403	0.29848438580534
N	-4.06892896611223	-0.77093774814533	-0.82055038104946
H	-3.54933251285421	-1.31997050421590	-1.49284604900156
H	-5.23147062517967	0.10605860266949	-2.30796884244626
H	-4.80523909212942	-0.36994373692431	1.72381957644426
F	-3.20530793768391	0.61901278924496	2.42389534596977
H	-3.82680878697355	1.07098248749156	-1.85526612682839
H	-2.36289256601771	0.21627322229186	0.00297480208443
H	-2.80008516424044	-1.33463801346854	0.73833281173697
H	-5.39314291524040	1.92521058316062	1.55113360937759
C	-3.67837569915360	2.68257697306664	0.53262210150879
H	-6.32615742925070	0.55934826056607	-0.18416762934390
H	-5.87140881769420	2.08964657856530	-0.92755995282975
H	-2.95076458245002	2.40872341250603	-0.23100669999868
H	-3.13316211636864	2.94021254145393	1.43978415280498
H	-4.20262318288293	3.57416686278540	0.18648536212069

3C_ea, Gas Phase

C	-5.49136040192062	1.19775063619121	-0.45211701106064
C	-4.64582031895676	0.39999520237919	-1.44363859843824
C	-4.69102163853536	1.57677249552535	0.80232959634510
C	-4.03851628832382	0.31677745824543	1.36324689036289
C	-3.23770975764416	-0.44328049695848	0.31497671822600
N	-4.03445734317332	-0.78004437520561	-0.85162672874705
H	-4.74596358374184	-1.45969929613298	-0.61055037531543
H	-5.24792556861204	0.09214111609619	-2.29883625823956
H	-4.81607599204846	-0.34438958221648	1.76083456239574
F	-3.20686686130888	0.64358020678377	2.42571660995357
H	-3.84009334143798	1.02415849245636	-1.84021647052346
H	-2.39210706850722	0.17079677770292	-0.00648411076017
H	-2.82557884144197	-1.35153120126951	0.75420209478400
H	-5.38776336938655	1.94632761433952	1.55918690077586
C	-3.67613810380842	2.68237703571879	0.52859207836361
H	-6.35406424245113	0.58710909275344	-0.16822047651786
H	-5.88261844739962	2.10307562406960	-0.92274584062752
H	-2.95619091141360	2.40521169250056	-0.24129441291045
H	-3.12075594896555	2.92635350659822	1.43237102286791
H	-4.19175197092270	3.58195800042253	0.19071380906569

3C_ea, Water

C	-5.48836417334949	1.19452598031831	-0.45075078563799
C	-4.64281819382989	0.40392153118010	-1.44590526012639
C	-4.68864832283503	1.57560719705830	0.80370138079863
C	-4.04407728758583	0.31099472539645	1.35476730220699

C	-3.23308214682797	-0.44176397702989	0.31176130044816
N	-4.03457560748554	-0.78494513804273	-0.85453965461771
H	-4.76485014533213	-1.43557561630561	-0.58360648577510
H	-5.24998862570976	0.09858664584666	-2.29821139364713
H	-4.81849121585658	-0.35133640051054	1.75065532662369
F	-3.20663628758156	0.63058938333604	2.43197041937932
H	-3.83594218605517	1.02871029603078	-1.83878937881657
H	-2.39323096762577	0.17887335475329	-0.01260217352434
H	-2.82193856325568	-1.34934095915379	0.75369147999659
H	-5.38852359443599	1.94263626642284	1.55864627866161
C	-3.67441608613245	2.68157947184135	0.52735368118891
H	-6.34588509790100	0.57822064459785	-0.16554154932678
H	-5.88189797002101	2.09916658166736	-0.91985856514066
H	-2.94634910893141	2.39739947063554	-0.23258913333952
H	-3.12943013265359	2.94463694599416	1.43305972245614
H	-4.19363428659414	3.57295359596358	0.17322748819215

4A_a, Gas Phase

C	-5.32063049336662	1.67757617801567	0.54796178642108
C	-5.16179435138591	0.77450368828591	-0.67685186635826
C	-4.01139050504999	2.43055284505668	0.80187857055429
C	-5.79317068589127	0.88368933013287	1.76085641478462
H	-6.08655341971812	2.41524111220052	0.29688786434739
C	-2.80317077352875	1.51293281321855	0.86119864526217
H	-4.07112899291601	3.00688051535896	1.72608101518220
H	-3.83736200362426	3.14125731479495	-0.01194535113079
C	-2.75649990430487	0.56785138258753	-0.32653760472106
F	-2.83046440454224	0.76673196359218	2.02920826177716
H	-1.88187574998132	2.09955102009349	0.88186675901916
N	-4.02593328619052	-0.11315324256089	-0.51160864258013
H	-1.97643788256147	-0.17460577463641	-0.18899729125547
H	-2.53018559742452	1.15494066009562	-1.22374296422168
H	-4.98638082003521	1.38338801940143	-1.57062677794177
H	-6.06866098068237	0.20364657965984	-0.84208762166933
C	-4.03129348218651	-1.46096947224668	-0.55587417657582
O	-3.05459609759532	-2.15636827164205	-0.44882367515009
C	-5.39485364685087	-2.17151904354664	-0.77630741785036
F	-6.27743830012572	-1.86883728772563	0.18342982719092
F	-5.22362921638940	-3.47731317014679	-0.77265618287014
F	-5.93703534936831	-1.82944326161659	-1.95269890272270
H	-5.85115663260297	1.52499339863893	2.64020345453362
H	-5.10826006943900	0.06952716050039	1.99298063900698
H	-6.77937735423844	0.45651554248816	1.58002523696800

4A_a, Chloroform

C	-5.31766957395031	1.67220975639895	0.54856495027434
C	-5.16195605524306	0.77886323094643	-0.68331662042696
C	-4.00923735882080	2.42362180771485	0.80907485237546
C	-5.79503978964420	0.87308704400748	1.75615150397726
H	-6.08190645778595	2.41136567161726	0.29993900002490
C	-2.79700556416393	1.51419698433495	0.84796800667268
H	-4.06759034642810	2.98926444761981	1.73997603522501
H	-3.83845699595225	3.14348155829948	0.00353457338990
C	-2.75466561919164	0.57771367791495	-0.34387029645366
F	-2.81031753053168	0.74738534065129	2.01527269340536
H	-1.87804546290457	2.10120004790520	0.87002193947089
N	-4.02420350232441	-0.11405420481088	-0.51892134088829
H	-1.96060359600492	-0.15160212884323	-0.22422301493404
H	-2.55533264944513	1.17386742594316	-1.23904381347914
H	-4.97586074826301	1.39033714701736	-1.57058618809102
H	-6.07101831216350	0.21351311669258	-0.85412422821149
C	-4.04045277047807	-1.45028275249828	-0.52010704971566
O	-3.07210171561068	-2.16210795109172	-0.36036483438203
C	-5.39875118880840	-2.16171959982549	-0.77139949792752
F	-6.30365598957256	-1.86811620777172	0.16712741998308
F	-5.22099405641766	-3.47200359636650	-0.76112249674645
F	-5.91466616771453	-1.83127184857636	-1.96018225172213
H	-5.85103283001540	1.51234331060074	2.63751756880159
H	-5.11485619280938	0.05297223030334	1.98499389375162
H	-6.78385952575586	0.45330549181633	1.57093919562628

4A_e, Gas Phase

C	-5.35777216886636	2.14518523542154	0.20158376920497
C	-5.34693593346625	0.78647428020659	-0.49159763750300
C	-4.10066339760125	2.93712933771292	-0.09981952860126
H	-5.47813708325856	2.00666543834861	1.27926349086172
F	-6.46246482039927	2.84301304121939	-0.25184717749973
C	-2.84372070266521	2.12122971934170	0.20139990037274
H	-4.10453767183705	3.86795585650365	0.46950739038770
H	-4.11317864341616	3.20270063182780	-1.16250520616640
C	-2.91286966732125	0.79433546030227	-0.54790141083797
H	-2.82287369587951	1.88468642939930	1.27067445074771
C	-1.57272771826979	2.86826789616558	-0.16578104110474
N	-4.11720827065508	0.07501337997963	-0.18473518649012
H	-2.03137151542708	0.19644974373687	-0.34857530101691
H	-2.94160530362690	0.98635451201972	-1.62651350902818
H	-5.40529392681538	0.95281782730924	-1.57157815340519
H	-6.19522815992841	0.18572229363973	-0.17905021514205
C	-4.17923939212575	-1.09188453989332	0.48910734624515
O	-5.19481961476339	-1.62677839118759	0.85090059926453
C	-2.83497414759517	-1.80199544775385	0.81502627440207
F	-2.05486357875660	-1.03832040410382	1.59394500464767
F	-2.14602191405175	-2.09128829573240	-0.29533399350915
F	-3.06625366944261	-2.92891689548849	1.45506525667232
H	-1.55171789180561	3.09051285596983	-1.23465623482294
H	-0.69042490484371	2.27316480815756	0.07318578807235
H	-1.50144620718186	3.81086522689756	0.37570532424872

4A_e, Chloroform

C	-5.35106385837162	2.15513701238145	0.20535257557451
C	-5.34334546054104	0.78956971776552	-0.47206373036282
C	-4.10221818390316	2.94713743317910	-0.11506326691272
H	-5.47197834823492	2.03684346264057	1.28395524866208
F	-6.46919174507816	2.84226701739701	-0.25844229704589
C	-2.84670817295655	2.13663083672500	0.20610066886228
H	-4.10488519314132	3.88612859414645	0.44041386309591
H	-4.11188076178314	3.18874236141204	-1.18327588115011
C	-2.90687763078741	0.79698796368936	-0.51991250170485
H	-2.82972378627305	1.92427884649393	1.28021611651844
C	-1.57686376868127	2.87972824649759	-0.17380853634462
N	-4.11477770476570	0.07919248131619	-0.14778666331806
H	-2.02418009429955	0.20766967067867	-0.30224975783633
H	-2.93416562769589	0.96528111142800	-1.60126218055171
H	-5.38803504628353	0.93969491323417	-1.55437730414363
H	-6.19762684265222	0.19920184629608	-0.15807315327567
C	-4.17655968270690	-1.09906556620512	0.48247238359643
O	-5.19546317301599	-1.65888761025745	0.82284093317892
C	-2.83594088878822	-1.82066575472464	0.79825712046023
F	-2.05437759798090	-1.08932173545738	1.60074397282915
F	-2.14577323046783	-2.09509972711304	-0.31316402076571
F	-3.08128749526013	-2.96550769371339	1.41165713121146
H	-1.55799059989839	3.08259695570422	-1.24659826288852
H	-0.69401313050835	2.28980573376228	0.07613971938984
H	-1.51142197592471	3.83101388272343	0.35339782292140

4B_a, Gas Phase

C	-5.37169444385011	1.35528190571695	-0.50508886231781
C	-4.52838229875610	0.55984245780691	-1.49448660949698
C	-4.57292956947122	1.65368441639660	0.77149693176156
C	-3.88331817734129	0.43050050898243	1.34472419152197
C	-3.04325173473630	-0.28304686589981	0.30363088073081
N	-3.95316630422718	-0.65735132212569	-0.82276195538503
H	-5.11966231059244	0.21772651454938	-2.34119911542550
H	-3.67652206273211	1.13240310812396	-1.86009388195680
H	-5.22917555516508	2.07968057712311	1.53007641263202
H	-3.80054195989152	2.39983062346881	0.56624771195686
H	-2.26523505323487	0.36535458256169	-0.09579741135274
H	-2.60202638268324	-1.19005127185470	0.70804894713847
H	-3.46056796552787	-1.23687898500314	-1.50156231604703
H	-4.71592074066647	-1.22232037249246	-0.43509697764310
F	-4.82448109680916	-0.49817478588784	1.76703029791886
H	-3.26134342572151	0.69261647934424	2.20011026509318
H	-5.57496878274714	2.30890418022531	-0.99576161262730
C	-6.71607002687500	0.68271521014707	-0.23428042415939
H	-6.61831849773341	-0.30736645561746	0.21204202407120
H	-7.29599502785190	0.59555044023744	-1.15238114002181
H	-7.29471858338611	1.28020905419721	0.46879264360857

4B_a, Water

C	-5.37736418422571	1.35616249547129	-0.50423711485264
C	-4.52405938058506	0.57344177632356	-1.49164422518121
C	-4.57338710614793	1.66524181372514	0.76308716775306
C	-3.88556486944607	0.44951169668065	1.34811368030686
C	-3.08893093212529	-0.31063158693248	0.31378230003738
N	-3.95995243151230	-0.64758675543777	-0.84281524966180
H	-5.10078436035640	0.24465727540114	-2.35206270196430
H	-3.66948424199911	1.15580693826785	-1.83086596168978
H	-5.21519311092090	2.11397487503384	1.52161411864459
H	-3.79036658289572	2.39106840550385	0.53061991704575
H	-2.27595892252608	0.30796411151598	-0.06111720613518
H	-2.69084230796979	-1.23475615023208	0.72114458686847
H	-3.42546902008197	-1.18261618844327	-1.52639925603218
H	-4.71552374685866	-1.26279363219915	-0.53372783909223
F	-4.83843153285533	-0.43213451574790	1.87213903943397
H	-3.22430282380034	0.73187409823968	2.16572634372957
H	-5.59731083708649	2.30665359519032	-0.99281267693726
C	-6.70631066123006	0.66009271149926	-0.22259887876559
H	-6.57538493641059	-0.33727618087267	0.19764620477844
H	-7.28901489176716	0.57132995290708	-1.13925604443253
H	-7.28465311919906	1.23912526410570	0.49735379614662

4B_e, Gas Phase

C	-5.45427792442840	1.34054222173241	-0.48606921618633
C	-4.61400460692865	0.56956749656253	-1.49973023526816
C	-4.66757544975187	1.65109878690303	0.77020687426642
C	-4.05585303333124	0.38816688461368	1.37893536154146
C	-3.21487832350824	-0.34395786606981	0.34725265768233
N	-4.05003676386629	-0.66255740300983	-0.86354918554985
H	-4.86195256548007	-0.28485535757758	1.69573507149572
C	-3.20227317333190	0.71771046731262	2.59764594103193
F	-5.84028530618167	2.49283348438796	-1.11313756441456
H	-6.35825059608016	0.77193258585373	-0.24104086152165
H	-5.20280754955651	0.27677331675625	-2.36520150878695
H	-3.77182042656877	1.18190978625414	-1.81999351409065
H	-5.32041868604834	2.13545214549655	1.49642891472311
H	-3.87531937310336	2.36313075607136	0.52034366923575
H	-2.38844751283940	0.27640918799913	-0.00193298859733
H	-2.81920282881695	-1.28250917482564	0.73019250380132
H	-3.49321507290436	-1.17580384174073	-1.54813363376696
H	-4.81219021224860	-1.28511820041742	-0.58390745076158
H	-2.39650891501684	1.40412581495057	2.33269606184782
H	-2.76125346731547	-0.17674666349475	3.03655986541359
H	-3.81037821269287	1.19914557224180	3.36178923790458

4B_e, Water

C	-5.44995525996082	1.32742739227508	-0.47733513342176
C	-4.60933406494213	0.56821195397838	-1.49032526935513
C	-4.66287082082782	1.65943585197133	0.76894113185861
C	-4.05842543516503	0.39319290477415	1.37719174506663
C	-3.22248580193812	-0.34138646172875	0.34427313266079
N	-4.04684513899998	-0.65294286074162	-0.85941991408153
H	-4.87077040473931	-0.27260229480721	1.68616815405815
C	-3.20615263416743	0.71820110161171	2.59570139662441
F	-5.86727856927594	2.49385253391228	-1.10430755645411
H	-6.34627574459195	0.75533023435785	-0.22830031315824
H	-5.19914506108758	0.26637034353879	-2.35033566026013
H	-3.76895618687641	1.18113530643019	-1.81208070828398
H	-5.31280482353177	2.14809799782962	1.49542585482575
H	-3.86199783746736	2.35655169851828	0.50471867432618
H	-2.38960287126123	0.27225186874861	0.00120358654818
H	-2.84080920589548	-1.28384595468361	0.72767740478070
H	-3.48028513652347	-1.15289334036473	-1.54474964065089
H	-4.79977619875935	-1.29380537539151	-0.60070113461692
H	-2.39012141932873	1.39056709522011	2.32409698735235
H	-2.77676136327512	-0.18514766408717	3.02913619171132
H	-3.81029602138493	1.20924766863822	3.35811107046962

4C_ae, Gas Phase

C	-5.37353495094612	1.36370955778750	-0.51497385403349
C	-4.48941911228446	0.57451648541974	-1.48427524083930
C	-4.57393352935595	1.67182505992033	0.75586286556581
C	-3.89256255788304	0.44930122160945	1.34648342412022
C	-3.12549160168623	-0.31897181367745	0.28491712915893
N	-3.98542771212175	-0.62282829390299	-0.83758517050799
H	-5.07335381883360	0.28069174630407	-2.35774133900973
H	-3.67265215055984	1.22980977946011	-1.83174694257775
H	-5.21020064047750	2.13207084331201	1.51394633540481
H	-3.78443873298410	2.39115610261641	0.51662795126759
H	-2.26007577225996	0.30610018582841	0.00283589786374
H	-2.74656472114544	-1.24436117154557	0.71779705033811
H	-3.52398317037030	-1.23328546308987	-1.49530410178892
F	-4.83440290275032	-0.38362722740388	1.93521291970400
H	-3.20550912122784	0.75082285198409	2.14082631957263
H	-5.61067480736629	2.31791895456488	-0.99338594610201
C	-6.68325327110919	0.63823764088672	-0.22364262346393
H	-6.49508476114230	-0.36175111145138	0.16132096370753
H	-7.28313015534541	0.55215966055734	-1.13088868856172
H	-7.26882651015035	1.18298499082009	0.51845305018146

4C_ae, Water

C	-5.37526801393418	1.35796248705453	-0.51256965765440
C	-4.49561615005996	0.56298056704050	-1.47955388802487
C	-4.57759688978656	1.66523441146467	0.75948825117116
C	-3.88266211189371	0.45051972659779	1.34304717856632
C	-3.12225760922529	-0.32023435241183	0.28388347206679
N	-3.98824934079272	-0.64163086101202	-0.83636694688862
H	-5.07681386634512	0.27285323152791	-2.35606762897500
H	-3.67374343262967	1.21149556344633	-1.82148987291106
H	-5.21335507683486	2.12370403381843	1.51944122414883
H	-3.79008824680331	2.38617274287658	0.52124324236638
H	-2.26956674975503	0.31127305084470	-0.01249661076970
H	-2.72451963631066	-1.23775954281995	0.71702115120179
H	-3.48138875170328	-1.20435981946838	-1.50669164683018
F	-4.82891208511974	-0.39816797470913	1.94109088186161
H	-3.20076054473548	0.74892948174342	2.14014733830053
H	-5.60335539687741	2.31217604835397	-0.99418217573374
C	-6.69566261965750	0.65036634933280	-0.22463440182946
H	-6.52403329635746	-0.34508525989965	0.18089059007843
H	-7.28506957294195	0.55405786027152	-1.13801191505521
H	-7.28360060823611	1.21599225594780	0.50055141491039

4C_aa, Gas Phase

C	-5.38158216734614	1.36607674568950	-0.50397212349760
C	-4.52142324774851	0.53423641637144	-1.46495244877702
C	-4.57468175661505	1.67374012119431	0.76434770596319
C	-3.89392050470825	0.45167865026182	1.35563362762541
C	-3.13450331032911	-0.32665118986578	0.29437987050121
N	-3.94009708365871	-0.65470861453831	-0.86383534749039
H	-5.11105729322760	0.23633854209275	-2.33375185682415
H	-3.69367360028573	1.14806688327307	-1.83602582777969
H	-5.20599179990917	2.13998225987802	1.52309609206929
H	-3.78335565175861	2.38965318315824	0.52047120070031
H	-2.28776006280174	0.28438371890278	-0.03681342458632
H	-2.72855850654120	-1.23995768998214	0.72926745874814
H	-4.66121834286271	-1.31658542139477	-0.60597839532949
F	-4.85261667303443	-0.39742484973555	1.91096662943279
H	-3.22631420760605	0.74149171534727	2.17044988395085
H	-5.60317291991922	2.31874178119650	-0.99368453344824
C	-6.70796972862081	0.67160313256239	-0.21173589597046
H	-6.55618012210426	-0.32306576029336	0.20608632646144
H	-7.29442867086753	0.57404820074238	-1.12661377035058
H	-7.29653435005516	1.23754217513946	0.51091482860133

4C_aa, Water

C	-5.38195075271078	1.36364874999332	-0.50404050560851
C	-4.52010276555183	0.53891320333171	-1.46702021590341
C	-4.57621081414323	1.67261700398360	0.76392931943494
C	-3.88387510936343	0.45902537756168	1.35234456351681
C	-3.13649046201660	-0.33116597776712	0.29477770398150
N	-3.94765053601596	-0.66073945540521	-0.86558265888451
H	-5.10838506932926	0.24867142093483	-2.33933881149462

H	-3.68782343547104	1.15349980383138	-1.82469889967720
H	-5.20749207222729	2.13675119396504	1.52424818353643
H	-3.78755758994745	2.39053278953823	0.51968077551199
H	-2.28836634827096	0.27640170230958	-0.03632080943790
H	-2.73581389367289	-1.24441568550435	0.73494960509280
H	-4.68669965499323	-1.29810554979495	-0.59117986381051
F	-4.84232719869773	-0.39379030447644	1.93343367992155
H	-3.20968320870616	0.75112961418880	2.15856066461324
H	-5.60472198939828	2.31615662492654	-0.99225229536931
C	-6.70756925795522	0.66473327951414	-0.21712961909360
H	-6.55428730576812	-0.33683187130634	0.18519483187527
H	-7.29385434703920	0.57687790171111	-1.13332996056734
H	-7.29417818872132	1.22528017846446	0.51202431236240

4C_ee, Gas Phase

C	-5.44219363267079	1.31695295134887	-0.49594593460961
C	-4.56353341802956	0.56680217301526	-1.48237103490063
C	-4.67789555782341	1.64237667068300	0.77029800161210
C	-4.04980957137766	0.38583356189708	1.37583387005117
C	-3.23384736436580	-0.33963423636835	0.30978740036116
N	-4.06666752928050	-0.63556431672694	-0.84208227760230
H	-4.85338655497859	-0.29109941770042	1.68223957207935
C	-3.19599798107136	0.72510372634484	2.58881768603751
F	-5.87449357480048	2.49345553192271	-1.09098587972096
H	-6.32853874706403	0.71972178225675	-0.26861628854899
H	-5.14967652581459	0.29769192209882	-2.36056041712067
H	-3.76069557255661	1.25120859177267	-1.79952080413307
H	-5.33641690670471	2.13185133798882	1.49045738995319
H	-3.88571741376156	2.35387003029052	0.51244936460048
H	-2.36821222881299	0.29325550447337	0.04763250244970
H	-2.84751702256051	-1.27600220152991	0.71548157528070
H	-3.58066287894483	-1.22251523103627	-1.50424002426472
H	-2.37484691645050	1.38839524340573	2.30831118190115
H	-2.76549265992225	-0.17299428635640	3.03415294623492
H	-3.78554794300927	1.23080066221986	3.35423117033953

4C_ee, Water

C	-5.44163078413628	1.30932942267686	-0.49036076144939
C	-4.56572701145695	0.56419829590082	-1.47999263542496
C	-4.67888293185201	1.64408267991723	0.77127091820131
C	-4.05305966111407	0.38565315511260	1.37683410591034
C	-3.23942149534585	-0.34308771483272	0.31209972439150
N	-4.07158631064558	-0.64564145974351	-0.84439529590568
H	-4.85932595282332	-0.28494610781479	1.69116722306036
C	-3.19486700940340	0.72621220800468	2.58658612443214
F	-5.87882186518589	2.49504434544405	-1.09480886190376
H	-6.33431398076524	0.72316344404627	-0.26349401707550
H	-5.14776662441011	0.30004788772603	-2.36251644850351
H	-3.75462225769380	1.24183087539307	-1.78770379503102
H	-5.33678417982152	2.13178955832645	1.49322468526695
H	-3.88198636716088	2.34959615030857	0.51101252723974
H	-2.37793191056149	0.28916071341072	0.04126408464102
H	-2.85008340297371	-1.27741259710818	0.71892222519858
H	-3.55212619272761	-1.19682965758368	-1.51473386631334
H	-2.37065677774578	1.38285140506375	2.29849328537936
H	-2.76979866439154	-0.17429615422772	3.03263199850360
H	-3.78175661978496	1.23876354997952	3.34986877938227

4C_ea, Gas Phase

C	-5.45423337908562	1.33212647280812	-0.48332263735391
C	-4.60157152382584	0.54248469961155	-1.46636915461478
C	-4.67501434274791	1.65639578495600	0.77523132515335
C	-4.06697934588045	0.39675722501162	1.39546633314109
C	-3.27415880955682	-0.35926451014379	0.32756951831557
N	-4.03227674805777	-0.65385433081032	-0.87534845306916
H	-4.88270294430006	-0.25962317823475	1.72180304542750
C	-3.19728455822182	0.73054178817324	2.59835107604239
F	-5.87215635048320	2.51002768269037	-1.08687540386991
H	-6.35529828815468	0.76052197503033	-0.23698948183155
H	-5.18604873593766	0.27683459299272	-2.34687079867685
H	-3.78236572373153	1.18813949177301	-1.79886840702436
H	-5.31973936916920	2.16985359947253	1.49123009904388
H	-3.86995358942505	2.34822465657891	0.50420431001196
H	-2.40952544666024	0.24958960834980	0.03619359129452
H	-2.87992393132102	-1.29185635780054	0.73526567441361
H	-4.75240480802016	-1.33901327544586	-0.68319336635576
H	-2.38193494217884	1.39633547428983	2.30662224516219
H	-2.75586273135010	-0.16983938757327	3.02847842210965
H	-3.77344443189202	1.22990798827048	3.37781206268057

4C_ea, Water

C	-5.45029777159853	1.32143552969700	-0.47933648411130
C	-4.59792760539990	0.54370502949796	-1.46867963768077
C	-4.67267282543810	1.65766039670803	0.77333734215351
C	-4.06469147468607	0.39623123903604	1.39085821591939
C	-3.26725111991502	-0.35588317957760	0.32566615673702
N	-4.03227417330086	-0.65896163035232	-0.87756469156188
H	-4.88055328122895	-0.26152626785008	1.71120946694249
C	-3.19854000462800	0.72843767553019	2.59672745348029
F	-5.87865838214995	2.50784605555307	-1.09220097028374
H	-6.35085510401338	0.75198788788620	-0.23637037160480
H	-5.18701404540104	0.27888935661275	-2.34655457702219
H	-3.77662608045085	1.18997350754400	-1.79508073756588
H	-5.31954538034614	2.16431407822416	1.49236610781771
H	-3.86584399143312	2.34760364694549	0.50179828016875
H	-2.40624884306480	0.25633994985319	0.03157588015679
H	-2.87545870704176	-1.28727428317690	0.73779805441114
H	-4.77494641070836	-1.31196978458145	-0.65170429854441
H	-2.38148127575088	1.39380026518289	2.30753315081495
H	-2.76179533656723	-0.17499129228457	3.02573379504070
H	-3.78019818687704	1.22667181955195	3.37327786473222

5A_a, Gas Phase

C	-5.35156944613965	1.52792395526125	0.55258893582623
C	-5.05550002473412	0.88148605541547	-0.79604015885387
C	-4.11083534024932	2.21964842567233	1.10890262249906
C	-2.91156624208247	1.29040726482192	1.09203763761524
H	-4.28119420500294	2.57472986832429	2.12513598297369
H	-3.86211896220589	3.09173084469829	0.49686561948947
C	-2.70011841888429	0.68148666982358	-0.29429644009005
F	-3.11523790152011	0.26312462842068	1.99825749816711
H	-2.00400140410945	1.81767513054891	1.39671385866379
N	-3.92671067693156	-0.03339395642719	-0.67719096241124
C	-1.38184582877786	-0.06638754864989	-0.39359105067355
H	-2.64492332863389	1.52610520478295	-0.98928322314150
C	-4.05434456943486	-1.37156931597331	-0.53414250025538
O	-3.18317974885289	-2.13846230231790	-0.22314026395910
C	-5.45357512021448	-1.99100412413028	-0.83574095086604
F	-5.87564848103951	-1.70484999384462	-2.07430331938824
F	-6.38314254938169	-1.55099432635340	0.02467852970914
F	-5.39175537904637	-3.30177388186613	-0.72388486045993
H	-6.16902765518337	2.24113044125891	0.44576180725426
H	-5.67949971348004	0.75036966279242	1.24439478735514
H	-5.92676012689435	0.36929873282520	-1.18410682912599
H	-4.77912251205574	1.64308035018653	-1.53040269871275
H	-1.29758400355620	-0.60731509367328	-1.33303091945476
H	-1.25151651834788	-0.77202143502022	0.42002286306995
H	-0.58389184324106	0.67754480342349	-0.35227596523066

5A_a, Chloroform

C	-5.35102640485844	1.53042246383733	0.55625248018235
C	-5.05848239827673	0.87981372590034	-0.79051231919649
C	-4.10838481818539	2.21913848716943	1.11222151189152
C	-2.90475172576868	1.29937721311163	1.08999295466850
H	-4.27663929813574	2.57418747201324	2.12886739459639
H	-3.86067129293037	3.09046974193237	0.49993599212138
C	-2.70052240291694	0.67792016666619	-0.28909447725001
F	-3.09292967420362	0.26843366549863	2.01129596499144
H	-1.99855142271027	1.83104373436956	1.38515812233528
N	-3.93258037670308	-0.04335323228132	-0.65597565335580
C	-1.38163559067334	-0.06551234797895	-0.39742524538542
H	-2.65605550199367	1.51506336906338	-0.99099976644892
C	-4.06399901221463	-1.36933029629185	-0.51953705114261
O	-3.20085610554313	-2.14908038728791	-0.18738194024851
C	-5.45626369635103	-1.99009188034649	-0.85031846486100
F	-5.85934258045356	-1.70473873490306	-2.09298553218686

F	-6.40253997216699	-1.56899812277429	-0.00204688273825
F	-5.38773424888013	-3.30668144991720	-0.74610764549079
H	-6.16162026642067	2.24954504895461	0.44151062544340
H	-5.68960035635980	0.75877610737974	1.25033605378417
H	-5.93250146972674	0.37268389741337	-1.17862286919193
H	-4.76926851943901	1.63374461171339	-1.52521861964145
H	-1.30518105051226	-0.60878862992198	-1.33660665788305
H	-1.23336960850147	-0.76128289777974	0.42193235341001
H	-0.59016220607429	0.68520827445958	-0.37074032840333

5A_e, Gas Phase

C	-5.21965986631879	1.78490561473569	0.66499931661582
C	-5.14645535976138	0.82260905350617	-0.52468911108741
C	-3.90241564335368	2.46790482009921	0.95483238006518
C	-2.83368463585809	1.40543959173479	1.17876033606894
H	-4.01092196857453	3.11060509271175	1.82816114967513
H	-3.63327279293227	3.10517739968930	0.10890890237792
C	-2.73142731617377	0.48112188325925	-0.02348378541131
H	-3.07857329919610	0.80805245990320	2.06068575772185
H	-1.86283683754271	1.86706254416773	1.35579252120065
N	-4.02826905604260	-0.10964037427690	-0.32183334751586
H	-2.01013919548479	-0.30204700564939	0.17501531021140
H	-2.37726037852872	1.03923082058196	-0.89604229783036
C	-5.04646725009895	1.54121725981085	-1.86393983421315
H	-6.05241503502795	0.22138179124948	-0.52324889312081
C	-4.21186825711848	-1.37840648662533	-0.75159702221847
O	-5.24429900812758	-1.80778659756527	-1.19808578821640
C	-3.01623621609837	-2.36719627382066	-0.64822146985301
F	-2.00524506609483	-1.98944279527820	-1.44136895935530
F	-3.39791281241402	-3.57053493939685	-1.02360126408385
F	-2.54587327511008	-2.45663032232670	0.60235640137537
F	-6.19187441821884	2.73211210996373	0.39594199602209
H	-5.55016770886763	1.23015517834963	1.54717977098812
H	-4.11562327007214	2.09897118547991	-1.96452039392687
H	-5.87186037559299	2.24279872917394	-1.96777699018252
H	-5.10565095739070	0.81260926052271	-2.67101468530714

5A_e, Chloroform

C	-5.22480834696386	1.71904403702067	0.65276236361777
C	-5.13661376594976	0.84088880813108	-0.60048651235461
C	-3.92264760980063	2.40914978123673	0.98640675854476
C	-2.82425209915139	1.36068816168076	1.12766624509735
H	-4.04009259295805	2.97760440976342	1.90872173768552
H	-3.66991191478052	3.11398394990186	0.19069107146144
C	-2.72180669728695	0.51630166999921	-0.13230991224349
H	-3.03924913729182	0.70522562676050	1.97534579258683
H	-1.86312337924088	1.83796365781687	1.31367597091528
N	-4.01252242030303	-0.09216545427299	-0.43476543187945
H	-1.96553039999294	-0.24891893187487	-0.01353356248379
H	-2.42403551400445	1.13812519344244	-0.97963816840555
C	-5.01659812833364	1.64806365519640	-1.88597266923516
H	-6.04031907107843	0.23990558195984	-0.64916016430545
C	-4.22250132590048	-1.38986440674903	-0.68962863677475
O	-5.28060694245658	-1.87335992674874	-1.02980423157055
C	-3.02735128552890	-2.37316526394067	-0.53172651130374
F	-2.04404155212124	-2.10166877347613	-1.39613585741903
F	-3.43609492174710	-3.60870151346255	-0.76595913376539
F	-2.51373925457265	-2.34436380382737	0.70271838696469
F	-6.22090075931877	2.66825617926238	0.44292048737061
H	-5.54818588772883	1.10288330060938	1.49428911055192
H	-4.11547321984284	2.25931967573383	-1.91149184898704
H	-5.87540516882679	2.31026326197631	-1.97980783043538
H	-5.00459860481946	0.97421112386067	-2.74156745363279

5B_a, Gas Phase

C	-5.30245000103960	1.34087243303636	-0.40927694851086
C	-4.45407088251991	0.62424958808334	-1.45330532478753
C	-4.49204444530705	1.71718385795238	0.81300087349567
C	-3.84033489028377	0.47943886782439	1.42370927103133
C	-3.06765628134134	-0.32676911079303	0.39563844032510
N	-3.95131166868052	-0.62701432807634	-0.78125654713651
C	-5.20931230869411	0.27504429438845	-2.71279485055262
H	-3.57021307158012	1.22364311925841	-1.67504092156069
H	-5.13888622160076	2.20416541719527	1.54098394313471
H	-3.73758055274114	2.44871022447942	0.51256078224014
H	-2.21653967215877	0.22764269105150	0.00116731592347
H	-2.71736437713869	-1.27417125385490	0.79739835644651
H	-3.46127109319145	-1.20922906489665	-1.46086023350431
H	-4.77464090170823	-1.15270674657080	-0.46864402946445
H	-5.78601664674922	2.20569065292723	-0.86312408393400
F	-6.29333768460192	0.43947762736975	-0.03774018389446
H	-4.60542196091105	-0.14966312397720	1.88166569872576
H	-3.15172053200235	0.76471181435240	2.21753281463088
H	-6.11199363142443	-0.29103171534061	-2.48548541124911
H	-4.59010784885134	-0.29131910400822	-3.40829067116819
H	-5.50587532747421	1.19645405359886	-3.21175829019086

5B_a, Water

C	-5.28837993307852	1.37051550935102	-0.41759079132961
C	-4.46151666377202	0.61460117834246	-1.44361971748597
C	-4.48817584845815	1.71270157586821	0.81909802490763
C	-3.86075121438477	0.46077591126981	1.42201808437982
C	-3.06691336821608	-0.31589889174883	0.39117513567919
N	-3.93149046423157	-0.62048672448183	-0.78718688758797
C	-5.23120945647766	0.25213445891180	-2.69053365014418
H	-3.58921222221040	1.21938753769330	-1.69158261905534
H	-5.13387393317373	2.21118834058659	1.54094478129595
H	-3.71286783332001	2.42418085181408	0.52526476071378
H	-2.22673716971612	0.26234786038561	0.01028267216632
H	-2.70486477668882	-1.26282084456154	0.77953682114347
H	-3.40502425762561	-1.16537182887187	-1.47032654356846
H	-4.71688363455025	-1.20871463013466	-0.49664707226530
H	-5.70815597333902	2.25954335872513	-0.88613693497642
F	-6.35789360829031	0.54364366012252	-0.05398793298618
H	-4.63755888521270	-0.18181761923143	1.83962537455635
H	-3.19052274877224	0.72860443235743	2.23714965544687
H	-6.10812408743012	-0.34638561585730	-2.44759440928617
H	-4.60003802564991	-0.30122642575558	-3.38433036138432
H	-5.55795589540196	1.16847790521510	-3.17947839021949

5B_e, Gas Phase

C	-5.37879805157138	1.36485345394075	-0.43854993249419
C	-4.54720329878734	0.60214770623883	-1.45044104432100
C	-4.57693077821790	1.70560554533464	0.81608018729226
C	-3.98464703554929	0.44716214315472	1.41844848511581
C	-3.13242462717048	-0.34594405360422	0.41741680856248
N	-3.98611600853128	-0.63189271534800	-0.80017433986488
H	-5.21821197032631	2.18231172570311	1.55593373468964
H	-3.77773142633580	2.41154095697185	0.58475736598948
C	-1.85512083897703	0.36146877033865	0.02040321146867
H	-2.89768743603491	-1.32035178167735	0.84470274941610
H	-3.42511966305642	-1.15484241799959	-1.47485758478685
H	-4.76344105975246	-1.24028719887618	-0.53267445431038
H	-4.78759672135100	-0.19446714133531	1.79859521818341
F	-3.16647240363778	0.75232954441925	2.47181561081248
H	-5.74416667076776	2.27426244873292	-0.91354928713091
H	-6.26388877201636	0.77724916268397	-0.17750003090912
H	-5.13087344187332	0.27813928172551	-2.30891503132655
H	-3.69984082858934	1.18471064677344	-1.80474827955857
H	-2.02666140392737	1.36812588124010	-0.35462501440697
H	-1.30091232920821	-0.20621305948433	-0.72748991592448
H	-1.22915523431827	0.44696110106722	0.90590154350358

5B_e, Water

C	-5.37147905370215	1.36755087905366	-0.43759537663377
C	-4.53972417952531	0.59654134348764	-1.44175803152152
C	-4.57137588676922	1.70921144496651	0.81784120820733
C	-3.99504734107002	0.44232577284746	1.40871206579326
C	-3.13632277858941	-0.34142947230729	0.41418677885771
N	-3.98144758858713	-0.62633017911071	-0.79154243756877
H	-5.21419667306398	2.18671857406680	1.55626768228892
H	-3.76518343417289	2.40478974139507	0.57904672860176
C	-1.86043849483963	0.37129422587161	0.01839175006176
H	-2.89945417159596	-1.31414982043683	0.84004914636528
H	-3.42504678594925	-1.15290860801142	-1.46611971702738
H	-4.75195494484277	-1.24415292173849	-0.52876302980534

H	-4.79936902698961	-0.19999090159247	1.77479280543275
F	-3.18705515980383	0.74232168024855	2.49648336363484
H	-5.72916095685550	2.27745377950060	-0.91617458368881
H	-6.25101133993127	0.77645818946962	-0.17133728957788
H	-5.12831510481325	0.26206830477890	-2.29079840823593
H	-3.69790667044857	1.17942081940436	-1.80688814475603
H	-2.03727561413765	1.37846680052728	-0.35312674427395
H	-1.33090561897436	-0.19669979596137	-0.74572041136943
H	-1.22032917533825	0.44391014354049	0.89458264521521

5C_ae, Gas Phase

C	-5.27191402229245	1.39076427023461	-0.43100440782299
C	-4.43388339925708	0.60251799138947	-1.43367192687306
C	-4.48319212089023	1.70245026565255	0.82657014311330
C	-3.87725674095202	0.43140769523292	1.41088985121235
C	-3.06110329450710	-0.29717564153375	0.35164964698283
N	-3.90323848315624	-0.58618329613386	-0.79451905398950
C	-5.24368477535786	0.22458911430065	-2.65859642857471
H	-3.62467700688325	1.29327852177069	-1.73517193794825
H	-5.13042950366535	2.20516918611702	1.54599124881848
H	-3.68722250161680	2.40357863643281	0.55726169884074
H	-2.19112258219842	0.32576372415002	0.08200138124631
H	-2.68167627737712	-1.23775001038476	0.75112943226866
H	-3.42353705688933	-1.16514315830936	-1.46987638139560
H	-5.62182341343715	2.31048109491165	-0.90752090827382
F	-6.40311839704665	0.66432006929438	-0.08663869516377
H	-4.67733971223328	-0.22571430143228	1.75479970724826
H	-3.24800006292407	0.67171711201563	2.26878777219676
H	-6.09576437062751	-0.38544437166925	-2.36366549559576
H	-4.63431399441541	-0.34472721707353	-3.36182380826713
H	-5.60690228427269	1.11606031503440	-3.17053183802308

5C_ae, Water

C	-5.26839011122947	1.39119358622293	-0.43104207356975
C	-4.44113117276307	0.59424095558247	-1.43010232405927
C	-4.48575553206485	1.70123537394824	0.82742098301644
C	-3.87446537464621	0.43372850923709	1.41461340255196
C	-3.06789909542224	-0.30173908109034	0.35390744011298
N	-3.91801087580270	-0.60470399216203	-0.79069743757974
C	-5.24400397973158	0.22893423962717	-2.66357530861993
H	-3.62313613735496	1.27262670844407	-1.72563267582579
H	-5.13159260651335	2.20831698568158	1.54534036610042
H	-3.69114831327469	2.40125768064672	0.55366376407693
H	-2.20779293534620	0.32266860897765	0.06565544793256
H	-2.67789949920450	-1.23722159873273	0.75486374819855
H	-3.39922859501090	-1.14320791892761	-1.47383551915822
H	-5.62681288424886	2.30553813582885	-0.90677010649513
F	-6.41162989281178	0.65601119960172	-0.08036697278088
H	-4.66725801104926	-0.22157845834616	1.78159970316917
H	-3.23442315554006	0.68281563122096	2.26166489281732
H	-6.11031332800779	-0.37163546468351	-2.38721551737684
H	-4.63117348826591	-0.34668271462041	-3.35820704784840
H	-5.58813501171164	1.12816161354336	-3.17522476466236

5C_aa, Gas Phase

C	-5.29199889287462	1.38222403869983	-0.41680245348370
C	-4.45959108651634	0.58307048296515	-1.41709603237274
C	-4.48426046752409	1.72095613795320	0.82214261799402
C	-3.86687331718457	0.46394076535621	1.42965966732401
C	-3.11278844708032	-0.33108465324904	0.36531127613780
N	-3.91028447708604	-0.62180651085170	-0.81657174713157
C	-5.23951520275565	0.24446056229869	-2.67300512560704
H	-3.60929254998650	1.22001386814188	-1.69007179029631
H	-5.11710483912111	2.23958986396120	1.54294792823366
H	-3.69482894500722	2.41713060761538	0.52224801816024
H	-2.23712091082266	0.23903172683439	0.03619389195039
H	-2.74090941369922	-1.27018746057600	0.77566165676725
H	-4.67245026114317	-1.24788198314205	-0.57852007681072
H	-5.69684498594508	2.28345171566444	-0.88363857613625
F	-6.38502559890737	0.60050421635795	-0.03983455003272
H	-4.66004881701759	-0.16051756754031	1.84684320017519
H	-3.19761866211182	0.72946581700227	2.24998929653514
H	-6.10008664437311	-0.37992806275247	-2.43154615886604
H	-4.60153519179195	-0.29754853585839	-3.36899511407646
H	-5.60279128905158	1.14989497111936	-3.16013592846414

5C_aa, Water

C	-5.28063808414466	1.39140982779517	-0.42401818535281
C	-4.45916814686869	0.57856319260721	-1.41878932120211
C	-4.48443413587173	1.71653809984316	0.82298629431433
C	-3.86882350626748	0.45825443056355	1.42909415448389
C	-3.10763662913866	-0.33000573708554	0.36732140644511
N	-3.91297383211961	-0.63086155609139	-0.81302152896257
C	-5.24326135798392	0.24039813091679	-2.67256868677711
H	-3.60911256335498	1.21312589219759	-1.69392294452612
H	-5.12154309888698	2.23444962325179	1.54090435349168
H	-3.69325612467576	2.41275424952428	0.52979740626490
H	-2.24125078669557	0.25083400133561	0.03416052135539
H	-2.72922413400633	-1.26531793189588	0.78016660915689
H	-4.68335056362328	-1.23867690902459	-0.54941624731540
H	-5.66890902884592	2.29599103550602	-0.89455528221184
F	-6.40181558607246	0.62465372231128	-0.05461754125774
H	-4.65768495202241	-0.17469592154407	1.84296580076107
H	-3.20152102017250	0.72438144167471	2.25045902909053
H	-6.08129386109232	-0.41650878840960	-2.43596529669415
H	-4.59908563627552	-0.26645343747521	-3.39026460057847
H	-5.63598695188122	1.14594663399912	-3.13593594048547

5C_ee, Gas Phase

C	-5.37942591438599	1.36889780917727	-0.44176052417783
C	-4.52398126980586	0.58957123999242	-1.43103406757606
C	-4.57609808845976	1.70125565726565	0.81425156664057
C	-3.97910242911899	0.43377841059431	1.39019343599674
C	-3.14128511833474	-0.33509864745826	0.36787623856295
N	-4.00111936798237	-0.60181240666197	-0.78045307028470
H	-5.20268448314382	2.18039125425628	1.56738844861144
H	-3.76910208761307	2.39738665959051	0.57325465302721
C	-1.82824487117724	0.37231395604631	0.04034412956215
H	-2.90201139291078	-1.30174914201104	0.81715285584407
H	-3.52559600884837	-1.19935479517369	-1.44251768924695
H	-4.77742672620928	-0.22154921740927	1.74667482803629
F	-3.18606845594809	0.74776444242101	2.48552098578721
H	-5.75094023387065	2.28351540268670	-0.90530777077288
H	-6.24557967175470	0.75909568939858	-0.17366684408430
H	-5.12952145793765	0.27301456969064	-2.28062018146024
H	-3.73688474627922	1.25015151063851	-1.82310865849204
H	-1.98744631117619	1.35098097141498	-0.41088685025365
H	-1.25248713545854	-0.23268017215946	-0.66136230772728
H	-1.23554422958471	0.51058680770053	0.94316082200732

5C_ee, Water

C	-5.37715442822765	1.36860994849063	-0.44149023992526
C	-4.52363829277466	0.58412224044289	-1.42739445386748
C	-4.57506245564919	1.70319668298761	0.81543225049815
C	-3.98749233284174	0.43259436251758	1.38759935452520
C	-3.14356405619872	-0.33688839567838	0.37357591112717
N	-4.00256409720005	-0.61446491701741	-0.77740252317653
H	-5.20538981267478	2.18375666979071	1.56468607948219
H	-3.76633336847980	2.39544252143208	0.56910281860651
C	-1.83583072370508	0.37429533685362	0.03430048624648
H	-2.89674937173146	-1.30020654081265	0.82564213015002
H	-3.49220992494860	-1.17949504678985	-1.44495252482675
H	-4.78605661787454	-0.21749029442963	1.75089936724159
F	-3.19112204561220	0.74435382179379	2.49649178334658
H	-5.74092955397503	2.28414667509889	-0.90854141026806
H	-6.24934183171206	0.76790293246082	-0.17027449805896
H	-5.12620344194721	0.27097093305846	-2.28007784079616
H	-3.72949764866809	1.23665533182440	-1.81411073361723
H	-1.99994125690930	1.35933473708790	-0.40101667839854
H	-1.27733841007266	-0.22612618226161	-0.68489079750101
H	-1.22413032879717	0.49574918315016	0.92752151921208

5C_ea, Gas Phase

C	-5.38878697421703	1.38424494015091	-0.42960621815286
C	-4.54905379996220	0.56642038600689	-1.40850048540025
C	-4.57469076841789	1.71478953698938	0.82082290441949
C	-3.98628958447234	0.44760783861697	1.40907845525799
C	-3.16410006109727	-0.34271490406799	0.38676841145193
N	-3.95835523088080	-0.62030805405434	-0.80779344841620
H	-5.19043684772246	2.20824177553169	1.57340957449696
H	-3.76096587083899	2.39905720579667	0.56949305149249
C	-1.85761797806821	0.35584979852346	0.03377683199966
H	-2.91245570501641	-1.30687097397401	0.83333821508601
H	-4.68183835974062	-1.29655711154861	-0.59742028989489
H	-4.79412833977571	-0.19403396354255	1.77701653067708
F	-3.19626398429087	0.76538463621331	2.50597742690022
H	-5.74615988076084	2.30035714136422	-0.90353008565833
H	-6.27255768646245	0.80172801325062	-0.15088170043942
H	-5.14603108361518	0.25369402659762	-2.26555299431188
H	-3.74219795113776	1.18752077282383	-1.80572892374785
H	-2.01773930924804	1.33998503753428	-0.40519610799710
H	-1.30800752602110	-0.24847975994978	-0.68608975124239
H	-1.25016305825384	0.48764365773742	0.92725860347933

5C_ea, Water

C	-5.38431939360078	1.38219469729783	-0.43035413348782
C	-4.54569716788534	0.56757408474092	-1.41060106131321
C	-4.57118572251492	1.71365067706049	0.82089324043763
C	-3.99306104909419	0.44128596563895	1.40037834755582
C	-3.16045140008838	-0.34417103323869	0.38585640083501
N	-3.95947454697882	-0.62752603812583	-0.80843695198422
H	-5.19278340954683	2.20363940632393	1.57122185101900
H	-3.75703131018718	2.39701362892555	0.56742483723428
C	-1.85818216346995	0.36109355024955	0.03040488182185
H	-2.90930117549152	-1.30688545526677	0.83532879745479
H	-4.70360829267601	-1.27279553712647	-0.56599597854777
H	-4.79881192258949	-0.20078552787011	1.76586876797291
F	-3.20067754582278	0.75278853999930	2.51424778833870
H	-5.73905953588885	2.29873559279117	-0.90443434060576
H	-6.26582743349618	0.79748305590258	-0.15107523662110
H	-5.14829158705804	0.25597633245979	-2.26392605780702
H	-3.73780472072005	1.18819115237703	-1.80563371227918
H	-2.02443166423443	1.34147396860786	-0.41472180380885
H	-1.29869870847432	-0.24412091084909	-0.68241672589120
H	-1.24914125018195	0.49874385010201	0.92261108967612

6A_a, Gas Phase

C	-5.58116969620360	1.05703266168691	0.11361930661183
C	-4.84870271723792	0.68413667980728	-1.17069919150009
C	-4.61504077111808	1.48403400577474	1.20175642704247
C	-3.52419661247637	0.43692273549356	1.40232830739513
C	-2.85726283127750	0.08717560017572	0.07358432243895
N	-3.88080957597255	-0.35964953773765	-0.89161949902441
C	-4.18484250260682	-1.66968237774537	-1.05123950347371
O	-3.68959805930751	-2.58459615328182	-0.44959023747009
C	-5.19703770986528	-2.03023240495704	-2.18428095281321
F	-4.92711869300325	-1.36684162451054	-3.31707223427671
F	-6.45875100949551	-1.75416814763754	-1.84353312894964
F	-5.12177028834001	-3.32142155006953	-2.44464588229054
H	-2.48436077335003	1.01944752779629	-0.36612476592523
C	-1.65882831743285	-0.82959517313756	0.25365986437504
H	-6.31627976049219	1.83666715866187	-0.09318229308329
F	-6.27360958157742	-0.06401972277021	0.54461081017138
H	-5.16567756617331	1.66588835531328	2.12454061486151
H	-4.16503569704806	2.43420824502454	0.89801555376264
H	-3.94412760076330	-0.47479802984822	1.83245839705600
H	-2.76706861959337	0.80772109199500	2.09562955886100
H	-5.56142241158214	0.36831726780494	-1.92348096394208
H	-4.32270159769443	1.56180394221118	-1.55340636778592
H	-1.88680799530746	-1.67480923119393	0.89601431379847
H	-0.86054685574681	-0.24526091909342	0.71383550277529
H	-1.29623275633421	-1.21318039976248	-0.69827795861479

6A_a, Chloroform

C	-5.57766036979628	1.06630972241095	0.11108946195768
C	-4.84968314838212	0.67829923371440	-1.16861519639491
C	-4.61501440314451	1.48567709496407	1.20201220886475
C	-3.52375135790583	0.44014930178378	1.40751418828625
C	-2.85922953506792	0.08022292892812	0.08116585373773
N	-3.89283647228796	-0.37690809976505	-0.87468539968642
C	-4.17530051551430	-1.67387211711300	-1.06836165222088
O	-3.66234683799733	-2.61031599583636	-0.50009276393496
C	-5.20297089939245	-2.02469754410633	-2.19042671551038
F	-4.95782606022672	-1.35686426399661	-3.32328322128534
F	-6.46221620829130	-1.76695640953809	-1.82700903223551
F	-5.12495295824394	-3.31775336223386	-2.46430704935949
H	-2.49390114898366	1.00797908841238	-0.37033013677502
C	-1.65691235579446	-0.82897474624604	0.26412086053389
H	-6.30586386739165	1.84814115747764	-0.10255744032296
F	-6.28866145791973	-0.05482853367917	0.54451464445622
H	-5.16765140790269	1.66996588915622	2.12315623526528
H	-4.16388389362411	2.43407091820469	0.89707769667679
H	-3.93717833732851	-0.46801344112064	1.85237255935949
H	-2.76312551206106	0.82141456869476	2.09067232446079
H	-5.56550926548912	0.36971591543353	-1.92070190980404
H	-4.31134115942141	1.54618510384682	-1.55204186839843
H	-1.87515241447506	-1.66718485584847	0.91966694435658
H	-0.86486537833785	-0.23151131260559	0.71771753354732
H	-1.29116503502000	-1.21515024093815	-0.68576812557443

6A_e, Gas Phase

C	-5.44674043076812	1.23782193434707	0.28646800158793
C	-4.99781398869235	0.60180021160314	-1.03017045433333
C	-4.27334780118878	1.66502302029042	1.16401718158554
C	-3.34932703409851	0.48884101719549	1.38731231803956
C	-2.88761645440569	-0.10162785231361	0.06345830631540
N	-4.03437857948094	-0.47177395604968	-0.74240020108559
C	-4.22622740121422	-1.66785801756657	-1.33774267224362
O	-5.11789426382564	-1.91557019461932	-2.10896911001352
C	-3.24798201164196	-2.82190714445579	-0.98575422250198
F	-3.20852434970376	-3.04547319393546	0.33444806608727
F	-2.00236933387380	-2.55251689997230	-1.39493439906010
F	-3.63702323925793	-3.93403209762871	-1.57417685017220
H	-5.85301908149740	0.11528298080375	-1.49156908786541
C	-4.43715808373047	1.61158087716786	-2.02568975646175
H	-2.29149299104788	0.64997270315925	-0.46220473351683
H	-2.24890843065245	-0.95620960547468	0.24955064025778
H	-6.08345145140216	2.09530024195046	0.06462430994121
H	-6.06222080323230	0.51573403968132	0.82790077799608
H	-4.62808573397015	2.04628373560647	2.12135691457729
H	-3.70022854089462	2.46351823538774	0.68627935703659
H	-3.84631071681829	-0.28663390787766	1.97772932722371
F	-2.22480053324549	0.89348381731771	2.08071362438308
H	-3.56526008434370	2.13969789466399	-1.64013278072661
H	-5.19958980332600	2.35613648755941	-2.25638483410831
H	-4.15838885768740	1.11313567315968	-2.95329972294220

6A_e, Chloroform

C	-5.44630074568741	1.23646398577490	0.28407529752736
C	-4.99952493959730	0.60607222293411	-1.03533588336736
C	-4.27304927152965	1.66364381358728	1.16184257423276
C	-3.35742108267758	0.48300548040463	1.38062862435540
C	-2.88824774421214	-0.09996459405276	0.05647674353877
N	-4.03522699047718	-0.47359210112803	-0.75208283668830
C	-4.22901825480661	-1.66809204514129	-1.32476474027089
O	-5.13098581962637	-1.93730242170994	-2.08847974522739
C	-3.24356662882626	-2.81822458626209	-0.97767662080466
F	-3.19148347153844	-3.04613143551515	0.33921299934251
F	-2.00343035325042	-2.55547253067261	-1.40210323167551
F	-3.63984144101706	-3.93518743428454	-1.56325581215922
H	-5.85875741961260	0.12768467856796	-1.49582092879363
C	-4.43119728302144	1.61810092353292	-2.02297466791014
H	-2.30281666438092	0.65672977626725	-0.47170094384748
H	-2.24300004508301	-0.94992953416987	0.24033805313664
H	-6.08102308189517	2.09473014810430	0.06158796481246
H	-6.06019493437447	0.51347953248796	0.82547742479247
H	-4.63257257948435	2.04016071955692	2.11919107244572

H	-3.69953380058668	2.46155457010843	0.68374082110130
H	-3.85331417042980	-0.29073169814715	1.97186514751044
F	-2.22399834656494	0.88170507093174	2.08001380811437
H	-3.56525562449468	2.14700016854847	-1.62571398194289
H	-5.19687503832450	2.35916069616879	-2.25362246457319
H	-4.14152426850103	1.12514659410775	-2.95048867364954

6B_a, Gas Phase

C	-5.42984278330012	1.23729709461387	-0.39047107285102
C	-4.60936816777374	0.48922240993774	-1.42891104863178
C	-4.63785244520150	1.56321550151633	0.87125339341951
C	-3.98925893628778	0.31218659065491	1.42720631843646
C	-3.11332568225452	-0.36905274771728	0.39234575263843
N	-3.98788343476695	-0.72272081307758	-0.76532371834165
H	-3.46796152889987	-1.26203846559551	-1.45829883692731
H	-4.74107059193526	-1.32530962829827	-0.41671088674228
C	-5.42671808380552	0.03002819724939	-2.61402229540783
H	-3.75915036677445	1.09482149154819	-1.75000181339660
H	-2.32875334817228	0.29508679353105	0.03392163880267
H	-2.67499821318138	-1.28352154468362	0.78350301266941
H	-6.30792425135360	0.63943265167057	-0.13319724576567
H	-5.79999052529969	2.15118088833127	-0.85405657145649
H	-5.29059026614192	1.99748081330151	1.62685560483631
H	-3.85562004822706	2.29703146635322	0.66127620333052
H	-3.41040304686221	0.51662830060430	2.32676618220262
F	-4.97893016610702	-0.60880592919518	1.74470037631639
H	-4.82216763901074	-0.49753473547509	-3.35178791127810
H	-5.86538056004365	0.89820454225094	-3.10268109520432
H	-6.24527991460073	-0.61638287752075	-2.29254598664930

6B_a, Water

C	-5.44911886840393	1.21462318226168	-0.38274985430187
C	-4.61318225227120	0.48635958733064	-1.42082313350980
C	-4.64492922010606	1.56366967545286	0.86322189185161
C	-3.97479113867314	0.33238271532328	1.43139406767604
C	-3.14744090768598	-0.39194781635836	0.39476482897985
N	-3.99893345578832	-0.72442184043128	-0.77756395456603
H	-3.44257461432395	-1.23120509103407	-1.46665076744122
H	-4.74053963884233	-1.36582666697738	-0.48439749176341
C	-5.41532598397418	0.04892082803943	-2.62381424770613
H	-3.76624001446684	1.10359286887655	-1.72395162988501
H	-2.33877213803273	0.24628599690679	0.04509088472660
H	-2.73973786181270	-1.31815613651656	0.78809129048503
H	-6.30589734693042	0.59000589043558	-0.11683491552867
H	-5.84331541495944	2.11882003066791	-0.84494915953266
H	-5.28276212932979	2.01193263756259	1.62392783864189
H	-3.86459292266972	2.28853124273686	0.62022239259147
H	-3.34685793781866	0.57311349032882	2.28714555212163
F	-4.96134580911472	-0.55940574841392	1.86813062793135
H	-4.79108713935075	-0.47144098764615	-3.34919194187097
H	-5.84336305082800	0.92782235149380	-3.10282480321288
H	-6.23166215461711	-0.60720621003906	-2.31841747568686

6B_e, Gas Phase

C	-5.53424450992260	1.18323789711790	-0.36093502076935
C	-4.69772052653869	0.42628067287230	-1.38895420750602
C	-4.72951027785184	1.53962943488949	0.87236585100132
C	-4.07012846924052	0.29167306785780	1.45797380414411
C	-3.19547052358912	-0.44035109796523	0.44924864645681
N	-4.06270525061734	-0.77207024675220	-0.75599178137005
H	-3.49901896572808	-1.26347959316140	-1.45168874021928
H	-4.79308305324137	-1.42375615828227	-0.46022149430425
C	-1.96518436510819	0.32846791684166	0.01077940817542
H	-2.89503957562696	-1.40886048469863	0.84806324889101
H	-6.40966383052140	0.58593312629042	-0.08200787554106
F	-5.97925545597350	2.31142798153733	-0.99246309867569
H	-5.38629883272057	1.99450530460969	1.61234342751899
H	-3.98655683841150	2.29145083704553	0.60149894507147
H	-3.44601694186210	0.55919599210535	2.31016003979068
H	-4.83355820053370	-0.39129412680119	1.84161216619640
H	-5.30832741619890	0.08927308248759	-2.22294196283930
H	-3.90220034259010	1.06605313666200	-1.76418116986791
H	-1.41748534825853	-0.19052372905857	-0.77664193568248
H	-1.29790881597081	0.41772631435215	0.86679640959976
H	-2.19441245949416	1.33674067205028	-0.32746466007058

6B_e, Water

C	-5.53213091694137	1.16852930752267	-0.35302351298903
C	-4.69167877551452	0.43009237820069	-1.38230634677981
C	-4.72681564955762	1.54691246742939	0.86842477910518
C	-4.06935659559547	0.29847433754594	1.45476088283210
C	-3.20304456956059	-0.43697606429605	0.44235817406299
N	-4.05865233958639	-0.75944262737475	-0.75553000949348
H	-3.49635704541162	-1.24735524339213	-1.45391194365405
H	-4.78239207395234	-1.42321092235082	-0.47316135443012
C	-1.96362161864470	0.32370380473739	0.01632868297521
H	-2.91219253891112	-1.40629419258624	0.84141272840719
H	-6.39544066997997	0.56181060331207	-0.07047945538149
F	-6.01691528255678	2.30743603441315	-0.98126145029315
H	-5.38028470457312	2.00507471659790	1.60969408648374
H	-3.97736097949612	2.28698879511548	0.58231409177177
H	-3.44242709299115	0.56640720636279	2.30414746107577
H	-4.83821386418722	-0.38224356663921	1.82735305049406
H	-5.29876148346612	0.08514532639592	-2.21380803313863
H	-3.89923307564244	1.07506751416398	-1.75466893179034
H	-1.42870455875257	-0.20667167808294	-0.77111651271655
H	-1.30471984922306	0.40629389868457	0.87931739553618
H	-2.18548631545569	1.33151790424020	-0.32949378207755

6C_ae, Gas Phase

C	-5.44386598600272	1.21049820565204	-0.39150518759749
C	-4.57948054738933	0.48376218630107	-1.41832018357992
C	-4.64620243775690	1.55943203805268	0.85914264301716
C	-3.97906490201820	0.32165821745093	1.42840263232743
C	-3.17433296911227	-0.39778811807579	0.36030195049753
N	-4.00090332404159	-0.69322017481971	-0.78983838452569
H	-3.49661070585005	-1.25152861740122	-1.46485548129566
C	-5.40040714841048	0.05753716887908	-2.62131639055294
H	-3.79122795777344	1.18453352717844	-1.74929726691535
H	-2.31604337350829	0.25316749324062	0.12004856455378
H	-2.78435305114937	-1.32854442159632	0.77192213222486
H	-6.27577221108910	0.55644913624665	-0.12053646130944
H	-5.86145145160371	2.11377124183725	-0.83998856660609
H	-5.27978986268810	2.01537666490062	1.62062607647576
H	-3.86118427795339	2.28084809762439	0.61266587240772
H	-3.32379400195373	0.58307438712250	2.26242770170228
F	-4.95258313968440	-0.52780504464249	1.93297796761044
H	-4.78657175602799	-0.46958918015278	-3.35372410218301
H	-5.84731970684160	0.92205817406993	-3.11239238150469
H	-6.19757118914535	-0.61342098186789	-2.29873113474669

6C_ae, Water

C	-5.44770766586366	1.20886331572973	-0.39039410373948
C	-4.58730050884607	0.47211825678826	-1.41218888987446
C	-4.65197086145248	1.55563505270879	0.86270571743938
C	-3.96815740398714	0.32804906710731	1.42605842934333
C	-3.17340891970226	-0.39633542157358	0.35955650934391
N	-4.01498017451758	-0.71470415735628	-0.78180265233987
H	-3.47536592200286	-1.23383018159670	-1.46401656559683
C	-5.40235980403620	0.05829716411640	-2.62325002484527
H	-3.78694151746836	1.15889738038880	-1.73496713139247
H	-2.33141301114602	0.26139606414504	0.09481491947504
H	-2.76114671792158	-1.31660873280228	0.77309310698258
H	-6.29467060100419	0.56946980843228	-0.12647251585832
H	-5.85065301871138	2.11641380054385	-0.84307066049602
H	-5.28524707169494	2.00889099082188	1.62634849461593
H	-3.87005823343604	2.27965691013988	0.61703839868103
H	-3.31826547567952	0.58727177781852	2.26201118776707
F	-4.94555646185723	-0.54014110816371	1.93998660771693
H	-4.78939829469738	-0.49030346096380	-3.34029527100387
H	-5.81676045696839	0.93322919624499	-3.12487188463889
H	-6.22716787900671	-0.58599572252938	-2.31227367157971

6C_aa, Gas Phase

C	-5.45339944700565	1.23202317127429	-0.38175095390784
C	-4.60745292417157	0.45722134919513	-1.39513526188251
C	-4.64438481936257	1.57227542929950	0.86709864315813
C	-3.98202553254370	0.33225165495591	1.43667438602061
C	-3.19324434741328	-0.40528635565552	0.36737025139501
N	-3.98168535676895	-0.72219413471120	-0.80639749647684
H	-4.69786345214589	-1.39738687963513	-0.56002902494253
C	-5.41542080256489	0.04565355006745	-2.61148363275872
H	-3.78655329832997	1.10850131243733	-1.72226124484775
H	-2.35252249389726	0.22731472810127	0.06350662820966
H	-2.77723559096311	-1.32328063101730	0.78268033055976
H	-6.31129380827834	0.61331945923704	-0.10249886404308
H	-5.84626802520996	2.14350379063977	-0.83707884693460
H	-5.27016801528627	2.03649771553820	1.62991537410382
H	-3.85442943468436	2.28673991834160	0.61557507378755
H	-3.34510976496426	0.57933509910205	2.28874257197751
F	-4.97349454504485	-0.52865343553963	1.90841296948798
H	-4.78068410839440	-0.46095357062738	-3.33716880983611
H	-5.87530725185686	0.91191597753423	-3.08729633707649
H	-6.21406698111386	-0.64014814853760	-2.31867575599353

6C_aa, Water

C	-5.45877270347251	1.22042138840622	-0.38254759340352
C	-4.60842199825778	0.45331439920677	-1.39595644120832
C	-4.64927783712891	1.56817974900165	0.86390247737977
C	-3.96810074884355	0.34252592197618	1.43511407431059
C	-3.19584733619796	-0.41260858652893	0.36953216358631
N	-3.99318488191023	-0.73566444528695	-0.80290769984945
H	-4.72716310335721	-1.38619353116583	-0.53741266877289
C	-5.40969625629847	0.05410198637267	-2.62098648534258
H	-3.78272146914369	1.10417211339426	-1.70861528509635
H	-2.35467989243137	0.21496537782598	0.05968438940215
H	-2.78380339099356	-1.32867574515414	0.79318975987633
H	-6.31490061941216	0.59590063419373	-0.10842263858079
H	-5.85402137077274	2.13027578208376	-0.83823323657151
H	-5.27409071297961	2.03087090649086	1.62875352188066
H	-3.86571781312383	2.28695521904444	0.60711236299441
H	-3.31984624661068	0.60003485174100	2.27322390425941
F	-4.95535435290200	-0.52255767053413	1.94516670036025
H	-4.77155055586253	-0.43379001457458	-3.35793347268335
H	-5.87095931075102	0.92763671853029	-3.08236869258020
H	-6.20449939955019	-0.64121505502321	-2.34009513996088

6C_ee, Gas Phase

C	-5.54247238533095	1.15231455046734	-0.36024583551627
C	-4.66740153177576	0.43769317177310	-1.37497042907554
C	-4.73837879830879	1.54109684832817	0.86168770327662
C	-4.04811086992162	0.30761376062924	1.44344294895687
C	-3.21476142898492	-0.43458830415035	0.39629101607909
N	-4.07482599515244	-0.73002659563046	-0.74700957838392
H	-3.58551103142044	-1.29405875386683	-1.42786751048050
C	-1.92635707574210	0.31025716732515	0.04107548523903
H	-2.93178337997263	-1.39916767350523	0.82447997428446
H	-6.37882835706007	0.50734199376009	-0.07854610718541
F	-6.07332726853437	2.28912842365704	-0.95191176397963
H	-5.38844703733433	2.00502297597963	1.60435569283409
H	-4.00377358509513	2.29167932008149	0.55940379828924
H	-3.41475062921515	0.59395665607353	2.28435469687124
H	-4.80599242977394	-0.37953541578594	1.82605242059704
H	-5.28047358821667	0.11286373355454	-2.21535479122424
H	-3.93701750592503	1.16581771357176	-1.75599561968292
H	-1.35895559447937	-0.24927423263242	-0.70427522594220
H	-1.29709321975691	0.42805053401659	0.92436727825836
H	-2.12245828799938	1.30327412635356	-0.36283415321540

6C_ee, Water

C	-5.54092513135678	1.14358786507266	-0.35506059061375
C	-4.66728651129886	0.43591376590582	-1.37289721373901
C	-4.73872765783219	1.54413765661385	0.86091317371922
C	-4.05002411936986	0.30988190255404	1.44460124912695
C	-3.21837595858194	-0.43589772271245	0.39948003446054
N	-4.07729425188499	-0.73961369387911	-0.74919766757836
H	-3.55508978301749	-1.26560048422642	-1.43884484801956
C	-1.93208381785645	0.30670916003994	0.03707329364252
H	-2.93197044022333	-1.39794917931881	0.83003719589681
H	-6.38201357472973	0.50672786767414	-0.07248706760328
F	-6.08249327628414	2.28655597746917	-0.95648743210904
H	-5.38740639720352	2.00786371391077	1.60498620680570
H	-3.99944028426385	2.28843961682828	0.55400393258827
H	-3.41330319183968	0.59963040616549	2.28136647070973
H	-4.80995525572416	-0.37118295408867	1.83524856549503
H	-5.27513876099341	0.11690255145629	-2.21940599494624
H	-3.92768073745031	1.15967960983948	-1.74190450181865
H	-1.37443138959747	-0.25517920155034	-0.71389956779671
H	-1.29985028913345	0.41606736509887	0.91943449635934
H	-2.12722917135836	1.30278577714700	-0.36045973457950

6C_ea, Gas Phase

C	-5.54440723888623	1.17020819480989	-0.34890718307124
C	-4.69277956707711	0.41010581173302	-1.35543569442041
C	-4.73353825569315	1.55299347898607	0.87204442370987
C	-4.05939212005589	0.31658351787855	1.46739907643532
C	-3.23937079309129	-0.44087697265642	0.41401351372199
N	-4.03407367044534	-0.74815708133641	-0.77587249058354
H	-4.71642803532003	-1.46331463010470	-0.55757548550825
C	-1.96190006654365	0.30129789960618	0.03381454891200
H	-2.93982886825799	-1.40262836659671	0.83585024720096
H	-6.40259384383940	0.55603674531154	-0.05472068558662
F	-6.04929221145448	2.31358756746080	-0.95175470156284
H	-5.37745611279836	2.03517710500102	1.60815523094553
H	-3.98617347996675	2.28863407699940	0.56519564361671
H	-3.42658429595205	0.60030734019731	2.31074635551012
H	-4.82613228286963	-0.35883675084442	1.85886266375077
H	-5.30364170509496	0.08954051283134	-2.19909545299389
H	-3.93806806062747	1.09503519604241	-1.75014670093349
H	-1.41668743691877	-0.25789802020393	-0.72512896859275
H	-1.32144044238084	0.41998793193341	0.90835950612597
H	-2.16262151272661	1.29663644295165	-0.36208384667622

6C_ea, Water

C	-5.54156320996501	1.15963517532666	-0.34450173447452
C	-4.69044155164937	0.41340279012376	-1.35845518619382
C	-4.73187797842051	1.55307348976419	0.86997070355518
C	-4.05859295230785	0.31405450821670	1.46193538796901
C	-3.23612212878166	-0.44080245617755	0.41061813981079
N	-4.03657511503399	-0.75293740221406	-0.78047117141595
H	-4.74207201006211	-1.43701597077448	-0.52953683184968
C	-1.95921801368042	0.30230333888881	0.03239848652903
H	-2.94050723108487	-1.40208881678630	0.83513139216668
H	-6.39833546504410	0.54592941598949	-0.05386728077639
F	-6.06010680424484	2.31071446253020	-0.95438282115622
H	-5.37602272688595	2.02936706038698	1.60992056454410
H	-3.98247599075464	2.28658386736046	0.56163323855760
H	-3.42590238592486	0.59612625051633	2.30541607101500
H	-4.82698603331468	-0.36216565541205	1.84705021196341
H	-5.30448788668738	0.09621177258863	-2.20126923132313
H	-3.93229827542435	1.10015919661249	-1.74390317490221
H	-1.40001864181584	-0.26028173207632	-0.71534488564884
H	-1.32875576347980	0.42757303853302	0.91333426076533
H	-2.16004983543777	1.29457766660304	-0.37195613913539

7A_a, Gas Phase

C	-5.46371274550601	1.57921187873227	0.40971136785179
C	-4.96830282732472	0.98180006697679	-0.90338122829461
C	-4.33058161546097	2.21919245713745	1.18887768272937
C	-3.17451119741827	1.23950952738996	1.37767259022446
H	-4.70504440005897	2.56633346637615	2.15129649391743
H	-3.99196361631863	3.09933049158707	0.63426812128283
C	-2.77427036605278	0.65411484181187	0.02623864104449
N	-3.91501555378476	0.01570750784429	-0.63246513535212
C	-4.21075172806306	-1.29337761601261	-0.40110942235306
O	-3.56680595207382	-2.02526818867308	0.29341526810936
C	-5.41378362119894	-1.89724229577402	-1.18994399319338

F	-5.39585229496729	-1.51724767043493	-2.47340835971453
F	-6.58973451647026	-1.53172191183362	-0.67485041638981
F	-5.34215882332285	-3.21231340421090	-1.15057337361080
H	-6.26592978694241	2.29272802863718	0.21440989999877
F	-5.99556391757406	0.54877249306903	1.16773388296503
H	-2.50314425102836	1.47830887072347	-0.64035662204319
C	-1.46794136882766	-0.14625468082415	0.12623553528175
H	-3.46815771202791	0.42501196528139	2.04017557819396
H	-2.31878177292974	1.74385170420382	1.82736141284661
H	-5.78907839487356	0.51357361250153	-1.43432955601232
H	-4.56939670522351	1.77595231578962	-1.53781585576294
F	-1.32931535522057	-0.83532380753479	1.25015730490875
F	-0.46246629296870	0.75340268226591	0.11627989976898
F	-1.28102518436216	-0.95596233502972	-0.90931971639680

7A_a, Chloroform

C	-5.46260981471200	1.58729493809566	0.40590259244202
C	-4.96985751136491	0.97928984915273	-0.90041109348150
C	-4.33191216201635	2.22308278503197	1.18723582165441
C	-3.17267415170271	1.24822125843554	1.38081251439097
H	-4.70651636325263	2.57149376544828	2.14895887687987
H	-3.99418929980829	3.10081984440624	0.63018694696552
C	-2.77497470667162	0.65300403219308	0.03309369830041
N	-3.92183744173733	0.00580661321001	-0.61139314685391
C	-4.20209689266018	-1.29896047762230	-0.41632115522854
O	-3.54080262960161	-2.05792103075234	0.24494667317234
C	-5.41848372129286	-1.89412014063285	-1.19133188347366
F	-5.42272861245446	-1.51292753919724	-2.47196633389023
F	-6.58674640195611	-1.54158817129504	-0.65309579481688
F	-5.34332429768590	-3.21410205491547	-1.16147362537253
H	-6.26135426896446	2.30016553591871	0.20410655458444
F	-6.00719949948773	0.55477922216551	1.16986459204470
H	-2.51172493198878	1.47088869993010	-0.64287210585157
C	-1.47082862483608	-0.14285663274149	0.13255108019705
H	-3.45843072648965	0.44136374522259	2.05631977299264
H	-2.31877047092630	1.76530464389298	1.81879063226370
H	-5.79197897558087	0.51639091378151	-1.43278435816839
H	-4.55901238264721	1.76488394149820	-1.53502638825933
F	-1.33377435445744	-0.85294396645039	1.24885364047146
F	-0.46153079482276	0.75029215580242	0.14183499087231
F	-1.26993096288172	-0.94557193057842	-0.91050250183529

7A_e, Gas Phase

C	-5.37384456323784	1.72147630971147	0.47773897051460
C	-5.06848369469930	0.90597869836897	-0.78112308664320
C	-4.13141168700660	2.30917866812984	1.14591079467008
C	-3.07561984978118	1.24186974657340	1.34268291064631
H	-4.40031733739991	2.74274403422069	2.10894982848764
H	-3.69995243886817	3.10316988691542	0.53528153088493
C	-2.75264785280296	0.54977006539662	0.02655405797671
N	-3.96654072764758	-0.01547814703580	-0.54378819020378
C	-4.23901171981720	-1.34197278021786	-0.65640981020031
O	-5.27839723597490	-1.79145316807777	-1.05672469942568
C	-3.14440849774968	-2.35037267617055	-0.20908129310654
F	-2.01953561383694	-2.21217529569016	-0.91492867979428
F	-3.58261240339442	-3.58044688652096	-0.37005215523128
F	-2.84610429837426	-2.18291283178725	1.08671215242699
H	-6.08736545476366	2.50901860758711	0.23669666337267
H	-5.87466101967076	1.04505291432240	1.17346767167550
H	-5.93593916629687	0.29935246486334	-1.03624988173798
C	-4.84408530723467	1.77047871192501	-2.01878530593905
H	-2.33115888543954	1.27931928214195	-0.66534541128190
H	-2.00925289728217	-0.22143595674325	0.18371777934920
H	-3.40300158764552	0.49659897778007	2.07419264257699
F	-1.91345370033082	1.81500635511278	1.81601445268357
F	-3.83951480565323	2.64915136918406	-1.88361916730980
F	-5.94858850308662	2.47831515528490	-2.27556628742756
F	-4.57728075200517	1.02470649472557	-3.08757548696383

7A_e, Chloroform

C	-5.36834771648390	1.74428964041601	0.48673638829950
C	-5.07826318674059	0.90528729019829	-0.75924765622403
C	-4.12117265907894	2.33254494404640	1.14337953650115
C	-3.08830896219848	1.25082544888850	1.36099264909939
H	-4.39159548536360	2.78409704621181	2.09739739910898
H	-3.67824259699270	3.10920512272767	0.51832115121792
C	-2.76112705072593	0.54322793041174	0.05527199977157
N	-3.97746430906775	-0.02022987249067	-0.51709661015328
C	-4.22505258556281	-1.33711584995037	-0.68101344074186
O	-5.24605577253491	-1.79373406732937	-1.13339375027230
C	-3.13260769882883	-2.35007757723728	-0.23613064036167
F	-2.00287799599031	-2.20111248327587	-0.93054182648352
F	-3.57020837505351	-3.58053320719309	-0.42979510907632
F	-2.84645497186516	-2.21688155455738	1.06294702049379
H	-6.07556630684408	2.53364865339905	0.23488585930338
H	-5.87118081120639	1.08363083776604	1.19482992395360
H	-5.95654278656124	0.30725788339369	-0.99396883085079
C	-4.85565301372674	1.74244621998880	-2.01438488334045
H	-2.33270140427310	1.26210061398722	-0.64338151981024
H	-2.02285650565947	-0.23011140787434	0.22441465832689
H	-3.42907408248789	0.52378760423370	2.10251855524674
F	-1.91156050061396	1.81259232007001	1.83543057666619
F	-3.84276319014948	2.61451064589078	-1.91067278872776
F	-5.95494864060291	2.45392588201627	-2.28511398861090
F	-4.60256339088727	0.97135793626240	-3.07371467333598

7B_a, Gas Phase

C	-5.30844272664122	1.45988350820399	-0.35915459731360
C	-4.49778577470216	0.66624709928890	-1.36970052713444
C	-4.51480017926743	1.73300781243147	0.91650203752189
C	-3.94039010457307	0.44576924293747	1.47275111131856
C	-3.07212377008059	-0.26231818948120	0.44914494239733
N	-3.94334284999593	-0.57391898080233	-0.73050063347418
H	-5.16104312940432	2.19227759682916	1.66275863592631
H	-3.69441282151868	2.43029163235900	0.72943546046547
H	-3.43120723069014	-1.12294470286982	-1.42505012463734
H	-4.72543643131071	-1.15098482742711	-0.39666467427139
H	-5.61166419758794	2.39464139134118	-0.82806002830454
H	-6.21952902579780	0.91034087193289	-0.11605136688126
C	-5.32411456178951	0.21138907441327	-2.57006476379017
H	-3.64449716133111	1.23568021748972	-1.74248699310105
F	-4.97841947000376	-0.43022715609952	1.75225998136021
H	-3.37348868387898	0.61289918049348	2.38779397094280
H	-2.25173687167667	0.36783202036953	0.11002568521162
H	-2.68553058290142	-1.20195369654637	0.83466256030101
F	-6.30436848547920	-0.59910582243240	-2.16782506357032
F	-4.54856616621010	-0.47758672447932	-3.41140839931495
F	-5.83961977515922	1.24657045204799	-3.19694721365196

7B_a, Water

C	-5.33266414799742	1.41732094824966	-0.35224645666071
C	-4.50090125858613	0.64201839595290	-1.35931148230124
C	-4.52515317112785	1.72996856454205	0.90288390676705
C	-3.91851045389156	0.47254315413276	1.48360500328987
C	-3.09926961043931	-0.27800606215200	0.46043558174142
N	-3.95084621124944	-0.59054562968032	-0.72891760734420
H	-5.16074735837737	2.20446099293796	1.64871142143558
H	-3.71272332394276	2.42256988083807	0.67386725101449
H	-3.39346574790621	-1.11933600827359	-1.40321301505688
H	-4.71366582980485	-1.20733429944313	-0.43211261941265
H	-5.66631767081165	2.34070597472645	-0.82093776814058
H	-6.21880404248037	0.83641438607897	-0.09131847564258
C	-5.30759331529264	0.23970905017464	-2.58366530978436
H	-3.64810089596919	1.22538971263561	-1.70799644820354
F	-4.94603228849817	-0.37934595773165	1.89622571630326
H	-3.29609944213425	0.68902804670289	2.34988890378230
H	-2.26485465408427	0.32976735367652	0.11830924335799
H	-2.73454266390934	-1.22229724513794	0.85157368814973
F	-6.33829056751345	-0.54332251953461	-2.25255617845018
F	-4.55636105052918	-0.43822476128701	-3.45452132288240
F	-5.78557629545454	1.31630602259177	-3.19728403196236

7B_e, Gas Phase

C	-5.54480557166163	1.36085944235390	-0.31819601961937
C	-4.68119770085968	0.62336646136388	-1.33791770410275
C	-4.75181122771340	1.75855457281722	0.91080017226005

C	-4.03888102150632	0.54953228269323	1.51946241566490
C	-3.21144052070700	-0.25211647066174	0.52544723637809
N	-4.03487190170720	-0.57120313346841	-0.69283725793469
H	-5.42748694519609	2.18449497769982	1.65119290983898
H	-4.04456774942993	2.53817140427976	0.63184020813114
H	-3.44863029162991	-1.05688153799657	-1.37756328172118
H	-4.76385773753492	-1.23175361449431	-0.41049213383690
F	-6.02223181403622	2.46755024497791	-0.95950255162531
H	-6.40237488426415	0.73816373480053	-0.03746952709406
C	-1.89458582884236	0.40470052009767	0.09267411988795
H	-2.91931815867706	-1.21051097883152	0.95579793045639
H	-5.27468152597966	0.26580297917152	-2.17553691560095
H	-4.76968727435954	-0.12881031916256	1.96771934458026
H	-3.38157887249387	0.86104911344648	2.33143967615499
H	-3.88958528130768	1.27375017321725	-1.70270107117215
F	-2.04019003083547	1.66816203501691	-0.29300473682633
F	-1.38542378869309	-0.27930223369020	-0.93936181055622
F	-1.04737187256478	0.36536034636923	1.10155899673714

7B_e, Water

C	-5.54383905514661	1.35253609783839	-0.30810752901213
C	-4.69108620089197	0.62255125388871	-1.33392283229361
C	-4.74169760449100	1.76296012736724	0.90565198589742
C	-4.03323124463457	0.54886501691518	1.50616723280602
C	-3.21294726840367	-0.25089891069135	0.50518103672979
N	-4.03896714398391	-0.55928061998474	-0.69815580666700
H	-5.40805768128537	2.18984020327638	1.65375594585331
H	-4.02957219878666	2.53385174694612	0.61337575183187
H	-3.48367519099960	-1.06361712825665	-1.39397436332953
H	-4.76068857755321	-1.22417166042637	-0.40647343325624
F	-6.04764236623493	2.47486175733588	-0.94524327290461
H	-6.39460397902338	0.73162077506898	-0.01924857685238
C	-1.89394461545560	0.40052477754656	0.10252543436822
H	-2.93333895587003	-1.21275275121568	0.93254186826988
H	-5.29352611509254	0.25100067084052	-2.15686130507357
H	-4.77179547341227	-0.13403720006996	1.92957717905784
H	-3.38263378715826	0.85048064502169	2.32546701968380
H	-3.91079053958068	1.27558981585838	-1.71640269668926
F	-2.01575961059582	1.66615567260966	-0.30535747251476
F	-1.31795504728531	-0.28092900433943	-0.89200205343914
F	-1.06882734411458	0.39378871447049	1.14485588753405

7C_ae, Gas Phase

C	-5.33052998437363	1.40133893483788	-0.35456587544443
C	-4.47137911907797	0.64246108426863	-1.36176756599519
C	-4.52558688814046	1.72397757851092	0.89870967750949
C	-3.92266932090495	0.45964752468922	1.48059348508334
C	-3.12238183364738	-0.28395868296360	0.42722126944179
N	-3.92425473155761	-0.54829903107325	-0.75194624692363
H	-5.15314041921242	2.20782671145377	1.64712358608013
H	-3.70917054572140	2.41267923200677	0.66236657808015
H	-3.40437865115948	-1.09573109592305	-1.42376213678385
H	-5.71237143697003	2.31455119088234	-0.80984428713315
H	-6.17928182823067	0.77365195095852	-0.08208984748556
C	-5.29975431060246	0.23699861729494	-2.56772106042514
H	-3.69388623486024	1.32046435122610	-1.75371273336447
F	-4.93986032934525	-0.34985392925358	1.95762656259510
H	-3.27479521243052	0.69272995876119	2.32852543656263
H	-2.23379520136778	0.33263494326568	0.21273879769319
H	-2.78033275295376	-1.23284263096365	0.83832369618919
F	-6.37845729965239	-0.47291865435923	-2.24507911180498
F	-4.57412350924069	-0.50559001977385	-3.41907637792854
F	-5.71600039055090	1.31727196615424	-3.24048384594609

7C_ae, Water

C	-5.32534737014020	1.40895780846473	-0.35952954886482
C	-4.46994304306605	0.63664393808563	-1.36013640553392
C	-4.52693644683233	1.72178889127876	0.90052734919926
C	-3.92033020316152	0.46172766497701	1.47837551786662
C	-3.12486362274423	-0.28471835628859	0.43027771658889
N	-3.93374326272250	-0.56607215883311	-0.74996589766674
H	-5.15719573560407	2.20639871775081	1.64615214197168
H	-3.70712465784342	2.40705528308379	0.67039721452699
H	-3.36472201303899	-1.06879752234533	-1.42021315315547
H	-5.68182951918423	2.33160608706188	-0.81570470249262
H	-6.19367206421897	0.80346134264361	-0.09594286706295
C	-5.30049663467401	0.23565329467795	-2.56172033848023
H	-3.68264523991734	1.29949975060690	-1.75022078598075
F	-4.95037171043611	-0.36245447359953	1.95290813903100
H	-3.28312528842958	0.68701369312024	2.33350648211207
H	-2.24818989483222	0.33714829692285	0.20001826698440
H	-2.76774702577604	-1.22782909794859	0.84131234414579
F	-6.37549658004323	-0.49284544634136	-2.23664355589699
F	-4.58807198431271	-0.49761486551776	-3.43234124201453
F	-5.74429770302223	1.31041715220009	-3.22787667527768

7C_aa, Gas Phase

C	-5.34099849688281	1.43183179001673	-0.35454325571070
C	-4.50160004774296	0.60701310581378	-1.33151929317488
C	-4.51953428084256	1.74228416796135	0.89618974149734
C	-3.91599982120435	0.48199794524208	1.48750800150226
C	-3.15532784536025	-0.30583687308870	0.43325718025709
N	-3.96055345170475	-0.60132447389771	-0.73831015953254
H	-5.13332654918883	2.23966263642263	1.64707952052526
H	-3.69911202583591	2.42196537701158	0.64664040936951
H	-4.71957814959683	-1.22054017811494	-0.47446075111332
H	-5.68100601646467	2.35583941717800	-0.82092271416912
H	-6.22494305585861	0.85402049212810	-0.07587227068537
C	-5.28546157194060	0.23078391581716	-2.57518381901905
H	-3.65905605288634	1.21141412518813	-1.68633000385896
F	-4.94646966116828	-0.32562525597544	1.96341371413955
H	-3.27259279317574	0.71470938260274	2.33859283697607
H	-2.28184704690297	0.27705463300098	0.12480534923573
H	-2.79151951043913	-1.23984894576992	0.85972466707236
F	-6.25956089497585	-0.64607599509476	-2.29027598304351
F	-4.51367423576778	-0.32028260894739	-3.51012927431959
F	-5.86697849206077	1.30637734250562	-3.12199389594813

7C_aa, Water

C	-5.35424121509003	1.42064474617776	-0.35003814260188
C	-4.50897264303354	0.60445705182968	-1.32672008243657
C	-4.52646848942400	1.73644465975033	0.89560641744175
C	-3.89758244773095	0.49020994455069	1.48332516736245
C	-3.15606562914673	-0.31064054800056	0.42898306249954
N	-3.98069917552207	-0.61460107958158	-0.73488767970634
H	-5.13857365807742	2.22852591110406	1.65132127355581
H	-3.71543318058160	2.42284238961472	0.63771288958980
H	-4.75343064027313	-1.20345968677402	-0.43779945978750
H	-5.69469227511089	2.34671694162908	-0.81156186869457
H	-6.23615934102000	0.83851980655636	-0.07221655623837
C	-5.27441724845506	0.24151217061823	-2.58069974492762
H	-3.66191462362230	1.21015484619352	-1.66518325337696
F	-4.91954185301293	-0.32577517177234	1.99969495011574
H	-3.23963814004729	0.73408100558647	2.31767861710079
H	-2.28786968553794	0.27036985028618	0.10692957486887
H	-2.79119284578634	-1.24174699062585	0.86047298032888
F	-6.26722321267162	-0.62711078674965	-2.32854441195671
F	-4.49206912493413	-0.32451593831820	-3.50743774331298
F	-5.83295457092201	1.31879087792515	-3.14896598982414

7C_ee, Gas Phase

C	-5.55569020384134	1.33350911699343	-0.30943489692465
C	-4.67651565791121	0.63628797603343	-1.33243546590042
C	-4.74173404218902	1.75876099703175	0.89369259996835
C	-4.01019492337766	0.55539809254060	1.48933615983034
C	-3.21968128851011	-0.24719890204772	0.45468705944098
N	-4.03530532420250	-0.51049862763824	-0.70624855290082
H	-5.39466057763570	2.20518146710937	1.64429874327245
H	-4.03625361475177	2.52604644698443	0.57251385683410
H	-3.55937885551124	-1.09461618889763	-1.37756121827078
F	-6.12486297675480	2.44751432701711	-0.90266478625904
H	-6.37015475881061	0.66911164249341	-0.00835967838465
C	-1.86590794840434	0.38271794154761	0.13446342945386
H	-2.95263435387468	-1.20892459929673	0.90055689035494
H	-5.29663070484465	0.27385811635476	-2.15141611131436
H	-4.74355359590517	-0.12822484068345	1.92079937725767

H	-3.35175471708324	0.87095692358858	2.29817623557329
H	-3.97718361170845	1.37699803046519	-1.73777166447774
F	-1.94864981051810	1.63563547910136	-0.33199322596894
F	-1.22198570211568	-0.33729229482638	-0.79556567506021
F	-1.08360733204974	0.41639889612912	1.22046692347562

7C_ee, Water

C	-5.55318600463420	1.32793753446789	-0.30626596111964
C	-4.67661282380503	0.63448776525623	-1.33165411572210
C	-4.74319249369381	1.75934326888264	0.89427238124415
C	-4.01010900097464	0.55630397871106	1.48956107337889
C	-3.22157378923012	-0.24919467467887	0.45581762496456
N	-4.03865210703835	-0.51510487526103	-0.70565976502101
H	-5.39676112776786	2.19960148480602	1.64777450245366
H	-4.03769463689178	2.52752702875059	0.57373717111982
H	-3.56127539182225	-1.09466720683289	-1.38168163209198
F	-6.12448451576944	2.45290055368430	-0.90783133120117
H	-6.37567863572779	0.67455129127863	-0.00804413023799
C	-1.87055704375439	0.38034023995956	0.13266620432577
H	-2.95159713220368	-1.20876081333782	0.90292964145277
H	-5.29258273717755	0.27368002895049	-2.15423208972847
H	-4.74275236617793	-0.12622582588871	1.92317263323343
H	-3.35428527768863	0.87520130799461	2.29886332184430
H	-3.97170291441292	1.37260936749339	-1.73260108108794
F	-1.94479809690809	1.63760696365162	-0.33182505305675
F	-1.21884232342099	-0.33225153995034	-0.79976325358076
F	-1.08000158090058	0.41573412206263	1.21630385883045

7C_ea, Gas Phase

C	-5.57425451588622	1.35749836182805	-0.27256169085031
C	-4.72826283110564	0.62732300422549	-1.30462448705514
C	-4.72787169964769	1.77965890227196	0.90970423400379
C	-4.01924974400922	0.56491142604081	1.50833385816885
C	-3.24870188716992	-0.25051555584975	0.46151997452971
N	-3.99792183733190	-0.50472722704157	-0.75270222936628
H	-5.35526391437303	2.25641398801209	1.66320105028828
H	-4.00476760133084	2.52074352151437	0.56547576732735
H	-4.59826560043282	-1.30688382920899	-0.63218868685206
F	-6.13703004781361	2.47438847826835	-0.86731172959236
H	-6.39951618583814	0.71798536506265	0.05931556190751
C	-1.88697558889920	0.35569830989876	0.13196238162369
H	-2.99047126689298	-1.21920618093309	0.89351917016867
H	-5.35451543552137	0.26796190081241	-2.11987650110792
H	-4.76784760885012	-0.10610233745046	1.93789378280324
H	-3.36103279408127	0.86487099504478	2.32318839664704
H	-4.02400002516302	1.34387425359966	-1.73045918732078
F	-1.97144080947427	1.55645837050392	-0.45949029650496
F	-1.17864223355738	-0.43243684811168	-0.67354644845448
F	-1.16972837262136	0.52388510151224	1.25347707963617

7C_ea, Water

C	-5.57299639000181	1.34592049189871	-0.26989897176113
C	-4.72411127954824	0.63026220690891	-1.30656599053154
C	-4.73281715441421	1.78104913087509	0.90827035812706
C	-4.01959068868074	0.56809941042303	1.50453473326233
C	-3.24608861151541	-0.24703139493175	0.46177284742495
N	-3.99025567134297	-0.50521713688293	-0.75889480902543
H	-5.36394067727815	2.24694891014330	1.66535382789485
H	-4.01398508706674	2.52768551696032	0.56494278797174
H	-4.61754571313974	-1.28301581441998	-0.60089454587846
F	-6.14690171410478	2.47018880484470	-0.87353449635900
H	-6.39744874330772	0.70705771433922	0.05634890548367
C	-1.88606271239790	0.35119083611354	0.13444983948858
H	-2.99608533514692	-1.21559149352453	0.89777745072406
H	-5.35077183847209	0.26841688979508	-2.12033309637189
H	-4.76629217589031	-0.10738138622887	1.92757660405717
H	-3.36674977881437	0.86958268707518	2.32302170877138
H	-4.02242765009052	1.35387497717304	-1.72646263400503
F	-1.95115776167742	1.55729063784996	-0.45422139211659
F	-1.17913340100558	-0.43558558453599	-0.68539223326599
F	-1.15139761610438	0.50805459612399	1.24697910610927

8A_a, Gas Phase

C	-5.49967351429139	1.36069973752981	-0.01931461824505
C	-4.94320879322033	0.60894919517504	-1.21591646230291
C	-4.41321767731005	1.73034003572787	0.98254712644670
C	-3.54917618030946	0.51388029936608	1.35272782208288
C	-3.00544793110638	-0.12377473011380	0.07424512978805
N	-4.09378246581906	-0.49698775680204	-0.80961015416283
C	-4.31734204935890	-1.71156618866080	-1.35119250594221
O	-5.19198752048963	-1.96370621524115	-2.13891474451623
C	-3.37116229237096	-2.87155072174251	-0.93602326248657
F	-3.38536714852031	-3.07879574022579	0.38623543361732
F	-2.10565815516626	-2.62013726969722	-1.29750785640175
F	-3.74638220745675	-3.98761106561664	-1.52585236325630
C	-4.98318425089016	2.45845880819232	2.19087616203576
H	-3.75722609148998	2.42644885244766	0.44499531887608
C	-4.25768002388594	-0.52353391545056	2.21788427924644
H	-2.68541838366156	0.89219441412917	1.90708720053356
H	-5.75240904323864	0.22268925085163	-1.82831144134562
H	-4.35545384487091	1.31253006130769	-1.81665384768012
H	-5.99984149194070	2.26594275151119	-0.37518815432037
F	-6.47294489116655	0.58336425636753	0.59095672122656
H	-2.36684995835356	0.59124729321099	-0.45674838598482
H	-2.39910878371190	-0.98924573124573	0.31548943560281
H	-5.77263241349212	1.87766213074531	2.66480024045823
H	-5.40417208615847	3.42153111648683	1.89989222635862
H	-4.20213184751705	2.64131229882828	2.93031698644410
H	-5.08332688588282	-0.99036531100537	1.68293354356502
H	-4.66115319851937	-0.07434479109795	3.12402698399877
H	-3.55912086980077	-1.30690106497784	2.51179918636387

8A_a, Chloroform

C	-5.49314785483089	1.36768705963081	-0.02871798492558
C	-4.93254606953114	0.61951084422512	-1.22316250627268
C	-4.41607025011233	1.72758362136199	0.98315200623636
C	-3.55183220950768	0.51096857146127	1.35079389900987
C	-3.00059946010685	-0.12086130901977	0.07289025049352
N	-4.09072775665464	-0.49672722505469	-0.81504452055165
C	-4.33214646607498	-1.71023216418173	-1.32027711949062
O	-5.23809358802073	-1.98604706545013	-2.07715314929012
C	-3.36897720560853	-2.86570137027699	-0.93002685777302
F	-3.35548957107262	-3.08824987602907	0.38762346798151
F	-2.11473089879706	-2.61290940642797	-1.31984420180405
F	-3.75454243538733	-3.98356315989652	-1.52178510212944
C	-4.98883450095916	2.45360873467665	2.19166866679284
H	-3.75907681217873	2.42599138639251	0.45154459439849
C	-4.25794748004688	-0.53056590753945	2.21303157259448
H	-2.68942158405052	0.88857812214202	1.90619339256076
H	-5.73905863130708	0.25055714730867	-1.84811798547580
H	-4.32783283215645	1.31945193734192	-1.80753562585498
H	-5.99164274510685	2.27199785658019	-0.38414491166460
F	-6.47925482808490	0.58087850259069	0.57022794472055
H	-2.37103832103409	0.59787143242089	-0.45969085997438
H	-2.38997360331988	-0.98290291673188	0.31487093789278
H	-5.77042830673433	1.86925382244614	2.67510366758094
H	-5.41745918873935	3.41204855383135	1.89619620656232
H	-4.20401638198291	2.64463247717627	2.92520706879765
H	-5.07465861698338	-1.00868361305886	1.67286971068692
H	-4.67297602523806	-0.08077416524684	3.11397551967763
H	-3.55253637637262	-1.30467189067258	2.51573191922028

8A_e, Gas Phase

C	-5.44821095893474	1.15565304393099	-0.10480329720037
C	-4.69407038701665	0.65132772730399	-1.33247359007651
C	-4.46729504889139	1.57358880250257	1.00712852467415
C	-3.52749418119369	0.40314529144795	1.29114047803682
C	-2.83786005510041	-0.12257113112986	0.03596487337111
N	-3.83396005853413	-0.45708838892490	-0.96862071080747
C	-4.00273933953256	-1.74629470028314	-1.32551562669985
O	-3.34952046680877	-2.66900835765729	-0.91187729772883
C	-5.12040708060026	-2.06003831906833	-2.35976608785376
F	-4.93717036475828	-1.39586156243848	-3.50694823926528
F	-6.33059633415515	-1.73002317707474	-1.88446664533206
F	-5.13116984782528	-3.34786305857802	-2.63273024153690
H	-5.39358598618702	0.35848004547591	-2.10658670027492

H	-4.07007069735650	1.44643750591499	-1.75050594620453
H	-2.16891963878115	0.64572059685905	-0.35983581817755
H	-2.25486505330494	-1.00806909545861	0.26943640751332
C	-6.41625574424719	2.25860599706155	-0.50278950100901
H	-6.02861757275677	0.30594638569301	0.27099368439553
H	-4.08706504676945	-0.41371052651922	1.75497790321400
F	-2.54617079361917	0.78607181902487	2.18797896172449
H	-5.05109360612610	1.74562011977254	1.91618574514966
C	-3.70486705687101	2.85822823837478	0.69750679820112
H	-5.90387671737552	3.06526364499126	-1.02886675423812
H	-6.90943640898921	2.68450954899910	0.37122160917373
H	-7.18555870528998	1.86453283985678	-1.16760247798406
H	-3.13597556621300	2.79643609269878	-0.23085047262140
H	-3.00533338784561	3.08282422948065	1.50006033746592
H	-4.39333389491603	3.69739638774384	0.60936408409075

8A_e, Chloroform

C	-5.45352582789407	1.16083784421480	-0.08937024511502
C	-4.71241891740938	0.64118425339404	-1.31819012580053
C	-4.46465503190964	1.58510286374071	1.01314292551981
C	-3.53945001317503	0.40708671678655	1.29928857505356
C	-2.85234946764918	-0.12966112368292	0.04807247776213
N	-3.85731289301802	-0.47412266258279	-0.94721001020177
C	-3.99681841322573	-1.74525411614662	-1.33772246397979
O	-3.32187471342755	-2.67520494750467	-0.95321267405531
C	-5.10695482252257	-2.06099619868245	-2.37957117846904
F	-4.92795455271741	-1.38618279039106	-3.51936169569591
F	-6.32484977832350	-1.76525414759145	-1.91059850558994
F	-5.09143403975263	-3.35053373053410	-2.66944287526843
H	-5.42110151550355	0.34769274760351	-2.08343202341583
H	-4.08071066057440	1.42344975954776	-1.74648901558770
H	-2.19088154426866	0.63508131552873	-0.36532711022468
H	-2.26507507903327	-1.00931522747209	0.29015485154078
C	-6.41399342267847	2.26819921115750	-0.49417958478815
H	-6.03824963539874	0.32134757008805	0.30146406313497
H	-4.10319821017749	-0.39947188534828	1.77297288179647
F	-2.54361333315061	0.78345524763575	2.19483396543008
H	-5.04546389287126	1.76813568703180	1.92146657673349
C	-3.69519387649849	2.86098665785932	0.68496864933888
H	-5.89602369824060	3.06307295743875	-1.03257483283462
H	-6.89493440508510	2.70681714310174	0.38044844878184
H	-7.19072483115317	1.87237202370884	-1.14933483848530
H	-3.12663908083592	2.78208231007414	-0.24232062777796
H	-2.99737791539356	3.09885655101390	1.48588136555122
H	-4.38274042811194	3.69949597001055	0.58336302668671

8B_a, Gas Phase

C	-5.43279897469535	1.21801311953231	-0.50759097030802
C	-4.57835388731408	0.48794382381110	-1.52452247606867
C	-4.66584111025161	1.54386197555168	0.76716154777633
C	-3.99087517925445	0.29383201313193	1.36800069500382
C	-3.18808461982574	-0.44153612818461	0.30250623319743
N	-4.06804264852629	-0.75842439855789	-0.87510973535957
H	-3.56486897739639	-1.33198117711518	-1.55117329412411
H	-4.87646626648037	-1.30156110844000	-0.55495456909045
C	-5.54417018275148	2.28531426631244	1.76634014348112
H	-3.86373604371300	2.22287579574803	0.45439961789843
H	-3.25482251294170	0.65174647955175	2.09199753552298
C	-4.93503208206685	-0.65118012110555	2.10953981278494
H	-5.15921315366846	0.20842845674645	-2.39955035764382
H	-3.71819373423697	1.08471905910313	-1.82289380789254
F	-6.49797173823648	0.37047438183262	-0.23287281520452
H	-5.83676947681687	2.12645187860050	-0.95654824189487
H	-2.37155748915717	0.16639070608397	-0.08613082877779
H	-2.78837547648003	-1.38191903278500	0.67582651716336
H	-5.71326881811671	-1.07141285984481	1.47161879528099
H	-5.44629340117137	-0.12509922747698	2.91299686919727
H	-4.37401333971870	-1.46852113735589	2.56180425328047
H	-6.45732560047732	1.73342206307762	1.98275271757911
H	-5.82665201641359	3.26312590781489	1.37741515493220
H	-5.01054327028903	2.44279526396749	2.70386720326593

8B_a, Water

C	-5.43787098406545	1.22306777518140	-0.49784480047857
C	-4.60882191958474	0.45846038637662	-1.50294176648594
C	-4.66752009875955	1.54074821128062	0.77380674651851
C	-4.00150712537999	0.28940974753005	1.37404629478765
C	-3.18787014206254	-0.43004375977679	0.30829684325070
N	-4.04428674683731	-0.76125803671520	-0.86879884234960
H	-3.49837591089951	-1.27944986561934	-1.55651083497953
H	-4.80461933252637	-1.38377519428016	-0.58719529550137
C	-5.52585175360497	2.30450402995860	1.77221333346954
H	-3.85721802286506	2.20247735806870	0.44960922392408
H	-3.27553538724302	0.64273850583350	2.10983876458962
C	-4.95549055688152	-0.66389047204950	2.09009935455732
H	-5.20574306717116	0.15285054054901	-2.35676957221631
H	-3.77252281291624	1.06805131126767	-1.83921290346131
F	-6.56089862826763	0.44185485345867	-0.20241424520388
H	-5.79889533268959	2.13968554280546	-0.96445037027200
H	-2.38330099387222	0.19769302133991	-0.07070451385311
H	-2.77194387272316	-1.36329601045637	0.67841858318070
H	-5.69690515554992	-1.09835521490544	1.41953594844857
H	-5.49815366457785	-0.14501311559360	2.87869567965403
H	-4.39092672887366	-1.47543128614688	2.54894895169704
H	-6.43410898257382	1.75693011792310	2.02054706135551
H	-5.81444432762154	3.27294595622429	1.36259525509950
H	-4.97045845245319	2.47703559774569	2.69507110426887

8B_e, Gas Phase

C	-5.46601738688283	1.06390900419875	-0.47766967949466
C	-4.61400847897395	0.32738238253169	-1.49916982750301
C	-4.65792258184669	1.43449223475066	0.78179033703003
C	-4.01539396218857	0.16943027703020	1.34181661100191
C	-3.16714921531903	-0.56747758949866	0.30990923495969
N	-4.01435775550347	-0.90358315541404	-0.87630268923907
H	-3.45968130650685	-1.41332693269314	-1.56490760485117
H	-4.77076367322797	-1.52989892019014	-0.58913429262977
H	-5.20069562928879	0.00929927258879	-2.35870577337446
H	-3.77936881876770	0.93523090052007	-1.84729641533273
H	-2.35257632822291	0.06431914004652	-0.04032678826066
H	-2.76220019477923	-1.48772474243206	0.72303597225928
F	-3.18154741459283	0.45303612288153	2.38840692820730
H	-4.79640707969804	-0.50338700862396	1.71469213650093
H	-5.37609469564701	1.77804167100575	1.53026020498015
C	-3.64555420045699	2.55347556182477	0.56211433285135
H	-6.27240226839864	0.38491809293088	-0.17353201651467
C	-6.10646059618299	2.27944687048600	-1.13869604089665
H	-2.95641195307131	2.35967128501409	-0.26201335965804
H	-3.05434157915220	2.70626204964868	1.46243137091594
H	-4.15813218083961	3.48879535093792	0.34630558401993
H	-5.35439456914948	2.94270877484254	-1.56634562281040
H	-6.67538190342474	2.85035060947065	-0.40558466366653
H	-6.78840622788573	1.98399874814253	-1.93506793849467

8B_e, Water

C	-5.46366762446135	1.06687828270370	-0.47223585513163
C	-4.61219366301806	0.32719360643938	-1.49120730851043
C	-4.65695110562215	1.44097639634718	0.78637211411747
C	-4.02779516501245	0.16732386440057	1.33216764363851
C	-3.17403088880544	-0.56006725029777	0.30651233194375
N	-4.00959954119119	-0.89019513155846	-0.87513736207114
H	-3.44231533082001	-1.38260206497706	-1.56517120430508
H	-4.75108818949129	-1.54078051737659	-0.60740717702364
H	-5.20660907599244	-0.00015869834624	-2.34009088009707
H	-3.78473398073062	0.93537447651517	-1.85373847258241
H	-2.35520244486054	0.06767747494748	-0.03908689813751
H	-2.77946683509304	-1.48535219537586	0.71498526685350
F	-3.20269297397856	0.44679289638084	2.41202486247229
H	-4.81113251375632	-0.50306180060191	1.69374415808704
H	-5.37410425720768	1.78864193092086	1.53408432802950
C	-3.63649338938014	2.55078867164915	0.55702035437836
H	-6.26184245568374	0.38502908862608	-0.16076455093538
C	-6.10929614380830	2.27837783995207	-1.13178038645095
H	-2.95199772411632	2.33340866299154	-0.26397372304306
H	-3.04433753130009	2.71156687843455	1.45638712491296
H	-4.14619543087093	3.48468427006839	0.32542168831048

H	-5.35804895333183	2.93838481966254	-1.56710542781742
H	-6.67857697811176	2.85006516426956	-0.39835910117085
H	-6.78729780335576	1.96842333422487	-1.92665152546725

8C_ae, Gas Phase

C	-5.44376992748150	1.21291757300228	-0.49027089039399
C	-4.60175791060318	0.41956223168122	-1.47371424525571
C	-4.66357447818475	1.53860183284239	0.77802338291407
C	-3.99157567847418	0.28986642870016	1.37639270256515
C	-3.18107231332979	-0.40740190777368	0.28095447753127
N	-4.04404048454188	-0.75084839364078	-0.83350040783291
H	-3.57071415616713	-1.34078099228784	-1.50158944576626
C	-5.51150863309023	2.31617970151892	1.77446829248553
H	-3.84949200819496	2.19350251814186	0.44310547710147
H	-3.28237174350934	0.64577447325424	2.13074517704628
C	-4.95746648568252	-0.67672533894890	2.05526543643066
H	-5.22487927993380	0.11560842713683	-2.31449661712991
H	-3.82725933847494	1.10822929435738	-1.85425792355871
F	-6.59462469156646	0.49996542527301	-0.17950833355705
H	-5.77535985491258	2.14131097339519	-0.96468407495486
H	-2.35631121909033	0.25835502166141	-0.02572192444297
H	-2.73886526842667	-1.32305302440351	0.67620629361935
H	-5.63978883672602	-1.11801549124691	1.33201768580001
H	-5.54736568598026	-0.17623676652453	2.82300310091900
H	-4.40105830636019	-1.48163224232860	2.53799804993281
H	-6.42814882375574	1.77960248275565	2.01431699120127
H	-5.79030705038031	3.28946338169418	1.36770815424300
H	-4.96063782513325	2.48279439174004	2.70163864110249

8C_ae, Water

C	-5.43605813589013	1.21839628876526	-0.49694031253341
C	-4.59918373494564	0.42124049033219	-1.47629418898903
C	-4.66875468083812	1.53440262244908	0.77819648148723
C	-3.99654567332258	0.28543042163935	1.37538839358133
C	-3.19253787840470	-0.41704620602864	0.27975238100440
N	-4.05544849535606	-0.76545601599494	-0.84065555672114
H	-3.53837234442631	-1.30731602475212	-1.52051413075079
C	-5.51587403522154	2.31432823314822	1.77366213216873
H	-3.85474703038159	2.18961408678634	0.44640951868914
H	-3.28147291580679	0.64209112026431	2.12298705523814
C	-4.95692332605740	-0.67502436002343	2.07102054247503
H	-5.21342068223090	0.13303156135580	-2.32933641808862
H	-3.81187846073175	1.09990581100277	-1.84057243206233
F	-6.60285048725054	0.49843865308113	-0.19348795725280
H	-5.77011443074181	2.14452671547822	-0.96926814631155
H	-2.37359362311448	0.24815857561863	-0.03640225838402
H	-2.74568544843144	-1.33044252192927	0.67488410876832
H	-5.65773920016841	-1.11082501247154	1.36131856712942
H	-5.52888323250805	-0.17079750792362	2.85034061282377
H	-4.39647616838653	-1.48492005321355	2.54120282034215
H	-6.42239918873247	1.77101685674191	2.03845271204229
H	-5.81015225390394	3.27769378608102	1.35404374470473
H	-4.95283857314882	2.50059247959289	2.68991233063899

8C_aa, Gas Phase

C	-5.44631689890879	1.22270388840578	-0.49012031023383
C	-4.61495721745942	0.41415025992443	-1.47165264793292
C	-4.66732429963725	1.54359721262145	0.77956944349374
C	-3.99940479779406	0.29329521937829	1.38273051354056
C	-3.22395906027671	-0.44313030070007	0.28238682730623
N	-4.02217333575143	-0.77035894057216	-0.88673544384251
H	-4.74440612138769	-1.43990940835859	-0.65300828737714
C	-5.52002835964524	2.31771806413249	1.77428687755776
H	-3.85161810915263	2.19829555790128	0.44771812199117
H	-3.27380367303087	0.64796704269248	2.12261177381668
C	-4.96019538021897	-0.65484204198950	2.09496797964975
H	-5.22901011713914	0.13284276019590	-2.32715840176662
H	-3.80849938867840	1.05581952988101	-1.84285836395937
F	-6.58637892365865	0.48036221172660	-0.17467161446737
H	-5.79963621569949	2.14754837461053	-0.95558989986821
H	-2.39261067978072	0.18419457704309	-0.05714271052765
H	-2.78862375693139	-1.36154061578715	0.68003661530208
H	-5.69333363518112	-1.07661432106689	1.40827301307328
H	-5.51029879545086	-0.15146519875459	2.88903514493758
H	-4.40114368260507	-1.47598291267392	2.54655447945228
H	-6.43414217638149	1.77433600127835	2.00957225401589
H	-5.80383248004252	3.28903897812554	1.36672489059455
H	-4.97350289518807	2.48595406198567	2.70360974524405

8C_aa, Water

C	-5.43878161554749	1.23013811362014	-0.49391130868293
C	-4.61646672822124	0.41284135314405	-1.47254694247699
C	-4.66854726516255	1.54147665172901	0.78035028915083
C	-4.00163958004324	0.29064594062697	1.38196971143689
C	-3.22113654907795	-0.44133857573499	0.28421725982398
N	-4.02535509229107	-0.77754358407180	-0.88490380841159
H	-4.75868984386904	-1.42812193662659	-0.62683619840338
C	-5.51971696111360	2.31772369082626	1.77502502813838
H	-3.85272080700374	2.19618515304095	0.45118461899312
H	-3.27767833122495	0.64504653775861	2.12265681456743
C	-4.95957146024457	-0.66328186019478	2.09090173714507
H	-5.23475647424397	0.13345022437624	-2.32583308582459
H	-3.81051578030281	1.05347389448517	-1.84397906931714
F	-6.59948704834861	0.49606963798729	-0.18397239256941
H	-5.78235455412738	2.15488891539493	-0.96280931005813
H	-2.39723935310647	0.19333285061582	-0.05788243100214
H	-2.78204882940124	-1.35653033052443	0.68520176049206
H	-5.67685129073597	-1.10323591968677	1.39790804724905
H	-5.52420169867570	-0.15883618807611	2.87450682615423
H	-4.39428887092636	-1.47380657723111	2.55410920152700
H	-6.42603772957312	1.76972576143715	2.03108435782847
H	-5.81465546497628	3.28157491144691	1.35727905369119
H	-4.96245867178262	2.50010133565707	2.69541984054859

8C_ee, Gas Phase

C	-5.45866864169348	1.06562093882224	-0.48486948581549
C	-4.58572373472965	0.30461858627467	-1.48113315541525
C	-4.66168430948703	1.43144817637231	0.78310315500095
C	-4.01228282363095	0.16104191948548	1.32236140915405
C	-3.16627257865765	-0.54336727131905	0.27578939832594
N	-4.01864901868796	-0.87529085261980	-0.85083477420628
H	-3.52299175666717	-1.44408029019664	-1.52183503394108
H	-5.20131864412934	-0.01168273112776	-2.32493604174795
H	-3.81344839638849	0.98204971649421	-1.87899590456096
H	-2.32055513902338	0.10732792935028	0.00882148427537
H	-2.76215191274869	-1.45884675308589	0.70710787898730
F	-3.21254418768212	0.46024535947335	2.41673625521651
H	-4.78828471238065	-0.52640467577775	1.66774373711343
H	-5.37509178551804	1.77675294893119	1.53794384916267
C	-3.64364733977227	2.54699551844249	0.56242356740180
H	-6.25180707181796	0.37577329781145	-0.17962365554547
C	-6.10207182412842	2.28169093445995	-1.13765357690863
H	-2.98387973070996	2.34518264349589	-0.28155173540353
H	-3.02497763180305	2.67317539136207	1.44915787097341
H	-4.14930592351242	3.49209177757905	0.36884796164307
H	-5.35091609960437	2.94144629469410	-1.57482990665137
H	-6.67295837347105	2.86185242028249	-0.41071019588508
H	-6.78187836375587	1.97933872079569	-1.93510310117342

8C_ee, Water

C	-5.46011409783577	1.06520159014133	-0.48069115542824
C	-4.58933227071967	0.30386531034508	-1.47763047994382
C	-4.66118677168474	1.43312345539077	0.78622368854974
C	-4.02151400062022	0.15776389189493	1.31785195775643
C	-3.17088815962643	-0.54520300270925	0.27660386715087
N	-4.02128243568055	-0.88494169220480	-0.85357866639077
H	-3.49042413193298	-1.41379096419378	-1.53310853544136
H	-5.20272337254544	-0.00653470586332	-2.32493173131385
H	-3.81034277303226	0.97521043269992	-1.86837927295193
H	-2.33251011691672	0.10923537312283	-0.00041360380112
H	-2.75986641175367	-1.45807191201615	0.70723075419733
F	-3.21612419469712	0.44943170287819	2.42528293181441
H	-4.79864031384425	-0.52415470628510	1.66940859480003
H	-5.37481650014394	1.78136321268395	1.53913749121619
C	-3.63976819790618	2.54449263514573	0.55808453334601
H	-6.25672153465809	0.38119200317378	-0.17010361103583

C	-6.10265050074274	2.28097472013253	-1.13527069690054
H	-2.97667599145857	2.33194426185985	-0.28063643539377
H	-3.02513829313468	2.68610137090443	1.44612249753425
H	-4.14673009683555	3.48597677252277	0.35040526838243
H	-5.34999217562209	2.93556621511101	-1.57807927470150
H	-6.66846986868474	2.86451739255867	-0.40683569846017
H	-6.78519778992354	1.97371664270665	-1.92873242298479

8C_ea, Gas Phase

C	-5.47895455108928	1.07545202939813	-0.46695918896806
C	-4.62124397564974	0.28217347167776	-1.45692584204227
C	-4.66741337649571	1.44034703904001	0.79280975791955
C	-4.02265472700800	0.16972814685747	1.34021965385114
C	-3.20096030005962	-0.56276976875831	0.28979971124535
N	-3.98866807910003	-0.89378894990804	-0.88380982386443
H	-4.68646555079255	-1.59171685686107	-0.65623438774249
H	-5.22891566125817	-0.02469405318012	-2.31016300271986
H	-3.83112772008007	0.92605882052732	-1.85741698816117
H	-2.36350012566770	0.06957504824299	-0.01786473691288
H	-2.77800293332877	-1.47116575359062	0.71812707693152
F	-3.21405098673961	0.47314219162719	2.42738460033562
H	-4.80569658086088	-0.50231512963532	1.70742240515295
H	-5.36967154684525	1.79917235607020	1.55183050435813
C	-3.64074657509506	2.54509735978180	0.55643623956331
H	-6.28826695274961	0.40732075884062	-0.14813463520083
C	-6.10431305360875	2.29161570835814	-1.13596291576125
H	-2.99008051768559	2.33565001517574	-0.29308381967347
H	-3.01340540079983	2.66958533772319	1.43724113852339
H	-4.14019238256932	3.49388836289482	0.36511385418391
H	-5.33923347234332	2.94098209192312	-1.56468243159268
H	-6.68486522397980	2.88173038671351	-0.42506038560888
H	-6.76915030619334	1.98770138708143	-1.94554678381660

8C_ea, Water

C	-5.47433962221296	1.07403675254729	-0.46734857101967
C	-4.61521710876895	0.28872816890606	-1.46047511858039
C	-4.66411981574869	1.44106891355259	0.79296393202871
C	-4.02945612709062	0.16521021621166	1.33120371292953
C	-3.19788953164050	-0.56204192514673	0.28764254919958
N	-3.98864816038507	-0.89732679098137	-0.88744415140415
H	-4.70804714463675	-1.56539114003056	-0.63103618510240
H	-5.22637359114536	-0.01468048704076	-2.31218215940897
H	-3.82234529649786	0.93315370317192	-1.85415503105940
H	-2.36387364577265	0.07455660566368	-0.02092851880295
H	-2.77933491930348	-1.47113422077989	0.71926763332999
F	-3.21578167989408	0.46032620021883	2.43345665096776
H	-4.81076825633963	-0.50633128398387	1.69673857294458
H	-5.36994884193618	1.79841942814057	1.54903959720076
C	-3.63627771239559	2.54442264810717	0.55440818577475
H	-6.27819997234522	0.40068636663944	-0.14851521929474
C	-6.10685410243422	2.28800161678657	-1.13368382840201
H	-2.97746885200920	2.32493926411662	-0.28636262506365
H	-3.01771617103200	2.68639534064479	1.43962512676969
H	-4.13855700975933	3.48814570440183	0.34550613398996
H	-5.34604917074281	2.94124060665211	-1.56483132626081
H	-6.68748242190735	2.87296744298983	-0.41827310872042
H	-6.77283084600148	1.97737776921221	-1.94007625201575

9A_a, Gas Phase

C	-5.45512787027931	1.74095195247107	0.35706678056840
C	-5.15620419058618	0.82142409669314	-0.81399652655789
C	-4.18433055880674	2.35433880133483	0.93352908020464
C	-3.14573155628344	1.27936164157165	1.22393350274246
C	-4.45435103923639	3.23957996592982	2.13784482835228
H	-3.75952242334648	2.96863988384269	0.13042173274128
C	-2.88797496283584	0.40679736776603	0.00792761375217
N	-4.12571664628895	-0.14956776851917	-0.48989707698386
C	-4.35073709405174	-1.44772467504440	-0.79031464494911
O	-5.36873977490733	-1.87181059909165	-1.26880557025316
C	-3.19954978469821	-2.45703660019659	-0.52974071521990
F	-2.83893919945733	-2.49129385418475	0.75547880673064
F	-2.11541670238530	-2.13660285520320	-1.25256537319031
F	-3.57870160585890	-3.66742202756391	-0.88257917931353
H	-6.05416504145776	0.28740893731689	-1.10986546497504
H	-4.82393188615823	1.43717983902592	-1.65837764776797
F	-3.56066815074677	0.47832346669356	2.26736959493022
H	-2.20582449687302	1.74813281454362	1.52660408565382
H	-2.42353024598962	1.02258414338765	-0.77121944592636
H	-2.19073293720973	-0.37711343444831	0.27611938311893
H	-6.12163062746171	2.53779929264044	0.01425482144028
F	-6.13148786534694	1.03752486784881	1.33216774710245
H	-5.14410426956246	4.04311138186752	1.87816179274397
H	-3.52802660397386	3.69148321281170	2.49407160591508
H	-4.88948446619772	2.65934014850664	2.94854026914051

9A_a, Chloroform

C	-5.44825688954170	1.74034232625536	0.33825421917575
C	-5.14195985050280	0.84395746838995	-0.84620314952211
C	-4.18641640563803	2.34572715068341	0.93586614013699
C	-3.13514147870724	1.27869320719076	1.19808974099230
C	-4.46482028979119	3.20521147462850	2.15747773620749
H	-3.76664919760140	2.98243239271900	0.14920848822754
C	-2.87474845484509	0.42818736523378	-0.03081169882180
N	-4.11607234601109	-0.13757808794638	-0.52204757457449
C	-4.35679885906307	-1.43308142132212	-0.75997118032549
O	-5.39492161902348	-1.87956274194538	-1.19323634784245
C	-3.21056227081339	-2.44591554511654	-0.48800105223588
F	-2.83762999382833	-2.45344035582344	0.79393112956825
F	-2.13250366028369	-2.17145307431017	-1.23160428991132
F	-3.61367454828964	-3.66538619011394	-0.79972092319319
H	-6.04014067152824	0.32677051382697	-1.16688610451248
H	-4.78622134146332	1.47181481118636	-1.66866746577884
F	-3.54373400249061	0.44412511445562	2.23213601059573
H	-2.20011878072523	1.74567430745034	1.51065014301525
H	-2.43897967911816	1.06253975409692	-0.80802778383541
H	-2.15839115440988	-0.34590384401895	0.21357604789037
H	-6.12225686362572	2.53552456384904	0.01361572440818
F	-6.12778826526324	1.00047792756283	1.30028409247411
H	-5.15978468449952	4.00743624599384	1.90792485090707
H	-3.54060817937395	3.65695013161284	2.51906542959689
H	-4.89645051356195	2.61186650546140	2.96122781735754

9A_e, Gas Phase

C	-5.48697840765020	1.14233212479594	0.37672191333676
C	-5.08370817267993	0.57704390054743	-0.97896333702117
C	-4.30760358021308	1.77010140865258	1.11888191796802
C	-3.18438426697832	0.73630844687002	1.20859779984421
C	-2.82649057401570	0.14442973311034	-0.14965251287955
N	-4.02513898155249	-0.39132506585993	-0.77685398574753
C	-4.05658907133988	-1.70241404300478	-1.10777411091039
O	-3.15872074457921	-2.47795918303391	-0.91419420327073
C	-5.34359197923271	-2.24149915299540	-1.78956518325578
F	-6.41503506986186	-2.08207187982948	-1.00113334904849
F	-5.21104626467642	-3.52582639849319	-2.04342499408212
F	-5.58546411908515	-1.61177362297372	-2.94470707418260
H	-5.94664570456116	0.12779795877469	-1.45495584222258
H	-4.72339101963851	1.38014094881449	-1.62638386912629
H	-2.39388999314334	0.92013464329145	-0.78623539453525
H	-2.10045916384320	-0.65416180115269	-0.03452513220394
H	-3.46936060444655	-0.07449482111335	1.88416053105621
F	-2.05307848129560	1.32788269288658	1.73423084416377
H	-4.63897005356266	1.99246947295524	2.13590815123876
C	-3.84302042825114	3.06907678414192	0.47093663626630
F	-6.47119005696665	2.08839110339115	0.17841488849368
H	-5.92219543890370	0.33474631940358	0.97250096665472
H	-3.43679292794185	2.91695994070769	-0.52882605317763
H	-3.06483714760899	3.52706014366912	1.07706562328761
H	-4.67682774797172	3.76327034644423	0.39231576935401

9A_e, Chloroform

C	-5.48251928987853	1.13832033811716	0.38580012955400
C	-5.08552373894697	0.57291924728380	-0.97127291363372
C	-4.30550573658974	1.77654341801861	1.12041337251264
C	-3.18997112509796	0.73681771518718	1.21187083493644
C	-2.82806975137055	0.14271787691369	-0.14386563710139
N	-4.02782275604698	-0.40023802737642	-0.76779242320203

C	-4.06026305435480	-1.69788463564478	-1.10839564381474
O	-3.16083471537425	-2.48515230497698	-0.92630937057405
C	-5.34456815834385	-2.23563827778977	-1.79757335758250
F	-6.42105468648834	-2.09207953228208	-1.01757131624226
F	-5.20221592558960	-3.52277916694310	-2.05798240257561
F	-5.58281069988256	-1.60805872201961	-2.95271711033610
H	-5.95139171341149	0.12727997978948	-1.44504643928145
H	-4.71632941924513	1.36936969331707	-1.62049802247202
H	-2.40640794305108	0.91602404108828	-0.78943090848446
H	-2.09646524404705	-0.64903759394469	-0.02270513136135
H	-3.47560623689487	-0.06692240800102	1.89309571644373
F	-2.04881725503481	1.32719069480759	1.73561989819770
H	-4.63550621285757	2.00077381149951	2.13707080085783
C	-3.84042577428118	3.07070999378721	0.46292891810547
F	-6.47487731939869	2.08415539593629	0.18300517989753
H	-5.91542352128714	0.33728556675271	0.98948149020012
H	-3.43562628469583	2.91285057069126	-0.53653578792552
H	-3.06424557223143	3.53476257722594	1.06777604075382
H	-4.67312786559960	3.76668974856268	0.38317408312794

9B_a, Gas Phase

C	-5.45193866615970	1.36399006016270	-0.39389279031403
C	-4.62052417341690	0.60236747888402	-1.40929972729781
C	-4.65421734169565	1.72471275122165	0.85620157197702
C	-3.96027731788801	0.49434211303645	1.43374716093902
C	-3.15111985488633	-0.25503315275494	0.39170132874539
N	-4.07662524971798	-0.61578673493062	-0.72892100867075
C	-5.50457620776775	2.44335304785815	1.89294582024188
H	-3.85676731798076	2.40171032809504	0.52717350796554
H	-3.60966414287655	-1.22503500854274	-1.39998005871272
H	-4.86809419375394	-1.13815295028105	-0.33190835419805
H	-5.23168686252584	0.28122876808563	-2.24864692553573
F	-4.89427603564828	-0.40463739622564	1.91022469215948
H	-3.30873729285754	0.78000701637416	2.26099619449334
H	-3.77805989706306	1.19290733766211	-1.76575735102924
H	-5.85560109026851	2.26472704046198	-0.85875190757179
F	-6.50828763979698	0.53571075657287	-0.06856832383990
H	-2.35142868660286	0.36030904259785	-0.01744299912358
H	-2.74040214295434	-1.17232443310605	0.80529014948145
H	-6.31008922057616	1.80524274542524	2.24896913877272
H	-5.94088958300999	3.34664684054817	1.46758775234392
H	-4.89490708255286	2.73946434885503	2.74614212917385

9B_a, Water

C	-5.45825946458241	1.36240484292247	-0.38842028197239
C	-4.63967522346081	0.58824461931467	-1.39535728105083
C	-4.66213946872031	1.71613785509336	0.85913788668333
C	-3.96798908670128	0.49220607522695	1.43832967037409
C	-3.17393756454329	-0.26615961353654	0.40015501546430
N	-4.05390972728815	-0.61538373615773	-0.74692440270432
C	-5.49767980634613	2.45414758391714	1.89239640639998
H	-3.85964951985140	2.37918827649782	0.52019921464995
H	-3.52344786631043	-1.14943288977618	-1.43514188029097
H	-4.80504775809712	-1.23168878389950	-0.42647388615452
H	-5.25239398405402	0.25740376659892	-2.22817131619279
F	-4.91282873449625	-0.38539593275092	1.97005304141107
H	-3.30151406340152	0.78521001574899	2.24911610270753
H	-3.81680332325569	1.19779551801459	-1.76337409462385
H	-5.83793318914559	2.26700007916283	-0.86253986781150
F	-6.55826407550435	0.57590277251560	-0.04615386695422
H	-2.36407958605536	0.35187698659597	0.01820857713734
H	-2.77132325195565	-1.18763647594884	0.80899731354984
H	-6.30907873090647	1.82921635593110	2.26070080261427
H	-5.92608090442940	3.35599583308484	1.45503730854360
H	-4.87613467089435	2.74871685144446	2.73803553822011

9B_e, Gas Phase

C	-5.54252852205404	1.27824839638951	-0.50272262171501
C	-4.63698524200036	0.49781271005697	-1.45163360192472
C	-4.83958873223821	1.67236120729485	0.79752204204872
C	-4.25345972854503	0.41381597462306	1.43983484660123
C	-3.34750588928065	-0.36681902436826	0.49142326734990
N	-4.11682385517544	-0.71817431310383	-0.74616432277506
H	-3.52122013158488	-1.25379417604100	-1.37964763898764
H	-4.89805224756318	-1.33313508305734	-0.50148551845706
H	-5.17976747081585	0.17961778054128	-2.33825015803623
H	-3.77722024058348	1.09660654136894	-1.74633766300468
H	-2.49556057192717	0.23725583785983	0.18500815497818
H	-2.99468611596810	-1.28538951849218	0.95370990435501
H	-5.60802125689544	2.04996061077141	1.47536250140545
C	-3.80048610037288	2.76719937296938	0.59483577913362
F	-5.94686256790603	2.38920314812369	-1.18550667198824
H	-6.43581986256529	0.68161996714459	-0.28402573790411
H	-5.05882182961532	-0.24161721755984	1.79128255124494
F	-3.48670502756039	0.73914132921741	2.52158799766468
H	-2.98830113387317	2.47677211364072	-0.07308732308217
H	-3.36172937409121	3.03920657955819	1.55195209748588
H	-4.27501409938386	3.65110776306262	0.17521211560730

9B_e, Water

C	-5.54067222218610	1.26890842875274	-0.49359676418266
C	-4.63602659621625	0.49895789989335	-1.44228471625350
C	-4.83951787050552	1.67747951486300	0.79979301071082
C	-4.26357639516222	0.41222557738968	1.43108442070758
C	-3.35382442983604	-0.35981297140173	0.48916008670140
N	-4.11017074689123	-0.70345515862552	-0.74371101207665
H	-3.50040874519692	-1.22034757403962	-1.37845149851860
H	-4.87611302781672	-1.34308680995202	-0.51941078223305
H	-5.18410108249329	0.16780017933066	-2.31896824086612
H	-3.78141798275097	1.09785657149950	-1.74893644313315
H	-2.49517520964378	0.23672343712814	0.18924611458917
H	-3.01555996508991	-1.28443978016644	0.94651160904616
H	-5.60613797407931	2.05786283237325	1.47758885592871
C	-3.79135004082546	2.76181414470753	0.58659277616784
F	-5.97157038550967	2.39455261904475	-1.17202798591892
H	-6.42504875952657	0.66923632948569	-0.26833204806362
H	-5.07153135721260	-0.23915797232878	1.77113054122115
F	-3.50830497070545	0.74077409825194	2.54227777302343
H	-2.98959111069413	2.45227295048067	-0.08383794254304
H	-3.34564532022571	3.03606475262027	1.54063910306664
H	-4.25941580743212	3.64877093069294	0.16440314262642

9C_ae, Gas Phase

C	-5.46173496677802	1.35316368217072	-0.39609754814270
C	-4.61450930920961	0.56904482944749	-1.38420668127221
C	-4.66569408142099	1.70450269572243	0.85650489269216
C	-3.96568615519059	0.48012601141352	1.43707483293180
C	-3.18377380338321	-0.26443230477721	0.36799105951465
N	-4.04362561036881	-0.59381460073591	-0.74503529155596
C	-5.49900055539979	2.44520767454281	1.88885040523505
H	-3.86017014402523	2.36518558698550	0.51382522339907
H	-3.58403858677454	-1.19881881231732	-1.40815486030756
H	-5.24185875951191	0.25325862015016	-2.21706383362814
F	-4.88604868792165	-0.36654967188669	2.02353806785479
H	-3.28088829366254	0.79925249092604	2.22856229578502
H	-3.85279420253679	1.26591545472111	-1.77656738576726
H	-5.80933300633035	2.27539509211405	-0.87141821088690
F	-6.58865623010278	0.62806233780528	-0.06113726260391
H	-2.33446855735807	0.38175859000641	0.08358831152045
H	-2.77987096969771	-1.18064991735860	0.79734058465386
H	-6.30828550899941	1.81430282276176	2.24996121342339
H	-5.93190419111459	3.34841802519850	1.45683501938996
H	-4.88382838021340	2.73887139310994	2.74041916776445

9C_ae, Water

C	-5.45566364804336	1.35549301759801	-0.39685012155847
C	-4.61709218933014	0.56516767787212	-1.38073091831638
C	-4.66877296136213	1.70277838307930	0.85833708174124
C	-3.96237068320668	0.48373950742320	1.43310839709295
C	-3.18927523397965	-0.26701565473034	0.36804723709014
N	-4.06098174616400	-0.61367961148795	-0.74045578188112
C	-5.49763550368133	2.45008980965800	1.89006872807773
H	-3.86412954689393	2.36267303857697	0.51611647851473
H	-3.55555682702822	-1.16905906128537	-1.41760280747854
H	-5.23703137601195	0.26273317913527	-2.22407518182320
F	-4.89700691015383	-0.37695480131331	2.01998894882670

H	-3.28629290908570	0.79323032938428	2.23206831217572
H	-3.84081709315487	1.24853652134604	-1.75800770710179
H	-5.81609033537080	2.27056909872903	-0.86999433019292
F	-6.59304888356597	0.61417749009427	-0.05671673075565
H	-2.35411089900383	0.38229402855676	0.06356062591091
H	-2.76903247119770	-1.17535644025811	0.79847479748080
H	-6.31160088917826	1.83038979687047	2.26237820146962
H	-5.92469917245549	3.35290946830459	1.45161658785843
H	-4.87496072113216	2.74548422244678	2.73547818286912

9C_aa, Gas Phase

C	-5.45991080824701	1.37163851904429	-0.38993723137525
C	-4.63861920354830	0.54487574558879	-1.36605211043813
C	-4.66029013093719	1.72291433474418	0.86087194103240
C	-3.96309235388882	0.49791025664582	1.44436273607657
C	-3.21612732141307	-0.28325296936732	0.37557306645274
N	-4.04597319617019	-0.63098066544453	-0.76056217241643
C	-5.50097971235305	2.45342724514290	1.89451558477922
H	-3.85589574038379	2.38639909674133	0.52054511256225
H	-4.77641296128030	-1.26866325826346	-0.46707383450189
H	-5.26562762771121	0.24535244145310	-2.20553100587421
F	-4.90137921115285	-0.34862149395307	2.02141807601847
H	-3.28317249597793	0.80711751174293	2.24362615023461
H	-3.83560109413112	1.17470926988883	-1.76403808699908
H	-5.81882547830991	2.28847225150802	-0.86674767441972
F	-6.58477652930860	0.63564520244209	-0.03935151873141
H	-2.37776255155304	0.32660720994850	0.02187135998348
H	-2.79907801077817	-1.19001274555830	0.81307432350978
H	-6.30586559959736	1.81177337472005	2.24677471558991
H	-5.94076292308040	3.35483895562882	1.46637390161375
H	-4.89187705017770	2.74448971734703	2.75099666690293

9C_aa, Water

C	-5.45495662567927	1.37270583430357	-0.39186379058665
C	-4.63905951069792	0.54590015429152	-1.36830903701458
C	-4.66322058941673	1.71876664549111	0.86130648469292
C	-3.95941596111528	0.49972435452298	1.44053843583340
C	-3.21366349536907	-0.28380698546839	0.37648943589382
N	-4.04383592518508	-0.63508451261926	-0.76421774914255
C	-5.50115220515529	2.45348654620215	1.89474110078727
H	-3.86041086127192	2.38309182217352	0.52249306522592
H	-4.77687635648682	-1.26926444103823	-0.46675897389353
H	-5.26692795899927	0.25001829825987	-2.20844625974008
F	-4.90497833849659	-0.35676226632414	2.02470828857791
H	-3.28287549625656	0.80479744655121	2.24113595356072
H	-3.83852092063399	1.18077577690193	-1.76012738116305
H	-5.81705889052884	2.28577894645326	-0.86819726519172
F	-6.59281985098137	0.63055670781852	-0.04030151512773
H	-2.37903354115846	0.33155229912106	0.02705603836921
H	-2.79530204138172	-1.18851373636321	0.81698387189335
H	-6.31018930355848	1.82062927594996	2.25561627431804
H	-5.93584623885905	3.35486779461358	1.46137093801113
H	-4.88588588876830	2.74542003915900	2.74649208469619

9C_ee, Gas Phase

C	-5.52332791480280	1.26934631957569	-0.51496442156869
C	-4.59748514051885	0.49066227228386	-1.43541053806306
C	-4.84037094348412	1.66522075700586	0.79486215559367
C	-4.23828383314447	0.40716970734068	1.42213316977249
C	-3.34462524866526	-0.34870661667607	0.45225189838261
N	-4.13635673115215	-0.68347352562863	-0.71735311646826
H	-3.64236485105584	-1.31731343771473	-1.32715250439531
H	-5.15640481148501	0.17949966262351	-2.31725431594342
H	-3.78316341752760	1.15015449137762	-1.76630157332046
H	-2.46304233297660	0.26618612917024	0.22353539117036
H	-2.99978300423352	-1.26480861063801	0.93046336984232
H	-5.61083114128697	2.04071709080105	1.47321818062729
C	-3.80380623977084	2.76471894934820	0.59593423108471
F	-5.96013752548826	2.40843688509611	-1.17032603812917
H	-6.40411343927101	0.65764446273534	-0.30624918971093
H	-5.03536371169774	-0.26099051195853	1.75639218290110
F	-3.49991436165690	0.75665450178611	2.54034660896270
H	-3.00055869865019	2.46176789868168	-0.07455681001784
H	-3.35846805455739	3.03159591738100	1.55197482376599
H	-4.27725859857447	3.64788765740902	0.17239649551389

9C_ee, Water

C	-5.52479734481900	1.26213656518417	-0.50827854216136
C	-4.60075582915292	0.48937447850431	-1.43251539671592
C	-4.84108192880494	1.66790571284037	0.79664579197392
C	-4.24703438799590	0.40466737604261	1.41801427631678
C	-3.34881564452709	-0.34918658746450	0.45361026806661
N	-4.13922174085119	-0.69116041949621	-0.71875157477878
H	-3.60867594625317	-1.28941405217190	-1.33693710372822
H	-5.15676666625130	0.18018044079771	-2.31678011240377
H	-3.78036587473493	1.14340992748177	-1.75634612225724
H	-2.47234034787233	0.26787637671681	0.21537382706795
H	-2.99964137693326	-1.26389723818286	0.93109187544666
H	-5.61068158040414	2.04462460160458	1.47484970406890
C	-3.79849044215709	2.76119048919746	0.59076154503549
F	-5.96950843903773	2.40766672468022	-1.16923146314181
H	-6.40836403916505	0.65771233459997	-0.29592493907731
H	-5.04592268255654	-0.25700853286806	1.75725151399343
F	-3.50595105382927	0.75256077260563	2.54788038541998
H	-2.99863396270869	2.45206975294563	-0.08100998316131
H	-3.35085099161185	3.03337815018958	1.54482642608901
H	-4.26775972033358	3.64828312679270	0.16940962394697

9C_ea, Gas Phase

C	-5.54461327113793	1.28089759325348	-0.49758046316897
C	-4.63959274287525	0.47071069512740	-1.41534002392839
C	-4.84300089330051	1.67797535141267	0.80233296723761
C	-4.25789515900911	0.41765773490477	1.44175689732840
C	-3.38761421911023	-0.36839814150126	0.47096803778227
N	-4.10175157527382	-0.70107425412396	-0.74832241426948
H	-4.82474890460117	-1.38733015840742	-0.57361939568064
H	-5.19018201144035	0.16567150772378	-2.30460438761245
H	-3.80889725757959	1.09689294180105	-1.75008721394768
H	-2.50710306035219	0.22473221668459	0.21178442775817
H	-3.03099969949701	-1.28097473893108	0.94745598240963
H	-5.60135026340458	2.07483925218295	1.48221716913680
C	-3.79040293324324	2.75840150004691	0.58416095792383
F	-5.97826593363230	2.41953051036120	-1.15700921107521
H	-6.43652795127381	0.68782979412395	-0.26968242709713
H	-5.06893177627638	-0.22986836531894	1.79112210450474
F	-3.51468388610234	0.76572257827044	2.55790954712606
H	-2.99501959272689	2.43785000370209	-0.08785296153071
H	-3.33614360307089	3.02898402162050	1.53499214844915
H	-4.25399526609241	3.64409995706690	0.15491825865400

9C_ea, Water

C	-5.54175761181992	1.27098621945375	-0.49392603264046
C	-4.63496523622148	0.47335414035751	-1.41788646478819
C	-4.84249653358431	1.67800661521008	0.80210705265774
C	-4.26328901835206	0.41299319699447	1.43331907735612
C	-3.38235464840786	-0.36580247018952	0.46928362236847
N	-4.09927137325750	-0.70415226486649	-0.75117346296414
H	-4.84336978044420	-1.36290180737630	-0.55012417058407
H	-5.19027186515300	0.16733409620149	-2.30375699606223
H	-3.80334563502619	1.10094002613913	-1.74808032478541
H	-2.50508293215961	0.23176144246067	0.20889354581667
H	-3.03034817479656	-1.27867144236944	0.94835549340395
H	-5.60317475267734	2.07058182391091	1.48144218056415
C	-3.78809393187567	2.75687032271100	0.58202151246136
F	-5.98498885085213	2.41685681239766	-1.15846841491362
H	-6.43106884565739	0.67802396802728	-0.26765330040028
H	-5.07191790303408	-0.23455078919378	1.78046036693201
F	-3.51823389048674	0.75974447877925	2.56282906592720
H	-2.99367604581425	2.43500474338656	-0.09071056734937
H	-3.33405448445299	3.03193454305646	1.53229706011822
H	-4.24995848592671	3.64583634490931	0.15629075688186

10A_a, Gas Phase

C	-5.49324781692372	1.37914126383482	-0.08839416538179
C	-4.86764354502281	0.58978391110263	-1.23178586525016
C	-4.47014465034097	1.67274209456630	0.99200170373785
C	-3.78010194448774	0.40161102447590	1.44944776406432
C	-3.17445706803361	-0.34196695090718	0.26534482225528
N	-4.19509751704161	-0.60468528975683	-0.73376135457524
C	-4.47919158967193	-1.79236111774168	-1.30179073660231
O	-5.30026541454156	-1.96533799923491	-2.16655368226643
C	-3.69078146713869	-3.03471240983643	-0.80490459391536
F	-3.87929440346100	-3.24770715733378	0.50494000460950
F	-2.37534889266884	-2.89478862633680	-1.01162066739861
F	-4.09063844391347	-4.10982768278264	-1.45184965164969
H	-5.61683704502710	0.28294385212633	-1.95422035598426
H	-4.12984878400145	1.21437830945899	-1.74281639541247
H	-2.39373751023898	0.27233631394672	-0.19101299183528
H	-2.71834766789841	-1.26582592310925	0.59785483037343
H	-4.51500506763390	-0.23782239511382	1.94407275000619
H	-3.00031548239534	0.64435815379012	2.17068081432576
H	-4.92824116096856	2.19323834859040	1.83408232988748
F	-3.50569330909610	2.52935002118523	0.46542528348970
H	-5.90271805546355	2.31570673185388	-0.46818371922338
H	-6.31424316403066	0.80932552722198	0.35223387674547

10A_a, Chloroform

C	-5.49062641428588	1.37715656409479	-0.09253581917011
C	-4.86005985175973	0.59661568613852	-1.23880345530115
C	-4.48096530632912	1.66386707418063	0.99955107674473
C	-3.77707968858676	0.39771116968462	1.44281201811515
C	-3.16715358635722	-0.33693710182003	0.25611779075470
N	-4.18856007070410	-0.60433253292359	-0.74617303341121
C	-4.48042280297929	-1.78740484817726	-1.29155550154606
O	-5.31160653258012	-1.97650183304105	-2.15496091450976
C	-3.69478249368019	-3.03082301704828	-0.79181839615786
F	-3.87450507755677	-3.24336438230576	0.51663315738538
F	-2.38183350269055	-2.90804548842395	-1.01381816176369
F	-4.11034306899136	-4.10931195261312	-1.43420066128129
H	-5.60886888917853	0.30306389694814	-1.96602048473708
H	-4.11370353460956	1.21648876710550	-1.74155096754393
H	-2.39521762388284	0.28125527537708	-0.20773089354528
H	-2.70149669639135	-1.25615949460087	0.58755586065465
H	-4.50766129963440	-0.24665826084340	1.93683093286574
H	-2.99643070591792	0.63924374708575	2.16325342153374
H	-4.94934036404109	2.16778819691419	1.84436738893799
F	-3.51781433345727	2.54773607805961	0.49124826887635
H	-5.89993193281500	2.31414904480883	-0.47109113170292
H	-6.31279622357095	0.80434341139962	0.34107950480191

10A_e, Gas Phase

C	-5.44409327954467	1.31117801682576	-0.25151520409335
C	-4.62930741163746	0.67313520164881	-1.37341781966527
C	-4.55985050085291	1.58697764834259	0.94989921269704
C	-3.83180903502384	0.33493329391906	1.39928665692377
C	-3.07707458394048	-0.29321182867732	0.22953961769369
N	-3.97704431532209	-0.52831050702457	-0.89041472260168
C	-4.24846781573438	-1.79779506168250	-1.25342171950999
O	-3.75136097699828	-2.77659744630410	-0.75848927170510
C	-5.27410980487642	-2.01216158463061	-2.39953405733500
F	-4.87834328148330	-1.42189543847088	-3.53371424035044
F	-6.47532872511279	-1.51302890199180	-2.07581402514396
F	-5.41998678287000	-3.29855091619680	-2.63860829736313
H	-5.25681993513439	0.45453996451827	-2.22861215950160
H	-3.84868395471541	1.36135031526879	-1.71243576482505
H	-2.28089109320828	0.37647072084879	-0.10981077068518
H	-2.62765175458405	-1.23860327269143	0.51575641369185
H	-4.56321589887873	-0.37737881526122	1.78678007641783
H	-3.13721604491503	0.57573175747165	2.20481787225531
H	-5.90107720554979	2.23693300496227	-0.60026705219469
H	-6.24125675655449	0.62707114015417	0.04775573815299
F	-5.33479150706211	2.06957643708203	1.98975084711374
H	-3.83446933600111	2.37000627188903	0.70317867002822

10A_e, Chloroform

C	-5.44648249902060	1.31937736319281	-0.23774685592645
C	-4.65011828902283	0.66168691015183	-1.36044294483055
C	-4.54403264930471	1.60100710044885	0.94602168942063
C	-3.83036527813336	0.34785652994709	1.40877511701212
C	-3.09313085325191	-0.30345107486611	0.24154178702259
N	-4.00907688151593	-0.54675080687418	-0.86810329464046
C	-4.24977961264126	-1.80021300396568	-1.26235500469553
O	-3.73672871616289	-2.79309334354241	-0.79217307140855
C	-5.26143505158091	-2.01269216518687	-2.42169010445070
F	-4.86374400075939	-1.40751425297615	-3.54546131759878
F	-6.47623833160913	-1.54741425269731	-2.10954082753950
F	-5.37974433331604	-3.30371097736468	-2.68014172214610
H	-5.29094823725919	0.44623171137945	-2.20595005527138
H	-3.86179516080315	1.33342844160220	-1.70998240782028
H	-2.29940329197791	0.35617789721170	-0.11876480174019
H	-2.64551922596775	-1.24437024372252	0.54181335209747
H	-4.56701780898532	-0.34994144007215	1.81271377153046
H	-3.11887098014295	0.59017313089904	2.19859665006903
H	-5.89293317286930	2.24460892666019	-0.60026672936773
H	-6.24689214943961	0.64704036307661	0.07975034016375
F	-5.31132117446881	2.10552526292593	1.99535223314259
H	-3.81727230176704	2.37640792377236	0.68876419697758

10B_a, Gas Phase

C	-5.48694681226254	1.04611901329964	-0.46823400731261
C	-4.61842752956454	0.34619557496348	-1.49580277597288
C	-4.67760064239436	1.38498272587294	0.77335018248207
C	-4.04570394703100	0.13435472699748	1.36323207571419
C	-3.18143653940865	-0.56422356779289	0.33117677294260
N	-3.99882358657227	-0.88476851792486	-0.89105052660585
H	-3.41013924238952	-1.34753699008932	-1.58453701046344
H	-4.73601464869150	-1.54794555945120	-0.64161842497009
H	-5.17964709183032	0.03749355483508	-2.37361477472170
H	-3.79127005933020	0.98473828195174	-1.80153432710673
H	-2.37629109914043	0.08781741438084	-0.00342346566255
H	-2.76141614372492	-1.49516074721726	0.70235874530631
H	-6.34120797311861	0.42413617044696	-0.18868588176848
H	-5.88389637662015	1.95738958809213	-0.91385227376587
H	-5.29322084288916	1.89629030414968	1.51250979109934
F	-3.65858832115091	2.24222850907727	0.39792241811815
H	-3.42073398262559	0.39951400665420	2.21510783798533
H	-4.83008516125531	-0.53134448824593	1.73245564470220

10B_a, Water

C	-5.46993838076889	1.05475410329419	-0.47486353274457
C	-4.60518238038167	0.33699041942170	-1.49264884415547
C	-4.69252265422172	1.38288657956224	0.78384425053983
C	-4.03047641353465	0.14460703530980	1.35404668709896
C	-3.18375993686382	-0.56436822808804	0.31493814833383
N	-4.00882490064557	-0.88679125318083	-0.88443565358994
H	-3.43225921992717	-1.36486767707527	-1.57609556443772
H	-4.74951133110021	-1.54229352048221	-0.62936324948722
H	-5.17676549248290	0.02761499268156	-2.36179280378533
H	-3.77047721248965	0.95689020931523	-1.81330474137442
H	-2.37117885180011	0.06879628767592	-0.03458617055702
H	-2.77554634607378	-1.49584127502100	0.69393913288314
H	-6.32223321833564	0.42987323202927	-0.19905005813788
H	-5.86135547148395	1.96687770149992	-0.92277624572347
H	-5.33479616593951	1.85588804171396	1.52476442926064
F	-3.69382285006065	2.31078019728249	0.45921811711486
H	-3.40081673618328	0.41193069062776	2.20138647686051
H	-4.81198243770681	-0.52344753656670	1.72253962190127

10B_e, Gas Phase

C	-5.43635797871924	1.07447001860310	-0.49648451191756
C	-4.58966732709615	0.33590558805703	-1.51519691956601
C	-4.64164461981149	1.40958652399877	0.75732012559890
C	-3.99301906689031	0.16124410350274	1.33771676761398
C	-3.15961852130775	-0.56992993657221	0.30286611329347
N	-4.01102042090974	-0.90291428456373	-0.89046183677226

H	-3.46152811195208	-1.41810495138013	-1.57946485849184
H	-4.77309812498355	-1.51959749685593	-0.59822814938776
H	-5.16751793293885	0.03239860093717	-2.38432761889614
H	-3.73904764055631	0.93034587442222	-1.84852464788534
H	-2.34206941784252	0.04514318111785	-0.07298558267650
H	-2.75005307707145	-1.49925388403358	0.69019017848350
H	-6.30153732324707	0.47031921996315	-0.20958430984184
H	-5.81969045176891	1.98558208069074	-0.95460746865111
F	-5.49227324959520	1.93806982813914	1.68997637628150
H	-3.88034102505944	2.16388196692198	0.53354945345169
H	-3.35312758904350	0.42519129825379	2.17905279200879
H	-4.77982812120644	-0.49205773120209	1.72492409735453

10B_e, Water

C	-5.43974844910443	1.07009878750032	-0.48810981631350
C	-4.59028198550318	0.33137149371141	-1.50517249412380
C	-4.62972653243041	1.40745842490286	0.74712739535050
C	-4.00354771761945	0.16051699162755	1.33782376345086
C	-3.17002695666764	-0.56775564023892	0.30021094904807
N	-4.00632852917640	-0.89380031170077	-0.88942890529615
H	-3.44207398734919	-1.38839465187165	-1.58022217519300
H	-4.75380720961328	-1.53635936223897	-0.62091565416050
H	-5.17403280705049	0.02194533869413	-2.36616006146634
H	-3.74890186506684	0.93423436977498	-1.84293843259901
H	-2.34938630947623	0.04854090594520	-0.06304230350193
H	-2.77063409398739	-1.49922464175875	0.68834772287947
H	-6.28988921704603	0.45020231833368	-0.19336896647431
H	-5.82589168350676	1.97707840411980	-0.95091806086012
F	-5.47262261434153	1.98173033762762	1.69584646806365
H	-3.86312779895149	2.14948572790409	0.51363824656181
H	-3.36286438873716	0.41736455946132	2.18020268899844
H	-4.79854785437209	-0.49421305179389	1.70280963563586

10C_ae, Gas Phase

C	-5.46099156021046	1.05225873520659	-0.48567656608927
C	-4.57254191525516	0.32157614348375	-1.48441379831153
C	-4.68974819124585	1.37835300231614	0.77953585317141
C	-4.01963755671917	0.14043743689811	1.34577715648890
C	-3.18576402375922	-0.55700612782778	0.27855339620797
N	-4.02565983484216	-0.87634850415136	-0.86574850084479
H	-3.50464765275352	-1.41473354519410	-1.54394407432095
H	-5.15935539376546	0.03121585143154	-2.35568332343999
H	-3.78573601311101	1.01101560714093	-1.82323624858298
H	-2.34481760990227	0.09769151438574	0.00762292803246
H	-2.77227102424869	-1.48176254897610	0.68061686546678
H	-6.30478898442636	0.41278147734744	-0.21619657401760
H	-5.85449971876607	1.97168564217535	-0.92034754477094
H	-5.33787553979471	1.84652499812192	1.52278826188351
F	-3.70335610977906	2.31558115781981	0.46923693741797
H	-3.39942175917547	0.41865597582824	2.19847790380695
H	-4.79940711224534	-0.53918581600621	1.69738732790209

10C_ae, Water

C	-5.45916253095438	1.05465390462445	-0.48698284031128
C	-4.57487253668638	0.31621436226966	-1.48331948782825
C	-4.69898013957432	1.37315797348761	0.78421713675398
C	-4.01686670013578	0.14227908529428	1.34558681381210
C	-3.18972128151131	-0.56143243819473	0.27754030875318
N	-4.03188745046947	-0.88867331594571	-0.86702210198790
H	-3.48831205515411	-1.40100630605579	-1.55011713463001
H	-5.16011512205228	0.03343197991677	-2.35777955069549
H	-3.77710723760918	0.99288933666057	-1.82137262912448
H	-2.35103296320837	0.08885541702749	-0.00944389718651
H	-2.76995871686620	-1.48166495439682	0.68270759686342
H	-6.31171400794184	0.42658932145988	-0.21720318043856
H	-5.84677714224748	1.97549541145947	-0.92420311710676
H	-5.34783336448382	1.83811193929996	1.52635689862816
F	-3.70923803272308	2.32955493379484	0.48067047908991
H	-3.39191034648155	0.42266407906520	2.19425389579749
H	-4.79503037190042	-0.53238072976711	1.71086080961099

10C_aa, Gas Phase

C	-5.47603680053546	1.06539693845350	-0.47330073054945
C	-4.60263833031395	0.29912039107611	-1.46717662642346
C	-4.69165960589900	1.38870708650821	0.78627127701636
C	-4.02967397705795	0.15028535789599	1.36462701298587
C	-3.21981532964451	-0.57675723194784	0.29072269669023
N	-3.99268334156981	-0.89245886918332	-0.89964870541076
H	-4.70352485861848	-1.58156491979424	-0.68382698733413
H	-5.17897928503805	0.01962717068705	-2.34894380779159
H	-3.79525592904086	0.95262492012768	-1.80764173154704
H	-2.38589460419595	0.05958500722254	-0.01672042328659
H	-2.78916869089668	-1.49467881140697	0.69045552635250
H	-6.33785010373320	0.45208684785989	-0.19391470060034
H	-5.85524859236117	1.98916728656071	-0.91329410003915
H	-5.32737200044774	1.87459320265471	1.52858566100612
F	-3.69415911573242	2.30778331418131	0.45838073061377
H	-3.39846356384466	0.43476341082989	2.20808481777760
H	-4.81289587107011	-0.51246110172522	1.74444009054006

10C_aa, Water

C	-5.47064389788886	1.06443103633532	-0.47685702844961
C	-4.59834870059306	0.30018971504680	-1.47115575624883
C	-4.70000026068961	1.38134887821162	0.78904702037624
C	-4.02500899166739	0.15024708891599	1.35985865011320
C	-3.21490061661608	-0.57678058482633	0.28802047908826
N	-3.99497903103212	-0.89977974230112	-0.90167203317372
H	-4.72874133182501	-1.55518351769116	-0.65182983220089
H	-5.18157652450364	0.02359958468853	-2.34912922722040
H	-3.78681807663581	0.94804330945191	-1.81416731966320
H	-2.37932758517400	0.05517373626119	-0.02566604023663
H	-2.78923143137182	-1.49366297446032	0.69503285375141
H	-6.33071813499956	0.44820473880757	-0.20127697837308
H	-5.85157881577097	1.98732356036737	-0.91731534722897
H	-5.33929615516915	1.85998120878251	1.53054618207996
F	-3.70093488837884	2.32456236947442	0.47149953823335
H	-3.39377399594606	0.43352535482255	2.20378348501246
H	-4.80544156173801	-0.51540376188685	1.73838135414045

10C_ee, Gas Phase

C	-5.43543551066717	1.06495634280322	-0.49571082254545
C	-4.55856631964368	0.31754011014118	-1.495887410849280
C	-4.63398136720500	1.39855586470931	0.74588174915441
C	-3.99753274116040	0.15485509645855	1.33189963741830
C	-3.17303639361730	-0.55980278489146	0.26541672333162
N	-4.01499944379017	-0.87638405459733	-0.87407377661467
H	-3.51218153391074	-1.43606286747679	-1.54845904250755
H	-5.15343612406623	0.02613789549766	-2.36109683786262
H	-3.76561825009710	0.99522742905538	-1.85328705180418
H	-2.31990979299981	0.07969457842724	-0.01567712772163
H	-2.76697253429842	-1.48519211441158	0.67302484282910
H	-6.27547868971275	0.43114178602146	-0.20479625005743
H	-5.83085809753257	1.97855489663524	-0.94092649169526
F	-5.46142673860710	1.98933094313491	1.69081405182421
H	-3.85919151784240	2.13342375713236	0.50212638415824
H	-3.37169885139768	0.42215681710498	2.18404571858889
H	-4.79170609345147	-0.50780369574435	1.68142570435330

10C_ee, Water

C	-5.43768505164479	1.06448986950278	-0.49439640761368
C	-4.56270613772291	0.31333459381186	-1.49360921272357
C	-4.62862870864536	1.39516708105361	0.73997322184659
C	-3.99955420532199	0.15425935851390	1.33341037142374
C	-3.17808421552195	-0.56351191861773	0.26645332939271
N	-4.02258143725201	-0.88904432458634	-0.87440663465714
H	-3.48772565481613	-1.41242341742267	-1.55613544047025
H	-5.15522681867677	0.02975787586202	-2.36258781500158
H	-3.76265179893461	0.98399939255025	-1.84222592441654
H	-2.33289169195013	0.07847483714161	-0.02499404621176
H	-2.76487711132816	-1.48414655206727	0.67671450616681
H	-6.28117339986687	0.43453443694102	-0.20145465278211
H	-5.82648323752365	1.97795341449647	-0.94558356205450

F	-5.46263439822687	1.99605397011696	1.69493864465599
H	-3.85767157453303	2.13217955378717	0.50030832683758
H	-3.36650507634793	0.42108937387263	2.18031515306698
H	-4.79494948168684	-0.50583754495629	1.68800014254071

10C_ea, Gas Phase

C	-5.44718003184861	1.07962238663514	-0.48255605830008
C	-4.58861929910582	0.29352357915851	-1.47872758877086
C	-4.63587616206024	1.41028102101517	0.75326728288765
C	-4.00647095349546	0.16762675948090	1.34868086591018
C	-3.20760039954959	-0.58078444752301	0.27671888483724
N	-3.98406847546948	-0.89711962020237	-0.90865254187674
H	-4.69868340537517	-1.57992472980855	-0.68651679935883
H	-5.17885479255914	0.01180378397972	-2.35019148141627
H	-3.77611258416325	0.93048619471900	-1.84388463091262
H	-2.35866767533684	0.03310348746316	-0.04220932200995
H	-2.79044072610895	-1.50037530443460	0.68600948470202
H	-6.30422589341440	0.47153359907805	-0.18017373387623
H	-5.82971791446835	1.99565431726259	-0.93549569792105
F	-5.45423894108285	2.01563161499180	1.69836734646425
H	-3.85577383938865	2.13662687598988	0.50114106997736
H	-3.36900267036764	0.43786023680763	2.19202648729143
H	-4.80530623620556	-0.47724975461302	1.72510643237250

10C_ea, Water

C	-5.44655959660868	1.07599671395509	-0.48307183976811
C	-4.58559943491444	0.29573098983933	-1.48132645898956
C	-4.62960997926150	1.40512064554334	0.74573611472752
C	-4.00724306075360	0.16503995474418	1.34631477773001
C	-3.20364465351315	-0.57947715019296	0.27555323319641
N	-3.98589084976688	-0.90191404144235	-0.90969034093089
H	-4.71926283604073	-1.55701014162410	-0.65923808823797
H	-5.18085016224068	0.01798196723786	-2.35030295378064
H	-3.77297002137032	0.93555733412378	-1.83946778197516
H	-2.35922119404645	0.04007345286645	-0.04249396818184
H	-2.78882041808998	-1.49697037772357	0.69113570907080
H	-6.29759818725721	0.45804624041790	-0.18365114550936
H	-5.82870954014170	1.99006283830349	-0.94006686915351
F	-5.45740330316870	2.01733283383087	1.70140194103133
H	-3.85513576054665	2.13673862758936	0.50062103970978
H	-3.36675686860219	0.43198590764910	2.18830912654517
H	-4.80556413367713	-0.48599579511775	1.71314750451603

11A_a, Gas Phase

C	-5.47163782932372	1.35940738804640	-0.30074536535532
C	-4.72873457889554	0.59869246323683	-1.39418580688826
C	-4.57121552736142	1.62463221224388	0.89072461096125
C	-3.92866289739065	0.34079897567083	1.39739611800493
C	-3.18896122970442	-0.33963650113100	0.24769681245292
N	-4.09458126673316	-0.59736643030494	-0.85442703474472
C	-4.40242709975567	-1.79796595603501	-1.37832452748087
O	-5.18399972489799	-1.98127033383538	-2.27720278805490
C	-3.69201615076267	-3.04322659528640	-0.77998836958810
F	-3.97197358913277	-3.18864879610109	0.52365711909149
F	-2.36185628127600	-2.96071223445220	-0.90561745930148
F	-4.09120009029811	-4.13248498769951	-1.40296059678379
H	-5.40222286219884	0.29877311076770	-2.19052082550582
H	-3.95086954274446	1.23868958571491	-1.81808661023644
H	-2.38981196919126	0.31850284108162	-0.10603362361187
H	-2.72943204791810	-1.25719059823977	0.59442859344141
H	-4.74547110441674	-0.32077405883895	1.70396139140168
C	-3.00087225641051	0.58104228546723	2.57465933786976
H	-5.84138808708423	2.30596601898515	-0.69657508349569
H	-6.33355443055819	0.77933353904121	0.03598069217710
H	-5.11851286127191	2.12242564685357	1.69420579781200
F	-3.56186011364137	2.50091052009476	0.49423363077031
H	-2.18653780839687	1.24828241570818	2.29309532430907
H	-2.57141891325013	-0.35942961677375	2.92069947633440
H	-3.53889173738524	1.03335910578571	3.40767918642093

11A_a, Chloroform

C	-5.47153254417970	1.36452066027805	-0.29756160078787
C	-4.73533407871651	0.59613781764801	-1.38879863573606
C	-4.57565790486472	1.62834580253604	0.89494546223357
C	-3.93191901249429	0.34718019867455	1.40108473837757
C	-3.19975333733152	-0.34749555643656	0.25560899191606
N	-4.11113590887137	-0.60795242149070	-0.84599280279007
C	-4.39671053768696	-1.79812234803736	-1.37783080319480
O	-5.16770750859708	-1.99172283406334	-2.29444381153932
C	-3.68269084886998	-3.04472996965460	-0.78605966720402
F	-3.96889910423200	-3.21461919581798	0.51007922426395
F	-2.35387071770207	-2.96005337458713	-0.90790142991588
F	-4.07519150641162	-4.13134736939201	-1.42882678246872
H	-5.41306192479663	0.30876846365726	-2.18510318347429
H	-3.94601935573799	1.22093641085075	-1.81286431137076
H	-2.39776164540379	0.29825585639844	-0.11211402060302
H	-2.74643606442990	-1.26453753222235	0.61145804163655
H	-4.75185570606688	-0.30433261986044	1.71918798625850
C	-2.99597509853701	0.58851595888087	2.57212570468735
H	-5.83611986219884	2.30997054047372	-0.70023217780305
H	-6.33633915294773	0.79232791686845	0.04397047413490
H	-5.12330939893496	2.12575797137765	1.69661549743176
F	-3.56174279967869	2.51308654262828	0.49985979986088
H	-2.16828751500578	1.23459352324099	2.27901423861438
H	-2.58384232393768	-0.35505424773769	2.93054999102653
H	-3.52695614236628	1.06367980578708	3.39697907644584

11A_e, Gas Phase

C	-5.42562394203148	1.36338568855010	-0.31271337445271
C	-4.65191849716132	0.63128335935347	-1.41540433680308
C	-4.46660258918714	1.65361721818381	0.83715841702974
C	-3.80434257112374	0.39144556218310	1.35367970361534
C	-3.13180157211790	-0.36061030595131	0.20738917214676
N	-4.05893444852078	-0.58418666024275	-0.89399008225923
C	-4.32038644463594	-1.84801119658902	-1.28186951878628
O	-3.85550039153189	-2.83465008310075	-0.77048767129493
C	-5.27197871907616	-2.05181399811735	-2.49416238507439
F	-4.81074892460958	-1.43063500004888	-3.58674714878030
F	-6.50160512569735	-1.58173637057466	-2.24597662886378
F	-5.37780430506700	-3.33529616565652	-2.76909098944202
H	-5.31159687950376	0.41123189396695	-2.24712993582480
H	-3.84400192652464	1.26528783946854	-1.79491059100367
H	-2.28917534221354	0.22466659182989	-0.17541797448779
H	-2.75427182221833	-1.32243742234770	0.53890110550700
H	-5.75361677362222	2.32085588175729	-0.72353504648062
C	-6.64499861673815	0.56992214491205	0.13736512667616
H	-4.55308337460590	-0.24192811759320	1.83079663944283
H	-3.06064527230552	0.64900659893991	2.10844161807693
F	-5.14672366088159	2.27632835650861	1.87001174011242
H	-3.69973634637208	2.35747631975576	0.49618909176578
H	-6.37428881303858	-0.43724884867477	0.45631605756870
H	-7.35736227109928	0.47301501432641	-0.68093558944259
H	-7.13801137011612	1.06738169916103	0.97021260105453

11A_e, Chloroform

C	-5.42759184441725	1.36347792387318	-0.30832351158949
C	-4.66099131986665	0.62416932281417	-1.40990038555757
C	-4.45941945544814	1.65552634684386	0.83094925663362
C	-3.80530333346874	0.39530956170221	1.35751335705955
C	-3.13915314919902	-0.36661777604425	0.21494913708710
N	-4.07510947573877	-0.59725031152711	-0.88241777354807
C	-4.31723784621172	-1.84646386917534	-1.28967485894433
O	-3.84078343175188	-2.84734738739594	-0.79793716467255
C	-5.26425660080344	-2.04699031359915	-2.50595518549171
F	-4.80560407001867	-1.42134911926982	-3.59499889864028
F	-6.49964594658496	-1.59788815239101	-2.26172551533832
F	-5.35535850937184	-3.33501731872755	-2.79071282753088
H	-5.32556265253075	0.40965030941719	-2.23868436725195
H	-3.84644763228014	1.24743158349771	-1.78850888472957
H	-2.30273286095898	0.21603904401676	-0.18124985309586
H	-2.75824625827642	-1.32229927161561	0.55732824529843
H	-5.75052034033377	2.31796953583609	-0.72837688626814
C	-6.64885713725532	0.57673274327346	0.14874751073746
H	-4.55456118955427	-0.23206082197486	1.84196313750424

H	-3.05180150302785	0.65317206719646	2.10202037551306
F	-5.13670270337875	2.29190578935093	1.87027233729364
H	-3.69390234500773	2.35596642754619	0.48611806959801
H	-6.37935162656694	-0.42353247371070	0.49061062910119
H	-7.35353922330325	0.46698141269885	-0.67489005838317
H	-7.15207954464472	1.08883474736431	0.96697411521557

11B_a, Gas Phase

C	-5.51577249272795	1.04217001775287	-0.54250499511221
C	-4.65893985729486	0.33317784550226	-1.57311541877916
C	-4.69866492148117	1.37434909237704	0.69404315344044
C	-4.08089710490699	0.11893651882471	1.30108518245087
C	-3.21821598294296	-0.57068808786750	0.25546644743943
N	-4.03518148609477	-0.89357005602711	-0.96612188776987
H	-3.44895378001472	-1.35851540932391	-1.66010433172409
H	-4.77079477412949	-1.55601335306895	-0.71077741080745
H	-5.23063621730689	0.01873549865566	-2.44206030646093
H	-3.83451329010737	0.96820174382823	-1.89256950439389
H	-2.41375865989711	0.08666248503317	-0.07344423747814
H	-2.79458383891468	-1.50097063328804	0.62643356397568
H	-6.37327276641365	0.42817933353534	-0.25609713226217
H	-5.90746409065119	1.95602470515204	-0.98741157604811
H	-5.30381561714838	1.89728988460855	1.43531963564888
F	-3.67214403066931	2.22213034311388	0.31177020668857
C	-3.26347611015563	0.43323229219497	2.54569207341830
H	-4.90463977310683	-0.54892318056072	1.57775342393561
H	-2.48069780476476	1.15732830348540	2.32327982524021
H	-2.80227489815690	-0.46402580228789	2.95639925739610
H	-3.90686250311440	0.85835845835999	3.31501403120192

11B_a, Water

C	-5.50266988542642	1.04756191087937	-0.54861415948859
C	-4.64315192884136	0.32690259544712	-1.56816325735468
C	-4.72127731028047	1.37223418588837	0.70750737848627
C	-4.07547252894565	0.12700093784026	1.29494231329947
C	-3.22438200324708	-0.57029981289128	0.24553900234764
N	-4.04087476672097	-0.89283799364167	-0.95849561075616
H	-3.45926176569381	-1.36689367489183	-1.64881726025031
H	-4.77821396640171	-1.55345187987708	-0.70699996680973
H	-5.22046636146169	0.01170265684582	-2.43146342198596
H	-3.81205891593283	0.94675324967180	-1.89761722626176
H	-2.41133152392155	0.07086946934640	-0.09186275987612
H	-2.81318827190476	-1.50192571620279	0.62352734795023
H	-6.35761063573777	0.42746054232743	-0.27079698482100
H	-5.89029724570322	1.96185567664534	-0.99553565811762
H	-5.35703733939625	1.85562245752347	1.44922232216637
F	-3.71409629472702	2.28902087081644	0.37867641974101
C	-3.25246817743043	0.43847585913283	2.53497177648866
H	-4.89560705482785	-0.54187132249578	1.57353809670096
H	-2.44345302329807	1.13035956776601	2.30083483177024
H	-2.81889274990782	-0.47092275819990	2.94993917121903
H	-3.88374825019328	0.89445317806967	3.29771764555203

11B_e, Gas Phase

C	-5.42684577843098	1.06852873430752	-0.57605364672807
C	-4.53739384039450	0.35188404001675	-1.58323328843273
C	-4.61678644246847	1.39079701420209	0.68272974465878
C	-3.95694930154702	0.14903340968119	1.26683718684536
C	-3.12769168191064	-0.58311471752804	0.22851399576891
N	-3.97554847175436	-0.89971590620008	-0.97066377480916
H	-3.42839173470477	-1.41309744299760	-1.66215136030815
H	-4.74831678806639	-1.50871113319497	-0.69098054298672
H	-5.09389215839839	0.06577157800167	-2.47311050504360
H	-3.67884439350540	0.95445315627365	-1.87922078653818
H	-2.30546109249575	0.03008273653901	-0.14007089679539
H	-2.72514351817591	-1.51734905352791	0.61110555535175
C	-6.69721211180386	0.28437094814925	-0.26552267304560
H	-5.71310666637117	2.01429939667619	-1.04038392796667
H	-3.30646840324105	0.43236087742344	2.09354245595914
H	-4.72441411853288	-0.50891007565768	1.67926892915949
F	-5.44657141182530	1.93287962379160	1.62859223053130
H	-3.85510154758063	2.14027158191540	0.44545571924102
H	-6.50696187601787	-0.67972548026277	0.21272953714481
H	-7.27125278370106	0.10686012563332	-1.17429880139258
H	-7.32119587907362	0.85061058675797	0.42199484938628

11B_e, Water

C	-5.43050978135079	1.06946559808424	-0.57047677966274
C	-4.54482768561467	0.34184556983550	-1.57366438603006
C	-4.60604887198227	1.38810006883998	0.67235787732503
C	-3.97324843252554	0.14581387676293	1.26795064731343
C	-3.13874446078747	-0.57993199510291	0.22906417596523
N	-3.96745030600118	-0.89307718082188	-0.96960214202732
H	-3.39644845289979	-1.37465341618499	-1.66403222544012
H	-4.71215063815099	-1.54575965454930	-0.71887130474454
H	-5.10837372590217	0.04568763724849	-2.45401863426256
H	-3.69780405457213	0.95278879371979	-1.88105995499885
H	-2.31581331374520	0.03871825194860	-0.12514639375467
H	-2.74318734735869	-1.51517614326077	0.61178526496894
C	-6.69967601400264	0.28737027864249	-0.25308935759785
H	-5.71021936228061	2.01369712196910	-1.04074736035567
H	-3.32518346728607	0.42123594000304	2.09875486359516
H	-4.74968198426411	-0.51499472949893	1.65711254547456
F	-5.42381716602626	1.98163247253041	1.63057317635482
H	-3.83381064364076	2.11875973887974	0.42249590741741
H	-6.49355901023402	-0.68391381549246	0.19883951956974
H	-7.26959472041101	0.11938685253686	-1.16618412199324
H	-7.32340056096365	0.84458473391005	0.44303868288329

11C_ae, Gas Phase

C	-5.49315244268074	1.04402401645664	-0.56028549660532
C	-4.60737249640689	0.31310367801782	-1.56086769404510
C	-4.72096201027066	1.36562843243198	0.70458975055173
C	-4.06705712105976	0.12096860280174	1.28790990246873
C	-3.22986660930602	-0.56571192882737	0.21146905561570
N	-4.05702128819921	-0.88239430962843	-0.94185795921495
H	-3.52933528347558	-1.41742480417003	-1.61744507514808
H	-5.19812772874716	0.01927038778653	-2.42841159767306
H	-3.82391339874995	1.00336420320086	-1.90519478005414
H	-2.38690973254271	0.09508228242604	-0.04352698859279
H	-2.81603287569181	-1.49135977549750	0.61377053432305
H	-6.33897548024374	0.40734579647093	-0.29082143588855
H	-5.88435849684186	1.96505415583168	-0.99373351594700
H	-5.36376125669075	1.84330839333117	1.44846425815780
F	-3.72620595297345	2.29434349249889	0.39253176010569
C	-3.25076707769118	0.44091935351444	2.52990228548779
H	-4.88354594438081	-0.55835443903952	1.55212168076192
H	-2.44576753746471	1.13669612636871	2.29376726267287
H	-2.80959145848192	-0.46427344805527	2.94842703006422
H	-3.87512580810109	0.89749978408068	3.29934102295949

11C_ae, Water

C	-5.49378575953082	1.04392389377411	-0.56138247827572
C	-4.60802095732562	0.30978950894519	-1.55906353080255
C	-4.73420909416392	1.35993976494618	0.71019802476267
C	-4.06748489715810	0.12220361025702	1.28842382192831
C	-3.23483531541430	-0.56959433750323	0.21230522041415
N	-4.06108279290103	-0.89338076831128	-0.94380281070177
H	-3.50730416920530	-1.39874838274122	-1.62385305346758
H	-5.19390662923604	0.02474426109388	-2.43244388559834
H	-3.81378686045981	0.98976118173813	-1.89817748592800
H	-2.39400839903965	0.08839772693881	-0.05531534393933
H	-2.81550061642821	-1.49119874435348	0.61756262378256
H	-6.34641565635656	0.41521323408482	-0.29368897863551
H	-5.88164858592675	1.96562001791288	-0.99665221481348
H	-5.38028587563883	1.83179525638120	1.45253143207969
F	-3.73920016634301	2.30961485853602	0.40573941704707
C	-3.24488453018150	0.44253344470074	2.52661621536149
H	-4.88359268328961	-0.55272972487223	1.56547572893380
H	-2.42201992439506	1.11542757576403	2.28301415213341
H	-2.82410438532991	-0.46809617108986	2.95435080006152
H	-3.86177270167599	0.92187379379827	3.28831234565763

11C_aa, Gas Phase

C	-5.50752785752476	1.05925476294351	-0.54969012996898
C	-4.63483982080186	0.29114813954101	-1.54303203429672
C	-4.72485954012838	1.37556276926852	0.71164366602285
C	-4.08435985580884	0.12764947726612	1.30667252977307
C	-3.26835765143569	-0.58824009079729	0.22585282160311
N	-4.02471448637701	-0.89934172254406	-0.97462093082750
H	-4.73396295706953	-1.59325918687569	-0.76989763087799
H	-5.21296989960020	0.00939730745722	-2.42288463274205
H	-3.82854169905366	0.94439463424601	-1.88620857243389
H	-2.42963282036951	0.05358101937350	-0.06194111745947
H	-2.84043963606003	-1.50742936850541	0.62888346500912
H	-6.37323011460704	0.45037387292925	-0.27326726754476
H	-5.88080330656913	1.98542020718218	-0.98994592281094
H	-5.35489596956402	1.87248685364783	1.45374262151124
F	-3.71594514616094	2.28330516255329	0.38429462805999
C	-3.25030943521674	0.44894009286933	2.53585590961795
H	-4.90951155883464	-0.53443579296244	1.59441262377090
H	-2.46135194311603	1.15872260439170	2.28716690660791
H	-2.78363361114055	-0.45320871634712	2.93334005001523
H	-3.86352269056140	0.88749797436255	3.32433301697092

11C_aa, Water

C	-5.50437500540069	1.05608824957699	-0.55253026891527
C	-4.63023886273639	0.29336219274144	-1.54639846876578
C	-4.73509381303624	1.36912938038657	0.71451163507125
C	-4.08002654965165	0.12896231101324	1.30228079358608
C	-3.26244341947548	-0.58599750923473	0.22392300561039
N	-4.02473904203229	-0.90484518991972	-0.97658334323717
H	-4.75619018595915	-1.56724473682179	-0.73934076144891
H	-5.21320823500445	0.01419507844250	-2.42368328035363
H	-3.82082251732604	0.94330433352743	-1.89004680647530
H	-2.42315332565434	0.05366110542737	-0.06873252138823
H	-2.83852522970952	-1.50394612378563	0.63354179971273
H	-6.36648861989035	0.44155264914610	-0.28016443929448
H	-5.88223761805479	1.98046864645836	-0.99272081492901
H	-5.37025782235082	1.85735246662304	1.45558903551266
F	-3.72760033147654	2.30210012002014	0.39596649933697
C	-3.24651466066027	0.44786527234795	2.53282759537010
H	-4.90150464273005	-0.53616949762454	1.59003477530966
H	-2.43949264575960	1.13843751788717	2.28487563819189
H	-2.80131559847196	-0.46096800117253	2.93979296683708
H	-3.85918187461933	0.90451173496063	3.31156696026897

11C_ee, Gas Phase

C	-5.42694471208787	1.06754472829188	-0.57847729871064
C	-4.51807649132984	0.31811901021064	-1.55999283158815
C	-4.61063130707058	1.38453212665438	0.66989746424416
C	-3.96908673931941	0.14769856980588	1.26604109015894
C	-3.14004600175936	-0.56392688039245	0.20073632607503
N	-3.98257180264576	-0.87948115263776	-0.93805244764452
H	-3.48654890584392	-1.44519271959498	-1.61190792468284
H	-5.09723015782395	0.03152189351198	-2.43880964975596
H	-3.71829584240085	0.99861500273703	-1.89567918965406
H	-2.29064407020786	0.08116135785728	-0.08021897029622
H	-2.72912888879087	-1.48790162042431	0.60656332252601
C	-6.69703921676545	0.28713677568117	-0.26361327366755
H	-5.70527154807422	2.01938355922634	-1.03830742808509
H	-3.34145891896565	0.43347923226792	2.11081189117004
H	-4.74832698558072	-0.52135458251316	1.63369690928786
F	-5.41423060551951	1.99875257588339	1.62125436320679
H	-3.83001182757379	2.10714277418214	0.40931789446979
H	-6.46506776246301	-0.72306263504187	0.07001654618608
H	-7.31801357011019	0.20825653073234	-1.15659646350006
H	-7.27590464566719	0.78882545356215	0.51040967026039

11C_ee, Water

C	-5.42936294235583	1.06510296957511	-0.57439453367230
C	-4.52226443997828	0.31584040390349	-1.55725619941552
C	-4.60511870824017	1.37644258702524	0.66705722371259
C	-3.96845772249295	0.14088513587842	1.26549405937786
C	-3.14180401767938	-0.56910083962616	0.19707920525548
N	-3.98531750060534	-0.89093374202408	-0.94481220135869
H	-3.45254472088698	-1.41197012591685	-1.62957477411866
H	-5.09881165397881	0.03958459307808	-2.44068780868150
H	-3.71797973413867	0.99335016844526	-1.88260858977613
H	-2.30049621682819	0.08030044527925	-0.09107957572491
H	-2.72311584557678	-1.48927943429061	0.60282494841371
C	-6.70454424617066	0.29263236508852	-0.25892584992902
H	-5.70302448761665	2.01603451316725	-1.03832491585452
H	-3.33309378013889	0.42490367814302	2.10507125213932
H	-4.74640769867529	-0.52868098831799	1.63699020351312
F	-5.41213944914527	1.99824930985022	1.63124768548756
H	-3.82821355360046	2.10127748459661	0.41011159078983
H	-6.48352862305572	-0.69576927329136	0.14165628283810
H	-7.29243306678786	0.16245409014957	-1.16832572903197
H	-7.31587159204781	0.82992665928698	0.46554772603564

11C_ea, Gas Phase

C	-5.43646078472431	1.07406885779122	-0.56732798482654
C	-4.54139487399297	0.29793609774190	-1.54903168415968
C	-4.61318051606321	1.39197485162508	0.67512747575578
C	-3.97803164951816	0.15655945124849	1.28081254889383
C	-3.17634526268836	-0.59230266389892	0.21032083562311
N	-3.94015294158130	-0.89610029038575	-0.98591900667088
H	-4.64269160259431	-1.59846661848854	-0.79433737057127
H	-5.11790926670029	0.02460486124067	-2.43400333393259
H	-3.72564507543622	0.94438101550965	-1.88905421423492
H	-2.32197382358286	0.02073638175233	-0.09620951754482
H	-2.76773921362542	-1.51660202227908	0.61746378277290
C	-6.70915383401683	0.30113267602682	-0.24637768000717
H	-5.71511929785537	2.02346819959694	-1.03283883859501
H	-3.33358833356523	0.44645622165660	2.11227215177103
H	-4.76092005253795	-0.49119604931969	1.68111417439186
F	-5.41084719846716	2.01886224319758	1.62532212704976
H	-3.82900511635576	2.10790851919896	0.40685845029206
H	-6.49261221552865	-0.69262660782817	0.14775985129932
H	-7.30640016455815	0.17584826511130	-1.15038569366462
H	-7.31011877660748	0.82643661050261	0.49340392635783

11C_ea, Water

C	-5.43641352502071	1.07418998402723	-0.56562736930343
C	-4.54159202274222	0.30150495017228	-1.54993884826858
C	-4.60513423958799	1.38797123638058	0.66912058440404
C	-3.97885364962756	0.15336702777964	1.27872675976678
C	-3.17356749448251	-0.59110818182709	0.20865090815613
N	-3.94470330578649	-0.90058994527207	-0.98699351768649
H	-4.66569507487096	-1.57762594338125	-0.76547380947346
H	-5.12306309091559	0.03330960357209	-2.43295027570173
H	-3.72537098818190	0.94966253143249	-1.88354637600797
H	-2.32469913992329	0.02837245408094	-0.09802711378112
H	-2.76615329162078	-1.51355527191997	0.62034899832161
C	-6.70713890300425	0.29574685867632	-0.24742966031927
H	-5.71306973879437	2.02236635942191	-1.03411698690162
H	-3.33101432125581	0.43861162930884	2.10908155286879
H	-4.76150225920995	-0.50027560312408	1.67001408195470
F	-5.40542838024918	2.02573039005272	1.63083902067728
H	-3.82339503293797	2.10492719284269	0.40438454685130
H	-6.48747395530900	-0.69404683927272	0.15460687953293
H	-7.29313730129993	0.16188734202623	-1.15756699060038
H	-7.32188428517951	0.82263422502321	0.48086761551049

12A_a, Gas Phase

C	-5.35141246300015	1.51091190098500	-0.22744175154024
C	-4.75097492863428	0.58731432715781	-1.29049461705169
C	-4.48631126198562	1.68164107985408	1.00455445373019
C	-4.07980532005669	0.33307566849497	1.56012083635346
C	-3.39039948410220	-0.49663740405993	0.48896694666155
N	-4.25336876740094	-0.65139600875851	-0.67117720368673
C	-4.40417978259490	-1.79018083113426	-1.37768352025543
O	-4.96692806258518	-1.86894156004381	-2.44083516515922
C	-3.83504136499833	-3.10539182989043	-0.77624092085501
F	-4.33480044574165	-3.35218345873907	0.44222988733912
F	-2.50084063608902	-3.05790797635571	-0.66995592608268

F	-4.14241715471841	-4.12385509596380	-1.55313070467157
H	-5.55229958678453	0.28126492302615	-1.95873691084377
C	-3.67102531620021	1.25692228894074	-2.13439247392534
H	-2.46322607741942	-0.00352416139365	0.18604274340769
H	-3.12848683535203	-1.46785346272002	0.88925141811261
H	-4.97541230203009	-0.18733675216333	1.90713732748581
H	-3.41146486714357	0.46635869643793	2.41000076698571
H	-5.01032007185931	2.27281511654980	1.75693147024701
F	-3.33226119748956	2.39046154162238	0.67600472973402
H	-5.55136943739959	2.48584572980475	-0.67367060994789
H	-6.30862543033422	1.10195300431275	0.10450380836220
H	-2.80632662083395	1.54068787887198	-1.53755302058709
H	-4.07165680947495	2.16446744019435	-2.58714308563766
H	-3.35659577577121	0.58713894496984	-2.93368847817504

12A_a, Chloroform

C	-5.35603344450294	1.49267802846478	-0.23806440252682
C	-4.73117741056353	0.59432859483489	-1.30835009977205
C	-4.51108792426567	1.66288816603250	1.00586144785924
C	-4.06651911656572	0.32031910279730	1.54218284788779
C	-3.36012435497481	-0.48394082081135	0.46310127663469
N	-4.22238375960207	-0.64839691971820	-0.70013154855400
C	-4.42268013821989	-1.79359764674686	-1.36012477346411
O	-5.02454556936872	-1.89966787247535	-2.40835232634135
C	-3.86306144043875	-3.10697749247496	-0.74475501416363
F	-4.33312468417604	-3.32348612876829	0.48868404202231
F	-2.52720485268138	-3.09533386381404	-0.68185400278676
F	-4.21863445196493	-4.13678150019027	-1.49473325917476
H	-5.52363916987350	0.29322858385506	-1.98779398867518
C	-3.64632061325166	1.28699930101737	-2.12602764506762
H	-2.44910935354553	0.03182268908970	0.15243123516532
H	-3.06891329084242	-1.44944645683811	0.85602608809451
H	-4.94961645948426	-0.22185131248106	1.88731048084454
H	-3.39865310944799	0.45844381978230	2.39145742303879
H	-5.05777529516335	2.22534844536016	1.76220287040112
F	-3.36985452164647	2.41920568478222	0.70108738592086
H	-5.57176082723485	2.46815128232005	-0.67546463016047
H	-6.30733273798954	1.06104601319546	0.08047757103973
H	-2.79132523274623	1.56615981982743	-1.51315294163359
H	-4.05386055843277	2.19798561060629	-2.56524864712359
H	-3.31081168301698	0.63652487235297	-2.93316938946499

12A_e, Gas Phase

C	-5.34939520835138	1.33144649555853	-0.53303988188621
C	-4.38742822851261	0.60666016319802	-1.47428490752796
C	-4.70094313927076	1.57398612777928	0.81771811283284
C	-4.11010476941076	0.30219809958472	1.39672432944135
C	-3.17509492456623	-0.35510387937902	0.37924833679315
N	-3.93171157422477	-0.63088127463944	-0.85605891327744
C	-4.56226560488233	-1.81199900866670	-1.04181063560204
O	-4.52456673285091	-2.75614402230462	-0.29695749192992
C	-5.38216971320101	-1.98358190564477	-2.35617070708555
F	-4.68264438175669	-1.63593111123794	-3.44155257445337
F	-6.49530073946899	-1.23526475226587	-2.32946746336782
F	-5.74640869501485	-3.24206849265321	-2.49308183652623
H	-2.43982622281871	0.39323842760632	0.06599926387369
C	-2.39007624289128	-1.51441136164142	0.97074983932259
H	-4.91196581074424	-0.39609147158719	1.64988590217544
H	-3.56165194463914	0.53083120971938	2.31201072523912
H	-5.66527950010493	2.27812474890802	-0.97107941344308
H	-6.23793671616100	0.71288607849788	-0.38620387318192
F	-5.64284276732031	2.09086233617634	1.69036706482503
H	-3.91326271948189	2.32878690741374	0.71921150739474
H	-4.85827116666959	0.41248959065411	-2.43019031108224
H	-3.50507605595261	1.21900656922099	-1.67164882803328
H	-3.02375482429726	-2.19778586674802	1.52707578854114
H	-1.64215106845215	-1.09809452275993	1.64706123697864
H	-1.87322124895559	-2.08094908478919	0.19807472997932

12A_e, Chloroform

C	-5.35456264210378	1.33982797869985	-0.51977685164102
C	-4.40971924611465	0.59968894449719	-1.46510190362859
C	-4.68229268408126	1.58822074041204	0.81586078725311

C	-4.10437718067973	0.31703309572238	1.40445469611537
C	-3.18727227644417	-0.36107603552547	0.38467384517181
N	-3.96707464662694	-0.64190430025864	-0.83879510534767
C	-4.54560983366559	-1.82898615047023	-1.06564890578709
O	-4.46812119239260	-2.81006247430286	-0.36126949730140
C	-5.37313498864754	-1.98596871067898	-2.37780788013001
F	-4.68591191509244	-1.61842440661626	-3.46318527255926
F	-6.49913772601089	-1.26232545553617	-2.33721416694778
F	-5.71966883216936	-3.25276806989812	-2.53307448299669
H	-2.45085022777012	0.37606649904418	0.05270042326496
C	-2.40540315138113	-1.51606128858145	0.98565780878327
H	-4.91302617072624	-0.36758223789919	1.67406762166428
H	-3.53694052549428	0.54851332670745	2.30705578098319
H	-5.66080038119334	2.28442221530808	-0.96816766024500
H	-6.24606979612665	0.72968138061696	-0.35550267218500
F	-5.61507115055093	2.12667327992060	1.70209300022369
H	-3.89198927466255	2.33542381834589	0.70452378081466
H	-4.89211055702261	0.41050052685574	-2.41553170049456
H	-3.52071852779780	1.19810397975461	-1.66905262234201
H	-3.03822111259023	-2.19063337959593	1.55453542426028
H	-1.66032704856307	-1.08753422601461	1.65716553238986
H	-1.88493891209209	-2.08861905050703	0.21992002068160

12B_a, Gas Phase

C	-5.46175214780796	1.02002285875724	-0.48898905739059
C	-4.56552293517118	0.36059892676318	-1.51790326630336
C	-4.67736261035401	1.37163487472208	0.76328057111658
C	-3.97956850697016	0.14593418382087	1.33267341557521
C	-3.11221129344531	-0.59685679670286	0.32104670103148
N	-3.93783748838290	-0.86916384385647	-0.92691197164132
H	-3.33808925208423	-1.31555039661417	-1.62248659638105
H	-4.67531860262954	-1.53908231654792	-0.69966675120607
C	-1.82848064954602	0.11511322428033	-0.05846312301922
H	-2.87114984622712	-1.58517812865751	0.71125539219455
H	-3.34861688488775	0.43684639172740	2.17212611999309
H	-4.74769862487932	-0.52217718950403	1.73121193426162
H	-6.29395476673232	0.36316702209295	-0.22225235227918
H	-5.88942072177813	1.92312221181409	-0.92197079990831
H	-5.33333363951917	1.80738105929949	1.51642993604200
F	-3.73730909214424	2.33011197335448	0.42732410626294
H	-5.11194628282555	0.05476806564878	-2.40621833489367
H	-3.75370928761687	1.02159557814947	-1.81200163182973
H	-1.25333841677120	-0.45760124110151	-0.78666080661686
H	-1.21813455898418	0.21204552924101	0.83811991472969
H	-2.00205439124283	1.11927801331312	-0.43537339973781

12B_a, Water

C	-5.43827236984107	1.03355892064533	-0.49601194946165
C	-4.54874572153870	0.35065339018276	-1.51510612098783
C	-4.68601927562021	1.36539733489251	0.77605711465959
C	-3.97578281142964	0.14536291806062	1.33037720499210
C	-3.11473686076583	-0.59394010270811	0.31240121705126
N	-3.93958602183086	-0.87073038478187	-0.91547798352719
H	-3.35772534487255	-1.34047470193382	-1.60974051153114
H	-4.68037540990364	-1.53302379892395	-0.67907968421447
C	-1.83492003361145	0.12373021850970	-0.06992562495844
H	-2.86655623230092	-1.57674940981233	0.70718610123333
H	-3.34875205778744	0.42867934282701	2.17516710596252
H	-4.74613629696698	-0.52867881098395	1.71178330577567
H	-6.27608484736909	0.38237700285400	-0.23714631139855
H	-5.85110437710550	1.94137335589705	-0.93300063601583
H	-5.36305927748263	1.77324160187250	1.52493051605302
F	-3.75265653567665	2.37208568666652	0.49473356828398
H	-5.10866567127534	0.03503313389402	-2.38980294190193
H	-3.73343879467902	0.99438803785637	-1.83432952717072
H	-1.27074088120363	-0.45538996003875	-0.80039388602427
H	-1.22597484018702	0.22923149084065	0.82669338995638
H	-2.01747633855180	1.11988473418376	-0.46474434677584

12B_e, Gas Phase

C	-5.34266275915710	1.07264238129165	-0.50334917219774
C	-4.49640435807597	0.35823317033020	-1.54359356911422
C	-4.56108911957702	1.39408658978149	0.76074279113159

C	-3.91321936936684	0.14188850472415	1.33132686110257
C	-3.07502508408998	-0.57595803168698	0.29150746684954
N	-3.91731282474741	-0.88742288160670	-0.90954587793146
H	-3.36911755624571	-1.39581303986704	-1.60548334288043
H	-4.68884532160138	-1.50353867633633	-0.63865584092858
C	-5.26873195593736	-0.02762601517059	-2.78441800220372
H	-3.62431041720354	0.96100841161479	-1.80584907171709
H	-2.25264964103131	0.04216188036239	-0.06817492270925
H	-2.66962003356270	-1.51110965704684	0.66912117443032
H	-3.27813536619013	0.39803282690500	2.17842180803573
H	-4.70129370350933	-0.51690335176169	1.70624644659983
H	-6.20498693593707	0.45306092090903	-0.23592959337451
H	-5.73341302550588	1.98932316364611	-0.94478503208795
F	-5.42236976844500	1.90954349318788	1.69245109767734
H	-3.80134500270786	2.15423299906025	0.55283168360515
H	-6.12721343531326	-0.65265137724778	-2.53311604304198
H	-4.64345075402090	-0.55149169440537	-3.50778070522231
H	-5.64538356777424	0.87365038331640	-3.26463815602282

12B_e, Water

C	-5.34677760971689	1.06959349984117	-0.49908925908039
C	-4.49493193178371	0.35405241685223	-1.53549182005169
C	-4.55018473131766	1.39199805199920	0.74811378112342
C	-3.92599396949418	0.14005830303687	1.32836717719544
C	-3.08666977130553	-0.57367653992788	0.28641947445473
N	-3.91498010319250	-0.88046674480158	-0.91323647108189
H	-3.34659693329007	-1.37183664759157	-1.60437087247421
H	-4.66936571481688	-1.52278720904018	-0.65904970712845
C	-5.27221548400788	-0.02615837510924	-2.77418459511954
H	-3.63128235993433	0.96477102062076	-1.80137072694662
H	-2.26182560800544	0.04524329154869	-0.06210854301385
H	-2.69200848801779	-1.51195346315397	0.66285727946088
H	-3.28994261894184	0.38815336795600	2.17667883668782
H	-4.72210270930892	-0.52005558719422	1.68088313090415
H	-6.19631868091181	0.43690019604109	-0.22556709238082
H	-5.73671769253780	1.98299086586508	-0.94701190796990
F	-5.40501805922984	1.95193540910567	1.69495546892499
H	-3.78445674125850	2.13985281571591	0.53158503503848
H	-6.11777182225946	-0.66472240911377	-2.51564162163615
H	-4.63722885594116	-0.54633546562377	-3.49040933594770
H	-5.65419011472779	0.87779320297353	-3.24499823095869

12C_ae, Gas Phase

C	-5.42444947574738	1.03645905073249	-0.50206534828551
C	-4.51058314748787	0.34520246807239	-1.50357254694610
C	-4.66836682110172	1.35993471399248	0.77105222732601
C	-3.96925996571446	0.13048282168572	1.32071063167549
C	-3.13062047057446	-0.61452680374194	0.27766617967989
N	-3.95622974092714	-0.85876779115866	-0.90329222680853
H	-3.43001729338153	-1.38475099999801	-1.58836405568689
C	-1.79954213513037	0.07849499219723	-0.01881688720141
H	-2.90223785400748	-1.59654257752051	0.69942493200957
H	-3.34952046100640	0.41360186624641	2.17267116453608
H	-4.74915598209907	-0.54266133854171	1.68498609689067
H	-6.25084267355601	0.36870584521695	-0.24750732200248
H	-5.84105644042045	1.95225772805817	-0.92237421189709
H	-5.33740002912483	1.78139942689577	1.52395921223061
F	-3.72579612774360	2.35195590695927	0.49151732606640
H	-5.08442231185141	0.05431016189281	-2.38339039102715
H	-3.74509379030882	1.05952081184806	-1.83414071714174
H	-1.21328731059645	-0.52281032026093	-0.71581384750920
H	-1.21849226327894	0.19595506789508	0.89703375887584
H	-1.94370570594159	1.06851896952892	-0.44662397478445

12C_ae, Water

C	-5.42072085411542	1.03939412141430	-0.50330225794725
C	-4.50876521270148	0.34176502341180	-1.50170402054934
C	-4.67802850663820	1.35424822737338	0.77757506776130
C	-3.96889428756671	0.13139354325981	1.32326386048437
C	-3.13260045479432	-0.61626235248757	0.28088267436053
N	-3.95798582627851	-0.86933381280454	-0.90348615995846
H	-3.40641393520837	-1.36764790065910	-1.59176303662565
C	-1.80430358943177	0.07547866226385	-0.02783799275015
H	-2.89685640712100	-1.59410847544251	0.70699231407027
H	-3.34705757661059	0.41546699175882	2.17347405188432
H	-4.74770451340705	-0.53836535944216	1.69672311187467
H	-6.25665279550138	0.38251687051617	-0.25033521333712
H	-5.83101047902737	1.95694792287325	-0.92632392056159
H	-5.35048285116503	1.77057821950748	1.52795849395045
F	-3.73327587411565	2.36517594746449	0.50943147643129
H	-5.07966128398098	0.05773315275316	-2.38536494655727
H	-3.73325857072753	1.04556294624293	-1.82963401951913
H	-1.22225732340001	-0.53496788750077	-0.72028657708924
H	-1.22294394301201	0.20193097114636	0.88685231761231
H	-1.95120571519659	1.05923318835083	-0.47005522353430

12C_aa, Gas Phase

C	-5.42894364615358	1.05436138129497	-0.49113401289276
C	-4.53621929769466	0.31352372876256	-1.48474758587554
C	-4.66304213522385	1.36901700278805	0.78039016411404
C	-3.97980999460223	0.13408791275620	1.34260500434209
C	-3.16752176247891	-0.64128417969018	0.29207363939035
N	-3.93283041640292	-0.88783161381639	-0.93007874993489
H	-4.65118023759917	-1.57885228437452	-0.75076773628321
C	-1.83582459766144	0.03002952378150	-0.03036283704724
H	-2.93773375063492	-1.62381511297842	0.70980293709857
H	-3.35103135377908	0.41915565474078	2.18807829347926
H	-4.76820554454066	-0.51907404792932	1.72902602953274
H	-6.28433354469183	0.42439096094849	-0.22922127014060
H	-5.81708941951464	1.97962443242674	-0.91975176826791
H	-5.32280584703503	1.80825149098169	1.53101303137778
F	-3.70583479913935	2.34282343378844	0.48968639171965
H	-5.10081414541625	0.03902884577133	-2.37568817578252
H	-3.73723687503968	0.97962172269742	-1.81570506807286
H	-1.28028914639212	-0.57604852293241	-0.74452004351411
H	-1.24118207089778	0.13742366260432	0.87754104029345
H	-1.97068141510190	1.02580600837875	-0.44753928353628

12C_aa, Water

C	-5.42193653317470	1.05606929887545	-0.49309732421998
C	-4.53274973908008	0.31552997129206	-1.48819128047167
C	-4.66925779353847	1.36001050439660	0.78547460332890
C	-3.97899787876377	0.12915645935080	1.33942388937885
C	-3.16375298733278	-0.64144274755014	0.28995117491971
N	-3.93499312363242	-0.89431482152201	-0.93245556085035
H	-4.67641804363387	-1.55292092399939	-0.71811106008210
C	-1.83424051237137	0.03190009931955	-0.03602648014957
H	-2.93334889469302	-1.62159325872763	0.71160980586485
H	-3.35317249624196	0.40813538122615	2.18917610174111
H	-4.76720621483264	-0.52745143636236	1.71843108356297
H	-6.27824762721791	0.42657595016645	-0.23577311523994
H	-5.80855659607595	1.98209150571850	-0.92167018601896
H	-5.33188222744056	1.79384900259862	1.53440170948072
F	-3.70615884557223	2.35211738997425	0.50888071135858
H	-5.10647277412794	0.04285577680619	-2.37368199211657
H	-3.73094964648017	0.97625664842591	-1.82411714279090
H	-1.25968298527817	-0.58646396774230	-0.72595572463469
H	-1.25188327583653	0.16556388647938	0.87647916530844
H	-1.97270180467544	1.01431528127394	-0.48404837836938

12C_ee, Gas Phase

C	-5.33195958327591	1.08243377197532	-0.50955044451128
C	-4.46219960549647	0.34923898117412	-1.53054881815853
C	-4.55128681179563	1.38456295177670	0.75263186381546
C	-3.93086163440634	0.12555816681623	1.32040425380806
C	-3.09213398974921	-0.56118813216163	0.24810407655050
N	-3.91843337570435	-0.84874625628602	-0.91003312409386
H	-3.40782053222442	-1.39548142562750	-1.59110133496338
C	-5.26684988321172	-0.03322106216099	-2.75932270837937
H	-3.65140094085721	1.03401865728309	-1.83578329293565
H	-2.23445798930952	0.08399705678203	-0.00364955647031
H	-2.69224959384795	-1.49753931423019	0.63627851747527
H	-3.31871214420639	0.36863242207855	2.18946953119499
H	-4.73297867200752	-0.54347202248998	1.63826498962131
H	-6.18159887369803	0.44644219254494	-0.24769910899684
H	-5.71652680406047	2.00827435346398	-0.94059331421282

F	-5.39603958029305	1.95708062255179	1.69419861229589
H	-3.76958357348270	2.12217027960811	0.54242106191112
H	-6.09349870424081	-0.68339617017635	-2.47057875615016
H	-4.64880397762646	-0.56834657190988	-3.48231147295634
H	-5.67155373050585	0.85195149898769	-3.24964097484404

12C_ee, Water

C	-5.33642659023360	1.07725303117339	-0.50700673869314
C	-4.46798135030850	0.34150439194980	-1.52726958810558
C	-4.54683318847657	1.38024141577975	0.74640684784352
C	-3.93097446982505	0.12576899796141	1.32243272466690
C	-3.09513465628314	-0.56345764349946	0.24938639626637
N	-3.92462773526227	-0.86355766009450	-0.90930050471570
H	-3.37923273186653	-1.37211260842730	-1.59572850813353
C	-5.26927950325514	-0.03074669318270	-2.76103622489880
H	-3.65197222750202	1.01952233059335	-1.82560559695998
H	-2.24721012145425	0.08620992922943	-0.01461435327805
H	-2.68571951971745	-1.49388295328020	0.64083747412048
H	-3.31086686669739	0.37057950236219	2.18529531875044
H	-4.73300778009753	-0.54164939881951	1.64712824621295
H	-6.18672086783951	0.44218007616324	-0.24131155847038
H	-5.71748111240088	2.00159710886602	-0.94422091485625
F	-5.39838658487592	1.96232313466319	1.69820387204584
H	-3.77057533017802	2.12076108377203	0.53776127359511
H	-6.11510752260979	-0.66133469740107	-2.48090552465265
H	-4.65131428732309	-0.58051308315940	-3.47274265251137
H	-5.65009755379335	0.86228373535036	-3.25674998822615

12C_ea, Gas Phase

C	-5.35096865471396	1.08904483931469	-0.49563688733098
C	-4.49083617959779	0.32532331713958	-1.51170289910736
C	-4.55416461068419	1.39655815410709	0.75573263066699
C	-3.93206998062569	0.14471136341465	1.33751553510890
C	-3.12974510385626	-0.58786453751335	0.25842154888069
N	-3.89599977523793	-0.87416098797449	-0.94043002898981
H	-4.62251228314758	-1.55432957728050	-0.74212139450175
C	-5.27430895427336	-0.03322761185259	-2.76067535513861
H	-3.65316519093639	0.97085029038150	-1.80311367405258
H	-2.27212106167260	0.02435424008677	-0.03915325012180
H	-2.72440630384742	-1.51938428404513	0.65231071142125
H	-3.29749252212754	0.40101999494954	2.18733784467581
H	-4.73442280542436	-0.50329184623127	1.70054249725436
H	-6.21039554180283	0.46935655470852	-0.21970982655852
H	-5.73261300463113	2.01526446916292	-0.92951817445715
F	-5.38413784891807	1.98693049972621	1.70062359670303
H	-3.77127367743958	2.12716482515907	0.52585808303383
H	-6.10050017809107	-0.70363538061268	-2.51250095003933
H	-4.62919642881239	-0.53861111030596	-3.47812822175107
H	-5.69351989415985	0.85703678766542	-3.22905178569590

12C_ea, Water

C	-5.35142669612571	1.08237321650259	-0.49723117444898
C	-4.48811349111215	0.32223060389862	-1.51333646036845
C	-4.54779493772203	1.39169583489696	0.74595581792414
C	-3.93160909263938	0.14440147976336	1.33600401177752
C	-3.12682721267289	-0.58843953947581	0.25934439843963
N	-3.90108622278195	-0.88400509231827	-0.93742407005716
H	-4.64678160816348	-1.53472515968480	-0.70590396554158
C	-5.27336091281259	-0.02901007277854	-2.76337818168358
H	-3.64948707104771	0.96836444340990	-1.79727748886743
H	-2.27371424241076	0.02761047118429	-0.04106937951467
H	-2.72398754119605	-1.51703861461649	0.66174373194447
H	-3.29238433533196	0.40058147545586	2.18224813636035
H	-4.73272027850893	-0.50827995059789	1.69341214194137
H	-6.20500323301228	0.45452321112766	-0.22131980520467
H	-5.73232454971082	2.00604186740892	-0.93679272719879
F	-5.38688413659916	1.99087243679375	1.70017614378311
H	-3.77129071943964	2.12722673616628	0.52013905751419
H	-6.08999644775730	-0.71181466312287	-2.51780733562384
H	-4.62764491327059	-0.51492803829627	-3.49493721016114
H	-5.70141235768461	0.86542935428274	-3.21594564101448

13_e, Gas Phase

C	-5.52703409219049	1.02935549136378	0.40525231639111
C	-5.09226054391193	0.62713187592862	-0.99782735035010
C	-4.36121353585887	1.59883272233949	1.19322130445060
C	-3.17442862128621	0.64976219680509	1.18327101398434
C	-2.80843530200452	0.24918507704816	-0.23781117391529
N	-3.97849214341395	-0.28297318541305	-0.90533980897708
C	-3.96256040627514	-1.56643331300976	-1.38208004632726
O	-2.99475353396394	-2.27865447153557	-1.23912911188474
C	-5.18919877979375	-2.07318791570765	-2.10414562487040
H	-5.41080973931148	-1.47016902335766	-2.98406533457541
H	-6.06614203949495	-2.07392047429332	-1.45750207581451
H	-4.97213258636211	-3.09094293872800	-2.41232039691733
H	-5.92143748908641	0.17021621494739	-1.52793308626828
H	-4.79648258271537	1.53074932329444	-1.54245215355834
H	-2.44739440184741	1.13655624280439	-0.76865453446590
H	-2.03066140489766	-0.50798571683782	-0.24204823946252
F	-6.52125174338349	1.98289506145162	0.30337024765188
H	-5.95409610519252	0.15950543154230	0.91297576488373
H	-4.66523659422655	1.81909192254464	2.21597412627653
H	-4.05246299236831	2.53455858142519	0.71918541831878
H	-3.38211483550677	-0.24831118038429	1.77124471686002
F	-2.09057052690819	1.28207807777200	1.76120402857017

13_e, Chloroform

C	-5.52282984552047	1.02012545051187	0.40126571262455
C	-5.08883977888821	0.62257322596808	-1.00228229933477
C	-4.36343656060444	1.59727963005114	1.19150293920867
C	-3.18176595193195	0.64509807922527	1.18197227130908
C	-2.80578464187329	0.25161357693107	-0.23783972822361
N	-3.97240689854711	-0.28989019580479	-0.90654978125671
C	-3.96261466288359	-1.56117337616102	-1.38492012570666
O	-2.98452821778017	-2.27986256949468	-1.25423723187332
C	-5.19202858867347	-2.06718916382280	-2.09438584628240
H	-5.41847865872774	-1.45900652198265	-2.96930408988236
H	-6.06064197369663	-2.05922070259309	-1.43697697036145
H	-4.98848628992017	-3.08638563279851	-2.40745274575870
H	-5.91608659276928	0.16602240918895	-1.53417985527368
H	-4.78335698564668	1.52270757758923	-1.54499539935622
H	-2.45810083467505	1.14222127869564	-0.77035401429570
H	-2.01801480653667	-0.49413312669720	-0.23681573071867
F	-6.52074095412114	1.98038417812304	0.29390121206664
H	-5.95599542307374	0.15601829644106	0.90995537602575
H	-4.67126396374061	1.81138301806786	2.21439149766172
H	-4.05424969764085	2.53139987712075	0.71444725985771
H	-3.38894867024175	-0.25022533568002	1.77159368265084
F	-2.09057000250702	1.27760002712080	1.76565386691929

13_e, DMSO

C	-5.52196903678386	1.01857802630263	0.40115098668968
C	-5.08892671973740	0.61984786975871	-1.00206531033637
C	-4.36363242657411	1.59791416548546	1.19104853116094
C	-3.18419718921990	0.64408118265133	1.18243840112619
C	-2.80583723680790	0.25127495573641	-0.23678479576210
N	-3.97177272252128	-0.29355130546140	-0.90474000204558
C	-3.96174110069563	-1.55939632802843	-1.38776029802674
O	-2.97812010459868	-2.27828149679433	-1.26556500022407
C	-5.19211331039448	-2.06583312631640	-2.09248451663211
H	-5.41928072649977	-1.45721682022454	-2.96694660087287
H	-6.05886049325226	-2.05363622721185	-1.43287151458779
H	-4.99304960456683	-3.08594318170637	-2.40566899801533
H	-5.91590973443410	0.16287659336162	-1.53356191890589
H	-4.78046329815635	1.51788428128997	-1.54589232013096
H	-2.46204904349947	1.14224220899271	-0.77074540066100
H	-2.01531380170727	-0.49124928856507	-0.23384433926056
F	-6.51943505393125	1.98196167178798	0.29096747209115
H	-5.95768863361241	0.15738975720996	0.91157051240179
H	-4.67232173684126	1.81131590548963	2.21381727232472
H	-4.05391651111784	2.53087492815128	0.71205570916864
H	-3.39241707893716	-0.24978012930830	1.77320871825599
F	-2.09015443611083	1.27597987739901	1.76706341224226

13_a, Gas Phase

C	-5.48723039641856	1.09621703137242	0.35206539847604
C	-5.04271012007656	0.71201438985221	-1.05100151610493
C	-4.31380408259333	1.55374478184615	1.20631821111964
C	-3.12406678571702	0.60965147861424	1.14092442101734
C	-2.78166253510450	0.26037082732604	-0.29823606733899
N	-3.94313524399220	-0.22469697315158	-0.99495468402557
C	-4.17966301255361	-1.54390717431803	-1.28562300602054
O	-5.22403015279696	-1.89936215982468	-1.78023929232725
C	-3.08389072456078	-2.53766344971623	-0.98619616124067
H	-2.18254424743702	-2.32243645428998	-1.55983240952498
H	-3.46175115211308	-3.51528530885700	-1.26642331572387
H	-2.83434619996252	-2.53256793055064	0.07401540740986
H	-5.86384840496360	0.24807435078333	-1.58977926985903
H	-4.73155543740896	1.61599877441668	-1.58563357491934
H	-2.41105672958975	1.16210048961821	-0.79911453227833
H	-1.98664554334344	-0.47806457380826	-0.32011474270906
H	-6.23828054603825	1.88769317430616	0.29265817268203
F	-6.08714535957087	0.00393727881789	0.94652927715211
H	-4.63301111617705	1.68296801129073	2.23988068944045
H	-3.98529678766753	2.52933227921842	0.83571454039329
F	-3.41106077769828	-0.56479441068677	1.81496243510682
H	-2.25709464421608	1.05884556774069	1.62996001927499

13_a, Chloroform

C	-5.48544544698375	1.11121240980533	0.34615207371743
C	-5.04255521014306	0.71243910145350	-1.05051327890712
C	-4.31414642651423	1.55663999265763	1.20424117382757
C	-3.12915056297792	0.61038171441667	1.13981749620937
C	-2.78477629965872	0.24989724495902	-0.29395143193540
N	-3.95634987746436	-0.24332518083007	-0.97899542994757
C	-4.18263869352537	-1.55056187213966	-1.26200769749597
O	-5.24536588921576	-1.92467475535287	-1.73358021129346
C	-3.07257692772227	-2.53471685075737	-0.99867078530059
H	-2.18234888024718	-2.28753943463576	-1.57592402498215
H	-3.43175427989606	-3.51608222915909	-1.29085544985158
H	-2.80634874853715	-2.54408337422067	0.05733182601119
H	-5.87057496193273	0.27199529539375	-1.59620539886597
H	-4.70490161431229	1.60734148666708	-1.58049184295911
H	-2.42543463323457	1.14892191379267	-0.80295596368048
H	-1.98733348225821	-0.48475540855275	-0.31474384561158
H	-6.23260397589832	1.90302921968817	0.28309663303975
F	-6.10341119055571	0.01382878079247	0.94238241154771
H	-4.63389561404567	1.69227497244301	2.23690597217721
H	-3.98006820721133	2.52922591455371	0.83313162781643
F	-3.43089060706716	-0.56915927331876	1.82042850918172
H	-2.26125847059811	1.04988033234399	1.63128763730263

13_a, DMSO

C	-5.48475473370795	1.11706191380792	0.34432301110334
C	-5.04304600567259	0.71190460710204	-1.04993936233232
C	-4.31381517490316	1.55762687132464	1.20363080482410
C	-3.13121054312712	0.60971651386231	1.13954666663387
C	-2.78603629701167	0.24686036880755	-0.29268279669226
N	-3.96101977567689	-0.24948892068040	-0.97326094043457
C	-4.18327335582119	-1.55277356098911	-1.25440055075803
O	-5.25161075685763	-1.93394702117231	-1.71864828863287
C	-3.06830838898470	-2.53337674518343	-1.00270297021704
H	-2.18158989798629	-2.27567413522238	-1.58054361579482
H	-3.42092216665633	-3.51582001697182	-1.29959317272644
H	-2.79636151624846	-2.54779417942856	0.05175838666157
H	-5.87312462562798	0.27758164755174	-1.59679854634699
H	-4.69754312745592	1.60326366561726	-1.57972059486633
H	-2.43104977902428	1.14548531079788	-0.80400080608182
H	-1.98735148187762	-0.48605183079302	-0.31391408257711
H	-6.22930210957885	1.91035904754238	0.27929897137937
F	-6.11057656853267	0.01949368461459	0.94191948708815
H	-4.63372882366725	1.69607830109215	2.23590480616827
H	-3.97692008954864	2.52889605121507	0.83229250946334
F	-3.43942004206858	-0.57208536087300	1.82069978815261
H	-2.26286473996417	1.04485378797848	1.63271129598599

14_a, Gas Phase

C	-5.59058644526492	1.15956992338351	0.07159048110107
C	-4.67137902078739	0.93331765375704	-1.11586528920859
C	-4.79527377986688	1.40613022189840	1.34330512979352
C	-3.69853477919098	0.37709537654197	1.56089573008196
C	-2.88309293753663	0.14957639124633	0.29412501876972
N	-3.73186488950946	-0.13229009203769	-0.83328834434016
C	-3.85041781210439	-1.29851651824306	-1.55018076602118
O	-4.65987288269030	-1.35812483285288	-2.45034977468956
C	-2.94990289181359	-2.51579174748094	-1.27014092720254
C	-1.50236557347328	-2.19515311647928	-1.65887602909536
C	-3.44269427786374	-3.64027717779768	-2.18712063433513
C	-3.06715500959852	-3.03179738482594	0.17024979428441
H	-5.24803634223704	0.66299538688692	-1.99386934556656
H	-4.12475186199884	1.86125250662101	-1.32051806988406
H	-2.31533158603585	1.06711687054189	0.09180810006844
H	-2.16708873643387	-0.64325930030452	0.45568158947878
H	-6.24773813043777	2.00820299137294	-0.13235512113254
F	-6.39348970964445	0.04775029447512	0.23501356852296
H	-5.45947603212692	1.42770415057163	2.20661059200612
H	-4.32485638115175	2.39004365203999	1.25958984864365
F	-4.25936761251216	-0.82131673386688	1.96149257084461
H	-3.03123036856369	0.70222008788647	2.36241947843804
H	-4.10965637719727	-3.05219108090422	0.48820124545948
H	-2.51431296264571	-2.45286906641520	0.90408995161647
H	-2.68146397603158	-4.05144916358340	0.20455814612719
H	-3.41761593832777	-3.34114583585600	-3.23225446709871
H	-4.47028273143108	-3.91068213809539	-1.94760651426597
H	-2.80748091882701	-4.51601017850613	-2.04937489817820
H	-1.45184408918683	-1.91227188530560	-2.71157777148563
H	-0.88782691093216	-3.08625984657043	-1.52078535718643
H	-1.06092903457811	-1.38975940809798	-1.07390793554580

14_a, Chloroform

C	-5.57242262571252	1.19449595987565	0.05243500824526
C	-4.64929196531326	0.94519815070515	-1.12435740685732
C	-4.78822443710809	1.41567934674377	1.33225631605013
C	-3.70916482103125	0.37227530299450	1.55233347980214
C	-2.88229862326083	0.13665540490642	0.29708972377802
N	-3.73453711924033	-0.14460811195166	-0.83481457817275
C	-3.89399269149669	-1.32150871852651	-1.50177860996582
O	-4.76578560162437	-1.41308797398017	-2.35274844347092
C	-2.96161154862207	-2.52188794534348	-1.26009316979553
C	-1.52617781430352	-2.16064555545789	-1.65961821085784
C	-3.43231269669914	-3.64827905808280	-2.18592080585339
C	-3.04809630195388	-3.06288361365067	0.17363273463156
H	-5.22379291966696	0.70411102791032	-2.01082413790729
H	-4.07282045506768	1.85626796753159	-1.31093159569623
H	-2.31340284150429	1.04970067615172	0.09225306652892
H	-2.17090259947463	-0.65720707650762	0.46809866710260
H	-6.21108883442215	2.05322926348342	-0.15696469624340
F	-6.40952383643317	0.09102637564560	0.20247157065716
H	-5.45924439563549	1.44800927447129	2.19002889771887
H	-4.30060991251132	2.39103205421946	1.25717711769203
F	-4.30113393769563	-0.83018335889589	1.93125913088186
H	-3.04949478979032	0.67266012158199	2.36687506948743
H	-4.08922844599265	-3.15966922800981	0.48374822453037
H	-2.53786999473818	-2.45465972023815	0.91391466884858
H	-2.59394896556872	-4.05445300071357	0.19398845443220
H	-3.41726492012801	-3.33965160453761	-3.22928584775794
H	-4.44845515714630	-3.95510440547246	-1.94034556188499
H	-2.76974999786506	-4.50537024621526	-2.06168235020367
H	-1.49505463468191	-1.85384014621123	-2.70666009469759
H	-0.89367703767939	-3.04288169839281	-1.54843829567751
H	-1.09474007763212	-1.36060946403330	-1.06153832534491

14_a, DMSO

C	-5.56601481712195	1.20602276777555	0.04464401114047
C	-4.64077311802943	0.94871560073356	-1.12769667261819
C	-4.78650980607527	1.41816113963138	1.32775917249015
C	-3.71339291237522	0.37013142574938	1.54914397937621
C	-2.88144735019794	0.13277941981808	0.29839904578917
N	-3.73475219030437	-0.14880822742065	-0.83493946612262
C	-3.90891812212021	-1.32952680709310	-1.48305771639171

O	-4.80383626915902	-1.43286452862940	-2.31342124993888
C	-2.96561841797354	-2.52388220684914	-1.25614647943347
C	-1.53474478894396	-2.14908947271964	-1.65992207230084
C	-3.42979838136758	-3.64981037861932	-2.18563910185249
C	-3.04034455374889	-3.07353986683311	0.17509214231314
H	-5.21263207722768	0.71712880022958	-2.01807646130189
H	-4.05417126802982	1.85389293082568	-1.30766007515884
H	-2.31237812325714	1.04462994342293	0.09323151066529
H	-2.17143931483395	-0.66125728419263	0.47285556147022
H	-6.19774728091618	2.06868266022018	-0.16670050022937
F	-6.41550346656760	0.10596095765739	0.18898530005228
H	-5.46024895945336	1.45403693556537	2.18328677999901
H	-4.29326170513554	2.39065958659642	1.25621378490749
F	-4.31595732946651	-0.83391517136034	1.91909227024122
H	-3.05724502906568	0.66199239189394	2.36879817983264
H	-4.07943033639951	-3.19424498558786	0.48446157186442
H	-2.54254674793916	-2.45719420338900	0.91693187633271
H	-2.56510828320511	-4.05529091142773	0.19010283368411
H	-3.41963320388706	-3.33656577471777	-3.22797976874250
H	-4.44147817639460	-3.96920712984074	-1.93745761146384
H	-2.75801042455597	-4.50052395763964	-2.06795225272201
H	-1.51092952558050	-1.83330954874448	-2.70455848465783
H	-0.89635177321565	-3.02826764007394	-1.55905213364905
H	-1.10569624745156	-1.35168646498095	-1.05717797357498

14_e, Gas Phase

C	-5.42637897504943	0.57037204429924	0.92313335933030
C	-5.40662458602652	0.59649742942250	-0.60165473097382
C	-4.20836565825466	1.26590036984110	1.50506931665498
C	-2.92379259421523	0.73141510784467	0.89341216586333
C	-3.00008562896183	0.76760831481603	-0.62458898714571
N	-4.16660883386424	0.02615650302644	-1.07294531197108
C	-3.96265134098476	-1.23384250611207	-1.58856970265013
O	-2.83786616141506	-1.68484996106224	-1.62157745974133
C	-5.12284039381334	-2.05170223752976	-2.18504675128365
C	-5.86761990473597	-1.25768251400610	-3.26316166300794
C	-6.05786421430940	-2.57581954994861	-1.08725039429039
C	-4.50137549799863	-3.27557333650262	-2.86714173706989
H	-6.26481906187315	0.06557227925876	-0.98871010135861
H	-5.47702933503585	1.63686942788197	-0.93452918982154
H	-3.09552110905663	1.80834477271876	-0.94738898877417
H	-2.10939441046316	0.33245098293144	-1.06391634284986
F	-6.57118675273890	1.21026206904182	1.36000893994275
H	-5.48082374542433	-0.46909859785056	1.26164853454006
H	-4.19074954179904	1.15997113739957	2.58936555939795
H	-4.27720853697341	2.33110064273786	1.26768273056050
H	-2.71985138261704	-0.29251263784891	1.21829342766357
F	-1.86832236238507	1.51947008520370	1.31207292938794
H	-6.61720331766790	-1.90146057741200	-3.72596417765186
H	-5.17104677383588	-0.94545724007576	-4.04271021738304
H	-6.37454947044838	-0.36946859060440	-2.89455630701998
H	-6.76325170209233	-3.27951270746225	-1.53207436304030
H	-6.63886577811810	-1.80504838471812	-0.58571481494359
H	-5.48375820185569	-3.11663055945162	-0.33282124757361
H	-5.29458943203666	-3.87110891003824	-3.32137724059314
H	-3.96107735965928	-3.89396829811195	-2.15329402080852
H	-3.79697793629015	-2.97907455768865	-3.64236321338923

14_e, Chloroform

C	-5.43218014777942	0.58451370015283	0.92362405067375
C	-5.39230006854059	0.57120228239015	-0.59964212576939
C	-4.22704794218037	1.29842231303107	1.50741929408191
C	-2.94032970579456	0.73699405140473	0.93110356101543
C	-2.98589523045360	0.73620811415234	-0.58785509126864
N	-4.14516099322652	-0.02081961810417	-1.03188564352223
C	-3.94497226165750	-1.23255180256956	-1.63042082327847
O	-2.80700601973660	-1.65740849490886	-1.74959787469970
C	-5.11565728585496	-2.05290069071177	-2.19754341935137
C	-5.88449066486231	-1.25448631874466	-3.25715040639999
C	-6.02397237805345	-2.57914597736259	-1.07815677016058
C	-4.51840140704742	-3.27680404634115	-2.90048214429792
H	-6.24735488247563	0.03574761477142	-0.98426919737427
H	-5.44593453846136	1.60154790450933	-0.96359831668033
H	-3.07990366896508	1.76723844429435	-0.93976026600247
H	-2.08609181927513	0.29523890128360	-1.00020731729100
F	-6.58721989202458	1.24582432532888	1.32244790221663
H	-5.50039577089820	-0.44076868103693	1.29598595515980
H	-4.22752399888639	1.21863568060052	2.59394012432629
H	-4.28889504334299	2.35500577314632	1.23257807786831
H	-2.74590629458377	-0.27424784308223	1.29514448119493
F	-1.87887496622784	1.53409040382950	1.34395562712518
H	-6.64666404658611	-1.89850608478839	-3.69850391207716
H	-5.20573415892968	-0.94507080932898	-4.05376996103651
H	-6.38022215856390	-0.36602271115579	-2.87499285250035
H	-6.73870532645746	-3.28069985192741	-1.51114939998571
H	-6.59348346766728	-1.81004547275585	-0.56242672358955
H	-5.43252391344136	-3.12048329638369	-0.33751346963379
H	-5.33019410787536	-3.86645267150527	-3.32832874520324
H	-3.96300484307720	-3.90274838858489	-2.20399037185024
H	-3.84225299707337	-2.98232674960284	-3.70162424168933

14_e, DMSO

C	-5.43370115470315	0.59081900099285	0.92453543575242
C	-5.38824438383016	0.56234369097348	-0.59803838153081
C	-4.23224947178264	1.31131374172393	1.50710072667253
C	-2.94561253835451	0.73966519373092	0.94301326620839
C	-2.98184839483081	0.72453539033492	-0.57594155562357
N	-4.13921397475622	-0.03694420914338	-1.01845853041708
C	-3.94098854849493	-1.23337894095033	-1.64058226916562
O	-2.79981794145241	-1.65061800984790	-1.78368592057622
C	-5.11367801720281	-2.05388757718217	-2.20151113733480
C	-5.88975753646575	-1.25258299409446	-3.25415918453010
C	-6.01379902203514	-2.58308590352142	-1.07691206290454
C	-4.52287247276087	-3.27612394176268	-2.91262894728744
H	-6.24283089835635	0.02457592612308	-0.97970766040865
H	-5.43683356619261	1.58856242375966	-0.97366852281735
H	-3.07408920468889	1.75180114668117	-0.93887265174440
H	-2.07921588884662	0.28044225196251	-0.97806250496664
F	-6.59130258703235	1.25893463729292	1.31067937126166
H	-5.50589073513944	-0.42929585557989	1.30894071648435
H	-4.23846122072196	1.24190685337116	2.59426376064703
H	-4.29147840425060	2.36442301229045	1.21866173426847
H	-2.75504417775628	-0.26701813937626	1.32028498438049
F	-1.88132038576810	1.53925063773060	1.35172324746814
H	-6.65420938832726	-1.89665847870773	-3.69139383281636
H	-5.21638107701798	-0.94059652679894	-4.05445380775744
H	-6.38375014480697	-0.36589551301052	-2.86610489805140
H	-6.73321346094667	-3.28107820325577	-1.50782711249020
H	-6.57734086994568	-1.81481929799327	-0.55394194281282
H	-5.41730218515722	-3.12792649912827	-0.34290810942275
H	-5.33958936405396	-3.86429771508220	-3.33285551053170
H	-3.96196953625278	-3.90470340708863	-2.22264322867133
H	-3.85629344806890	-2.98048269444381	-3.72151547128229

15_e, Gas Phase

C	-5.14799601480612	1.78425389676159	0.75578229175338
C	-4.92265546624293	1.09047421794244	-0.58063333102458
C	-3.83470627055468	2.16610588201696	1.41244871226651
C	-2.92411823406077	0.95785505354798	1.52449071681901
C	-2.74283448005669	0.28104653795045	0.17588259016800
N	-4.03797179566092	-0.03801716904653	-0.38981787016068
C	-4.16985327403127	-1.23224847833475	-1.05235215454510
O	-3.32779268003211	-2.09437616373918	-1.02623439273816
C	-5.64037005933267	-2.48611150008073	-2.51566768458136
C	-7.01049193559678	-2.14826824446017	-3.08186094194878
C	-5.71994379461762	-3.73777379167913	-1.65495894139755
C	-4.62728123296658	-2.63147181906977	-3.64171860922006
H	-5.86765033376104	0.75314478495274	-0.99097892686323
H	-4.47850326620638	1.81666214098256	-1.27324876339360
H	-2.19496268182305	0.96704752963619	-0.48236352759664
H	-2.16894230669341	-0.63421841092400	0.27719104622135
F	-5.89311186708619	2.92654604225409	0.53115238146420
H	-5.73417996616349	1.13257028041456	1.40893373062863
H	-4.01152812120037	2.60499417238868	2.39398384258005
H	-3.33864909607457	2.91340469082538	0.78706043814154
H	-3.31579899759848	0.23695043928030	2.24676737847739
F	-1.68384215248633	1.36654546643418	1.97712826861885
H	-7.35366415844066	-2.95884670287788	-3.72392530777364
H	-6.96375730067597	-1.23236811708222	-3.67056674207869
H	-7.73213882120105	-2.01132871960760	-2.27685842786509

H	-6.10145077140943	-4.55957478996391	-2.26216210636315
H	-6.40837769096757	-3.58048403691986	-0.82403854508991
H	-4.74414470866800	-4.01235385960217	-1.26467712283694
H	-4.96726995303128	-3.40903210774845	-4.32648210114355
H	-3.64715379348831	-2.90396198506548	-3.26031799761588
H	-4.54909881422133	-1.69739523068629	-4.19931220258921
O	-5.32982996084390	-1.32116000849995	-1.70407570031312

15_e, Chloroform

C	-5.14842729985777	1.77241958952297	0.75191822743740
C	-4.91828206203577	1.08404423294679	-0.58537992094636
C	-3.84121109664693	2.16979587575171	1.40843246191223
C	-2.93242618601731	0.96285332169476	1.52548138031107
C	-2.73552845782805	0.28791157408430	0.17883115699591
N	-4.02618580238959	-0.04156453568455	-0.39358803392084
C	-4.15942787379301	-1.23358715784948	-1.04690470199182
O	-3.31412195179806	-2.10153101211814	-1.01668437655057
C	-5.63761751803173	-2.48991326577644	-2.51486679387642
C	-7.00747352900120	-2.13696073357752	-3.07200290903837
C	-5.72478213983126	-3.74378660510751	-1.65950311800908
C	-4.63160143956537	-2.63323435425010	-3.64637845929930
H	-5.86209449131980	0.74303050781808	-0.99470010129974
H	-4.47156048709371	1.80858729411372	-1.27636457583748
H	-2.19440471237027	0.97731452080147	-0.47995902902894
H	-2.15389699509344	-0.62065446008427	0.29098923472488
F	-5.90362533997124	2.91621588647326	0.52017521093126
H	-5.73493825657206	1.12409430552468	1.40566802727853
H	-4.02470966096583	2.60642109819457	2.38969513885013
H	-3.34741376630570	2.91405105766424	0.77754375782452
H	-3.32072980120105	0.24418546127430	2.24964640999601
F	-1.68729680247634	1.37757147617100	1.98423599419335
H	-7.35778534467363	-2.94143768637353	-3.71782340092658
H	-6.95525263492080	-1.21744181695327	-3.65499916713689
H	-7.72430223309080	-2.00118916427308	-2.26212933824110
H	-6.12542261264694	-4.55513189064080	-2.26828699887989
H	-6.40139734288789	-3.58061985580568	-0.81987177668663
H	-4.74902761754851	-4.03745615633379	-1.28288771169985
H	-4.98462642772734	-3.40123025658370	-4.33523385885139
H	-3.65190459574006	-2.92087204822866	-3.27468451788956
H	-4.54749478070426	-1.69430813677744	-4.19503133079170
O	-5.31510073989421	-1.32896706561787	-1.69676687955276

15_e, DMSO

C	-5.14924070424420	1.76767031758633	0.74984137064095
C	-4.91569965312490	1.08340348392266	-0.58874989383266
C	-3.84470195125116	2.16915458045861	1.40850643629482
C	-2.93549155690365	0.96317085167330	1.52565177730149
C	-2.73207265646996	0.29195090873309	0.17825109244631
N	-4.02035734350089	-0.04075053873737	-0.39803798005120
C	-4.15465269395582	-1.23284860530096	-1.04640978077667
O	-3.30799501895158	-2.10266721191020	-1.01400168988960
C	-5.63655049425283	-2.49099915740349	-2.51397355708436
C	-7.00624095510195	-2.13276044668284	-3.06794567120923
C	-5.72689899558813	-3.74490374066845	-1.65940721014615
C	-4.63346279316219	-2.63537672625148	-3.64760367820133
H	-5.85826997541393	0.74103365005407	-0.99956502896970
H	-4.46808652276143	1.80901809134923	-1.27750897013446
H	-2.19338123060579	0.98452614070499	-0.47877740959341
H	-2.14725622234928	-0.61424942186077	0.29181056493299
F	-5.90783407742499	2.91161314628869	0.51741302931460
H	-5.73589047610079	1.11845827147278	1.40195073008119
H	-4.03157444308766	2.60302993011126	2.39033278036008
H	-3.35146962807295	2.91423667335435	0.77810649962706
H	-3.32340881672884	0.24329029198007	2.24826791062115
F	-1.68983973510865	1.37939013612085	1.98849491241266
H	-7.35990988731768	-2.93559734810623	-3.71392509635387
H	-6.95179992814191	-1.21275067185312	-3.65003207616853
H	-7.72095913836736	-1.99589718401867	-2.25632036734329
H	-6.13392312797958	-4.55309315553512	-2.26807770346199
H	-6.39987853532561	-3.57901961519165	-0.81736548887003
H	-4.75157570422240	-4.04513349405769	-1.28671311164928
H	-4.99144915506563	-3.40056154123861	-4.33696535948572
H	-3.65388497513782	-2.92812134234798	-3.27942560806643
H	-4.54716330486846	-1.69551532811236	-4.19431194925777
O	-5.30915029941191	-1.33109094453330	-1.69493947348765

15_a, Gas Phase

C	-5.71651007623119	1.06605061363484	0.44324547035441
C	-5.22182553057889	1.09680207748727	-0.99602050888137
C	-4.66152128642139	1.59182527532806	1.40559730119452
C	-3.29027384974914	0.97073015208148	1.18591271252669
C	-2.90304110974324	0.98610027857964	-0.28416695937348
N	-3.94944739719963	0.41970797975013	-1.10261496172707
C	-3.97406784344504	-0.90384666259404	-1.46577439863474
O	-4.93727221959025	-1.43434185906683	-1.95578324082323
C	-2.72030502811723	-2.93957548544846	-1.14401613068460
C	-1.28691153112217	-3.16037358340969	-0.68879448529343
C	-2.96198671320346	-3.59524428797463	-2.49431076036085
C	-3.69693807042844	-3.42496367640839	-0.08279169063548
H	-5.93698970762439	0.60622453358433	-1.64943664183108
H	-5.10665954317911	2.13817570853449	-1.31198458126236
H	-2.74359240507901	2.02481039454070	-0.59184787830896
H	-1.97891616724546	0.43910353983591	-0.43488408985041
H	-6.63371756518259	1.65301196291799	0.53161383840785
F	-6.02899800409439	-0.23800793098445	0.77817882711669
H	-4.98246489576176	1.42994818719444	2.43424698506023
H	-4.57048379795413	2.67109182434400	1.25207012975522
F	-3.29559790656623	-0.34140768398420	1.62870753066901
H	-2.53718991094000	1.50343039675523	1.77100627937825
H	-4.72696703103658	-3.29083647952557	-0.40482299800652
H	-3.54306504374383	-2.87027946576560	0.84327894583782
H	-3.52359688771925	-4.48378193815388	0.10966759113931
H	-2.29379040250485	-3.16993076291031	-3.24383983837330
H	-3.99016374751574	-3.45725526374984	-2.81630320261606
H	-2.75312986903021	-4.66272538631309	-2.41300155397439
H	-0.59023062326669	-2.77144540972922	-1.43166498453848
H	-1.09846372829483	-4.22482344550180	-0.55443850250365
H	-1.11298153680073	-2.64888447560468	0.25814182231108
O	-2.80013057063013	-1.49607912744384	-1.24720002607160

15_a, Chloroform

C	-5.73127007747186	1.11728706749896	0.42129072130123
C	-5.20869114455871	1.11600988117461	-1.00550415308961
C	-4.68450616809518	1.61855101041600	1.40115110513465
C	-3.32376353003725	0.97150480467455	1.21020498536079
C	-2.90574725540431	0.96620714475426	-0.24934308678195
N	-3.95201606641528	0.40041640576850	-1.07542191762106
C	-3.98065899867513	-0.91160674935222	-1.44037121592450
O	-4.95155052165813	-1.44705539256439	-1.92908496146477
C	-2.71108880729230	-2.95366798368774	-1.15075418496012
C	-1.26410391500199	-3.16276791094711	-0.73516542999420
C	-2.97843353563427	-3.60887407834099	-2.49618796005711
C	-3.65313463336964	-3.45048938164445	-0.06486731008990
H	-5.92727855684350	0.64918924301868	-1.67126409322869
H	-5.05112978056859	2.14950057736992	-1.32336846641951
H	-2.73345079247501	1.99849950832739	-0.56510555065567
H	-1.98475631664223	0.40912515276230	-0.37682148201113
H	-6.63498753536182	1.72417846817974	0.48559733984143
F	-6.08925484741852	-0.18657278491123	0.75984605723796
H	-5.02882245179113	1.47445328231141	2.42495197303630
H	-4.56640265008665	2.69358552062952	1.24200935111942
F	-3.36905409236949	-0.34915051830623	1.65698308130628
H	-2.57099338371854	1.48441691809251	1.80950205069591
H	-4.69350336255661	-3.31687795344030	-0.35307473867947
H	-3.46896526254360	-2.90449491089795	0.86090799695368
H	-3.47171248885959	-4.51063354093040	0.11121501358828
H	-2.34665096627871	-3.16036919090074	-3.26389310536247
H	-4.01995448699638	-3.50343736500997	-2.78588856758198
H	-2.73428932444090	-4.66948014375382	-2.42628852449068
H	-0.59134241725016	-2.77077931064749	-1.49849318885576
H	-1.06607511634464	-4.22595603564486	-0.60510695389976
H	-1.06669169504969	-2.64852325330696	0.20582419009707
O	-2.80694981879016	-1.50897848069150	-1.24550897450467

15_a, DMSO

C	-5.73772717958485	1.13607538163459	0.40782999564915
C	-5.20026803820933	1.12507321220198	-1.01233627592316
C	-4.69681241660393	1.62488223506773	1.39872156533446

C	-3.34044143938808	0.96690888995620	1.22115345692429
C	-2.90643024724765	0.95816539858664	-0.23298885892232
N	-3.95022906979369	0.39517180416051	-1.06642393989145
C	-3.98085108472826	-0.91323642586151	-1.43158375480423
O	-4.95468312533004	-1.44988737437103	-1.91993563018003
C	-2.70651677568674	-2.95757149936825	-1.15196125661622
C	-1.25294149010478	-3.16322911223806	-0.75841627849288
C	-2.99078175225301	-3.61444765662412	-2.49313533057032
C	-3.63083390803277	-3.45606037801889	-0.05211490000317
H	-5.91751940083912	0.66843255022135	-1.68627045092696
H	-5.02532302610841	2.15588074672567	-1.32819749374707
H	-2.72801048267155	1.98899085906458	-0.54824383958735
H	-1.98673469824880	0.39719610457583	-0.35094853921678
H	-6.63551724643009	1.75171869277283	0.46152541848550
F	-6.11597858422010	-0.16714149544962	0.74359833367239
H	-5.05310418435660	1.48536801853335	2.41907358351282
H	-4.56696384004734	2.69853762314367	1.24092430210924
F	-3.40419592385068	-0.35765610757737	1.66454129186861
H	-2.58932514115403	1.46976539037605	1.82993994777334
H	-4.67570150992799	-3.31311288563741	-0.31966943182304
H	-3.42475576008287	-2.91859113874888	0.87397568753641
H	-3.45389548431410	-4.51880692051987	0.11209802176901
H	-2.38025519496866	-3.15557357451915	-3.27192043903952
H	-4.03904400338046	-3.52524170102704	-2.76326021965497
H	-2.72894104238723	-4.67113702195940	-2.42940043228186
H	-0.59282171599258	-2.77556047990534	-1.53492587529408
H	-1.05261179126374	-4.22561414392752	-0.62559903197822
H	-1.04034777977052	-2.64336177886496	0.17616484430457
O	-2.80766666302199	-1.51272721240257	-1.24424446998615

16_a, Gas Phase

C	-3.56702829499626	2.01289453560131	0.12776159093122
C	-3.63215350812117	0.88206348520035	-0.89285393685084
C	-2.30213873880407	2.86341707587739	-0.03441811218287
H	-3.58214910825392	1.56508217602221	1.12481026963057
H	-4.45702215804605	2.63773599599894	0.03459882671239
C	-1.06707365600074	1.95440900508794	-0.04981517950737
H	-2.22646820344517	3.51428798867775	0.83985272366083
C	-2.36901267423036	3.76253446956981	-1.26660738265974
C	-1.20550221829453	0.80568605386954	-1.04492109301736
H	-0.91341891234683	1.52709031378763	0.94409303718119
H	-0.17509258231234	2.53627153315398	-0.29275689192724
N	-2.43606409165628	0.06551432677891	-0.79525415703201
H	-3.67820974182776	1.28081112634706	-1.90977289736732
H	-4.52065111690119	0.28008015741713	-0.74590794843008
C	-2.35582145532307	-1.18820675672157	-0.31538406582611
O	-1.33058735052324	-1.79600420833147	-0.13192217879471
C	-3.68799783867129	-1.91225579418614	0.01937953780638
F	-4.48775937601054	-2.00274680656326	-1.05040493965252
F	-4.36415368246968	-1.26795719730721	0.98188751852484
F	-3.44316836649966	-3.13399159066943	0.44716400850531
H	-1.24245392321994	1.18460761641526	-2.06969674462882
H	-0.36978502026810	0.11732824222286	-0.96740932657555
H	-1.49748326977945	4.41655920287309	-1.31266280312225
H	-3.25936492170352	4.39203604838896	-1.23700559224729
H	-2.39980979029482	3.19273300048890	-2.19621426313067

16_a, Benzene

C	-3.56564143935819	2.01524936751410	0.12905696230919
C	-3.63105507324044	0.88160000841327	-0.88774903405529
C	-2.30156288813573	2.86621788191403	-0.03560923359439
H	-3.58071760964132	1.57251010174963	1.12844658684820
H	-4.45638966185325	2.63784895006769	0.03034943757008
C	-1.06632706609096	1.95775148884444	-0.04885320389106
H	-2.22594535588560	3.51772607314696	0.83803707710516
C	-2.36911092673486	3.76373152962668	-1.26903601325343
C	-1.20414085192897	0.80670687576417	-1.04081945942403
H	-0.91161309349022	1.53485422446386	0.94682259124308
H	-0.17491018670187	2.53782181413654	-0.29741528931919
N	-2.43357934496220	0.06296845482060	-0.78252808151483
H	-3.66868952738243	1.27508508124770	-1.90628174012495
H	-4.52148979685822	0.28242430346796	-0.74208444601272
C	-2.35854124312367	-1.18796015928571	-0.31680106542665
O	-1.33387055763594	-1.80902312983408	-0.13859597586975
C	-3.69111530363099	-1.91256387454458	0.01596290193245
F	-4.48890778528022	-2.00708253327675	-1.05389652059458
F	-4.36992929493717	-1.27680206255244	0.97979796222154
F	-3.44266320250061	-3.13774749118593	0.44185880284576
H	-1.25289871768944	1.18252078732158	-2.06559264322023
H	-0.36382653127456	0.12408264456948	-0.96828527025285
H	-1.49620297633708	4.41621997374638	-1.31537901585058
H	-3.25927541795987	4.39367003348564	-1.23714513904131
H	-2.40196614736617	3.19216965637870	-2.19772019062966

16_a, Dichloromethane

C	-3.56485832967399	2.01599747738180	0.12931024426769
C	-3.63017973410609	0.88128623420596	-0.88579067447429
C	-2.30129438178700	2.86729939806973	-0.03603828092519
H	-3.58067262878309	1.57617091919492	1.13002885282218
H	-4.45598789185478	2.63709625591621	0.02660797394163
C	-1.06583520881399	1.95938613538424	-0.04841750834198
H	-2.22584381962022	3.51849689927428	0.83771298439233
C	-2.36907729629132	3.76464208100696	-1.26959585780430
C	-1.20315006571053	0.80755144543006	-1.03920388910491
H	-0.91022485769878	1.53908340602935	0.94827757900703
H	-0.17501754703033	2.53853302396702	-0.30065413100627
N	-2.43165265858745	0.06094544968391	-0.77485596909794
H	-3.66096931515141	1.27160335005360	-1.90516974983584
H	-4.52211014178309	0.28400742907256	-0.74227384281936
C	-2.36073201673299	-1.18703564557751	-0.31764917055802
O	-1.33620890638054	-1.81818109201013	-0.14250821776879
C	-3.69356121623749	-1.91134207449394	0.01484402890133
F	-4.48973116339257	-2.01119631068021	-1.05451171005568
F	-4.37362968867186	-1.28050617755241	0.97889568573348
F	-3.44187976431639	-3.13864905490187	0.44165346524571
H	-1.26086489664881	1.18204973378213	-2.06347032195061
H	-0.35927521452284	0.12928559296080	-0.97140208267154
H	-1.49553925938300	4.41654070982995	-1.31507834696790
H	-3.25953051531727	4.39422542482800	-1.23605025508540
H	-2.40254348150412	3.19268938914455	-2.19812080584338

16_a, Chloroform

C	-3.56487136714766	2.01842572548214	0.13102157680486
C	-3.63022812069024	0.88186375629277	-0.88216317179748
C	-2.30139231212276	2.86942659356052	-0.03639049689546
H	-3.58016602283439	1.57992708000889	1.13232749566615
H	-4.45588963673692	2.63976829590628	0.02838992481780
C	-1.06600128732065	1.96144137288230	-0.04668577679285
H	-2.22593274544672	3.52339188246663	0.83533771146613
C	-2.36942786822464	3.76292530429741	-1.27269720992439
C	-1.20286449467484	0.80723898977850	-1.03494839397322
H	-0.91080826413511	1.54304986981832	0.95085856825139
H	-0.17512279963291	2.54053932848496	-0.29897088410159
N	-2.43210185325972	0.06260579014409	-0.77171516519551
H	-3.66426756363796	1.27118430510286	-1.90204813727618
H	-4.52168288318589	0.28453633404257	-0.73572619551057
C	-2.36039718273052	-1.18948293626180	-0.32112187252553
O	-1.33625396021513	-1.82067921163001	-0.15335268244308
C	-3.69306444666254	-1.91387712170022	0.01194003491565
F	-4.49278412709540	-2.00652864295637	-1.05581931122853
F	-4.36973630253316	-1.28537057924913	0.98058227921927
F	-3.44242321299341	-3.14274142753102	0.43232275125847
H	-1.25542799673993	1.17946951999203	-2.06055230306177
H	-0.36053934415855	0.12745496033550	-0.96221382893854
H	-1.49590375865653	4.41458629866111	-1.32130570472554
H	-3.25952887925819	4.39311078042376	-1.24145191231864
H	-2.40355356990622	3.18771373164786	-2.19907729569087

16_a, DMSO

C	-3.56439240420561	2.01944177769525	0.13158200911494
C	-3.62967245703115	0.88187591294486	-0.88013136663397
C	-2.30128773482037	2.87060169023890	-0.03666534320597
H	-3.58021296479092	1.58316486753606	1.13388697808014
H	-4.45561301441150	2.63982966514483	0.02621884085534
C	-1.06575986948807	1.96299950744984	-0.04584948652208
H	-2.22596710141596	3.52498689582652	0.83466897466993
C	-2.36954428696167	3.76296504062208	-1.27377739159007
C	-1.20206836708428	0.80776588775701	-1.03281117517744

H	-0.91012376920319	1.54679084458817	0.95258363495660
H	-0.17527187740467	2.54147345206745	-0.30055169106581
N	-2.43090090927968	0.06167746234243	-0.76594823254703
H	-3.65957840193254	1.26886888003894	-1.90066298524789
H	-4.52201013441765	0.28577312112770	-0.73453674967649
C	-2.36182337445312	-1.18931028697073	-0.32292333936702
O	-1.33772504725998	-1.82749621869551	-0.15929673096102
C	-3.69456634188685	-1.91332842611874	0.01051481883628
F	-4.49427251768878	-2.00817502697896	-1.05623757959477
F	-4.37078451236335	-1.28848740704364	0.98047051354745
F	-3.44185746853046	-3.14401268307815	0.43023997283447
H	-1.25929285606981	1.17857669242460	-2.05838276441605
H	-0.35789926774160	0.13035566891971	-0.96176930514976
H	-1.49544626934648	4.41392329921149	-1.32287641848492
H	-3.25961246590915	4.39323212211886	-1.24183931744998
H	-2.40468658630313	3.18648726083096	-2.19936586580489

16_a, Water

C	-3.56448350855462	2.01653421869555	0.12969536057828
C	-3.62978408901895	0.88124381626636	-0.88454606864856
C	-2.30116844324841	2.86796539400418	-0.03626463063145
H	-3.58050735477015	1.57797377057859	1.13098372863142
H	-4.45578890083509	2.63701936129991	0.02550006065173
C	-1.06558111024801	1.96030588387119	-0.04804180920976
H	-2.22576365860315	3.51938218877933	0.83727644952382
C	-2.36921070050970	3.76473423235896	-1.27022561767188
C	-1.20270142489570	0.80784562025694	-1.03801309844979
H	-0.90961503018040	1.54130567029882	0.94916782592589
H	-0.17499735172522	2.53905668952779	-0.30180310532582
N	-2.43087731132922	0.06034214585753	-0.77146545211226
H	-3.65817498283020	1.27021201508955	-1.90430123022580
H	-4.52213310572952	0.28453951662669	-0.74166903089556
C	-2.36155664267947	-1.18705140850199	-0.31833351778471
O	-1.33705989310303	-1.82189414923810	-0.14475299454208
C	-3.69455588308652	-1.91108809329694	0.01392545722082
F	-4.49034770663397	-2.01192408591275	-1.05516338402475
F	-4.37462395387699	-1.28242808731762	0.97864435187509
F	-3.44188693295254	-3.13948462724168	0.44025715654077
H	-1.26351849653669	1.18158122582880	-2.06221253554767
H	-0.35770315760833	0.13097235842358	-0.97162655695583
H	-1.49543999430444	4.41636074338037	-1.31588306305349
H	-3.25974082090295	4.39421515102604	-1.23616728939043
H	-2.40314954583671	3.19226044933885	-2.19844100647802

16_e, Gas Phase

C	-3.56501536990004	2.18352386646870	-0.03372073070302
C	-3.63661428590143	1.00175369208867	-0.99229568407128
C	-2.31035694854937	3.02191613831862	-0.27268196908164
H	-3.55714805870312	1.80192972971603	0.99239114060810
H	-4.45892797217822	2.79947877930763	-0.14765282007624
C	-1.07318886549771	2.12595526619787	-0.22681238962813
C	-2.21069541581957	4.16763115060523	0.72329321333371
H	-2.38003651125949	3.44292448994482	-1.28351339877032
C	-1.20941959547943	0.92841238644275	-1.16076101469103
H	-0.92424941095040	1.76427230753682	0.79535751607225
H	-0.18336144079447	2.69823521823369	-0.49901219301027
N	-2.43592436167336	0.19592064617816	-0.87437406752713
H	-3.69192906267126	1.36305115818596	-2.02408088597433
H	-4.52024448719518	0.40199347318857	-0.81172561219578
C	-2.34981647753884	-1.05033991892771	-0.37552957835348
O	-1.32272690407527	-1.65579329430769	-0.19642552338110
C	-3.67730457719073	-1.76707874394876	-0.00722732620288
F	-4.48948869748409	-1.88413422182569	-1.06509927073632
F	-4.34266029219909	-1.10091269221906	0.94765339944143
F	-3.42559407944563	-2.97796116047333	0.44630131090882
H	-1.33814376394633	4.79115390877494	0.52454779238516
H	-2.12180662310623	3.78127830982974	1.74097700471228
H	-3.09685177279442	4.80248975892310	0.68290992637460
H	-1.25693809330009	1.27122792319490	-2.19910745314396
H	-0.37146693234623	0.24561182856604	-1.06179138628944

16_e, Benzene

C	-3.56187300632126	2.18981037064067	-0.02368064839269
C	-3.63610337474103	1.00041052581197	-0.97172498315431
C	-2.30942688701423	3.02759481345112	-0.27411756707324
H	-3.55060612395104	1.81909596723491	1.00649458146126
H	-4.45733631183721	2.80187931628705	-0.14414280276530
C	-1.07223500889693	2.13275008574589	-0.21978920040944
C	-2.20922342020251	4.18363261551819	0.71014220248909
H	-2.38100154937024	3.43754830052546	-1.28898143093474
C	-1.20722583551904	0.92742040548483	-1.14314702523657
H	-0.92262839792871	1.78216564776668	0.80619802093007
H	-0.18292681333806	2.70099930944144	-0.50125234910043
N	-2.43347382197940	0.19347704919684	-0.84686488115722
H	-3.68900265841411	1.35137877015512	-2.00641954106308
H	-4.52075693827244	0.40455791949041	-0.78447166188074
C	-2.35442270618426	-1.05893359512654	-0.38524346643423
O	-1.32915259078308	-1.68406834322627	-0.22706313790603
C	-3.68250422683361	-1.77912090114933	-0.02492559745346
F	-4.49648360546158	-1.88300092463042	-1.08172649541847
F	-4.34622150268185	-1.13500491495254	0.94359794362686
F	-3.42776790636538	-3.00074247213744	0.40757414555324
H	-1.33652973921730	4.80464230817530	0.50308302562806
H	-2.11974346699506	3.80741215696921	1.73179096053528
H	-3.09606467786643	4.81738350716626	0.66237995122229
H	-1.26187773326694	1.26078363995249	-2.18356889362461
H	-0.36532169655831	0.25046844220868	-1.04252114944157

16_e, Dichloromethane

C	-3.56003657388536	2.19208759167786	-0.01849989163297
C	-3.63566866259034	0.99994375090152	-0.96231390794473
C	-2.30894925891804	3.02980989175520	-0.27446176282466
H	-3.54741839567238	1.82646770520645	1.01357391511177
H	-4.45637159501399	2.80176605753981	-0.14282889080028
C	-1.07156721703212	2.13586701181167	-0.21641096254737
C	-2.20852858189168	4.19062632545672	0.70431290542400
H	-2.38167122565000	3.43443417843685	-1.29102745916728
C	-1.20582705489016	0.92749425003581	-1.13545227713303
H	-0.92107768202188	1.79055553424442	0.81131188602110
H	-0.18290266041891	2.70208928411381	-0.50335161669562
N	-2.43169946496350	0.19153146830751	-0.83326580035409
H	-3.68490280121963	1.34604730005720	-1.99813955827409
H	-4.52130141301606	0.40631751102895	-0.77333601300719
C	-2.35748835870924	-1.06111554793901	-0.38912072076332
O	-1.33285094416687	-1.69829986667589	-0.23919855523586
C	-3.68594741338425	-1.78297308695976	-0.03324156779610
F	-4.49950791464490	-1.88345166278591	-1.08946810973531
F	-4.34968649788815	-1.15114231424547	0.94130291615682
F	-3.42817538125619	-3.01023080471413	0.39023996056499
H	-1.33586078234040	4.81040874120863	0.49278445351475
H	-2.11868578519781	3.81927367820965	1.72787179428597
H	-3.09595991540248	4.82344948649513	0.65264784112095
H	-1.26698252426671	1.25689213140472	-2.17619491563064
H	-0.36084189555895	0.25469138542827	-1.03611366265782

16_e, Chloroform

C	-3.56057088577882	2.19145213829180	-0.01998619209382
C	-3.63577897681021	1.00004499253050	-0.96497176683949
C	-2.30910016135113	3.02921324454101	-0.27439505426360
H	-3.54825283162159	1.82436165832901	1.01153867788689
H	-4.45667065800991	2.80182900828953	-0.14310461418940
C	-1.07176112986520	2.13501273844888	-0.21743978586809
C	-2.20872260514260	4.18864720495029	0.70597351560401
H	-2.38152691879644	3.43539178245147	-1.29047746851243
C	-1.20623887748362	0.92744630140067	-1.13763585085781
H	-0.92150395405538	1.78820892775472	0.80978569705663
H	-0.18291034661280	2.70183550578460	-0.50279958908224
N	-2.43222106830323	0.19212187934877	-0.83715501012945
H	-3.68619093549605	1.34753672203999	-2.00049140381803
H	-4.52111021860719	0.40575535803370	-0.77643641670452
C	-2.35655397406717	-1.06058654188364	-0.38794329032844
O	-1.33178951436749	-1.69415173814896	-0.23563511082659
C	-3.68493324202088	-1.78197918268975	-0.03088518021279
F	-4.49856765801638	-1.88327642652617	-1.08738957588647
F	-4.34878176004610	-1.14650916085876	0.94185406524995
F	-3.42817886788106	-3.00756818603841	0.39516784005699
H	-1.33606545927223	4.80881046729829	0.49572932059380
H	-2.11891614566211	3.81585864470426	1.72896267613173

H	-3.09598904212602	4.82174134753687	0.65552336109922
H	-1.26540901816136	1.25791610330073	-2.17833446238918
H	-0.36216575044503	0.25342721111058	-1.03783438167686

16_e, DMSO

C	-3.55954421204508	2.19256646678962	-0.01715550375969
C	-3.63555488382117	0.99989327702087	-0.96004818453838
C	-2.30881995759249	3.03027112845338	-0.27446961106660
H	-3.54670888193204	1.82821913224194	1.01539656296879
H	-4.45609067346742	2.80158135392787	-0.14264854967076
C	-1.07138364323464	2.13659100303645	-0.21545969043018
C	-2.20839321695779	4.19229581197629	0.70291433589961
H	-2.38180259750537	3.43351927920356	-1.29144025527928
C	-1.20540374561201	0.92759774254507	-1.13360032366187
H	-0.92067392399024	1.79257149176371	0.81269660421139
H	-0.18291549346228	2.70227767600906	-0.50386867294187
N	-2.43117930125156	0.19093523067548	-0.82981637082612
H	-3.68342750116718	1.34481664467028	-1.99611122051358
H	-4.52151593471260	0.40686995127153	-0.77092839155115
C	-2.35839801314451	-1.06143571089620	-0.39022230942282
O	-1.33383855644450	-1.70221033380671	-0.24248846070088
C	-3.68691329571324	-1.78360639841152	-0.03524709775124
F	-4.50038842309652	-1.88378724540356	-1.09111318480133
F	-4.35047130048665	-1.15496422993556	0.94082456280422
F	-3.42809997553515	-3.01235365463656	0.38625383268477
H	-1.33572235024929	4.81174121745575	0.49021730978516
H	-2.11854554880433	3.82222007952529	1.72698579147606
H	-3.09599847226966	4.82483199284649	0.65009817574958
H	-1.26857838177954	1.25615135576288	-2.17430187336955
H	-0.35954171572476	0.25594673791459	-1.03484747529426

16_e, Water

C	-3.55949741963058	2.19260566168169	-0.01703249695069
C	-3.63554308492709	0.99988973043001	-0.95984632475284
C	-2.30880666347915	3.03030978891030	-0.27446074743881
H	-3.54664976184129	1.82837329667863	1.01556332340580
H	-4.45606222027394	2.80155819439107	-0.14264120337090
C	-1.07136386121909	2.13665623095896	-0.21537273261748
C	-2.20837944928046	4.19244269871036	0.70279269997370
H	-2.38181310932698	3.43343359487768	-1.29147223210826
C	-1.20536396330392	0.92761197185372	-1.13343783980236
H	-0.92062687227768	1.79275494710175	0.81282258084381
H	-0.18291856494425	2.70229482832037	-0.50392663750871
N	-2.43112671709453	0.19087516642999	-0.82949503791745
H	-3.68327093853419	1.34470211499345	-1.99593062002526
H	-4.52153862732433	0.40692487712961	-0.77072526352836
C	-2.35848771190635	-1.06145000457789	-0.39031351640189
O	-1.33393080934126	-1.70257359606948	-0.24275827253929
C	-3.68701042767146	-1.78365044801765	-0.03542863525771
F	-4.50046486677788	-1.88382751287320	-1.09126341913233
F	-4.35056048273668	-1.15531597392381	0.94077920132761
F	-3.42808787488240	-3.01253995468836	0.38589935765709
H	-1.33570991109292	4.81185873639171	0.48998493360623
H	-2.11852946431540	3.82248252204957	1.72691087808374
H	-3.09600332973645	4.82494898896767	0.64987004706975
H	-1.26875388003374	1.25609561998762	-2.17412890069190
H	-0.35940998804798	0.25607852028624	-1.03476318192348

17_a, Gas Phase

C	-2.33810123019430	0.83990500615326	-0.79005925366117
C	-2.82498099044754	-0.53593802259353	-0.36510496031478
C	-0.91267360297886	1.11956938794360	-0.32428159219710
F	-3.19649215297155	1.78304480749171	-0.26593193552094
H	-2.39233624691045	0.91487973401831	-1.87896298168775
N	-2.67493342204320	-0.70036024338579	1.06303291671053
H	-2.24056591102708	-1.30342917627359	-0.88285638701439
H	-3.85928177452616	-0.65168236499708	-0.66427995544731
C	-0.75927245326865	0.83295905802613	1.16751351653918
C	-1.32282727746650	-0.52227450080005	1.56201413867428
F	-1.40300952950637	1.81065580503508	1.89669288650414
H	0.29951904765132	0.86632510013982	1.44034370021751
H	-0.72269302984966	2.18400908067917	-0.47444757020514
C	0.09768433450059	0.31589895674547	-1.14250318855323
H	-1.33793246751719	-0.61156663940202	2.64415708065998
H	-0.67890677590452	-1.31110195684931	1.15967506894724
C	-3.65554480336866	-0.99294443118950	1.94493223935836
O	-3.49334509377847	-1.12159123277670	3.12907916774585
C	-5.08477095078550	-1.22107780943112	1.38131585709727
F	-5.09439552042444	-2.23857865545114	0.50640789962455
F	-5.55926444545200	-0.14088650627353	0.75657039163249
F	-5.90813124092436	-1.52262720351114	2.36347803318380
H	-0.03462631091154	0.49813890191686	-2.20903639494817
H	0.00472048944257	-0.75863966000067	-0.98002237418599
H	1.11710135663250	0.59830256478578	-0.88127630315920

17_a, Benzene

C	-2.33919550739111	0.82436272800042	-0.79077515831416
C	-2.81776817930132	-0.55349036905626	-0.36757345530037
C	-0.91953453232620	1.11838114520916	-0.32062946736662
F	-3.21122127213435	1.76302812359435	-0.26205167641557
H	-2.39578126368100	0.90270734006761	-1.87784065075567
N	-2.67210434274946	-0.71158822365597	1.06466991565413
H	-2.22018887554322	-1.31596280778554	-0.87469055132486
H	-3.84794785419074	-0.68183899047343	-0.67500254134582
C	-0.76186883872036	0.82352398953881	1.16748040745086
C	-1.31442643016953	-0.53608951114833	1.55671635750236
F	-1.42208533164564	1.79391510982753	1.90604381783198
H	0.29381175107716	0.86912167427708	1.44454186981482
H	-0.73807618687340	2.18470503756639	-0.46731382825719
C	0.09914276989175	0.32885189481704	-1.14227378385149
H	-1.31408499198866	-0.63737140888130	2.63722992508802
H	-0.67574397559048	-1.31735739641539	1.13522302983605
C	-3.65260316027491	-0.98166425523816	1.94145485287798
O	-3.50156816698548	-1.09473639792828	3.13395243021225
C	-5.08383409969430	-1.20283403348153	1.37961940555724
F	-5.11079327591648	-2.22854886315148	0.51811792748204
F	-5.55147577356241	-0.12350791487090	0.74868201720179
F	-5.90994598349069	-1.48535324733172	2.36935693857502
H	-0.03079036289584	0.52561901106612	-2.20633176117394
H	0.00854129639969	-0.74762243145973	-0.99251502680519
H	1.11448258775694	0.61473979691354	-0.86964099417368

17_a, Dichloromethane

C	-2.34272798542789	0.80691081454185	-0.78809457600668
C	-2.80786094110555	-0.57691591038633	-0.37226509611553
C	-0.92868834832502	1.11621455389639	-0.31401897734894
F	-3.22926476517438	1.73457592979142	-0.24885375596812
H	-2.40276487120057	0.89377995820516	-1.87328161006874
N	-2.66668904886953	-0.73155290351949	1.06298434845775
H	-2.19538404551921	-1.33101809472333	-0.87160225724323
H	-3.83335882872697	-0.72114527317550	-0.68786634425254
C	-0.76430507319531	0.80913610117560	1.16976696552308
C	-1.30490058128249	-0.55680898451266	1.54938416560502
F	-1.43871206880186	1.76856736812411	1.92136614319263
H	0.28936214013187	0.86547137648634	1.44845947853402
H	-0.75866014024950	2.18535490730775	-0.45352193034340
C	0.09965558246979	0.34669202692166	-1.14276887413022
H	-1.29124227677099	-0.67320769761534	2.62782182943513
H	-0.66976801070513	-1.32884596874940	1.10852719154450
C	-3.65136760884888	-0.95890170695836	1.93774557936484
O	-3.51334759732289	-1.03461908838507	3.13924657599700
C	-5.08300992742403	-1.18112006513058	1.37648549180537
F	-5.12255002425524	-2.22040565449005	0.53565585441844
F	-5.54398868233381	-0.10799964521784	0.73021252981503
F	-5.91285271010678	-1.43872167978602	2.37358806031189
H	-0.03549969220973	0.55276817292883	-2.20423336368786
H	0.02264017049844	-0.73197712498612	-1.00219748314035
H	1.11022533475565	0.64475858825698	-0.86608994569911

17_a, Chloroform

C	-2.34146401216443	0.81247992747693	-0.78930031863465
C	-2.81129629704040	-0.56919331415182	-0.37077356638558
C	-0.92569904050071	1.11697013499734	-0.31629073139199
F	-3.22339075960194	1.74406549449776	-0.25397100781547
H	-2.40016297144870	0.89625636188496	-1.87511423357811
N	-2.66845130331547	-0.72509029768245	1.06348591837322
H	-2.20401770476325	-1.32626342291856	-0.87271260207284

H	-3.83844264291909	-0.70788340032642	-0.68356784014477
C	-0.76375973643660	0.81397099499333	1.16892987995059
C	-1.30787808111406	-0.55010275631786	1.55156807151756
F	-1.43423577327171	1.77680184727756	1.91612360880989
H	0.29042602708151	0.86729692978914	1.44744337683208
H	-0.75198607359644	2.18519619971463	-0.45829096910533
C	0.09981914758323	0.34083693854607	-1.14232786466939
H	-1.29837829354554	-0.66169950746416	2.63069426103757
H	-0.67154392660726	-1.32489986932870	1.11661154618443
C	-3.65158208877936	-0.96708307681613	1.93899629914298
O	-3.50913435929607	-1.05611030788460	3.13763074804823
C	-5.08332219618565	-1.18796320197619	1.37774704910067
F	-5.11966048578795	-2.22232351486683	0.52949455723401
F	-5.54601646127522	-0.11208745664648	0.73730456050881
F	-5.91186018924666	-1.45389468682057	2.37254609404186
H	-0.03404415765609	0.54277697051710	-2.20480556269202
H	0.01921840445365	-0.73703444148505	-0.99748854728988
H	1.11180297543420	0.63596745499100	-0.86748272700187

17_a, DMSO

C	-2.34400554115532	0.80175446056332	-0.78652523576289
C	-2.80446634776211	-0.58429036882936	-0.37372022752780
C	-0.93146795976755	1.11535849617260	-0.31178894982593
F	-3.23482335796850	1.72531804378840	-0.24271062964920
H	-2.40572695255298	0.89203666589622	-1.87104604702097
N	-2.66505794479048	-0.73775348071429	1.06252168427873
H	-2.18692267120182	-1.33540567380564	-0.87057025929356
H	-3.82830120310611	-0.73385539661447	-0.69213464556254
C	-0.76455329335296	0.80443927613594	1.17060754095444
C	-1.30227517302946	-0.56299076765759	1.54761069536500
F	-1.44208056729271	1.76094134272410	1.92665370053836
H	0.28870958949989	0.86306786245008	1.44925840660882
H	-0.76467093987402	2.18530313237877	-0.44899420498994
C	0.09932196907012	0.35201268142518	-1.14344273402736
H	-1.28489079835086	-0.68378569343466	2.62537828956947
H	-0.66837707260988	-1.33267990499585	1.10172166761183
C	-3.65129755220335	-0.95049466798738	1.93621958588882
O	-3.51789605777994	-1.01190236098043	3.14056128987064
C	-5.08262639823016	-1.17503205533866	1.37481776289466
F	-5.12465518875764	-2.21846342726542	0.54061280565801
F	-5.54272638697808	-0.10443312565829	0.72377373216669
F	-5.91351863899481	-1.42578844055468	2.37406271580352
H	-0.03773095068699	0.56174181254110	-2.20389149378034
H	0.02641531717084	-0.72746799502001	-1.00684771933115
H	1.10856412070485	0.65335958478105	-0.86567773043730

17_a, Water

C	-2.34409594198365	0.80121374375473	-0.78643443704453
C	-2.80418477818204	-0.58497631059377	-0.37380950540078
C	-0.93174405362692	1.11529106819802	-0.31157035929899
F	-3.23535000607236	1.72446579722485	-0.24238329749689
H	-2.40591233339291	0.89168496968808	-1.87091167401656
N	-2.66491614456652	-0.73824959650747	1.06251527856096
H	-2.18621367594310	-1.33586523024437	-0.87040829091796
H	-3.82788632597963	-0.73500298553298	-0.69243370955664
C	-0.76458218911434	0.80397406887780	1.17066525660020
C	-1.30200302325003	-0.56358442211171	1.54737245525826
F	-1.44240971630878	1.76020125137633	1.92714161239182
H	0.28863507664476	0.86286155909884	1.44934636154273
H	-0.76528579535657	2.18531258068304	-0.44853938064676
C	0.09932225760257	0.35257209812774	-1.14345355021031
H	-1.28417279523310	-0.68489243631788	2.62506401382501
H	-0.66829669192542	-1.33303184647829	1.10082563396203
C	-3.65128349248099	-0.94961553273534	1.93608739520331
O	-3.51835002550347	-1.00979236447626	3.14069698294766
C	-5.08258898529245	-1.17446278946973	1.37470709389754
F	-5.12483795283251	-2.21833470317347	0.54121775746967
F	-5.54266406714482	-0.10416816865208	0.72314344700584
F	-5.91358286276152	-1.42452870421205	2.37419181968511
H	-0.03771639633852	0.56286853786931	-2.20379198430692
H	0.02663190934449	-0.72700773407866	-1.00742786004834
H	1.10842800969781	0.65405714968534	-0.86536105940541

17_e, Gas Phase

C	-2.26227574036423	0.73881746422458	-0.88374538602330
C	-2.71886817123230	-0.64001984440490	-0.42819060672577
C	-0.87978385594319	1.09759657020430	-0.35248177318771
H	-2.99781133379667	1.48894928933516	-0.57647007632560
F	-2.22152685194332	0.74489253815171	-2.26510800889083
N	-2.62039708212977	-0.72651029730603	1.01914016950353
H	-2.06940102954547	-1.39497558459310	-0.87998495040706
H	-3.74467805535890	-0.82710190015501	-0.72995880398859
C	-0.79659745665011	0.84830530108440	1.14802961127201
C	-1.27794198894597	-0.54426866521646	1.52826380993611
H	-1.38293407354753	1.59455830635602	1.69447298309203
F	0.51834307439549	0.97370171975125	1.55106637574571
C	-0.50878974570637	2.53224269176418	-0.69471304668662
H	-0.17257403681320	0.40651044372348	-0.82557179096800
H	-1.22262432635485	3.22478685484644	-0.24295877199553
H	-0.51981553889700	2.68762318365756	-1.77182596268090
H	0.48360883142075	2.77310246318707	-0.31830828617348
H	-1.23576752866263	-0.67597247406742	2.60284025910968
H	-0.62043254117430	-1.28814227228927	1.06975627725122
C	-3.75382213113357	-0.84900572006565	1.74694358077529
O	-4.85990960759970	-0.92073866263760	1.28176358885690
C	-3.61005289994052	-0.90760182652256	3.29187422266751
F	-2.85254887566705	-1.94040900099485	3.67589171268722
F	-4.79535827571065	-1.03882788307808	3.84801823345862
F	-3.04950075869891	0.21331730504479	3.76669663969758

17_e, Benzene

C	-2.26084720095799	0.75156091251873	-0.88175959980705
C	-2.72937459302933	-0.62166321473303	-0.42233883034150
C	-0.87374909592096	1.09932487300713	-0.35799787857149
H	-2.98781783844220	1.51135522580060	-0.58159372178018
F	-2.22339060941896	0.74649444785699	-2.26783934438242
N	-2.63455368831228	-0.70102634219503	1.02697948792359
H	-2.08401954744643	-1.38462629447267	-0.86546183788806
H	-3.75485015146466	-0.80072884347253	-0.72863103935006
C	-0.80243400882603	0.86376451847437	1.14451521329583
C	-1.28943102923609	-0.52352296950997	1.53533527746133
H	-1.38286573579397	1.62009494453222	1.68149128684243
F	0.51536936672162	0.98278592113738	1.55474405667670
C	-0.48716837930757	2.52700692237184	-0.71267792112758
H	-0.17460952536198	0.39427835965391	-0.82198822245153
H	-1.19460409268987	3.23052718102836	-0.26829802016295
H	-0.49267411126497	2.67243333883729	-1.79146014426072
H	0.50794149146807	2.76056779458678	-0.33818933511017
H	-1.24708647769990	-0.64553850128472	2.61084594396351
H	-0.63892033141049	-1.27518568313383	1.08088916161124
C	-3.75495631348860	-0.86784404338163	1.75319762509612
O	-4.86528406129698	-0.97467723853928	1.29360221818023
C	-3.60916413839766	-0.93431171729347	3.29796935491277
F	-2.83786815242051	-1.95793575305056	3.67631591215435
F	-4.79604535902148	-1.09265808035914	3.85110917387309
F	-3.07305641698072	0.19035424162026	3.78668118324252

17_e, Dichloromethane

C	-2.26005138548997	0.76249982343587	-0.87984440965489
C	-2.73811171498634	-0.60603462540815	-0.41692334868301
C	-0.86898451329988	1.10037251678961	-0.36252187854228
H	-2.97949181217428	1.53007909022575	-0.58383997791678
F	-2.22675093609698	0.74908402564170	-2.26915846633516
N	-2.64590440193038	-0.68074294371312	1.03386169322434
H	-2.09658392545856	-1.37549866084474	-0.85358663257000
H	-3.76361872724621	-0.77812659318313	-0.72604953319148
C	-0.80739683207889	0.87760968640246	1.14177011324619
C	-1.29899187943100	-0.50511218027933	1.54201306872680
H	-1.38413589794308	1.64203012306822	1.66992090980118
F	0.51182278515175	0.99250653810201	1.55826538695740
C	-0.46832935935773	2.52130795097515	-0.72882073695414
H	-0.17723578642558	0.38299960396170	-0.81828956573705
H	-1.16969242163855	3.23496466772837	-0.29136494589560
H	-0.46944261990842	2.65733265490551	-1.80900388313102
H	0.52902920867441	2.74821477916856	-0.35580512313636
H	-1.25735149673583	-0.61806335602908	2.61839120499461
H	-0.65345765271645	-1.26327600952880	1.09226416122935
C	-3.75566320636958	-0.88208068773488	1.75814600558245

O	-4.86846959194319	-1.01527457463835	1.30200243522679
C	-3.60823999003564	-0.95800644796841	3.30257467131187
F	-2.82392298768760	-1.97318833449219	3.67409788773493
F	-4.79583285169419	-1.14208641987179	3.85250514001831
F	-3.09465200317779	0.16931937328708	3.80483582369358

17_e, Chloroform

C	-2.26020050948991	0.75896144835220	-0.88055835696760
C	-2.73539176412263	-0.61099806554081	-0.41873050461798
C	-0.87046661249648	1.10011696807900	-0.36105114346051
H	-2.98211753557322	1.52414371600487	-0.58363886323512
F	-2.22526217458836	0.74798579160207	-2.26891439996092
N	-2.64243385730826	-0.68698231240581	1.03160521010573
H	-2.09267702009015	-1.37851236757578	-0.85729933701288
H	-3.76088129025863	-0.78524266915066	-0.72703659234809
C	-0.80578538541038	0.87307000257962	1.14265495721440
C	-1.29600190044356	-0.51109567351499	1.53974638193695
H	-1.38347640507637	1.63496073342969	1.67379850439793
F	0.51309992584259	0.98907649976473	1.55709317910174
C	-0.47447818498366	2.52333482615431	-0.72345012976274
H	-0.17624133452954	0.38679009455609	-0.81949862863633
H	-1.17790775068838	3.23359116505414	-0.28368438309177
H	-0.47694745939779	2.66251481038117	-1.80318151245748
H	0.52214626832693	2.75246950496694	-0.34995555022938
H	-1.25403546070995	-0.62718544827639	2.61582142326292
H	-0.64906749806897	-1.26720964797198	1.08825331998970
C	-3.75546642124542	-0.87796755912934	1.75659590397747
O	-4.86752872207304	-1.00351002717176	1.29951035139400
C	-3.60848940170456	-0.95019032546156	3.30117318200070
F	-2.82876060801290	-1.96830317436304	3.67526176230198
F	-4.79594484929402	-1.12528636755346	3.85231398799116
F	-3.08714404860333	0.17629807719074	3.79861123810614

17_e, DMSO

C	-2.25991743443316	0.76615470704262	-0.87914916518403
C	-2.74101030271713	-0.60084768525336	-0.41499994980357
C	-0.86750611634193	1.10061645070899	-0.36400781769595
H	-2.97680043914724	1.53625268021481	-0.58418978316301
F	-2.22825312602849	0.75016884737544	-2.26940176990910
N	-2.64945941861099	-0.67437430352773	1.03620173566996
H	-2.10086307724385	-1.37237588859062	-0.84984440409957
H	-3.76661944960052	-0.77061473959300	-0.72475450462496
C	-0.80893241000089	0.88225416629344	1.14085802050337
C	-1.30208505929040	-0.49889121624747	1.54439758230426
H	-1.38451756061021	1.64933402109604	1.66602662480542
F	0.51063803867580	0.99586664689215	1.55926928145139
C	-0.46198340240035	2.51915731848967	-0.73429798513495
H	-0.17832001475925	0.37911729923257	-0.81704814444160
H	-1.16110239777563	3.23634199140911	-0.29912589332186
H	-0.46184480598768	2.65195863008873	-1.81493243110734
H	0.53617787420877	2.74367598939695	-0.36186125706869
H	-1.26088950236541	-0.60858853476831	2.62108972186617
H	-0.65794529092885	-1.25919715431096	1.09655208909397
C	-3.75581959196864	-0.88651074528459	1.75971104948957
O	-4.86928636643088	-1.02733503372262	1.30438463946361
C	-3.60800035757054	-0.96601696702718	3.30397721185447
F	-2.81839310726581	-1.97767951943016	3.67292661917302
F	-4.79561376017640	-1.16006524412814	3.85253318699311
F	-3.10311292123029	0.16242828364360	3.81112534288633

17_e, Water

C	-2.25990661543665	0.76647681419087	-0.87907795609433
C	-2.74125254367206	-0.60040116642493	-0.41483770210174
C	-0.86737661926818	1.10063782239035	-0.36413944022078
H	-2.97656800542397	1.53678482992239	-0.58419848137151
F	-2.22840070790796	0.75026875881314	-2.26942388283056
N	-2.64978163909962	-0.67381466506682	1.03640660364325
H	-2.10119971048229	-1.37210092942281	-0.84949264935733
H	-3.76685605568031	-0.76997741290036	-0.72467992807677
C	-0.80908464513914	0.88266718733744	1.14077735321576
C	-1.30236068642856	-0.49834956669058	1.54460380601619
H	-1.38457884720799	1.64997629198094	1.66567151161280
F	0.51052101324931	0.99617280238350	1.55937593955548
C	-0.46143350397754	2.51897008920827	-0.73477822196650
H	-0.17841897719578	0.37877443185442	-0.81693785904850
H	-1.16036467082776	3.23645876651007	-0.29981504571034
H	-0.46117012601199	2.65148608966034	-1.81545258313194
H	0.53679394510056	2.74328594625460	-0.36238385202653
H	-1.26120304394298	-0.60775525801992	2.62132268528610
H	-0.65835569758870	-1.25884459786242	1.09692030815375
C	-3.75581891532618	-0.88687870845576	1.75984282269324
O	-4.86936762353586	-1.02839932426249	1.30460970611822
C	-3.60795660372487	-0.96671650013582	3.30409257115482
F	-2.81797108532256	-1.97812536419977	3.67280945666930
F	-4.79558833369448	-1.16154834231113	3.85254103345760
F	-3.10376030145440	0.16178200524647	3.81168380436035

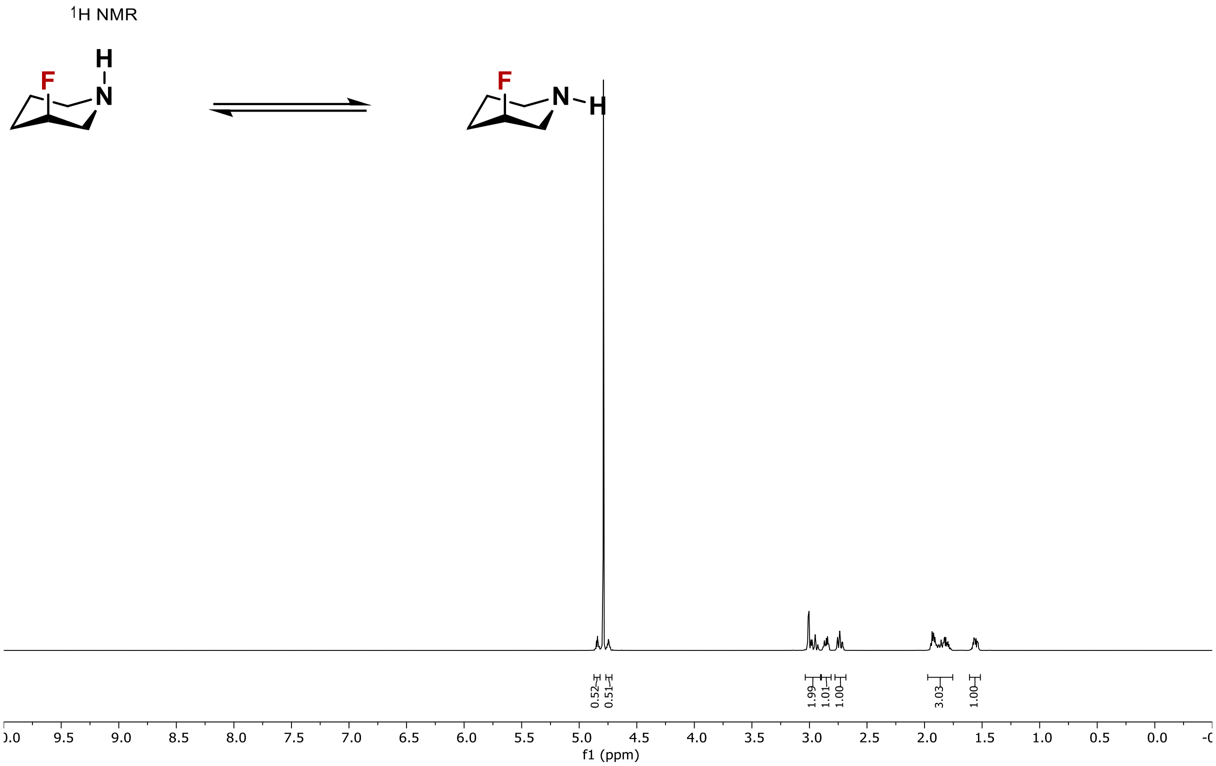
1H NMR
F
H
N
F
N
H
0.52
0.51
1.99
1.01
1.00
3.03
1.00
f1 (ppm)

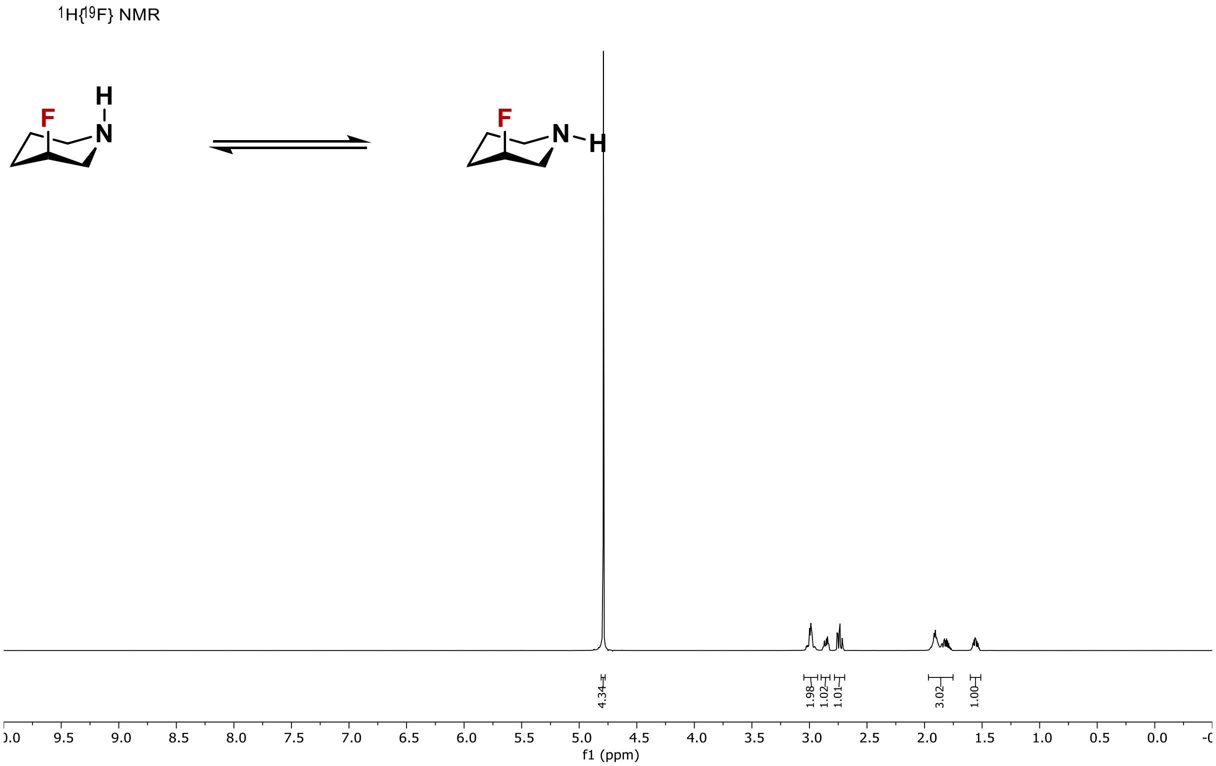
1H{19F} NMR
F
H
N
F
N
H
4.34
1.98
1.02
1.01
3.02
1.00
f1 (ppm)

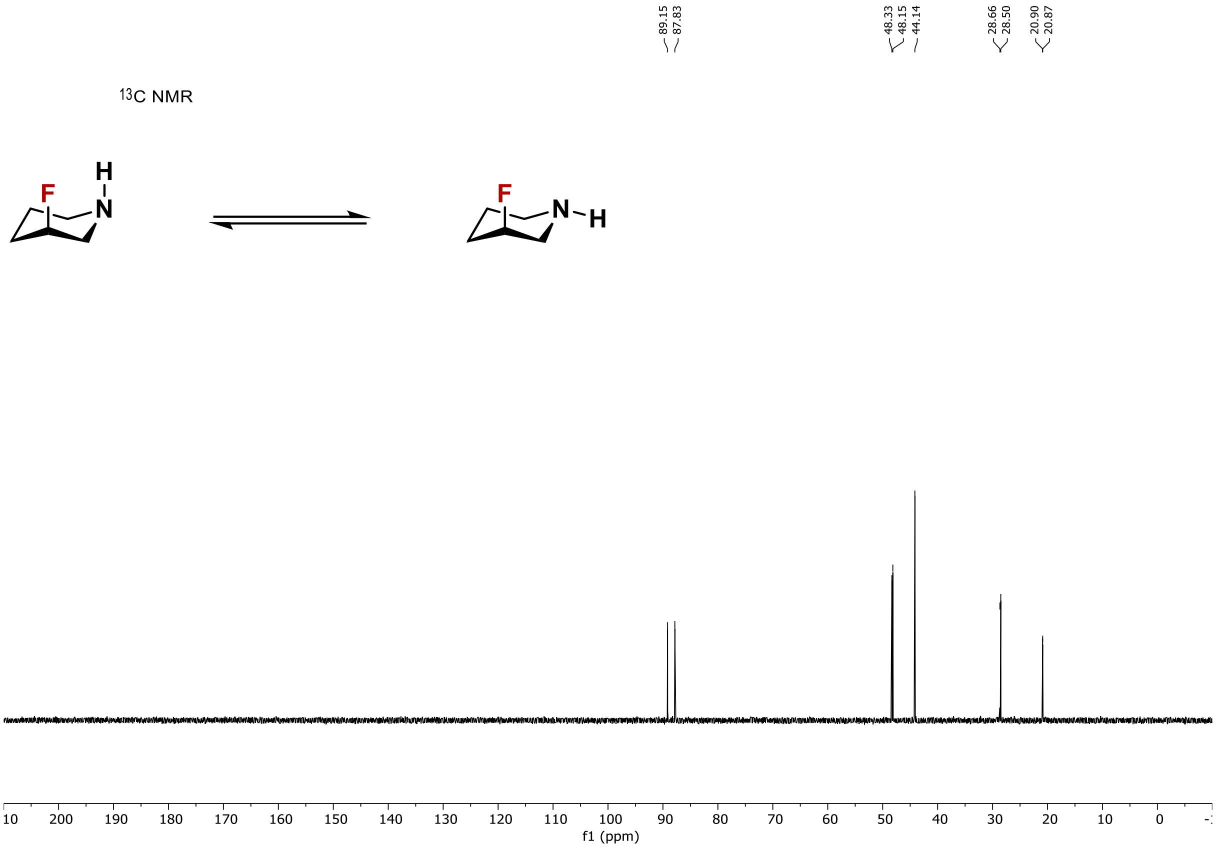
89.15
87.83
48.33
48.15
44.14
28.66
28.50
20.90
20.87
13C NMR
F
H
N
F
N
H
f1 (ppm)

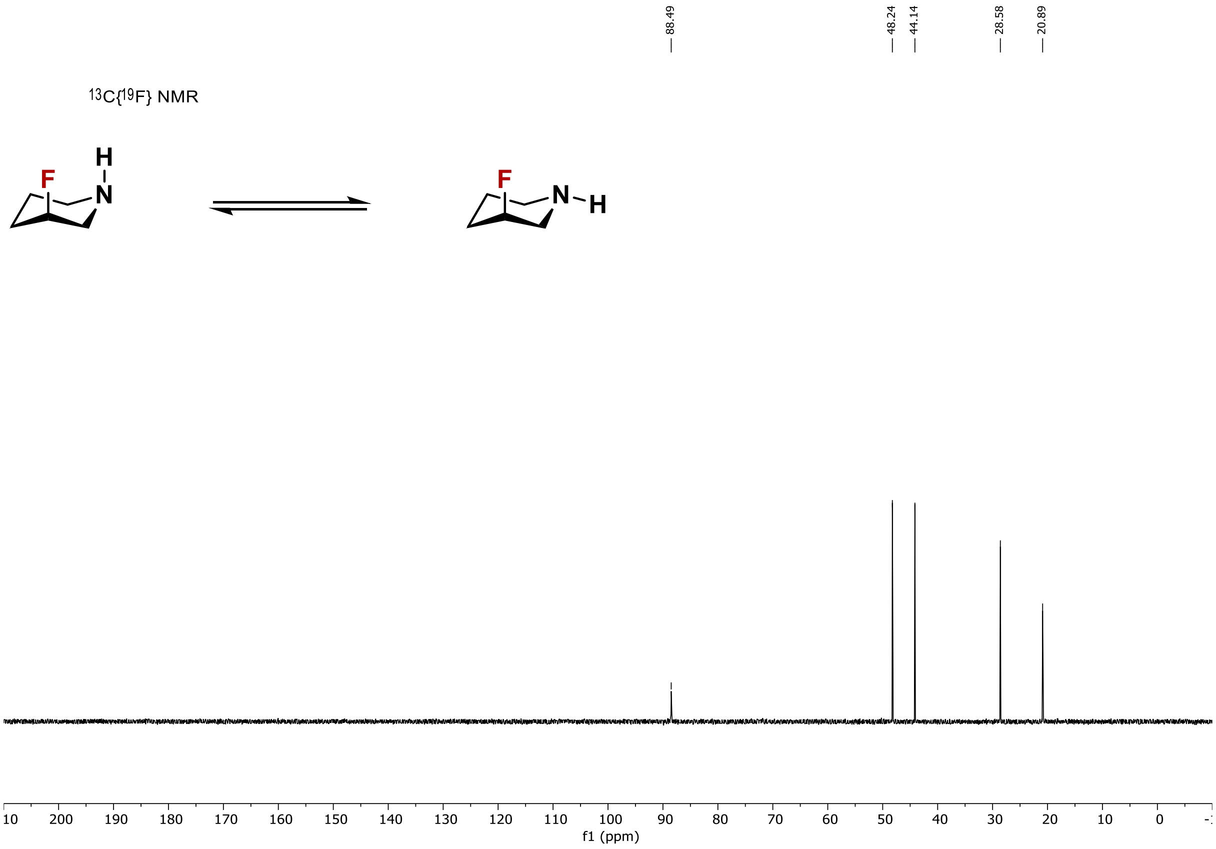
88.49
48.24
44.14
28.58
20.89
13C{19F} NMR
F
H
N
F
N
H
f1 (ppm)

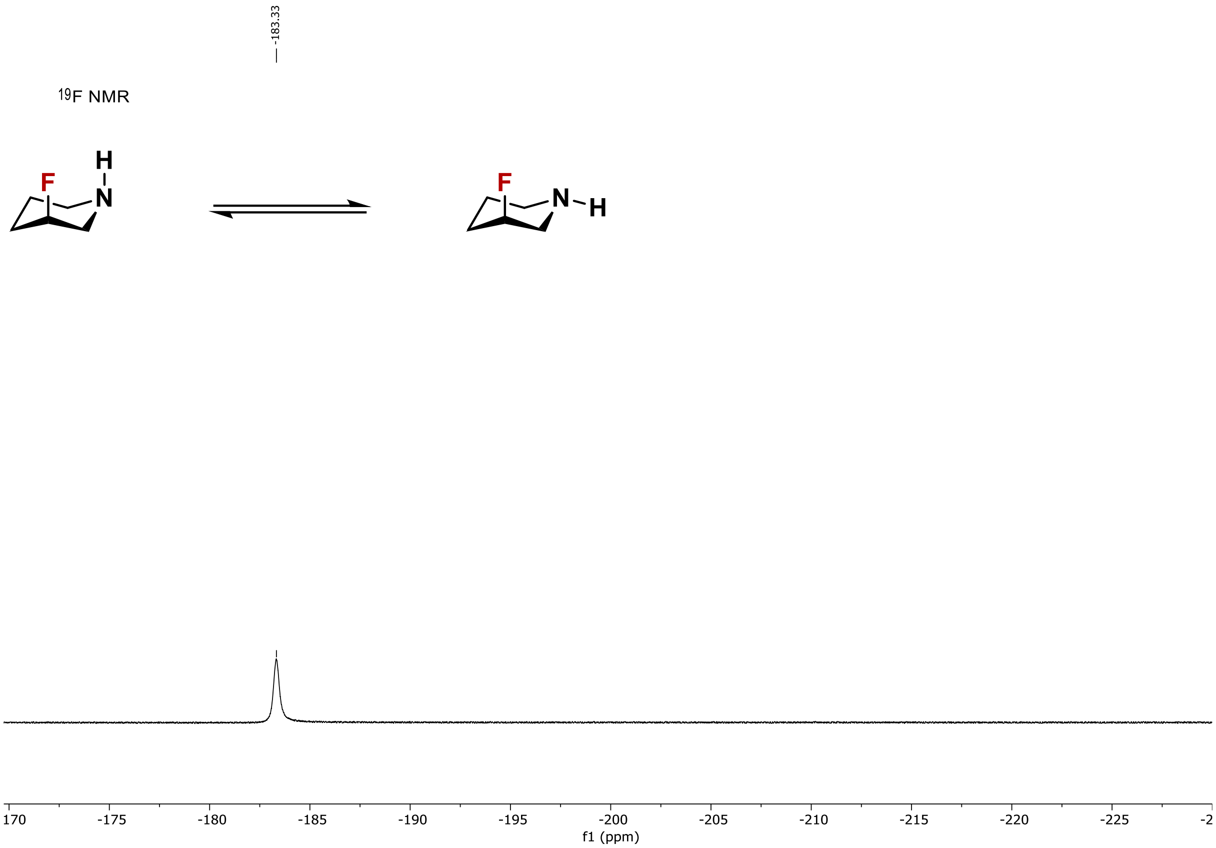

-183.33
19F NMR
F
H
N
F
N
H
170
-175
-180
-185
-190
-195
-200
-205
-210
-215
-220
-225
-2
f1 (ppm)

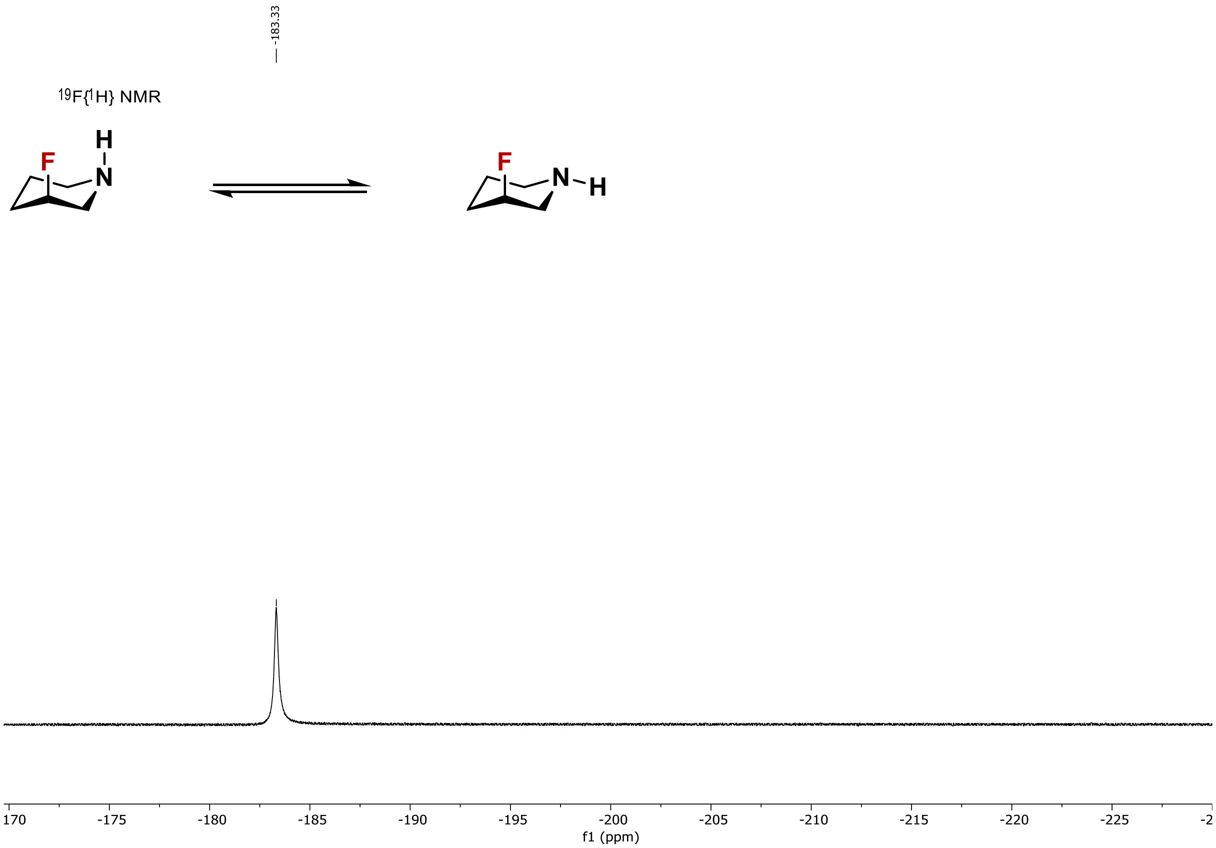

-183.33
19F{1H} NMR
F
H
N
F
N
H
170
-175
-180
-185
-190
-195
-200
-205
-210
-215
-220
-225
-2
f1 (ppm)

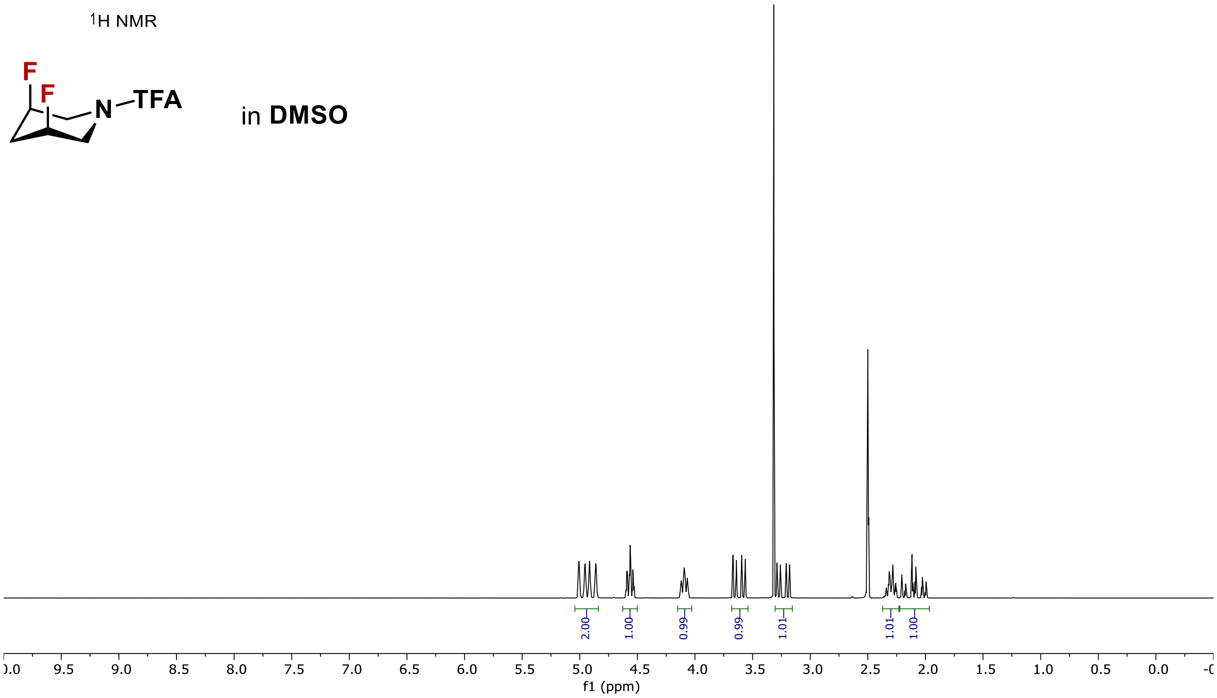
1H NMR
F
F
N–TFA
in DMSO
2.00
1.00
0.99
0.99
1.01
1.01
1.00
f1 (ppm)

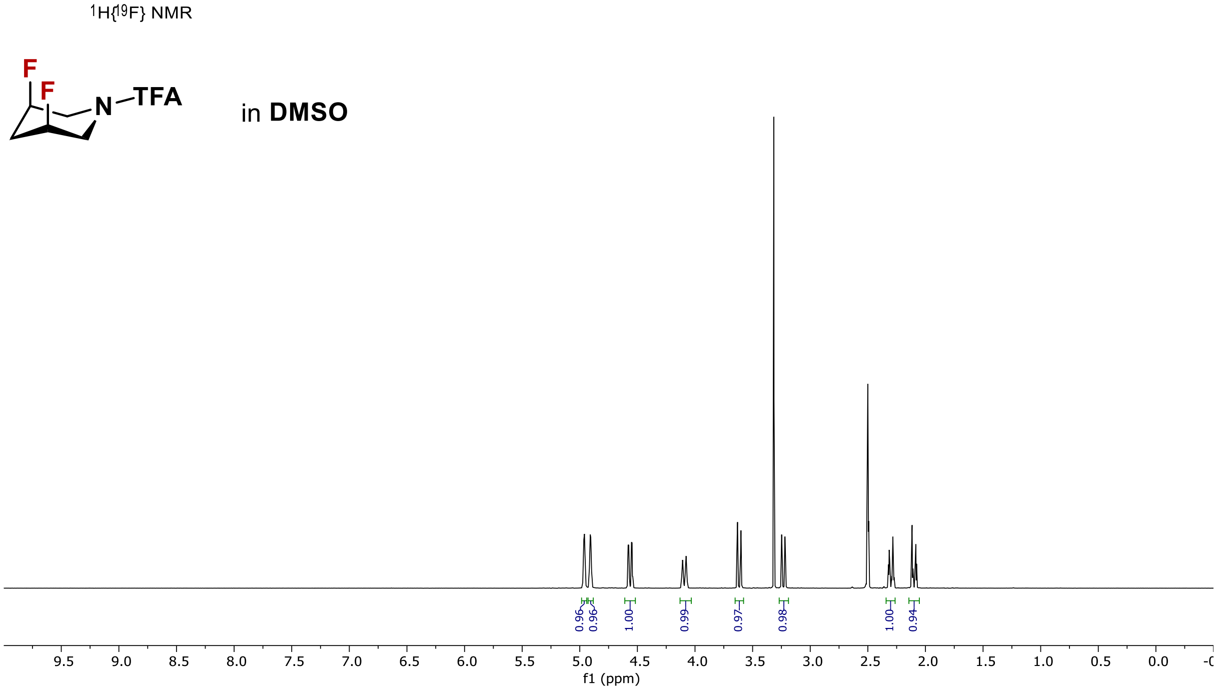
1H{19F} NMR
F
F
N–TFA
in DMSO
0.96
0.96
1.00
0.99
0.97
0.98
1.00
0.94
f1 (ppm)

^{1}H NMR

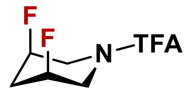

in CD_2Cl_2

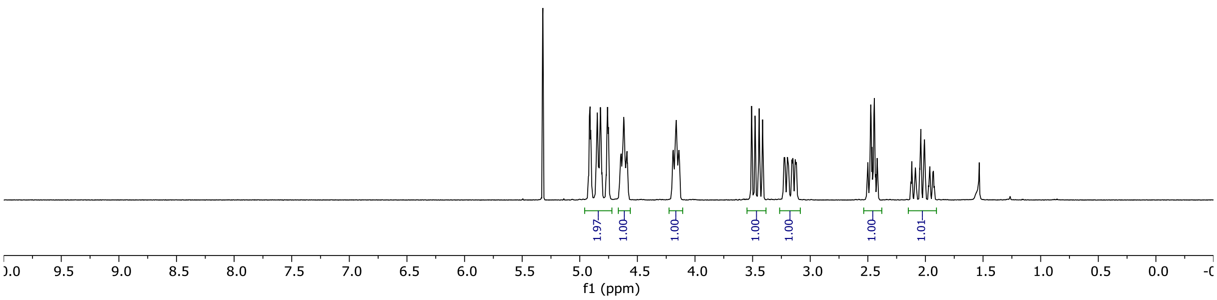

$^{1}H\{^{19}F\}$ NMR

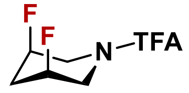

in CD_2Cl_2

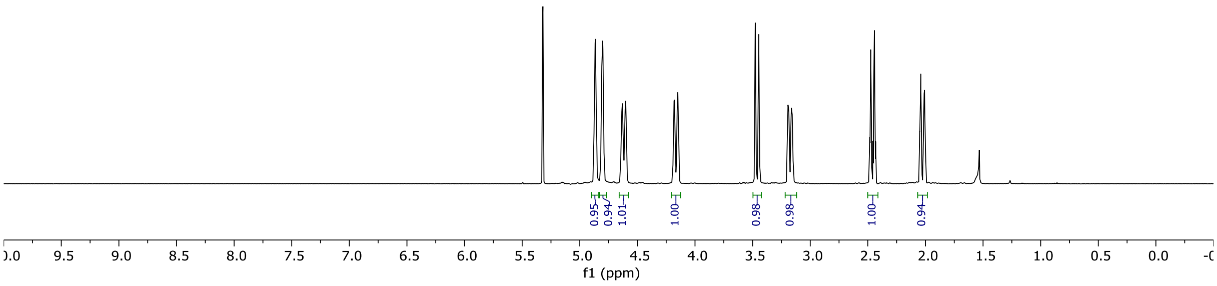

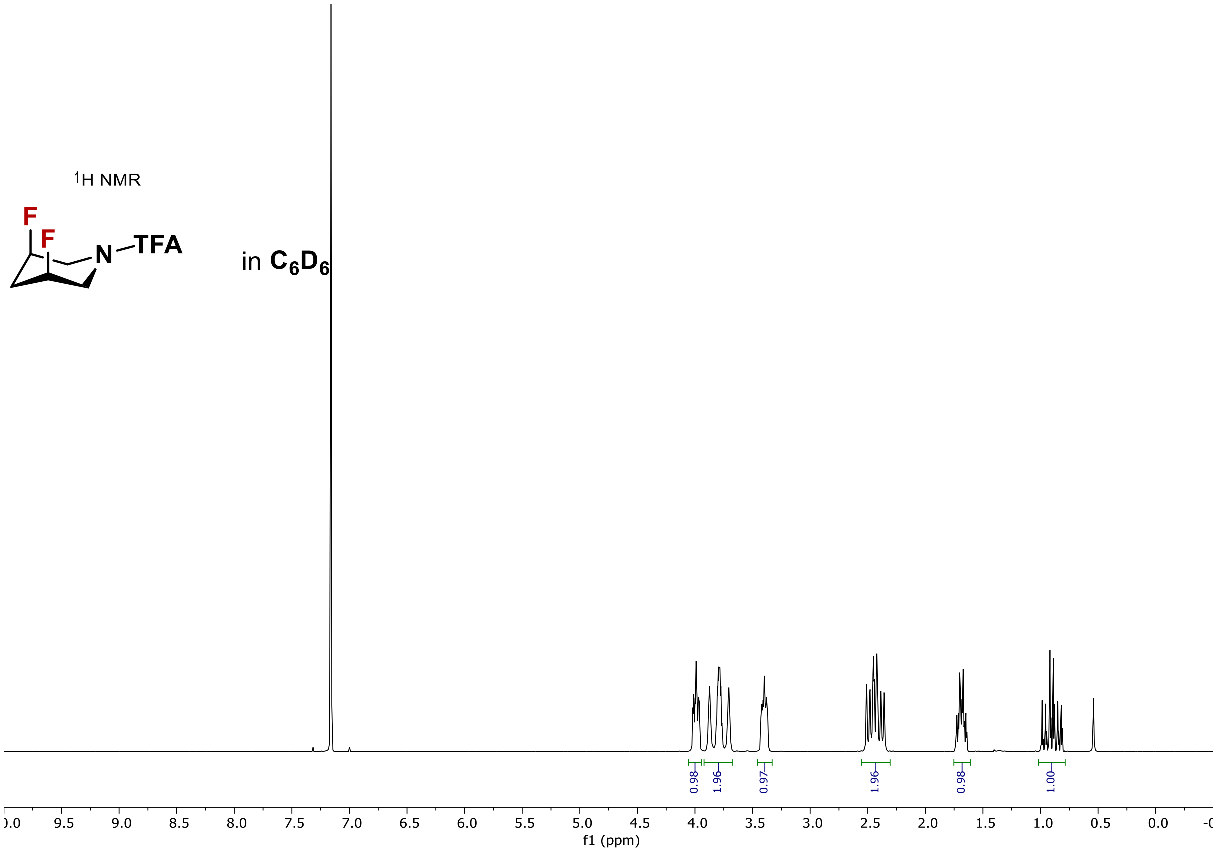
1H NMR
F
F
N–TFA
in C6D6
0.98
1.96
0.97
1.96
0.98
1.00
9.5 9.0 8.5 8.0 7.5 7.0 6.5 6.0 5.5 5.0 4.5 4.0 3.5 3.0 2.5 2.0 1.5 1.0 0.5 0.0
f1 (ppm)

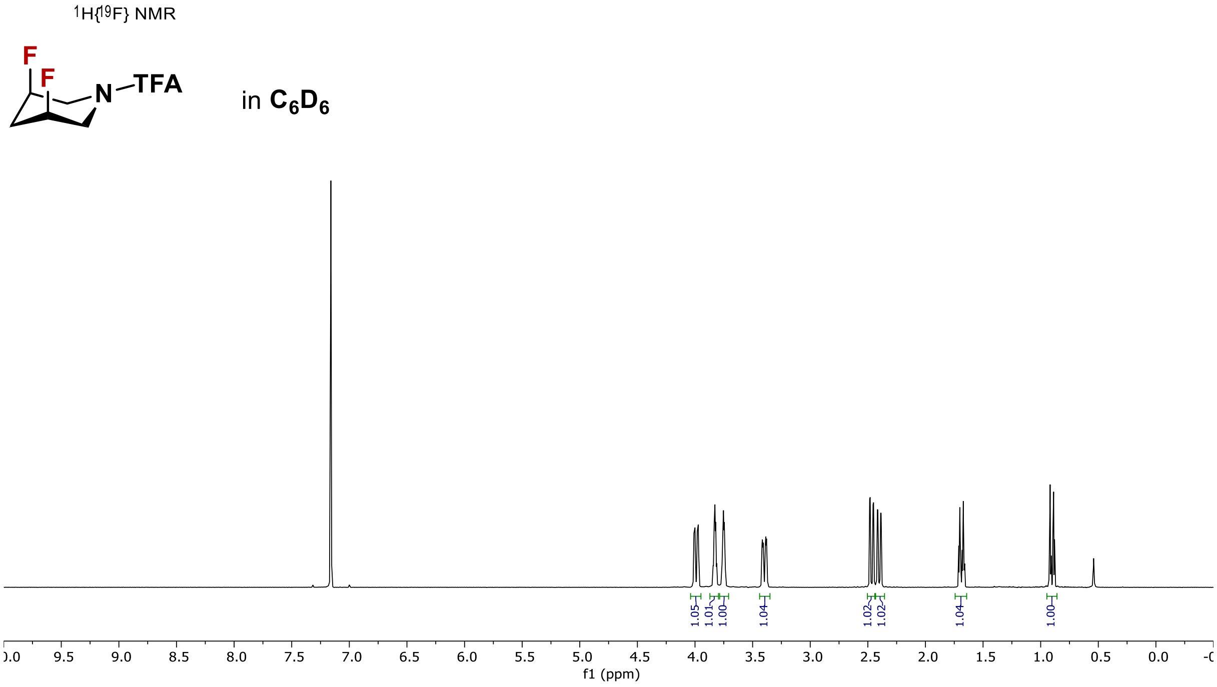
1H{19F} NMR
F
F
N–TFA
in C6D6
1.05
1.01
1.00
1.04
1.02
1.02
1.04
1.00
9.5 9.0 8.5 8.0 7.5 7.0 6.5 6.0 5.5 5.0 4.5 4.0 3.5 3.0 2.5 2.0 1.5 1.0 0.5 0.0
f1 (ppm)

^1H NMR

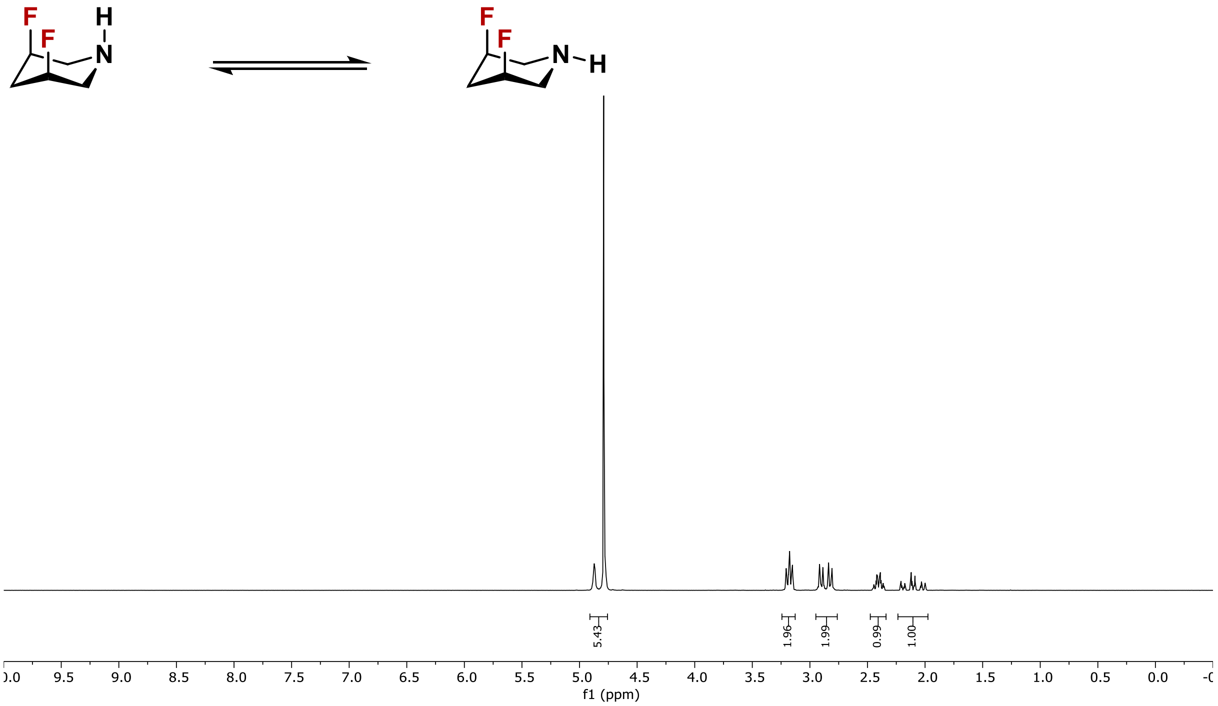

$^1H\{^{19}F\}$ NMR

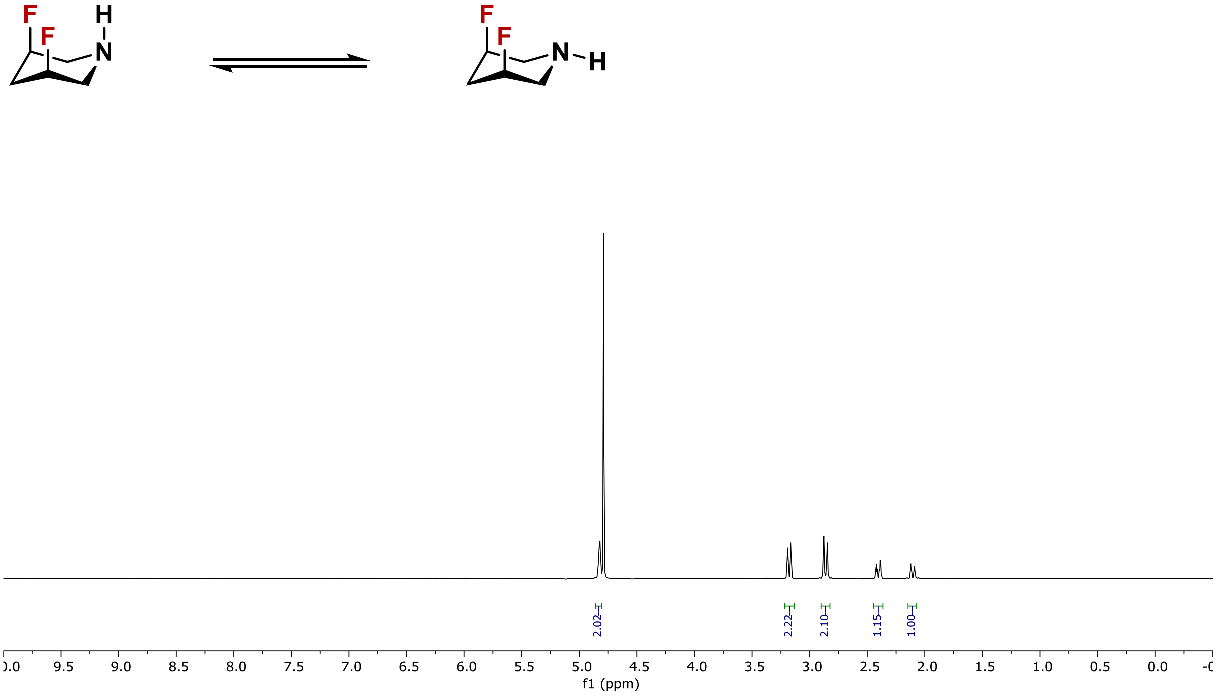

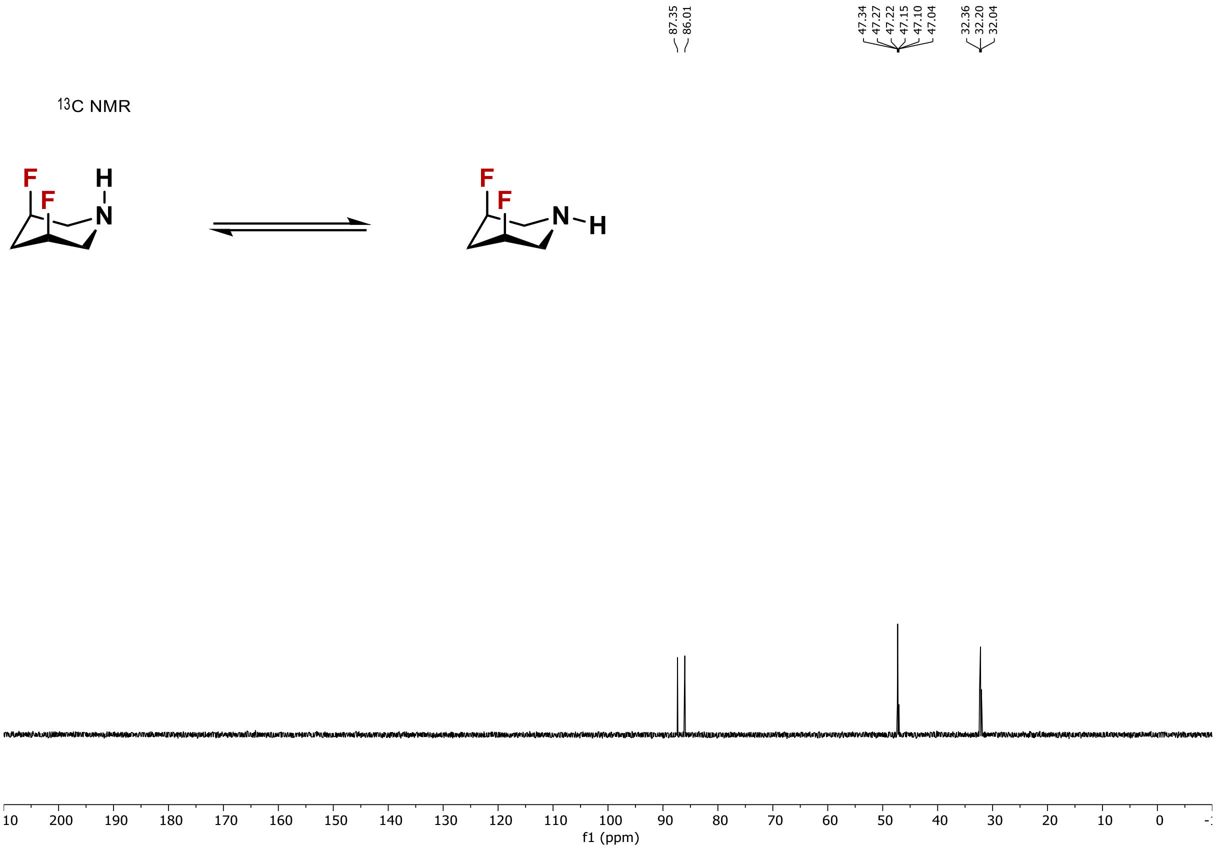
87.35
86.01
47.34
47.27
47.22
47.15
47.10
47.04
32.36
32.20
32.04
13C NMR
F
F
H
N
F
F
N
H
10 200 190 180 170 160 150 140 130 120 110 100 90 80 70 60 50 40 30 20 10 0
f1 (ppm)

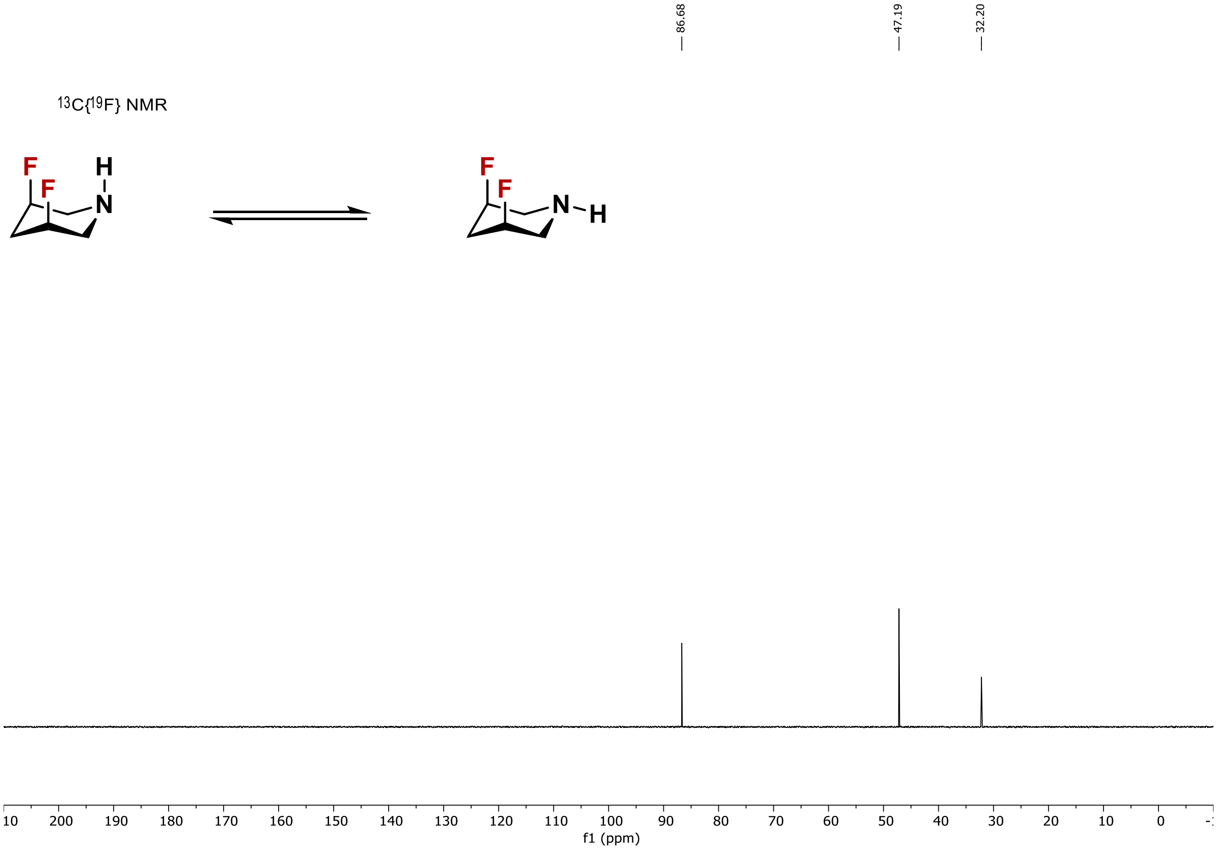
86.68
47.19
32.20
13C{19F} NMR
F
F
H
N
F
F
N
H
10 200 190 180 170 160 150 140 130 120 110 100 90 80 70 60 50 40 30 20 10 0
f1 (ppm)

^{19}F NMR

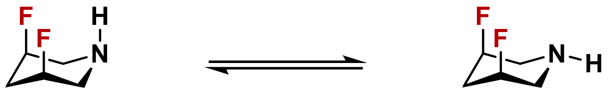

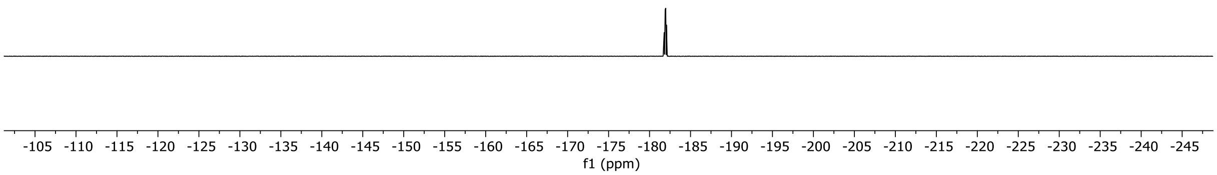

$^{19}F\{^1H\}$ NMR

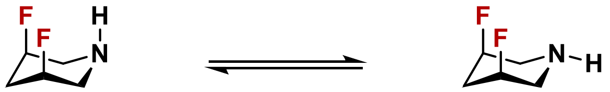

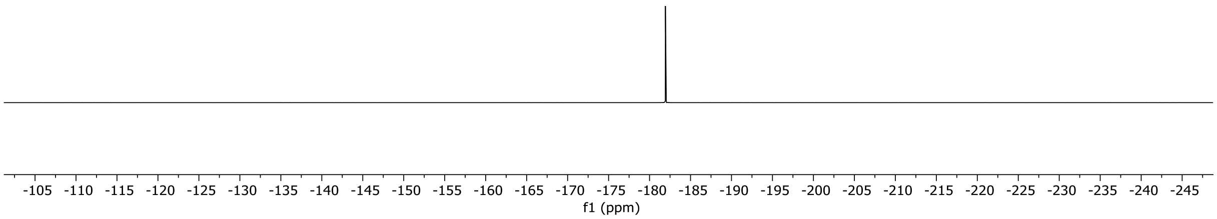

^{1}H NMR

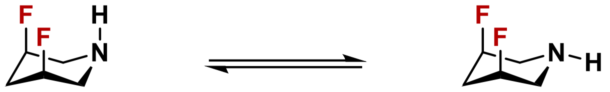

in **DMSO**

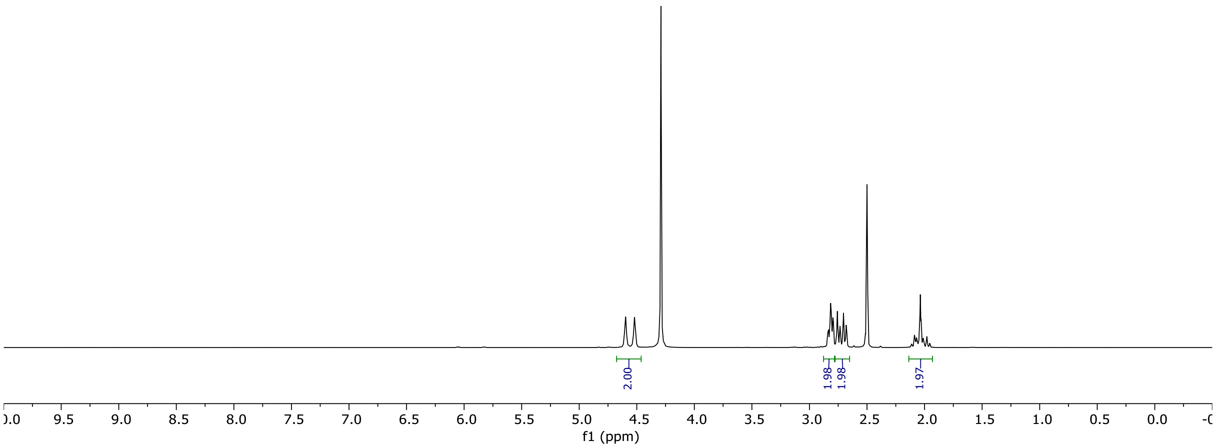

^{1}H{^{19}F} NMR

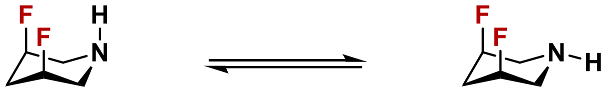

in **DMSO**

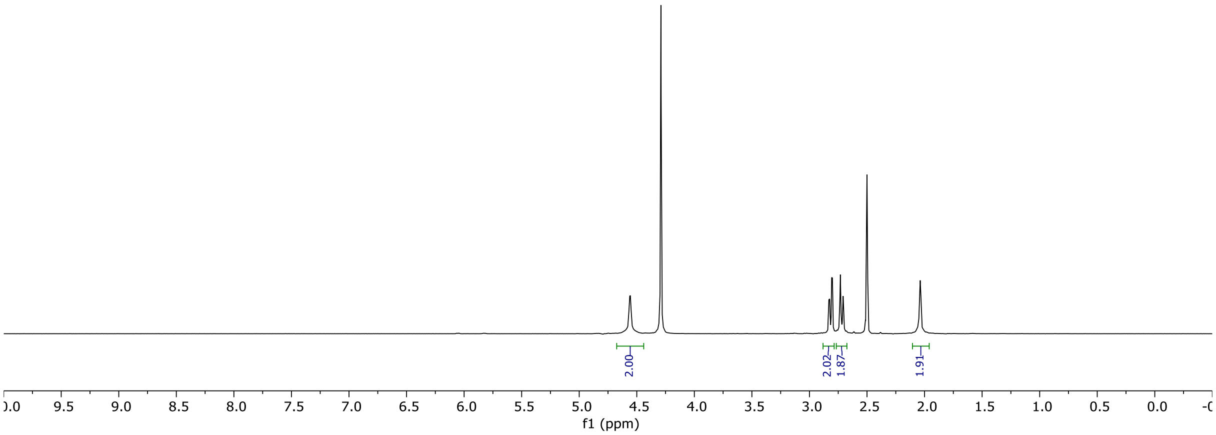

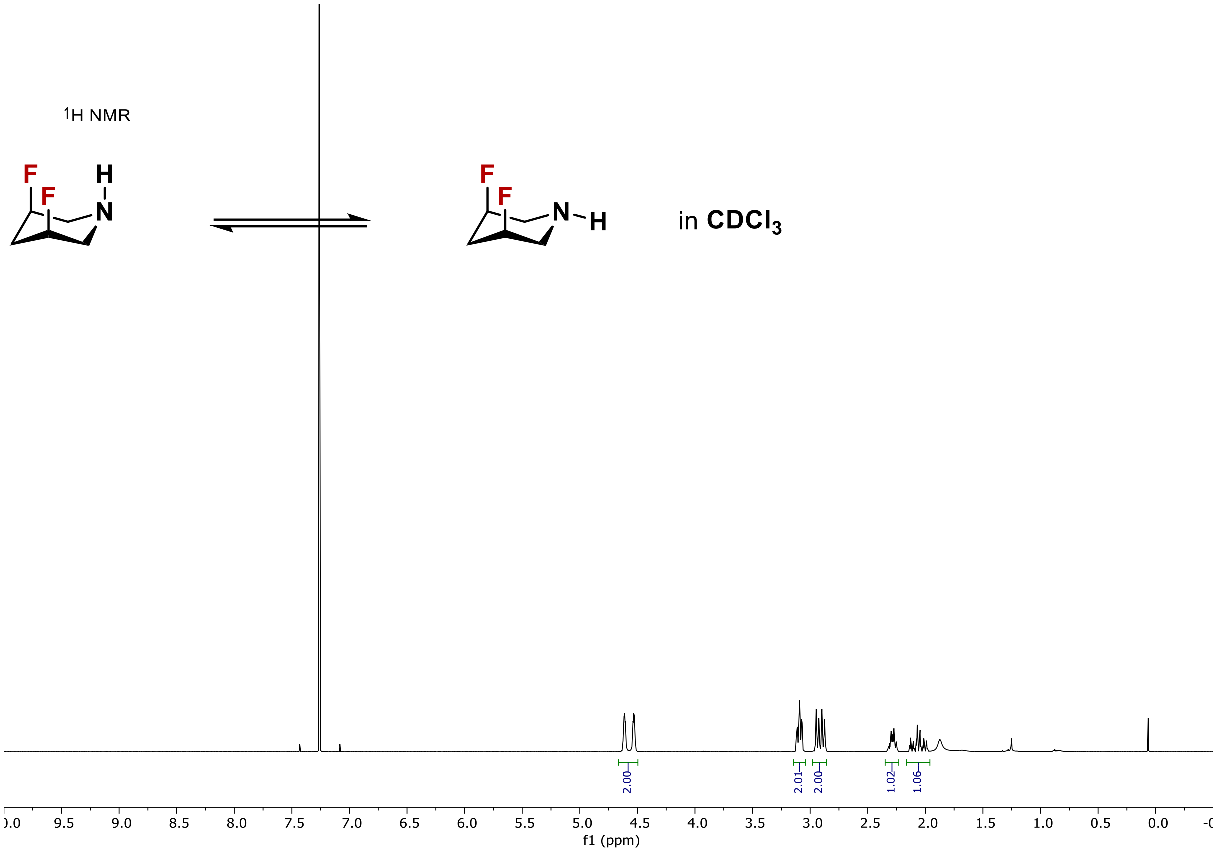

1H NMR
in CDCl3
2.00
2.01
2.00
1.02
1.06
f1 (ppm)

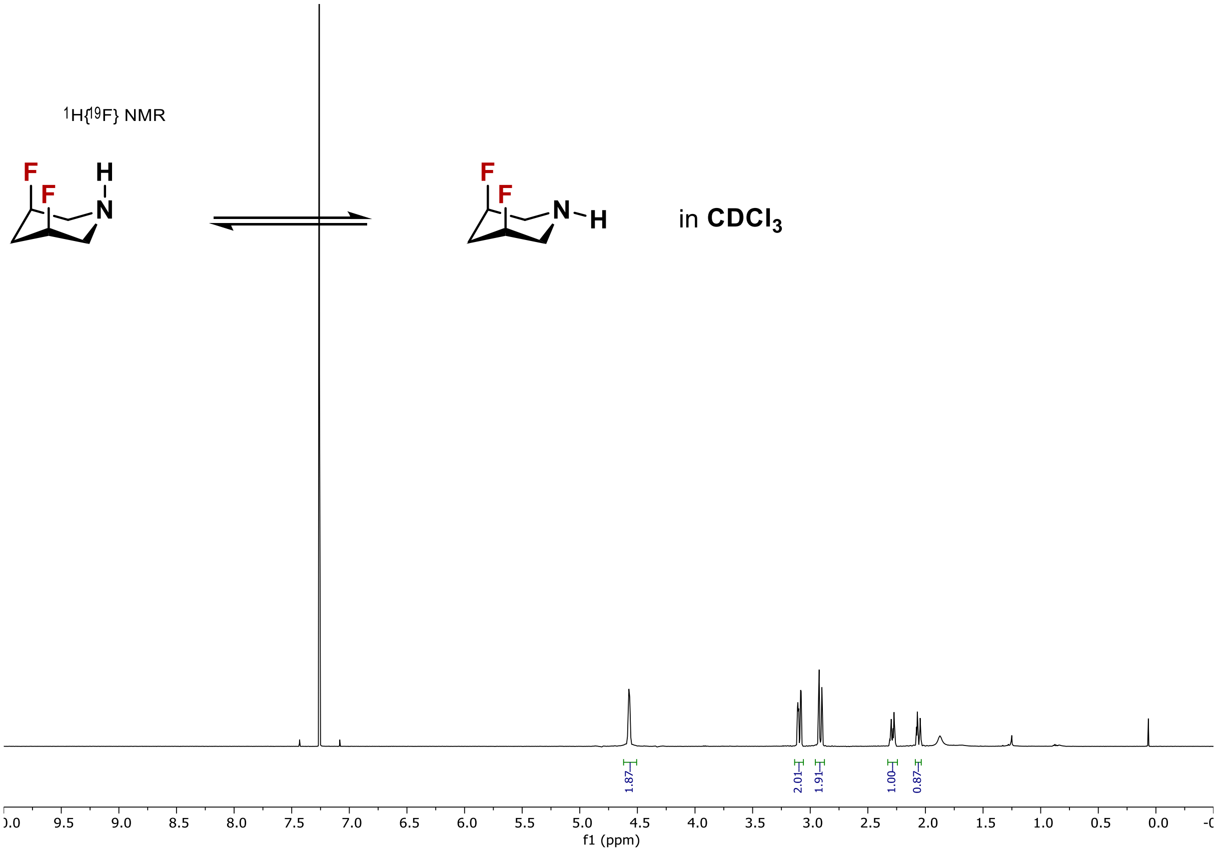

1H{19F} NMR
in CDCl3
1.87
2.01
1.91
1.00
0.87
f1 (ppm)

^1H NMR

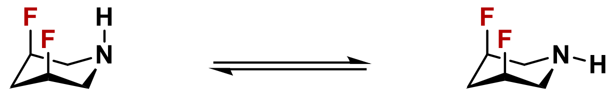

in CD_2Cl_2

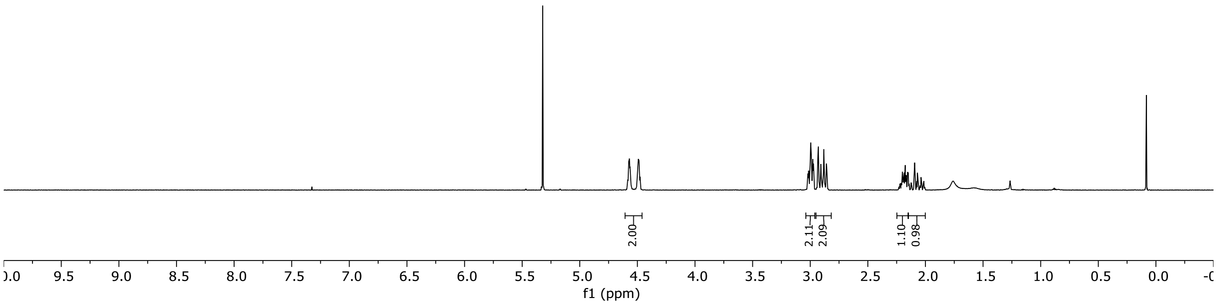

$^1H\{^{19}F\}$ NMR

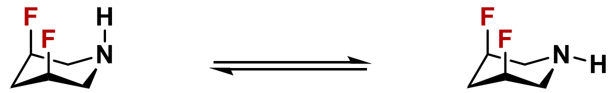

in CD_2Cl_2

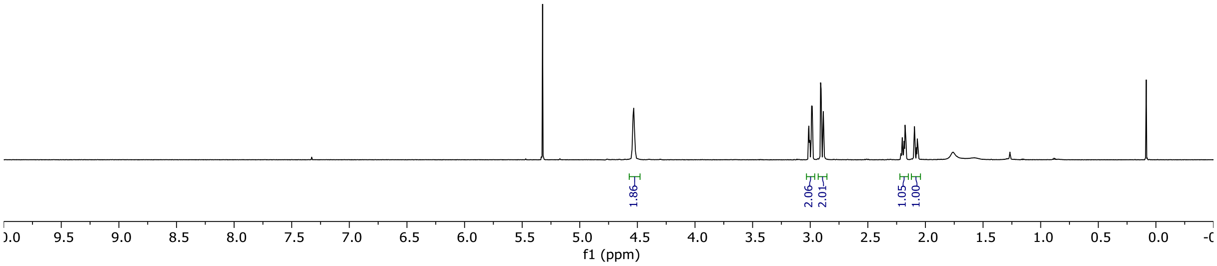

^{1}H NMR

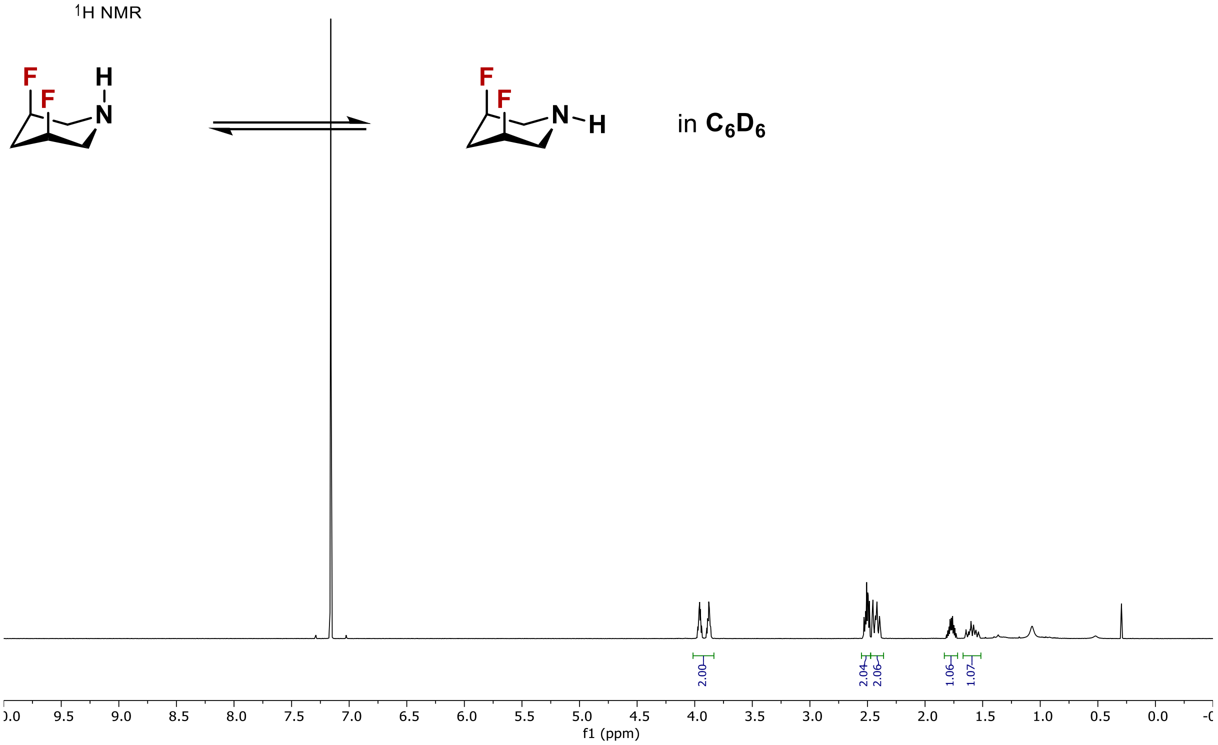

^{1}H{^{19}F} NMR

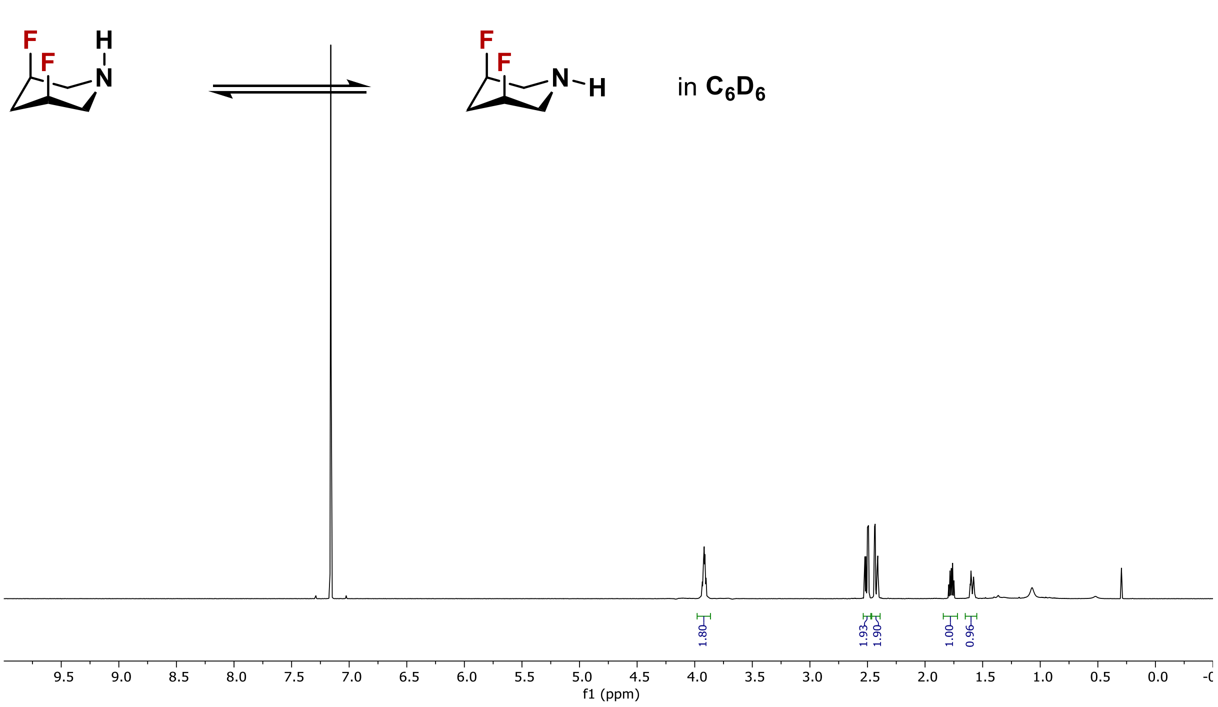

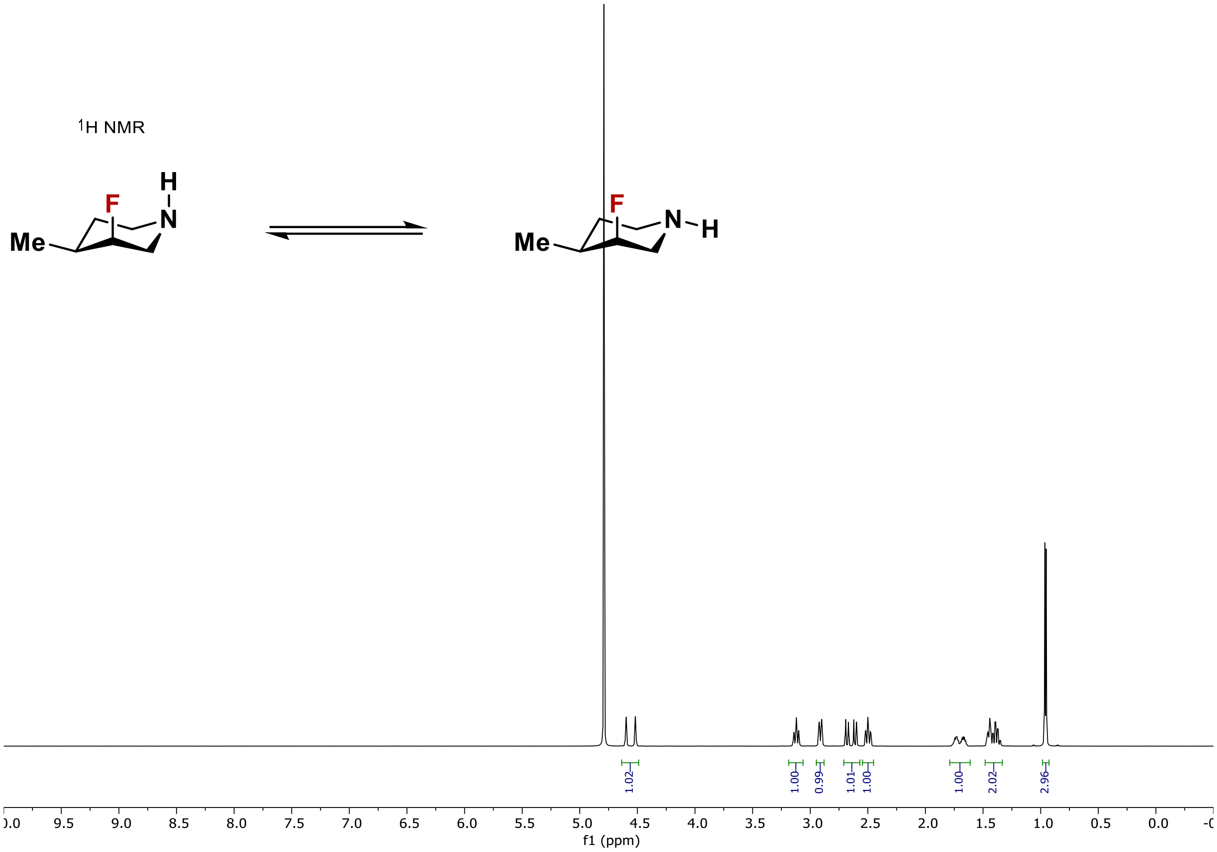

^1H NMR
Me
F
N
H
1.02
1.00
0.99
1.01
1.00
1.00
2.02
2.96
f1 (ppm)

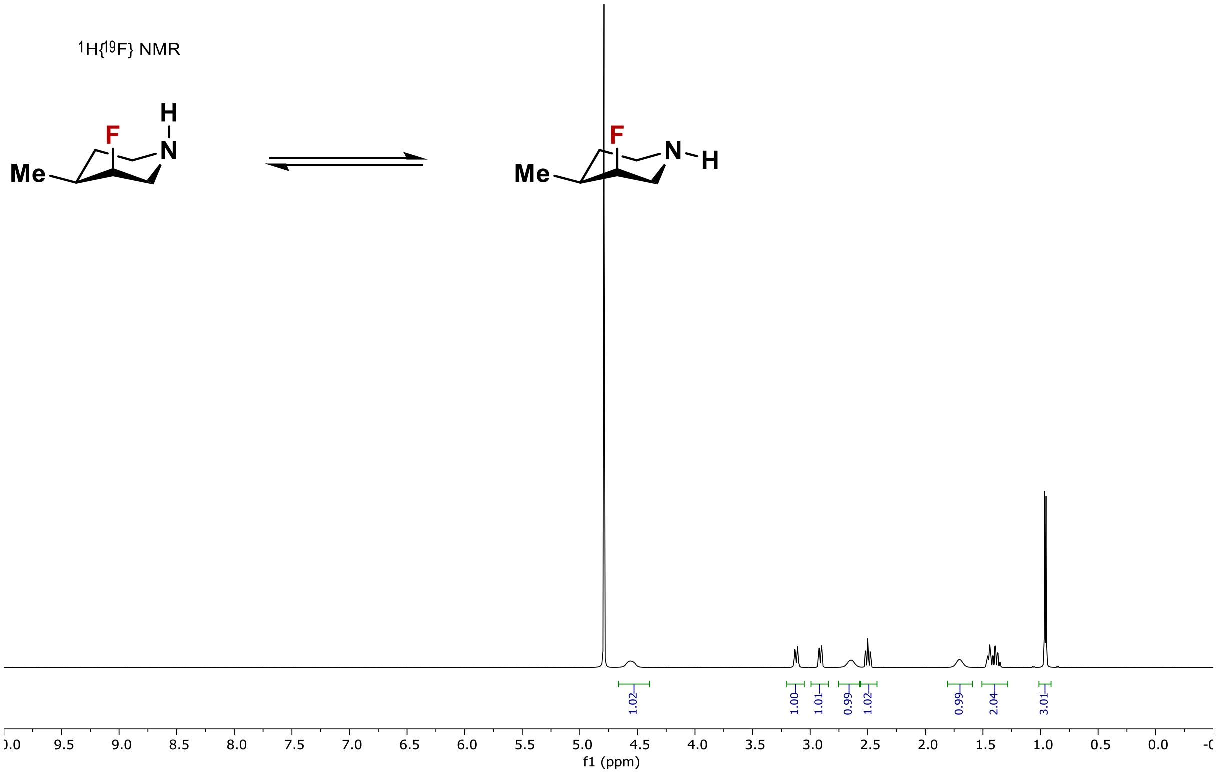

$^1H\{^{19}F\}$ NMR
Me
F
N
H
1.02
1.00
1.01
0.99
1.02
0.99
2.04
3.01
f1 (ppm)

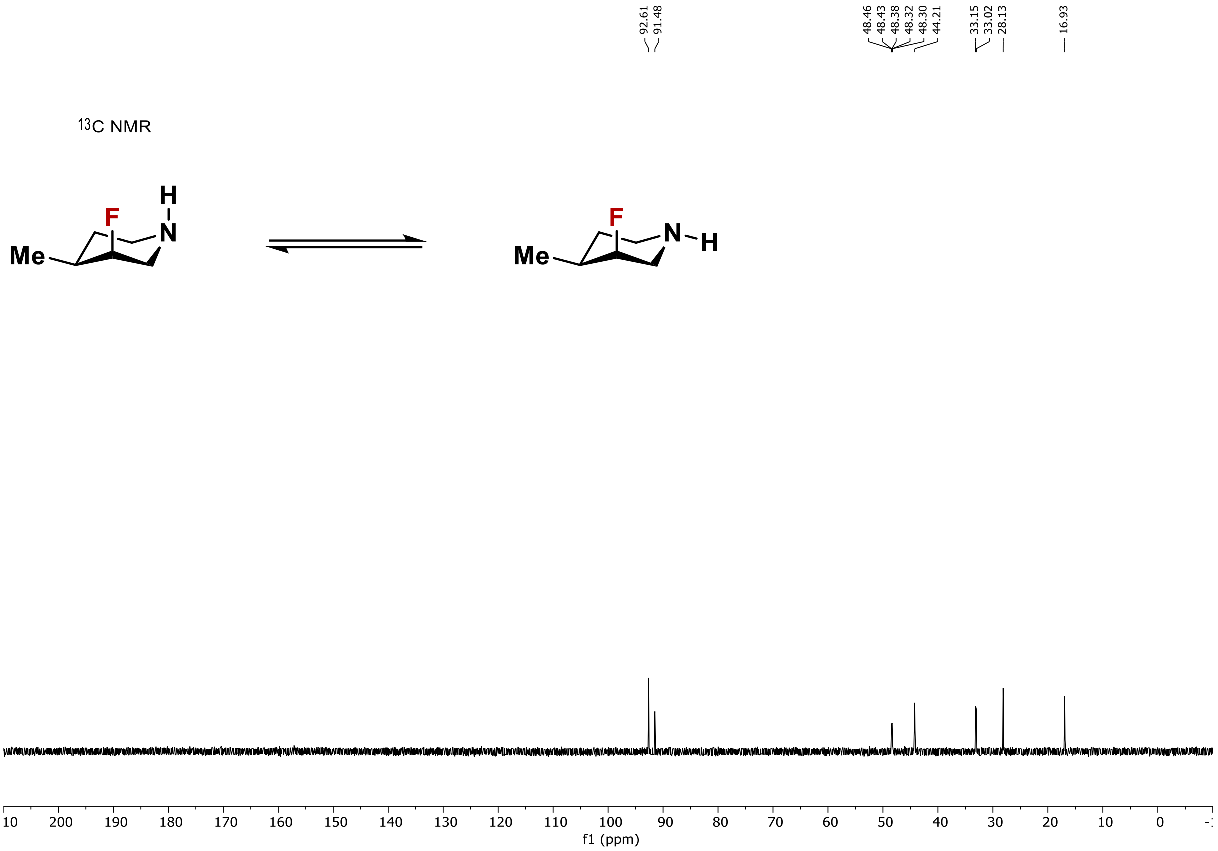

92.61
91.48
48.46
48.43
48.38
48.32
48.30
44.21
33.15
33.02
28.13
16.93
13C NMR
F
H
N
Me
F
N
H
Me
10 200 190 180 170 160 150 140 130 120 110 100 90 80 70 60 50 40 30 20 10 0
f1 (ppm)

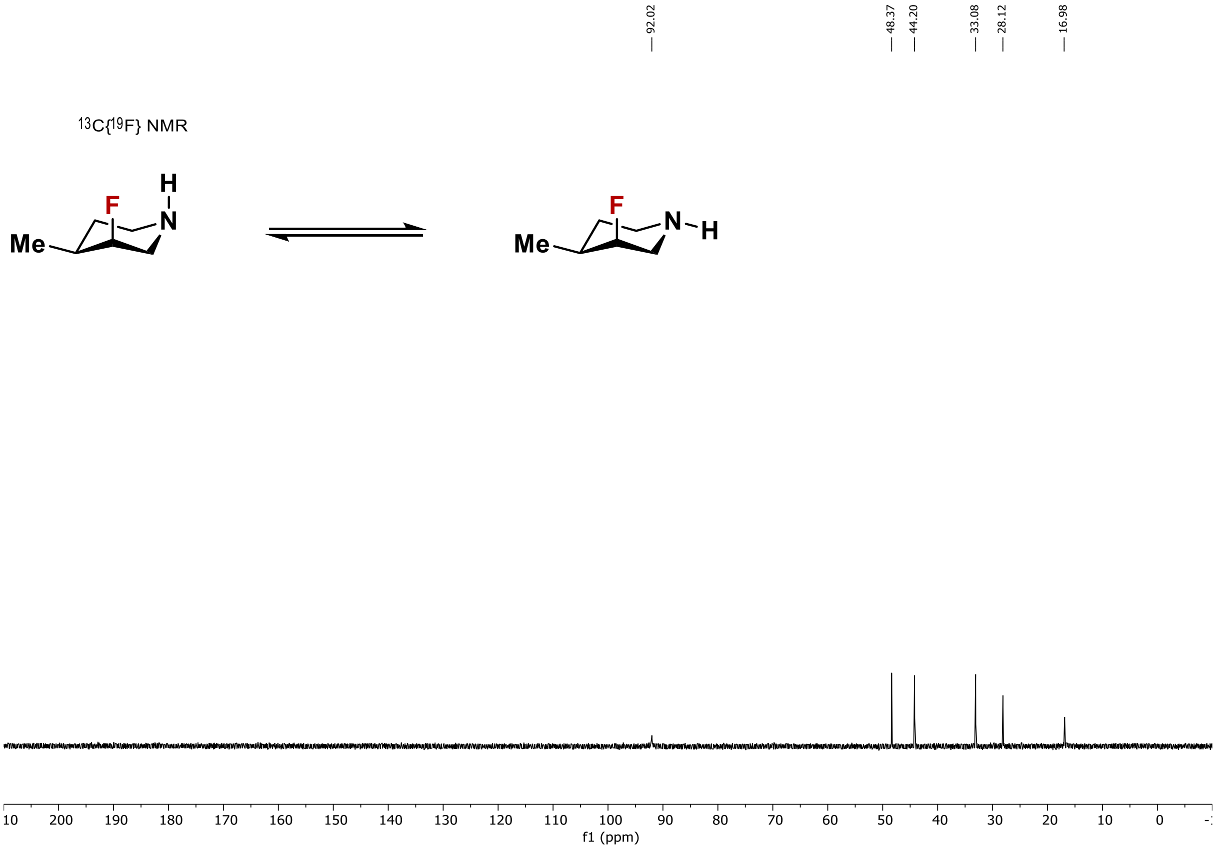

92.02
48.37
44.20
33.08
28.12
16.98
13C{19F} NMR
F
H
N
Me
F
N
H
Me
10 200 190 180 170 160 150 140 130 120 110 100 90 80 70 60 50 40 30 20 10 0
f1 (ppm)

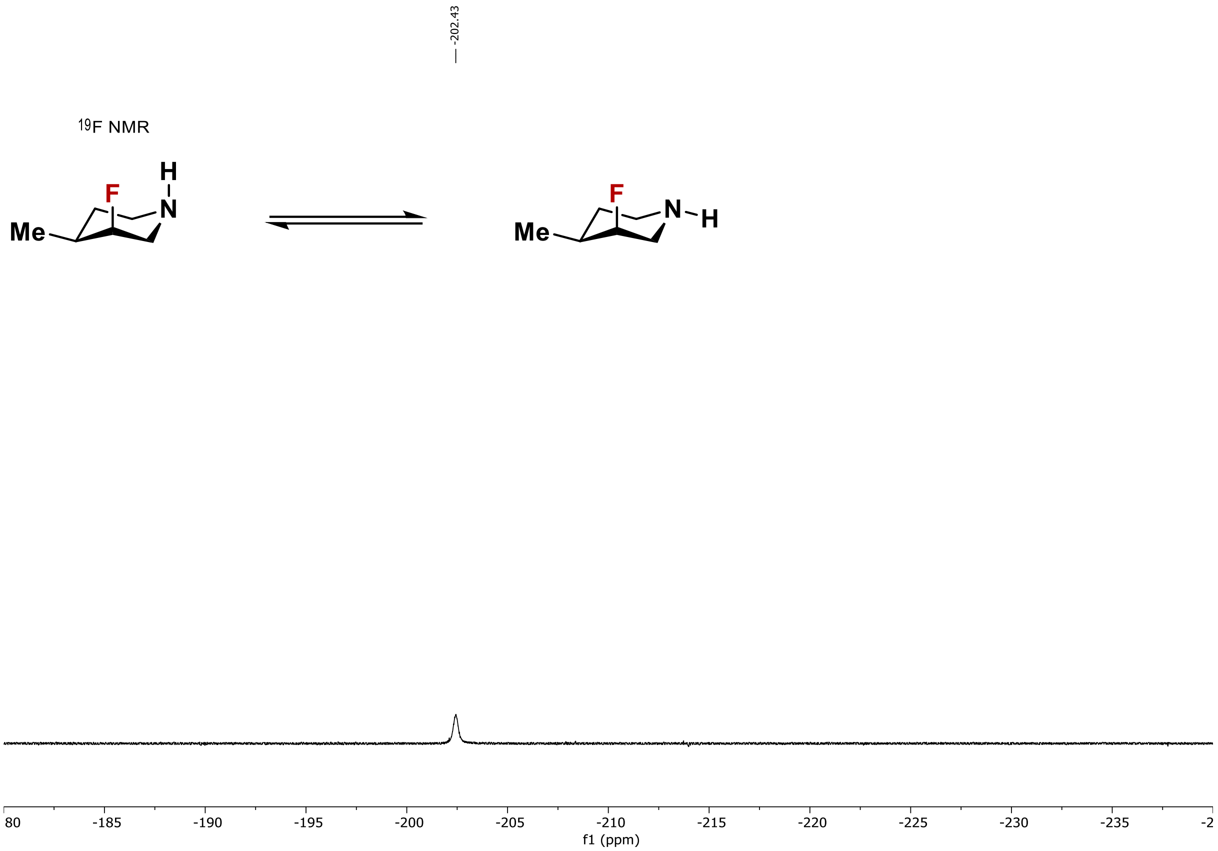
-202.43
19F NMR
F
H
N
Me
F
N
H
Me
80
-185
-190
-195
-200
-205
-210
-215
-220
-225
-230
-235
-2
f1 (ppm)

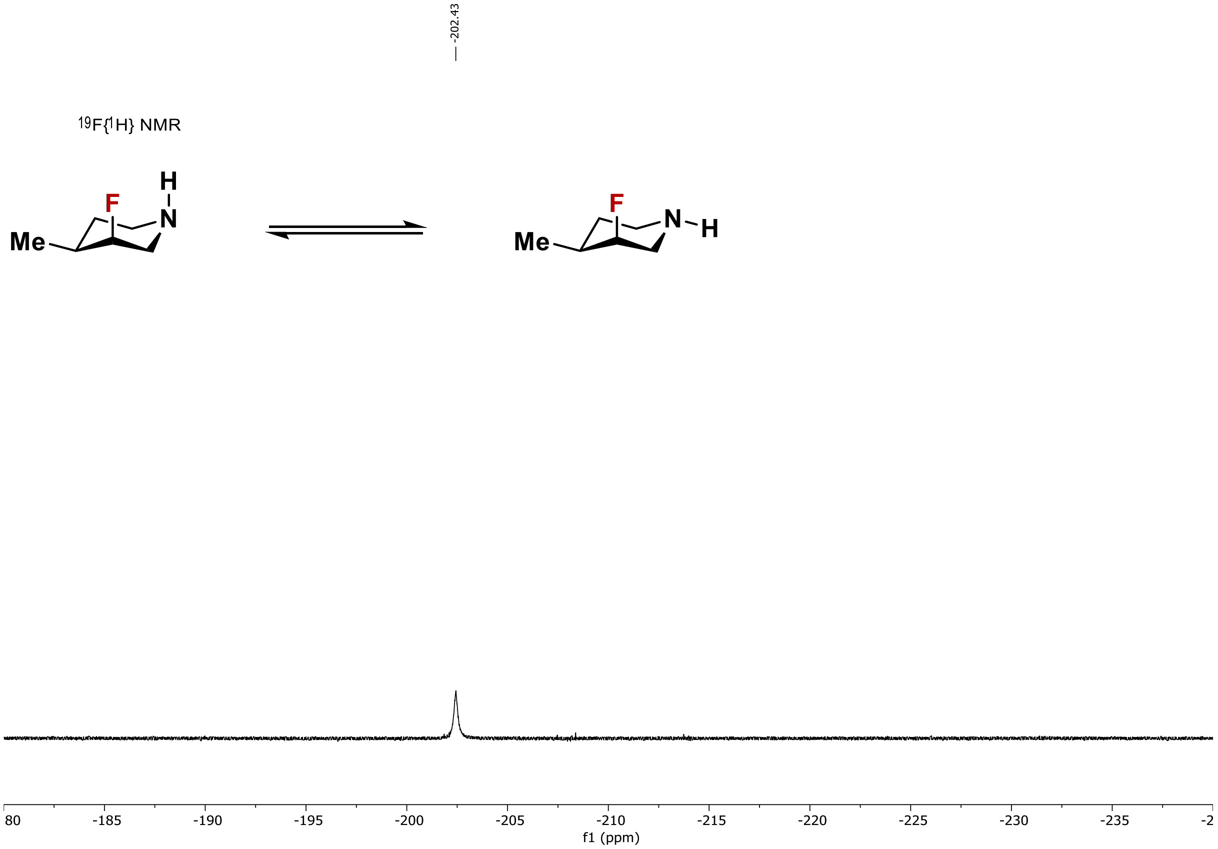
-202.43
19F{1H} NMR
F
H
N
Me
F
N
H
Me
80
-185
-190
-195
-200
-205
-210
-215
-220
-225
-230
-235
-2
f1 (ppm)

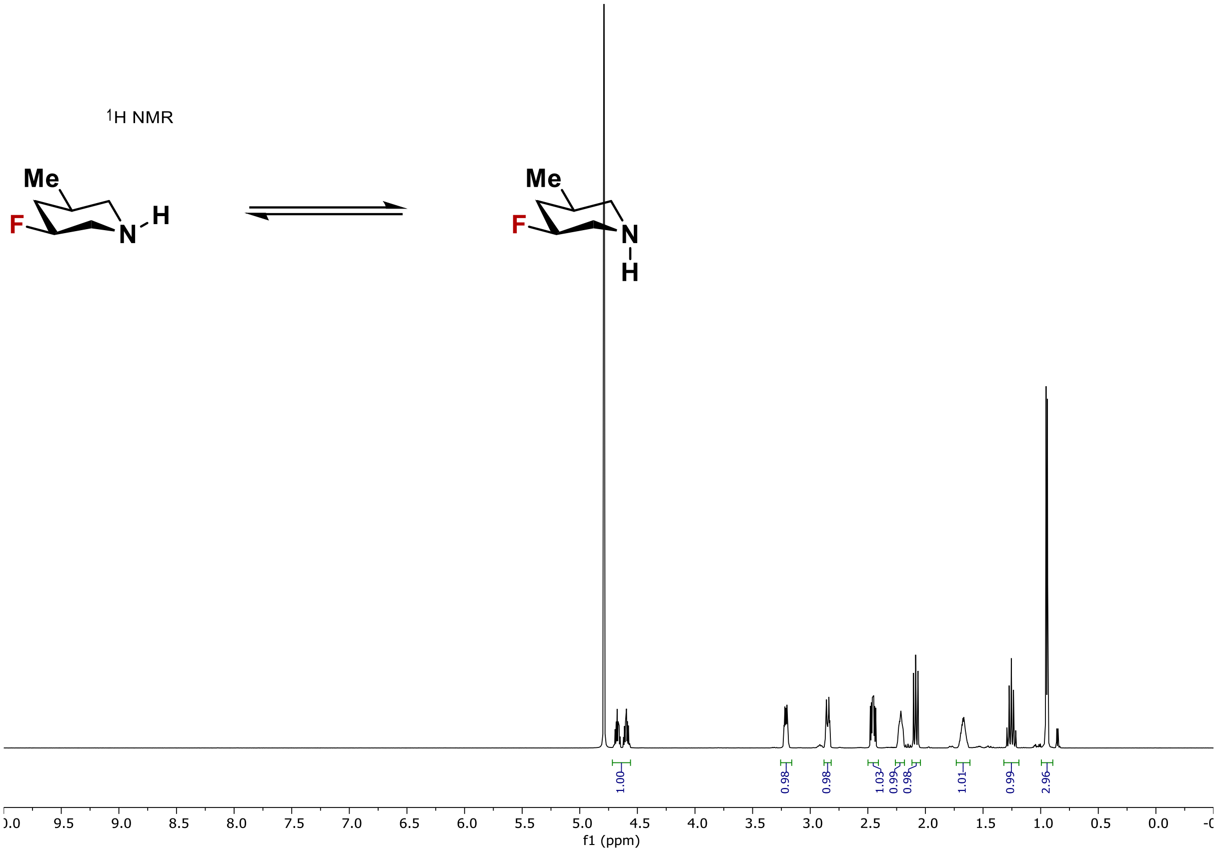

^{1}H NMR
Me
F
N
H
1.00
0.98
0.98
1.03
0.99
0.98
1.01
0.99
2.96
f1 (ppm)

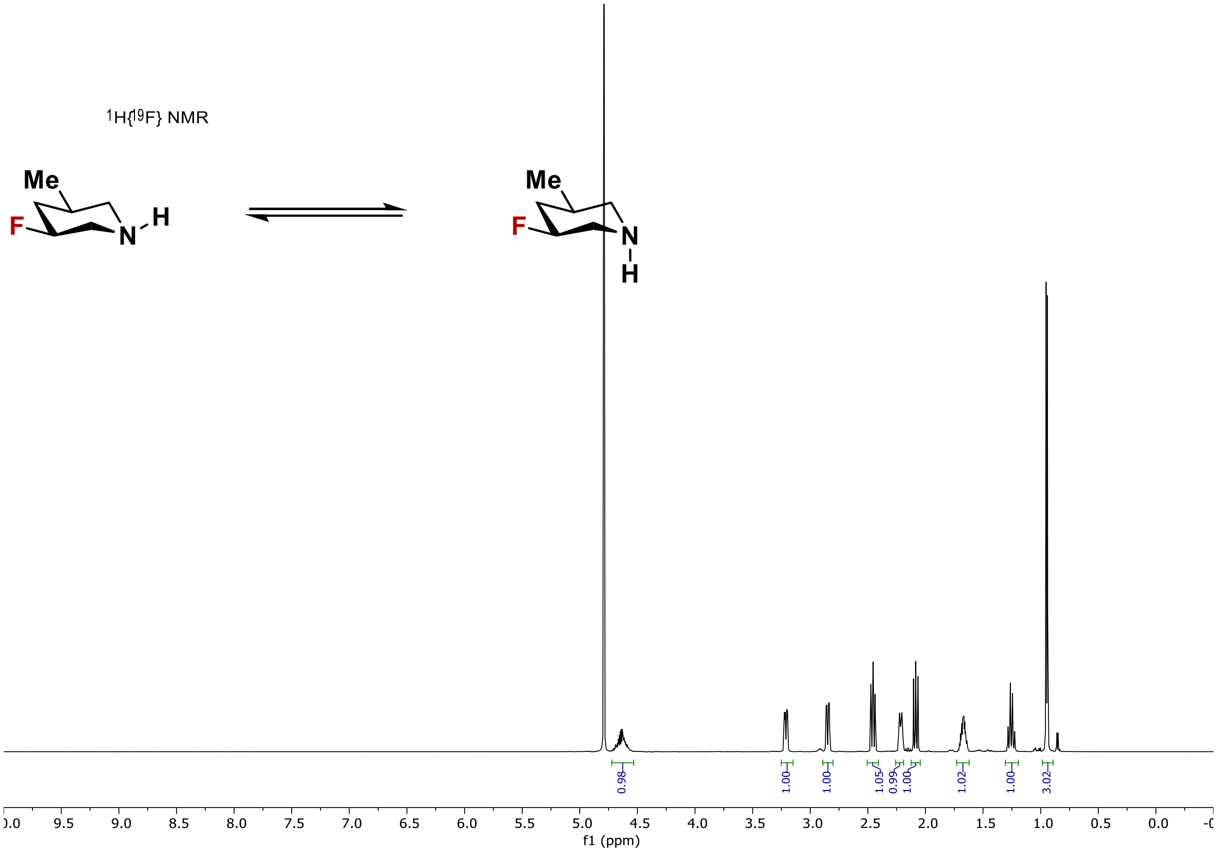

^{1}H{^{19}F} NMR
Me
F
N
H
0.98
1.00
1.00
1.05
0.99
1.00
1.02
1.00
3.02
f1 (ppm)

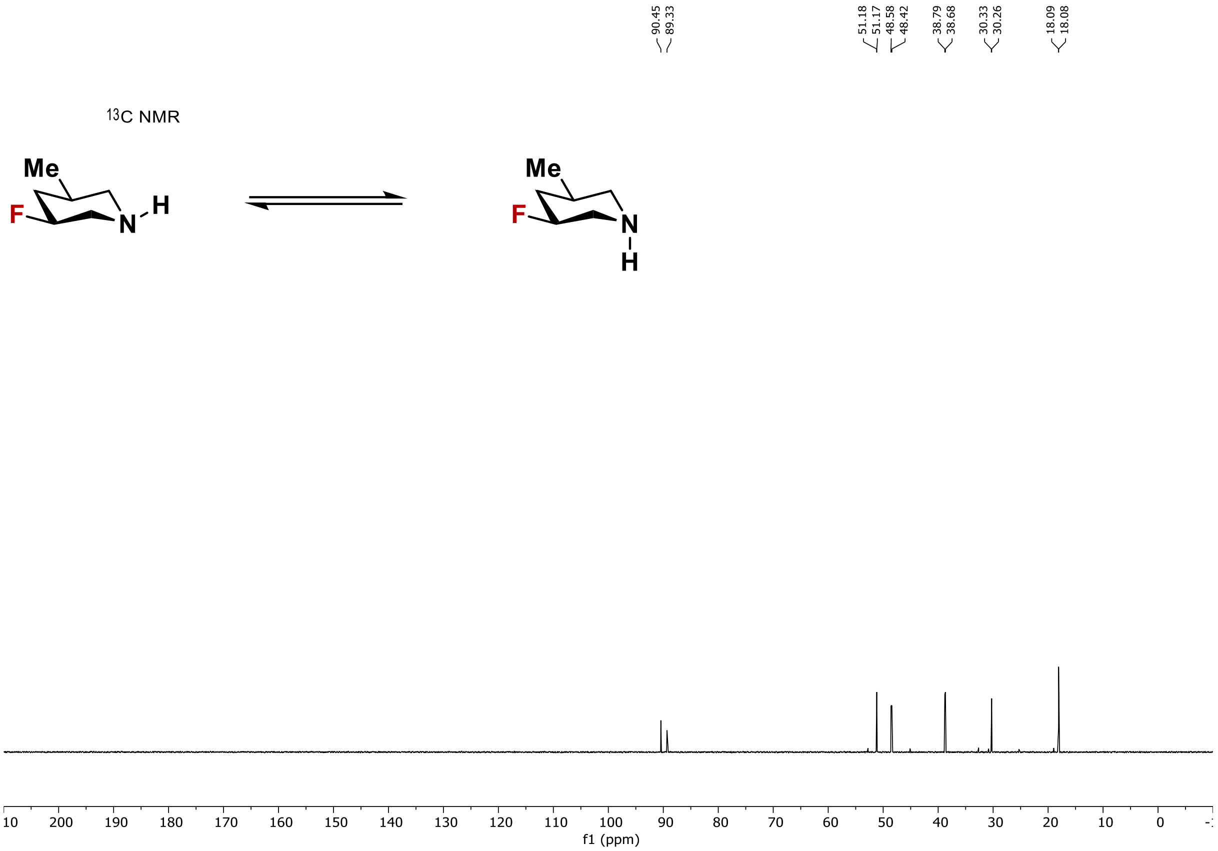

90.45
89.33
51.18
51.17
48.58
48.42
38.79
38.68
30.33
30.26
18.09
18.08
13C NMR
Me
F
N
H
Me
F
N
H
10
200
190
180
170
160
150
140
130
120
110
100
90
80
70
60
50
40
30
20
10
0
f1 (ppm)

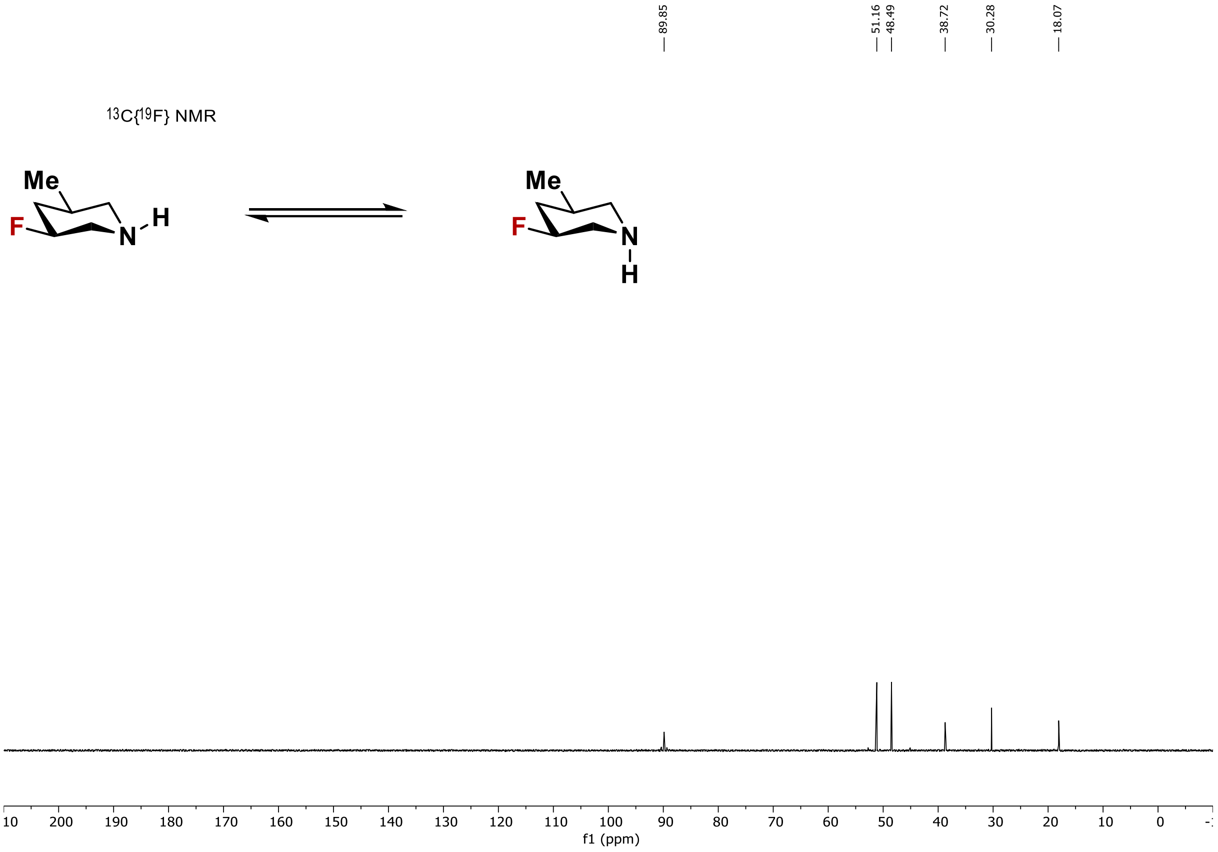

89.85
51.16
48.49
38.72
30.28
18.07
13C{19F} NMR
Me
F
N
H
Me
F
N
H
10
200
190
180
170
160
150
140
130
120
110
100
90
80
70
60
50
40
30
20
10
0
f1 (ppm)

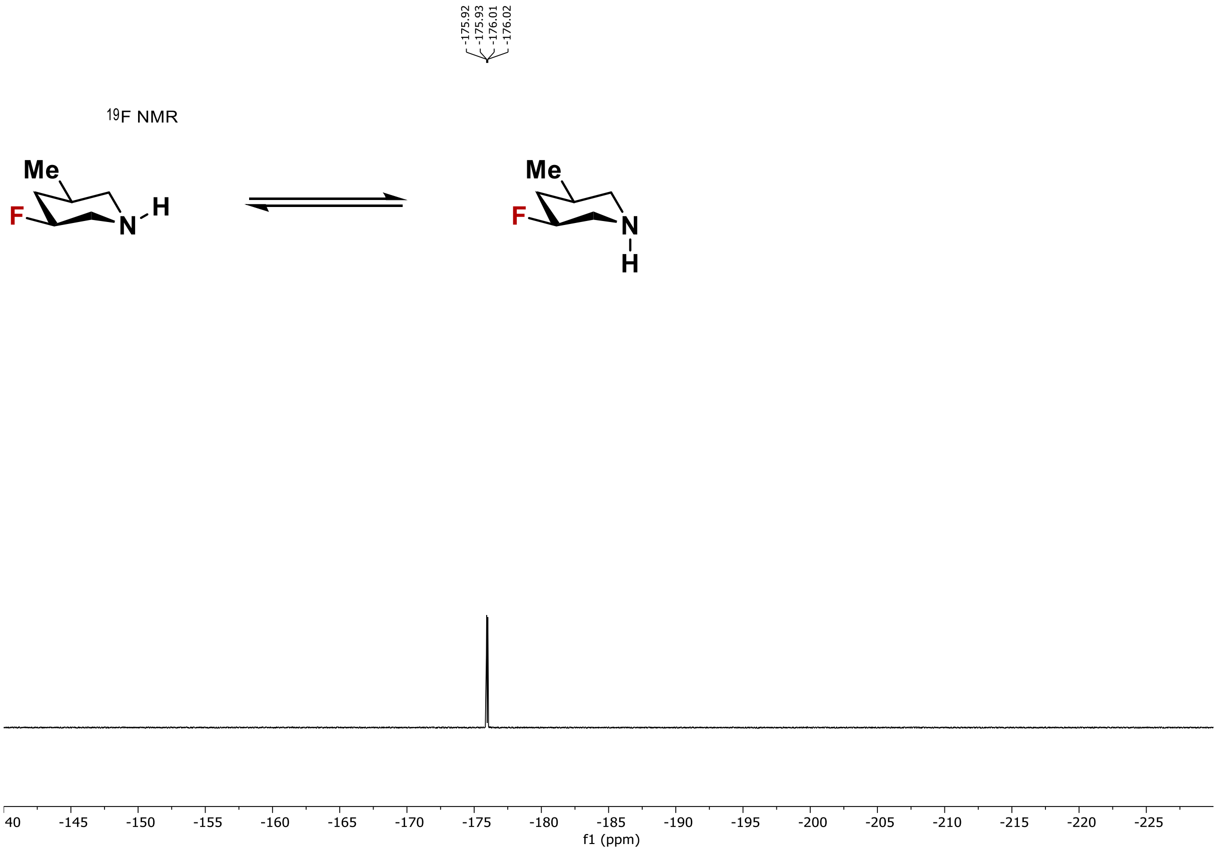

-175.92
-175.93
-176.01
-176.02
19F NMR
Me
F
N
H
Me
F
N
H
40 -145 -150 -155 -160 -165 -170 -175 -180 -185 -190 -195 -200 -205 -210 -215 -220 -225
f1 (ppm)

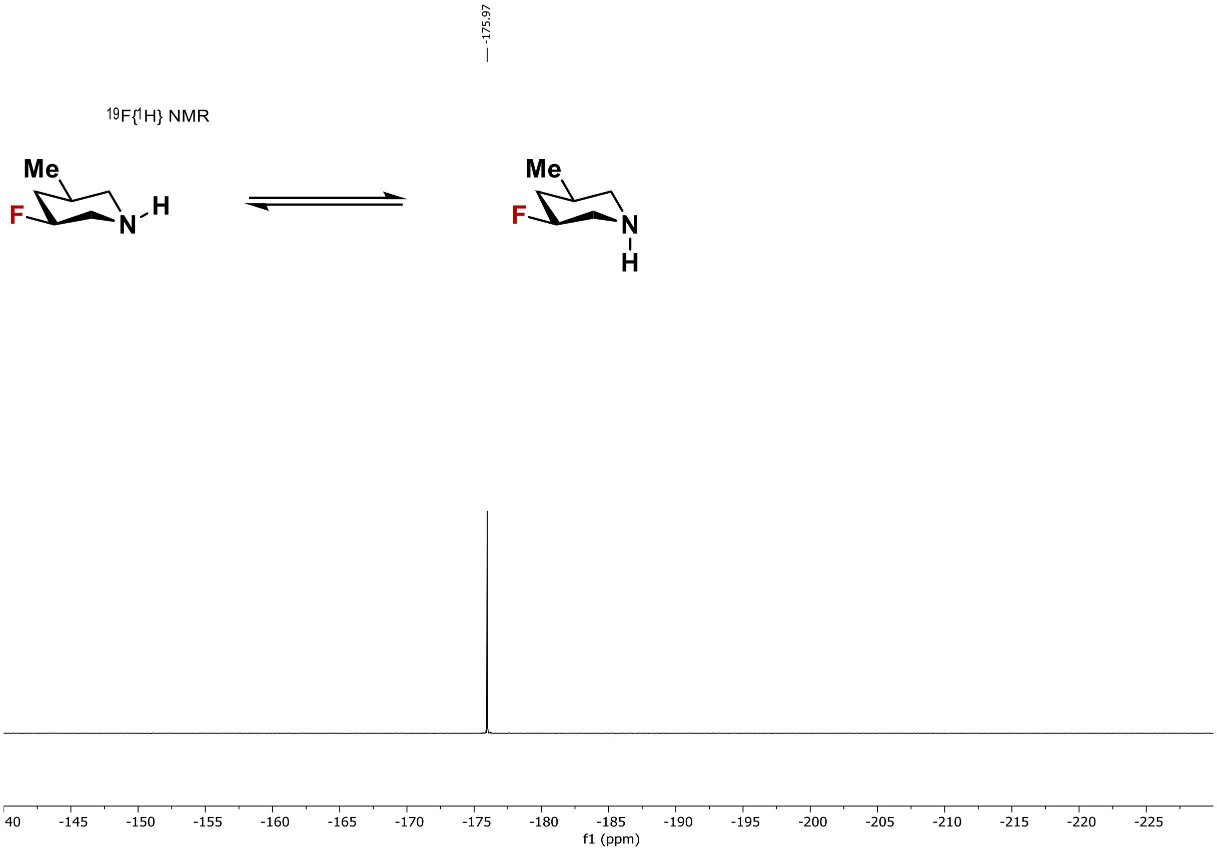

-175.97
19F{1H} NMR
Me
F
N
H
Me
F
N
H
40 -145 -150 -155 -160 -165 -170 -175 -180 -185 -190 -195 -200 -205 -210 -215 -220 -225
f1 (ppm)

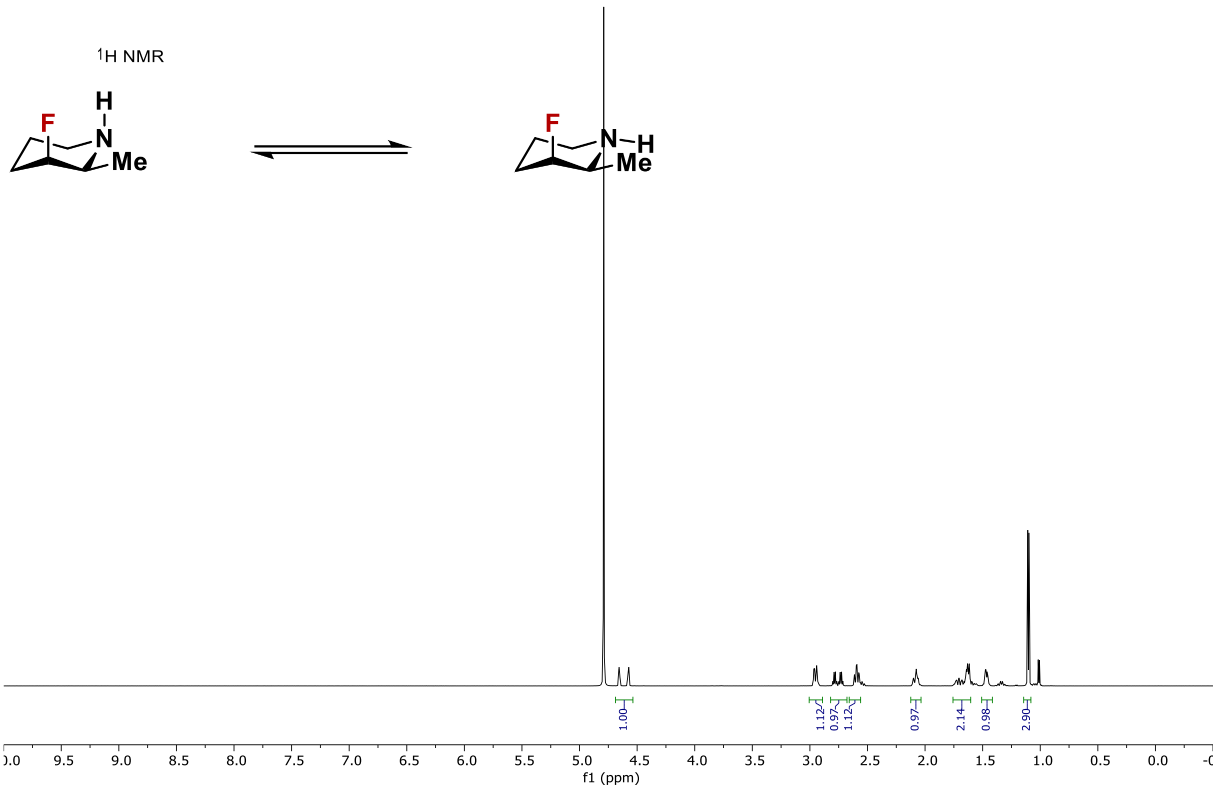

¹H NMR
F
H
N
Me
N–H
1.00
1.12
0.97
1.12
0.97
2.14
0.98
2.90
0.0 9.5 9.0 8.5 8.0 7.5 7.0 6.5 6.0 5.5 5.0 4.5 4.0 3.5 3.0 2.5 2.0 1.5 1.0 0.5 0.0
f1 (ppm)

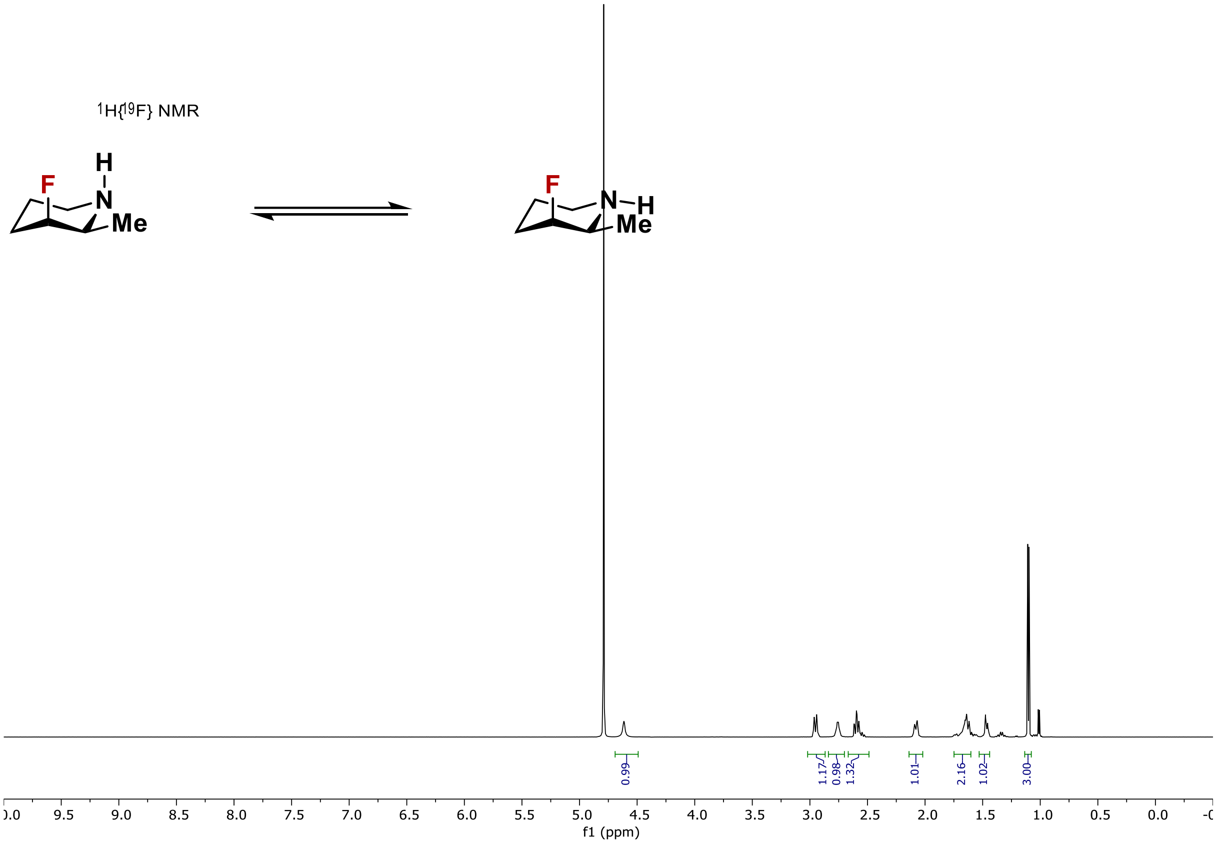

¹H{¹⁹F} NMR
F
H
N
Me
N–H
0.99
1.17
0.98
1.32
1.01
2.16
1.02
3.00
0.0 9.5 9.0 8.5 8.0 7.5 7.0 6.5 6.0 5.5 5.0 4.5 4.0 3.5 3.0 2.5 2.0 1.5 1.0 0.5 0.0
f1 (ppm)

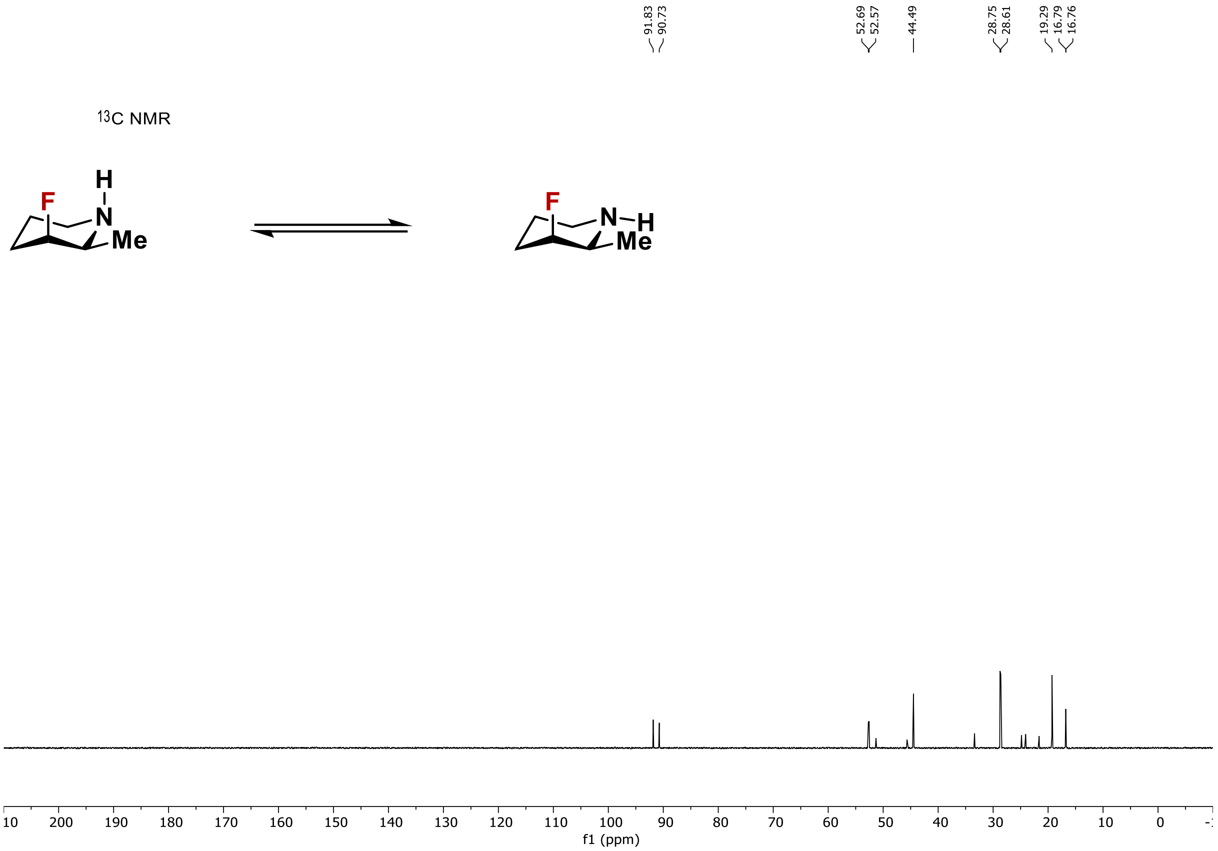
91.83
90.73
52.69
52.57
44.49
28.75
28.61
19.29
16.79
16.76
13C NMR
F
H
N
Me
F
N–H
Me
10 200 190 180 170 160 150 140 130 120 110 100 90 80 70 60 50 40 30 20 10 0
f1 (ppm)

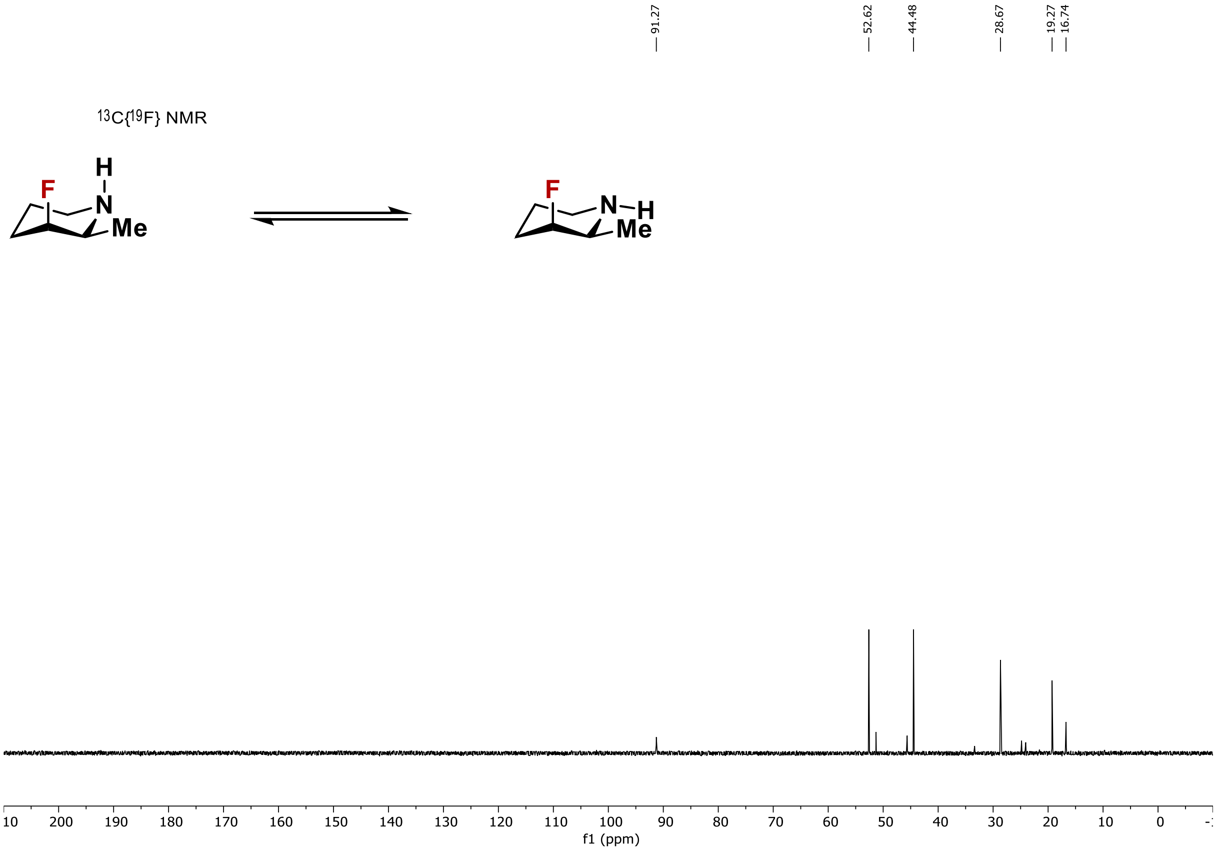
91.27
52.62
44.48
28.67
19.27
16.74
13C{19F} NMR
F
H
N
Me
F
N–H
Me
10 200 190 180 170 160 150 140 130 120 110 100 90 80 70 60 50 40 30 20 10 0
f1 (ppm)

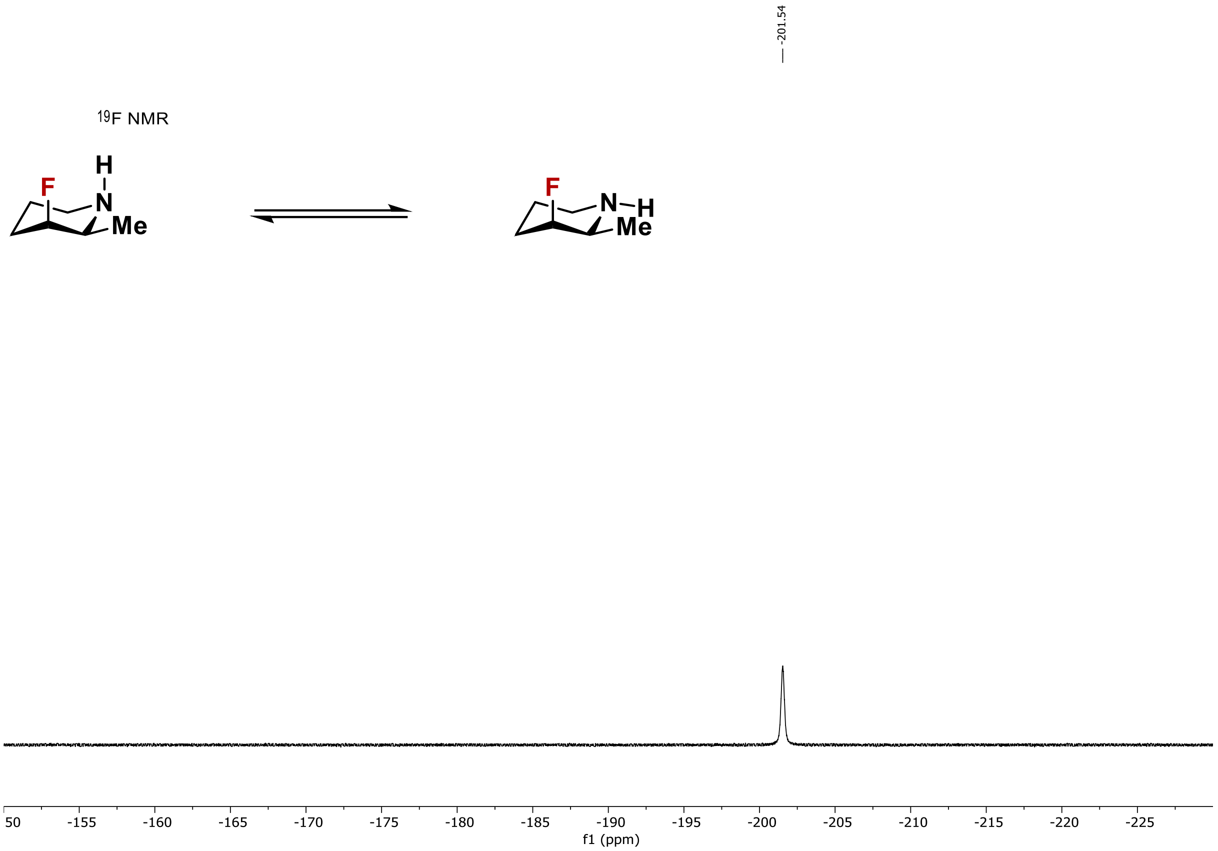
-201.54
19F NMR
F
H
N
Me
F
N
H
Me
50 -155 -160 -165 -170 -175 -180 -185 -190 -195 -200 -205 -210 -215 -220 -225
f1 (ppm)

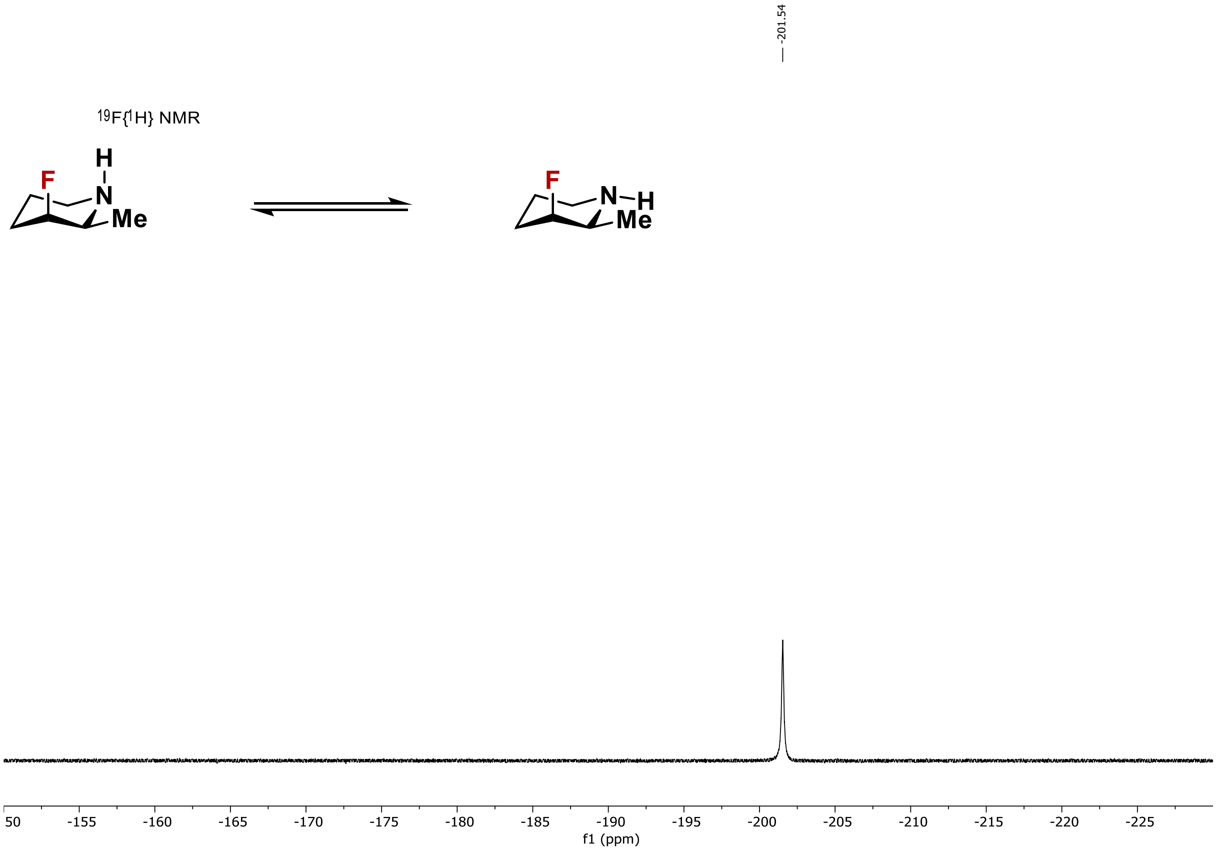
-201.54
19F{1H} NMR
F
H
N
Me
F
N
H
Me
50 -155 -160 -165 -170 -175 -180 -185 -190 -195 -200 -205 -210 -215 -220 -225
f1 (ppm)

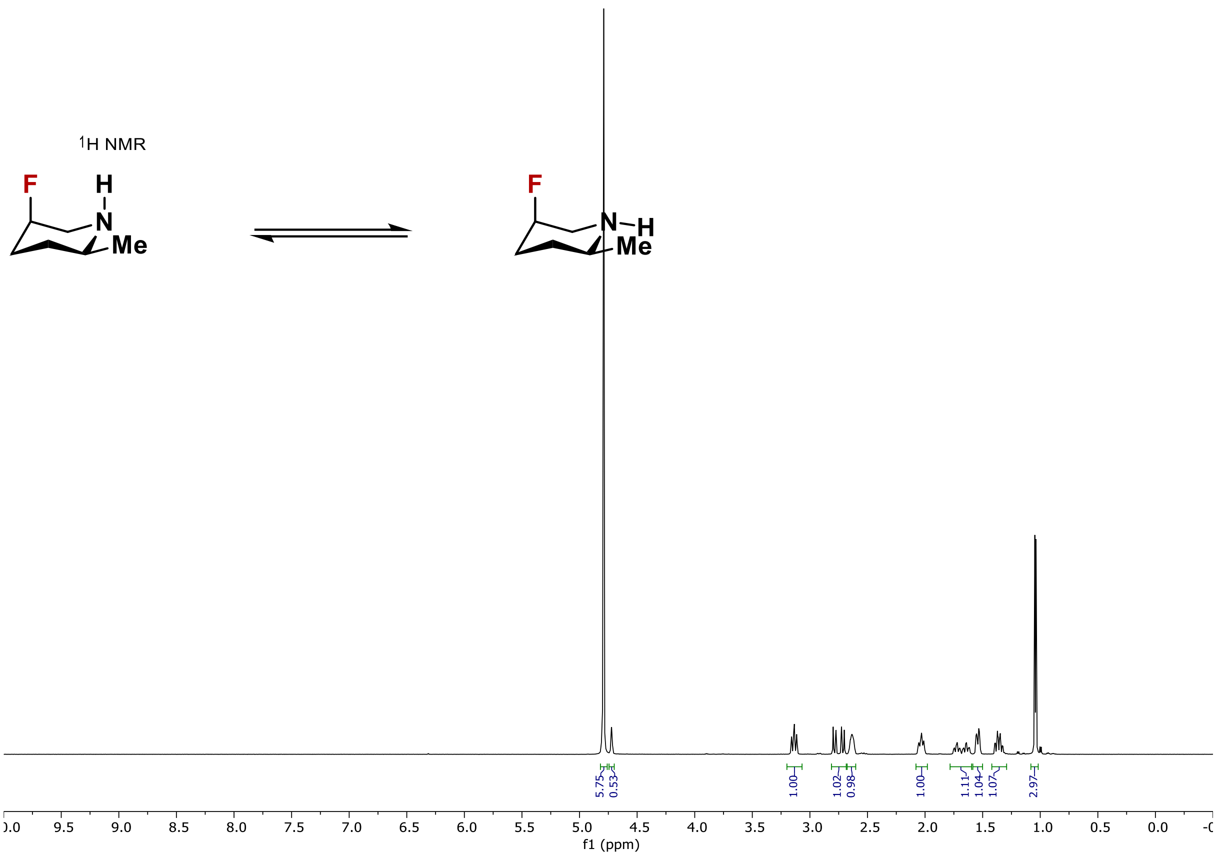
1H NMR
F
H
N
Me
F
N
H
Me
5.75
0.53
1.00
1.02
0.98
1.00
1.11
1.04
1.07
2.97
f1 (ppm)

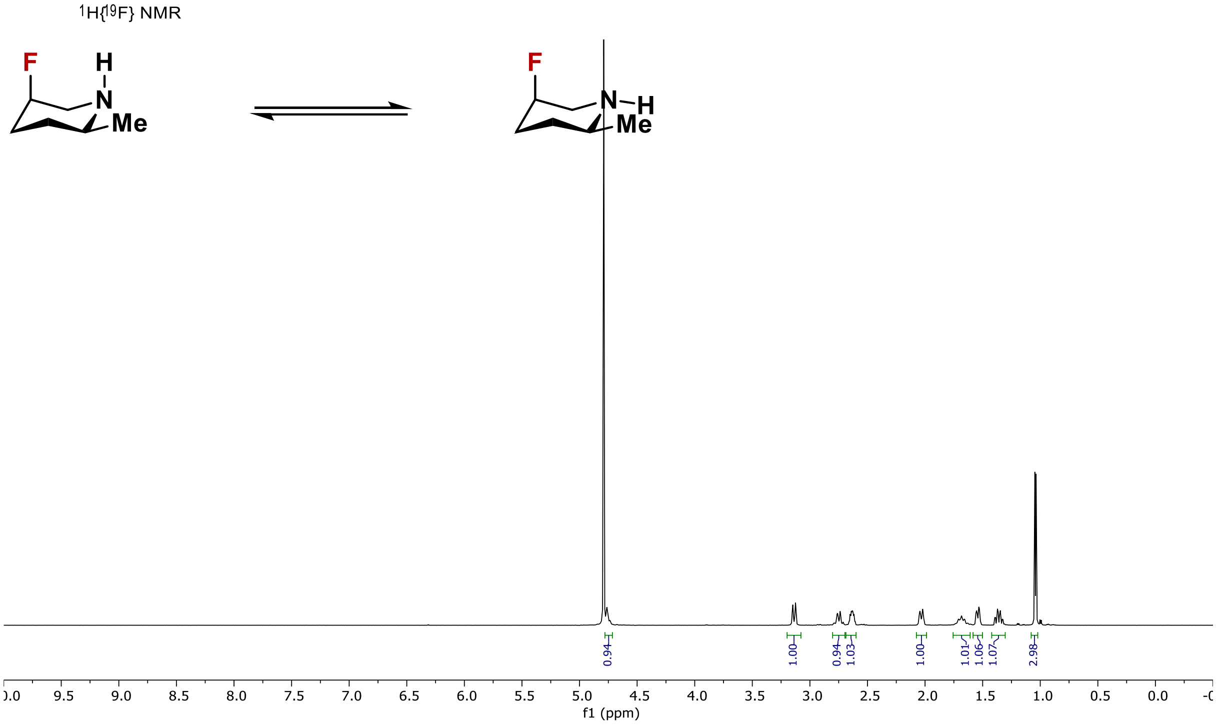
1H{19F} NMR
F
H
N
Me
F
N
H
Me
0.94
1.00
0.94
1.03
1.00
1.01
1.06
1.07
2.98
f1 (ppm)

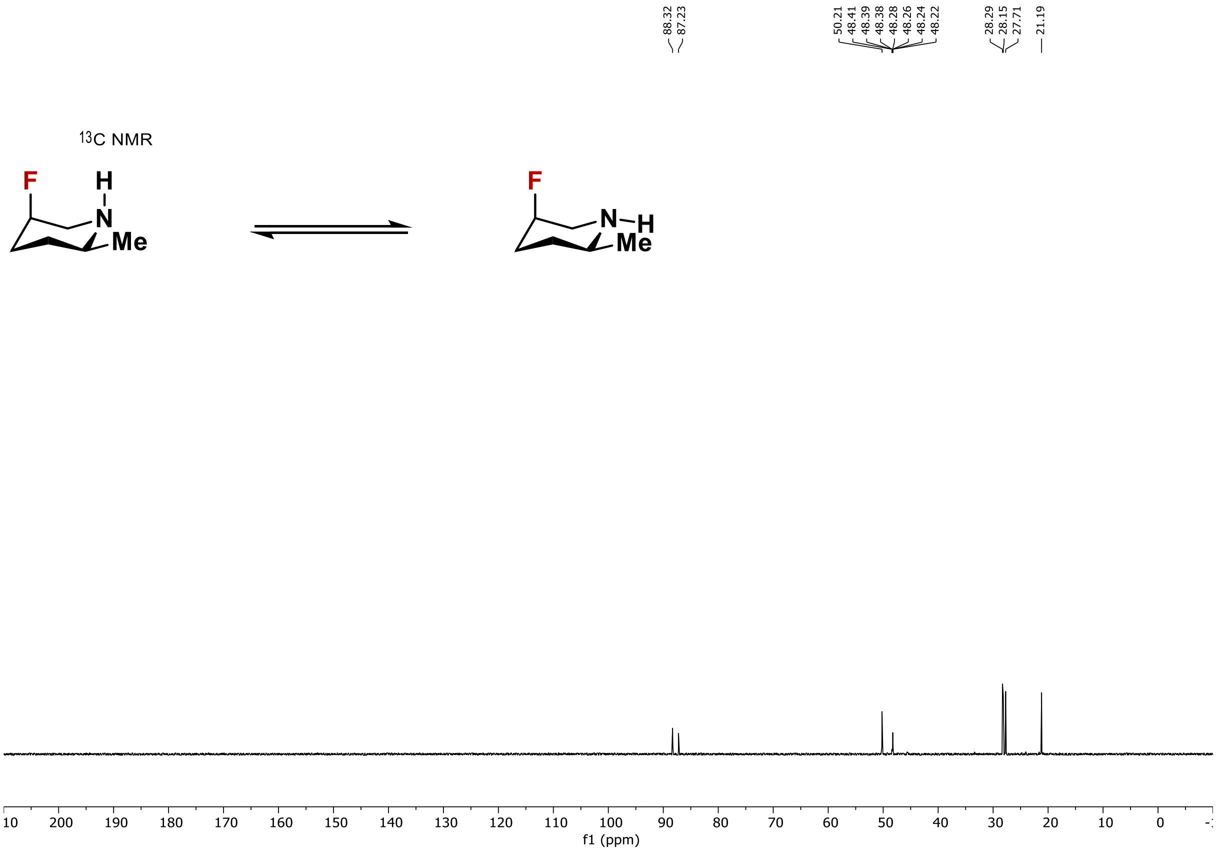

88.32
87.23
50.21
48.41
48.39
48.38
48.28
48.26
48.24
48.22
28.29
28.15
27.71
21.19
13C NMR
F
H
N
Me
F
N-H
Me
f1 (ppm)

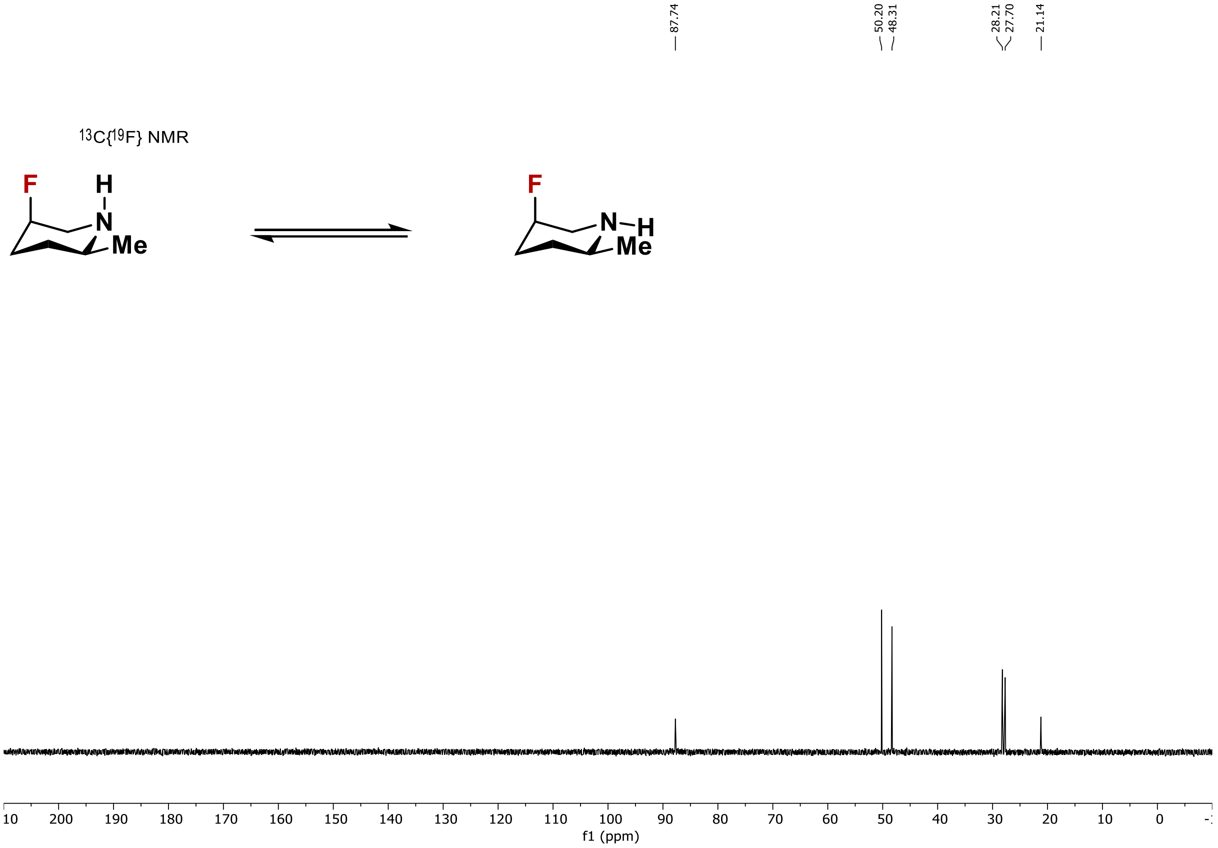

87.74
50.20
48.31
28.21
27.70
21.14
13C{19F} NMR
F
H
N
Me
F
N-H
Me
f1 (ppm)

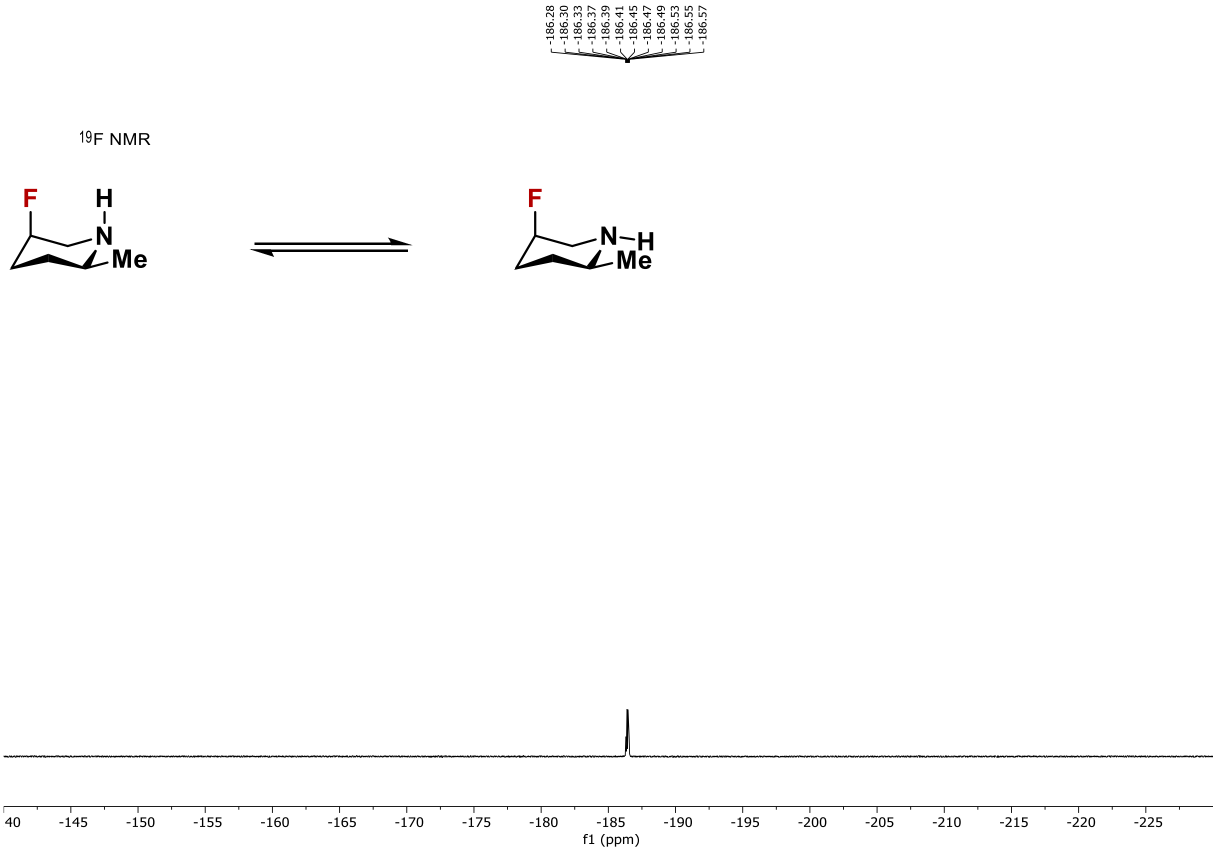

-186.28
-186.30
-186.33
-186.37
-186.39
-186.41
-186.45
-186.47
-186.49
-186.53
-186.55
-186.57
19F NMR
F
H
N
Me
F
N
H
Me
40
-145
-150
-155
-160
-165
-170
-175
-180
-185
-190
-195
-200
-205
-210
-215
-220
-225
f1 (ppm)

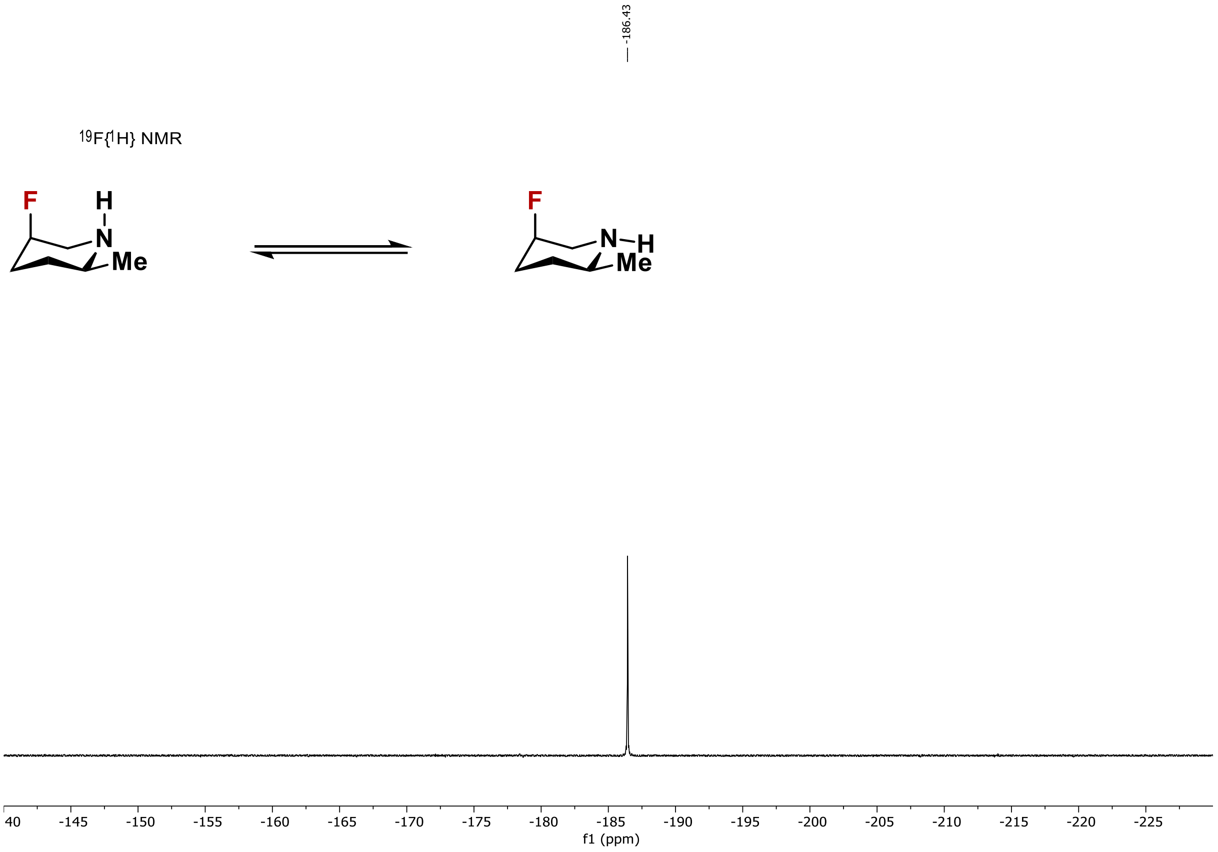

-186.43
19F{1H} NMR
F
H
N
Me
F
N
H
Me
40
-145
-150
-155
-160
-165
-170
-175
-180
-185
-190
-195
-200
-205
-210
-215
-220
-225
f1 (ppm)

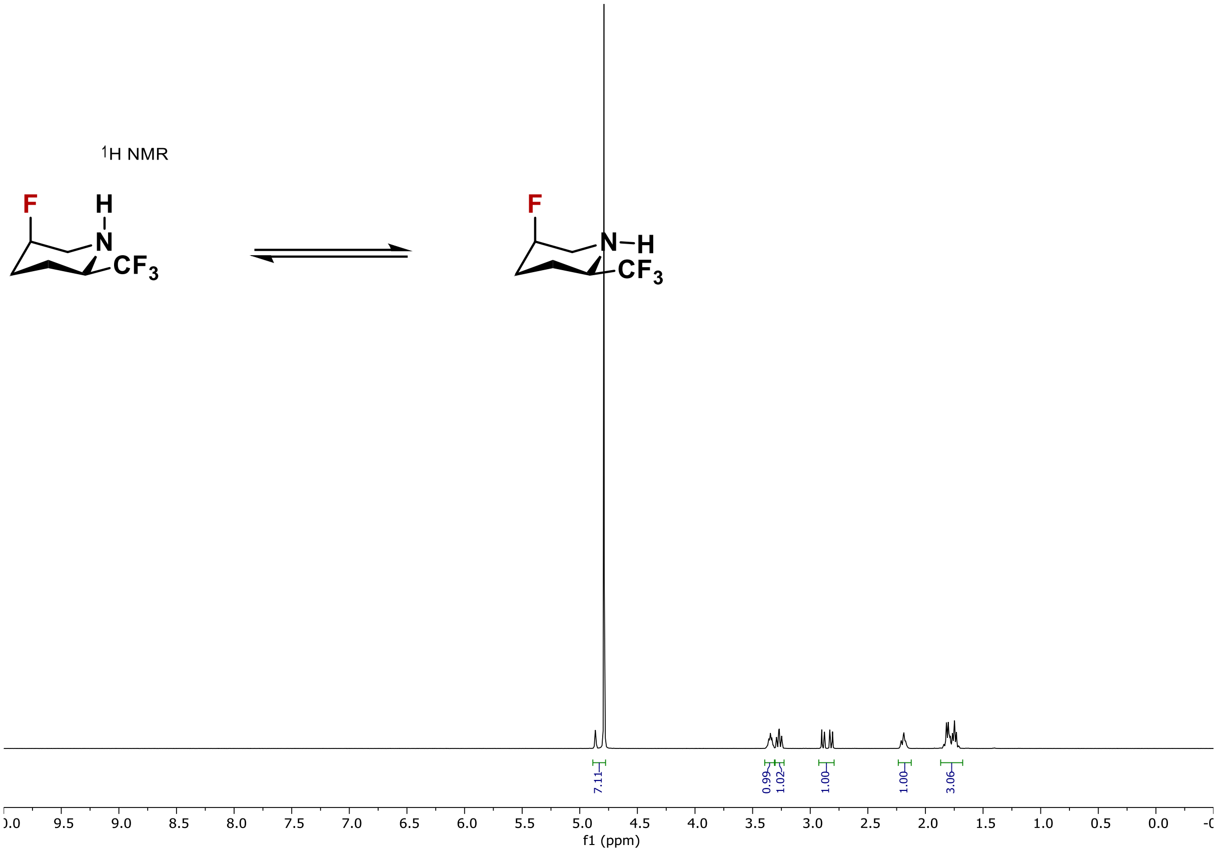
1H NMR
F
H
N
CF3
N–H
7.11
0.99
1.02
1.00
1.00
3.06
9.5
9.0
8.5
8.0
7.5
7.0
6.5
6.0
5.5
5.0
4.5
4.0
3.5
3.0
2.5
2.0
1.5
1.0
0.5
0.0
f1 (ppm)

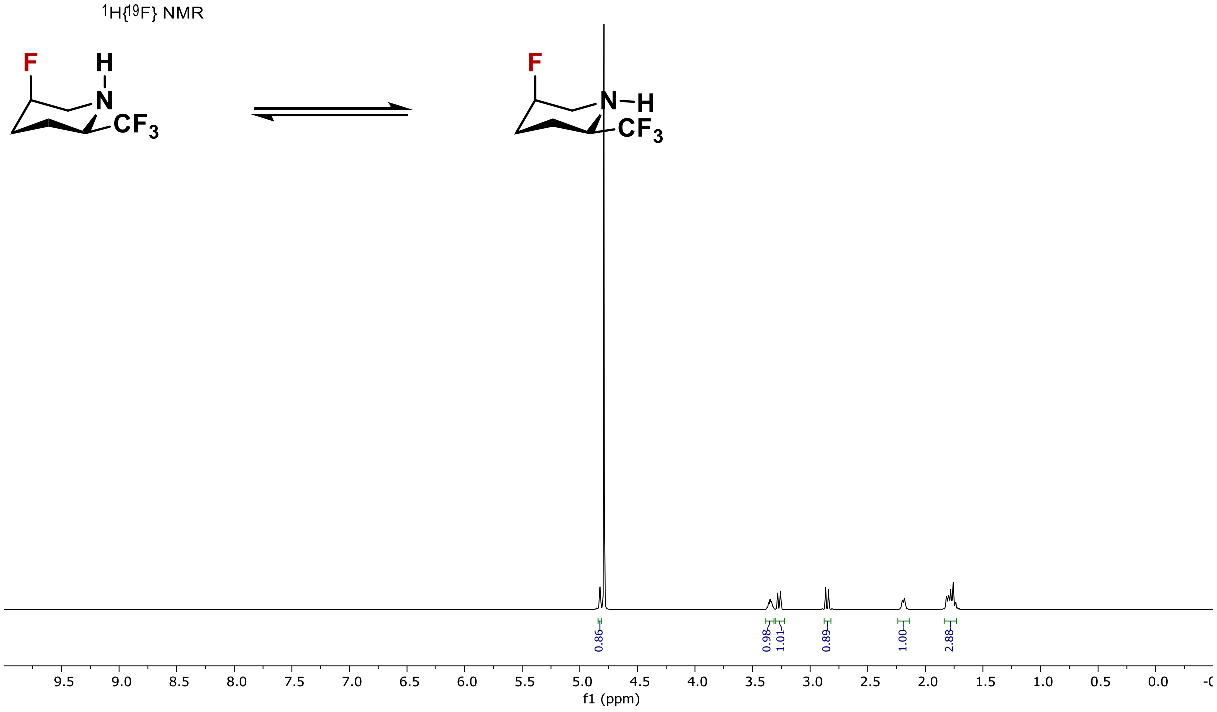
1H{19F} NMR
F
H
N
CF3
N–H
0.86
0.98
1.01
0.89
1.00
2.88
9.5
9.0
8.5
8.0
7.5
7.0
6.5
6.0
5.5
5.0
4.5
4.0
3.5
3.0
2.5
2.0
1.5
1.0
0.5
0.0
f1 (ppm)

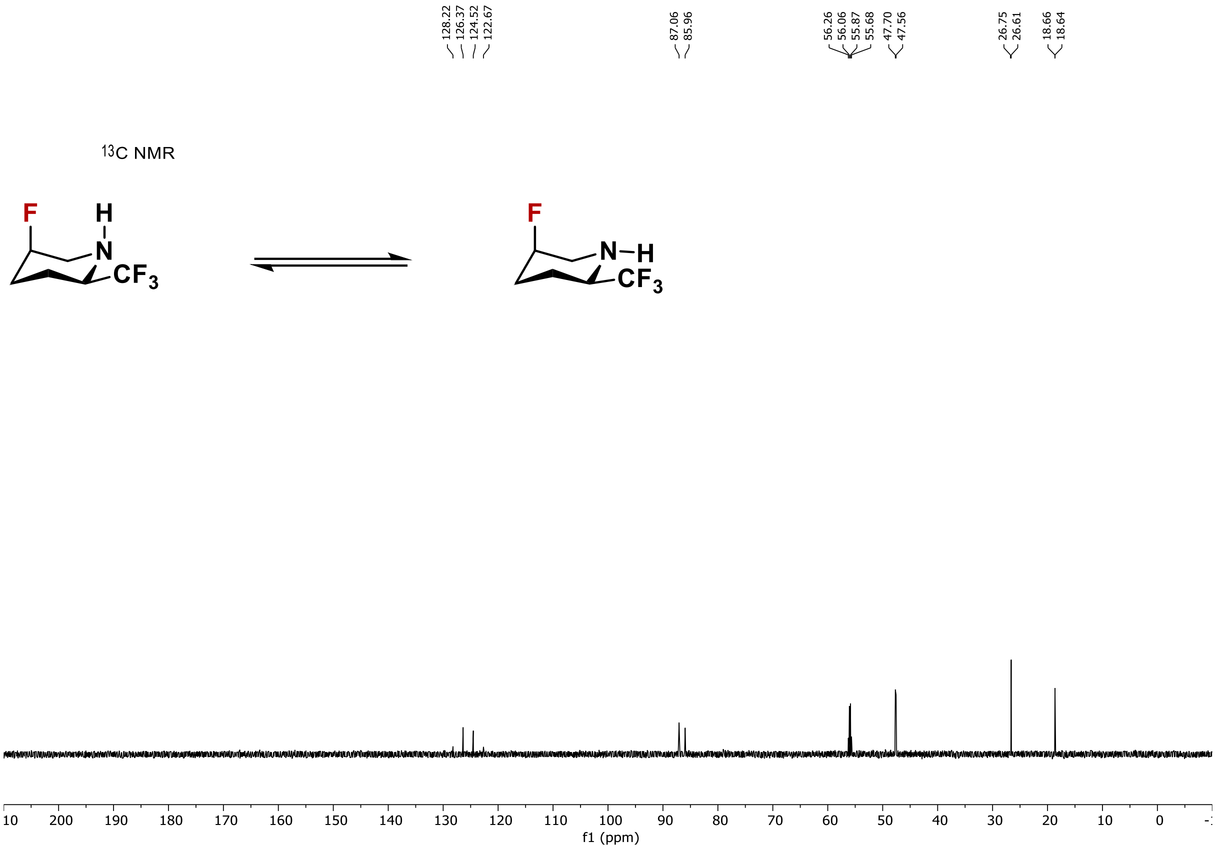
128.22
126.37
124.52
122.67
87.06
85.96
56.26
56.06
55.87
55.68
47.70
47.56
26.75
26.61
18.66
18.64
13C NMR
F
H
N
CF_3
F
N–H
CF_3
f1 (ppm)

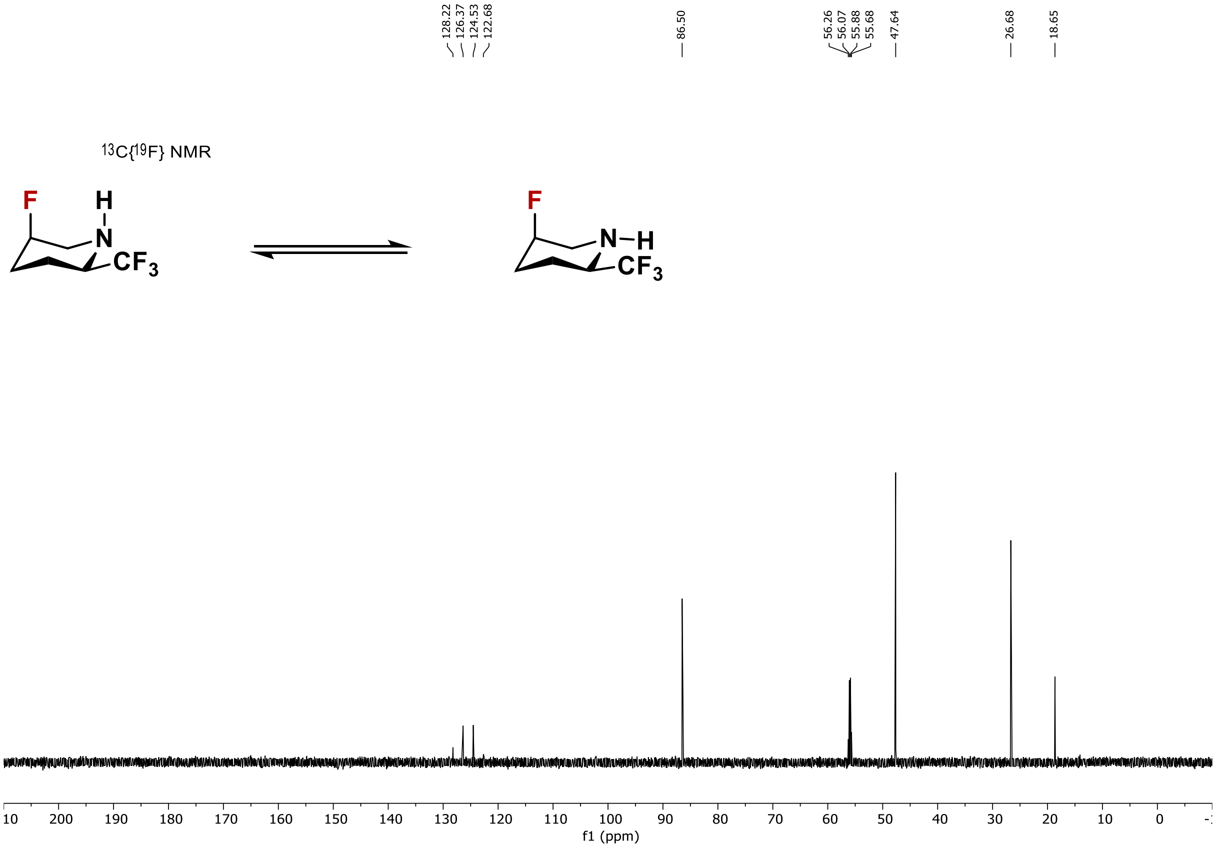
128.22
126.37
124.53
122.68
86.50
56.26
56.07
55.88
55.68
47.64
26.68
18.65
13C{19F} NMR
F
H
N
CF_3
F
N–H
CF_3
f1 (ppm)

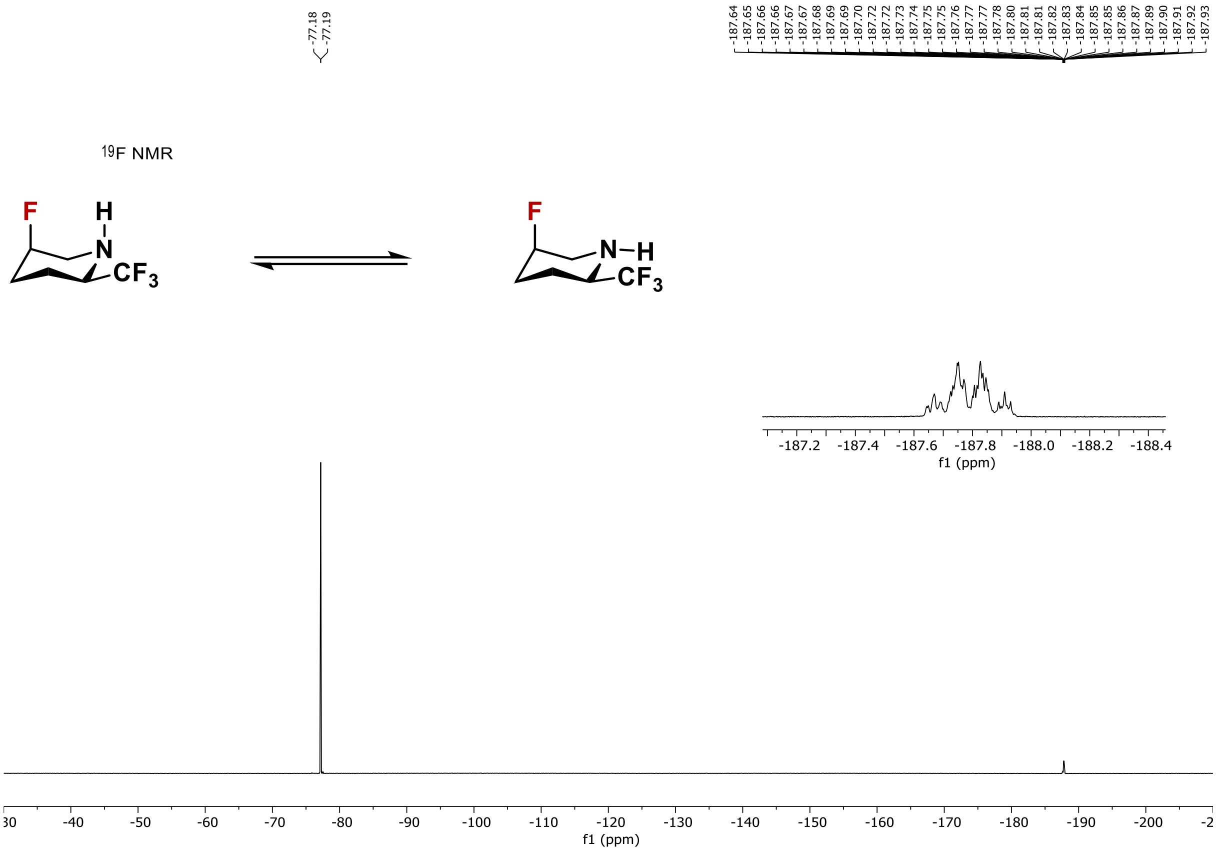
-77.18
-77.19
-187.64 -187.65 -187.66 -187.66 -187.67 -187.67 -187.68 -187.69 -187.69 -187.70 -187.72 -187.72 -187.73 -187.74 -187.75 -187.75 -187.76 -187.77 -187.77 -187.78 -187.80 -187.81 -187.81 -187.82 -187.83 -187.84 -187.85 -187.85 -187.86 -187.87 -187.89 -187.90 -187.91 -187.92 -187.93
19F NMR
F
H
N
CF3
N–H
-187.2 -187.4 -187.6 -187.8 -188.0 -188.2 -188.4
f1 (ppm)
-40 -50 -60 -70 -80 -90 -100 -110 -120 -130 -140 -150 -160 -170 -180 -190 -200
f1 (ppm)

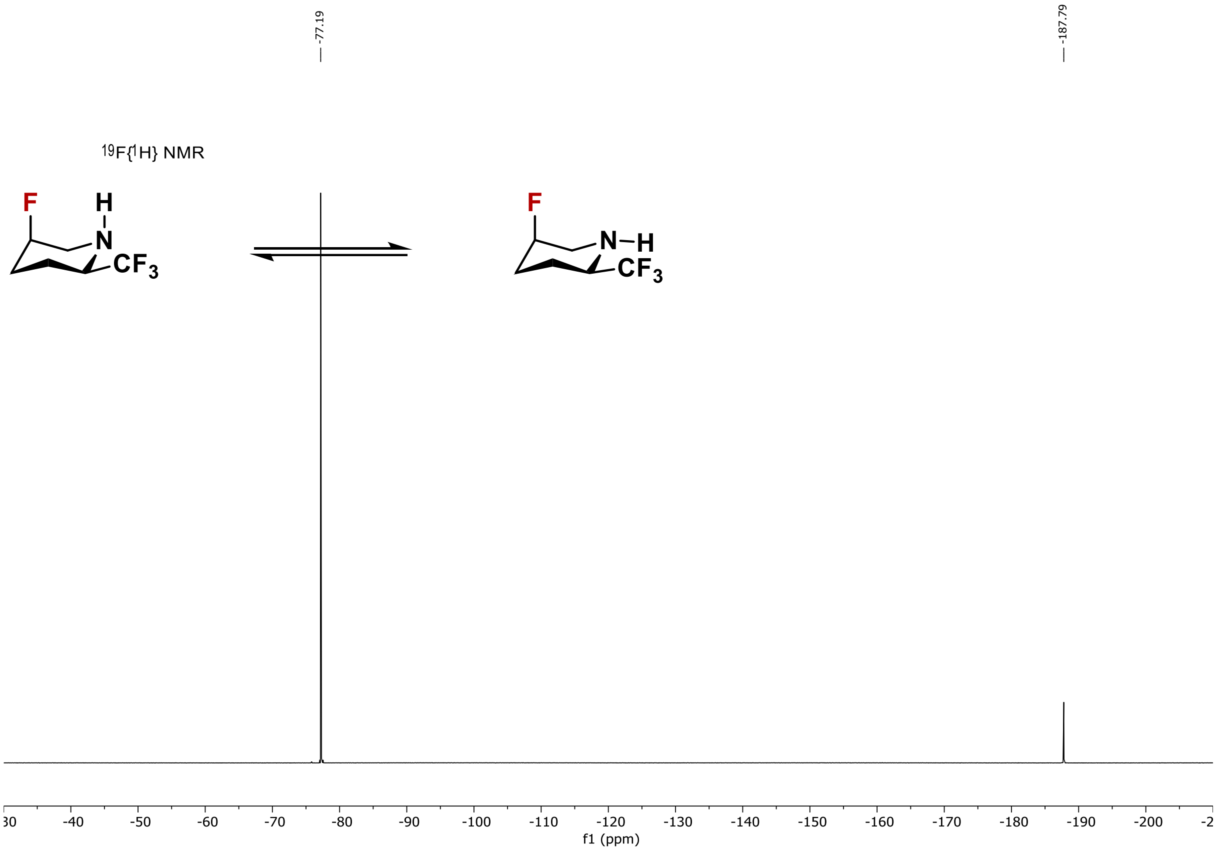
-77.19
-187.79
19F{1H} NMR
F
H
N
CF3
N–H
-40 -50 -60 -70 -80 -90 -100 -110 -120 -130 -140 -150 -160 -170 -180 -190 -200
f1 (ppm)

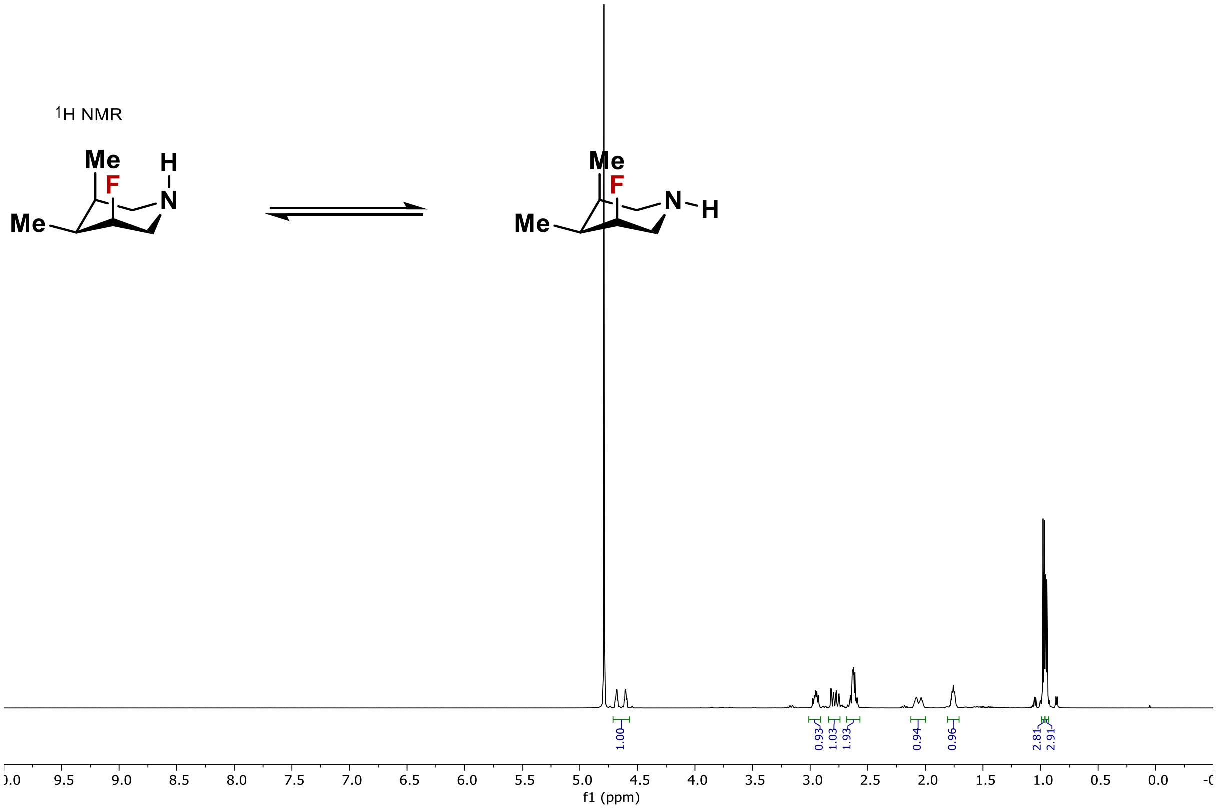

1H NMR
Me
F
H
N
Me
Me
F
N
H
Me
1.00
0.93
1.03
1.93
0.94
0.96
2.81
2.91
f1 (ppm)

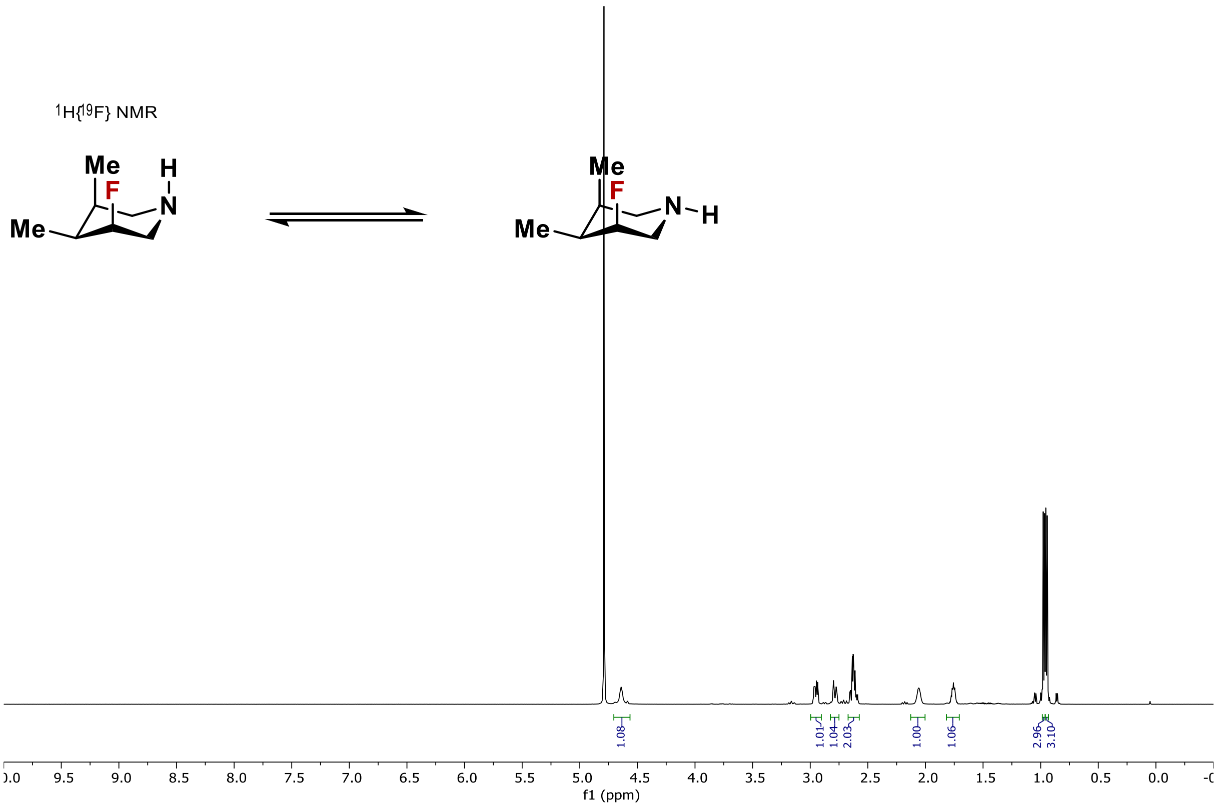

1H{19F} NMR
Me
F
H
N
Me
Me
F
N
H
Me
1.08
1.01
1.04
2.03
1.00
1.06
2.96
3.10
f1 (ppm)

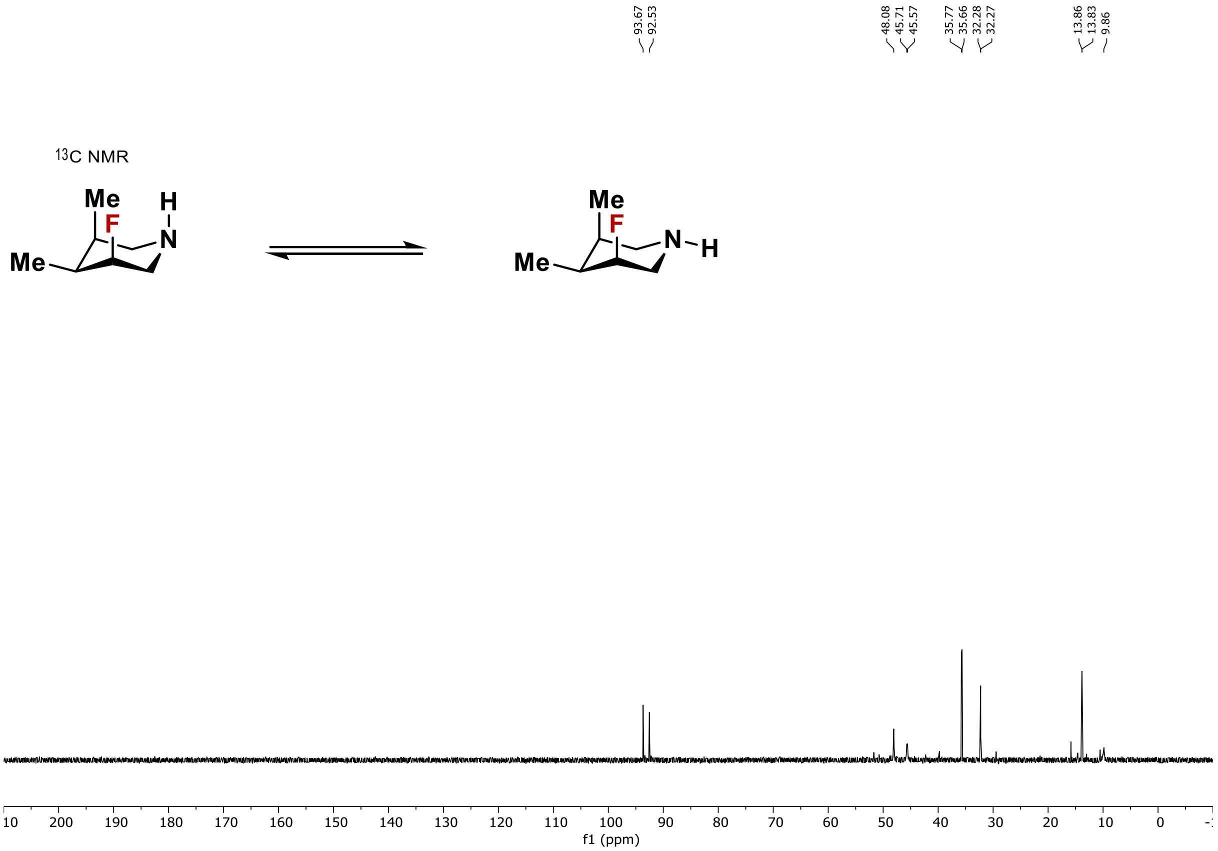
93.67
92.53
48.08
45.71
45.57
35.77
35.66
32.28
32.27
13.86
13.83
9.86
13C NMR
Me
H
F
N
Me
Me
F
N
H
Me
10
200
190
180
170
160
150
140
130
120
110
100
90
80
70
60
50
40
30
20
10
0
f1 (ppm)

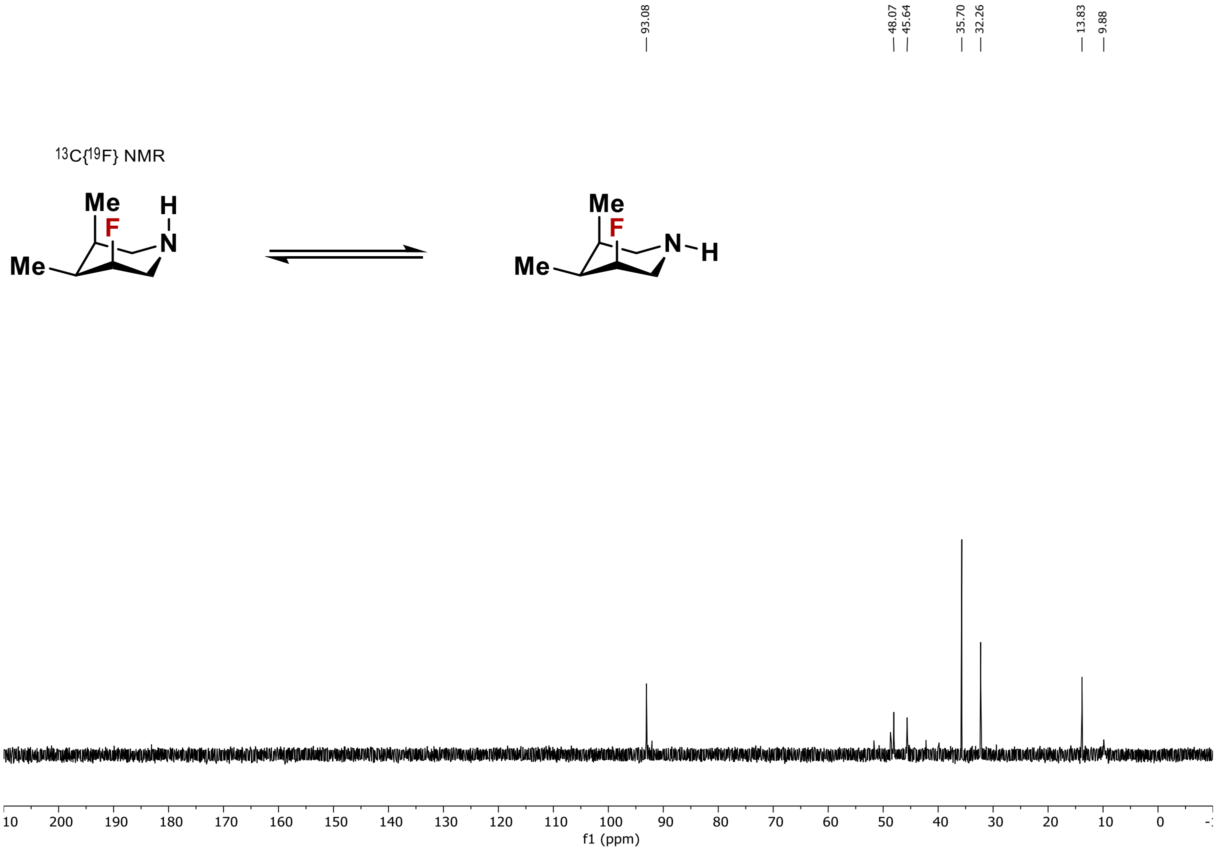
93.08
48.07
45.64
35.70
32.26
13.83
9.88
13C{19F} NMR
Me
H
F
N
Me
Me
F
N
H
Me
10
200
190
180
170
160
150
140
130
120
110
100
90
80
70
60
50
40
30
20
10
0
f1 (ppm)

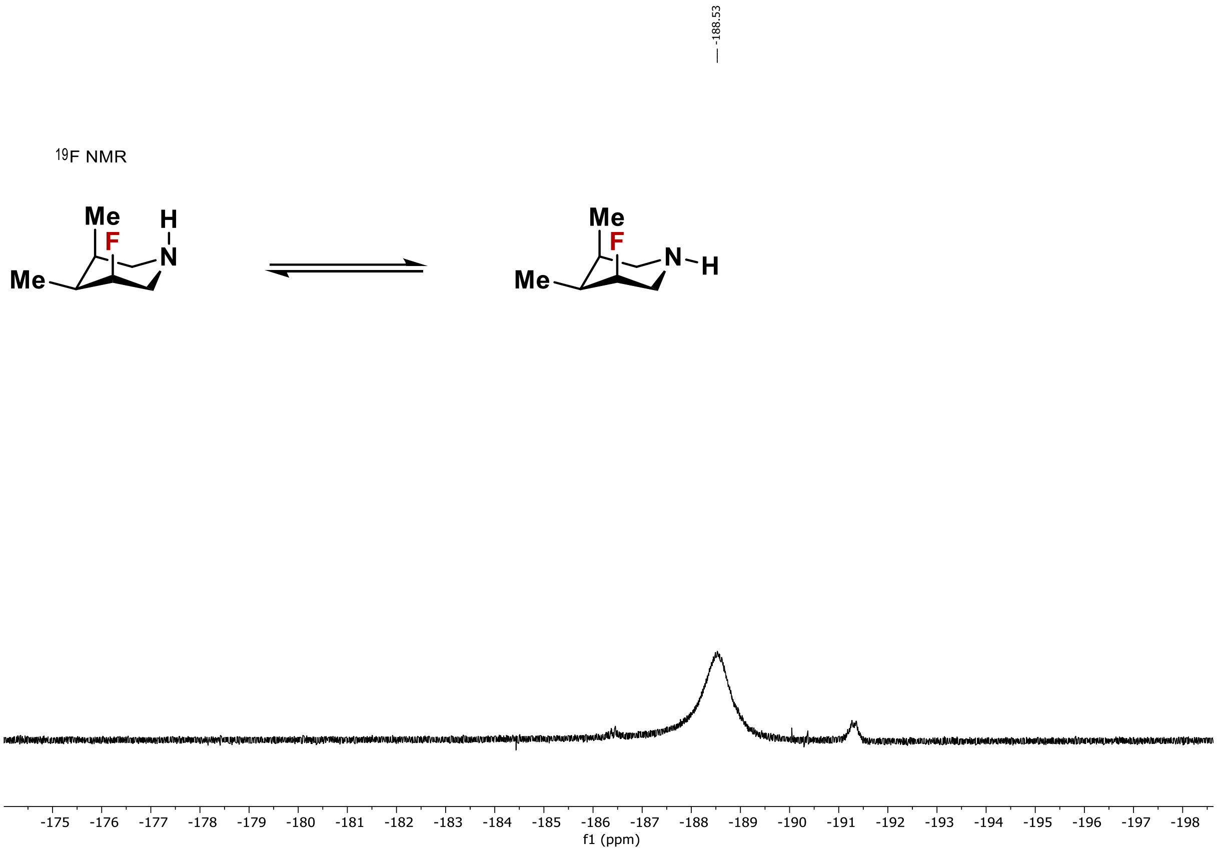
-188.53
19F NMR
Me
F
H
N
Me
Me
F
N
H
Me
-175 -176 -177 -178 -179 -180 -181 -182 -183 -184 -185 -186 -187 -188 -189 -190 -191 -192 -193 -194 -195 -196 -197 -198
f1 (ppm)

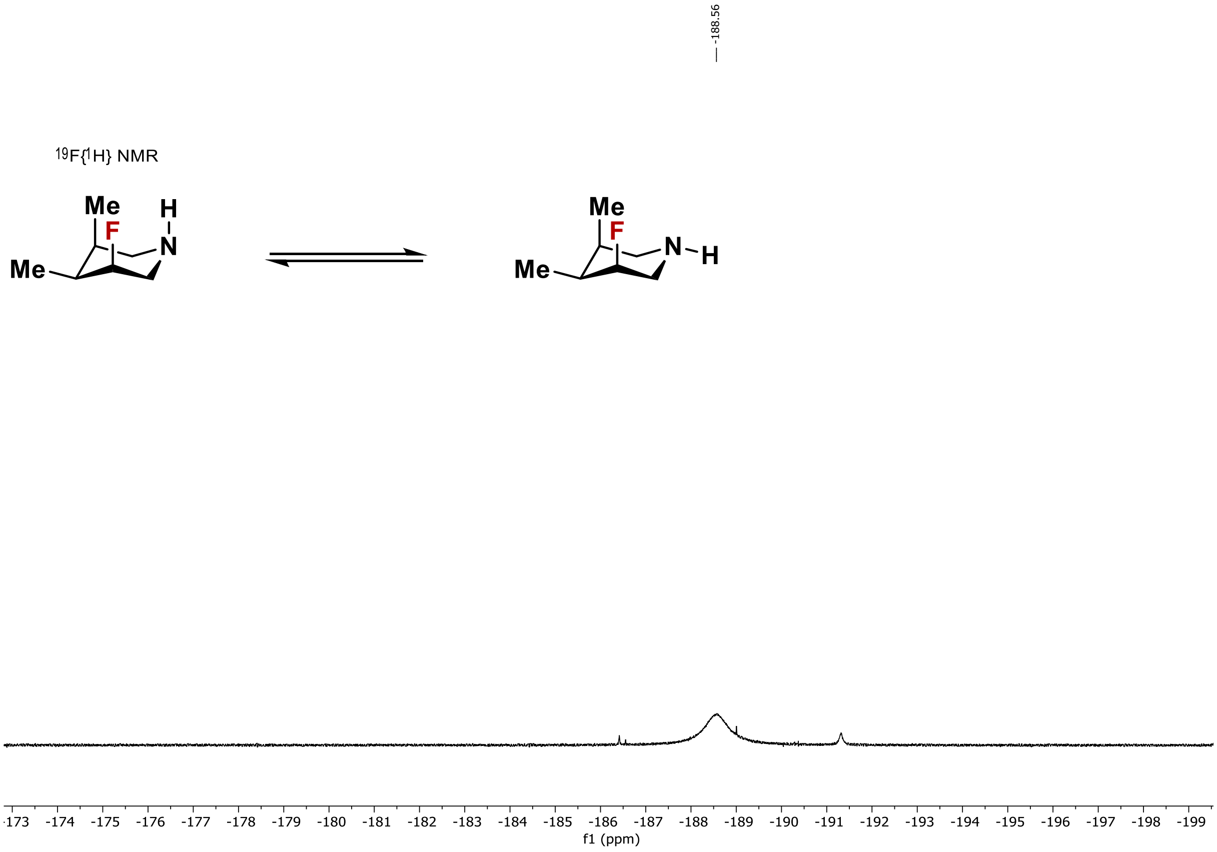
-188.56
19F{1H} NMR
Me
F
H
N
Me
Me
F
N
H
Me
-173 -174 -175 -176 -177 -178 -179 -180 -181 -182 -183 -184 -185 -186 -187 -188 -189 -190 -191 -192 -193 -194 -195 -196 -197 -198 -199
f1 (ppm)

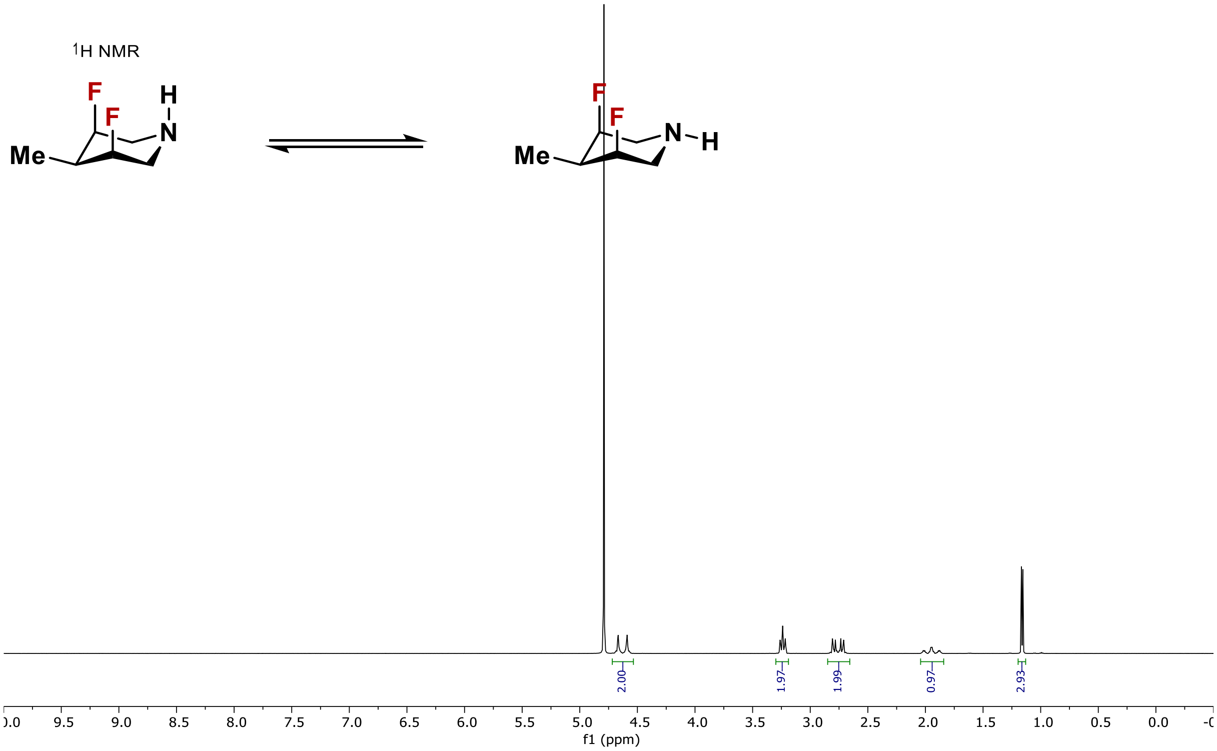

^{1}H NMR
F
F
H
N
Me
F
F
N
H
Me
2.00
1.97
1.99
0.97
2.93
9.5 9.0 8.5 8.0 7.5 7.0 6.5 6.0 5.5 5.0 4.5 4.0 3.5 3.0 2.5 2.0 1.5 1.0 0.5 0.0
f1 (ppm)

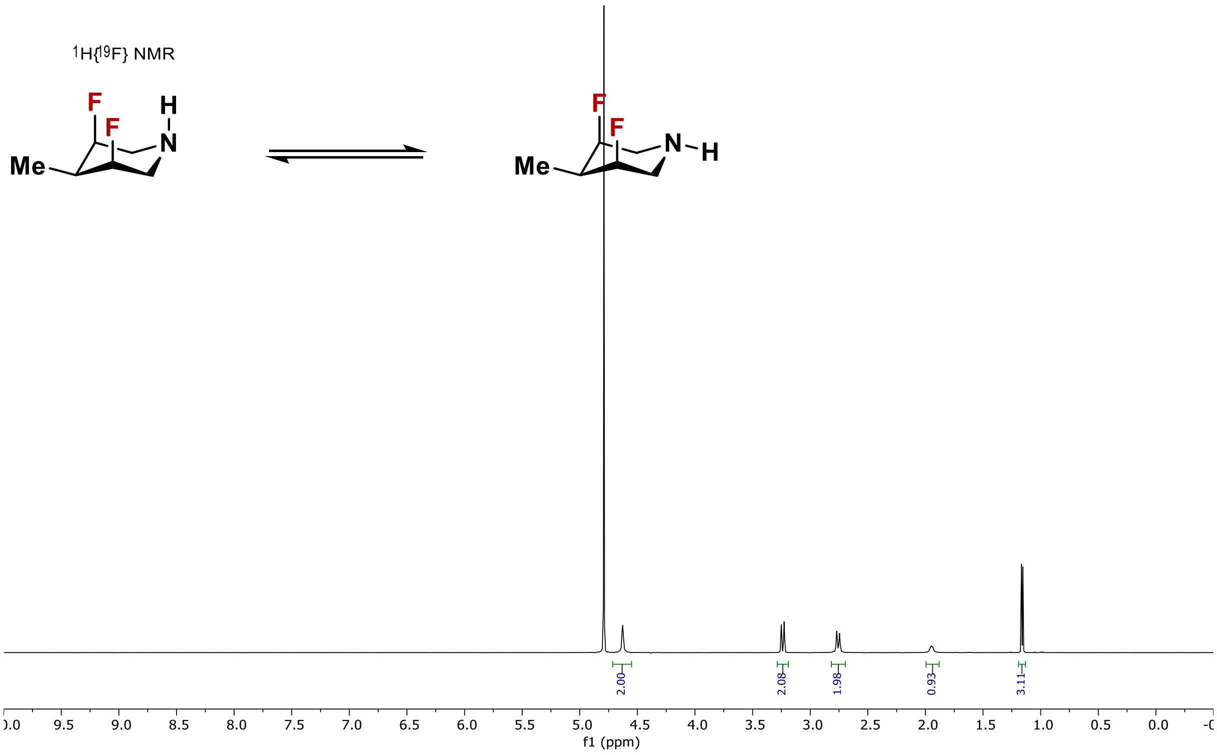

^{1}H{^{19}F} NMR
F
F
H
N
Me
F
F
N
H
Me
2.00
2.08
1.98
0.93
3.11
9.5 9.0 8.5 8.0 7.5 7.0 6.5 6.0 5.5 5.0 4.5 4.0 3.5 3.0 2.5 2.0 1.5 1.0 0.5 0.0
f1 (ppm)

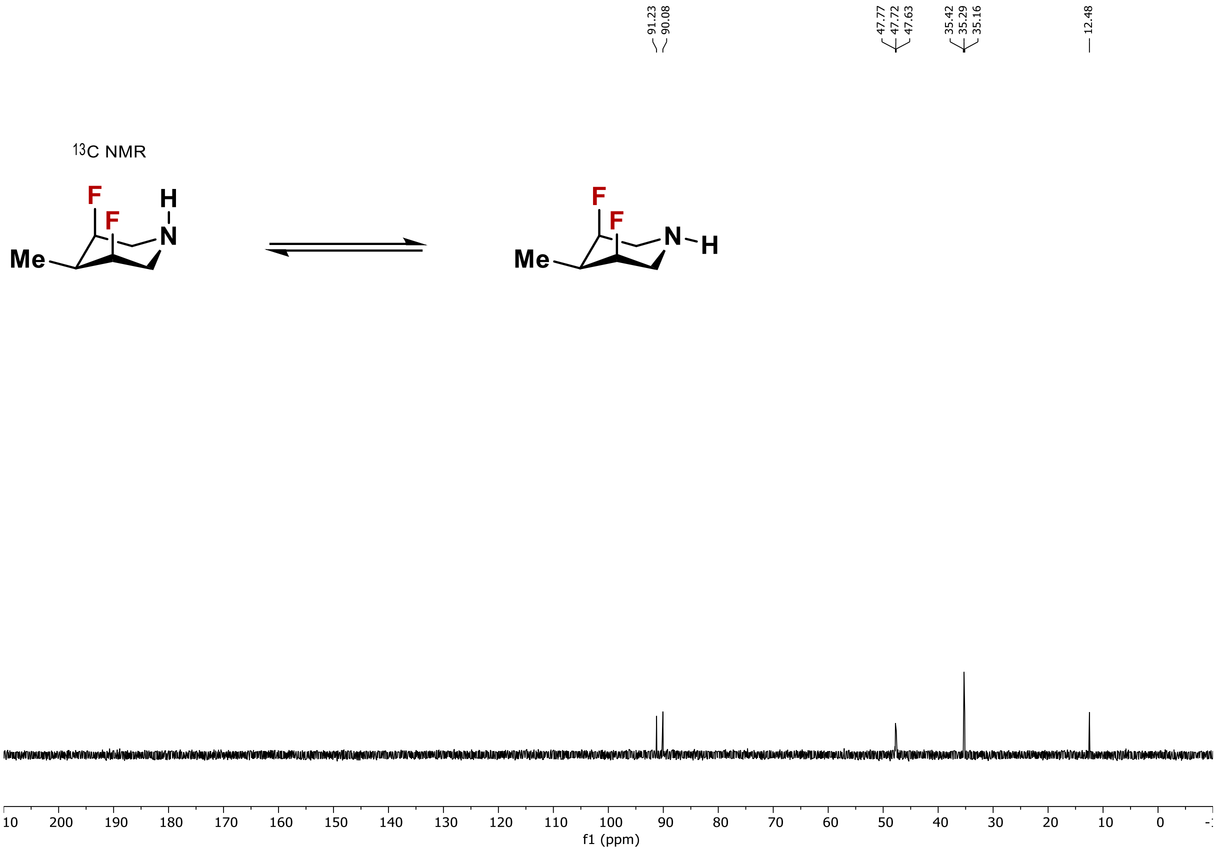

91.23
90.08
47.77
47.72
47.63
35.42
35.29
35.16
12.48
^{13}C NMR
F
F
H
N
Me
F
F
N
H
Me
10 200 190 180 170 160 150 140 130 120 110 100 90 80 70 60 50 40 30 20 10 0
f1 (ppm)

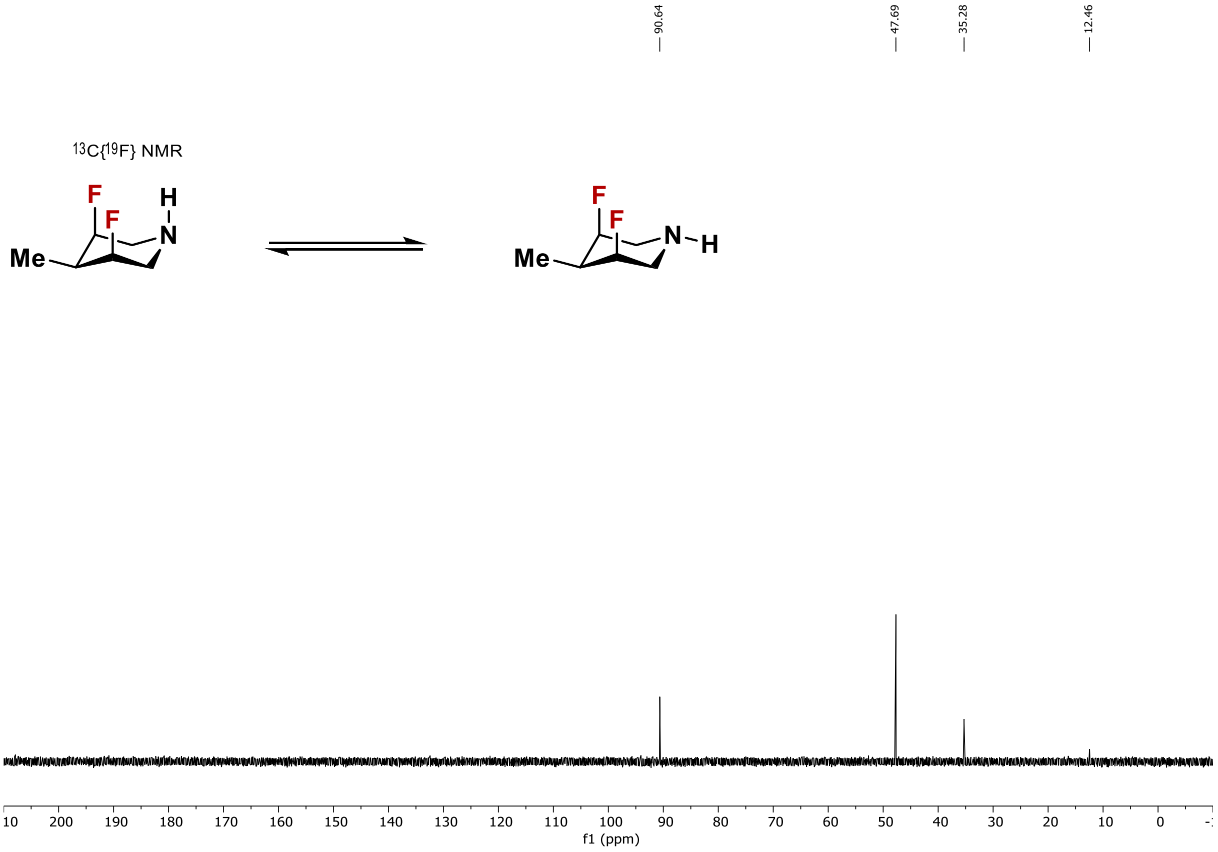

90.64
47.69
35.28
12.46
$^{13}C\{^{19}F\}$ NMR
F
F
H
N
Me
F
F
N
H
Me
10 200 190 180 170 160 150 140 130 120 110 100 90 80 70 60 50 40 30 20 10 0
f1 (ppm)

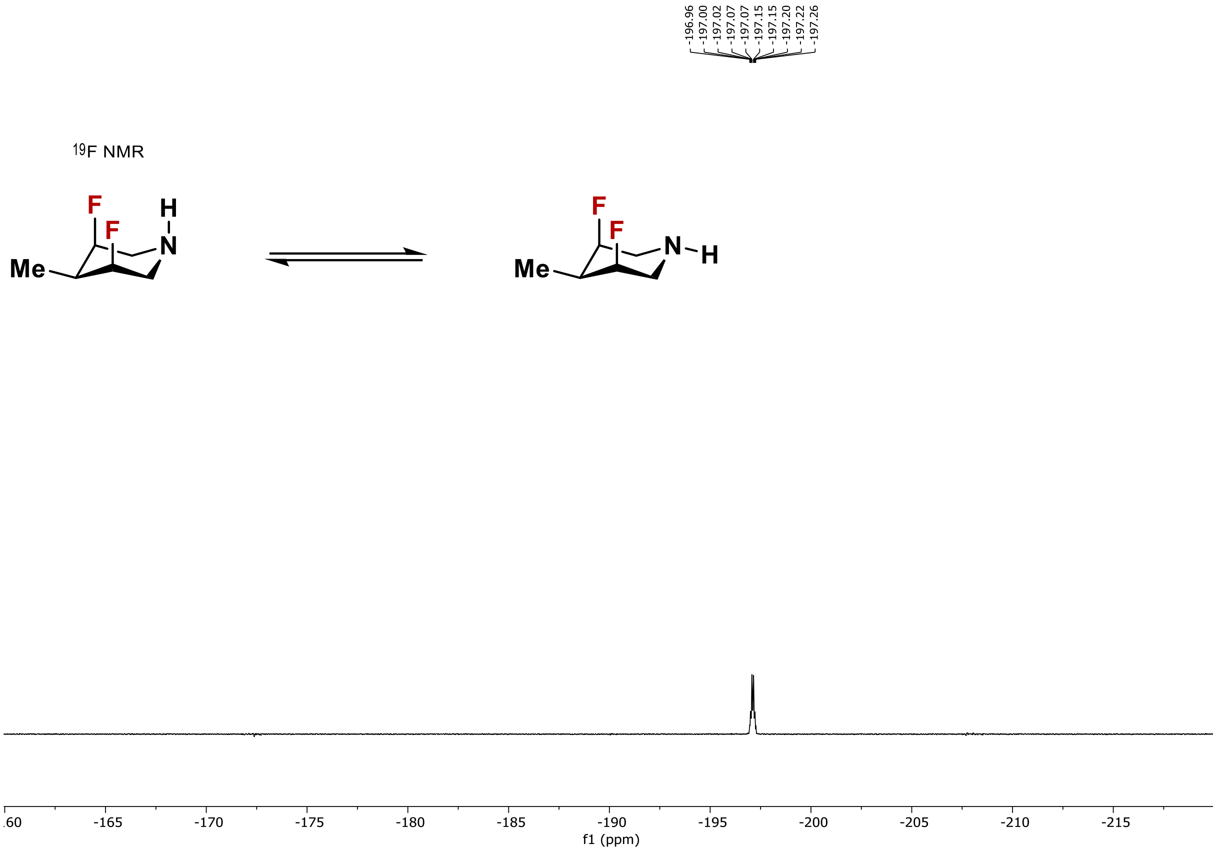
-196.96
-197.00
-197.02
-197.07
-197.07
-197.15
-197.15
-197.20
-197.22
-197.26
19F NMR
F
F
H
N
Me
F
F
N
H
Me
.60
-165
-170
-175
-180
-185
-190
-195
-200
-205
-210
-215
f1 (ppm)

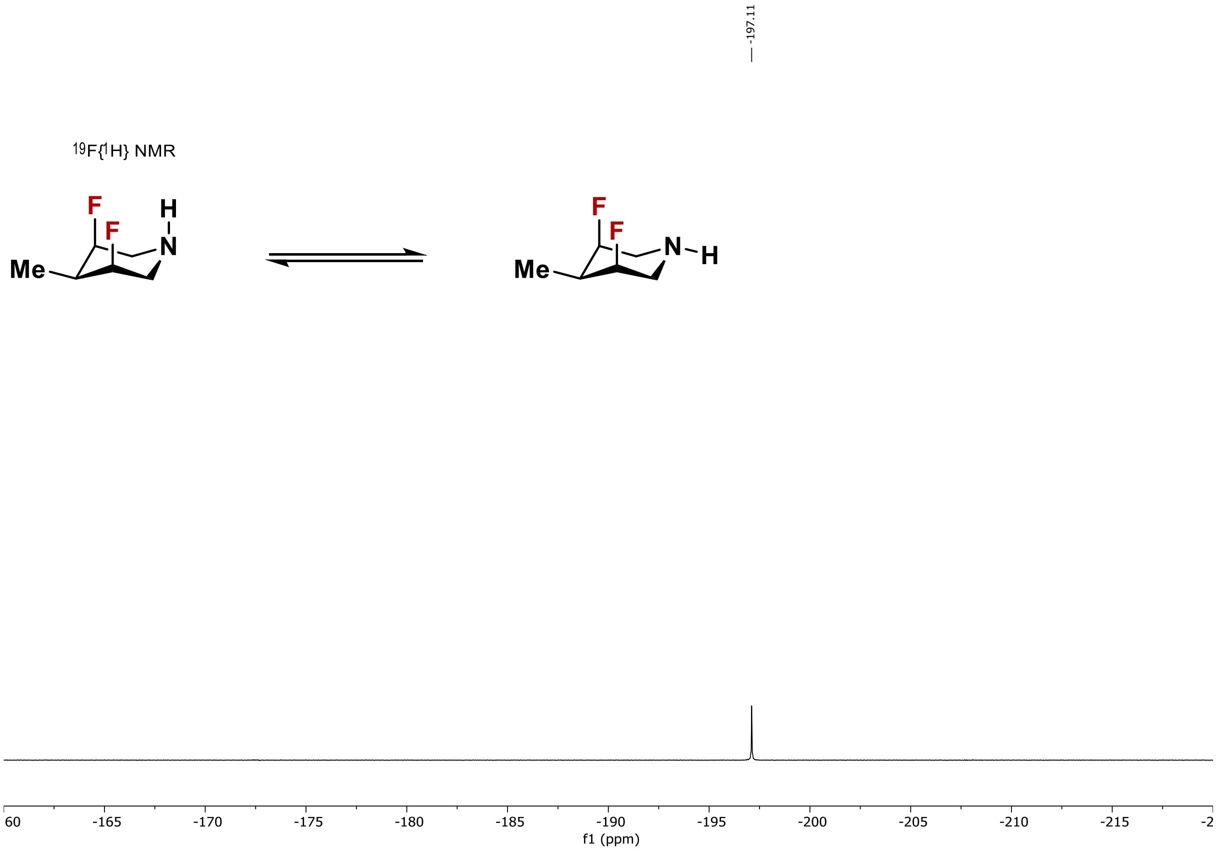
-197.11
19F{1H} NMR
F
F
H
N
Me
F
F
N
H
Me
60
-165
-170
-175
-180
-185
-190
-195
-200
-205
-210
-215
-2
f1 (ppm)

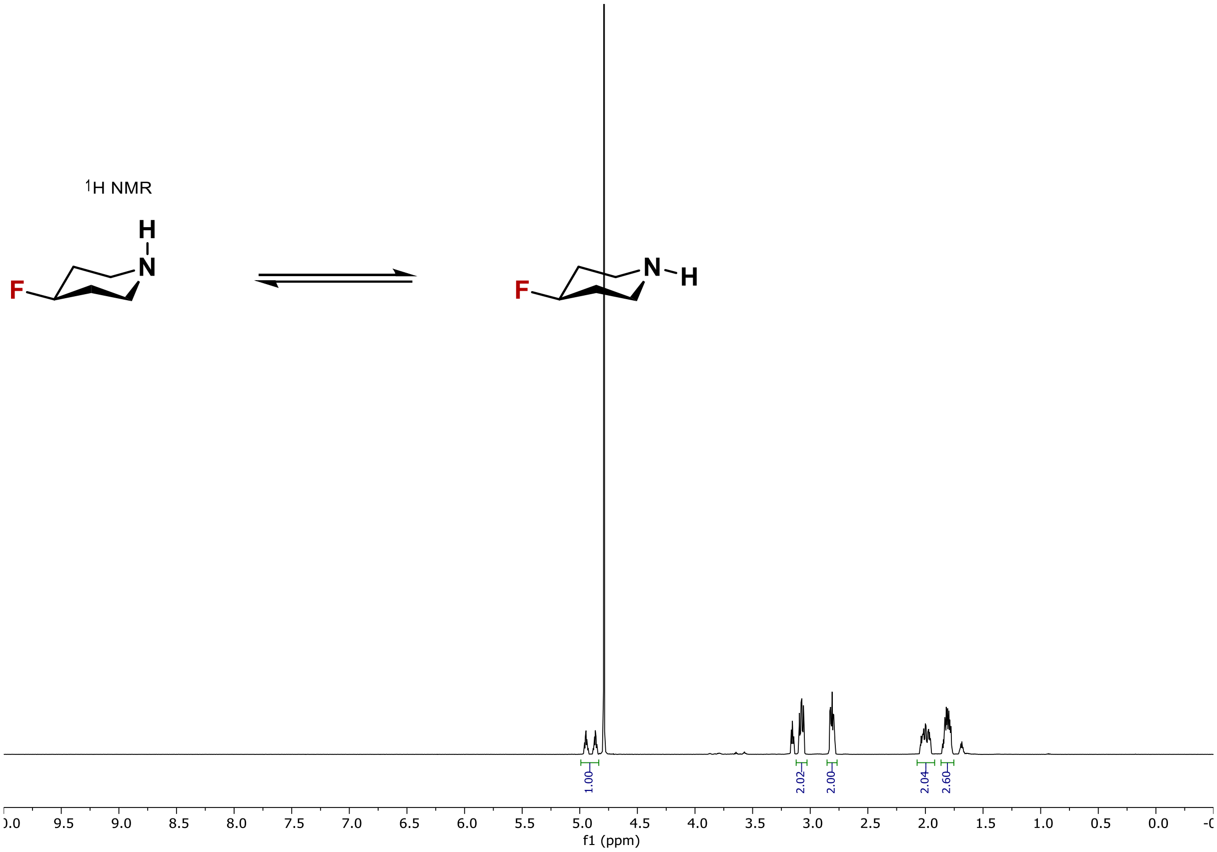

1H NMR
F
H
N
F
N
H
1.00
2.02
2.00
2.04
2.60
0.0
9.5
9.0
8.5
8.0
7.5
7.0
6.5
6.0
5.5
5.0
4.5
4.0
3.5
3.0
2.5
2.0
1.5
1.0
0.5
0.0
f1 (ppm)

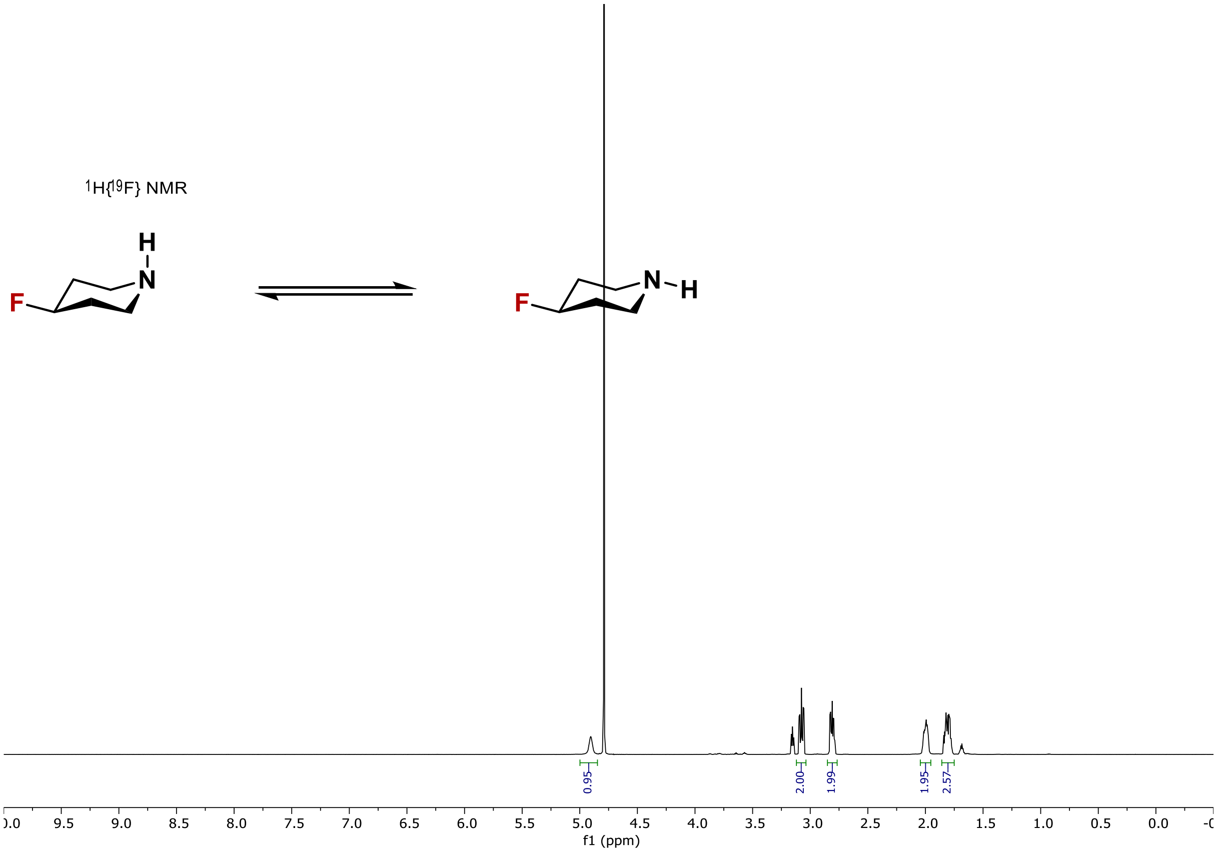

1H{19F} NMR
F
H
N
F
N
H
0.95
2.00
1.99
1.95
2.57
0.0
9.5
9.0
8.5
8.0
7.5
7.0
6.5
6.0
5.5
5.0
4.5
4.0
3.5
3.0
2.5
2.0
1.5
1.0
0.5
0.0
f1 (ppm)

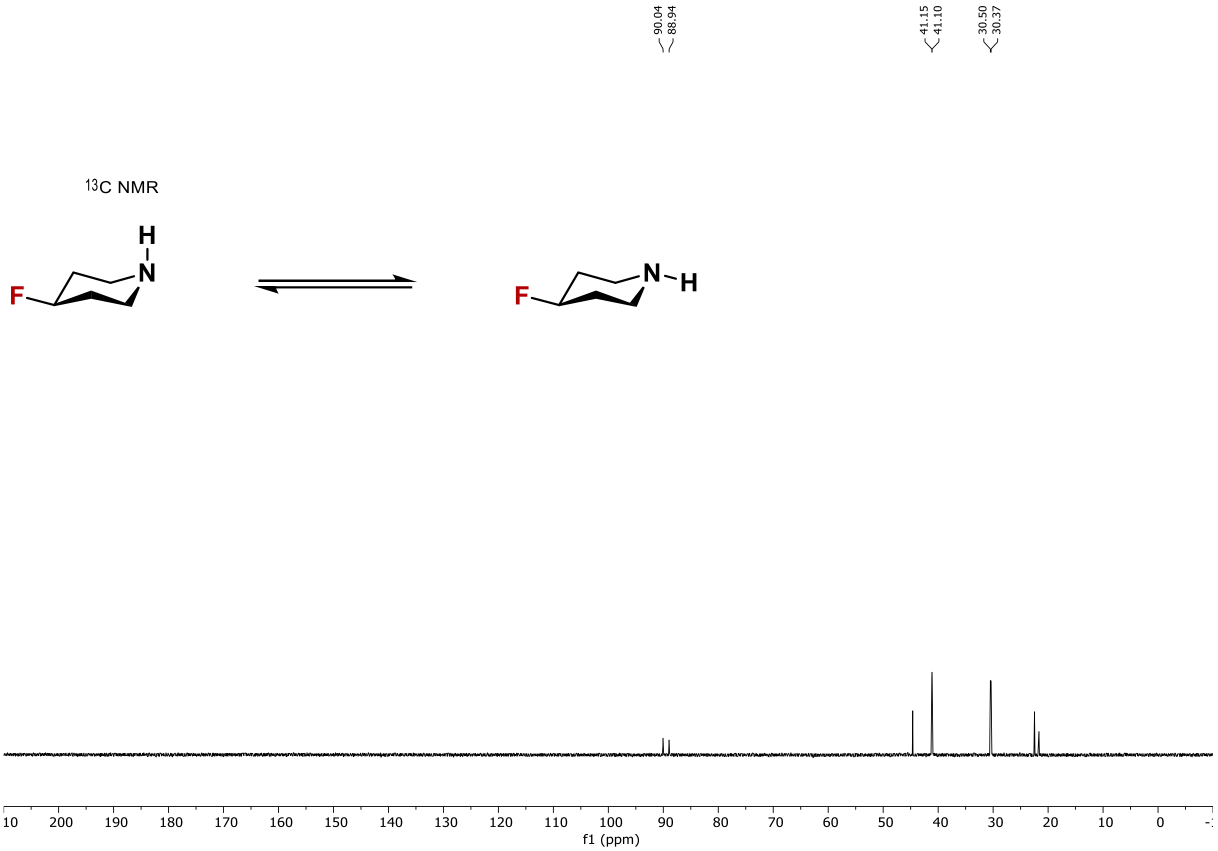

90.04
88.94
41.15
41.10
30.50
30.37
^{13}C NMR
F
N
H
f1 (ppm)

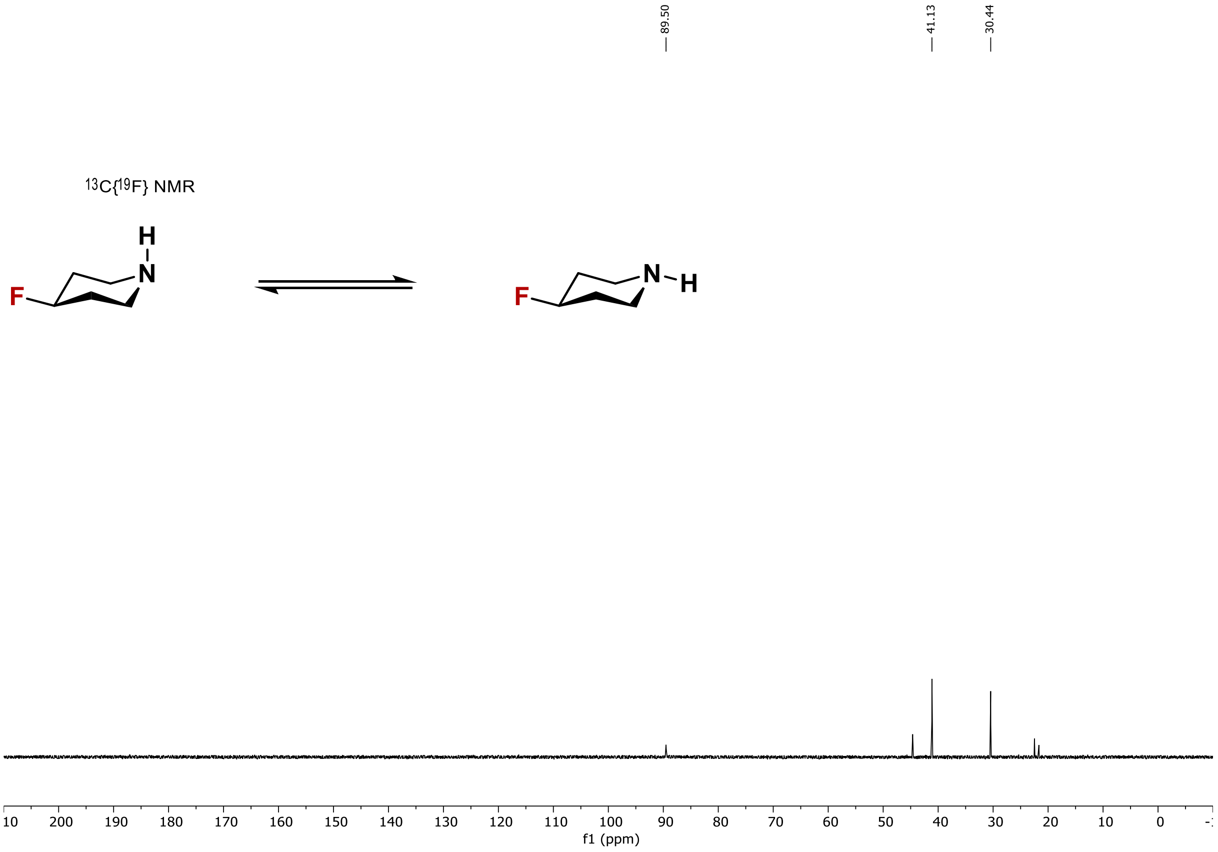

89.50
41.13
30.44
^{13}C{^{19}F} NMR
F
N
H
f1 (ppm)

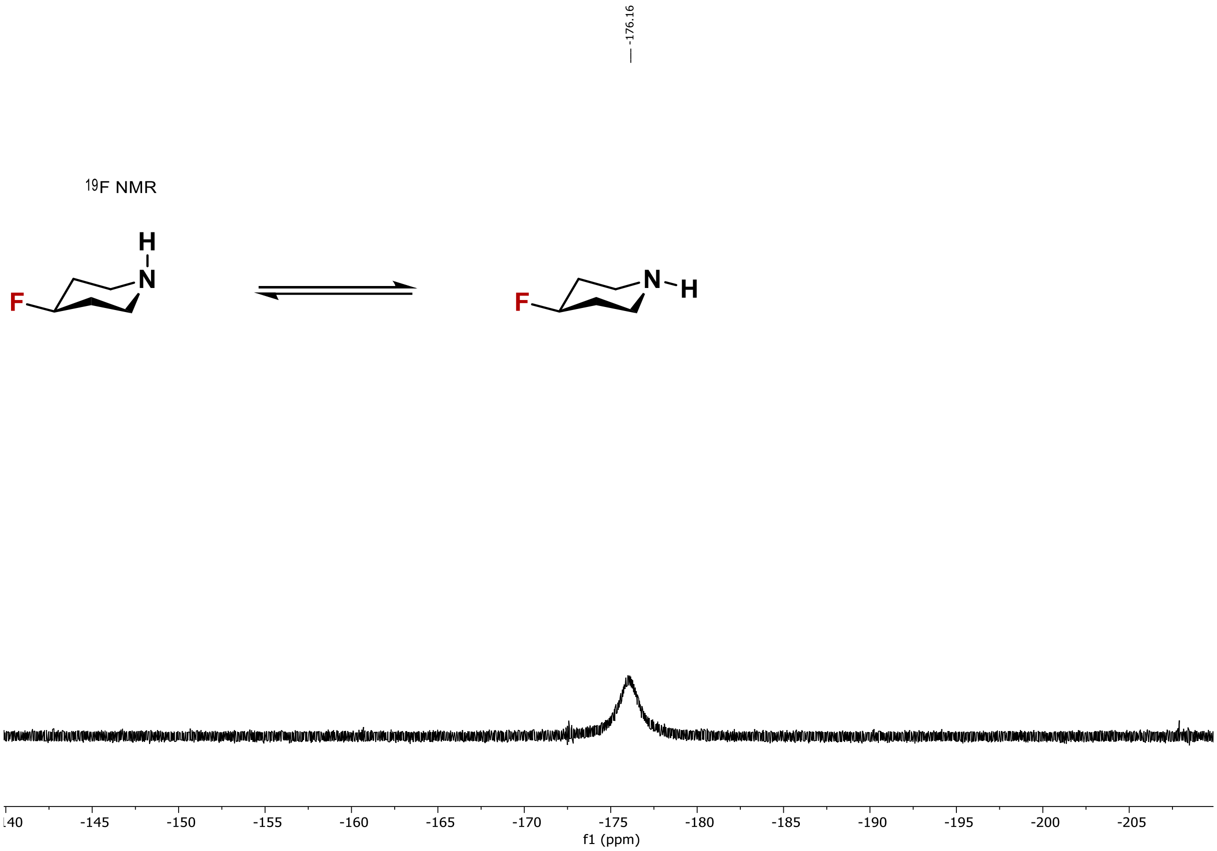
-176.16
19F NMR
F
N
H
f1 (ppm)

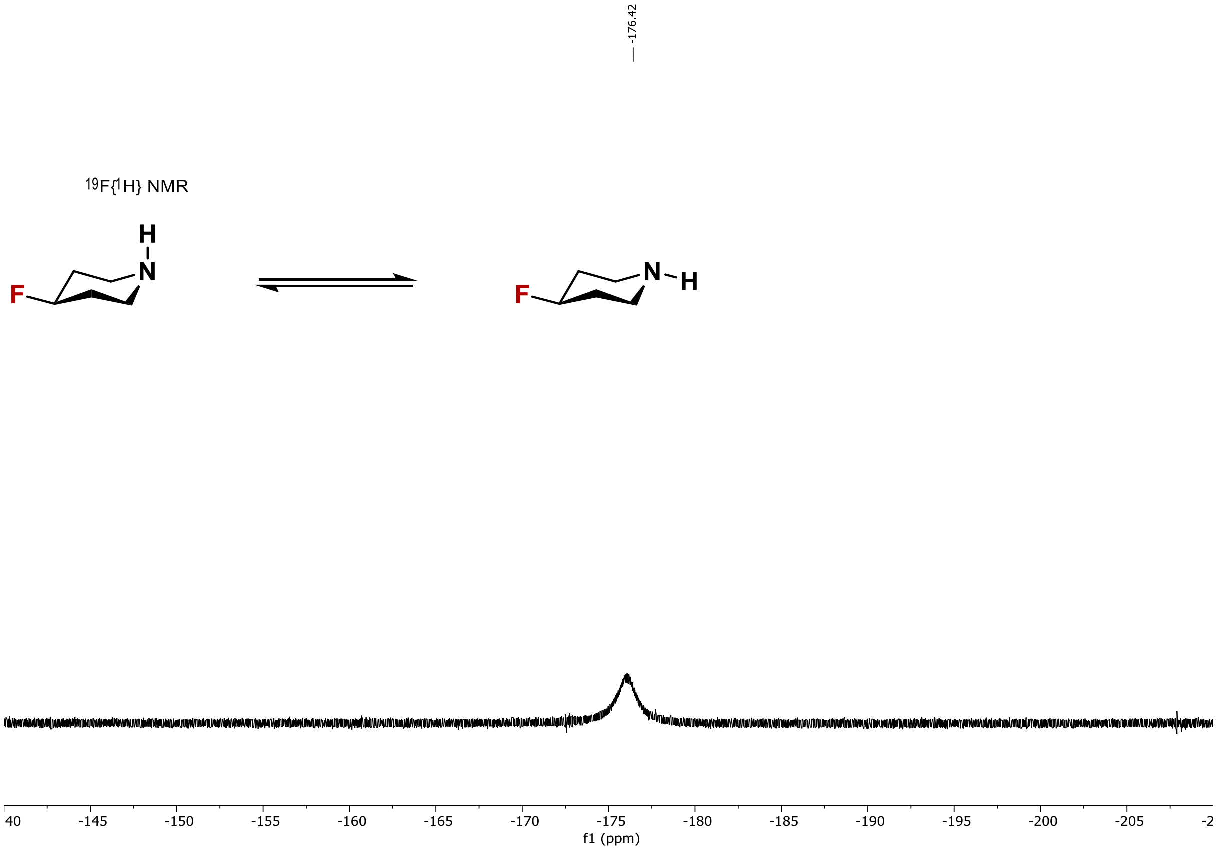
-176.42
19F{1H} NMR
F
N
H
f1 (ppm)

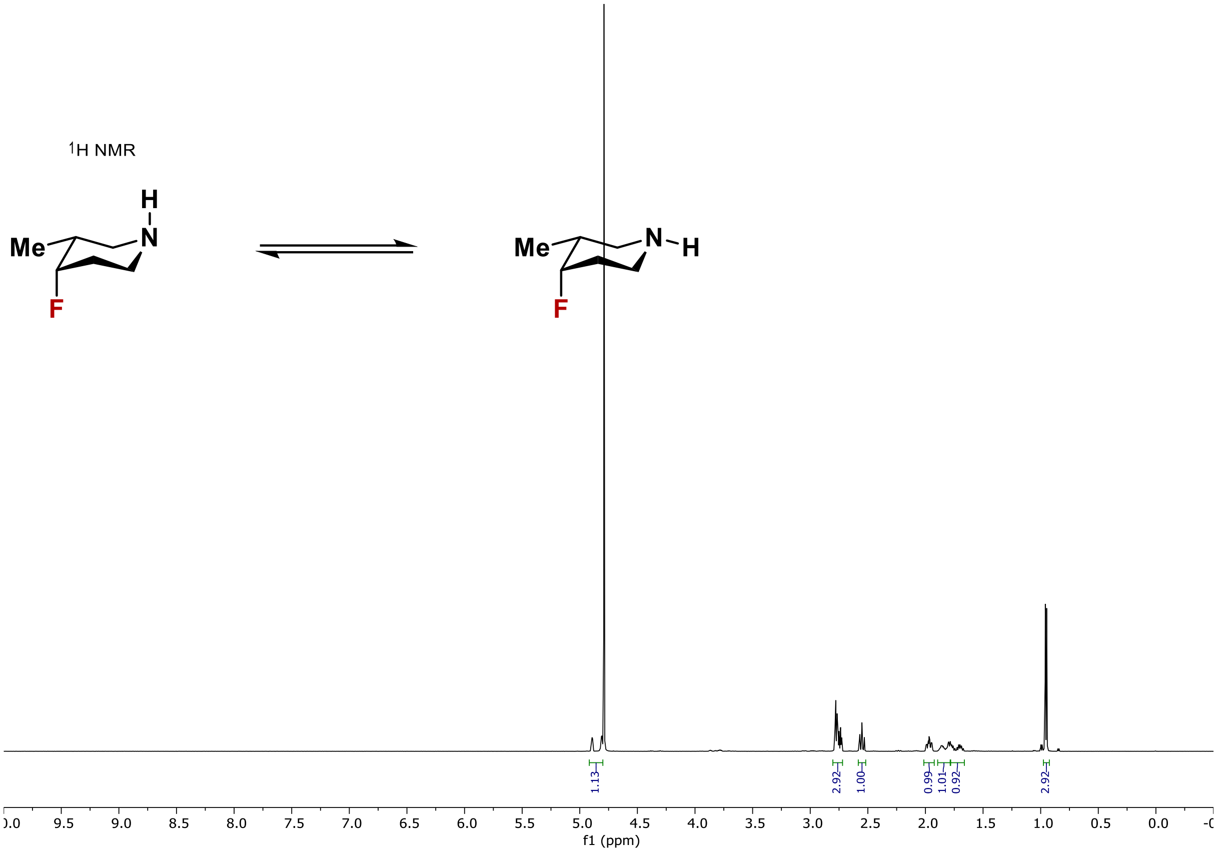

1H NMR
Me
H
N
F
Me
N
H
F
1.13
2.92
1.00
0.99
1.01
0.92
2.92
10.0 9.5 9.0 8.5 8.0 7.5 7.0 6.5 6.0 5.5 5.0 4.5 4.0 3.5 3.0 2.5 2.0 1.5 1.0 0.5 0.0
f1 (ppm)

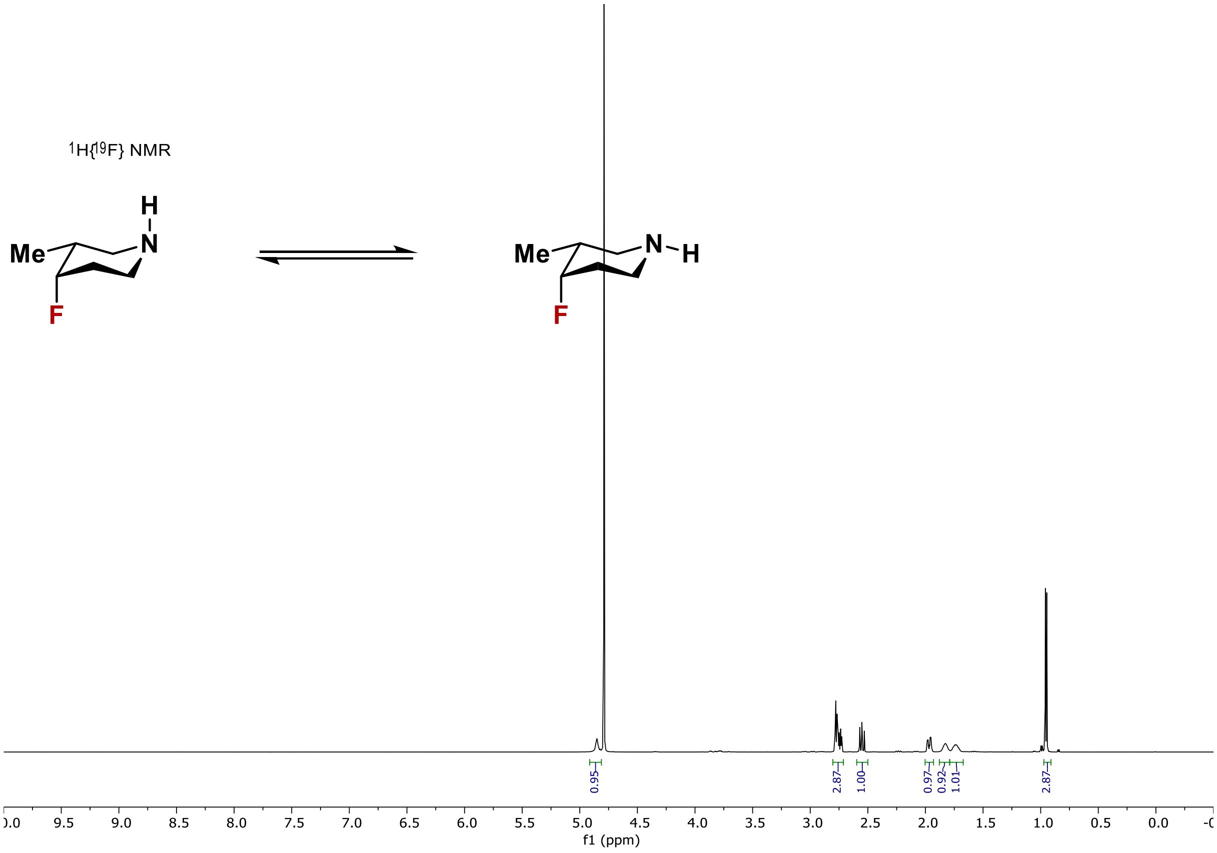

1H{19F} NMR
Me
H
N
F
Me
N
H
F
0.95
2.87
1.00
0.97
0.92
1.01
2.87
10.0 9.5 9.0 8.5 8.0 7.5 7.0 6.5 6.0 5.5 5.0 4.5 4.0 3.5 3.0 2.5 2.0 1.5 1.0 0.5 0.0
f1 (ppm)

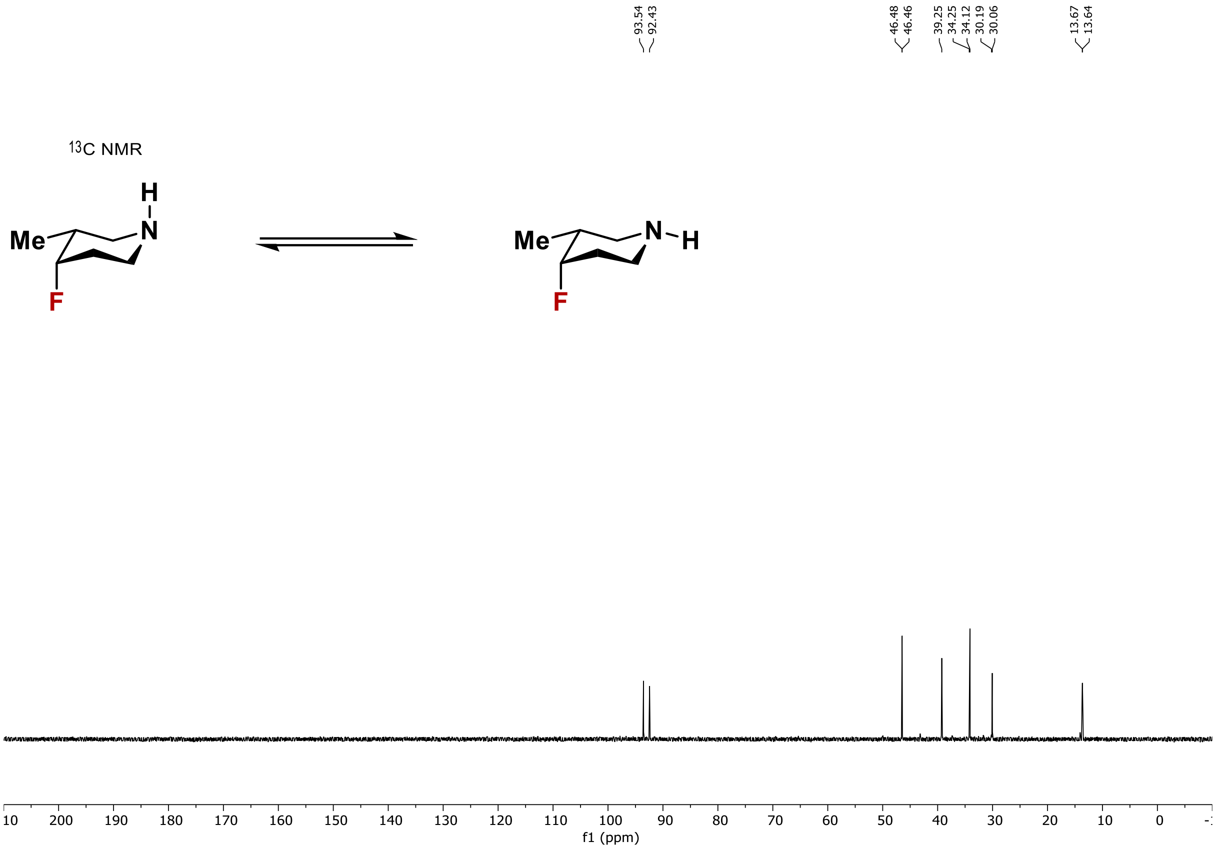

93.54
92.43
46.48
46.46
39.25
34.25
34.12
30.19
30.06
13.67
13.64
13C NMR
Me
F
N
H
f1 (ppm)

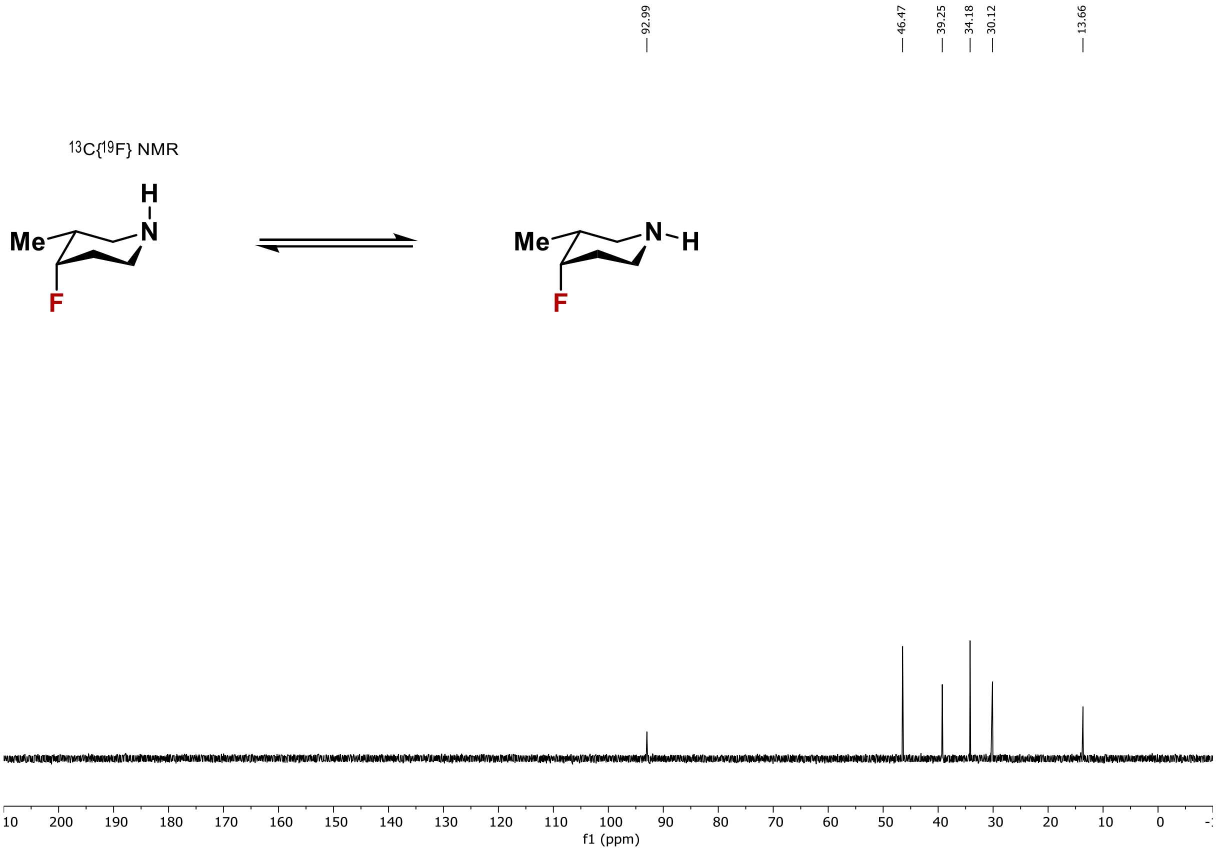

92.99
46.47
39.25
34.18
30.12
13.66
13C{19F} NMR
Me
F
N
H
f1 (ppm)

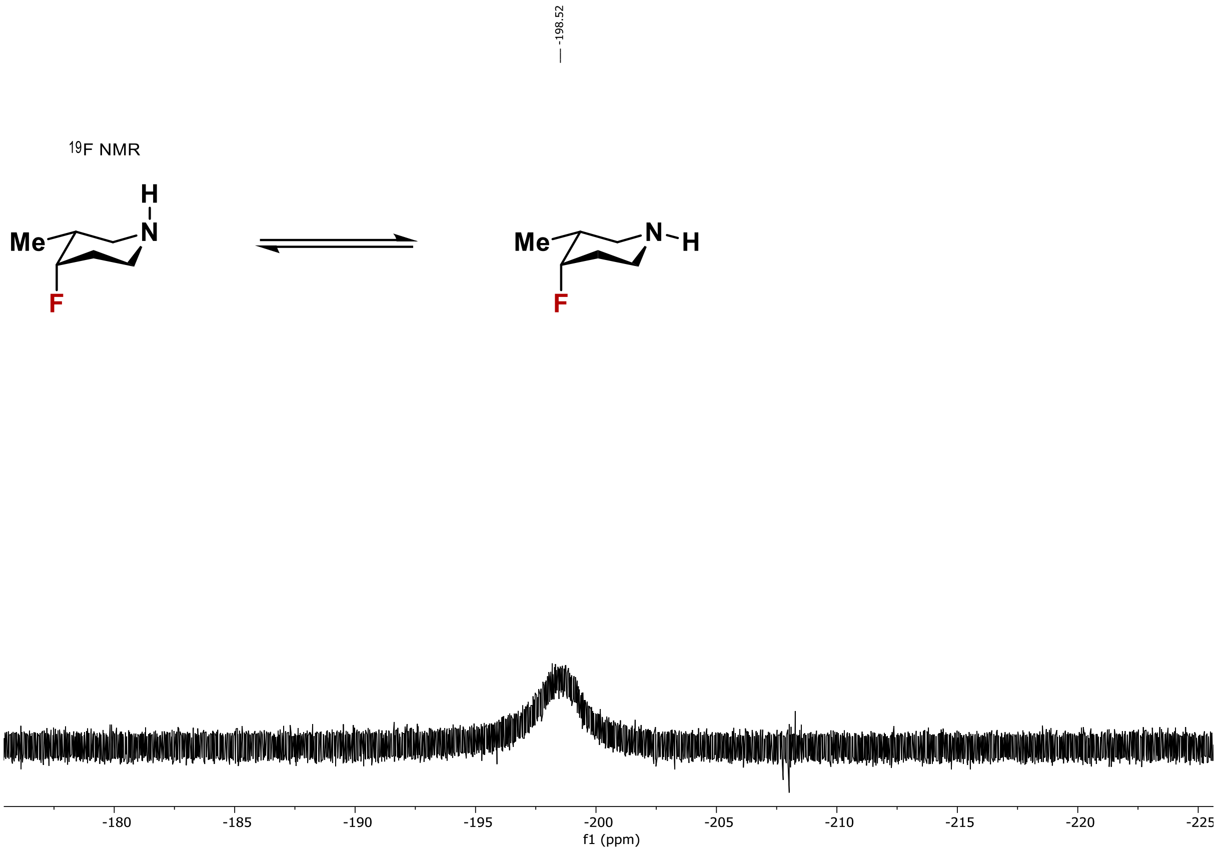

-198.52
19F NMR
H
N
Me
F
N
H
-180
-185
-190
-195
-200
-205
-210
-215
-220
-225
f1 (ppm)

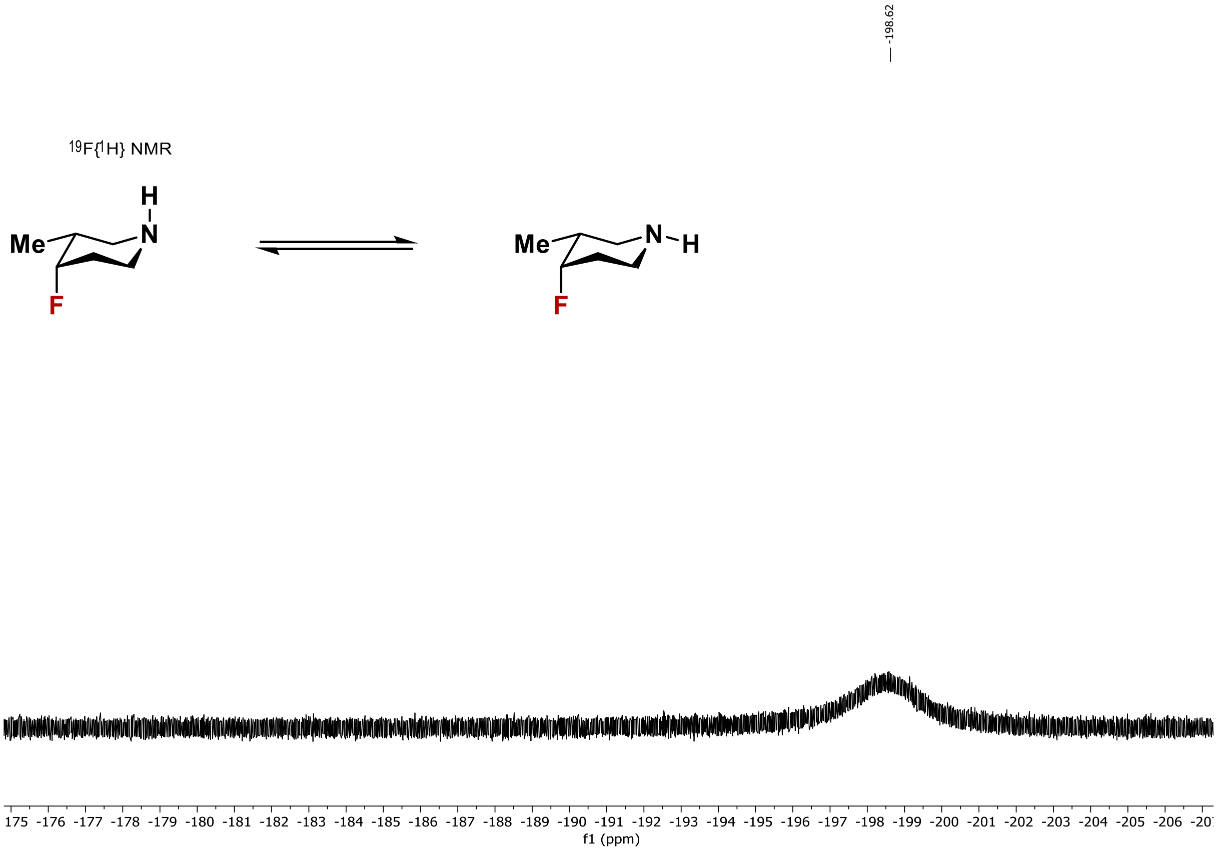

-198.62
19F{1H} NMR
H
N
Me
F
N
H
175 -176 -177 -178 -179 -180 -181 -182 -183 -184 -185 -186 -187 -188 -189 -190 -191 -192 -193 -194 -195 -196 -197 -198 -199 -200 -201 -202 -203 -204 -205 -206 -20
f1 (ppm)

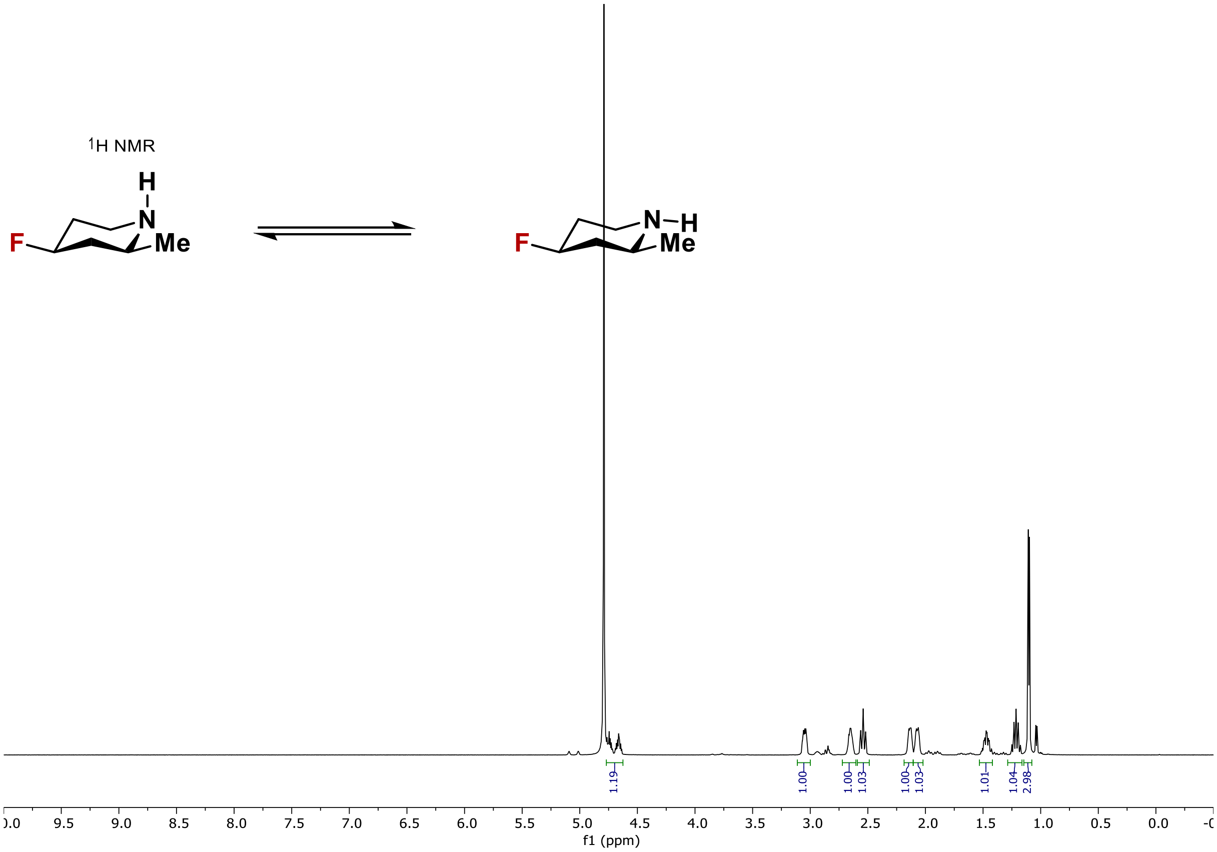

1H NMR
H
N
F
Me
N–H
F
Me
1.19
1.00
1.00
1.03
1.00
1.03
1.01
1.04
2.98
9.5 9.0 8.5 8.0 7.5 7.0 6.5 6.0 5.5 5.0 4.5 4.0 3.5 3.0 2.5 2.0 1.5 1.0 0.5 0.0
f1 (ppm)

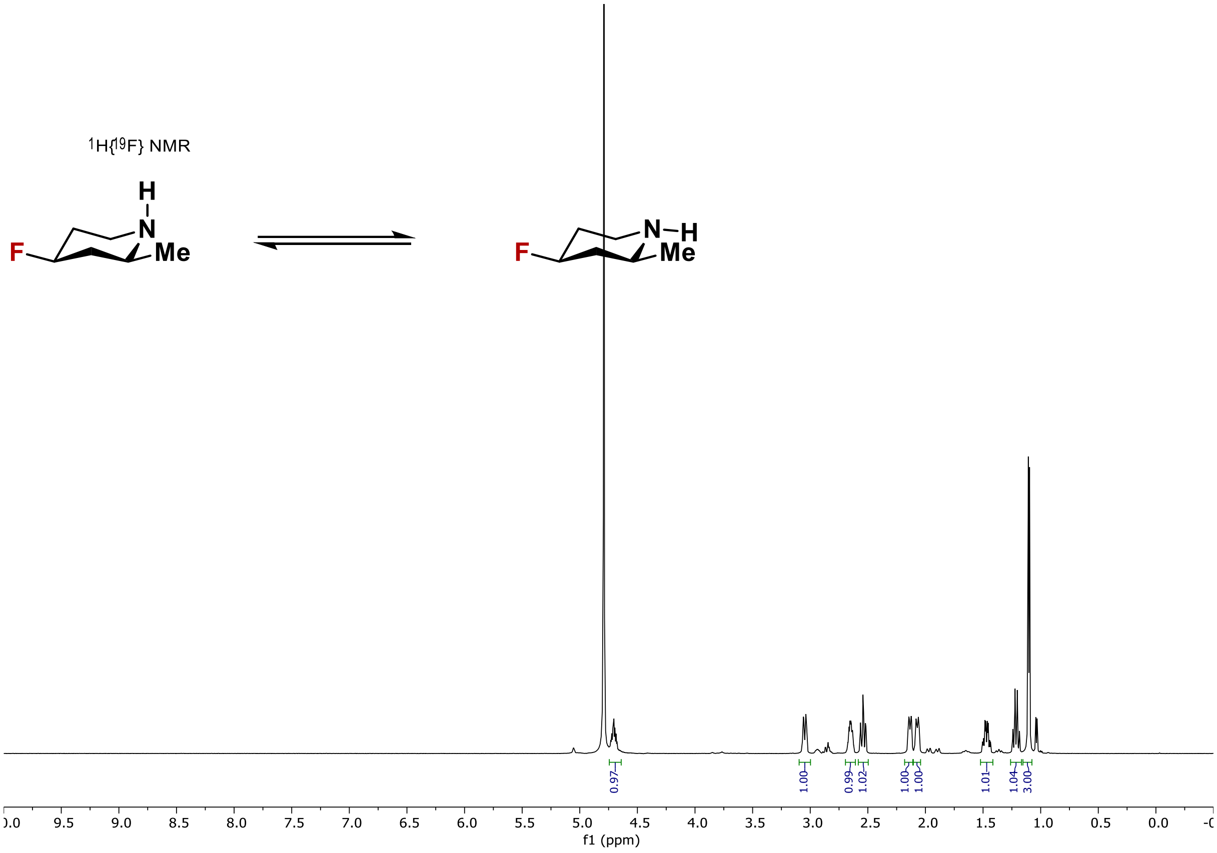

1H{19F} NMR
H
N
F
Me
N–H
F
Me
0.97
1.00
0.99
1.02
1.00
1.00
1.01
1.04
3.00
9.5 9.0 8.5 8.0 7.5 7.0 6.5 6.0 5.5 5.0 4.5 4.0 3.5 3.0 2.5 2.0 1.5 1.0 0.5 0.0
f1 (ppm)

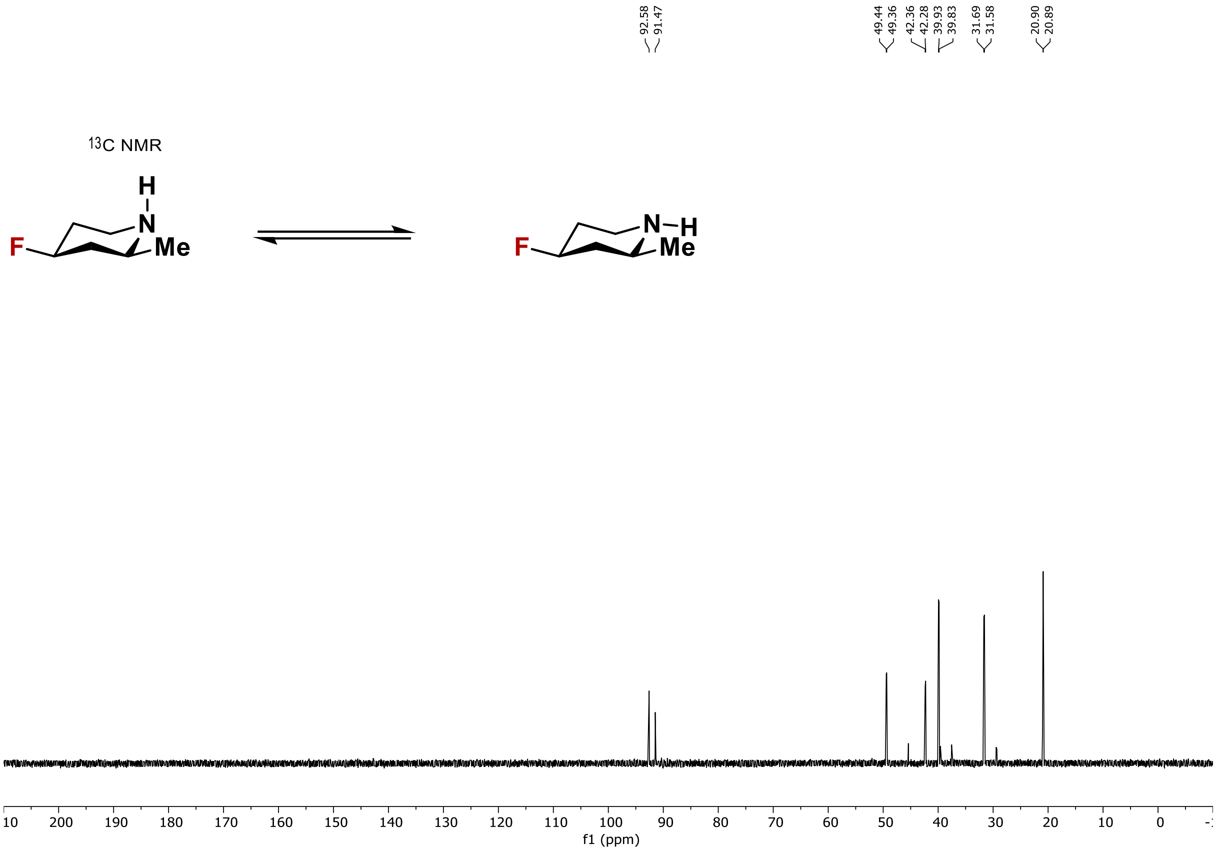
92.58
91.47
49.44
49.36
42.36
42.28
39.93
39.83
31.69
31.58
20.90
20.89
13C NMR
H
N
Me
F
N–H
Me
F
f1 (ppm)

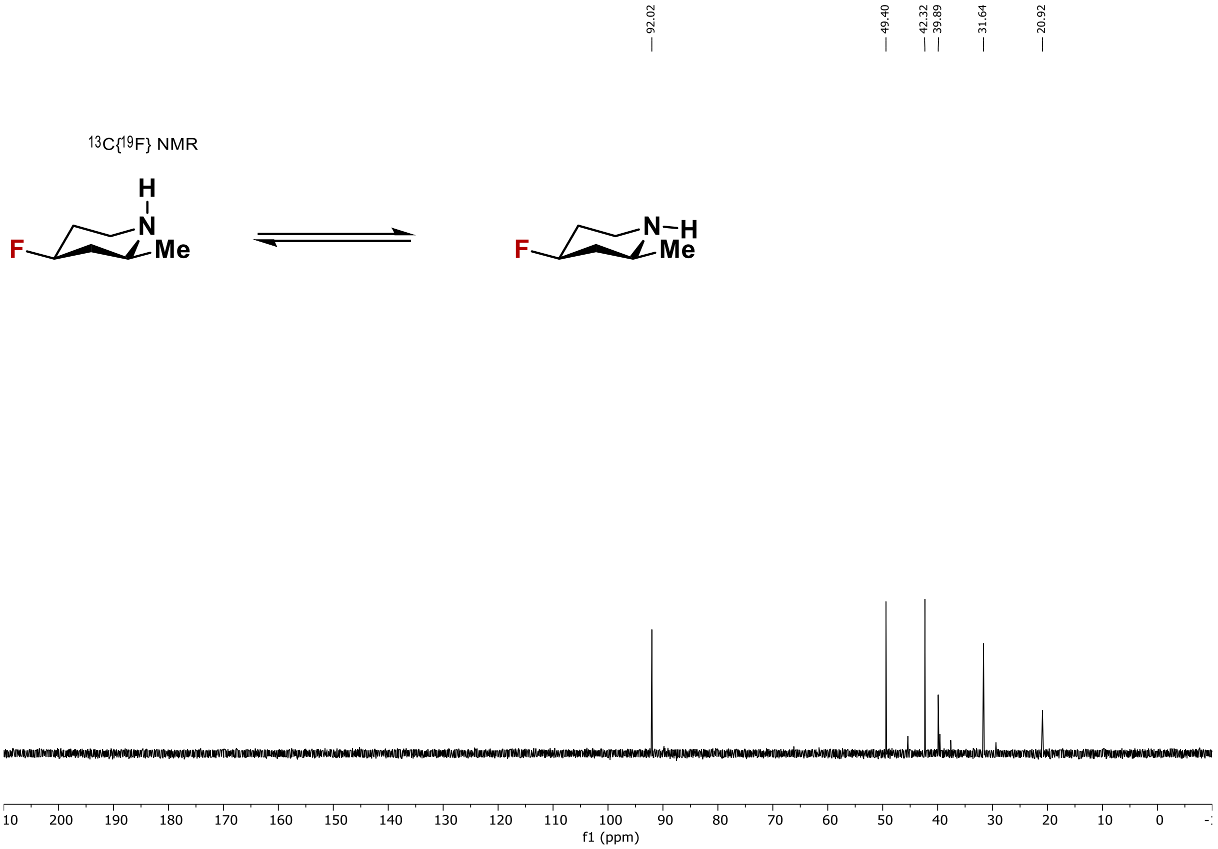
92.02
49.40
42.32
39.89
31.64
20.92
13C{19F} NMR
H
N
Me
F
N–H
Me
F
f1 (ppm)

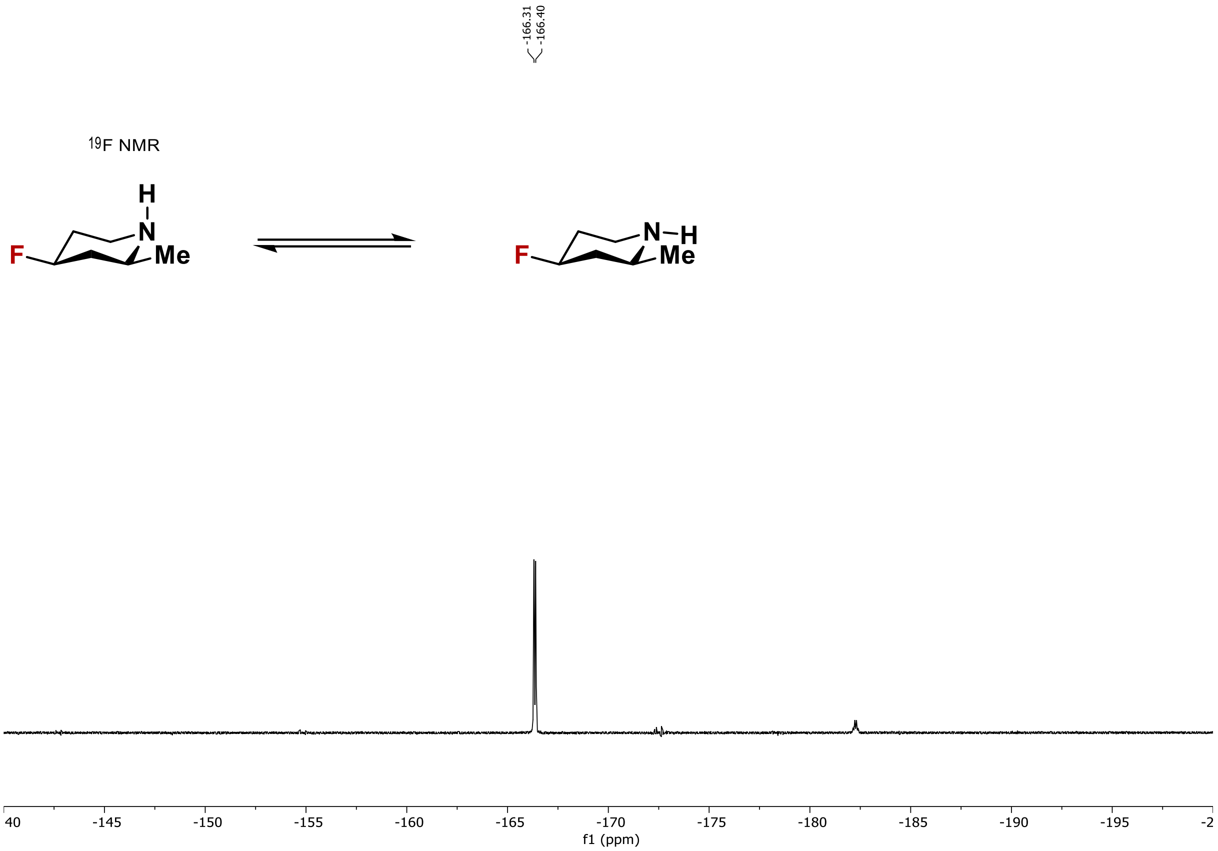
-166.31
-166.40
19F NMR
H
N
F
Me
N–H
F
Me
40
-145
-150
-155
-160
-165
-170
-175
-180
-185
-190
-195
-2
f1 (ppm)

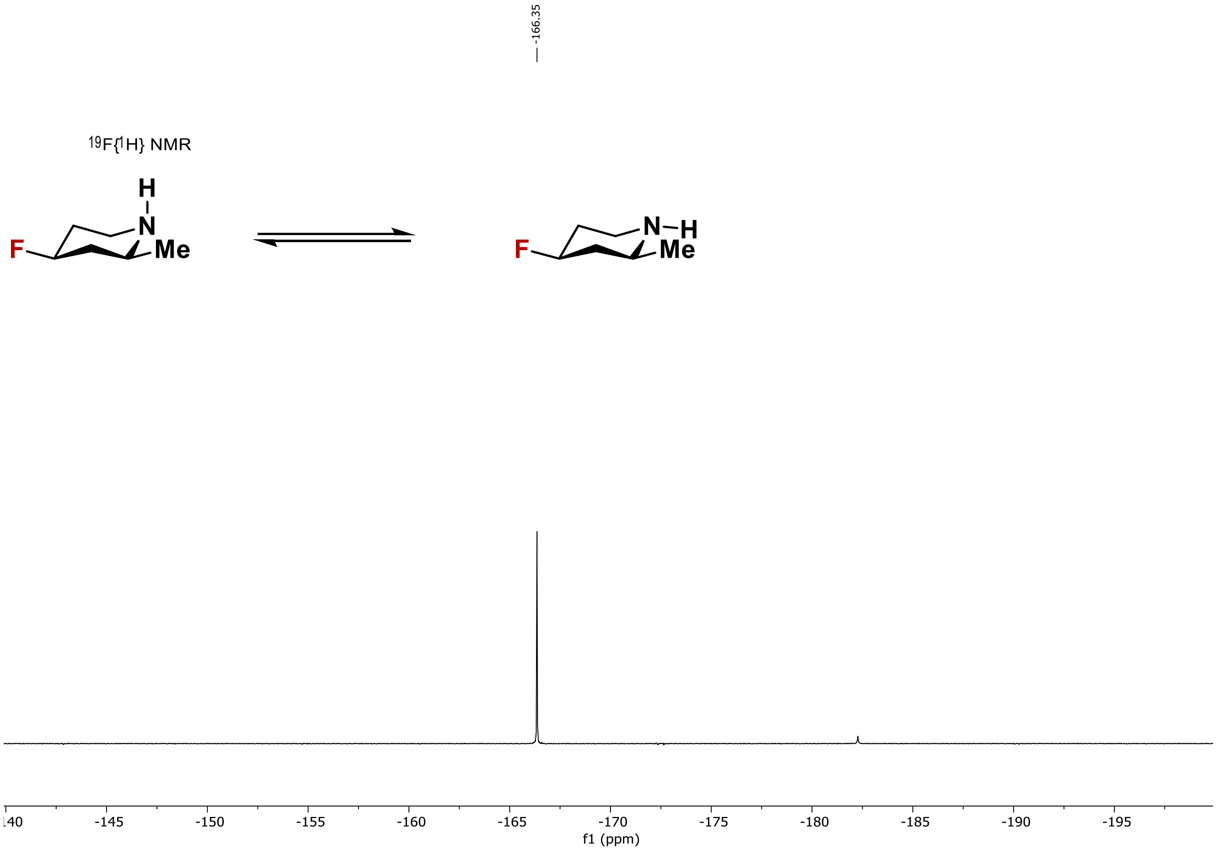
-166.35
19F{1H} NMR
H
N
F
Me
N–H
F
Me
.40
-145
-150
-155
-160
-165
-170
-175
-180
-185
-190
-195
f1 (ppm)

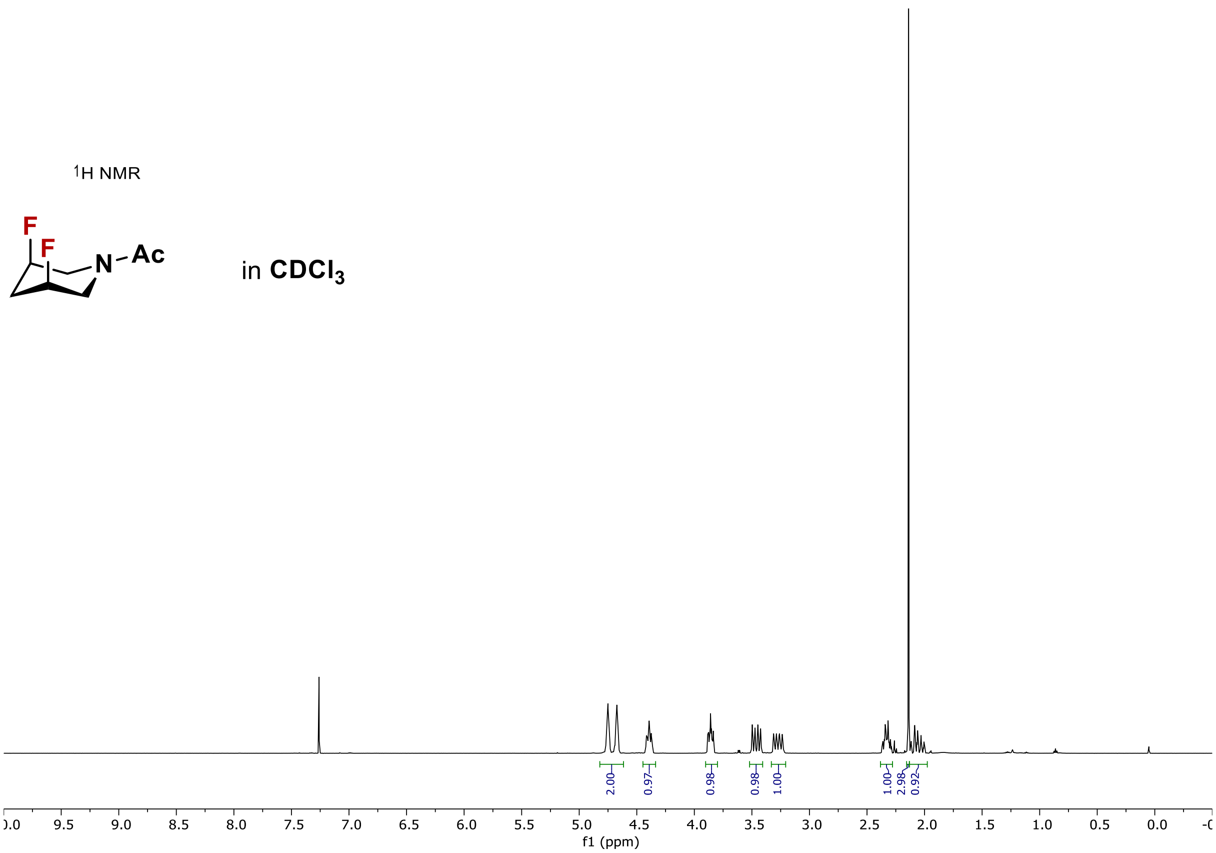

1H NMR
F
F
N
Ac
in CDCl3
2.00
0.97
0.98
0.98
1.00
1.00
2.98
0.92
f1 (ppm)

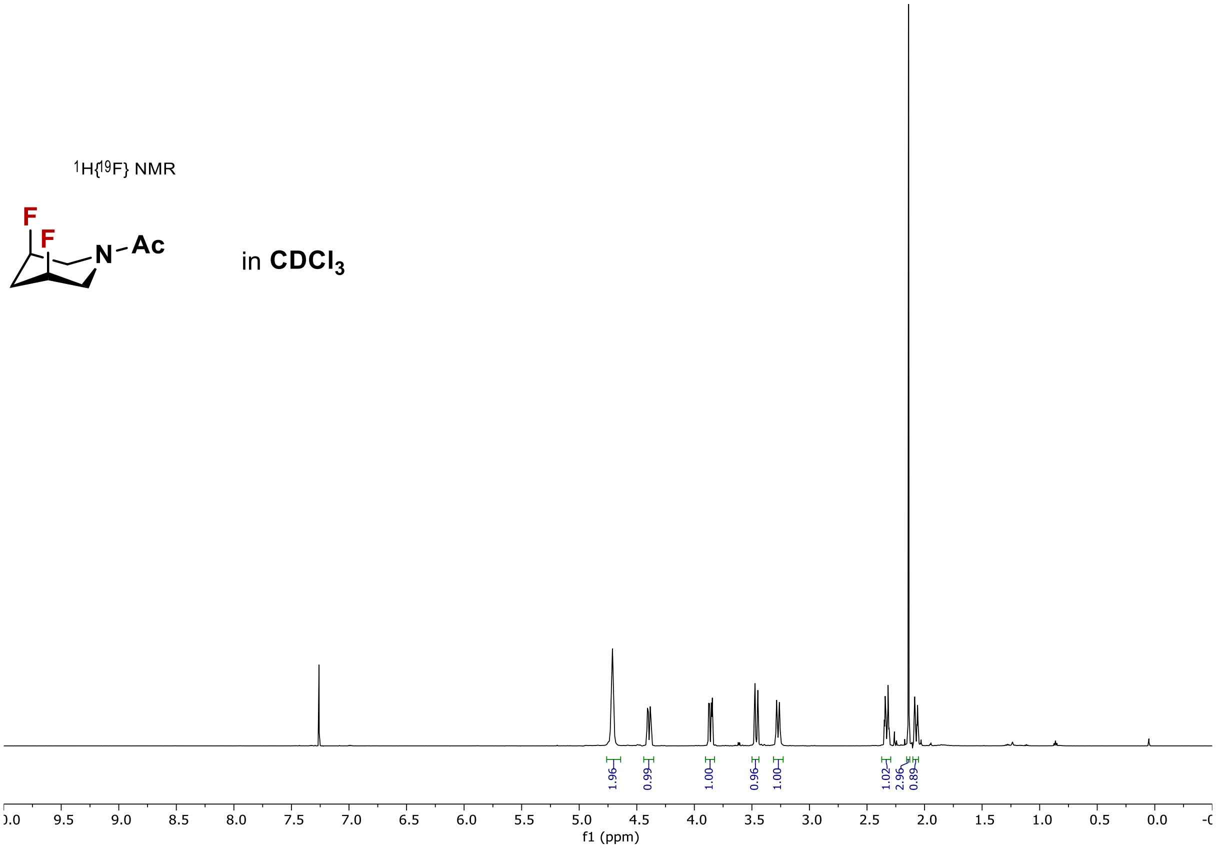

1H{19F} NMR
F
F
N
Ac
in CDCl3
1.96
0.99
1.00
0.96
1.00
1.02
2.96
0.89
f1 (ppm)

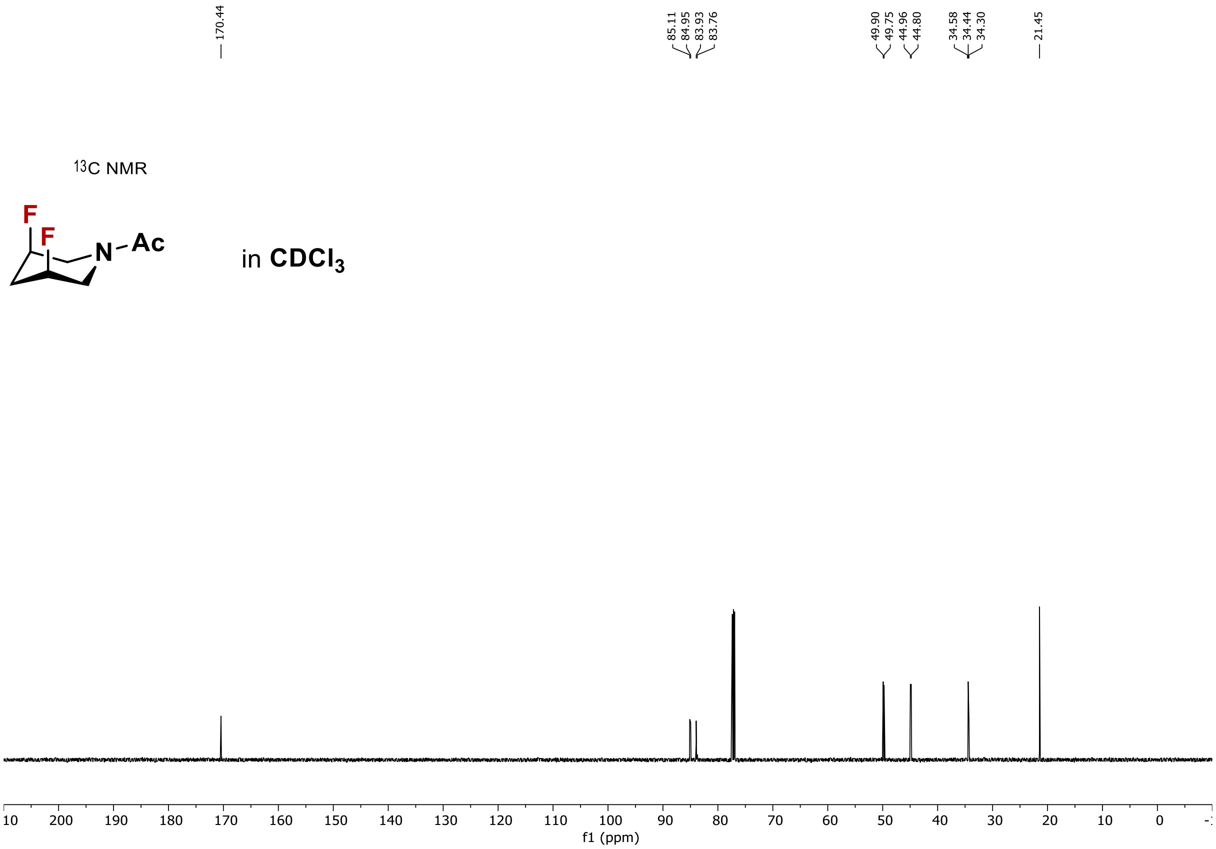
170.44
85.11
84.95
83.93
83.76
49.90
49.75
44.96
44.80
34.58
34.44
34.30
21.45
13C NMR
F
F
N
Ac
in CDCl3
210 200 190 180 170 160 150 140 130 120 110 100 90 80 70 60 50 40 30 20 10 0
f1 (ppm)

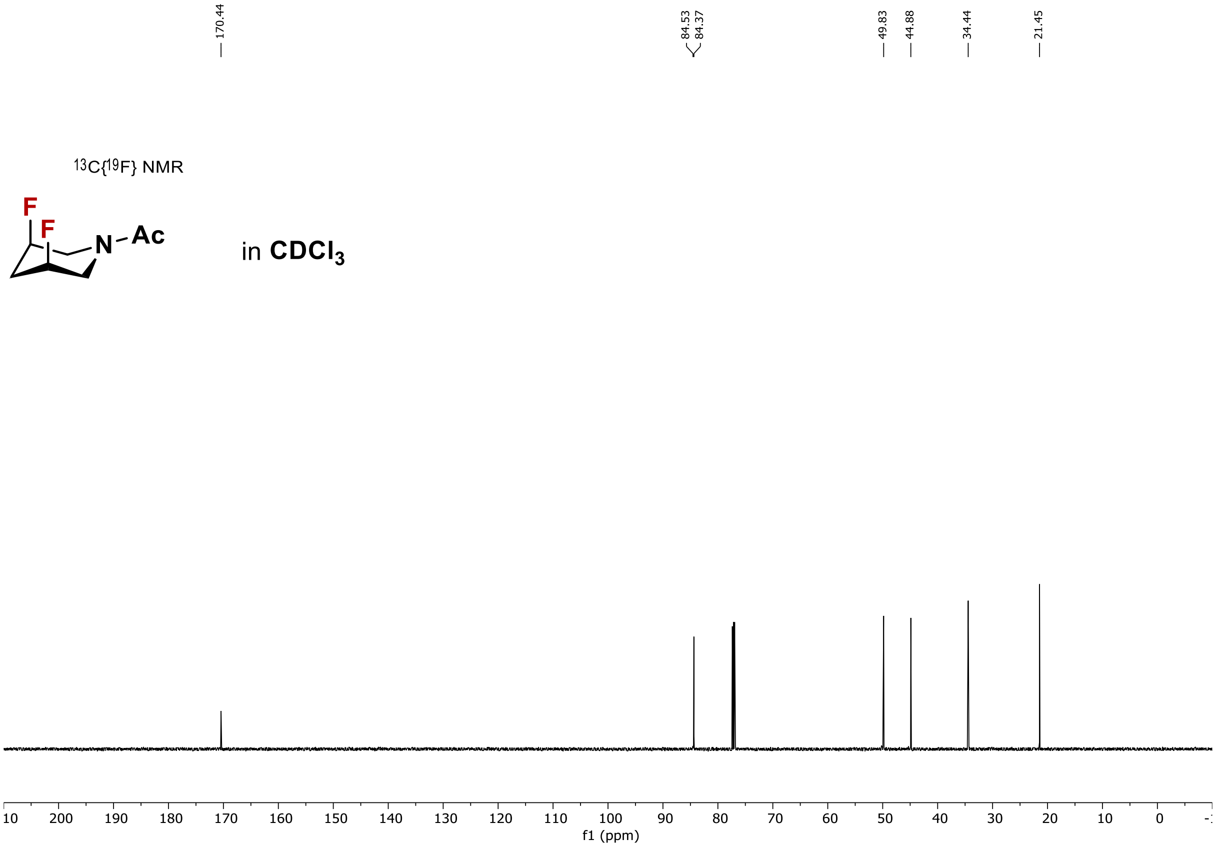
170.44
84.53
84.37
49.83
44.88
34.44
21.45
13C{19F} NMR
F
F
N
Ac
in CDCl3
210 200 190 180 170 160 150 140 130 120 110 100 90 80 70 60 50 40 30 20 10 0
f1 (ppm)

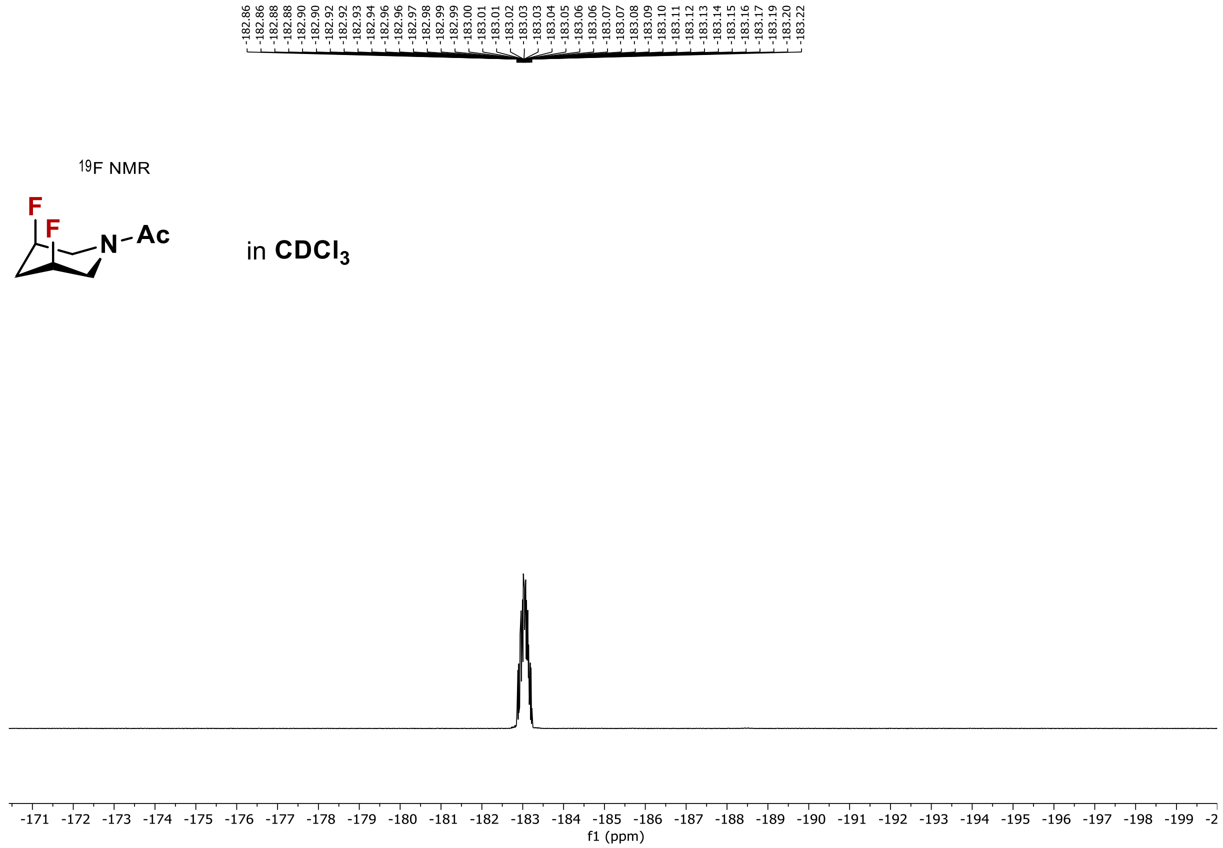
-182.86 -182.86 -182.88 -182.88 -182.90 -182.92 -182.92 -182.93 -182.94 -182.96 -182.96 -182.97 -182.98 -182.99 -182.99 -183.00 -183.01 -183.01 -183.02 -183.03 -183.03 -183.04 -183.05 -183.06 -183.06 -183.07 -183.07 -183.08 -183.09 -183.10 -183.11 -183.12 -183.13 -183.14 -183.15 -183.16 -183.17 -183.19 -183.20 -183.22
19F NMR
F
F
N
Ac
in CDCl3
-171 -172 -173 -174 -175 -176 -177 -178 -179 -180 -181 -182 -183 -184 -185 -186 -187 -188 -189 -190 -191 -192 -193 -194 -195 -196 -197 -198 -199 -2
f1 (ppm)

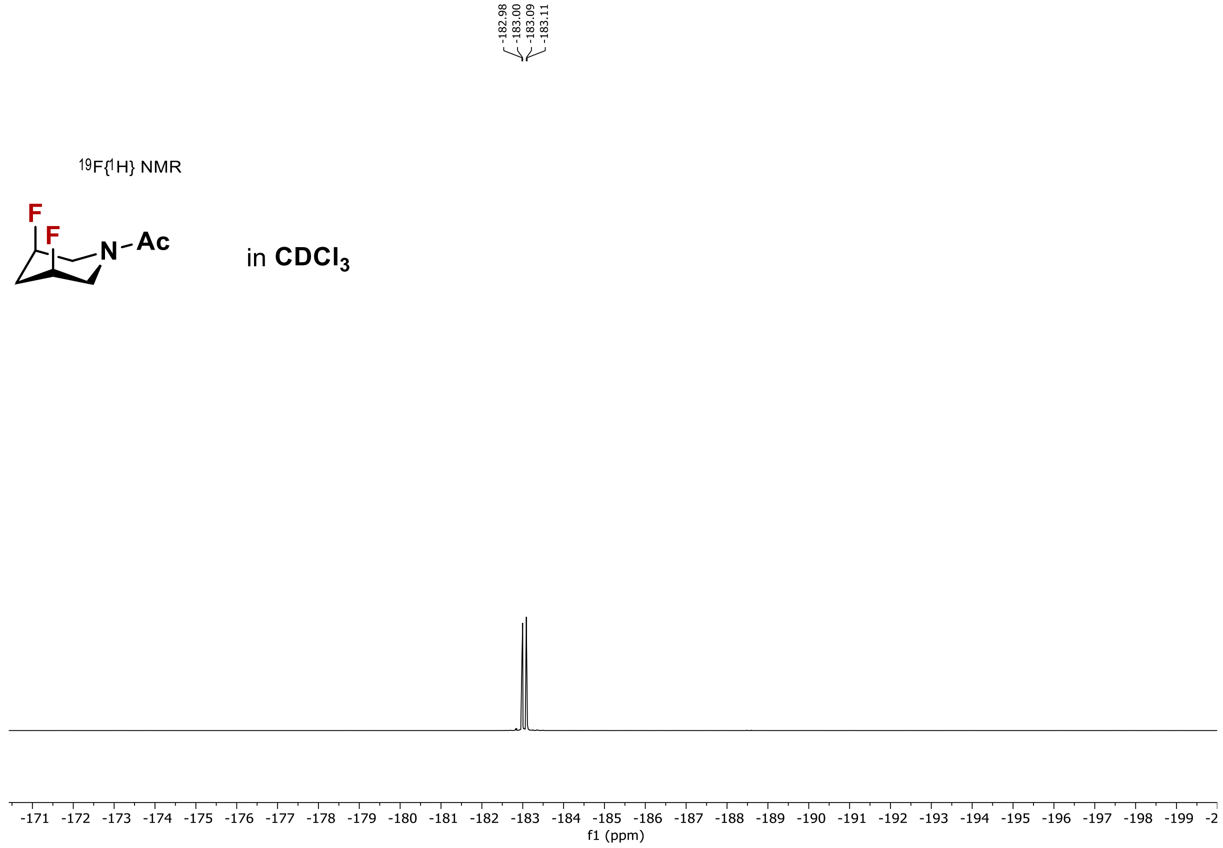
-182.98 -183.00 -183.09 -183.11
19F{1H} NMR
F
F
N
Ac
in CDCl3
-171 -172 -173 -174 -175 -176 -177 -178 -179 -180 -181 -182 -183 -184 -185 -186 -187 -188 -189 -190 -191 -192 -193 -194 -195 -196 -197 -198 -199 -2
f1 (ppm)

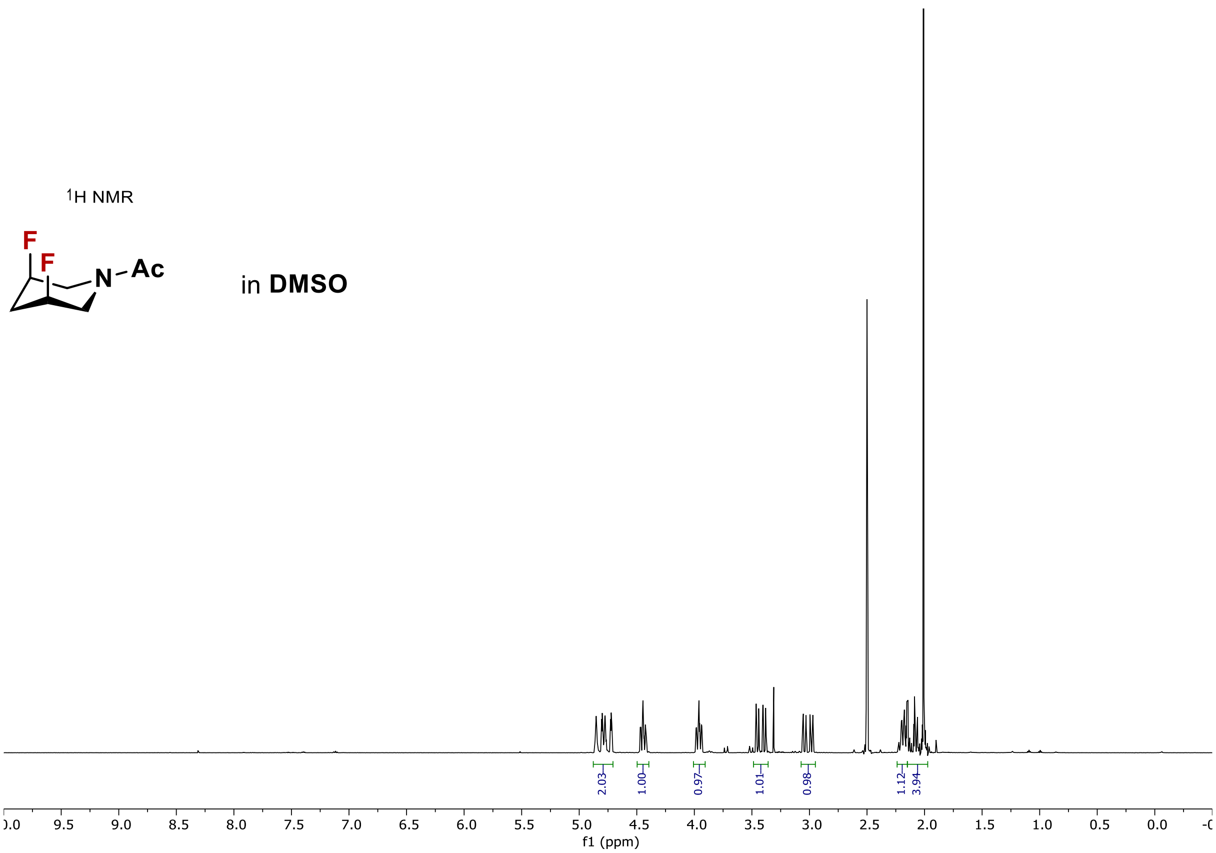

^{1}H NMR
F
F
N
Ac
in DMSO
2.03
1.00
0.97
1.01
0.98
1.12
3.94
10.0 9.5 9.0 8.5 8.0 7.5 7.0 6.5 6.0 5.5 5.0 4.5 4.0 3.5 3.0 2.5 2.0 1.5 1.0 0.5 0.0
f1 (ppm)

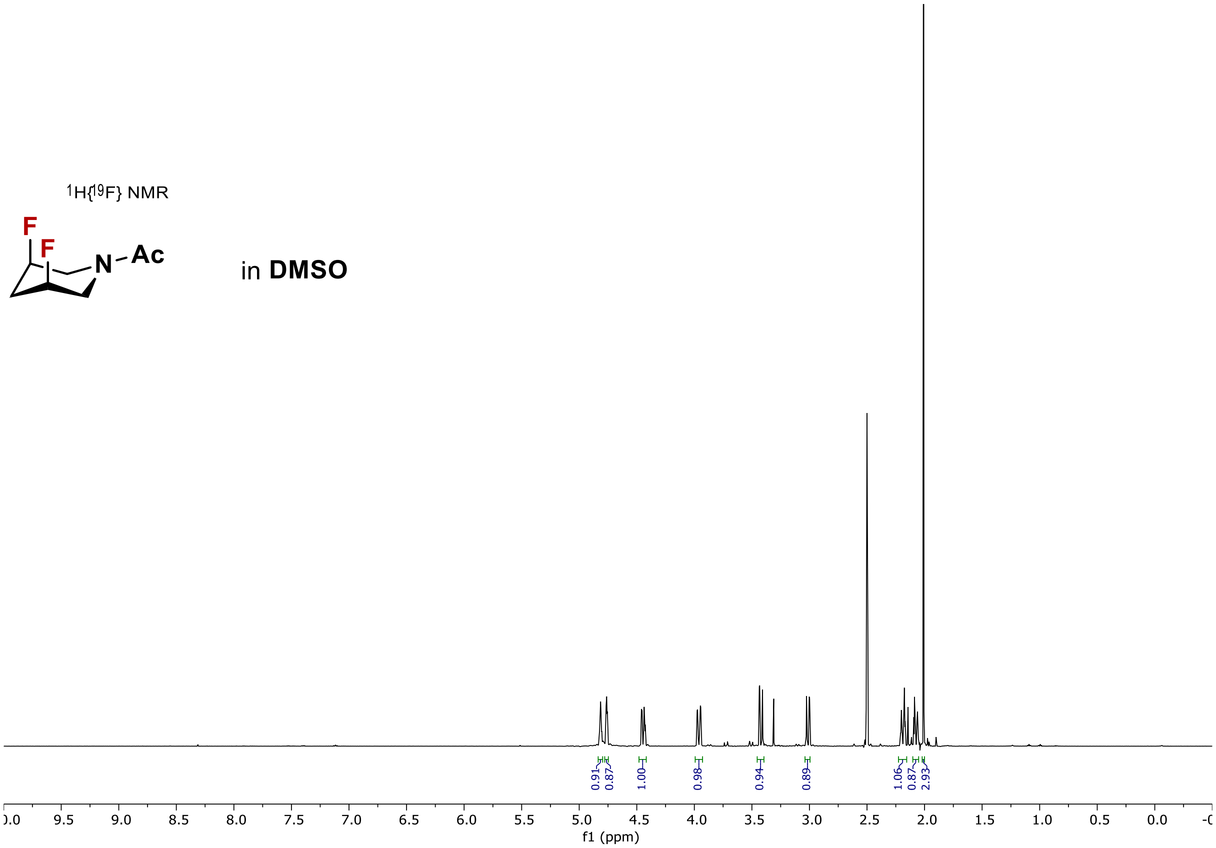

^{1}H{^{19}F} NMR
F
F
N
Ac
in DMSO
0.91
0.87
1.00
0.98
0.94
0.89
1.06
0.87
2.93
10.0 9.5 9.0 8.5 8.0 7.5 7.0 6.5 6.0 5.5 5.0 4.5 4.0 3.5 3.0 2.5 2.0 1.5 1.0 0.5 0.0
f1 (ppm)

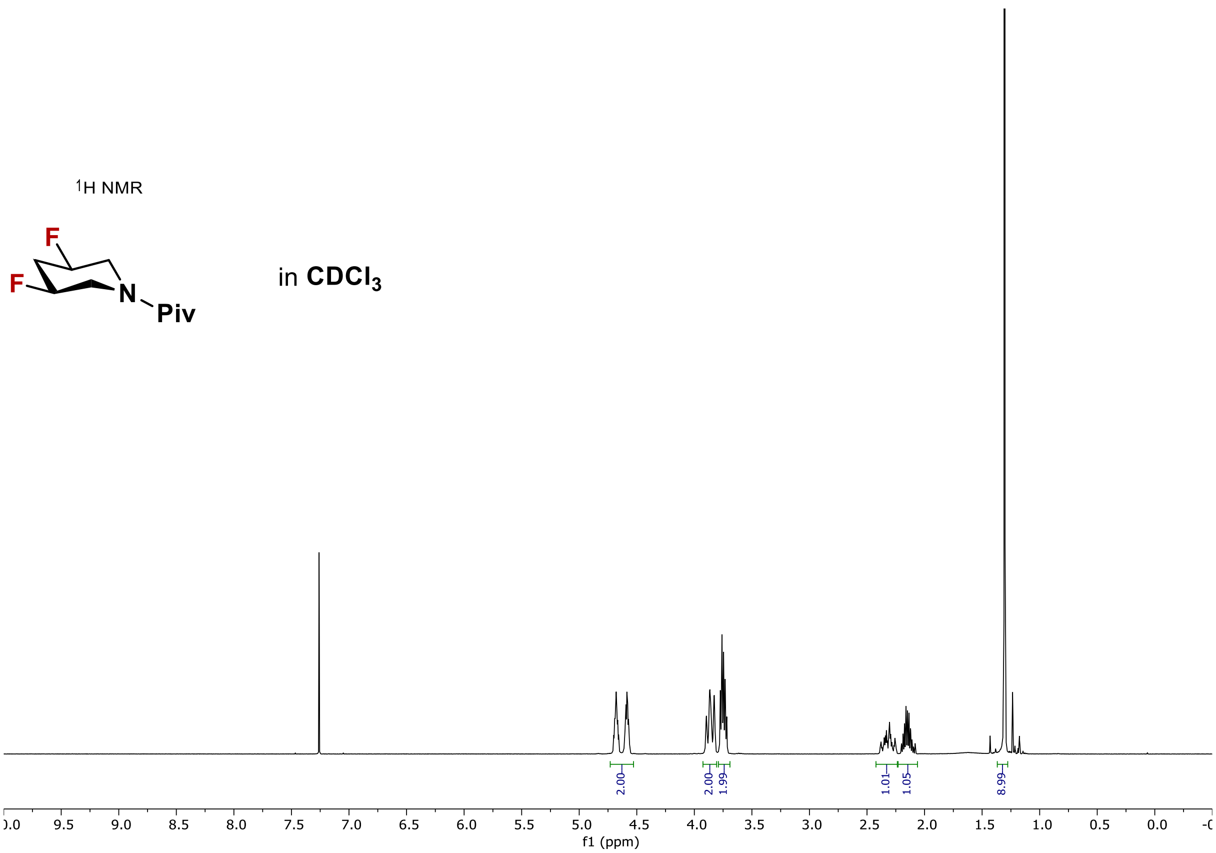

1H NMR
F
F
N
Piv
in CDCl3
2.00
2.00
1.99
1.01
1.05
8.99
f1 (ppm)

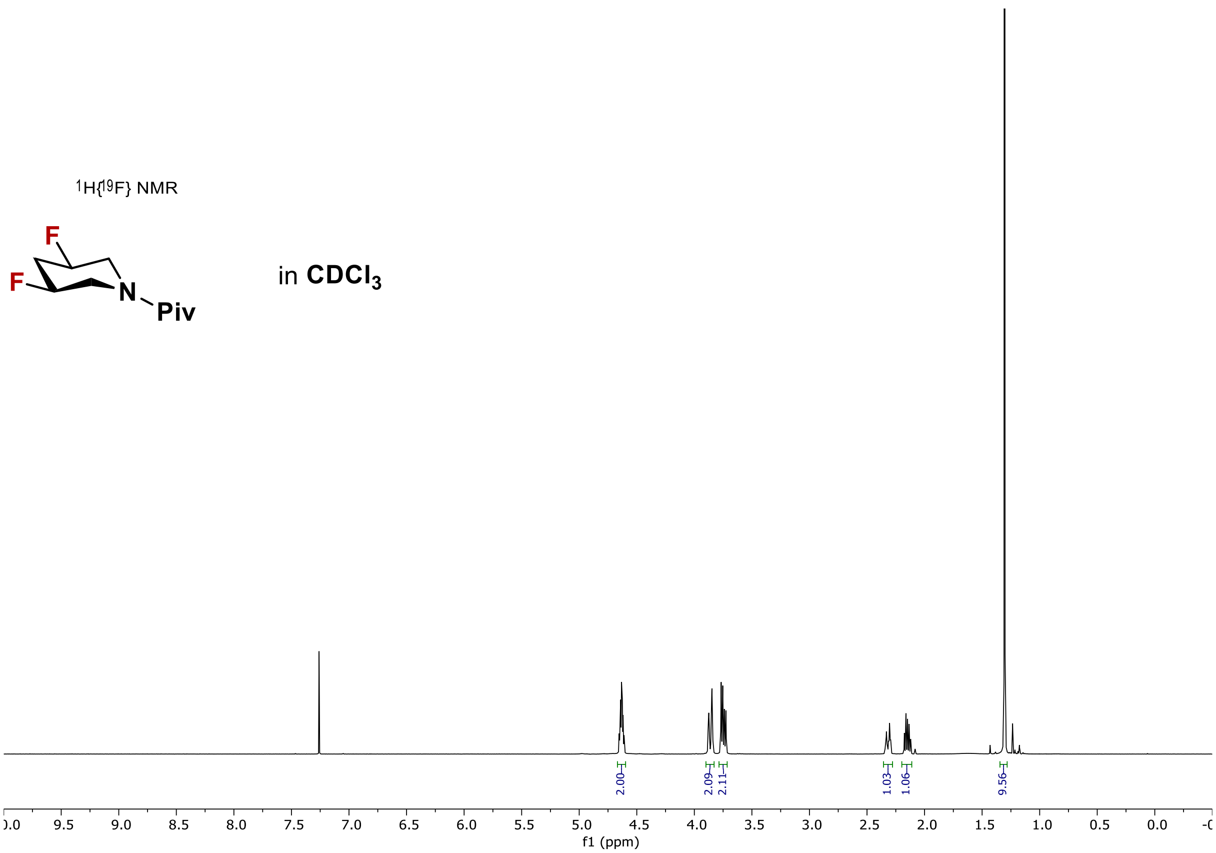

1H{19F} NMR
F
F
N
Piv
in CDCl3
2.00
2.09
2.11
1.03
1.06
9.56
f1 (ppm)

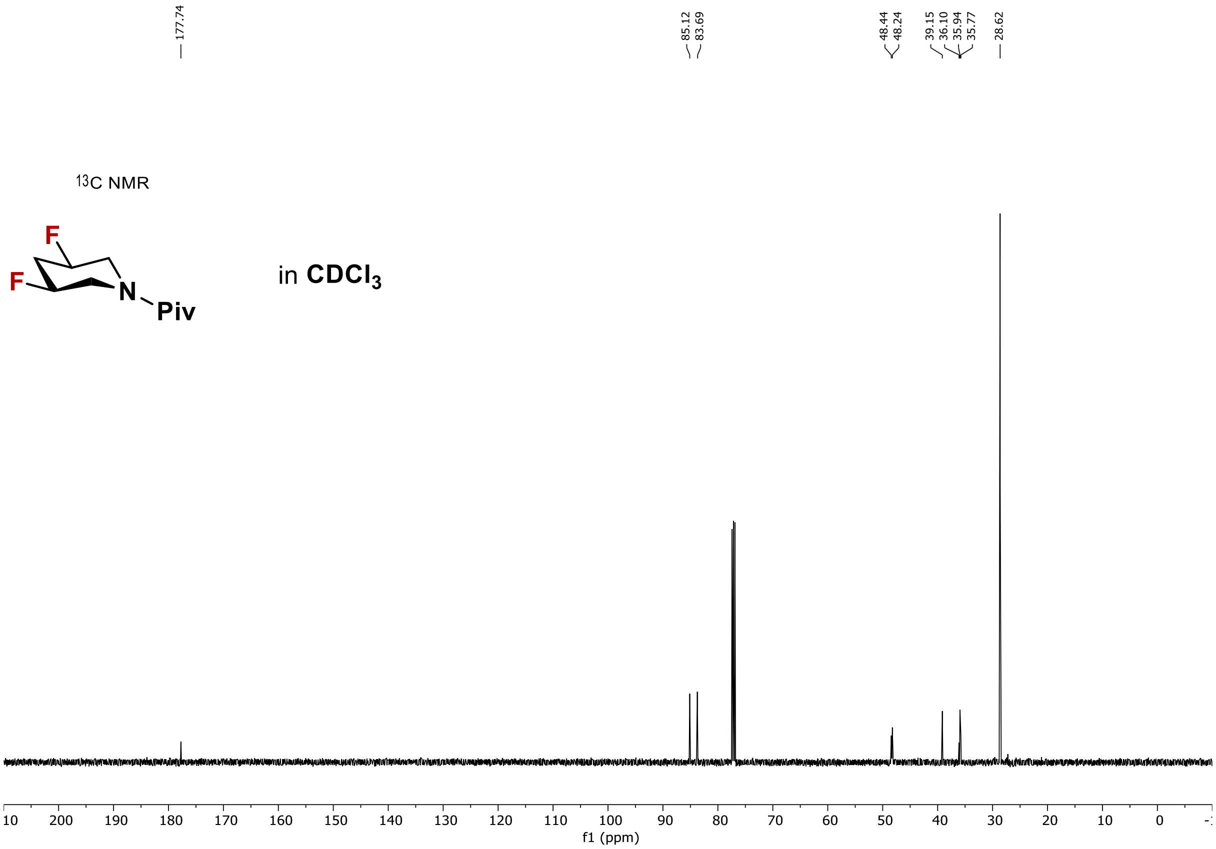

177.74
85.12
83.69
48.44
48.24
39.15
36.10
35.94
35.77
28.62
13C NMR
F
F
N
Piv
in CDCl3
f1 (ppm)

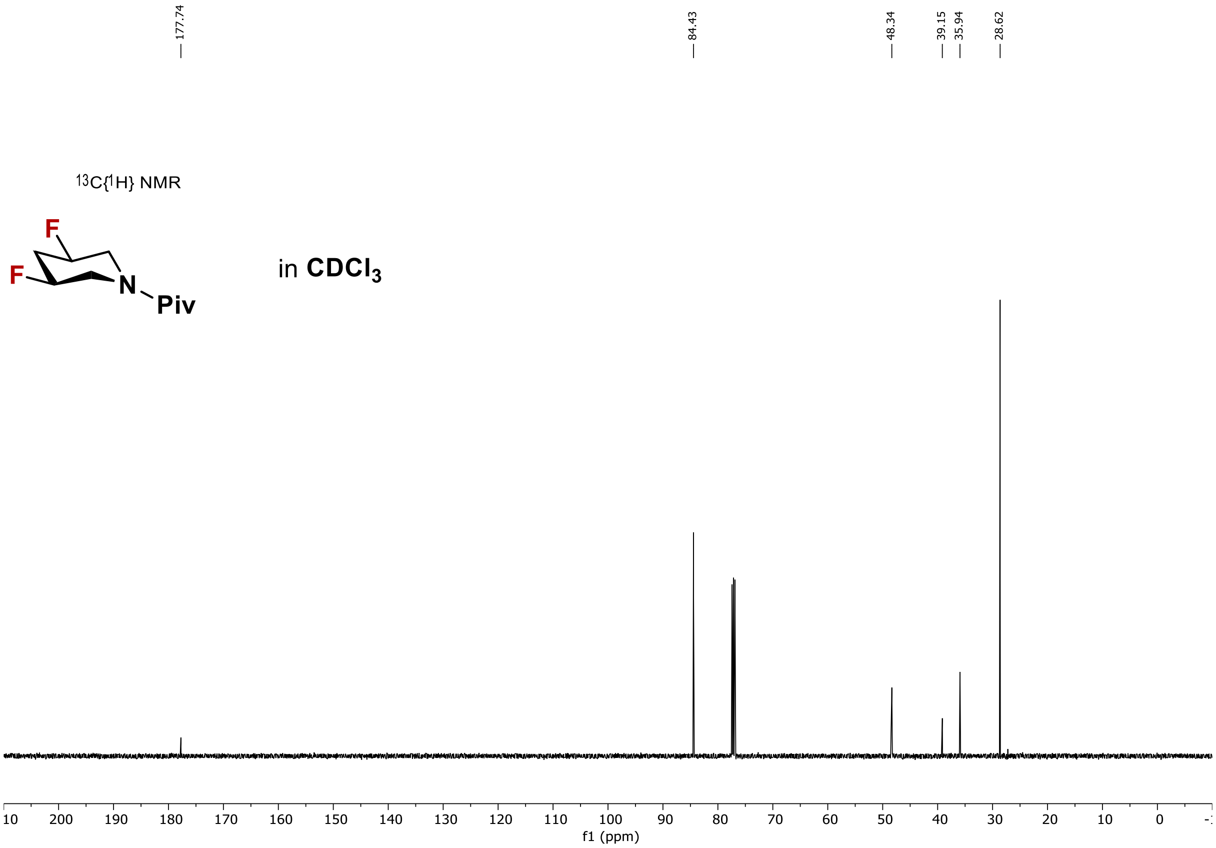

177.74
84.43
48.34
39.15
35.94
28.62
13C{1H} NMR
F
F
N
Piv
in CDCl3
f1 (ppm)

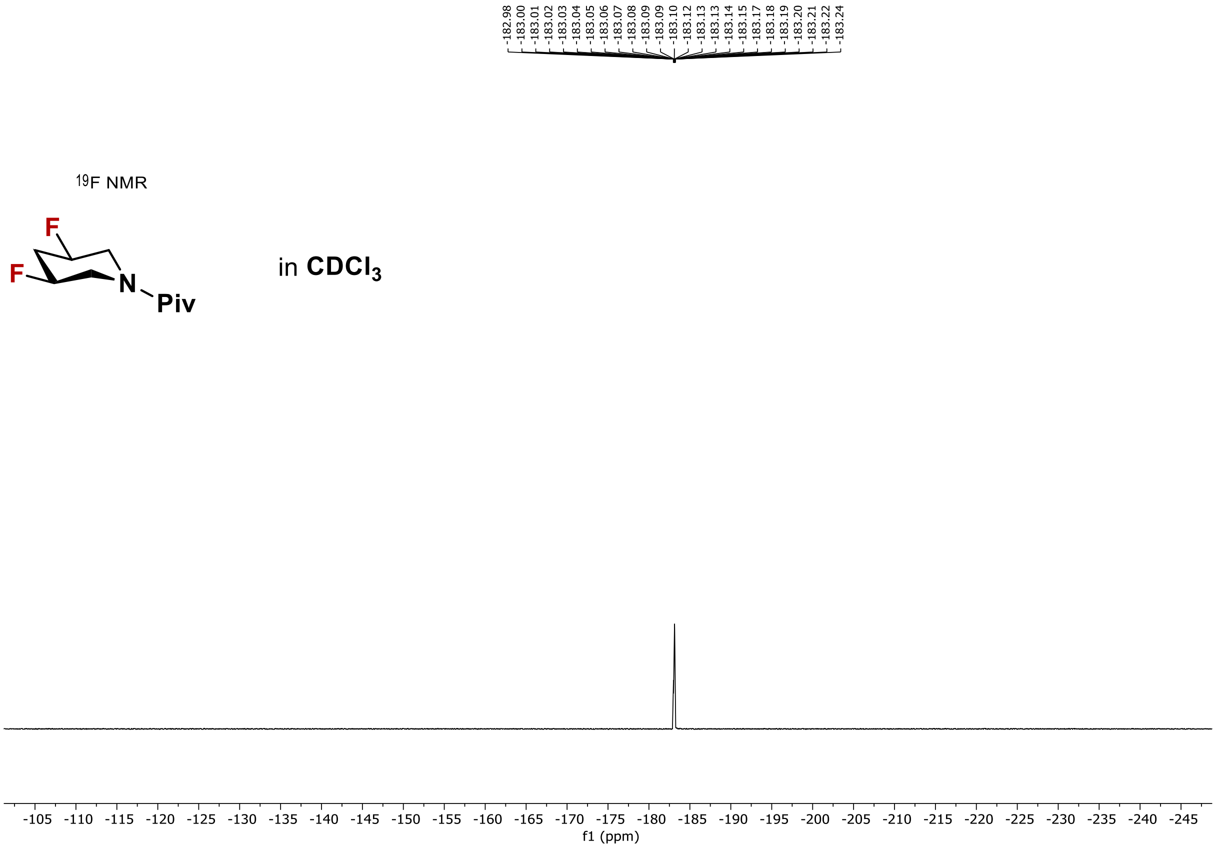

-182.98
-183.00
-183.01
-183.02
-183.03
-183.04
-183.05
-183.06
-183.07
-183.08
-183.09
-183.09
-183.10
-183.12
-183.13
-183.13
-183.14
-183.15
-183.17
-183.18
-183.19
-183.20
-183.21
-183.22
-183.24
19F NMR
F
F
N
Piv
in CDCl3
-105 -110 -115 -120 -125 -130 -135 -140 -145 -150 -155 -160 -165 -170 -175 -180 -185 -190 -195 -200 -205 -210 -215 -220 -225 -230 -235 -240 -245
f1 (ppm)

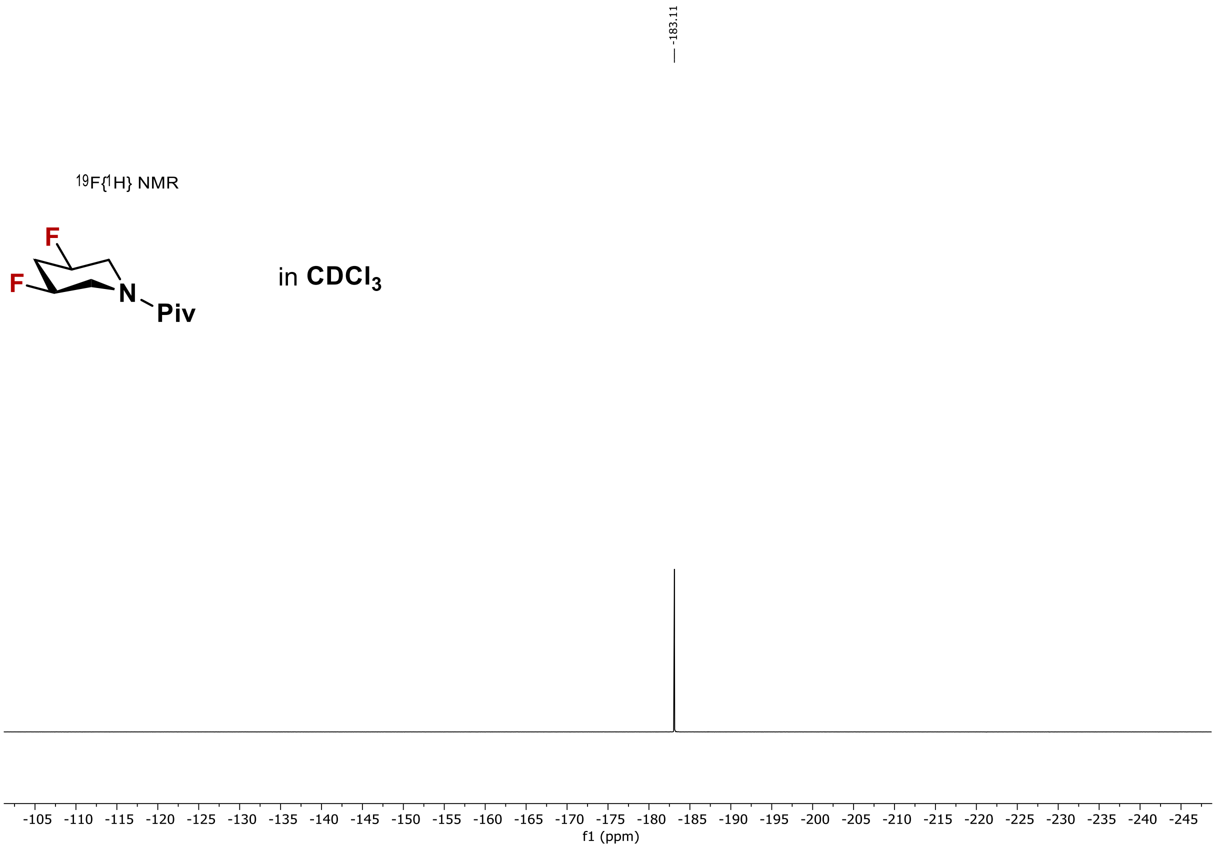

-183.11
19F{1H} NMR
F
F
N
Piv
in CDCl3
-105 -110 -115 -120 -125 -130 -135 -140 -145 -150 -155 -160 -165 -170 -175 -180 -185 -190 -195 -200 -205 -210 -215 -220 -225 -230 -235 -240 -245
f1 (ppm)

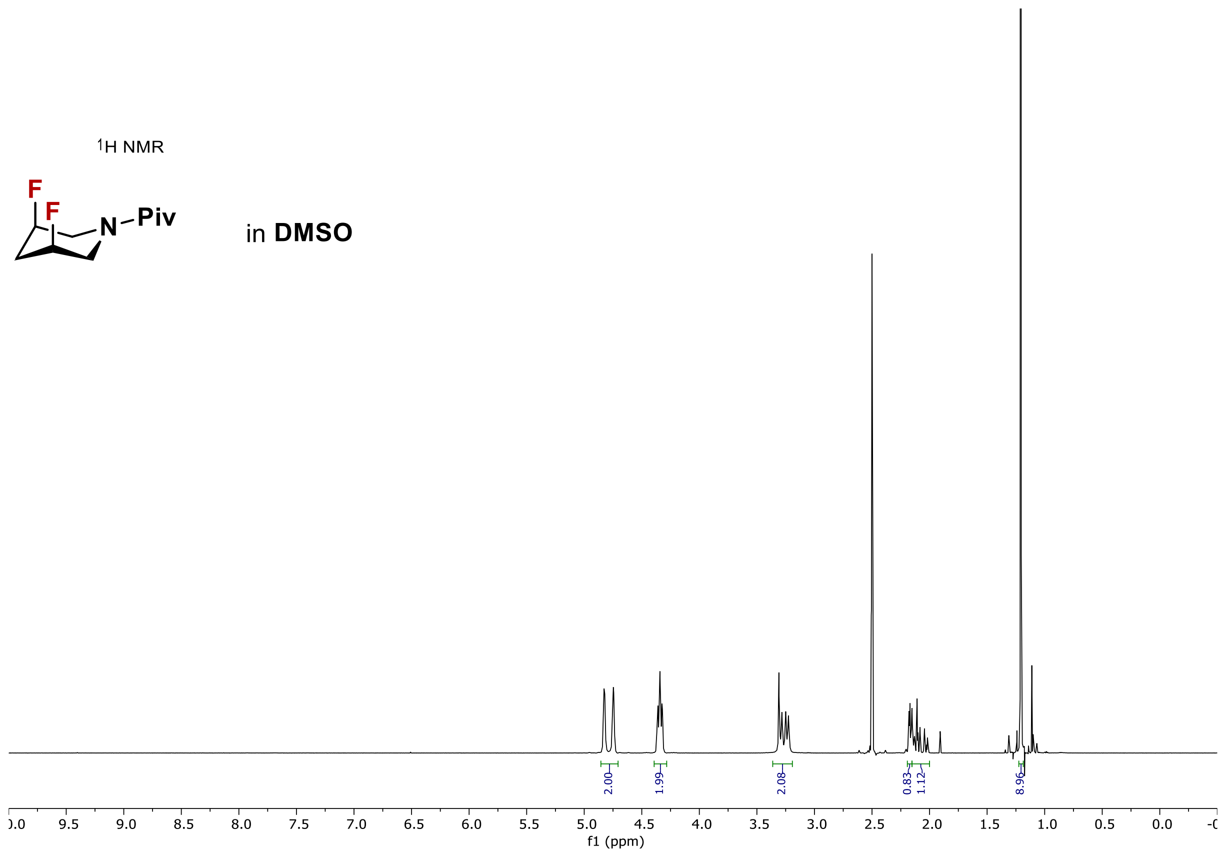
1H NMR
F
F
N
Piv
in DMSO
2.00
1.99
2.08
0.83
1.12
8.96
f1 (ppm)

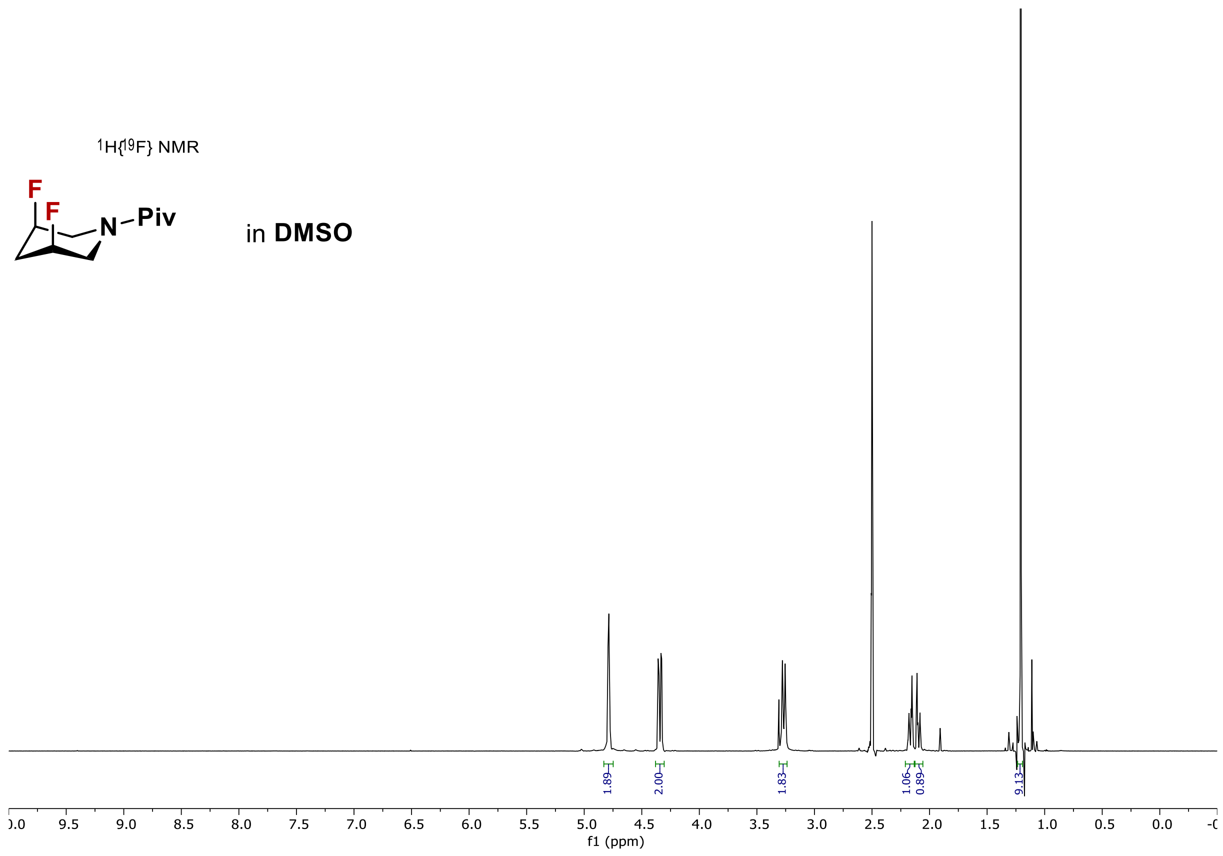
1H{19F} NMR
F
F
N
Piv
in DMSO
1.89
2.00
1.83
1.06
0.89
9.13
f1 (ppm)

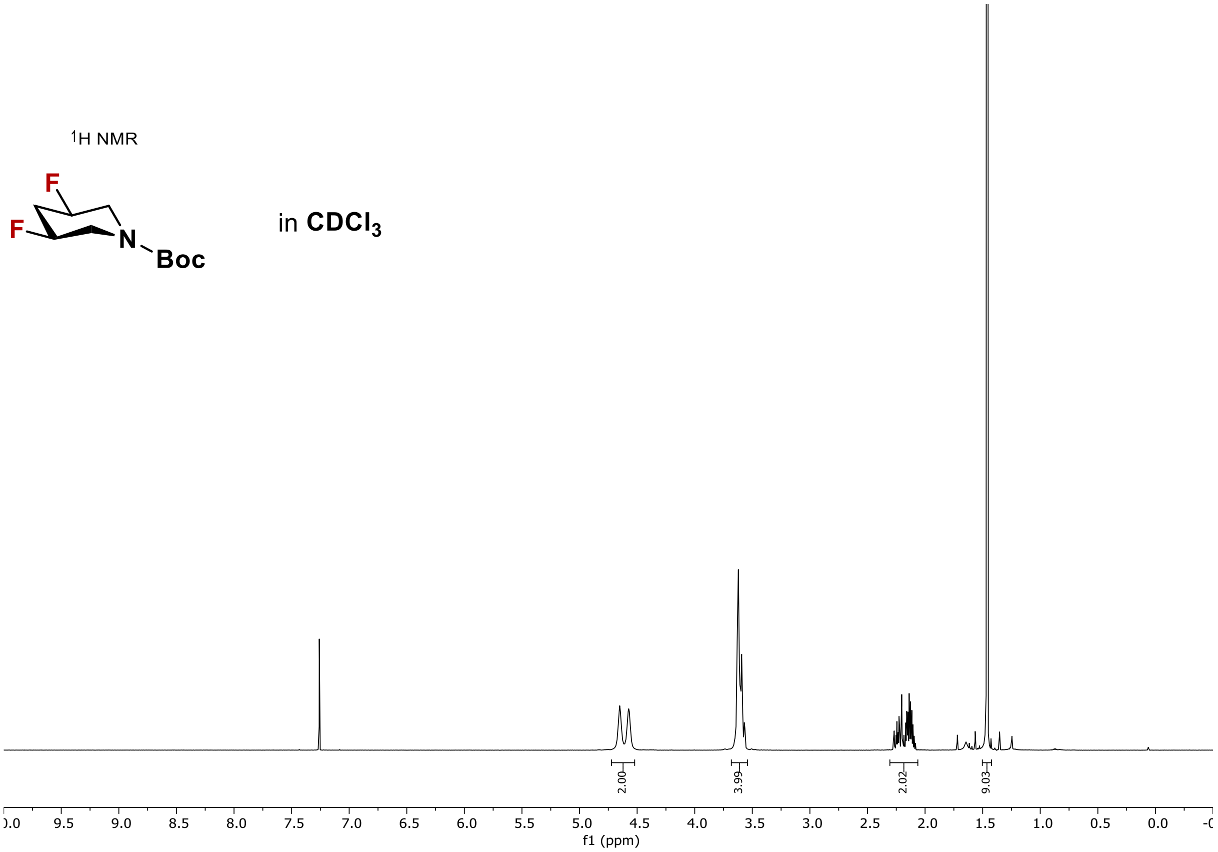
1H NMR
F
F
N
Boc
in CDCl3
2.00
3.99
2.02
9.03
9.5 9.0 8.5 8.0 7.5 7.0 6.5 6.0 5.5 5.0 4.5 4.0 3.5 3.0 2.5 2.0 1.5 1.0 0.5 0.0
f1 (ppm)

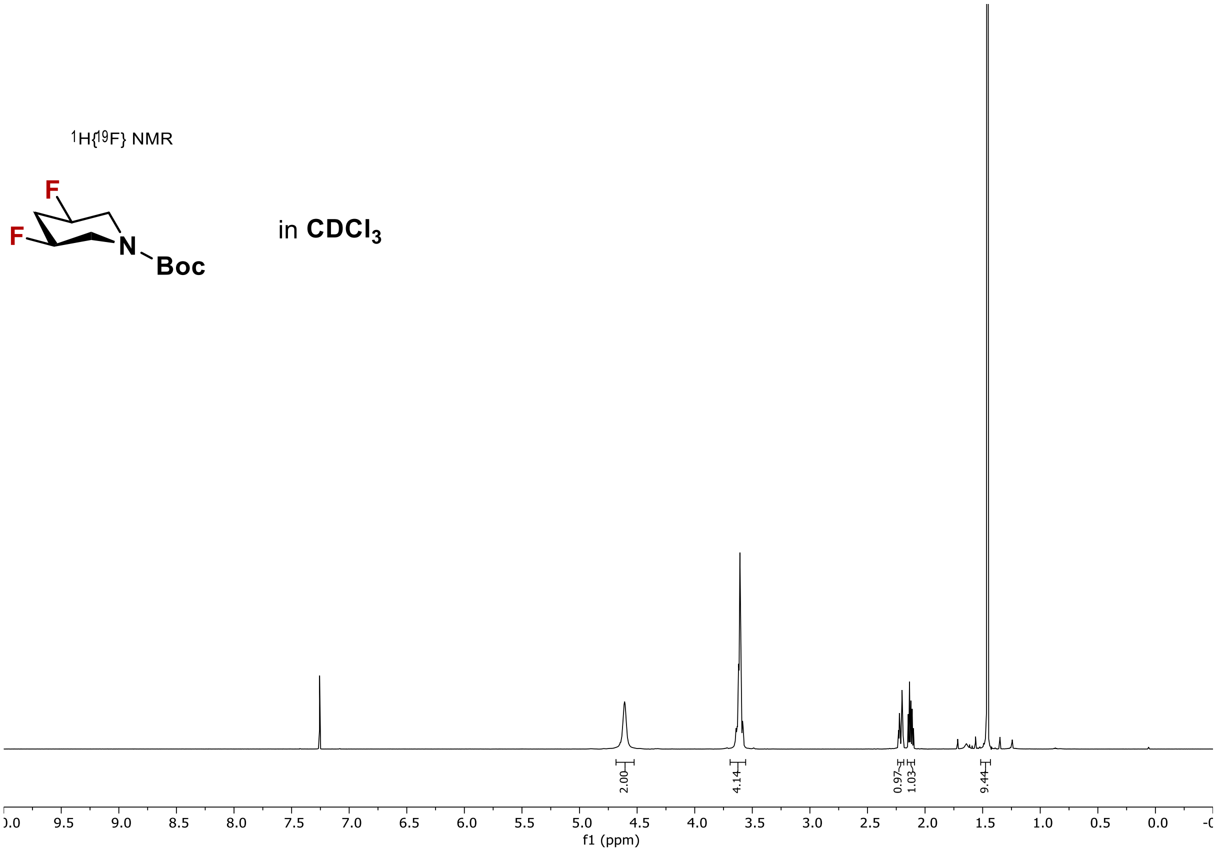
1H{19F} NMR
F
F
N
Boc
in CDCl3
2.00
4.14
0.97
1.03
9.44
9.5 9.0 8.5 8.0 7.5 7.0 6.5 6.0 5.5 5.0 4.5 4.0 3.5 3.0 2.5 2.0 1.5 1.0 0.5 0.0
f1 (ppm)

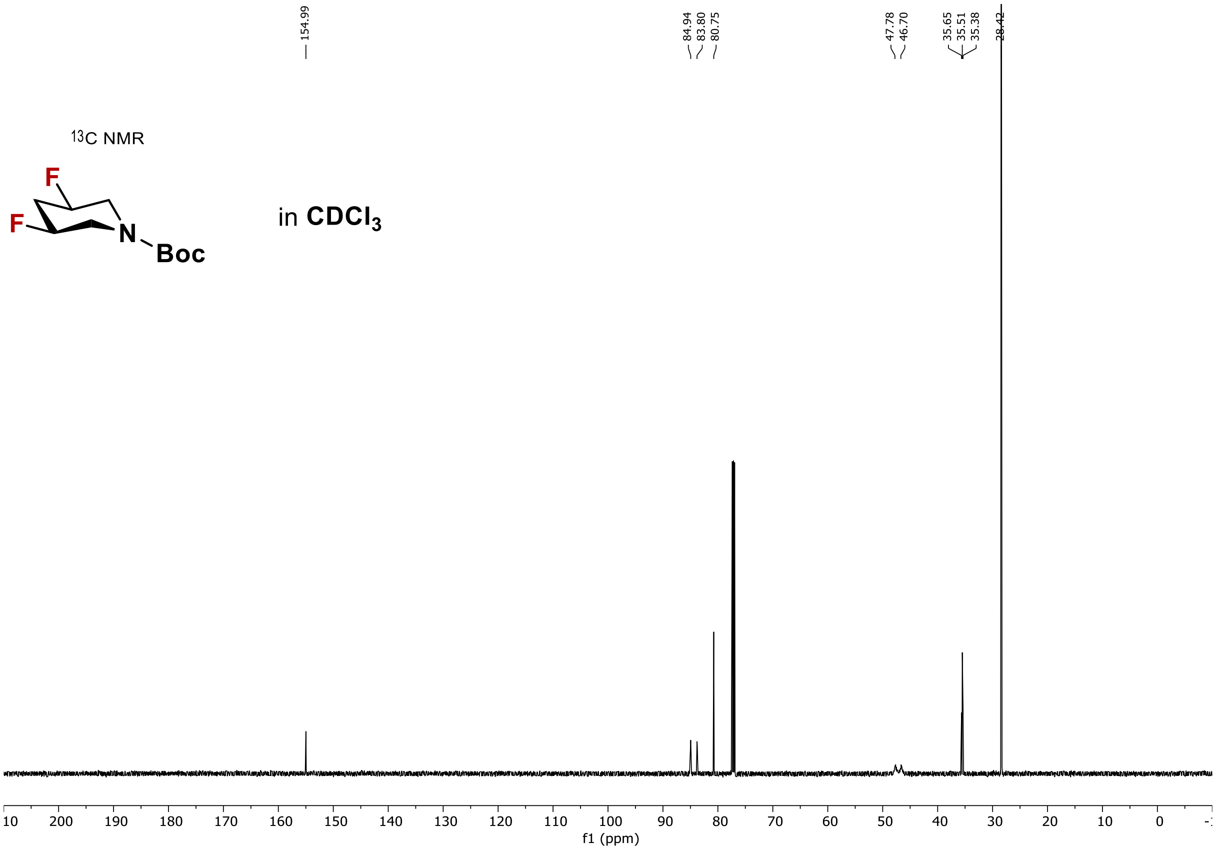

154.99
84.94
83.80
80.75
47.78
46.70
35.65
35.51
35.38
28.42
13C NMR
F
F
N
Boc
in CDCl3
f1 (ppm)

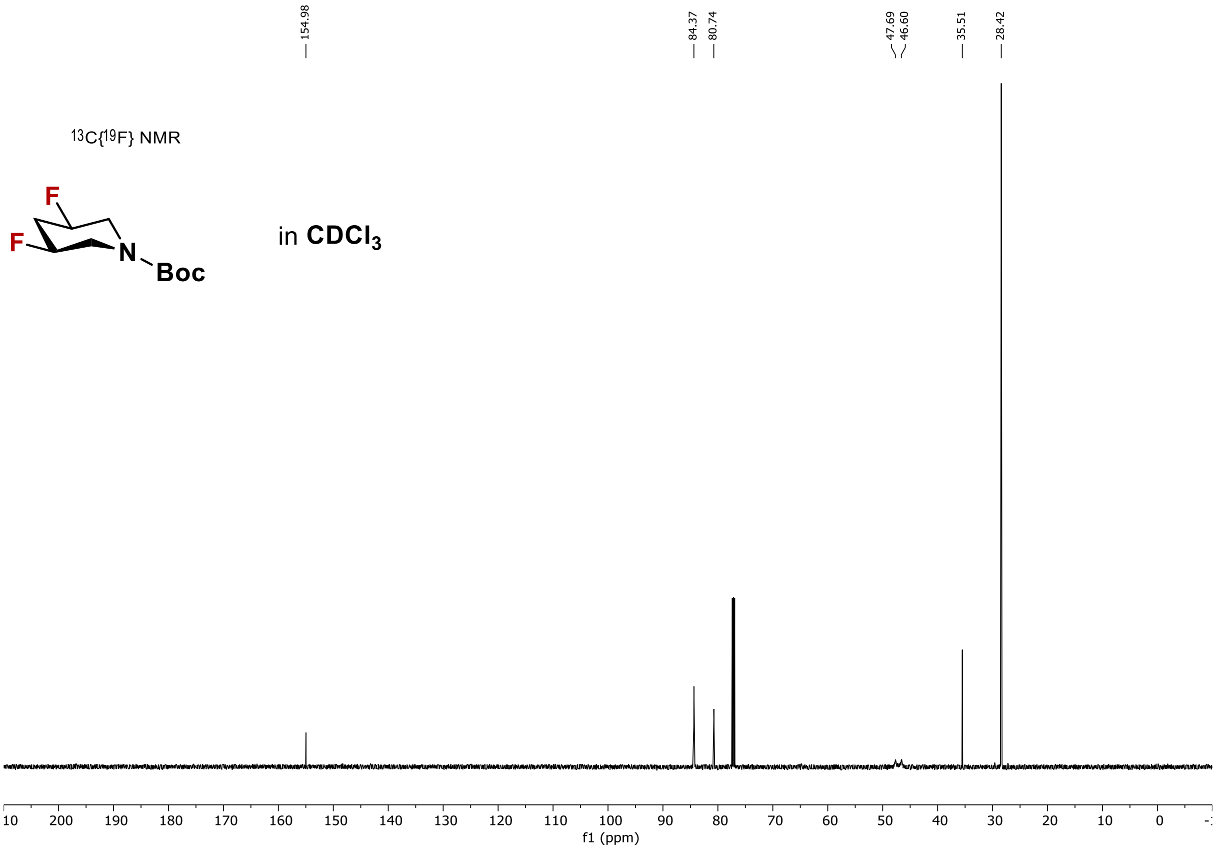

154.98
84.37
80.74
47.69
46.60
35.51
28.42
13C{19F} NMR
F
F
N
Boc
in CDCl3
f1 (ppm)

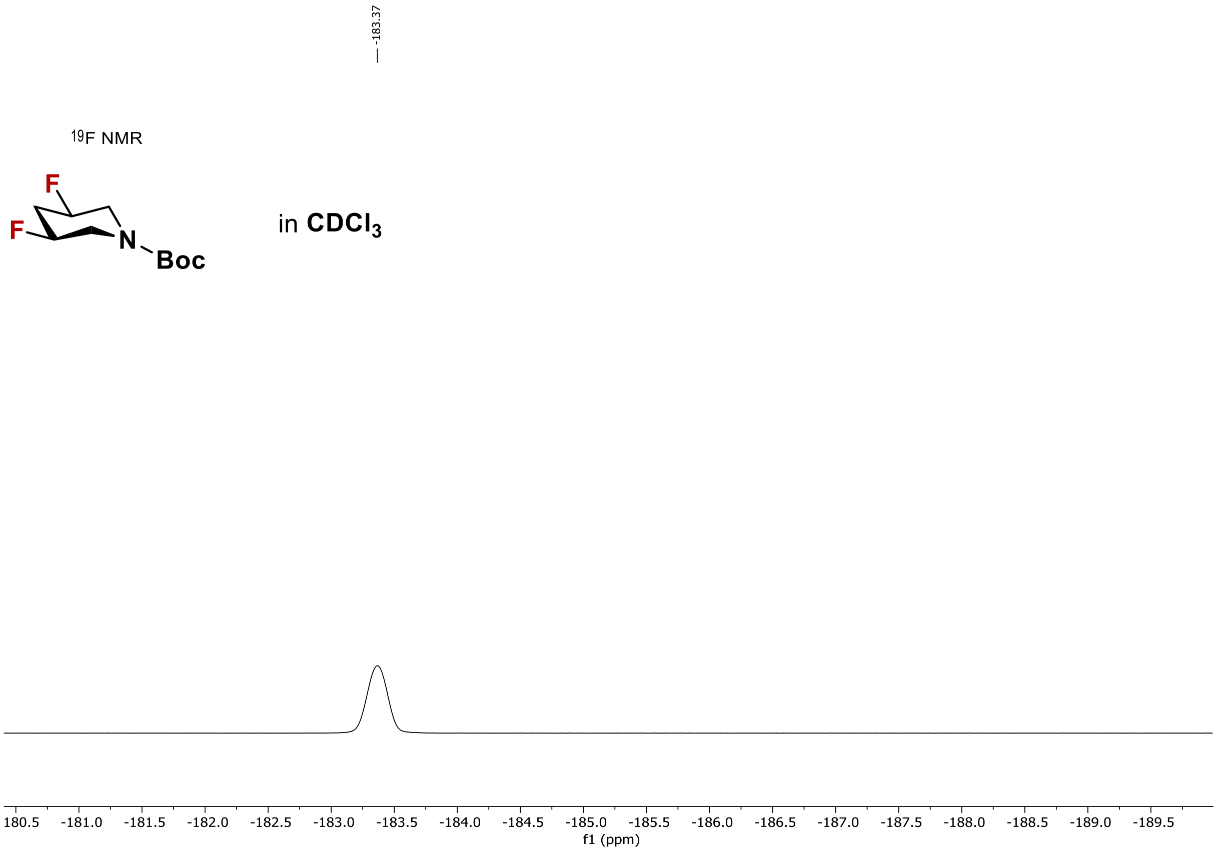

-183.37
19F NMR
F
F
N
Boc
in CDCl3
180.5 -181.0 -181.5 -182.0 -182.5 -183.0 -183.5 -184.0 -184.5 -185.0 -185.5 -186.0 -186.5 -187.0 -187.5 -188.0 -188.5 -189.0 -189.5
f1 (ppm)

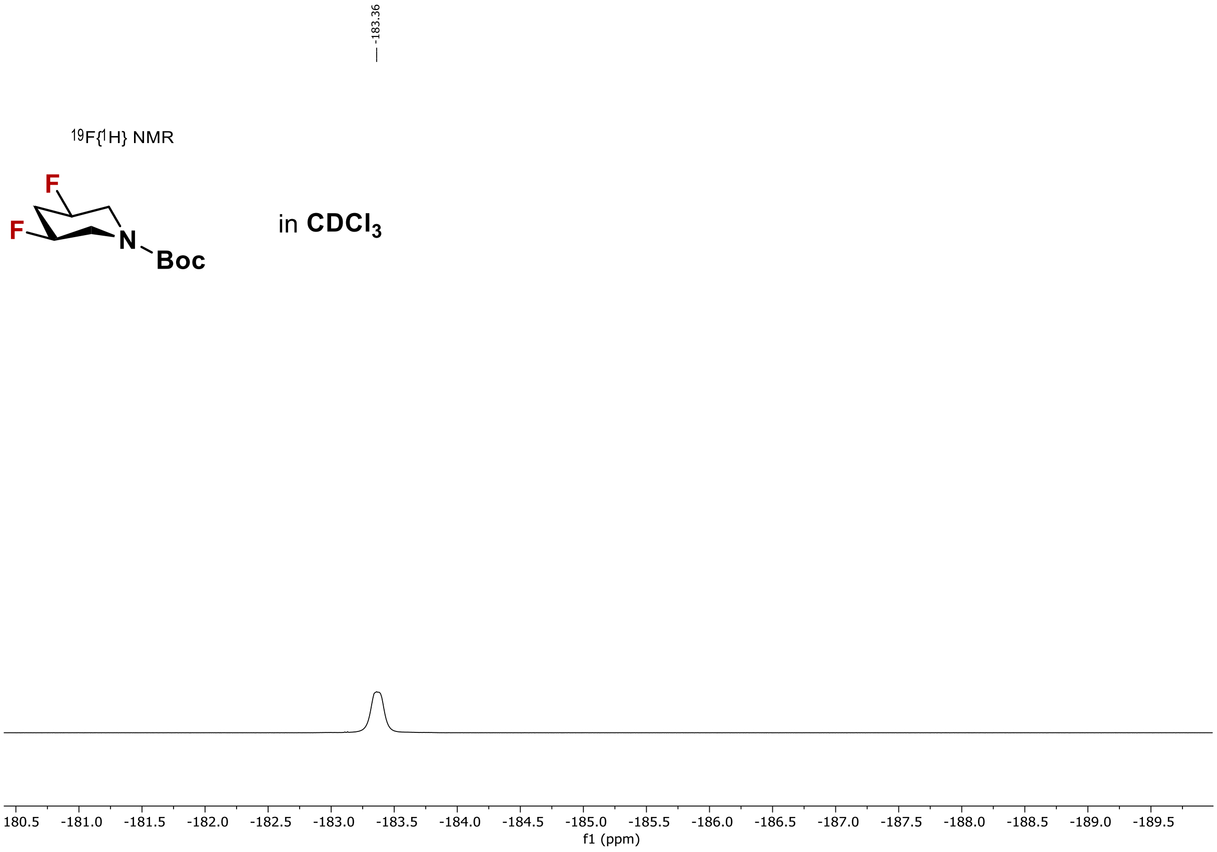

-183.36
19F{1H} NMR
F
F
N
Boc
in CDCl3
180.5 -181.0 -181.5 -182.0 -182.5 -183.0 -183.5 -184.0 -184.5 -185.0 -185.5 -186.0 -186.5 -187.0 -187.5 -188.0 -188.5 -189.0 -189.5
f1 (ppm)

^{1}H NMR

in **DMSO**

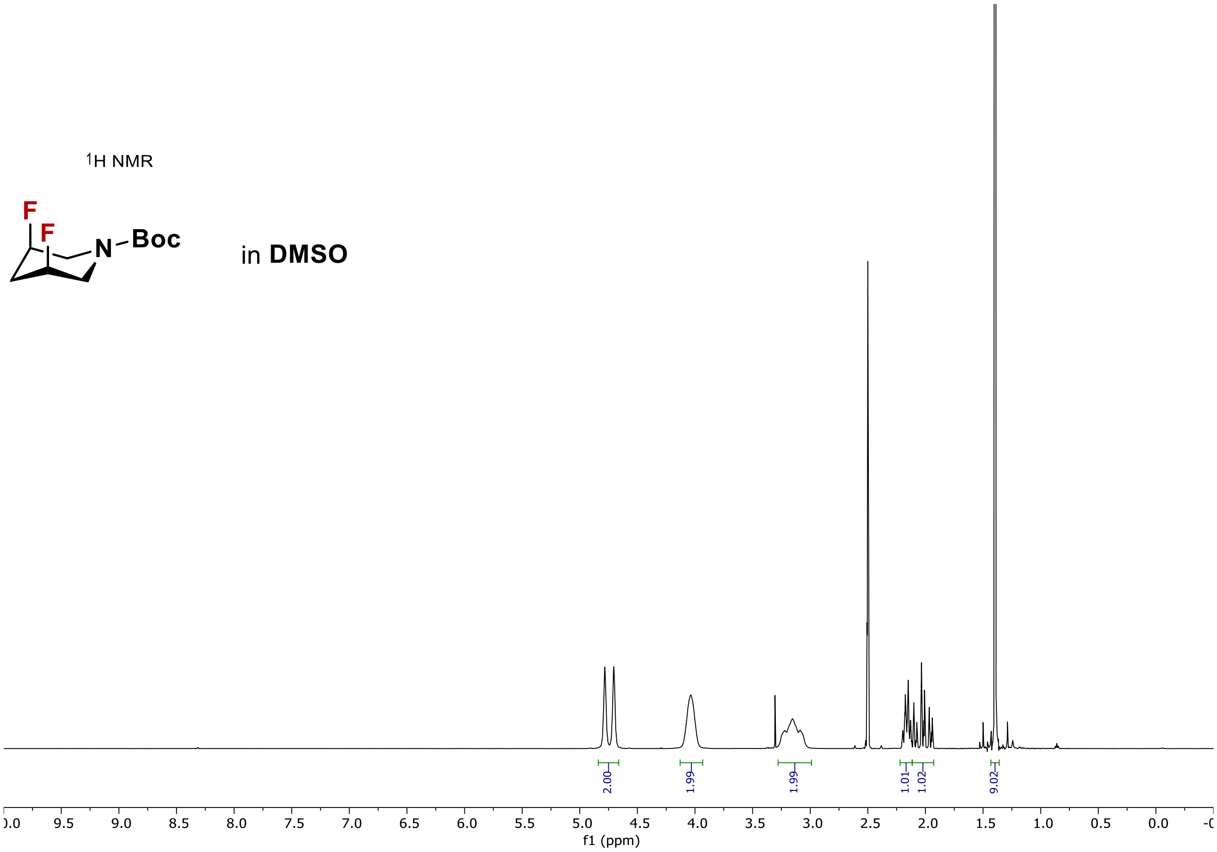

$^{1}H\{^{19}F\}$ NMR

in **DMSO**

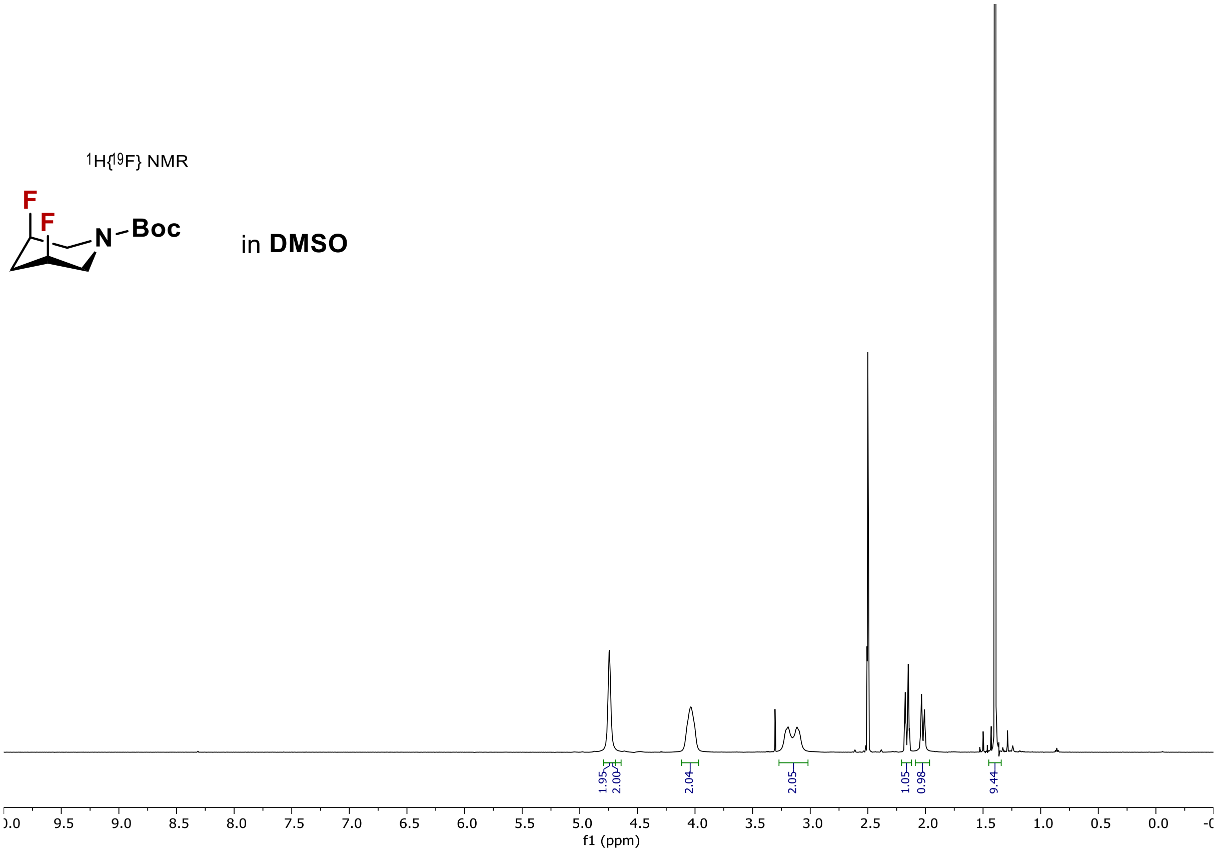

Supplement: Supplementary file 1 — Supplementary [file CHEM-26-6141-s001.pdf]
